# Causal relationships between blood cell perturbation responses, immune cell phenotypes, and cardiomyopathy: A two-sample Mendelian randomization and mediation analysis

**DOI:** 10.1097/MD.0000000000048404

**Published:** 2026-04-17

**Authors:** Kang Xu, Lanxin Ma, Hongfei Du, Huacui Huang, Ying Xu

**Affiliations:** a Department of Clinical Laboratory, People’s Hospital of Xindu District, Chengdu, China; b Precision Medicine Center, People’s Hospital of Chuxiong Yi Autonomous Prefecture, Chuxiong, China; c Department of Clinical Laboratory, The First Affiliated Hospital of Chengdu Medical College, Chengdu, China.

**Keywords:** blood cell perturbation, cardiomyopathy, immune cells, mediation analysis, Mendelian randomization

## Abstract

Clinical investigations have demonstrated that blood and immune cells are involved in the pathophysiological processes of dilated cardiomyopathy (DCM) and hypertrophic cardiomyopathy (HCM). However, the causal relationships between these cellular components and the development of DCM and HCM remain uncertain. A two-sample Mendelian randomization analysis was performed to evaluate the causal effects of blood cells and immune cells on DCM and HCM. The primary analytical method was inverse variance weighting, supplemented by Mendelian randomization-Egger, weighted median, and MR-PRESSO approaches. Furthermore, immune cells were examined as mediators to assess their intermediary roles in the causal pathways linking blood cells with DCM and HCM. Significant causal associations were observed between red blood cells, monocytes, eosinophils, and platelets and DCM and HCM (*P* < .05). CD127 on CD8br, naive CD8br %CD8br, CD16− CD56 on natural killer cell, CD45 on T cell, and lymphocyte AC were identified as mediators in the causal pathways connecting various blood cell types to DCM and HCM. This study provides robust evidence for the causal roles of specific blood cell and immune cell phenotypes in the development of DCM and HCM. These findings open new avenues for investigating the hematological immune system in cardiomyopathy and present novel opportunities for therapeutic interventions targeting DCM and HCM.

## 1. Introduction

Cardiomyopathy is defined as a condition involving structural and functional abnormalities of the myocardium, in the absence of coronary artery disease, hypertension, valvular disease, or congenital heart disease that is sufficient to explain the observed myocardial dysfunction.^[1]^ It encompasses several subtypes, including dilated cardiomyopathy (DCM), hypertrophic cardiomyopathy (HCM), non-dilated left ventricular cardiomyopathy, arrhythmogenic right ventricular cardiomyopathy, and restrictive cardiomyopathy.^[1]^ The incidence rates of DCM and HCM are approximately 1/250 and 1/500, respectively, making them the most common types of cardiomyopathy.^[2,3]^ The characteristic feature of DCM is the dilation of 1 or both ventricles accompanied by impaired systolic function, and major symptoms include heart failure (HF), arrhythmias, and sudden cardiac death.^[4]^ The most common feature of HCM is left ventricular hypertrophy, with potential outcomes including HF and sudden cardiac death.^[3]^ DCM and HCM pose significant threats to human life, therefore, thorough research and identification of their potential risk factors for the prevention of these conditions are imperative.

Traditionally, the understanding of the immune system is that the body protects itself from pathogens by recognizing self and nonself components. Inflammation is tissue damage caused by inflammatory factors, which may be aseptic or caused by pathogenic infections.^[5]^ Under physiological conditions, distinct populations of immune cells are distributed across various regions of the heart, and pathological processes may alter the distribution, subtypes, and polarization of these resident immune cells.^[6]^ Research has confirmed the involvement of immune cells in both HF and myocardial infarction.^[7–10]^ However, these studies have not established a causal relationship between immune cells and cardiac diseases, including cardiomyopathy.

Blood cells are critical components of the circulatory system and include red blood cells, white blood cells, and platelets. They play essential roles in gas transport and exchange, immune defense, and hemostasis. Studies have shown that neutrophil extracellular traps in myocardial tissue can directly impair mitochondrial function in cardiomyocytes, thereby contributing to diminished cardiac contractility and an increased incidence of adverse cardiac events in patients with DCM.^[11]^ In cases of idiopathic DCM, circulating leukocytes become activated and migrate toward the heart, inducing stress responses and apoptosis in cardiomyocytes.^[12]^ However, further research is warranted to elucidate the causal relationship between blood cell perturbations and cardiomyopathies, as well as the involvement of immune cell dynamics.

Mendelian randomization (MR) is an epidemiological approach employed to explore causal relationships between risk factors and outcomes via observational data.^[13]^ Genetic variants, primarily single nucleotide polymorphisms (SNPs), serve as proxies for these risk factors, allowing assessment of their associations with prognosis.^[14]^ Given that alleles are randomly distributed from parents to offspring and that their genetic composition is stable, MR is expected to be resistant to confounding factors and reverse causation, provided that conditions are appropriate. In this study, SNP data derived from genome-wide association studies (GWAS) were utilized as instrumental variables for exposure, and a two-sample MR approach was applied to investigate the associations between blood cell perturbation responses, immune phenotypes, and both DCM and HCM.

## 2. Methods

### 2.1. Study design

**Figure 1.**
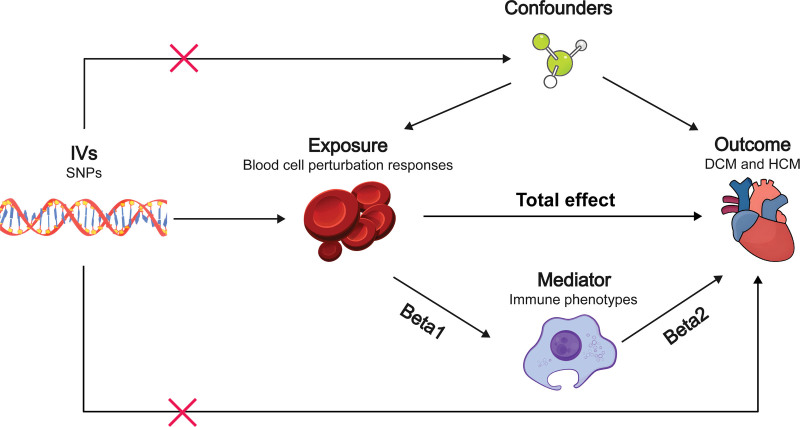
Study overview. First, two-sample MR analysis was conducted to investigate the causal relationship between the blood cell perturbation response and DCM/HCM, yielding the total effect. Next, immune phenotypes were selected for subsequent mediation analysis. Finally, two-sample MR analyses were carried out to assess the causal relationship between blood cell perturbation responses and immune phenotypes, as well as between immune phenotypes and DCM/HCM, yielding Beta1 and Beta2, respectively, followed by mediation analysis. DCM = dilated cardiomyopathy, HCM = hypertrophic cardiomyopathy, MR = Mendelian randomization.

This study adheres to the Strengthening the Reporting of Observational Studies in Epidemiology Using Mendelian Randomization: The STROBE-MR Statement guidelines.^[15]^ Two-step MR analysis was conducted to identify potential causal relationships between blood cells and cardiomyopathies, and to determine whether immune cells mediate these associations. The first step involved assessing the causal effects of blood cells and immune cells on cardiomyopathies through two-sample MR, thereby identifying blood cell and immune cell traits associated with cardiomyopathies. The second step evaluated the causal effects of the selected blood cell traits on the selected immune cell traits and calculated the proportion of the effect of blood cells on cardiomyopathies that were mediated through each immune cell phenotype. SNPs were used as instrumental variables (IVs). MR relies on 3 core assumptions: IVs are strongly associated with exposure; IVs are not associated with confounding factors; and IVs influence the outcome exclusively through exposure.^[16]^ Ethical approval was not required for this study, as the included GWAS had already received the necessary ethical clearances. The overall research design is schematically presented in Figure 1.

### 2.2. Data sources

The GWAS Catalog has become one of the most extensive freely available resources for GWAS summary statistics, comprising over 45,000 published GWASs and >40,000 complete datasets of *P*-value summary statistics for variant-trait associations, alongside supporting metadata for over 5000 human traits.^[17]^ The blood cell perturbation data (accession numbers GCST90257015–GCST90257105) were derived from the study by Homilius et al,^[18]^ in which physical, chemical, and pharmacological perturbations were applied to peripheral blood samples from 2600 individuals of European ancestry. GWASs were then performed on the induced traits. This study identified a total of 119 genomic loci involving 96 genes associated with these cellular responses and further revealed associations between the induced blood phenotypes and subtypes of common diseases.

We sourced data on immune phenotypes for GWASs from the GWAS catalog (accession numbers GCST90001391–GCST90002121). The GWAS dataset comprises 731 immune phenotypes, which include 118 absolute cell counts, 389 median fluorescence intensities of surface antigens, 32 morphological parameters, and 192 relative counts. The initial GWAS on immune characteristics utilized data from 3757 Europeans in distinct cohorts. Employing a Sardinian sequence as the reference panel, the analysis calculates approximately 22 million SNP genotypes from high-density arrays and assesses correlations after adjusting for covariates.^[19]^

GWAS data for DCM and HCM were derived from a comprehensive cross-population genetic association study (accession numbers GCST90018834 and GCST90018861).^[20]^ This study identified 2768 genome-wide significant loci, 394 of which were novel (*P* < 5.0 × 10^−8^). The full details of the GWAS datasets are provided in Table S1, Supplemental Digital Content, https://links.lww.com/MD/R740.

### 2.3. Selection of IVs

According to prior studies, the significance level for each immune phenotype in terms of IVs is established at 1 × 10^-5^.^[21,22]^ This relaxed threshold is typically used when there is a low count of genome-wide significant SNPs.^[23]^ The clumping procedure was performed by linkage disequilibrium analysis with a 10,000 kb distance within a *r*^2^ threshold <0.001. The IVs for blood cell perturbation and DCM and HCM adhere to the same criteria. To determine the robustness of these IVs, the *F* statistic for each IV is calculated via the following formula: *F* = *R*^2^ × (N − 2)/(1 − *R*^2^); *R*^2^ = 2 × EAF × (1 − EAF) × β^2^.^[24]^ In this formula, *R*^2^ refers to the explained genetic variation in IVs, N is the sample size, EAF is the allele frequency of the effect, and β represents the estimated effect of the SNP. Typically, IVs with an *F* statistic <10 are deemed weak and are subsequently excluded.

### 2.4. Statistical analysis

#### 2.4.1. MR analysis

A variety of MR methods have been employed to assess the causal relationships between blood cell perturbation responses, immune phenotypes, and DCM and HCM. These methods include inverse variance weighted (IVW),^[25]^ weighted median,^[26]^ MR-Egger regression,^[27]^ weighted mode, and simple mode, with the IVW model serving as the primary standard approach for MR analysis. In addition, reverse MR analysis was conducted using DCM and HCM as exposures, and blood cell perturbation responses and immune phenotypes were used as outcomes. This approach facilitates the evaluation of potential feedback loops between disease risk and blood cell or immune cell traits, which is critical for identifying and mitigating false-positive findings.

#### 2.4.2. Sensitivity and heterogeneity analysis

To evaluate the model’s accuracy and reliability, we performed a sensitivity analysis that included assessments of heterogeneity and pleiotropy. To analyze heterogeneity, Cochran *Q* statistic was utilized.^[28]^ We applied the widely accepted MR-Egger regression to evaluate horizontal pleiotropy, identifying minimal pleiotropy when the intercept approaches 0 and *P *< .05.^[27]^ MR-PRESSO was then employed to remove potential outlier values that could influence pleiotropy outcomes.^[29]^ Scatter plots and funnel plots were employed to visually depict the robustness of the findings.

#### 2.4.3. Mediation analysis

To determine whether immune cell phenotypes act as potential mediators between blood cell perturbations and the development of DCM and HCM, we performed a mediation analysis. First, a two-sample MR approach was used to estimate the causal effect of blood cell perturbations on immune cell phenotypes, yielding Beta1. Next, the same method was applied to evaluate the causal relationships between immune cell phenotypes and DCM or HCM, resulting in Beta2. The total effect was derived by analyzing the causal association between blood cell perturbations and DCM/HCM. The mediated effect and proportion were calculated via the following formulas: Mediated Effect = Beta1 × Beta2; Mediated Proportion = (Mediated Effect/Total Effect) × 100%.^[30]^

All MR analyses were performed via the “TwoSampleMR”^[31]^ (MRC Integrative Epidemiology Unit, Bristol, United Kingdom) R package (version 0.5.8).

## 3. Results

### 3.1. The causal effect of blood cell perturbation responses on DCM and HCM

**Figure 2.**
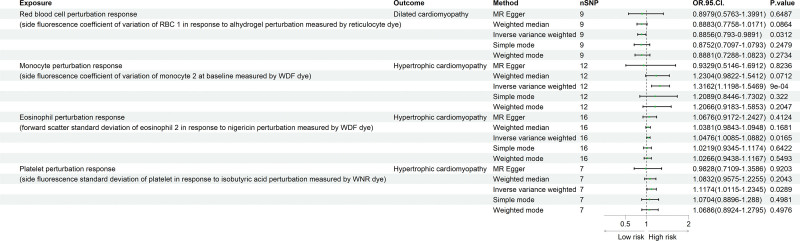
Forest plot generated via different methods showing a causal relationship between blood cell perturbation responses and DCM and HCM. DCM = dilated cardiomyopathy, HCM = hypertrophic cardiomyopathy, WDF = white blood cell differential, WNR = white blood cell, basophils, and nucleated red blood cell.

In the investigation of the causal relationships between blood cell perturbation responses and DCM/HCM, our findings indicate that perturbations in red blood cells are a protective factor for DCM (odds ratio [OR] = 0.885, 95% confidence interval [CI] = 0.793–0.989, *P* = .031). Conversely, perturbations in monocytes (OR = 1.316, 95% CI = 1.119–1.546, *P* < .001), eosinophils (OR = 1.047, 95% CI = 1.008–1.088, *P* = .016), and platelets (OR = 1.117, 95% CI = 1.011–1.234, *P* = .028) were identified as risk factors for HCM. These results are illustrated in Figure 2. Reverse MR analysis revealed no evidence of reverse causality between blood cell perturbation responses and either DCM or HCM. More detailed results are provided in Table S2, Supplemental Digital Content, https://links.lww.com/MD/R740.

### 3.2. The causal effect of immune phenotypes on DCM and HCM

**Figure 3.**
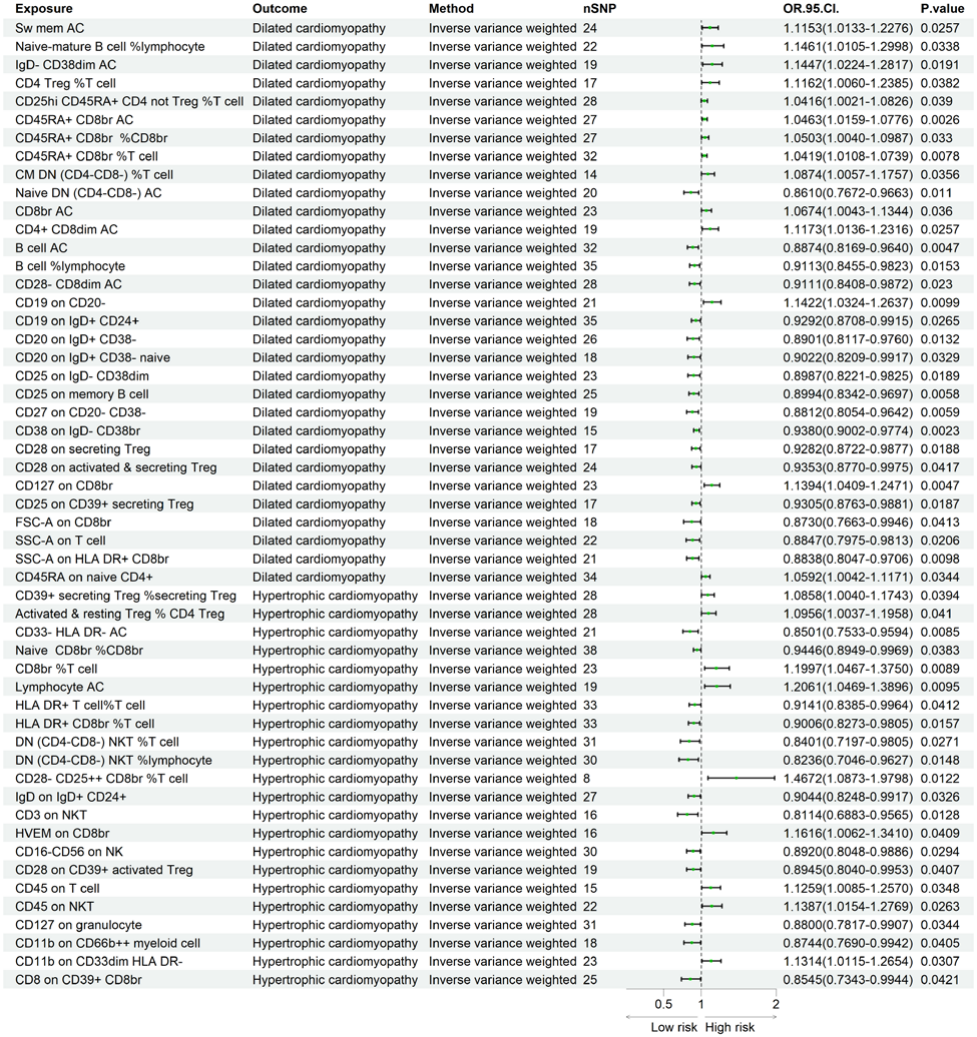
Forest plot generated via the IVW method showing a causal relationship between immune phenotypes and DCM and HCM. DCM = dilated cardiomyopathy, HCM = hypertrophic cardiomyopathy, IVW = inverse variance weighted.

Our results revealed 31 immune cell phenotypes significantly associated with DCM (*P* < .05), including 11 B cell traits, 7 TBNK traits, 7 Treg cell traits, and 6 traits related to the maturation stages of T cells. Additionally, 22 immune cell phenotypes were found to be associated with HCM (*P* < .05), including 10 TBNK traits, 6 Treg cell traits, 3 myeloid cell traits, 2 traits related to T cell maturation stages, and 1 B cell trait. Among them, naive-mature B cell %lymphocyte (OR = 1.146, 95% CI = 1.010–1.299, *P* = .033) emerged as the strongest risk factor for DCM, whereas naive DN (CD4−CD8−) AC (OR = 0.861, 95% CI = 0.767–0.966, *P* = .011) represented the most protective factor against DCM. For HCM, the strongest risk factor was CD28−CD25++ CD8br% T cell (OR = 1.467, 95% CI = 1.087–1.979, *P* = .012), while CD3 on NKT (OR = 0.811, 95% CI = 0.688–0.956, *P* = .012) was the most protective factor. All the results are illustrated in Figure 3. Reverse MR analysis demonstrated no evidence of reverse causality between immune cell phenotypes and either DCM or HCM. More detailed results are provided in Table S3, Supplemental Digital Content, https://links.lww.com/MD/R740.

### 3.3. The causal effect of identified blood cell perturbation responses on identified immune phenotypes

**Figure 4.**
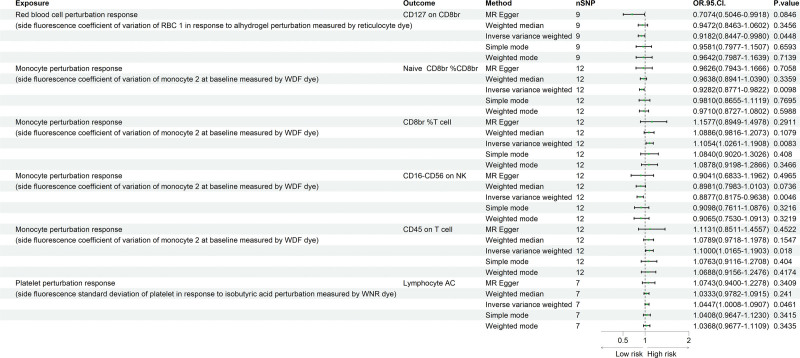
Forest plot generated via different methods showing a causal relationship between blood cell perturbation responses and identified immune phenotypes.

Following two-sample MR analyses between the selected blood cell perturbation responses and the selected immune cell phenotypes, we found that the red blood cell perturbation response was negatively associated with CD127 on CD8br (OR = 0.918, 95% CI = 0.844–0.998, *P* = .044). The monocyte perturbation response was positively associated with CD8br %T cell (OR = 1.105, 95% CI = 1.02 6–1.190, *P* = .037) and CD45 on T cell (OR = 1.099, 95% CI = 1.016–1.190, *P* = .017), and negatively associated with naive CD8br %CD8br (OR = 0.928, 95% CI = 0.877–0.982, *P* = .009) and CD16− CD56 on natural killer (NK) cells (OR = 0.887, 95% CI = 0.817–0.963, *P* = .004). The platelet perturbation response was positively associated with lymphocyte AC (OR = 1.044, 95% CI = 1.000–1.090, *P* = .046). These findings are illustrated in Figure 4. More detailed results are provided in Table S4, Supplemental Digital Content, https://links.lww.com/MD/R740.

### 3.4. Sensitivity analyses and detection of potential pleiotropy

MR-Egger regression analysis revealed no evidence of pleiotropy, and the funnel plot results confirmed this finding. The Cochran *Q* test did not indicate significant heterogeneity among immune phenotype SNPs. The leave-one-out method was used to analyze whether the IVW results of the remaining SNPs were similar to the original results by progressively excluding each SNP to identify outliers, and our results did not reveal that any SNP had a significant impact on the causal relationship results (Figs. S1–S12, Supplemental Digital Content, https://links.lww.com/MD/R742). The MR-PRESSO global test indicated no significant outliers, further confirming the robustness of the results (Tables S5–S7, Supplemental Digital Content, https://links.lww.com/MD/R740).

### 3.5. Mediation analysis results

Through mediation analysis, we explored the mediating role of immune cell phenotypes in the causal pathways from blood cell perturbations to DCM and HCM. The results indicated that CD127 on CD8br (total effect = –0.121, direct effect = –0.110) had a negative mediating effect on the pathway between the red blood cell perturbation response and DCM, with a mediation proportion of 9.17%. Furthermore, naive CD8br %CD8br (total effect = 0.274, direct effect = 0.270), CD8br %T cell (total effect = 0.274, direct effect = 0.256), CD16− CD56 on NK cells (total effect = 0.274, direct effect = 0.261), and CD45 on T cell (total effect = 0.274, direct effect = 0.263) positively mediated monocyte perturbation response and HCM, with percentages of 1.55%, 6.64%, 4.96%, and 4.11%, respectively. The lymphocyte AC (total effect = 0.111, direct effect = 0.102) had a positive mediating effect on the pathway between the platelet perturbation response and HCM, with a mediation proportion of 7.39%. These results are summarized in Table 1.

**Table 1:** Mediation effects of blood perturbation responses on DCM/HCM via immune phenotypes.

Exposure	Mediator	Outcome	Total effect	Direct effect	Mediation effect (95% CI)	Mediation Proportion
Red blood cell perturbation response (side fluorescence coefficient of variation of RBC 1 in response to alhydrogel perturbation measured by reticulocyte dye)	CD127 on CD8br	Dilated cardiomyopathy	‐0.1214	‐0.1103	‐0.0111 (‐0.0249 to 0.0026)	9.17%
Monocyte perturbation response (side fluorescence coefficient of variation of monocyte 2 at baseline measured by WDF dye)	Naive CD8br %CD8br	Hypertrophic cardiomyopathy	0.2747	0.2705	0.0043 (‐0.0010 to 0.0095)	1.55%
Monocyte perturbation response (side fluorescence coefficient of variation of monocyte 2 at baseline measured by WDF dye)	CD8br %T cell	Hypertrophic cardiomyopathy	0.2747	0.2565	0.0182 (‐0.0077, 0.0442)	6.64%
Monocyte perturbation response (side fluorescence coefficient of variation of monocyte 2 at baseline measured by WDF dye)	CD16-CD56 on NK	Hypertrophic cardiomyopathy	0.2747	0.2611	0.0136 (‐0.0017 to 0.0289)	4.96%
Monocyte perturbation response (side fluorescence coefficient of variation of monocyte 2 at baseline measured by WDF dye)	CD45 on T cell	Hypertrophic cardiomyopathy	0.2747	0.2634	0.0113 (‐0.0038 to 0.0264)	4.11%
Platelet perturbation response (side fluorescence standard deviation of platelet in response to isobutyric acid perturbation measured by WNR dye)	Lymphocyte AC	Hypertrophic cardiomyopathy	0.1110	0.1028	0.0082 (‐0.0184 to 0.0348)	7.39%

CI = confidence interval, HCM = hypertrophic cardiomyopathy, NK = natural killer, RBC = red blood cell.

## 4. Discussion

This study employed MR analysis to evaluate the causal relationships among blood cell perturbations, immune cell phenotypes, and both DCM and HCM. We identified 1 blood cell perturbation and 31 immune cell phenotypes associated with DCM, as well as 3 blood cell perturbations and 22 immune cell phenotypes associated with HCM. Furthermore, mediation analysis revealed that immune cell phenotypes play a mediating role in the pathogenic processes linking blood cell perturbations to the development of DCM and HCM.

DCM is caused by multiple etiologies, including genetic variations, epigenetic disorders, infectious damage, autoimmune diseases, and cardiac conduction abnormalities. Oxidative stress is a key contributor to the development of DCM.^[32–34]^ In mice lacking superoxide dismutase 2, red blood cell oxidative stress is elevated, and homozygous mutants with superoxide dismutase 2 gene inactivation die of DCM within 10 days after birth.^[35,36]^ Aluminum accumulation in the blood can induce toxic effects, resulting in structural alterations and dysfunction of red blood cells, ultimately leading to hematological disorders.^[37]^ Specifically, this manifests as a reduction in red blood cell count, an increase in microcytic red blood cells, accompanied by a decrease in mean corpuscular hemoglobin, and a decline in osmotic fragility similar to that seen in iron deficiency anemia.^[38]^ These changes suggest alterations in the cholesterol-to-phospholipid ratio of the red blood cell membrane. Additionally, severe oxidative stress is observed.^[39]^ Our findings revealed that the side fluorescence coefficient of variation in reticulocytes under alhydrogel perturbation was negatively associated with DCM, suggesting that reducing abnormal erythropoiesis may help lower the risk of DCM.

Dysregulation of the immune system and the inflammatory response are intrinsic factors in the pathogenesis of DCM.^[40]^ Previous studies have indicated significant infiltration of CD4+ and CD8+ T cells, as well as γδ T cells, in the myocardial tissue of patients with DCM. An increase in the expression of perforin and other cytolytic markers was also observed, suggesting cell-mediated cytotoxic activity within the cardiac tissue.^[41]^ NK cells play a crucial role in preventing acute viral pathogens associated with myocarditis. The reduced activity of NK cells in patients with dilated cardiomyopathy indicates a close relationship between NK cell suppression and the pathophysiology of chronic myocarditis, which may be related to the occurrence of mild dilated cardiomyopathy.^[42]^ Kanda et al^[43]^ investigated the differences in NK cells in the myocardium and circulating blood between patients with DCM and 12 normal control subjects and suggested that the dysfunction of NK cell subsets in DCM patients may be related to its pathogenesis. Similarly, Zhang et al^[44]^ reported that activated NK cells and eosinophils present in the myocardial tissue of DCM patients are central immune cells. Jiao et al^[45]^ analyzed CD24hiCD27+ B cells in the peripheral blood of 35 DCM patients and 44 healthy controls via flow cytometry and reported that the CD24hiCD27+ B cells in DCM patients presented lower IL-10 expression and a diminished capacity to suppress TNF-α production in CD4+ CD25− T cells and maintain Treg differentiation. In our study, 31 immune phenotypes were found to be causally linked to DCM, with both risk-enhancing and risk-protective effects. Notably, increased levels of side scatter area on HLA DR+ NK cells, CD3 on CD39+ CD4+ cells, and side scatter area on CD8bright cells were associated with a heightened risk of DCM. Conversely, this study revealed protective effects from other immune phenotypes, including CD25 on IgD+ CD38bright B cells, CD45 on T cells, TD DN (CD4− CD8−) %DN, and forward scatter area on CD4+ T cells. These findings suggest that NK cells and specific T cell subpopulations may contribute to the inflammatory processes that underlie the development of DCM, but certain immune cell functions may counteract the pathological mechanisms leading to DCM, possibly through the modulation of immune responses and prevention of excessive inflammation.

Moreover, the reticulocyte perturbation response induced by the alhydrogel exerted a negative mediating effect on DCM via CD127 on CD8br cells. Recent studies have shown that myocardial fibrosis is closely associated with immune infiltration, revealing substantial infiltration of T cells, with nearly 50% of DCM patients exhibiting cardiac T cell infiltration.^[46,47]^ However, compared with that in healthy controls, the percentage of Treg cells in DCM patients is markedly lower, and increasing the number or enhancing the function of Treg cells may represent a promising therapeutic strategy for DCM.^[48]^ The precise mechanism by which red blood cells mediate their effects through Treg cells requires further investigation.

HCM is caused by dominant mutations in 11 or more genes encoding the thick and thin filament proteins of the sarcomere or adjacent Z-disc; however, the currently identified pathogenic mutations do not fully explain the morphological features, primarily due to the heterogeneity between genetics and phenotypes.^[49]^ Immune cells play roles in heart development, homeostasis maintenance, and cardiac aging.^[50]^ Studies have shown a significant enrichment of neutrophils and naive and memory B cells in HCM tissue, while the enrichment of macrophages is lower than that in normal tissue.^[51]^ Another study revealed a significant increase in macrophages, NK cells, and monocytes in HCM tissue, whereas CD4 memory resting T cells infiltration was decreased compared with that in healthy controls.^[52]^ Our findings indicate that monocytes and platelets are risk factors for HCM and that they exert positive mediating effects through various TBNK cell subsets. In the early stages of myocardial injury, an inflammatory environment, driven by various inflammatory factors, recruits a large number of M1 macrophages from the circulatory pool. These macrophages produce potent cytokines, including but not limited to IL-1β, IL-6, and TNF-α, inducing effects such as apoptosis, cell lysis, and proteolysis.^[53,54]^ Subsequently, monocytes are recruited, differentiate into macrophages, and polarize into the M2 phenotype, playing a crucial protective and reparative role, including promoting the formation of new blood vessels.^[53,55,56]^ The timing and proportion of these 2 distinct macrophage phenotypes are critical, with the eventual predominance of M2 macrophages sufficient to stimulate cardiac repair and improve post-injury function.^[53,57,58]^ Interestingly, conclusions regarding the association between monocytes and HCM vary across studies, and the underlying reasons for this inconsistency warrant further investigation.

In patients with HCM, the levels of thrombin, platelets, and inflammatory markers are significantly elevated, and the increase in inflammatory markers may be a consequence of coagulation activation in these individuals.^[59]^ Additionally, increased platelet size, reduced phosphorus concentration, and enhanced cation permeability have been observed in HCM patients, suggesting potential abnormalities in membrane integrity and energy metabolism.^[60]^ Bioinformatic analyses have revealed that the infiltration of macrophages, monocytes, dendritic cells, Th1 cells, Treg cells, and plasma cells is markedly reduced in HCM patients, whereas the infiltration of CD8+ T cells, basophils, fibroblasts, and platelets is significantly increased.^[61]^ Platelets play a critical role in cardiac inflammation and immune responses by interacting with various leukocytes to induce systemic inflammatory reactions.^[62,63]^ Upon activation, platelets express specific adhesion molecules, including P-selectin, cluster of differentiation 40L, glycoprotein IIb, and tumor necrosis factor superfamily member 14, and secrete large amounts of cytokines and chemokines.^[63,64]^ Notably, P-selectin binds to P-selectin glycoprotein ligand-1 expressed on the surface of monocytes, thereby mediating monocyte recruitment to inflammatory sites and promoting the release of inflammatory mediators, which in turn further stimulates platelet activation and factor secretion.^[62]^ This positive feedback loop amplifies the inflammatory response and may contribute to myocardial fibrosis. Consistently, our findings indicate that platelets promote the development of HCM through leukocyte-mediated mechanisms. Currently, there are limited studies investigating the effects of isobutyric acid and isobutyrates on the circulatory system. Existing research has shown that, in patients with atherosclerosis, sodium isobutyrate treatment significantly reduces the platelet adhesion index.^[65]^ It is anticipated that further research on isobutyric acid and isobutyrates will help refine and strengthen the conclusions of this study.

However, this study has several limitations. The use of GWAS data, predominantly from European populations, limits the generalizability of the findings to other ethnic groups. Additionally, the study employed a relaxed threshold for selecting instrumental variables, which may increase the risk of false-positive findings. Although sensitivity analyses, including MR-Egger regression and MR-PRESSO, were conducted to assess pleiotropy and heterogeneity, the potential for bias cannot be entirely excluded. Furthermore, although these mediating effects are statistically detectable, their magnitude is limited, and their biological significance remains to be fully elucidated.

## 5. Conclusion

This study, grounded in the principles of Mendelian randomization, explored the potential causal relationships among blood cells, immune cells, and 2 distinct types of cardiomyopathy. The findings identified several potential risk factors associated with HCM/DCM and examined the mediating role of immune cells in the causal pathway between blood cells and cardiomyopathy. These results may offer new insights into how genetic exposures influence the development of cardiomyopathy.

## Acknowledgments

We would like to thank all the GWAS participants and researchers for generously sharing their data.

## Author contributions

**Conceptualization:** Kang Xu, Lanxin Ma, Ying Xu.

**Data curation:** Hongfei Du, Huacui Huang.

**Formal analysis:** Kang Xu, Lanxin Ma, Hongfei Du.

**Investigation:** Huacui Huang.

**Methodology:** Ying Xu.

**Project administration:** Ying Xu.

**Supervision:** Ying Xu.

**Writing – original draft:** Kang Xu, Lanxin Ma.

**Writing – review & editing:** Kang Xu, Lanxin Ma, Ying Xu.

## Supplementary Material




