# Supplementary material for: Causal relationships between blood cell perturbation responses, immune cell phenotypes, and cardiomyopathy: A two-sample Mendelian randomization and mediation analysis
Source: Medicine (Baltimore). 2026 Apr 17;105(16):e48404. doi: 10.1097/MD.0000000000048404 (PMC13095301; doi:10.1097/MD.0000000000048404)
Supplement: Supplementary file 2 [file medi-105-e48404-s002.pdf]

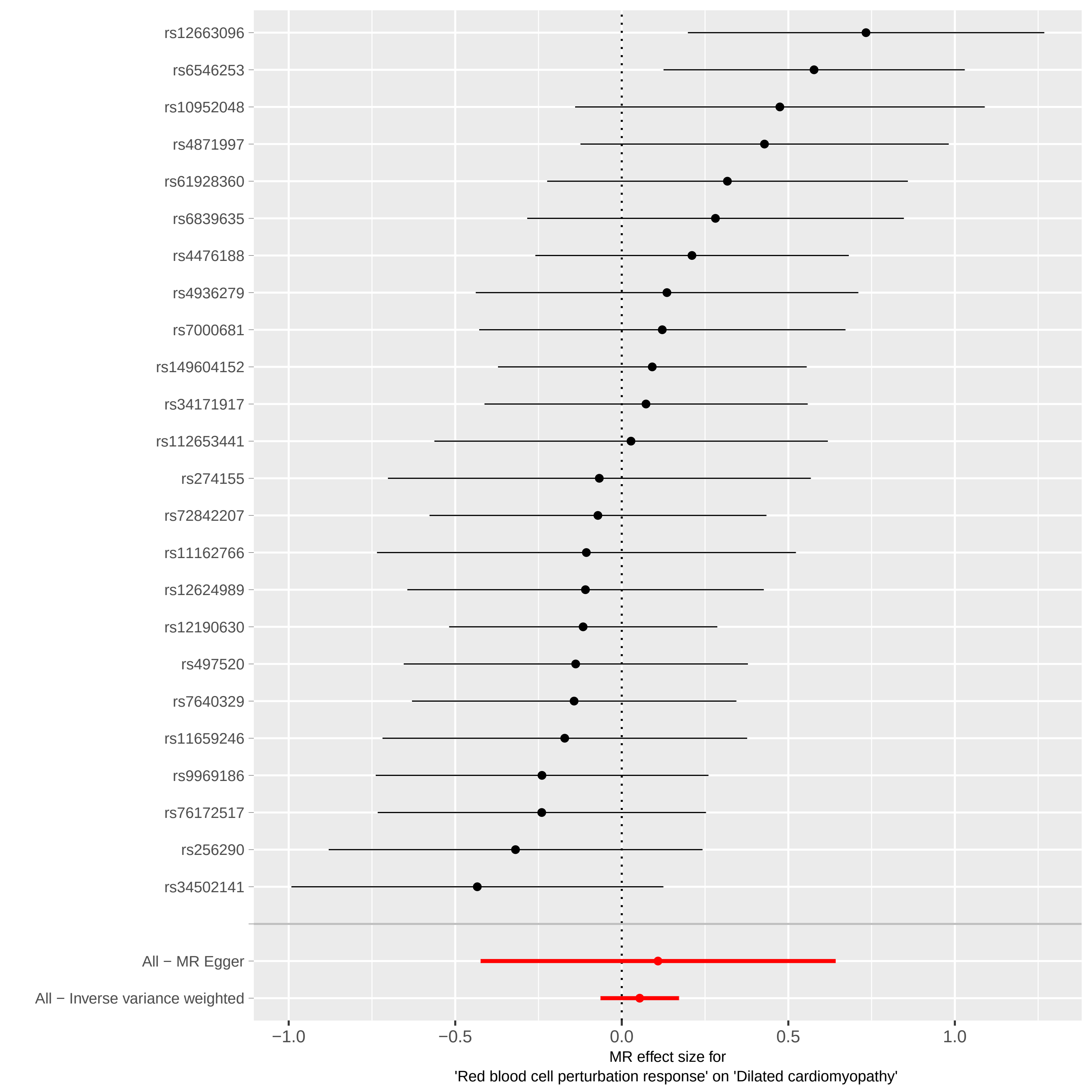
rs12663096
rs6546253
rs10952048
rs4871997
rs61928360
rs6839635
rs4476188
rs4936279
rs7000681
rs149604152
rs34171917
rs112653441
rs274155
rs72842207
rs11162766
rs12624989
rs12190630
rs497520
rs7640329
rs11659246
rs9969186
rs76172517
rs256290
rs34502141
All − MR Egger
All − Inverse variance weighted
−1.0
−0.5
0.0
0.5
1.0
MR effect size for
'Red blood cell perturbation response' on 'Dilated cardiomyopathy'

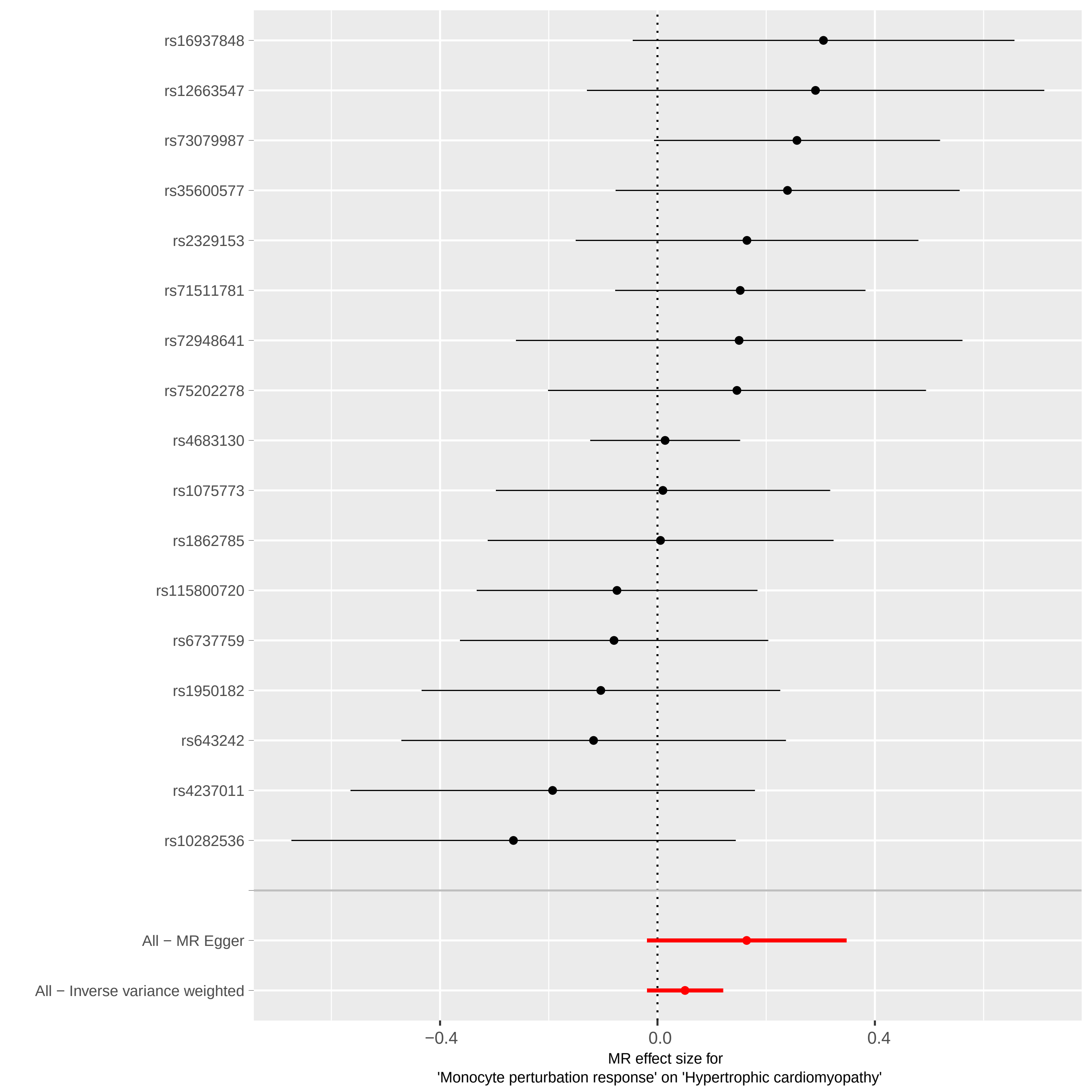
rs16937848
rs12663547
rs73079987
rs35600577
rs2329153
rs71511781
rs72948641
rs75202278
rs4683130
rs1075773
rs1862785
rs115800720
rs6737759
rs1950182
rs643242
rs4237011
rs10282536
All − MR Egger
All − Inverse variance weighted
−0.4
0.0
0.4
MR effect size for
'Monocyte perturbation response' on 'Hypertrophic cardiomyopathy'

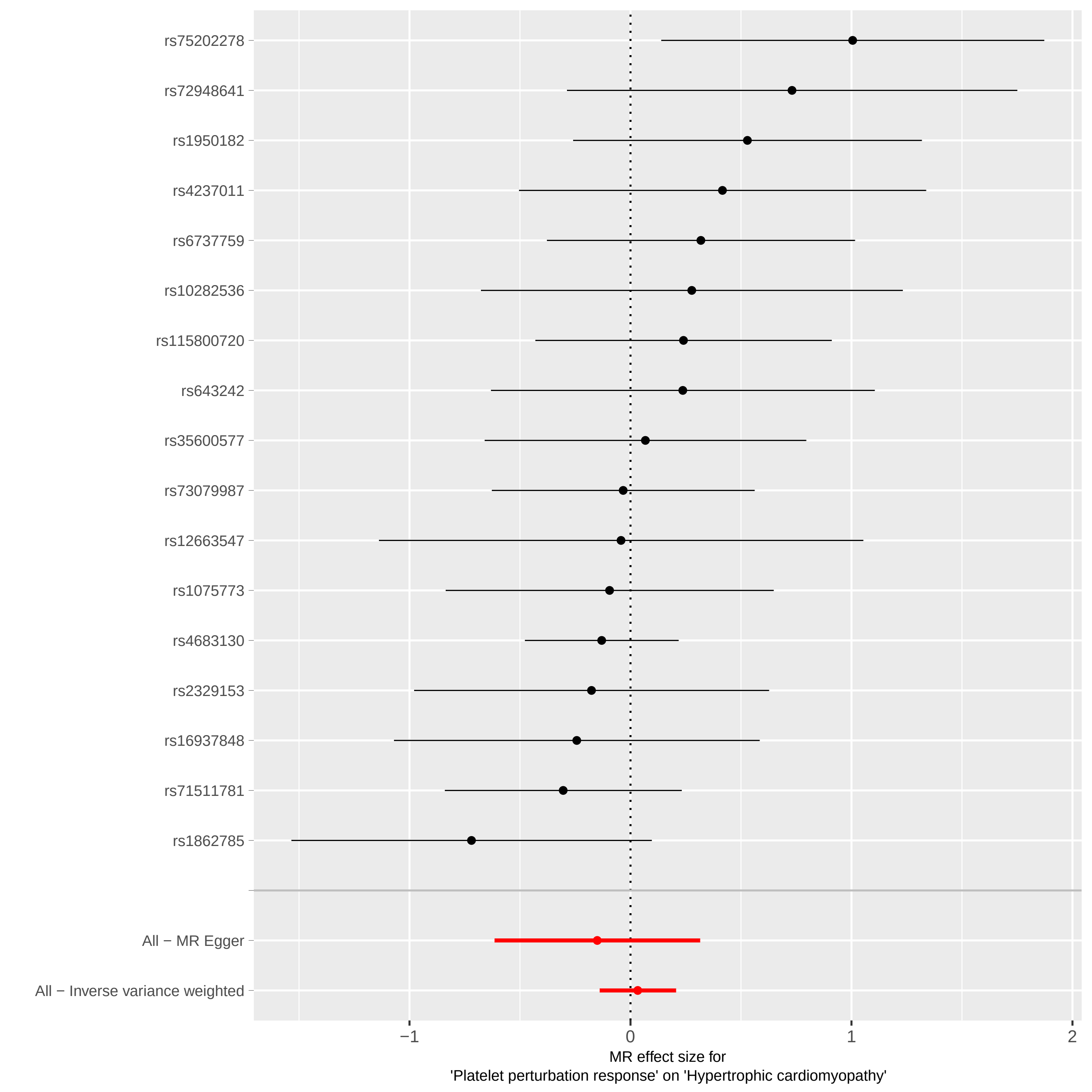
rs75202278
rs72948641
rs1950182
rs4237011
rs6737759
rs10282536
rs115800720
rs643242
rs35600577
rs73079987
rs12663547
rs1075773
rs4683130
rs2329153
rs16937848
rs71511781
rs1862785
All − MR Egger
All − Inverse variance weighted
−1
0
1
2
MR effect size for
'Platelet perturbation response' on 'Hypertrophic cardiomyopathy'

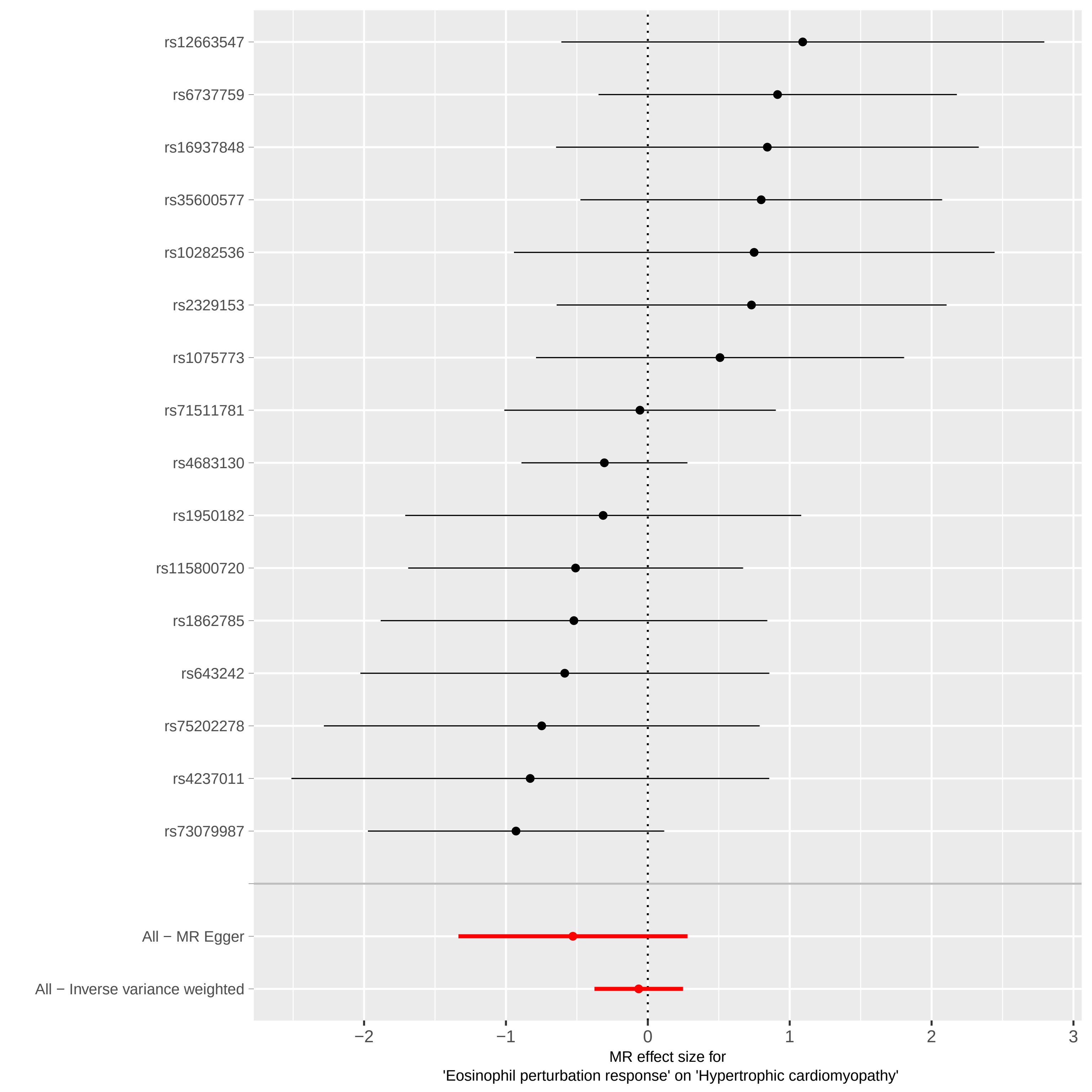
rs12663547
rs6737759
rs16937848
rs35600577
rs10282536
rs2329153
rs1075773
rs71511781
rs4683130
rs1950182
rs115800720
rs1862785
rs643242
rs75202278
rs4237011
rs73079987
All − MR Egger
All − Inverse variance weighted
−2
−1
0
1
2
3
MR effect size for
'Eosinophil perturbation response' on 'Hypertrophic cardiomyopathy'

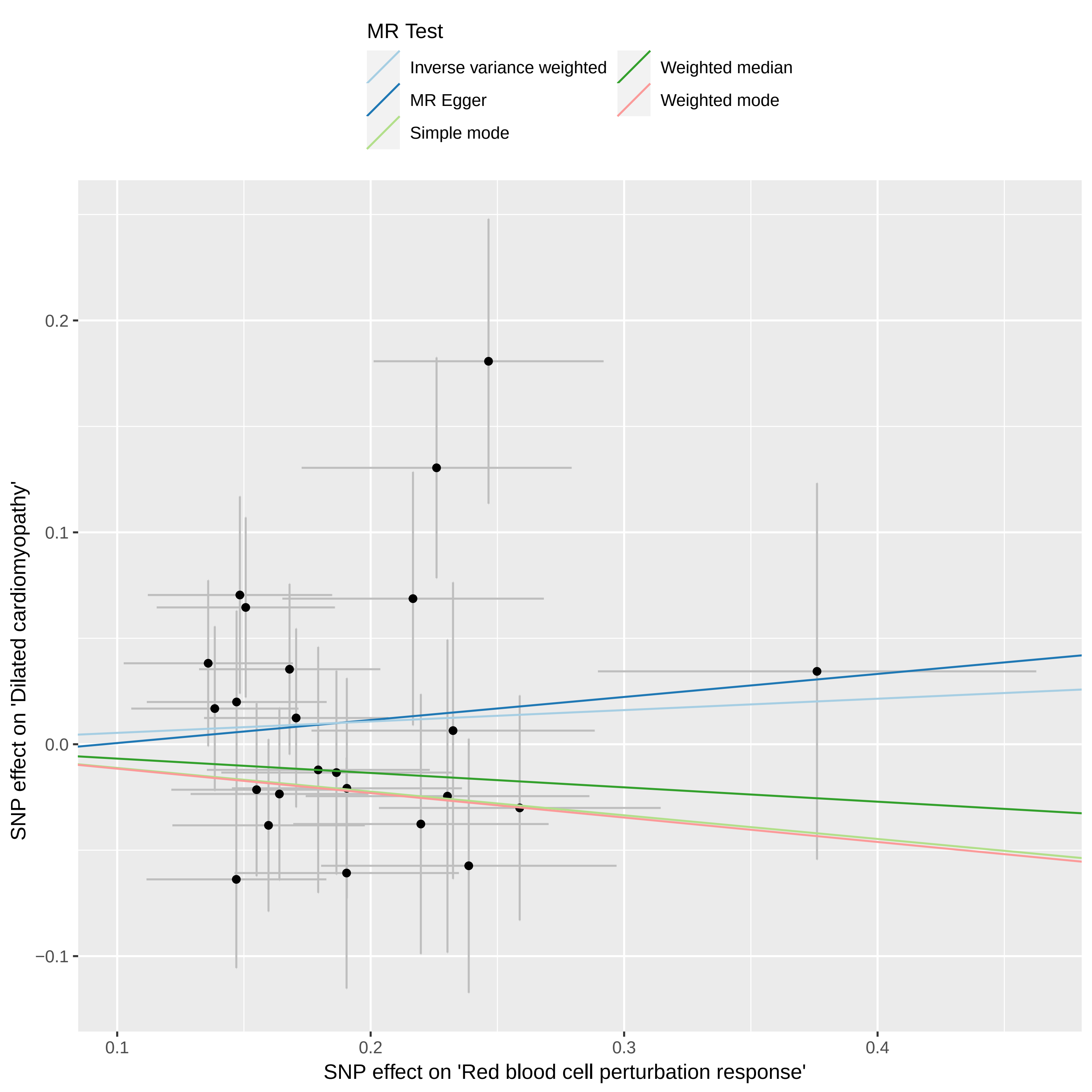
MR Test
Inverse variance weighted
MR Egger
Simple mode
Weighted median
Weighted mode
SNP effect on 'Dilated cardiomyopathy'
0.2
0.1
0.0
−0.1
0.1
0.2
0.3
0.4
SNP effect on 'Red blood cell perturbation response'

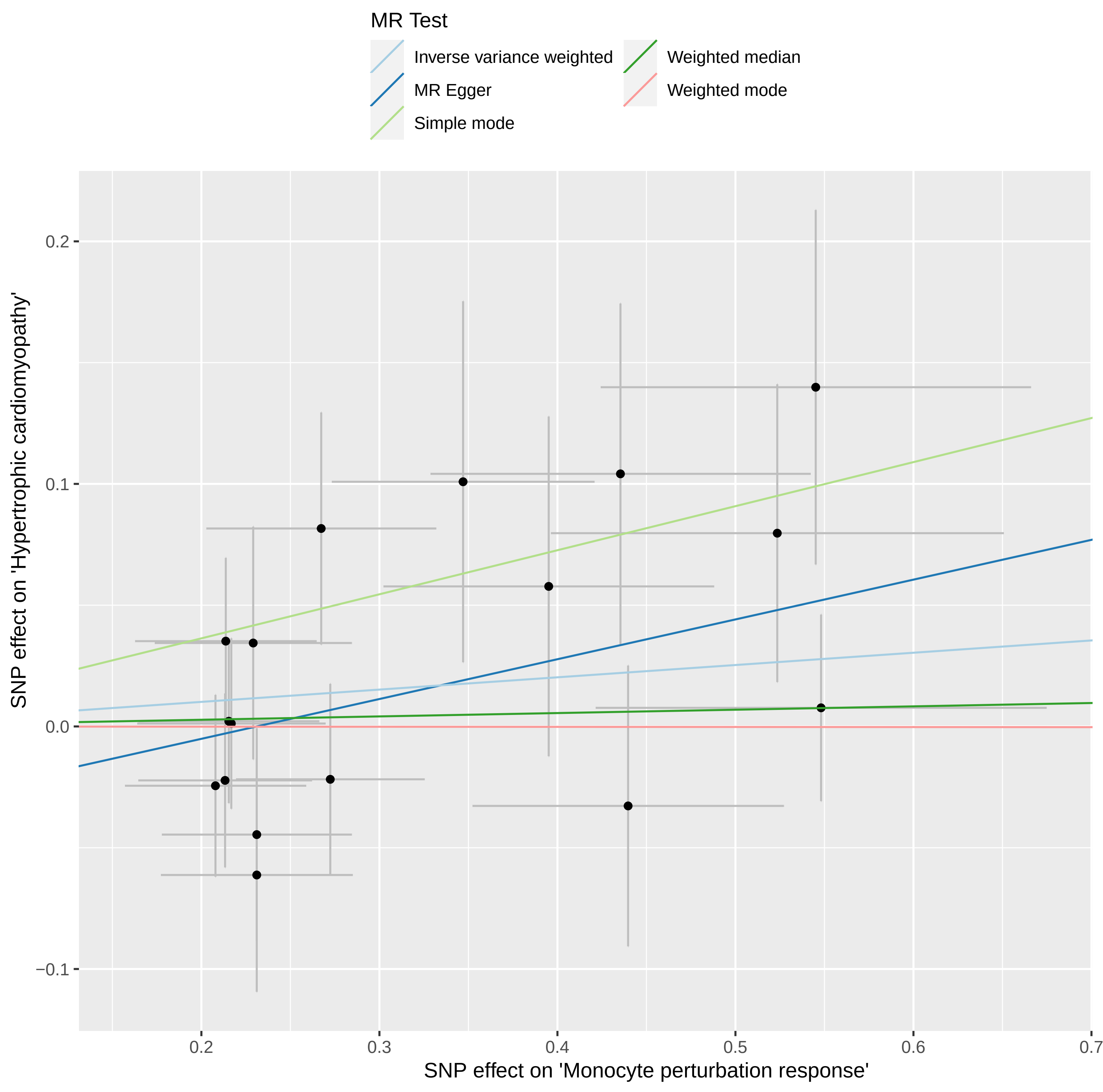
MR Test
Inverse variance weighted
MR Egger
Simple mode
Weighted median
Weighted mode
0.2
0.1
0.0
−0.1
0.2
0.3
0.4
0.5
0.6
0.7
SNP effect on 'Hypertrophic cardiomyopathy'
SNP effect on 'Monocyte perturbation response'

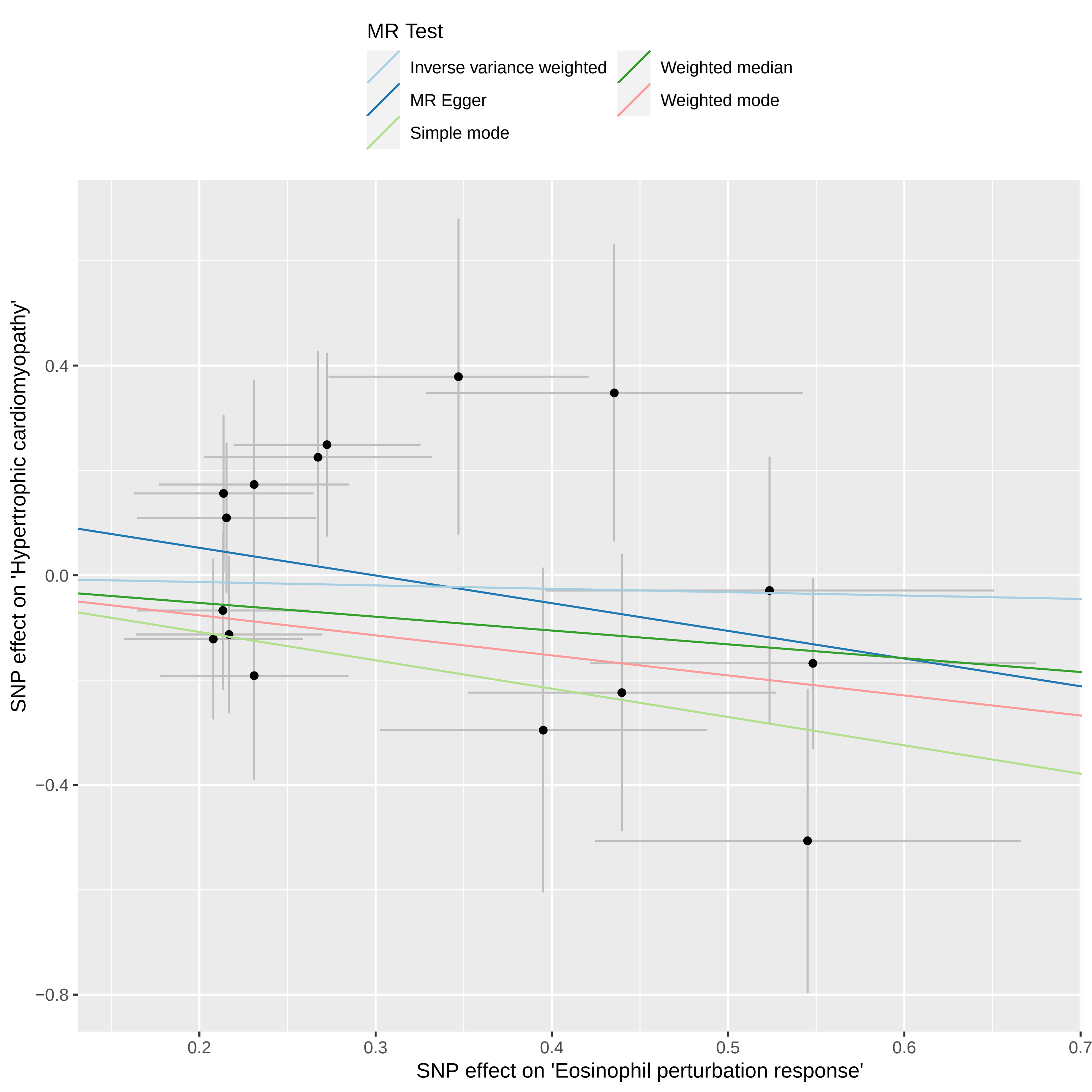

MR Test
Inverse variance weighted
MR Egger
Simple mode
Weighted median
Weighted mode
0.4
0.0
−0.4
−0.8
0.2
0.3
0.4
0.5
0.6
0.7
SNP effect on 'Eosinophil perturbation response'
SNP effect on 'Hypertrophic cardiomyopathy'

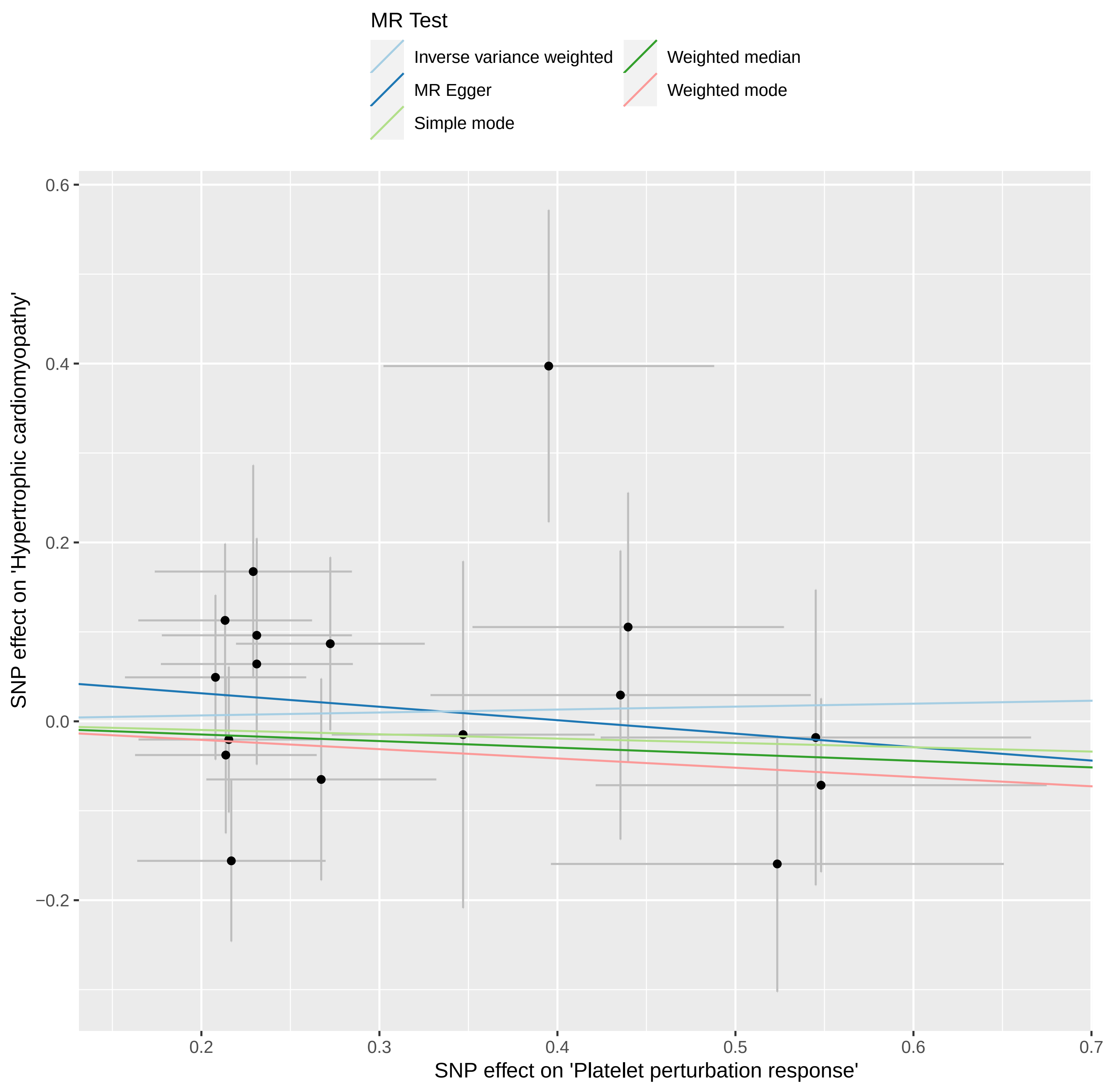
MR Test
Inverse variance weighted
MR Egger
Simple mode
Weighted median
Weighted mode
SNP effect on 'Hypertrophic cardiomyopathy'
SNP effect on 'Platelet perturbation response'
0.6
0.4
0.2
0.0
−0.2
0.2
0.3
0.4
0.5
0.6
0.7

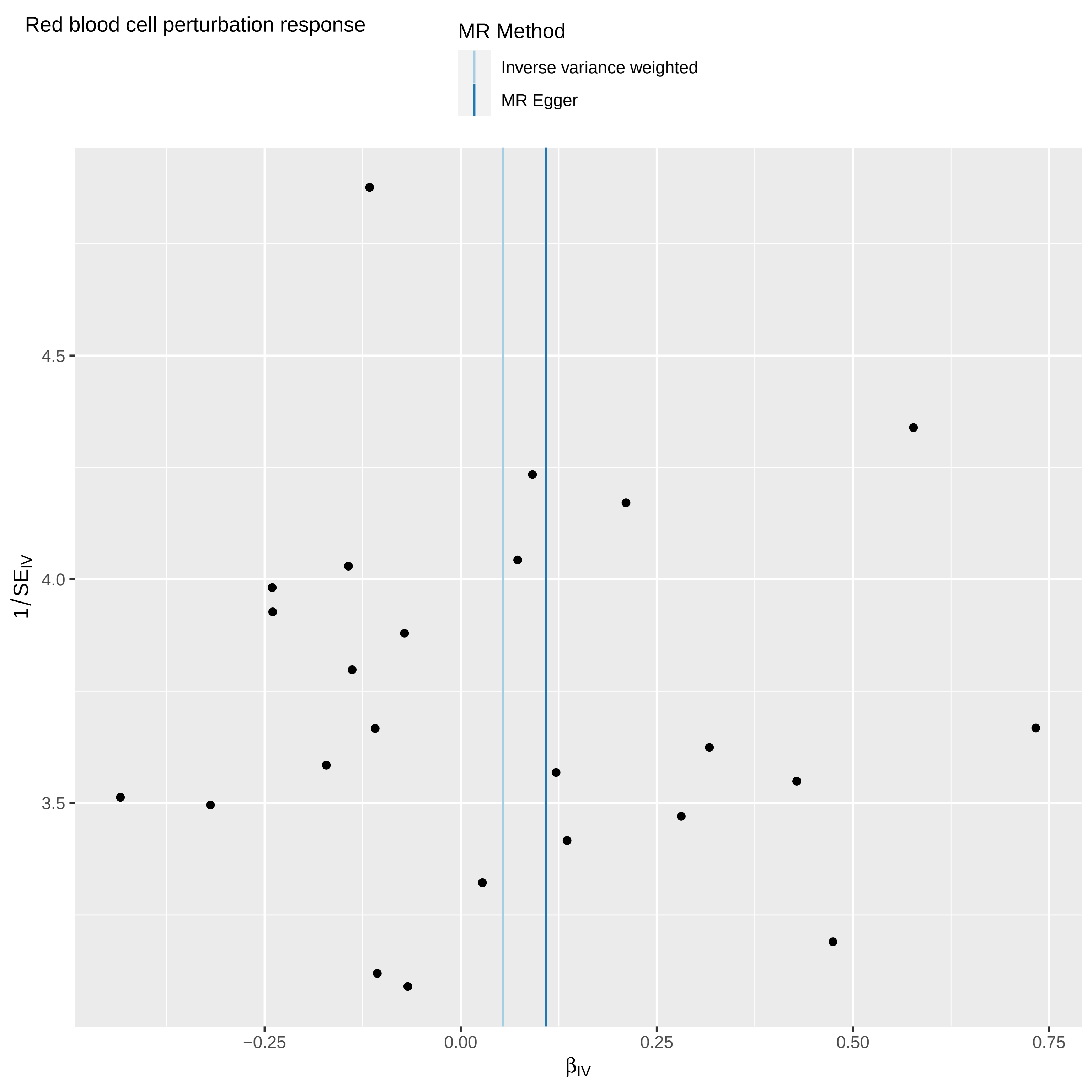
Red blood cell perturbation response
MR Method
Inverse variance weighted
MR Egger
1/SE_IV
4.5
4.0
3.5
β_IV
−0.25
0.00
0.25
0.50
0.75

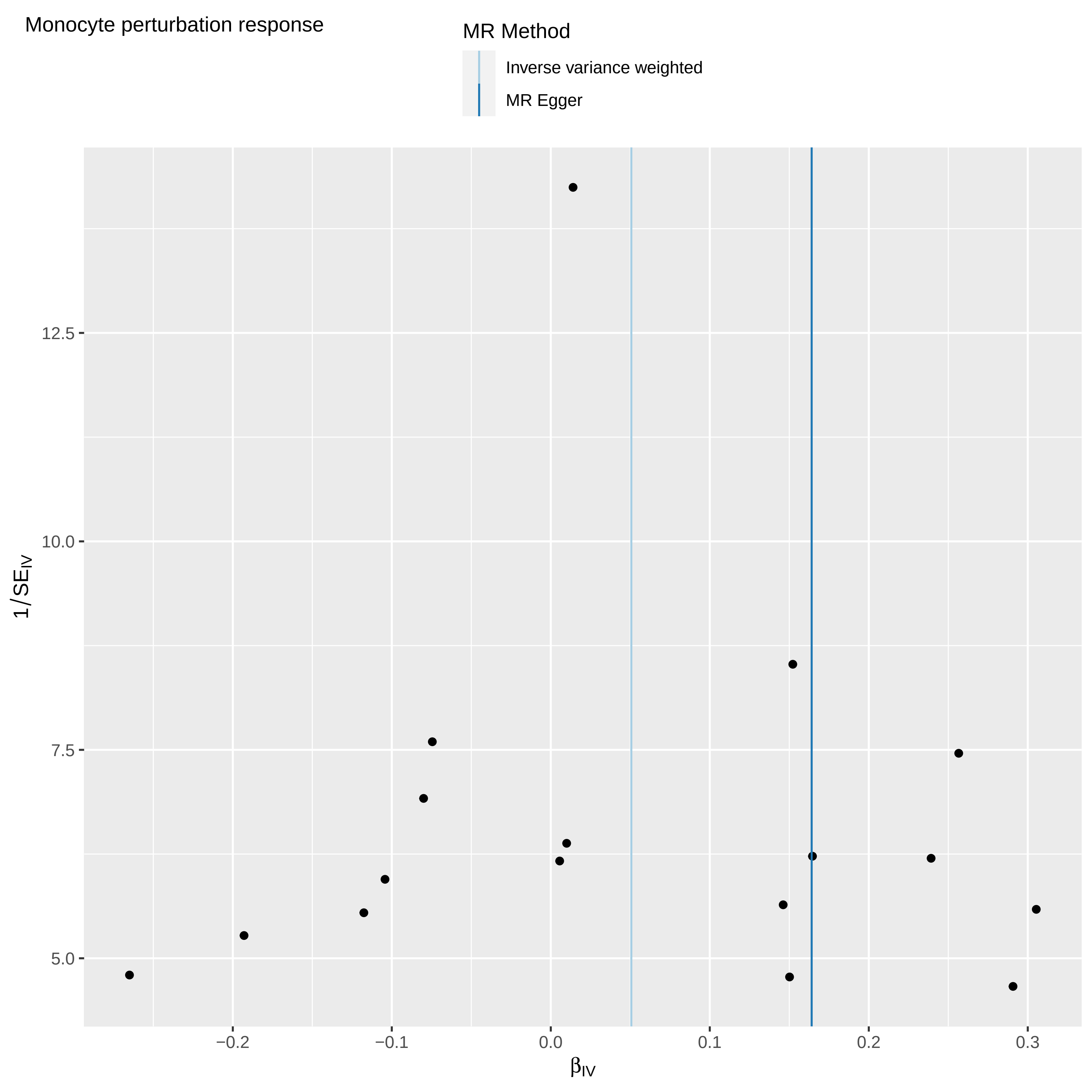
Monocyte perturbation response
MR Method
Inverse variance weighted
MR Egger
12.5
10.0
7.5
5.0
1/SE_IV
−0.2
−0.1
0.0
0.1
0.2
0.3
β_IV

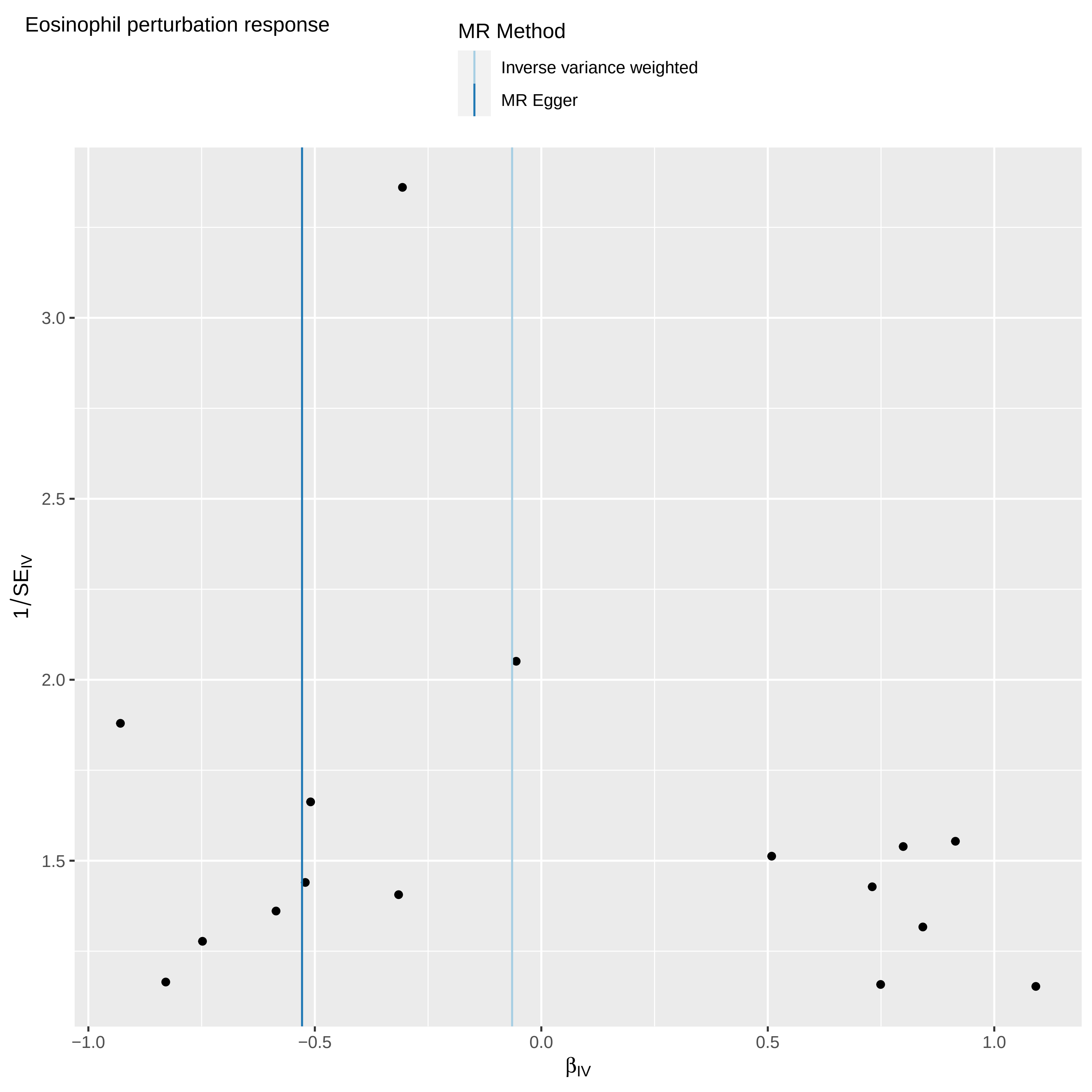

Eosinophil perturbation response
MR Method
Inverse variance weighted
MR Egger
3.0
2.5
2.0
1.5
$1/SE_{IV}$
−1.0
−0.5
0.0
0.5
1.0
β_{IV}

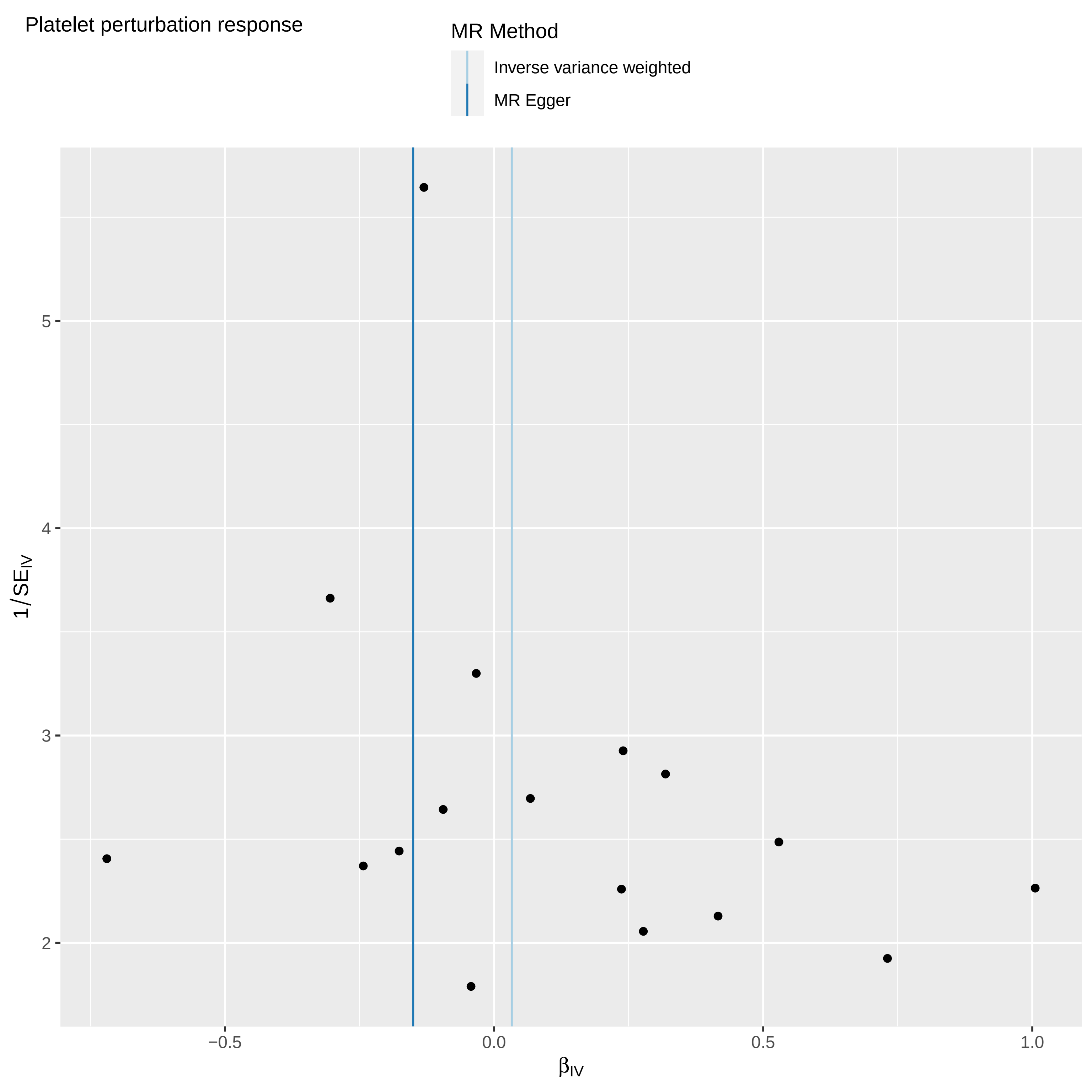

Platelet perturbation response
MR Method
Inverse variance weighted
MR Egger
1/SE_IV
β_IV
5
4
3
2
−0.5
0.0
0.5
1.0

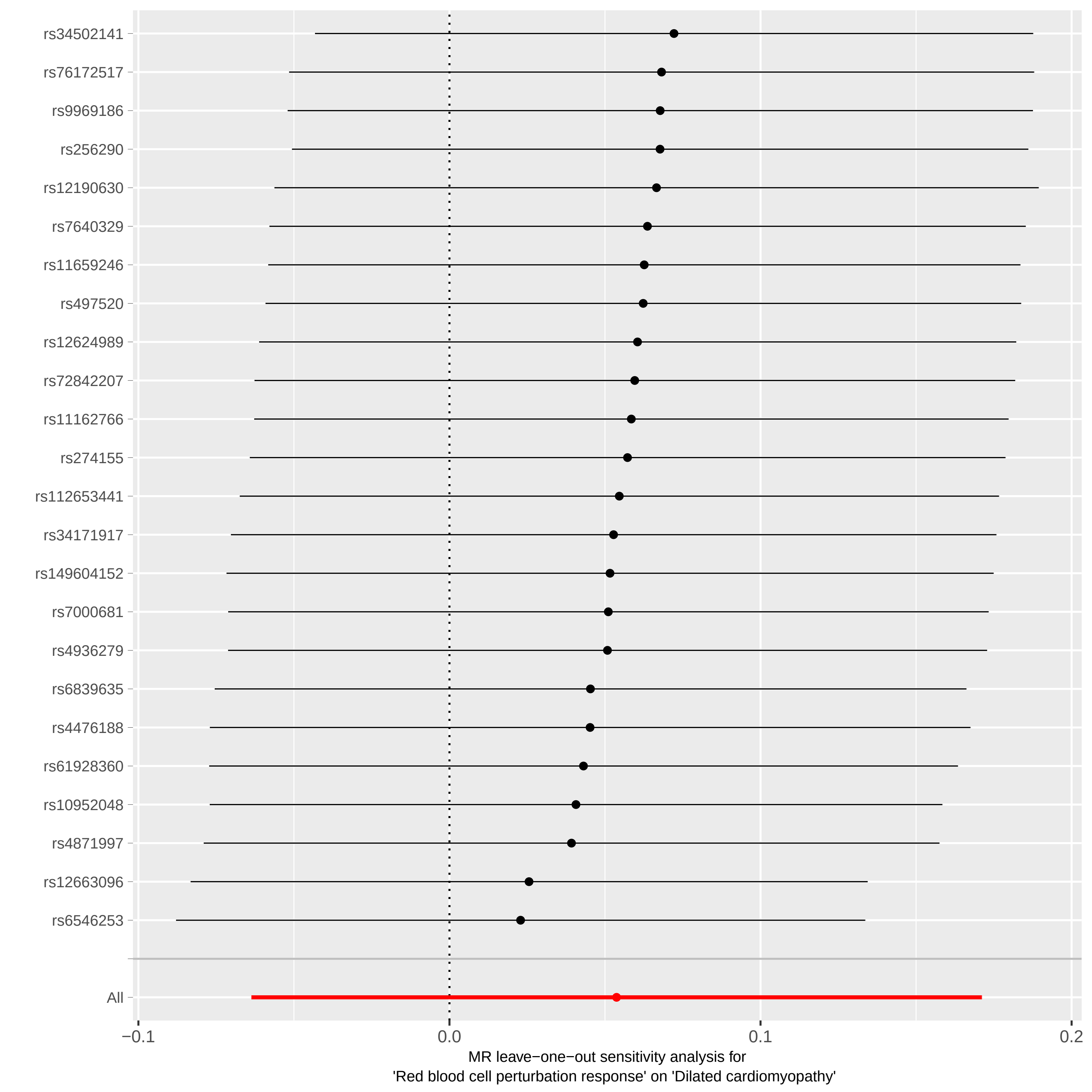
rs34502141
rs76172517
rs9969186
rs256290
rs12190630
rs7640329
rs11659246
rs497520
rs12624989
rs72842207
rs11162766
rs274155
rs112653441
rs34171917
rs149604152
rs7000681
rs4936279
rs6839635
rs4476188
rs61928360
rs10952048
rs4871997
rs12663096
rs6546253
All
−0.1
0.0
0.1
0.2
MR leave−one−out sensitivity analysis for
'Red blood cell perturbation response' on 'Dilated cardiomyopathy'

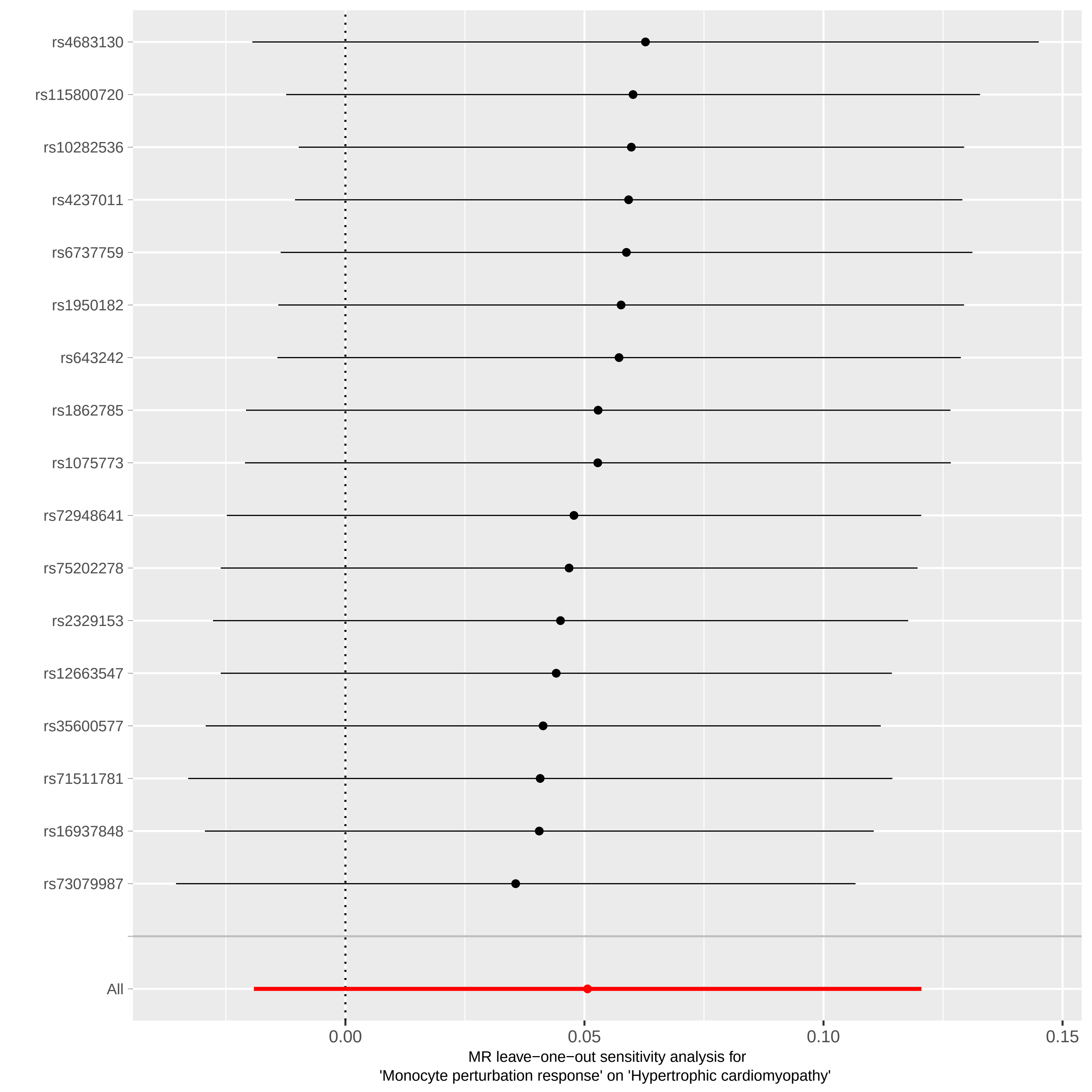
rs4683130
rs115800720
rs10282536
rs4237011
rs6737759
rs1950182
rs643242
rs1862785
rs1075773
rs72948641
rs75202278
rs2329153
rs12663547
rs35600577
rs71511781
rs16937848
rs73079987
All
0.00
0.05
0.10
0.15
MR leave−one−out sensitivity analysis for
'Monocyte perturbation response' on 'Hypertrophic cardiomyopathy'

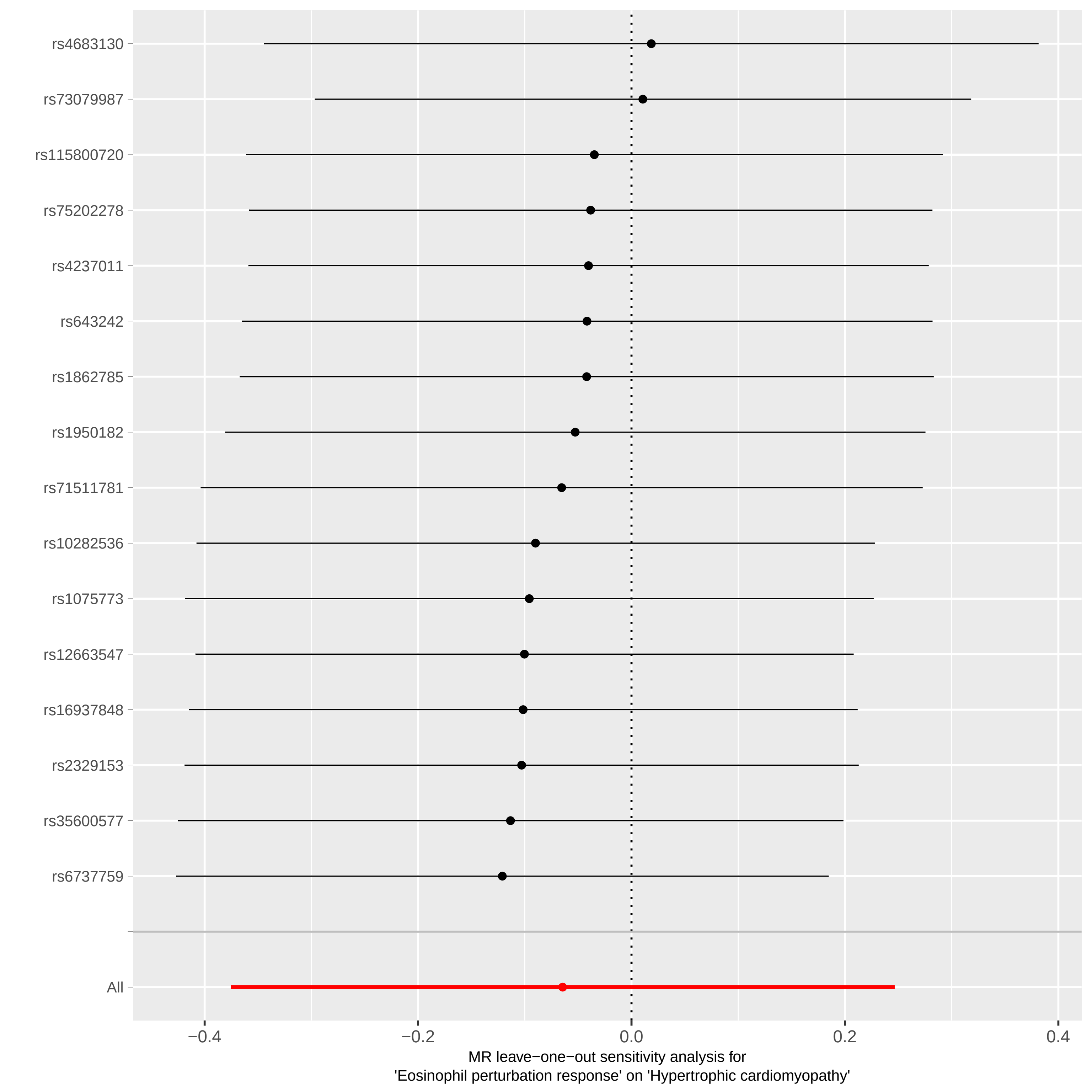
rs4683130
rs73079987
rs115800720
rs75202278
rs4237011
rs643242
rs1862785
rs1950182
rs71511781
rs10282536
rs1075773
rs12663547
rs16937848
rs2329153
rs35600577
rs6737759
All
−0.4
−0.2
0.0
0.2
0.4
MR leave−one−out sensitivity analysis for
'Eosinophil perturbation response' on 'Hypertrophic cardiomyopathy'

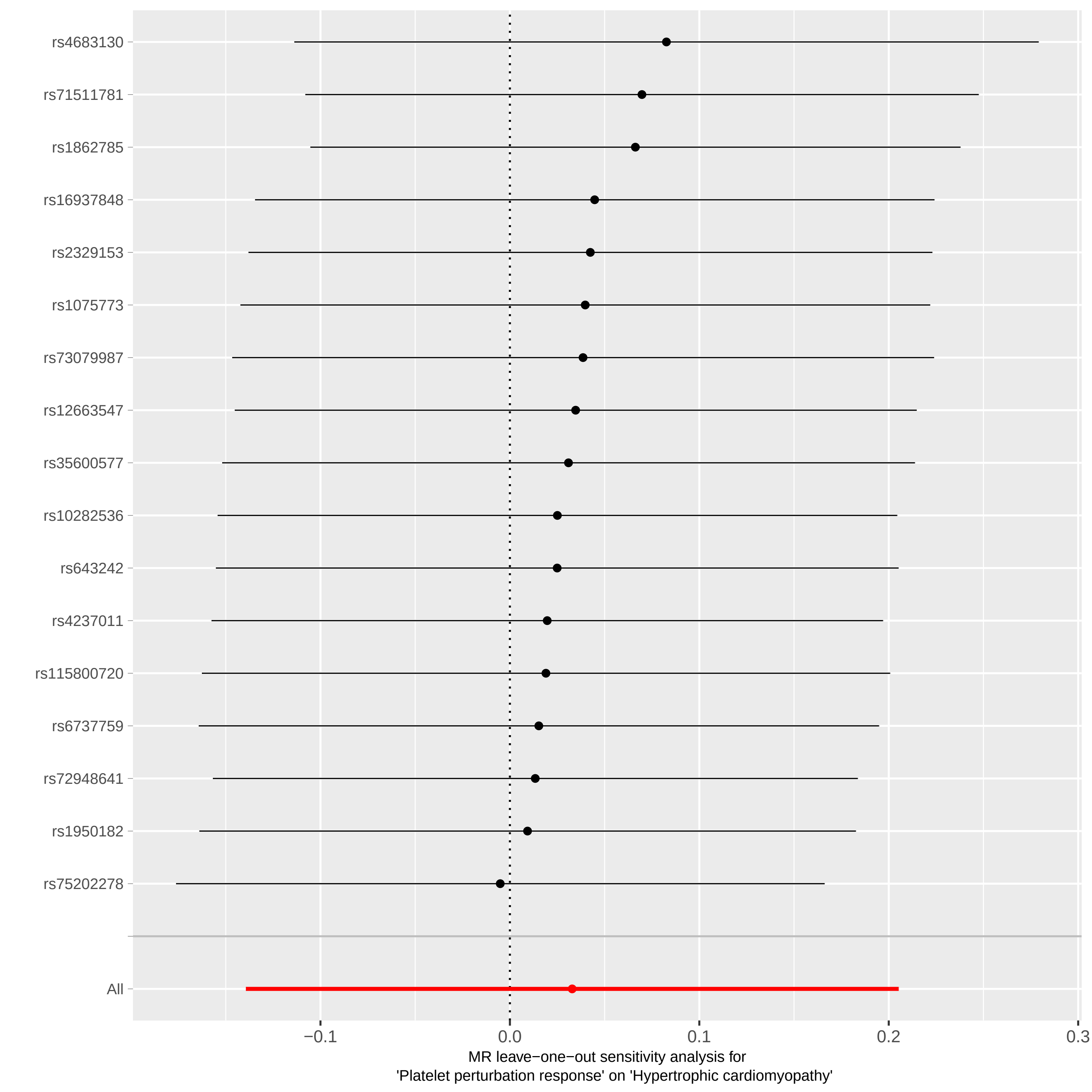
rs4683130
rs71511781
rs1862785
rs16937848
rs2329153
rs1075773
rs73079987
rs12663547
rs35600577
rs10282536
rs643242
rs4237011
rs115800720
rs6737759
rs72948641
rs1950182
rs75202278
All
−0.1
0.0
0.1
0.2
0.3
MR leave−one−out sensitivity analysis for
'Platelet perturbation response' on 'Hypertrophic cardiomyopathy'

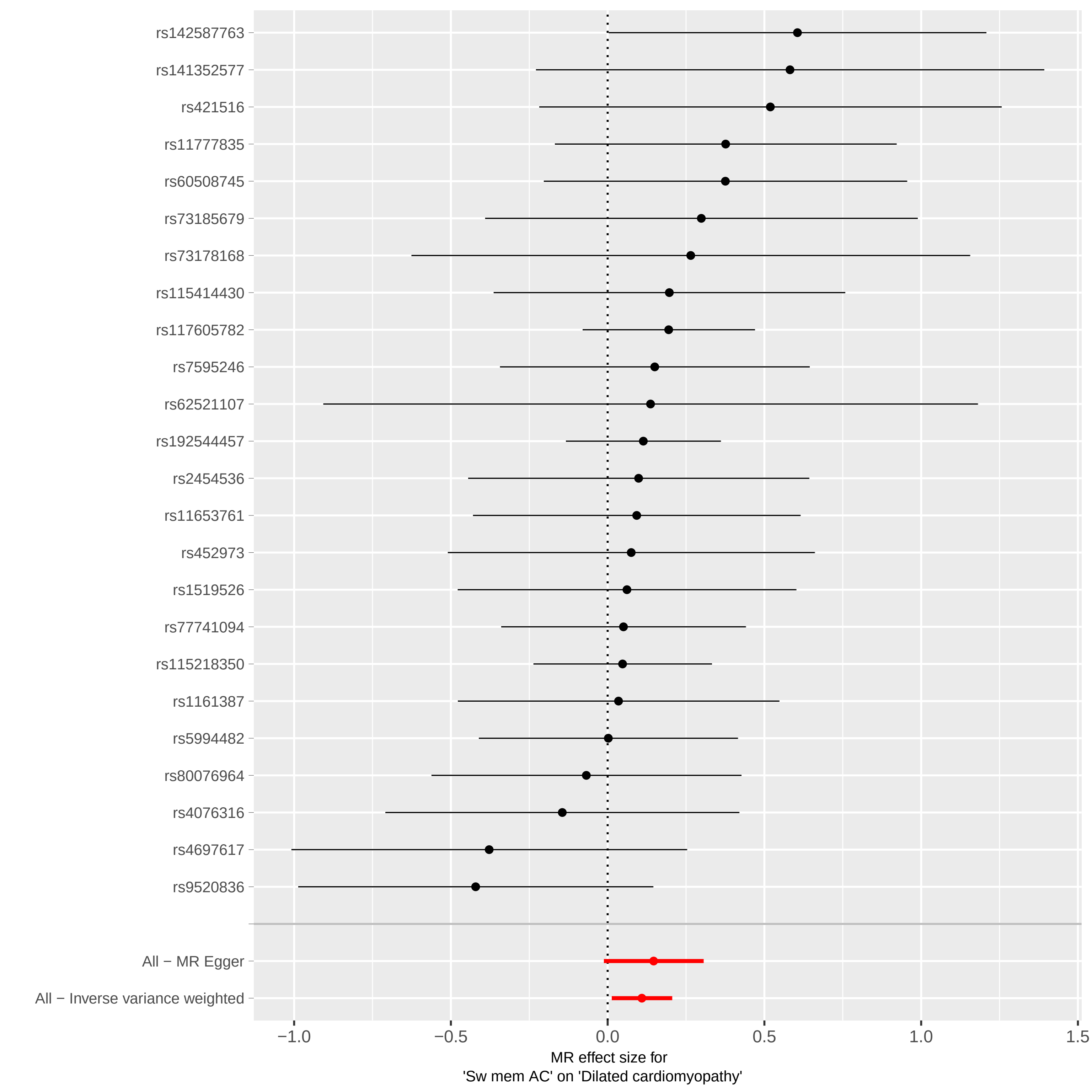
rs142587763
rs141352577
rs421516
rs11777835
rs60508745
rs73185679
rs73178168
rs115414430
rs117605782
rs7595246
rs62521107
rs192544457
rs2454536
rs11653761
rs452973
rs1519526
rs77741094
rs115218350
rs1161387
rs5994482
rs80076964
rs4076316
rs4697617
rs9520836
All − MR Egger
All − Inverse variance weighted
−1.0
−0.5
0.0
0.5
1.0
1.5
MR effect size for
'Sw mem AC' on 'Dilated cardiomyopathy'

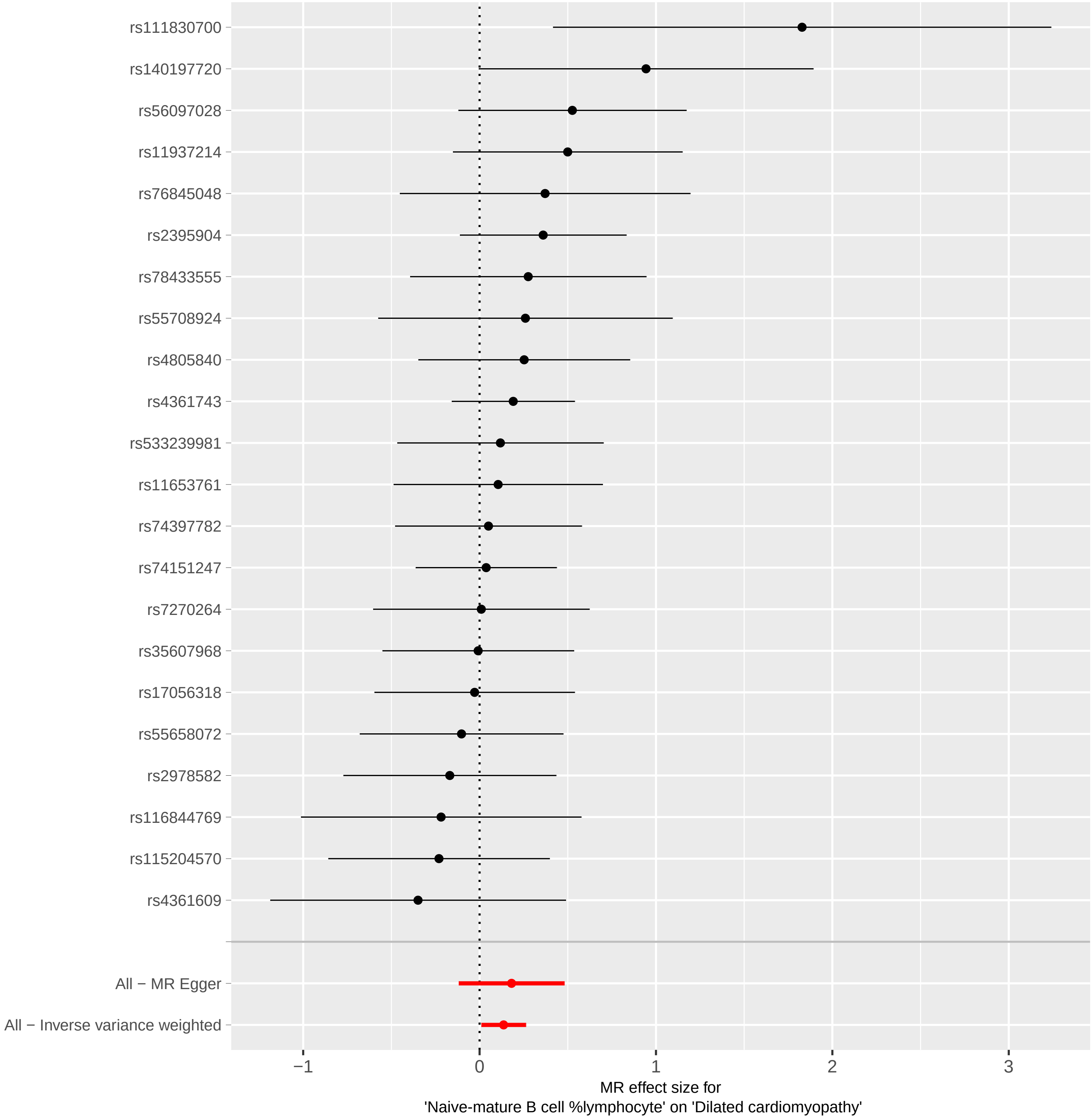
rs111830700
rs140197720
rs56097028
rs11937214
rs76845048
rs2395904
rs78433555
rs55708924
rs4805840
rs4361743
rs533239981
rs11653761
rs74397782
rs74151247
rs7270264
rs35607968
rs17056318
rs55658072
rs2978582
rs116844769
rs115204570
rs4361609
All − MR Egger
All − Inverse variance weighted
−1
0
1
2
3
MR effect size for
'Naive-mature B cell %lymphocyte' on 'Dilated cardiomyopathy'

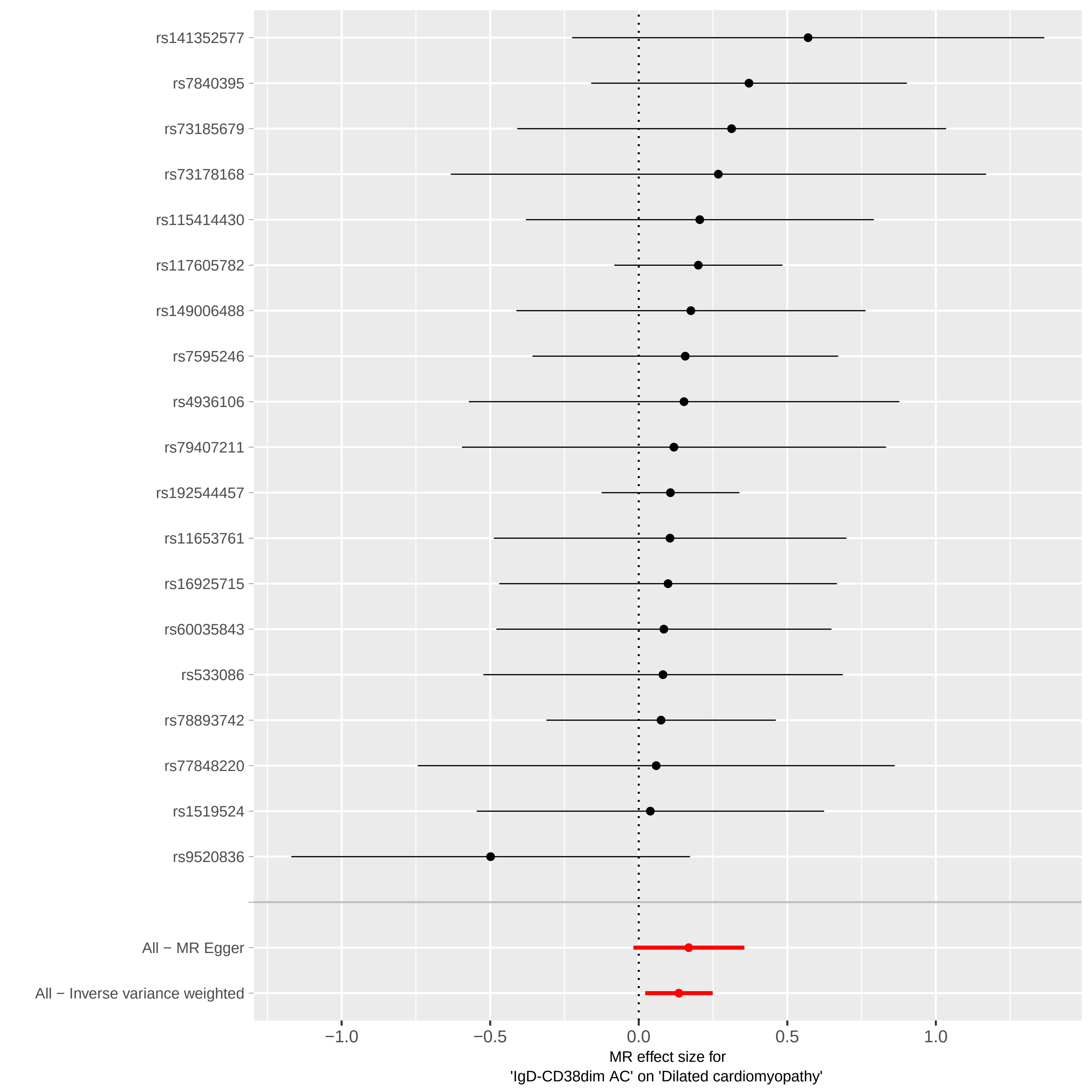

rs141352577
rs7840395
rs73185679
rs73178168
rs115414430
rs117605782
rs149006488
rs7595246
rs4936106
rs79407211
rs192544457
rs11653761
rs16925715
rs60035843
rs533086
rs78893742
rs77848220
rs1519524
rs9520836
All − MR Egger
All − Inverse variance weighted
−1.0
−0.5
0.0
0.5
1.0
MR effect size for
'IgD-CD38dim AC' on 'Dilated cardiomyopathy'

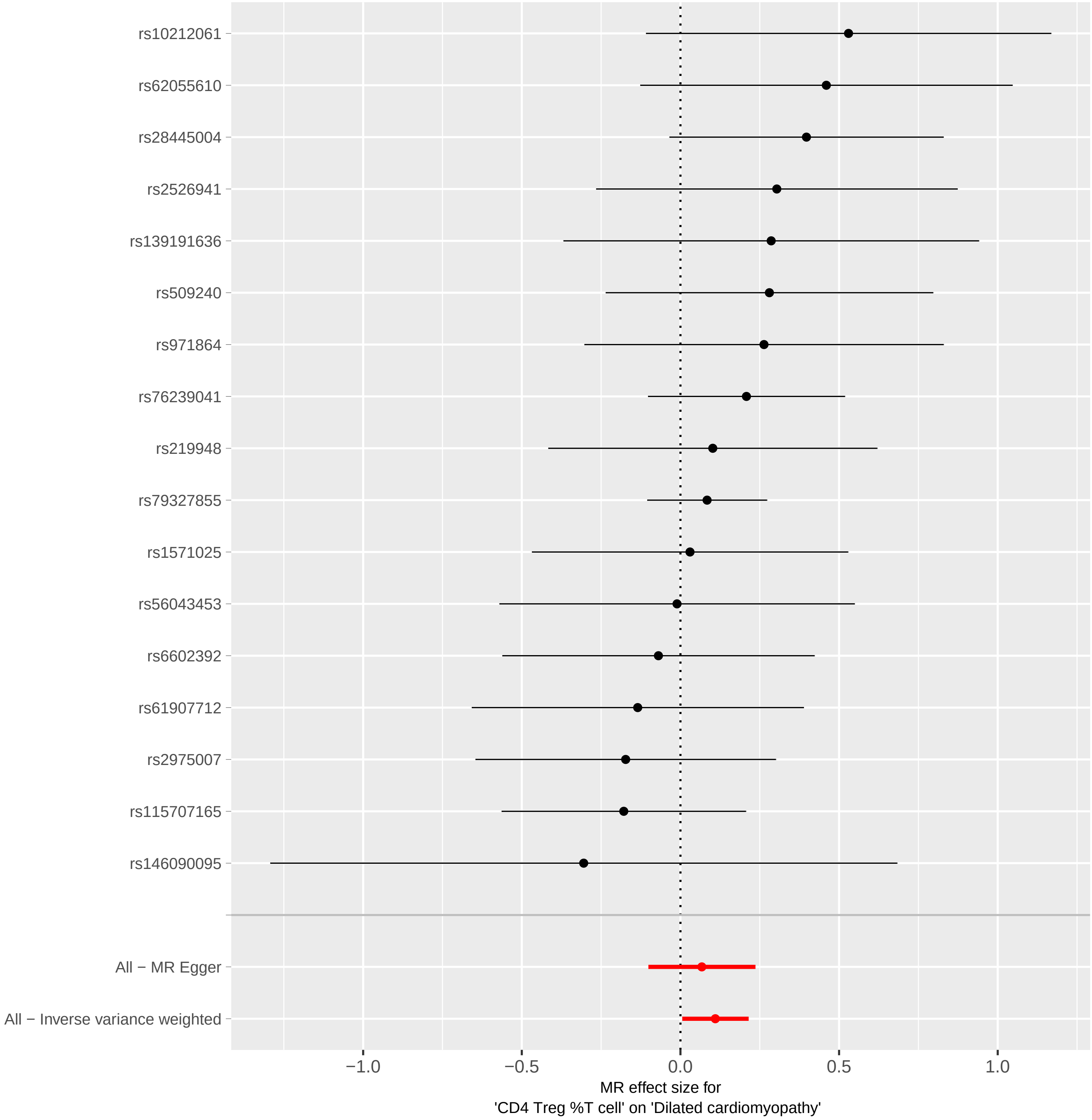
rs10212061
rs62055610
rs28445004
rs2526941
rs139191636
rs509240
rs971864
rs76239041
rs219948
rs79327855
rs1571025
rs56043453
rs6602392
rs61907712
rs2975007
rs115707165
rs146090095
All − MR Egger
All − Inverse variance weighted
−1.0
−0.5
0.0
0.5
1.0
MR effect size for
'CD4 Treg %T cell' on 'Dilated cardiomyopathy'

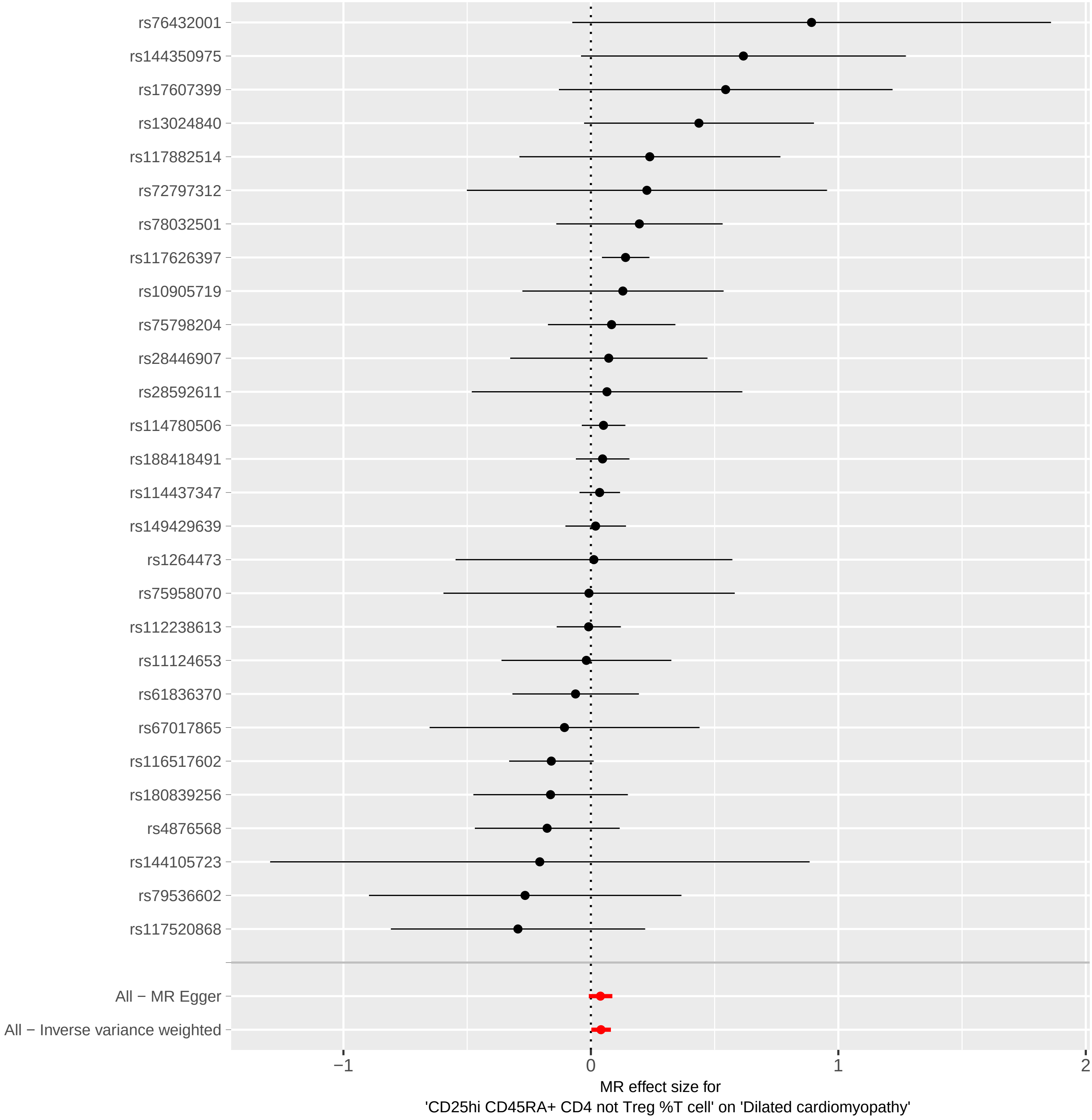

rs76432001
rs144350975
rs17607399
rs13024840
rs117882514
rs72797312
rs78032501
rs117626397
rs10905719
rs75798204
rs28446907
rs28592611
rs114780506
rs188418491
rs114437347
rs149429639
rs1264473
rs75958070
rs112238613
rs11124653
rs61836370
rs67017865
rs116517602
rs180839256
rs4876568
rs144105723
rs79536602
rs117520868
All − MR Egger
All − Inverse variance weighted
−1
0
1
2
MR effect size for
'CD25hi CD45RA+ CD4 not Treg %T cell' on 'Dilated cardiomyopathy'

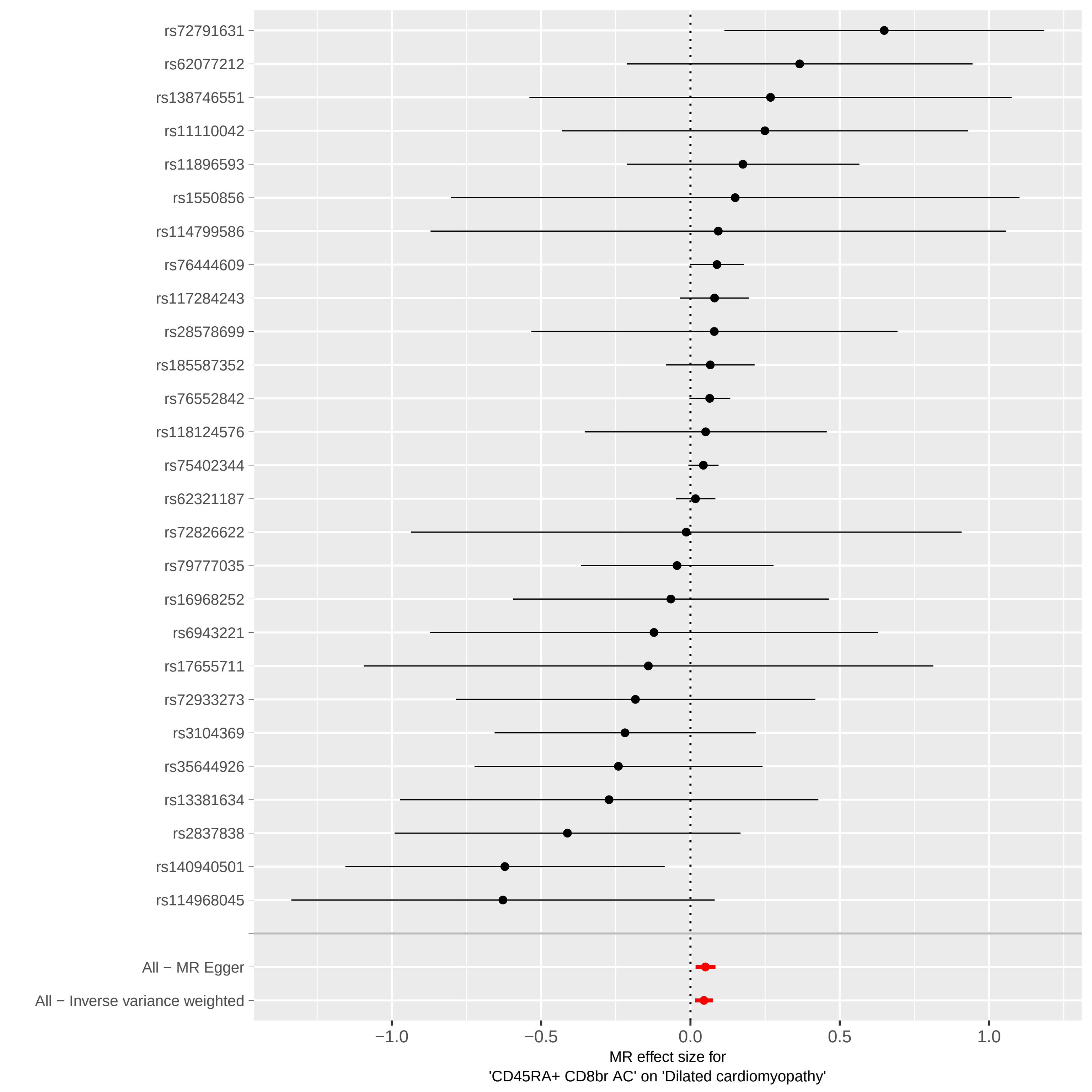
rs72791631
rs62077212
rs138746551
rs11110042
rs11896593
rs1550856
rs114799586
rs76444609
rs117284243
rs28578699
rs185587352
rs76552842
rs118124576
rs75402344
rs62321187
rs72826622
rs79777035
rs16968252
rs6943221
rs17655711
rs72933273
rs3104369
rs35644926
rs13381634
rs2837838
rs140940501
rs114968045
All − MR Egger
All − Inverse variance weighted
−1.0
−0.5
0.0
0.5
1.0
MR effect size for
'CD45RA+ CD8br AC' on 'Dilated cardiomyopathy'

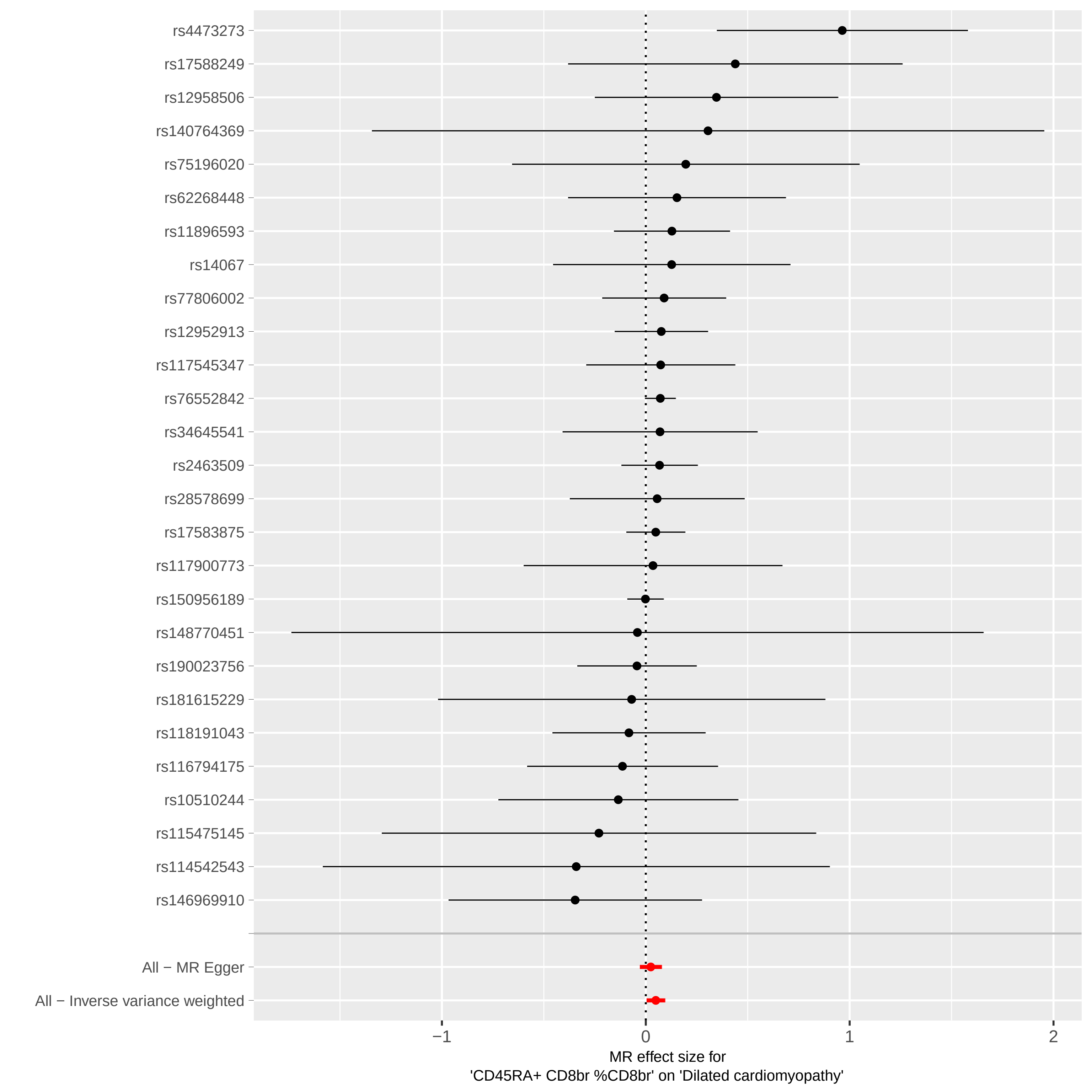

rs4473273
rs17588249
rs12958506
rs140764369
rs75196020
rs62268448
rs11896593
rs14067
rs77806002
rs12952913
rs117545347
rs76552842
rs34645541
rs2463509
rs28578699
rs17583875
rs117900773
rs150956189
rs148770451
rs190023756
rs181615229
rs118191043
rs116794175
rs10510244
rs115475145
rs114542543
rs146969910
All − MR Egger
All − Inverse variance weighted
−1
0
1
2
MR effect size for
'CD45RA+ CD8br %CD8br' on 'Dilated cardiomyopathy'

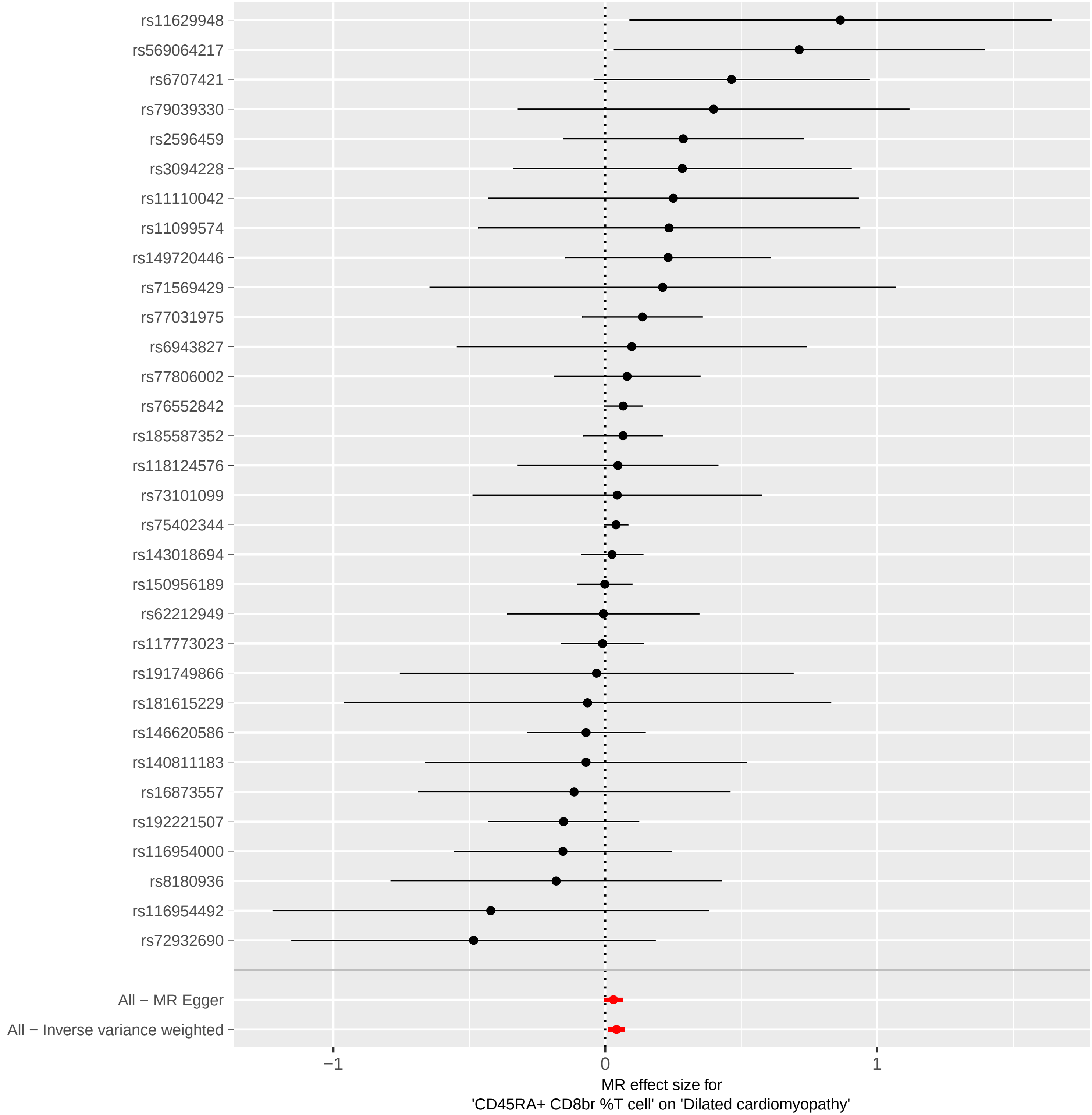
rs11629948
rs569064217
rs6707421
rs79039330
rs2596459
rs3094228
rs11110042
rs11099574
rs149720446
rs71569429
rs77031975
rs6943827
rs77806002
rs76552842
rs185587352
rs118124576
rs73101099
rs75402344
rs143018694
rs150956189
rs62212949
rs117773023
rs191749866
rs181615229
rs146620586
rs140811183
rs16873557
rs192221507
rs116954000
rs8180936
rs116954492
rs72932690
All − MR Egger
All − Inverse variance weighted
−1
0
1
MR effect size for
'CD45RA+ CD8br %T cell' on 'Dilated cardiomyopathy'

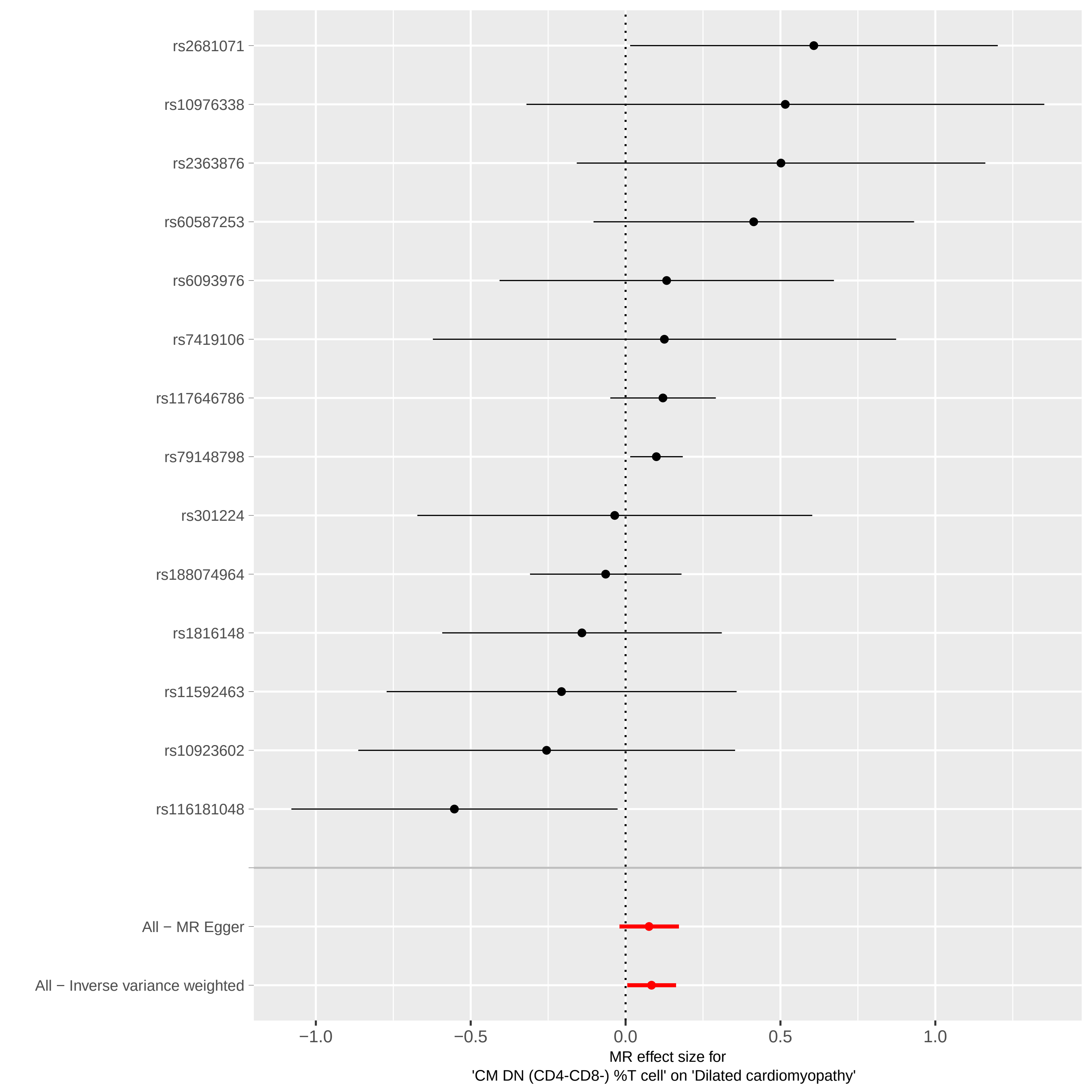
rs2681071
rs10976338
rs2363876
rs60587253
rs6093976
rs7419106
rs117646786
rs79148798
rs301224
rs188074964
rs1816148
rs11592463
rs10923602
rs116181048
All − MR Egger
All − Inverse variance weighted
−1.0
−0.5
0.0
0.5
1.0
MR effect size for
'CM DN (CD4-CD8-) %T cell' on 'Dilated cardiomyopathy'

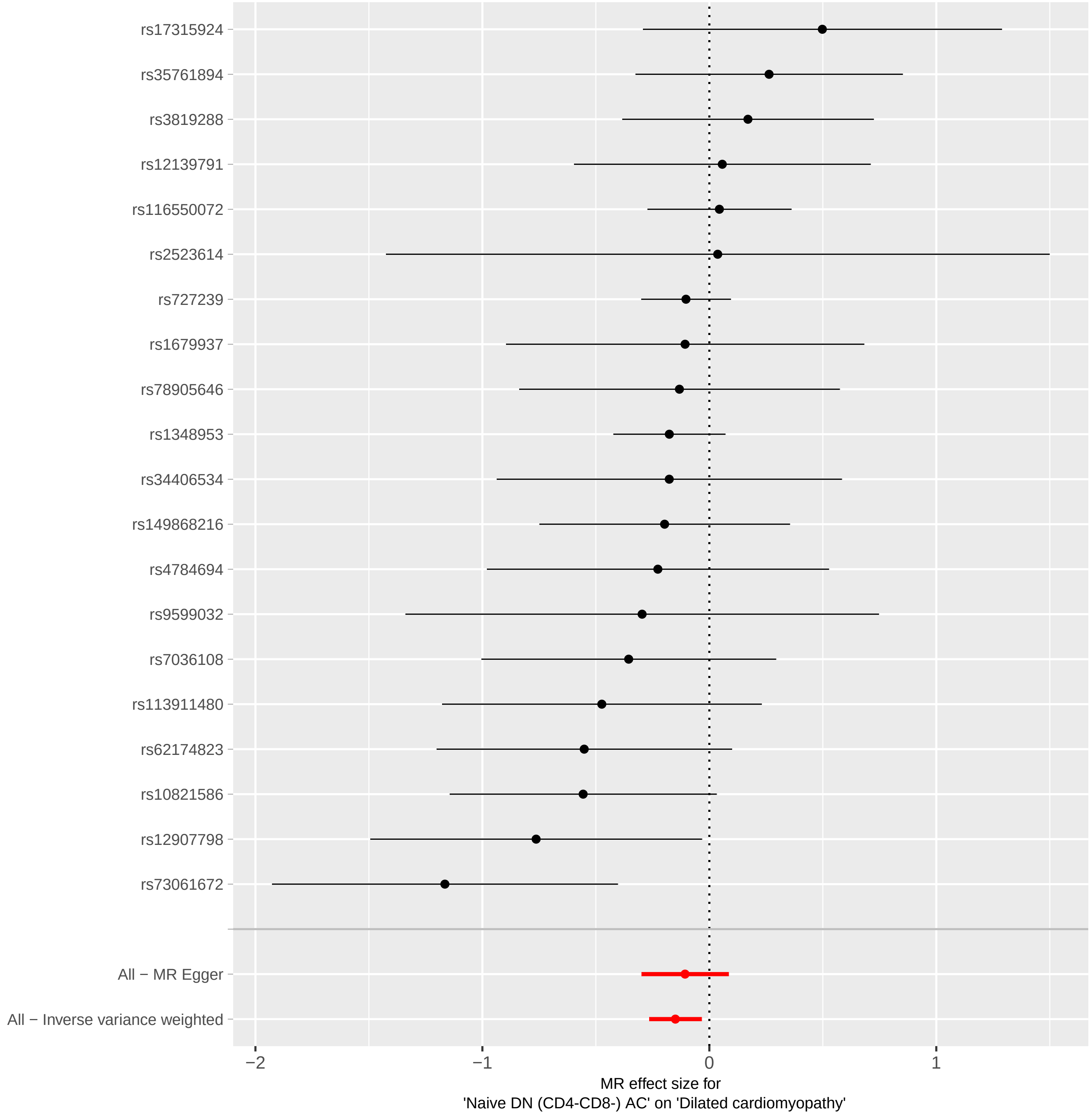
rs17315924
rs35761894
rs3819288
rs12139791
rs116550072
rs2523614
rs727239
rs1679937
rs78905646
rs1348953
rs34406534
rs149868216
rs4784694
rs9599032
rs7036108
rs113911480
rs62174823
rs10821586
rs12907798
rs73061672
All − MR Egger
All − Inverse variance weighted
−2
−1
0
1
MR effect size for
'Naive DN (CD4-CD8-) AC' on 'Dilated cardiomyopathy'

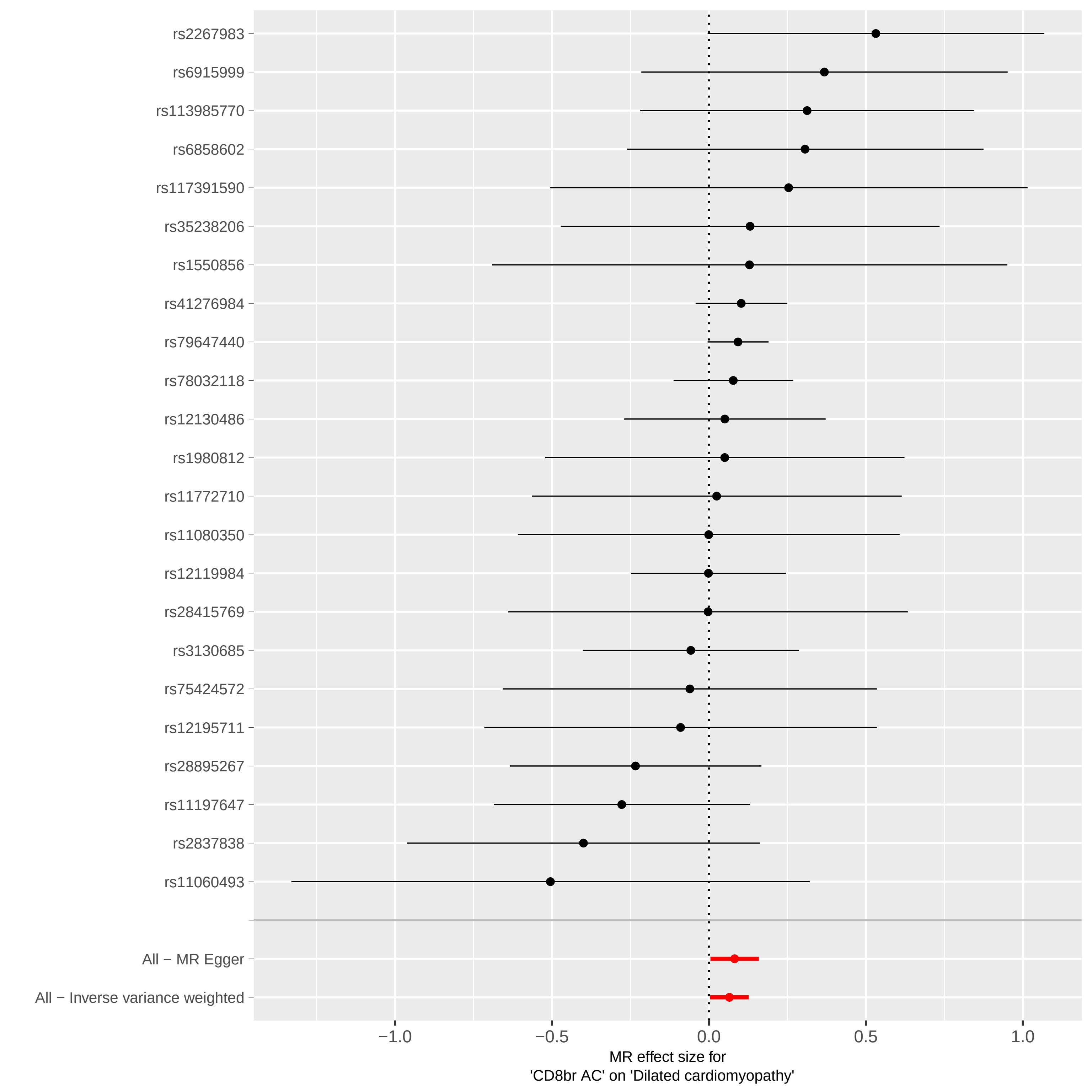

rs2267983
rs6915999
rs113985770
rs6858602
rs117391590
rs35238206
rs1550856
rs41276984
rs79647440
rs78032118
rs12130486
rs1980812
rs11772710
rs11080350
rs12119984
rs28415769
rs3130685
rs75424572
rs12195711
rs28895267
rs11197647
rs2837838
rs11060493
All − MR Egger
All − Inverse variance weighted
−1.0
−0.5
0.0
0.5
1.0
MR effect size for
'CD8br AC' on 'Dilated cardiomyopathy'

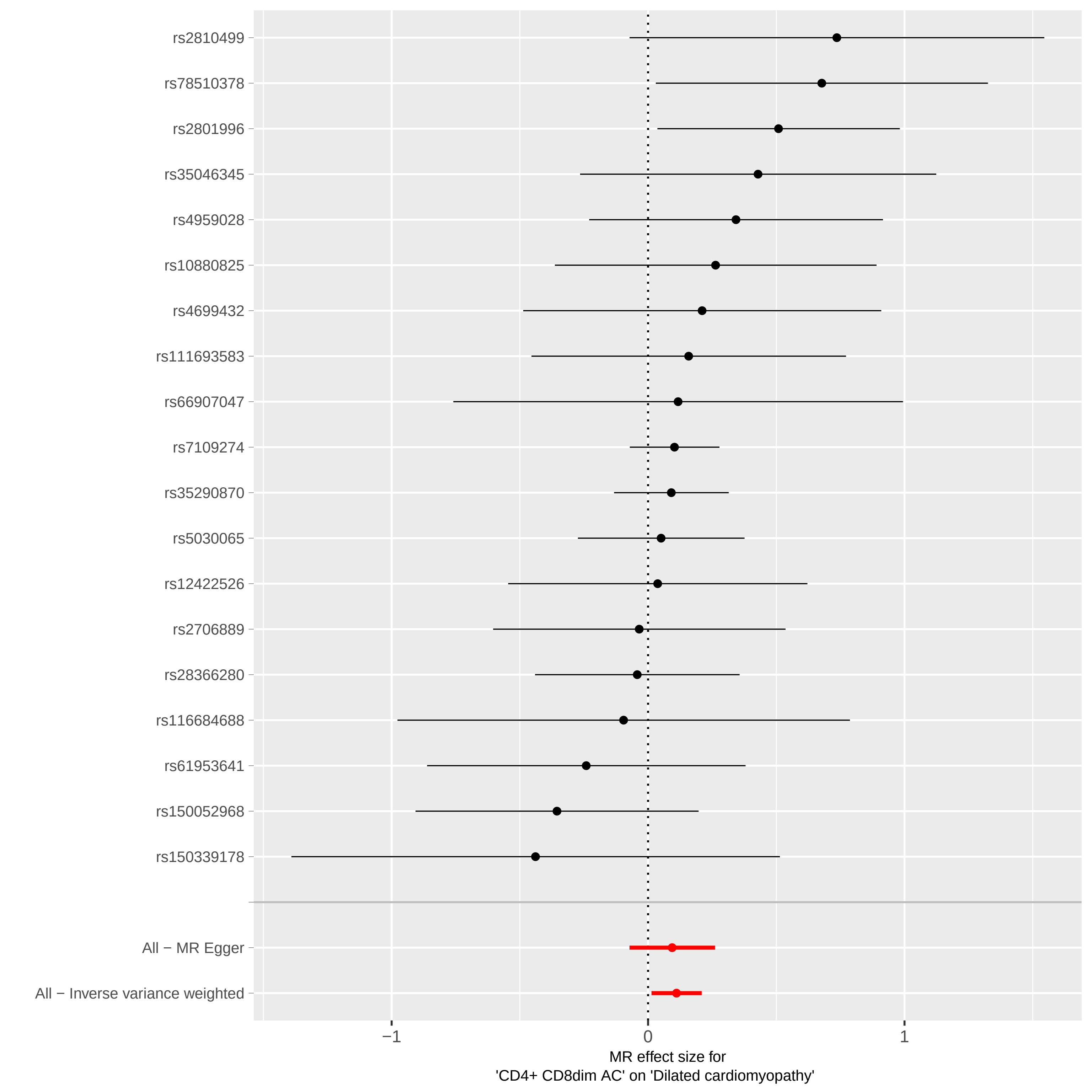
rs2810499
rs78510378
rs2801996
rs35046345
rs4959028
rs10880825
rs4699432
rs111693583
rs66907047
rs7109274
rs35290870
rs5030065
rs12422526
rs2706889
rs28366280
rs116684688
rs61953641
rs150052968
rs150339178
All − MR Egger
All − Inverse variance weighted
−1
0
1
MR effect size for
'CD4+ CD8dim AC' on 'Dilated cardiomyopathy'

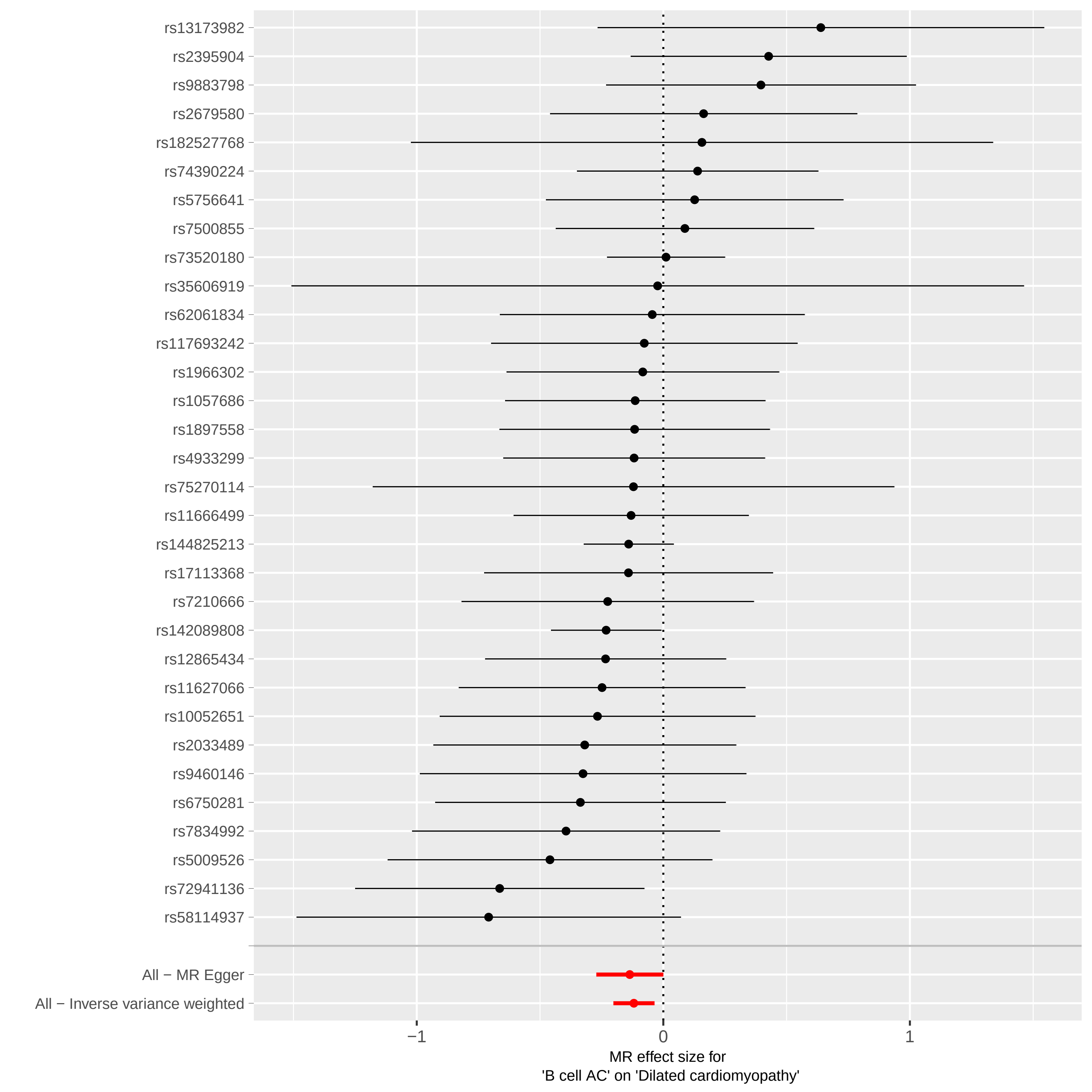
rs13173982
rs2395904
rs9883798
rs2679580
rs182527768
rs74390224
rs5756641
rs7500855
rs73520180
rs35606919
rs62061834
rs117693242
rs1966302
rs1057686
rs1897558
rs4933299
rs75270114
rs11666499
rs144825213
rs17113368
rs7210666
rs142089808
rs12865434
rs11627066
rs10052651
rs2033489
rs9460146
rs6750281
rs7834992
rs5009526
rs72941136
rs58114937
All − MR Egger
All − Inverse variance weighted
−1
0
1
MR effect size for
'B cell AC' on 'Dilated cardiomyopathy'

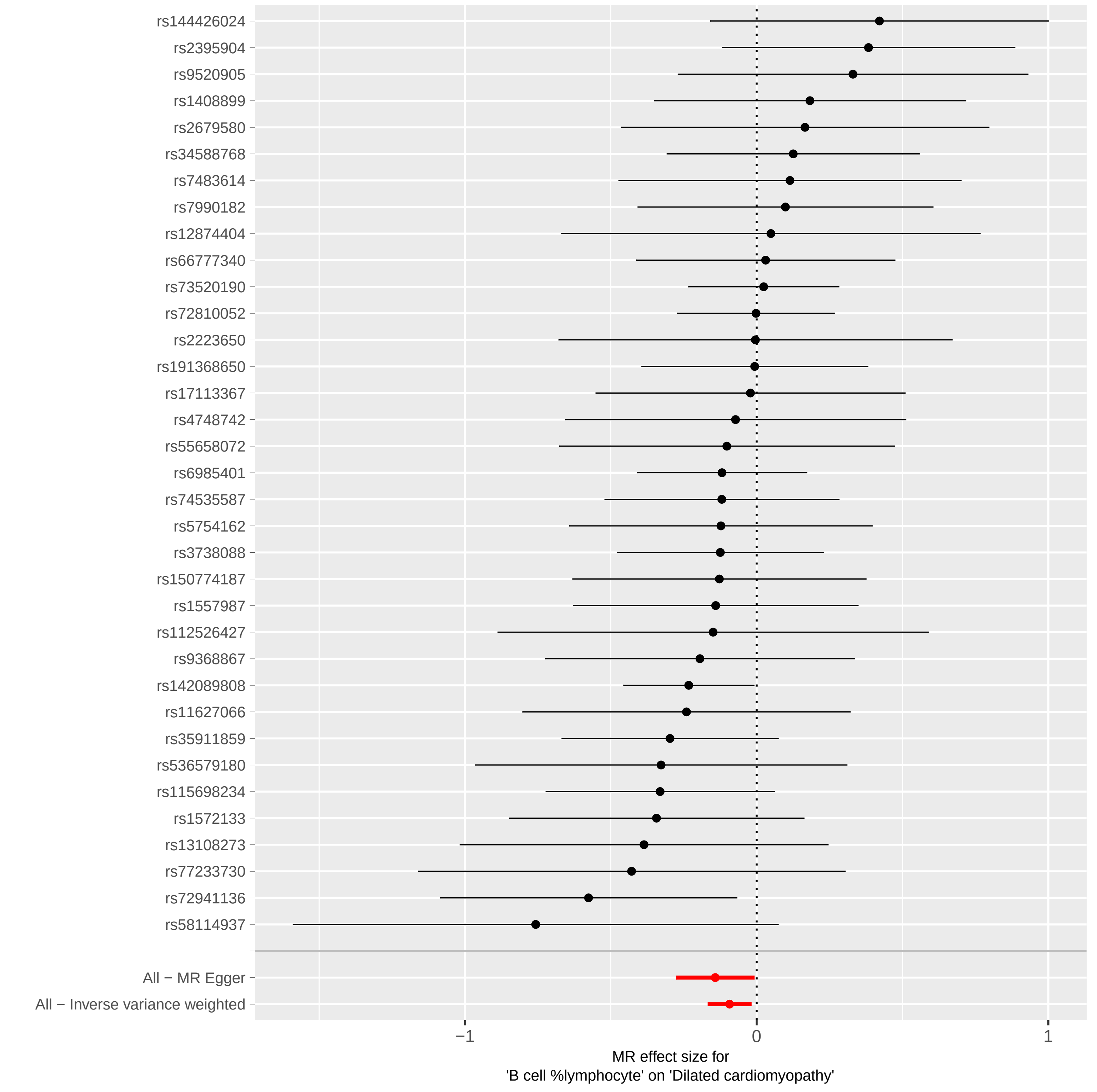
rs144426024
rs2395904
rs9520905
rs1408899
rs2679580
rs34588768
rs7483614
rs7990182
rs12874404
rs66777340
rs73520190
rs72810052
rs2223650
rs191368650
rs17113367
rs4748742
rs55658072
rs6985401
rs74535587
rs5754162
rs3738088
rs150774187
rs1557987
rs112526427
rs9368867
rs142089808
rs11627066
rs35911859
rs536579180
rs115698234
rs1572133
rs13108273
rs77233730
rs72941136
rs58114937
All − MR Egger
All − Inverse variance weighted
−1
0
1
MR effect size for
'B cell %lymphocyte' on 'Dilated cardiomyopathy'

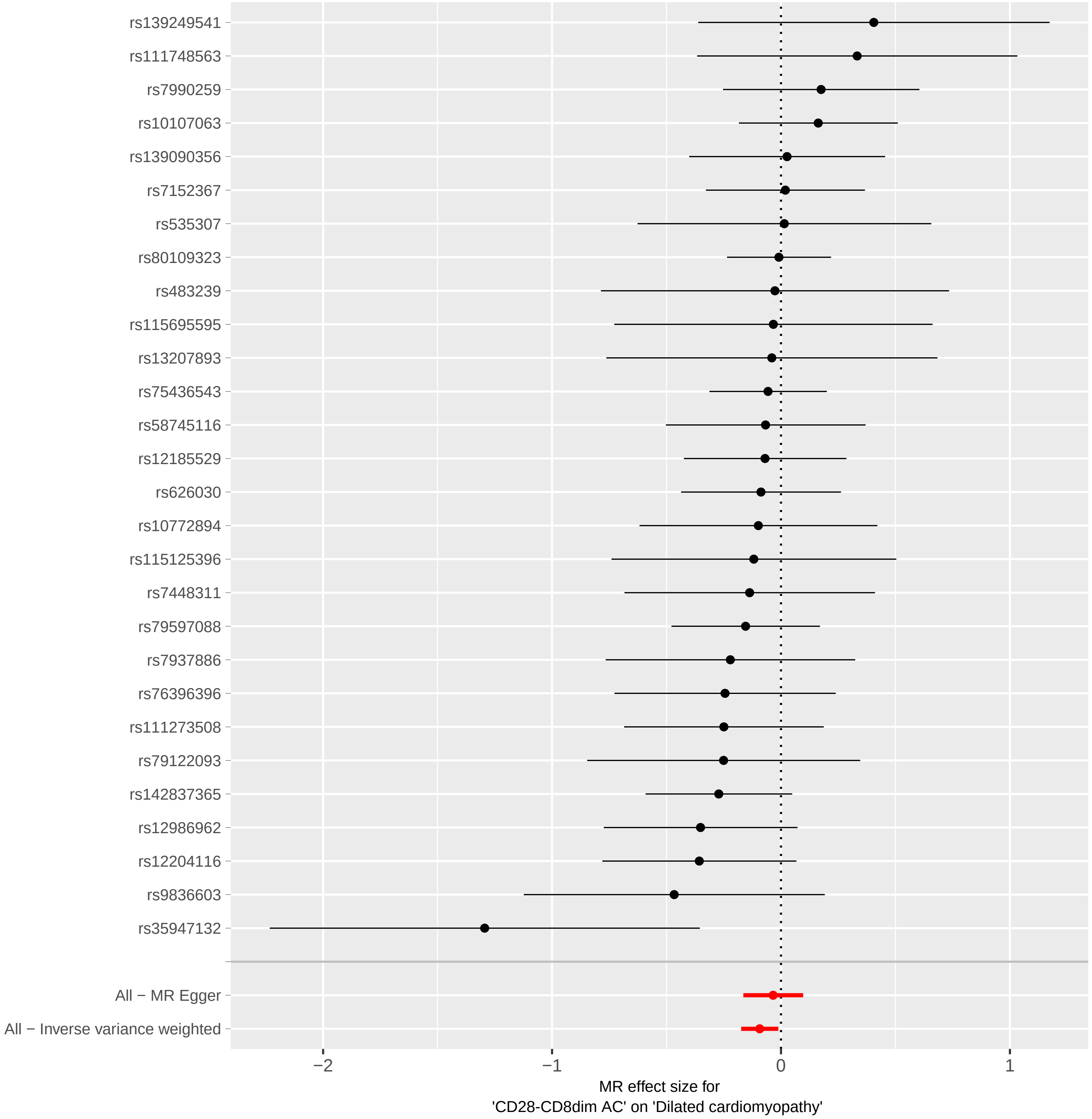
rs139249541
rs111748563
rs7990259
rs10107063
rs139090356
rs7152367
rs535307
rs80109323
rs483239
rs115695595
rs13207893
rs75436543
rs58745116
rs12185529
rs626030
rs10772894
rs115125396
rs7448311
rs79597088
rs7937886
rs76396396
rs111273508
rs79122093
rs142837365
rs12986962
rs12204116
rs9836603
rs35947132
All − MR Egger
All − Inverse variance weighted
−2
−1
0
1
MR effect size for
'CD28-CD8dim AC' on 'Dilated cardiomyopathy'

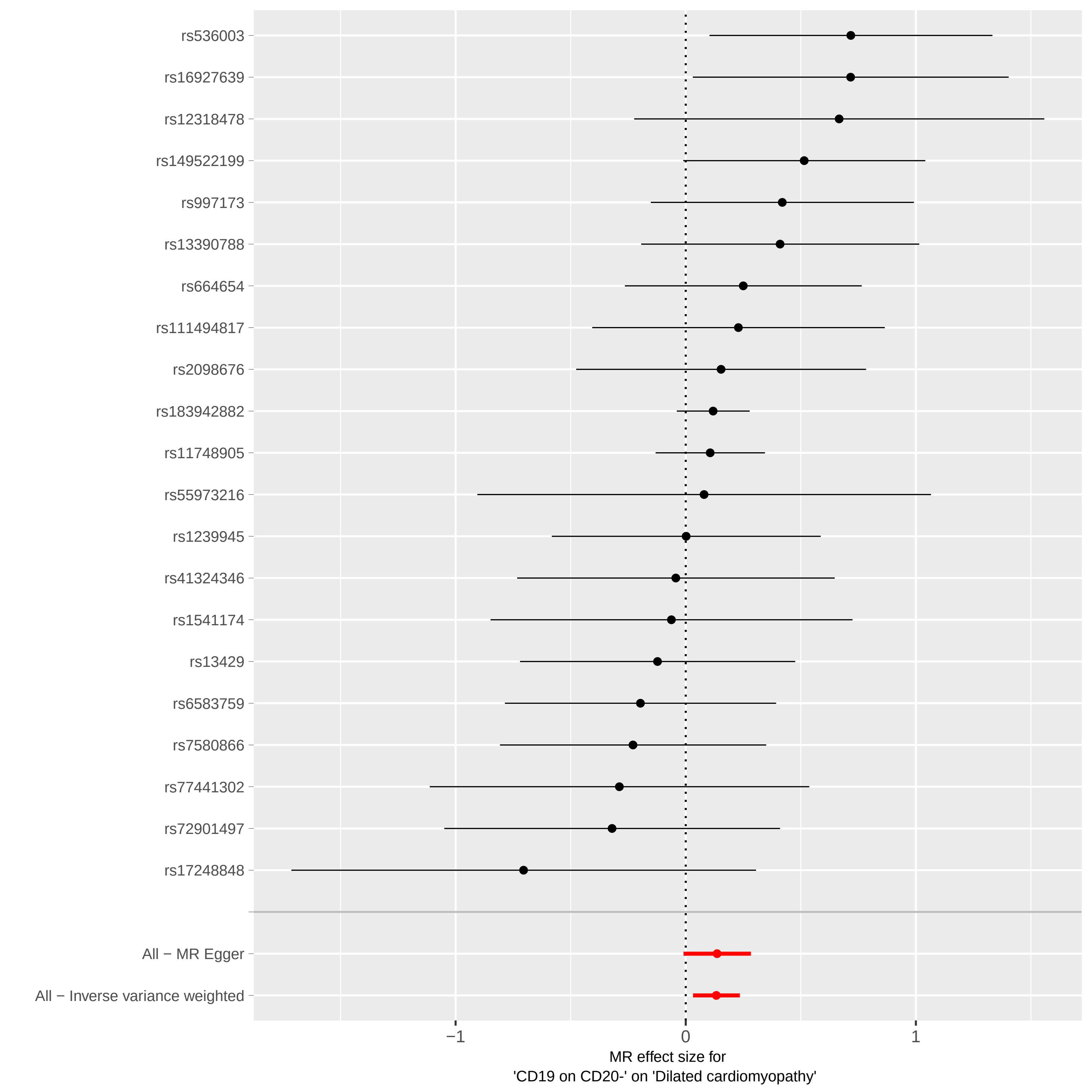
rs536003
rs16927639
rs12318478
rs149522199
rs997173
rs13390788
rs664654
rs111494817
rs2098676
rs183942882
rs11748905
rs55973216
rs1239945
rs41324346
rs1541174
rs13429
rs6583759
rs7580866
rs77441302
rs72901497
rs17248848
All − MR Egger
All − Inverse variance weighted
−1
0
1
MR effect size for
'CD19 on CD20-' on 'Dilated cardiomyopathy'

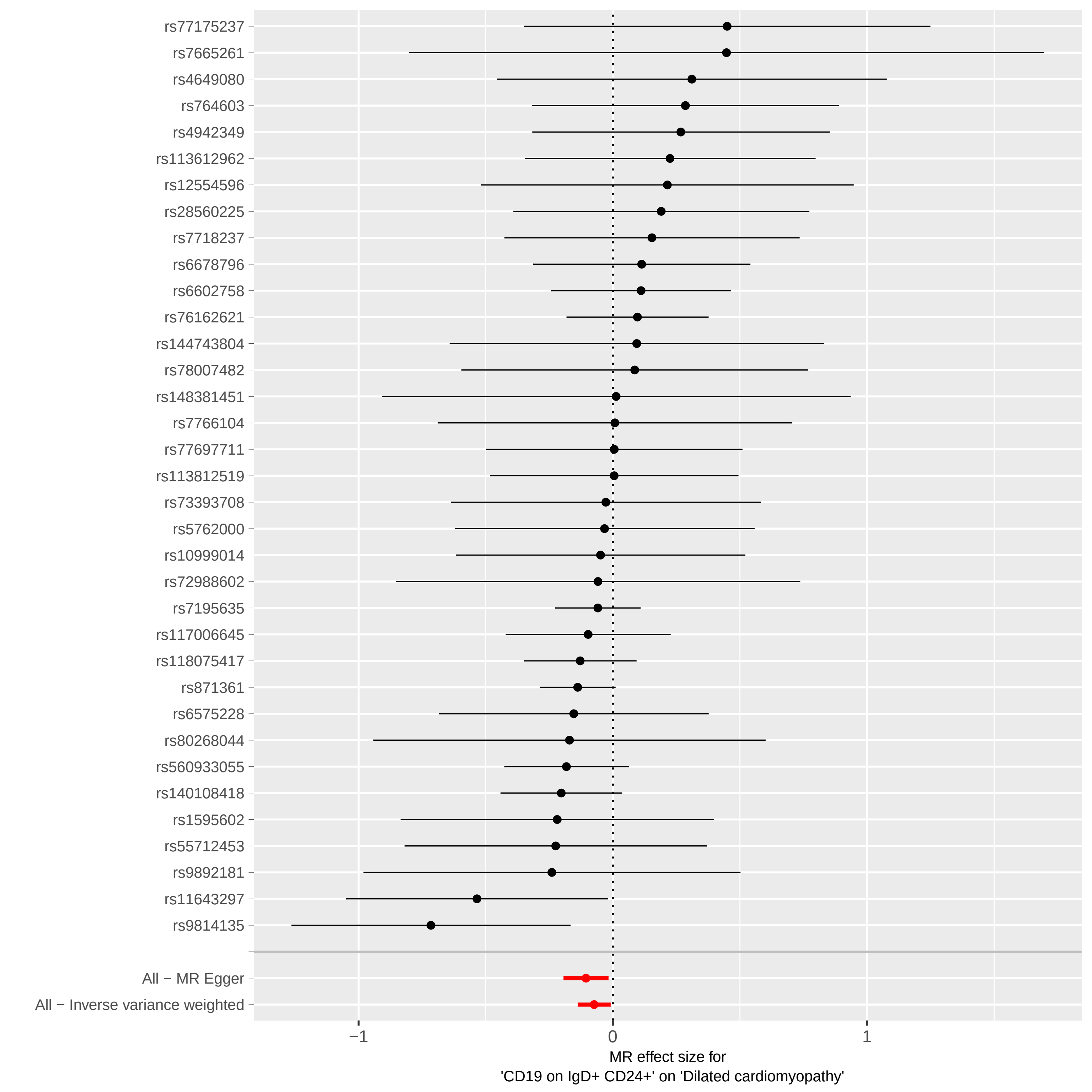
rs77175237
rs7665261
rs4649080
rs764603
rs4942349
rs113612962
rs12554596
rs28560225
rs7718237
rs6678796
rs6602758
rs76162621
rs144743804
rs78007482
rs148381451
rs7766104
rs77697711
rs113812519
rs73393708
rs5762000
rs10999014
rs72988602
rs7195635
rs117006645
rs118075417
rs871361
rs6575228
rs80268044
rs560933055
rs140108418
rs1595602
rs55712453
rs9892181
rs11643297
rs9814135
All − MR Egger
All − Inverse variance weighted
−1
0
1
MR effect size for
'CD19 on IgD+ CD24+' on 'Dilated cardiomyopathy'

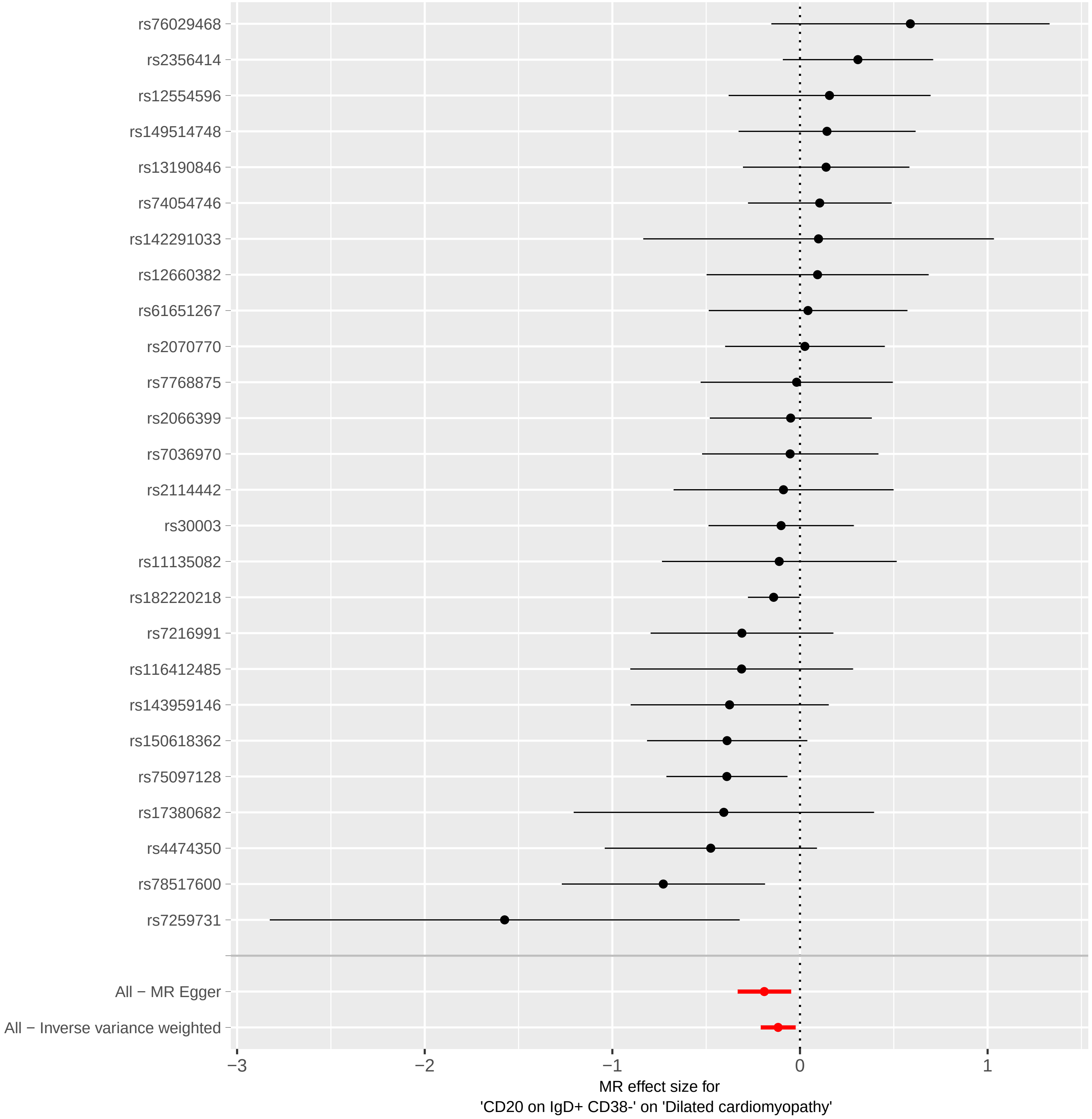
rs76029468
rs2356414
rs12554596
rs149514748
rs13190846
rs74054746
rs142291033
rs12660382
rs61651267
rs2070770
rs7768875
rs2066399
rs7036970
rs2114442
rs30003
rs11135082
rs182220218
rs7216991
rs116412485
rs143959146
rs150618362
rs75097128
rs17380682
rs4474350
rs78517600
rs7259731
All − MR Egger
All − Inverse variance weighted
−3
−2
−1
0
1
MR effect size for
'CD20 on IgD+ CD38-' on 'Dilated cardiomyopathy'

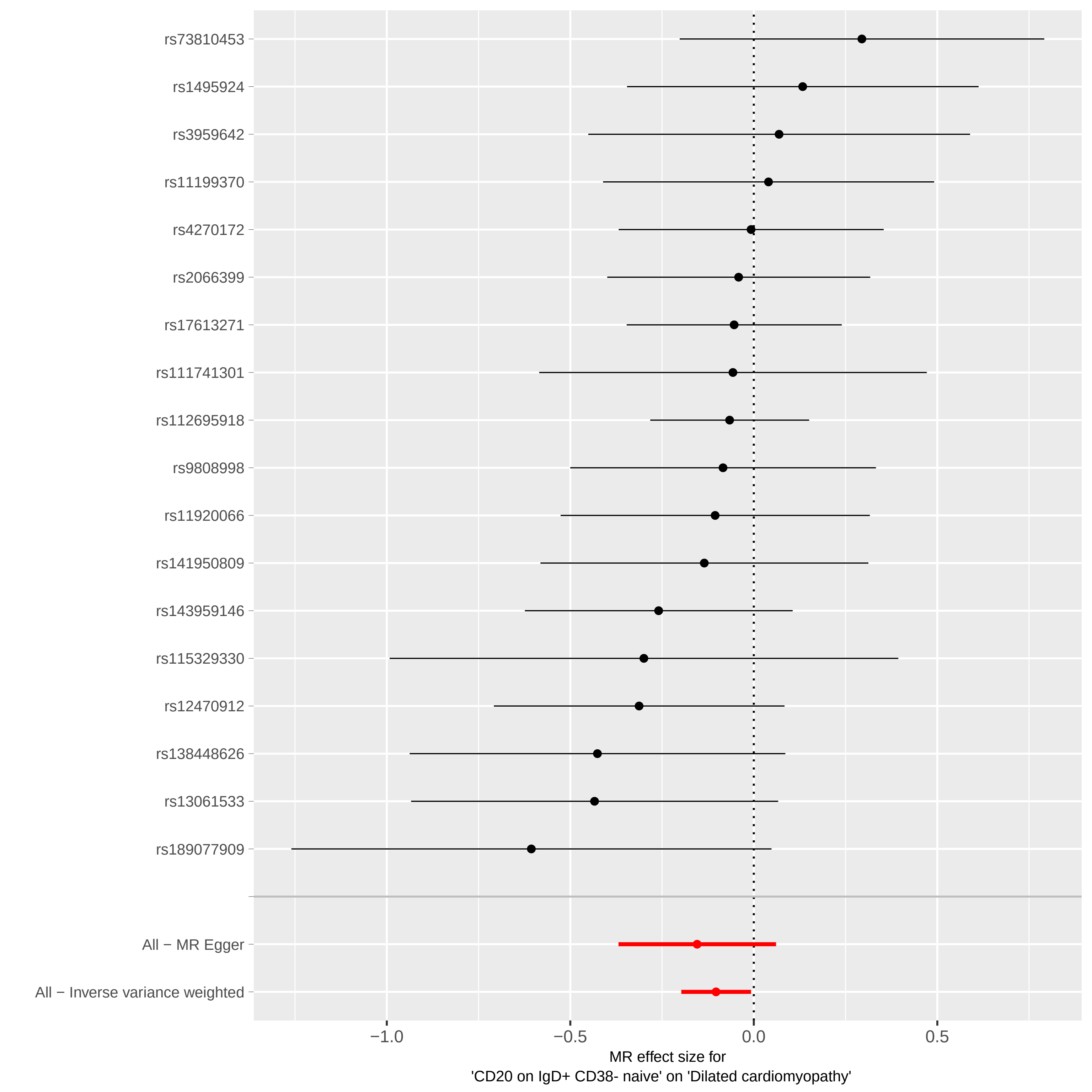

rs73810453
rs1495924
rs3959642
rs11199370
rs4270172
rs2066399
rs17613271
rs111741301
rs112695918
rs9808998
rs11920066
rs141950809
rs143959146
rs115329330
rs12470912
rs138448626
rs13061533
rs189077909
All − MR Egger
All − Inverse variance weighted
−1.0
−0.5
0.0
0.5
MR effect size for
'CD20 on IgD+ CD38- naive' on 'Dilated cardiomyopathy'

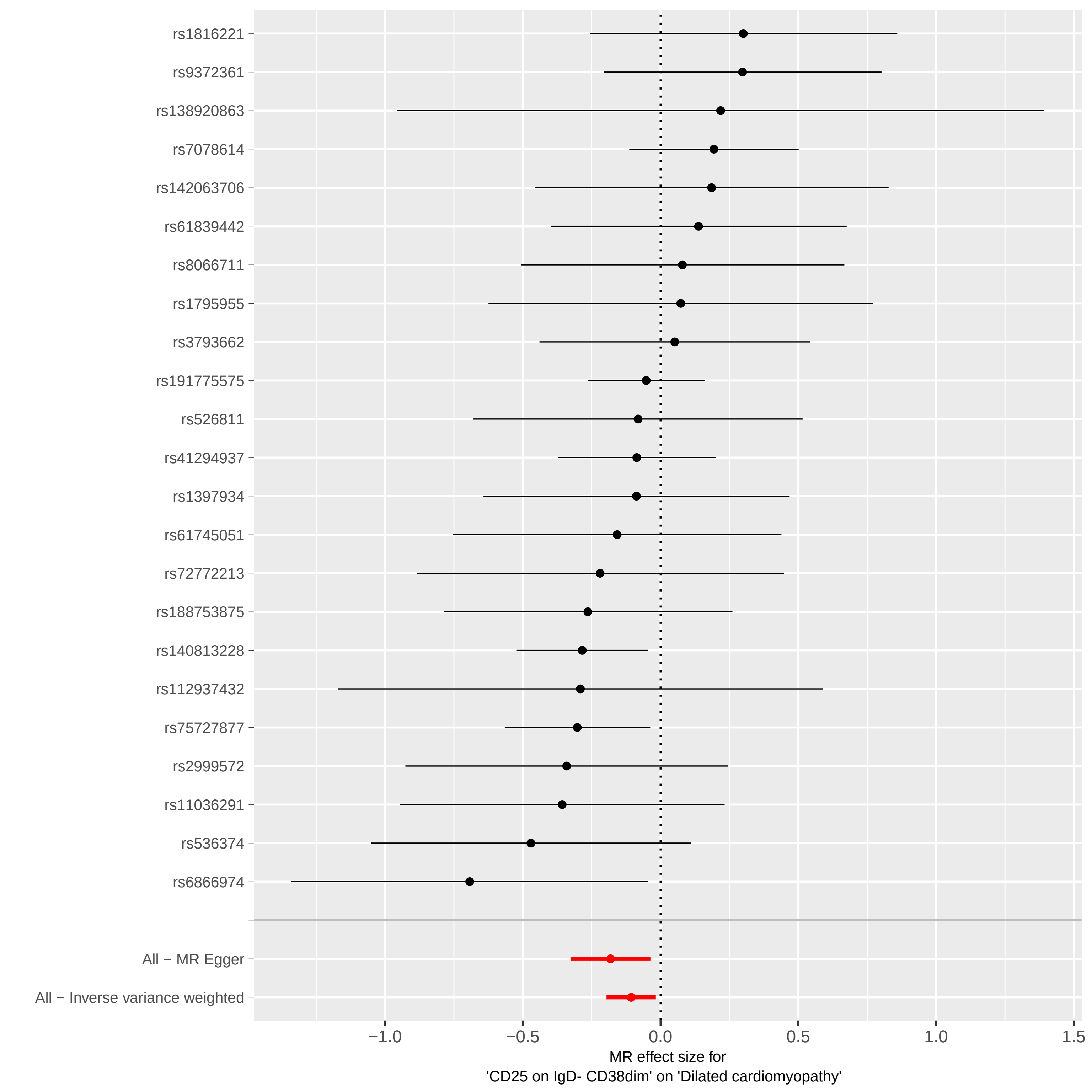
rs1816221
rs9372361
rs138920863
rs7078614
rs142063706
rs61839442
rs8066711
rs1795955
rs3793662
rs191775575
rs526811
rs41294937
rs1397934
rs61745051
rs72772213
rs188753875
rs140813228
rs112937432
rs75727877
rs2999572
rs11036291
rs536374
rs6866974
All − MR Egger
All − Inverse variance weighted
−1.0
−0.5
0.0
0.5
1.0
1.5
MR effect size for
'CD25 on IgD- CD38dim' on 'Dilated cardiomyopathy'

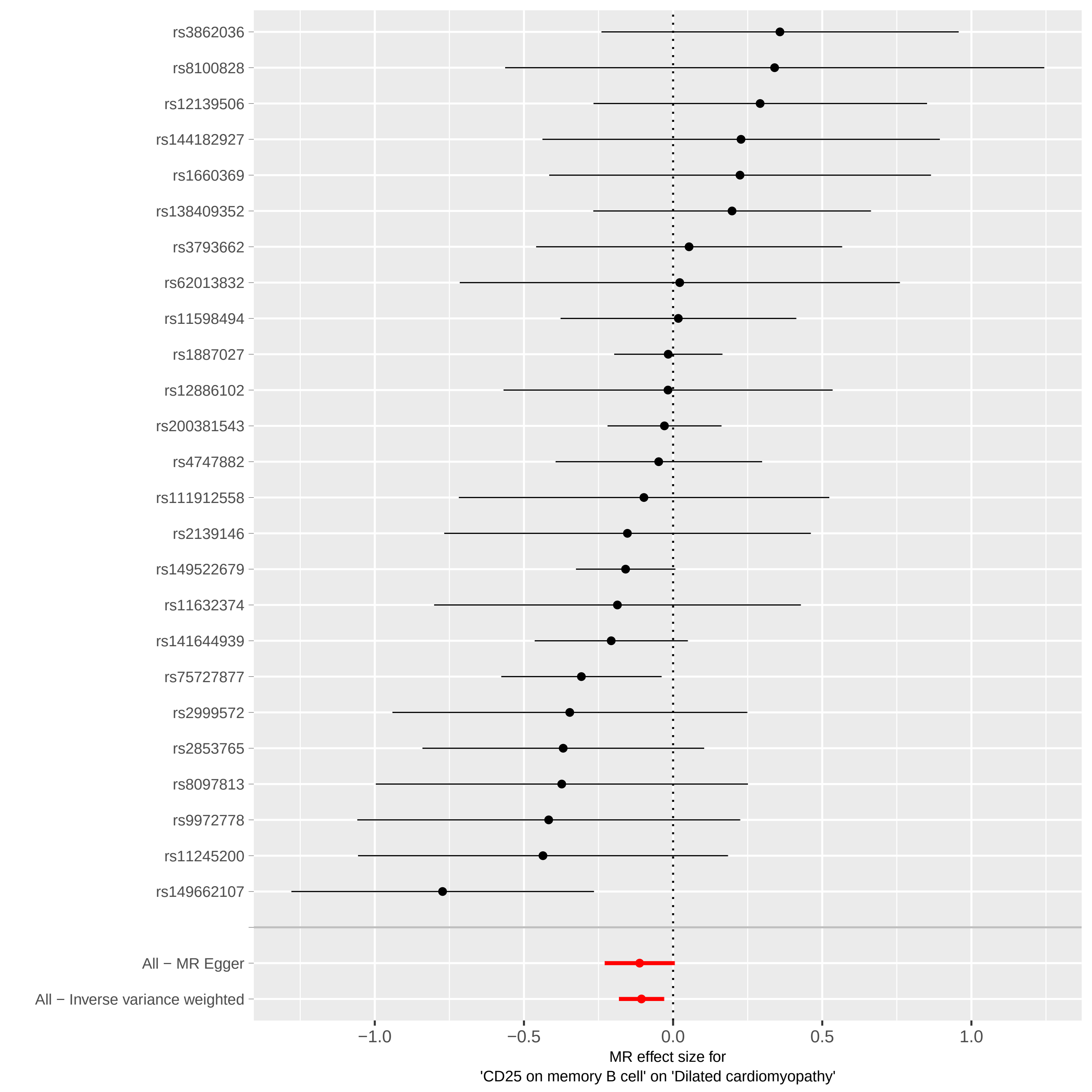
rs3862036
rs8100828
rs12139506
rs144182927
rs1660369
rs138409352
rs3793662
rs62013832
rs11598494
rs1887027
rs12886102
rs200381543
rs4747882
rs111912558
rs2139146
rs149522679
rs11632374
rs141644939
rs75727877
rs2999572
rs2853765
rs8097813
rs9972778
rs11245200
rs149662107
All − MR Egger
All − Inverse variance weighted
−1.0
−0.5
0.0
0.5
1.0
MR effect size for
'CD25 on memory B cell' on 'Dilated cardiomyopathy'

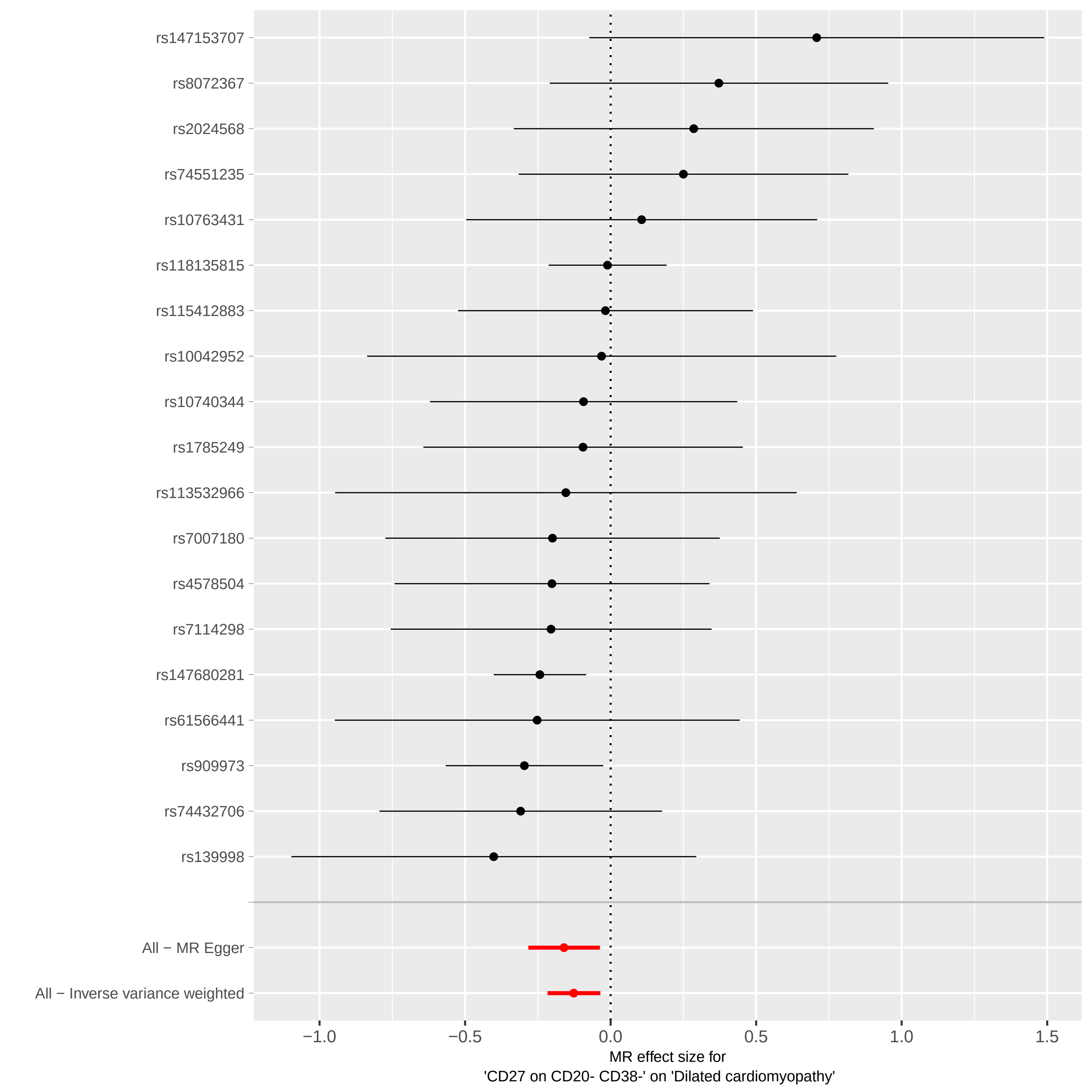
rs147153707
rs8072367
rs2024568
rs74551235
rs10763431
rs118135815
rs115412883
rs10042952
rs10740344
rs1785249
rs113532966
rs7007180
rs4578504
rs7114298
rs147680281
rs61566441
rs909973
rs74432706
rs139998
All − MR Egger
All − Inverse variance weighted
−1.0
−0.5
0.0
0.5
1.0
1.5
MR effect size for
'CD27 on CD20- CD38-' on 'Dilated cardiomyopathy'

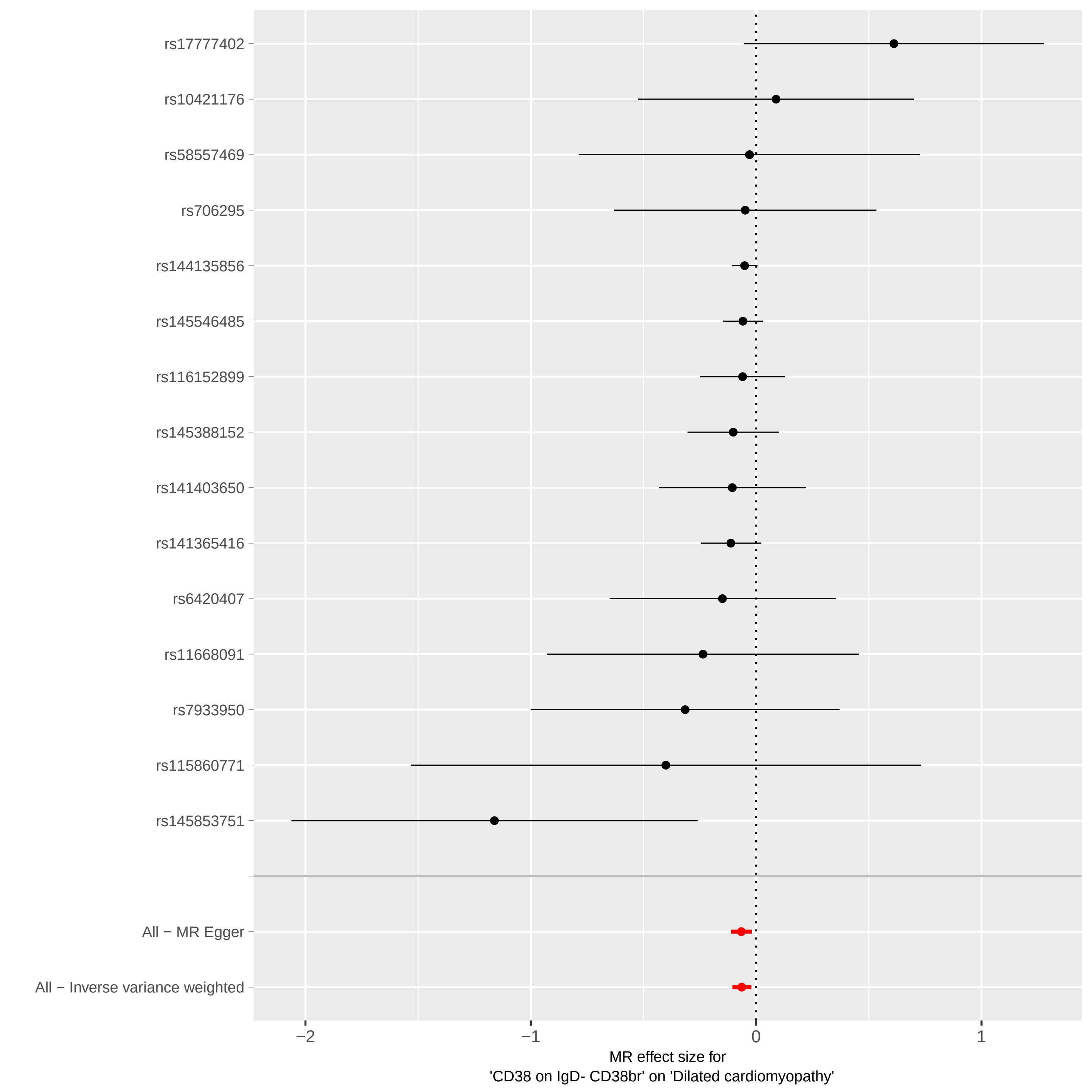

rs17777402
rs10421176
rs58557469
rs706295
rs144135856
rs145546485
rs116152899
rs145388152
rs141403650
rs141365416
rs6420407
rs11668091
rs7933950
rs115860771
rs145853751
All − MR Egger
All − Inverse variance weighted
−2
−1
0
1
MR effect size for
'CD38 on IgD- CD38br' on 'Dilated cardiomyopathy'

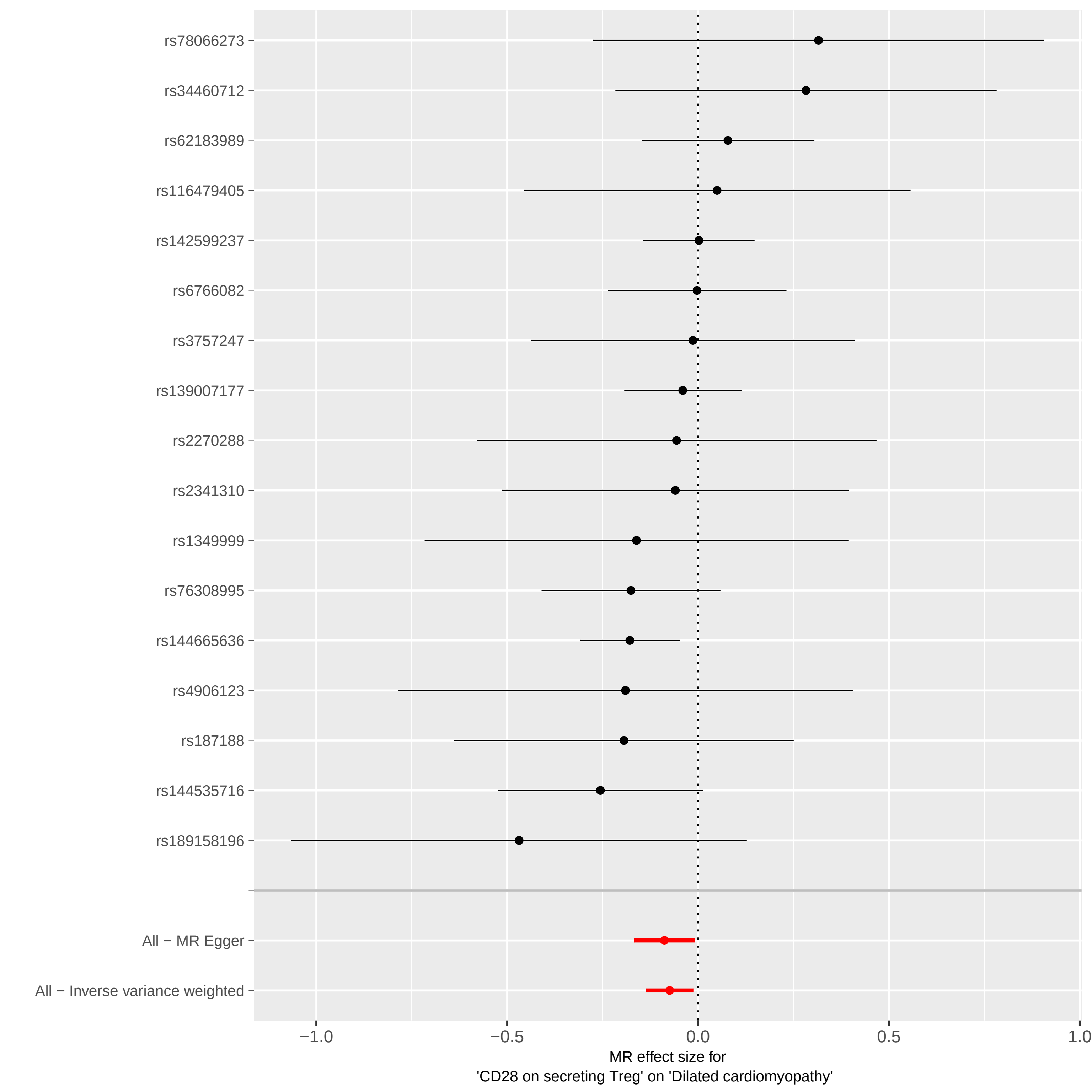
rs78066273
rs34460712
rs62183989
rs116479405
rs142599237
rs6766082
rs3757247
rs139007177
rs2270288
rs2341310
rs1349999
rs76308995
rs144665636
rs4906123
rs187188
rs144535716
rs189158196
All − MR Egger
All − Inverse variance weighted
−1.0
−0.5
0.0
0.5
1.0
MR effect size for
'CD28 on secreting Treg' on 'Dilated cardiomyopathy'

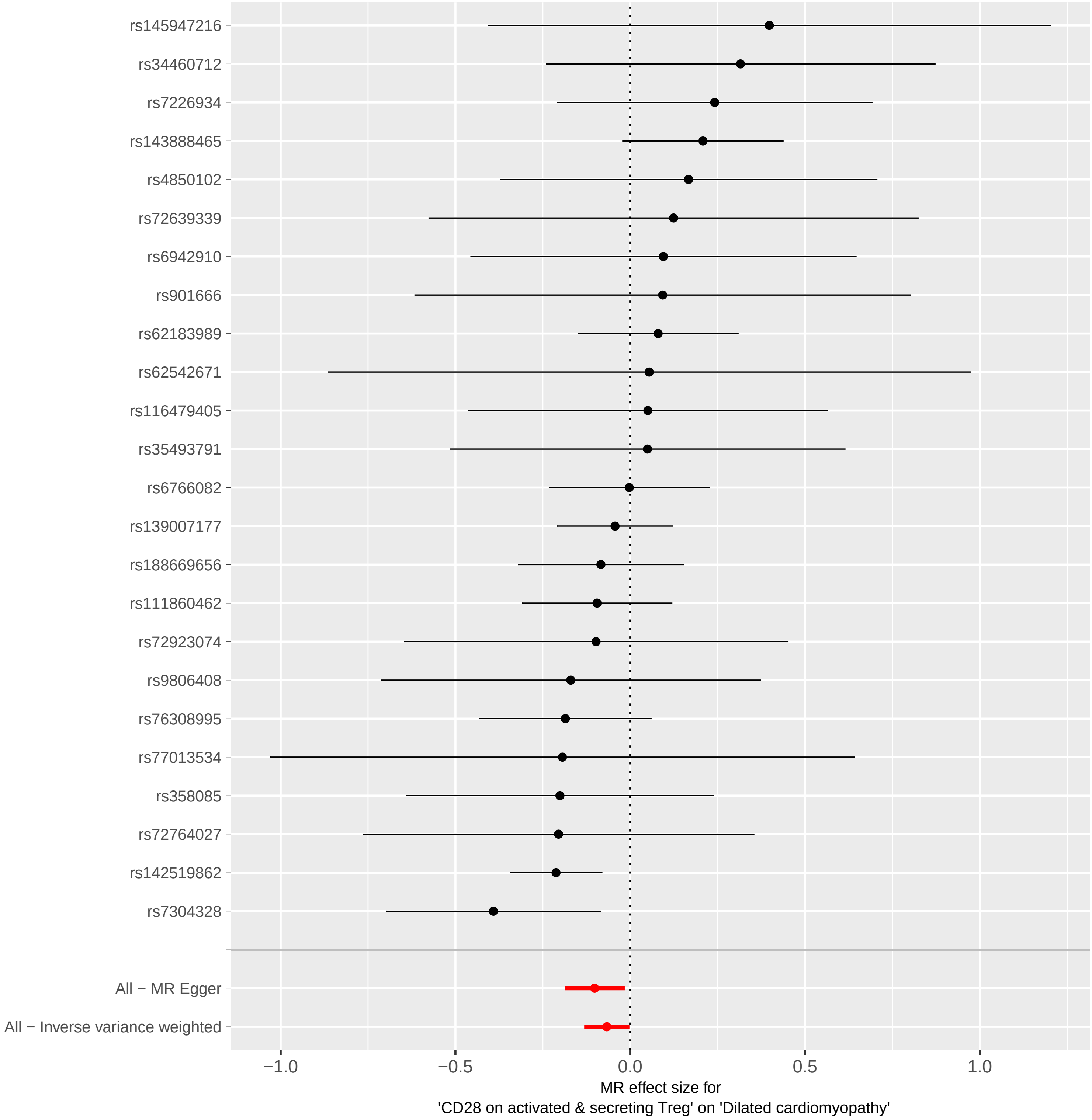
rs145947216
rs34460712
rs7226934
rs143888465
rs4850102
rs72639339
rs6942910
rs901666
rs62183989
rs62542671
rs116479405
rs35493791
rs6766082
rs139007177
rs188669656
rs111860462
rs72923074
rs9806408
rs76308995
rs77013534
rs358085
rs72764027
rs142519862
rs7304328
All − MR Egger
All − Inverse variance weighted
−1.0
−0.5
0.0
0.5
1.0
MR effect size for
'CD28 on activated & secreting Treg' on 'Dilated cardiomyopathy'

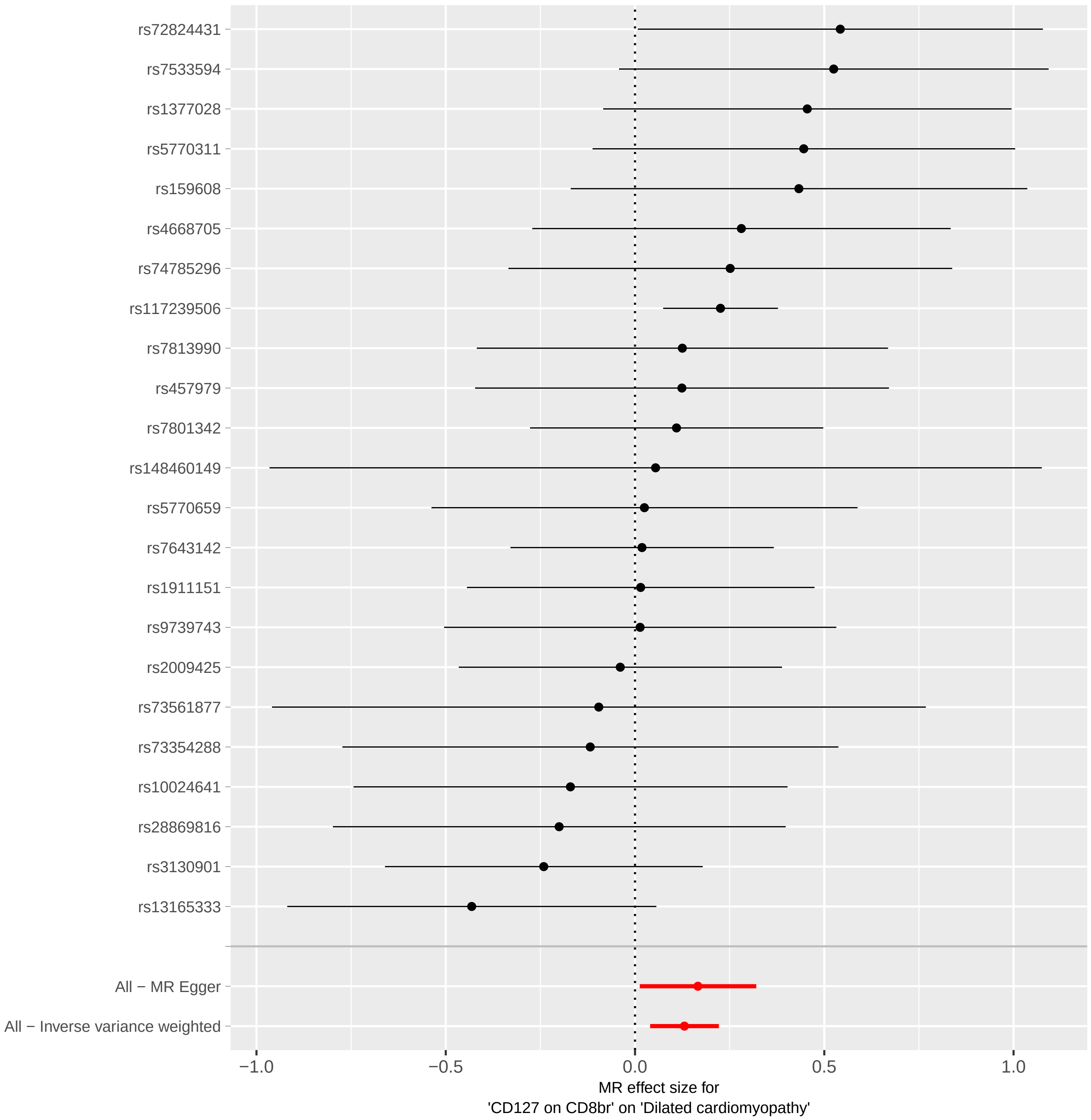
rs72824431
rs7533594
rs1377028
rs5770311
rs159608
rs4668705
rs74785296
rs117239506
rs7813990
rs457979
rs7801342
rs148460149
rs5770659
rs7643142
rs1911151
rs9739743
rs2009425
rs73561877
rs73354288
rs10024641
rs28869816
rs3130901
rs13165333
All − MR Egger
All − Inverse variance weighted
−1.0
−0.5
0.0
0.5
1.0
MR effect size for
'CD127 on CD8br' on 'Dilated cardiomyopathy'

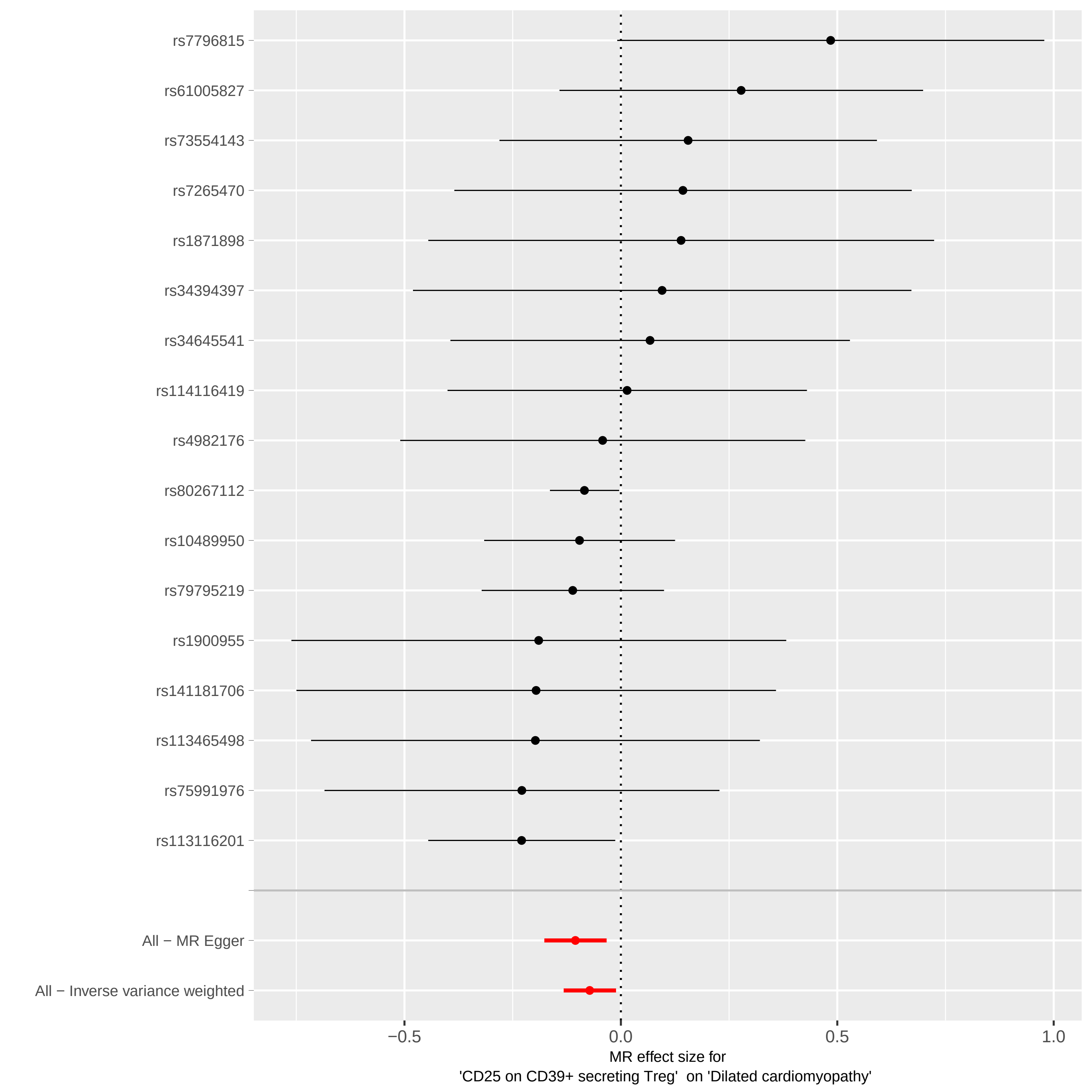
rs7796815
rs61005827
rs73554143
rs7265470
rs1871898
rs34394397
rs34645541
rs114116419
rs4982176
rs80267112
rs10489950
rs79795219
rs1900955
rs141181706
rs113465498
rs75991976
rs113116201
All − MR Egger
All − Inverse variance weighted
−0.5
0.0
0.5
1.0
MR effect size for
'CD25 on CD39+ secreting Treg' on 'Dilated cardiomyopathy'

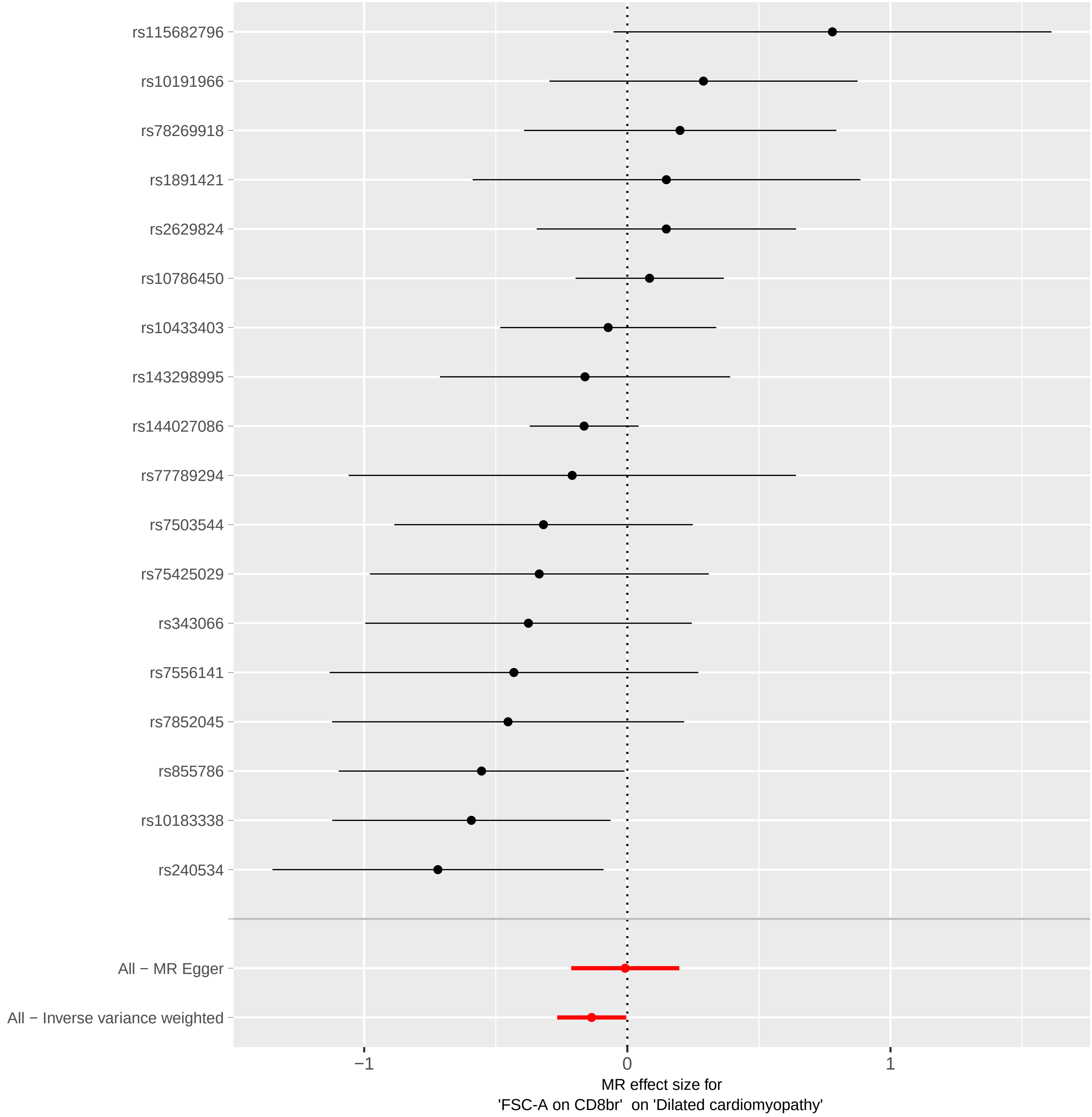
rs115682796
rs10191966
rs78269918
rs1891421
rs2629824
rs10786450
rs10433403
rs143298995
rs144027086
rs77789294
rs7503544
rs75425029
rs343066
rs7556141
rs7852045
rs855786
rs10183338
rs240534
All − MR Egger
All − Inverse variance weighted
−1
0
1
MR effect size for
'FSC-A on CD8br' on 'Dilated cardiomyopathy'

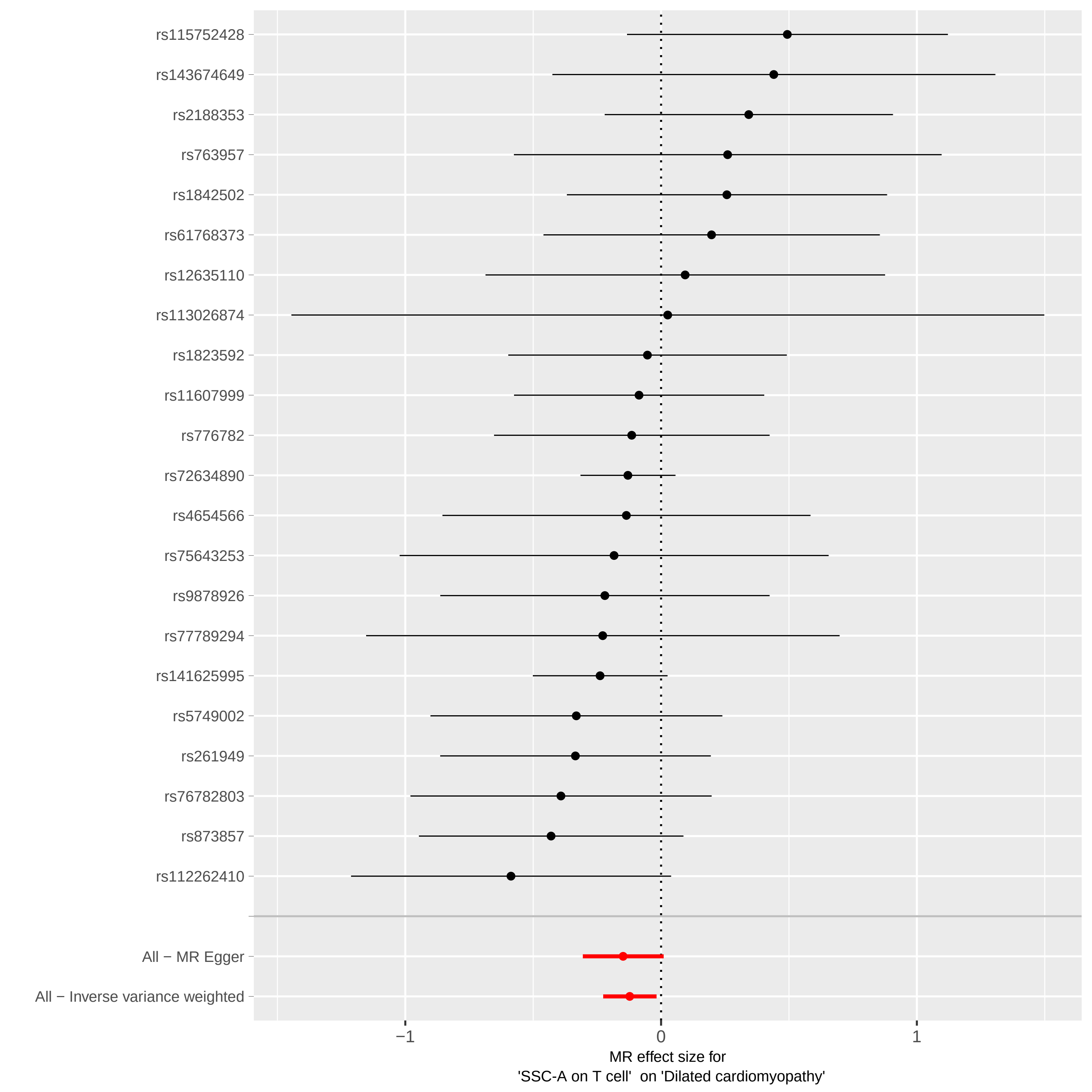
rs115752428
rs143674649
rs2188353
rs763957
rs1842502
rs61768373
rs12635110
rs113026874
rs1823592
rs11607999
rs776782
rs72634890
rs4654566
rs75643253
rs9878926
rs77789294
rs141625995
rs5749002
rs261949
rs76782803
rs873857
rs112262410
All − MR Egger
All − Inverse variance weighted
−1
0
1
MR effect size for
'SSC-A on T cell' on 'Dilated cardiomyopathy'

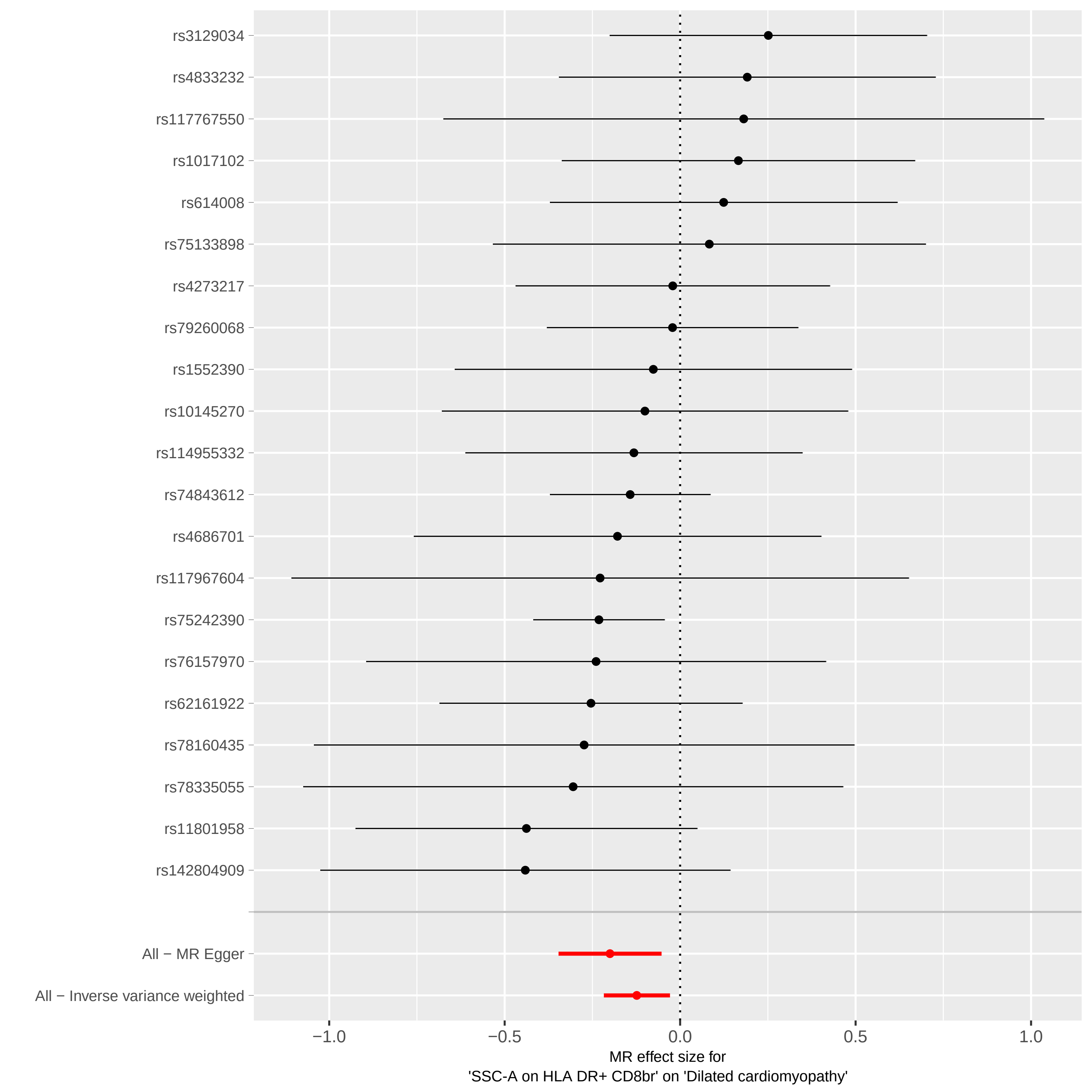
rs3129034
rs4833232
rs117767550
rs1017102
rs614008
rs75133898
rs4273217
rs79260068
rs1552390
rs10145270
rs114955332
rs74843612
rs4686701
rs117967604
rs75242390
rs76157970
rs62161922
rs78160435
rs78335055
rs11801958
rs142804909
All − MR Egger
All − Inverse variance weighted
−1.0
−0.5
0.0
0.5
1.0
MR effect size for
'SSC-A on HLA DR+ CD8br' on 'Dilated cardiomyopathy'

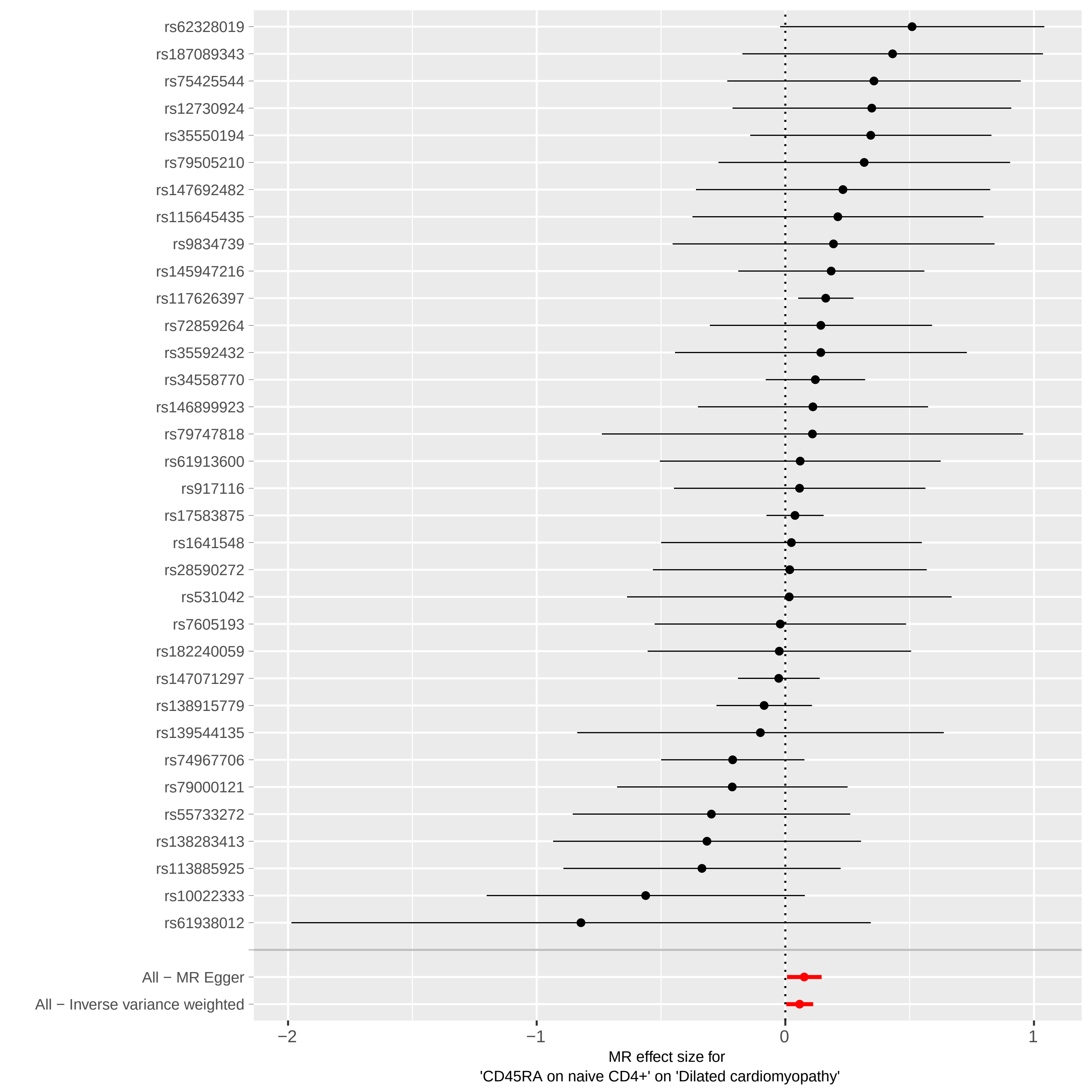
rs62328019
rs187089343
rs75425544
rs12730924
rs35550194
rs79505210
rs147692482
rs115645435
rs9834739
rs145947216
rs117626397
rs72859264
rs35592432
rs34558770
rs146899923
rs79747818
rs61913600
rs917116
rs17583875
rs1641548
rs28590272
rs531042
rs7605193
rs182240059
rs147071297
rs138915779
rs139544135
rs74967706
rs79000121
rs55733272
rs138283413
rs113885925
rs10022333
rs61938012
All − MR Egger
All − Inverse variance weighted
−2
−1
0
1
MR effect size for
'CD45RA on naive CD4+' on 'Dilated cardiomyopathy'

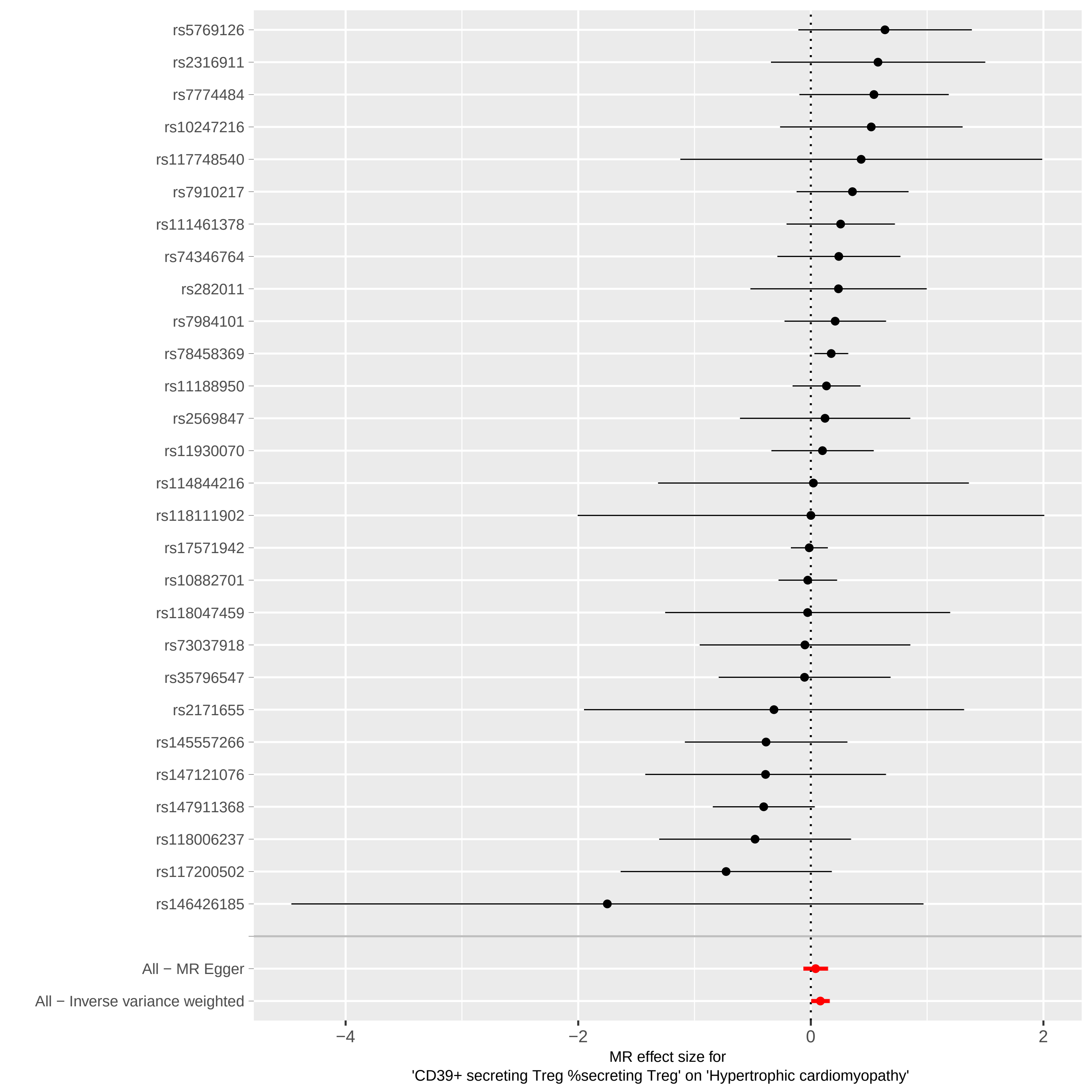
rs5769126
rs2316911
rs7774484
rs10247216
rs117748540
rs7910217
rs111461378
rs74346764
rs282011
rs7984101
rs78458369
rs11188950
rs2569847
rs11930070
rs114844216
rs118111902
rs17571942
rs10882701
rs118047459
rs73037918
rs35796547
rs2171655
rs145557266
rs147121076
rs147911368
rs118006237
rs117200502
rs146426185
All − MR Egger
All − Inverse variance weighted
−4
−2
0
2
MR effect size for
'CD39+ secreting Treg %secreting Treg' on 'Hypertrophic cardiomyopathy'

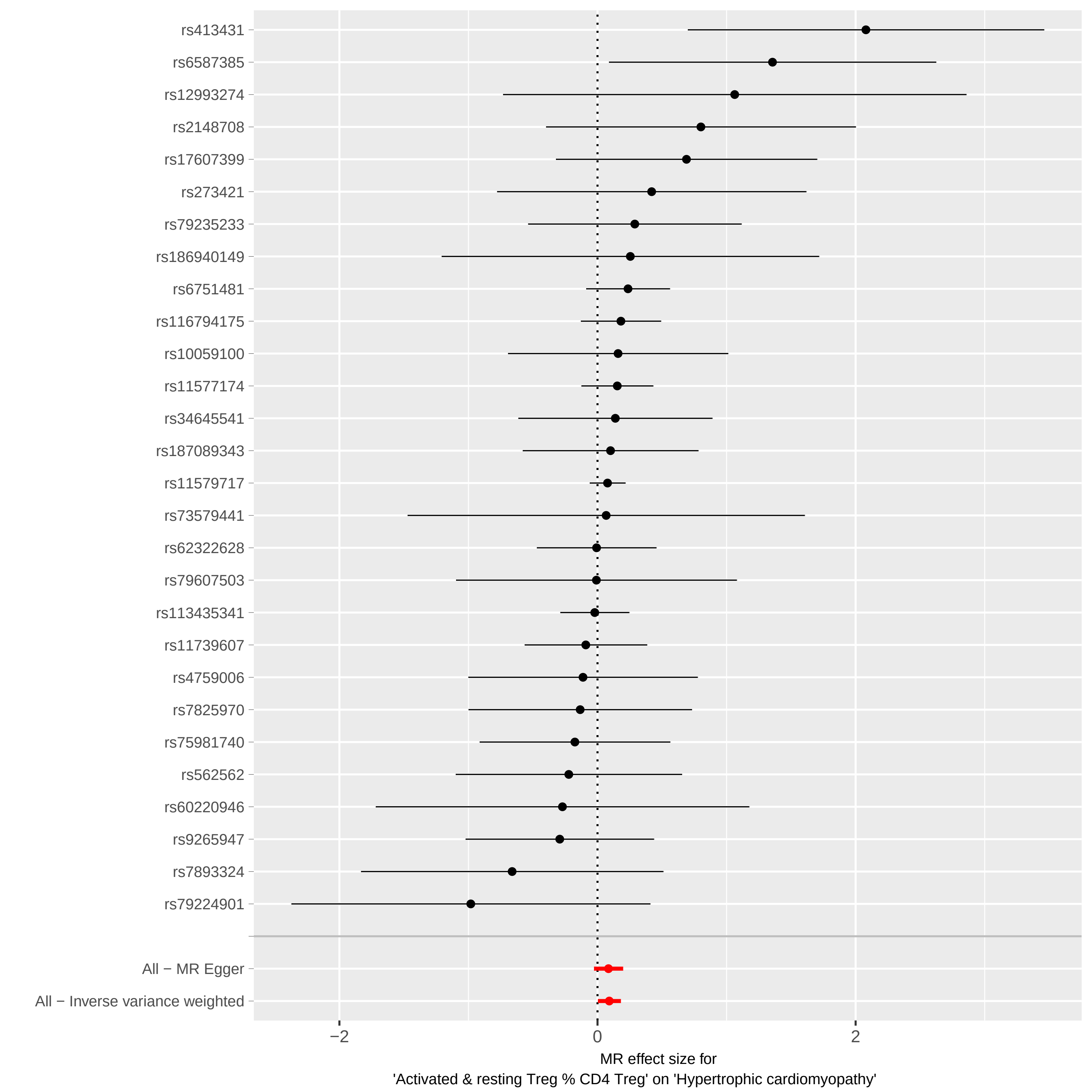
rs413431
rs6587385
rs12993274
rs2148708
rs17607399
rs273421
rs79235233
rs186940149
rs6751481
rs116794175
rs10059100
rs11577174
rs34645541
rs187089343
rs11579717
rs73579441
rs62322628
rs79607503
rs113435341
rs11739607
rs4759006
rs7825970
rs75981740
rs562562
rs60220946
rs9265947
rs7893324
rs79224901
All − MR Egger
All − Inverse variance weighted
−2
0
2
MR effect size for
'Activated & resting Treg % CD4 Treg' on 'Hypertrophic cardiomyopathy'

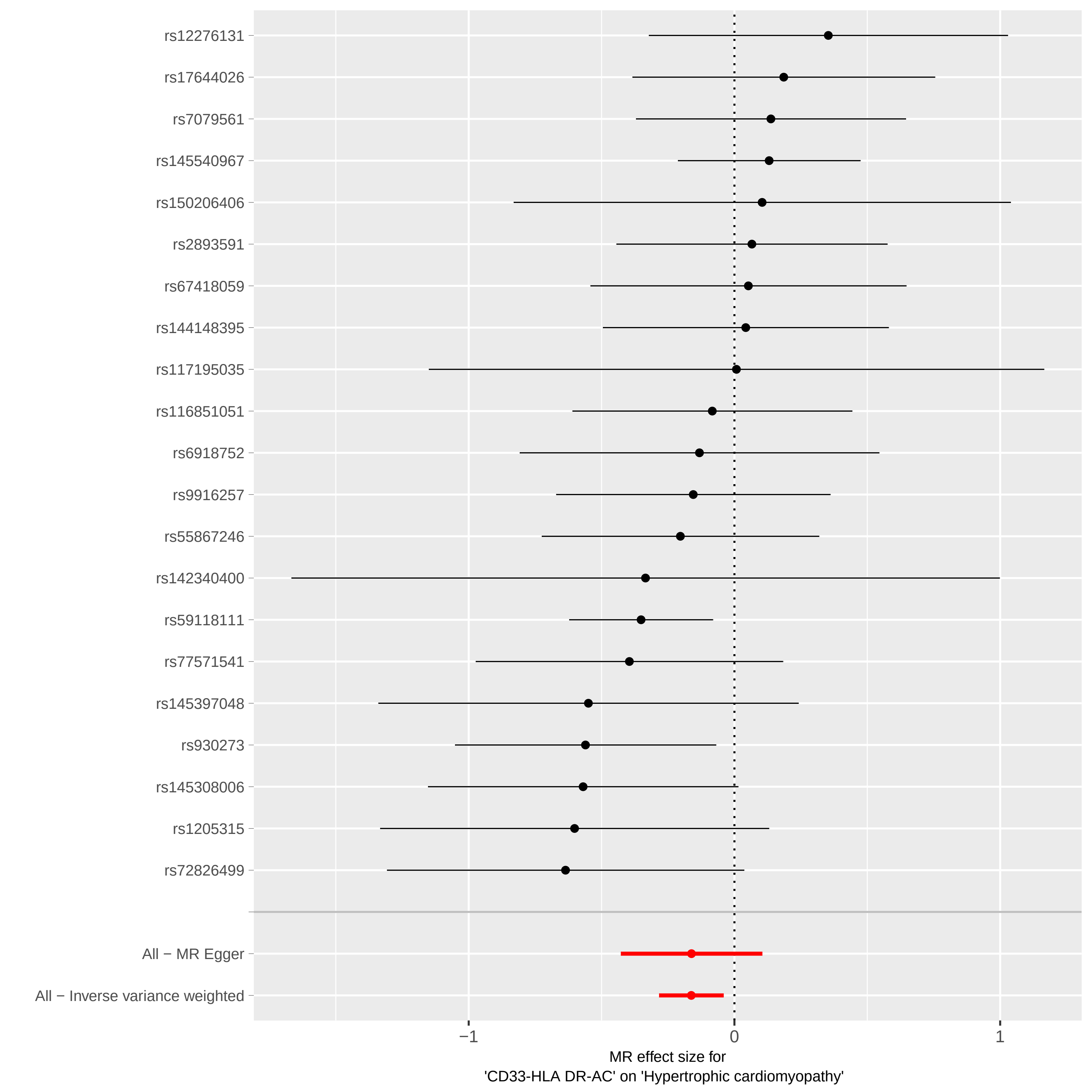
rs12276131
rs17644026
rs7079561
rs145540967
rs150206406
rs2893591
rs67418059
rs144148395
rs117195035
rs116851051
rs6918752
rs9916257
rs55867246
rs142340400
rs59118111
rs77571541
rs145397048
rs930273
rs145308006
rs1205315
rs72826499
All − MR Egger
All − Inverse variance weighted
−1
0
1
MR effect size for
'CD33-HLA DR-AC' on 'Hypertrophic cardiomyopathy'

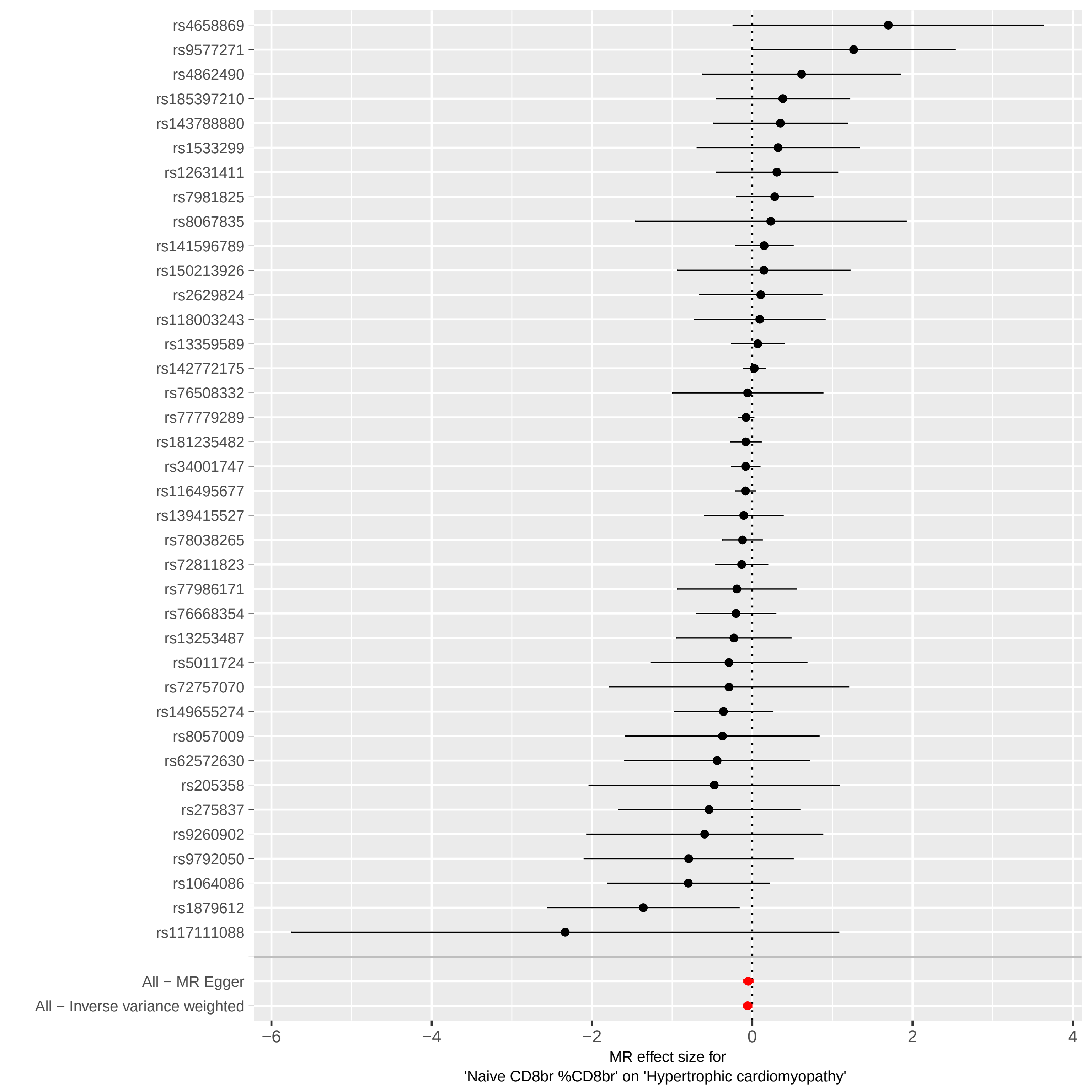
rs4658869
rs9577271
rs4862490
rs185397210
rs143788880
rs1533299
rs12631411
rs7981825
rs8067835
rs141596789
rs150213926
rs2629824
rs118003243
rs13359589
rs142772175
rs76508332
rs77779289
rs181235482
rs34001747
rs116495677
rs139415527
rs78038265
rs72811823
rs77986171
rs76668354
rs13253487
rs5011724
rs72757070
rs149655274
rs8057009
rs62572630
rs205358
rs275837
rs9260902
rs9792050
rs1064086
rs1879612
rs117111088
All − MR Egger
All − Inverse variance weighted
−6
−4
−2
0
2
4
MR effect size for
'Naive CD8br %CD8br' on 'Hypertrophic cardiomyopathy'

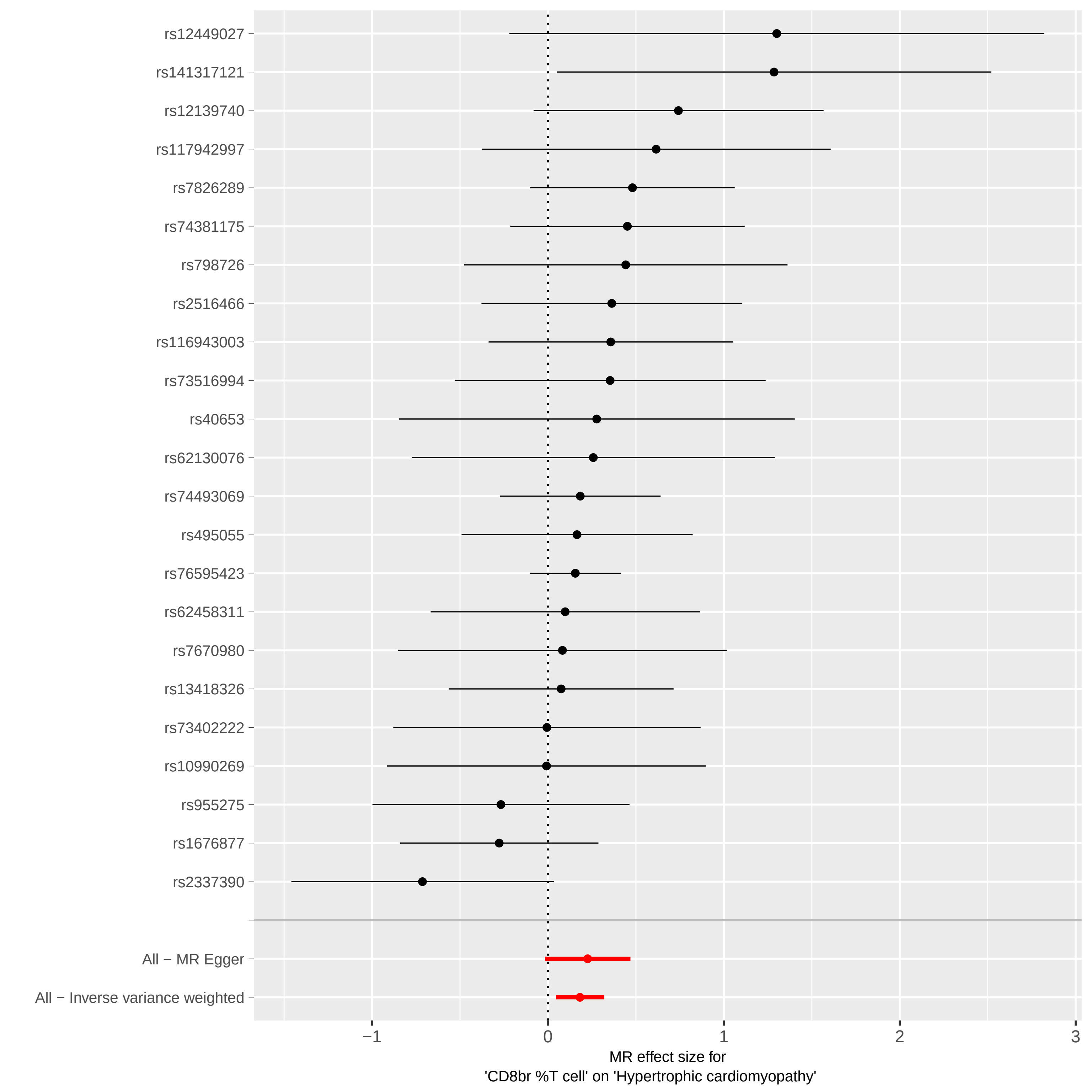

rs12449027
rs141317121
rs12139740
rs117942997
rs7826289
rs74381175
rs798726
rs2516466
rs116943003
rs73516994
rs40653
rs62130076
rs74493069
rs495055
rs76595423
rs62458311
rs7670980
rs13418326
rs73402222
rs10990269
rs955275
rs1676877
rs2337390
All − MR Egger
All − Inverse variance weighted
−1
0
1
2
3
MR effect size for
'CD8br %T cell' on 'Hypertrophic cardiomyopathy'

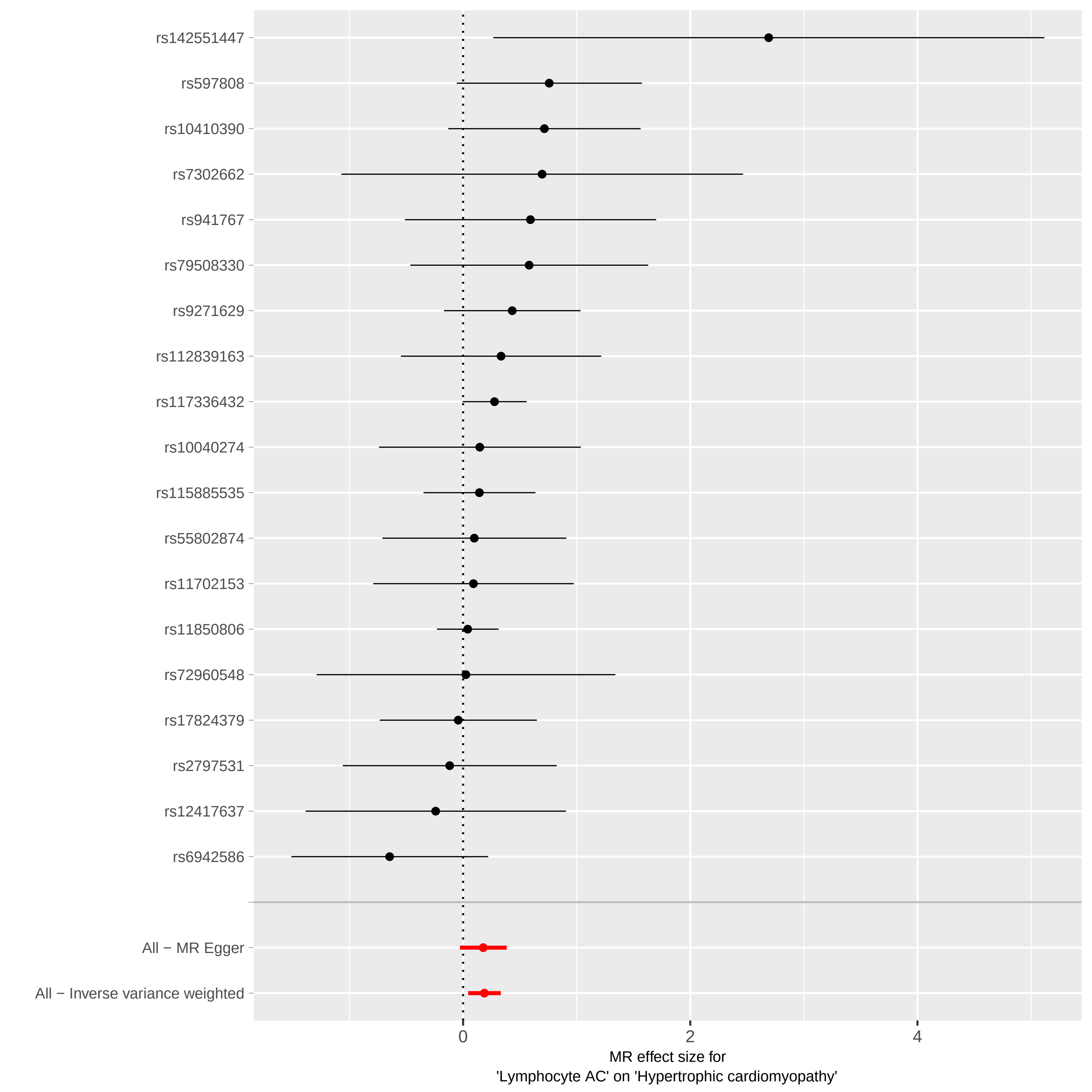
rs142551447
rs597808
rs10410390
rs7302662
rs941767
rs79508330
rs9271629
rs112839163
rs117336432
rs10040274
rs115885535
rs55802874
rs11702153
rs11850806
rs72960548
rs17824379
rs2797531
rs12417637
rs6942586
All − MR Egger
All − Inverse variance weighted
0
2
4
MR effect size for
'Lymphocyte AC' on 'Hypertrophic cardiomyopathy'

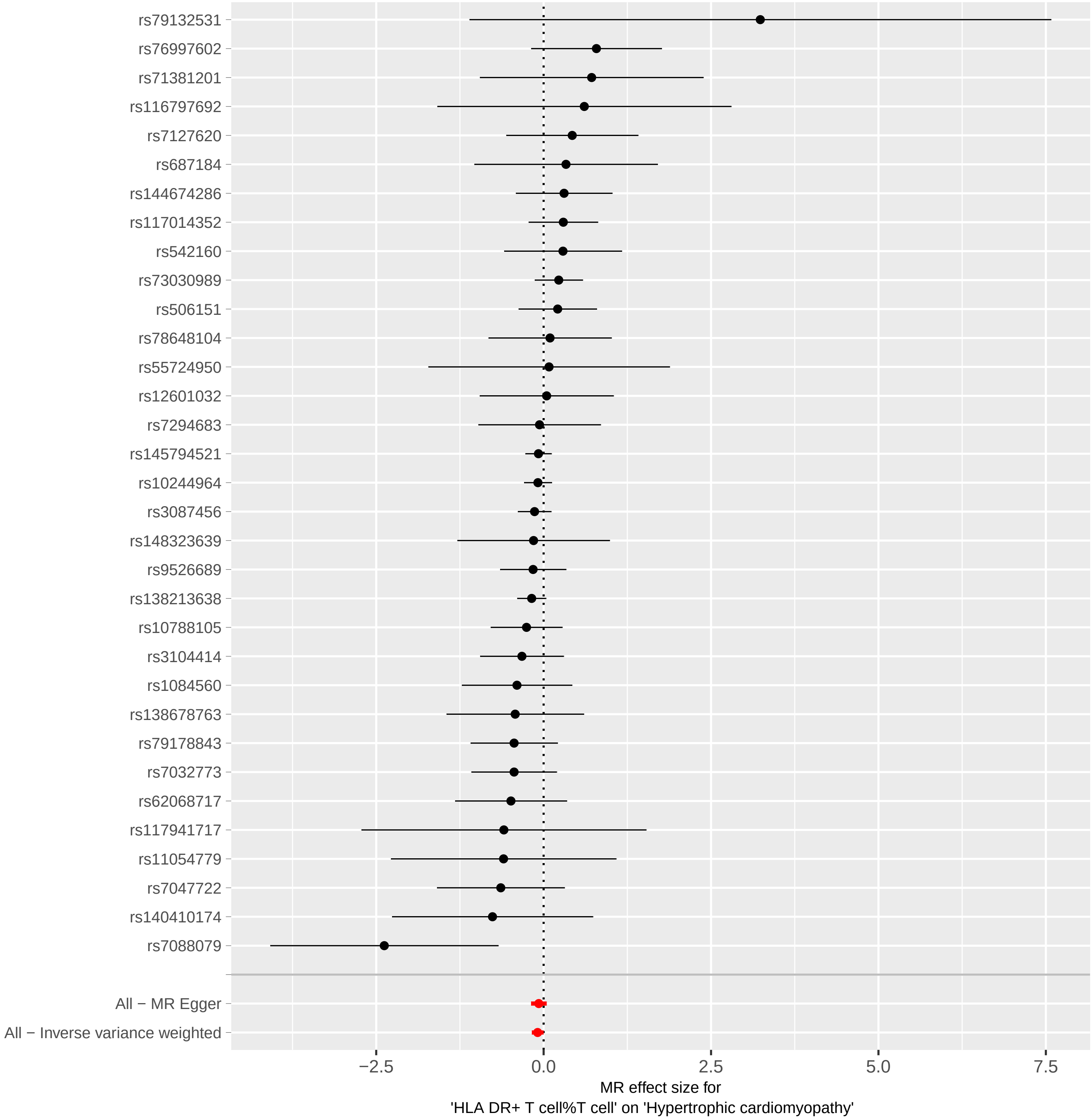
rs79132531
rs76997602
rs71381201
rs116797692
rs7127620
rs687184
rs144674286
rs117014352
rs542160
rs73030989
rs506151
rs78648104
rs55724950
rs12601032
rs7294683
rs145794521
rs10244964
rs3087456
rs148323639
rs9526689
rs138213638
rs10788105
rs3104414
rs1084560
rs138678763
rs79178843
rs7032773
rs62068717
rs117941717
rs11054779
rs7047722
rs140410174
rs7088079
All − MR Egger
All − Inverse variance weighted
−2.5
0.0
2.5
5.0
7.5
MR effect size for
'HLA DR+ T cell%T cell' on 'Hypertrophic cardiomyopathy'

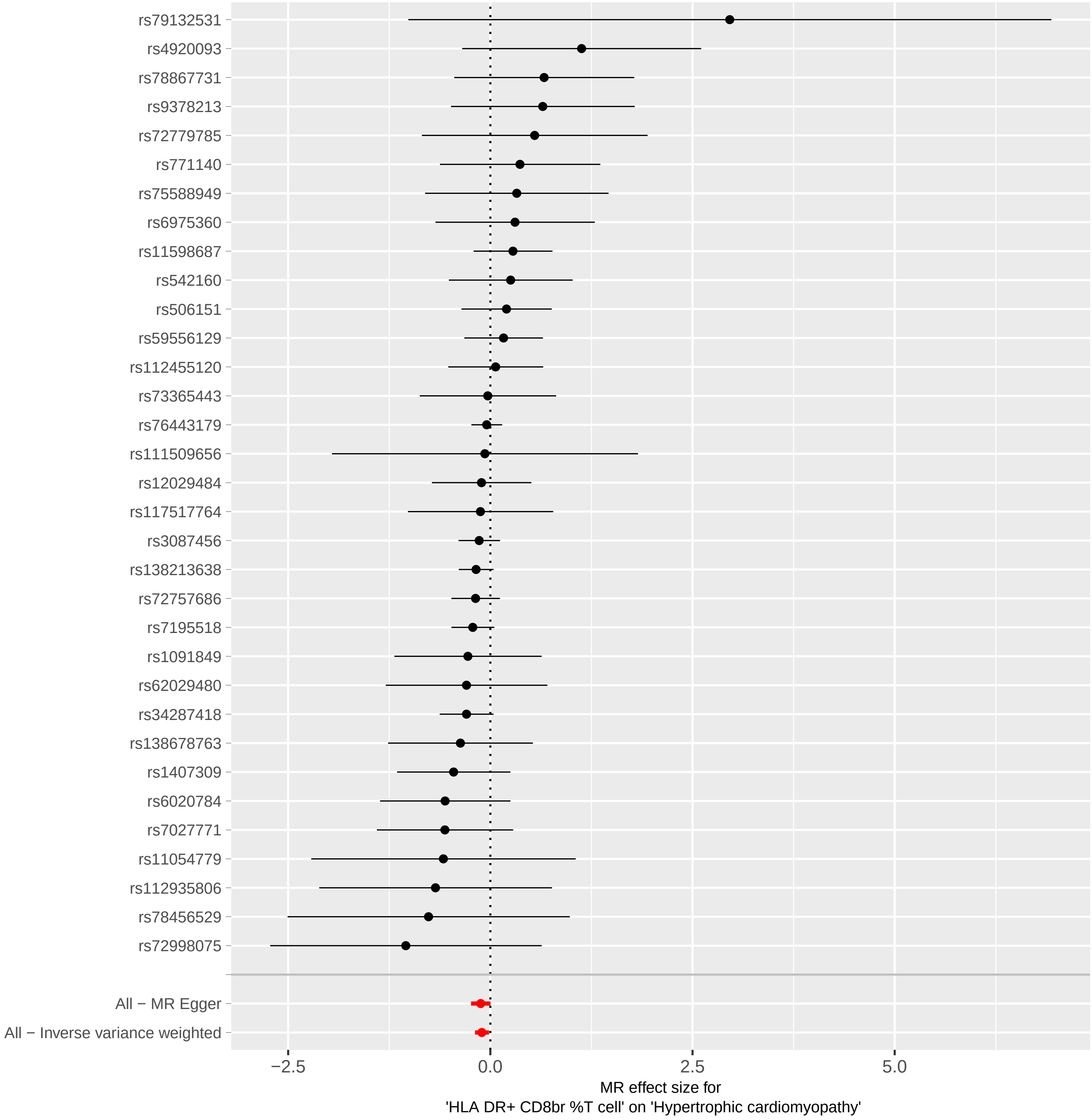
rs79132531
rs4920093
rs78867731
rs9378213
rs72779785
rs771140
rs75588949
rs6975360
rs11598687
rs542160
rs506151
rs59556129
rs112455120
rs73365443
rs76443179
rs111509656
rs12029484
rs117517764
rs3087456
rs138213638
rs72757686
rs7195518
rs1091849
rs62029480
rs34287418
rs138678763
rs1407309
rs6020784
rs7027771
rs11054779
rs112935806
rs78456529
rs72998075
All − MR Egger
All − Inverse variance weighted
−2.5
0.0
2.5
5.0
MR effect size for
'HLA DR+ CD8br %T cell' on 'Hypertrophic cardiomyopathy'

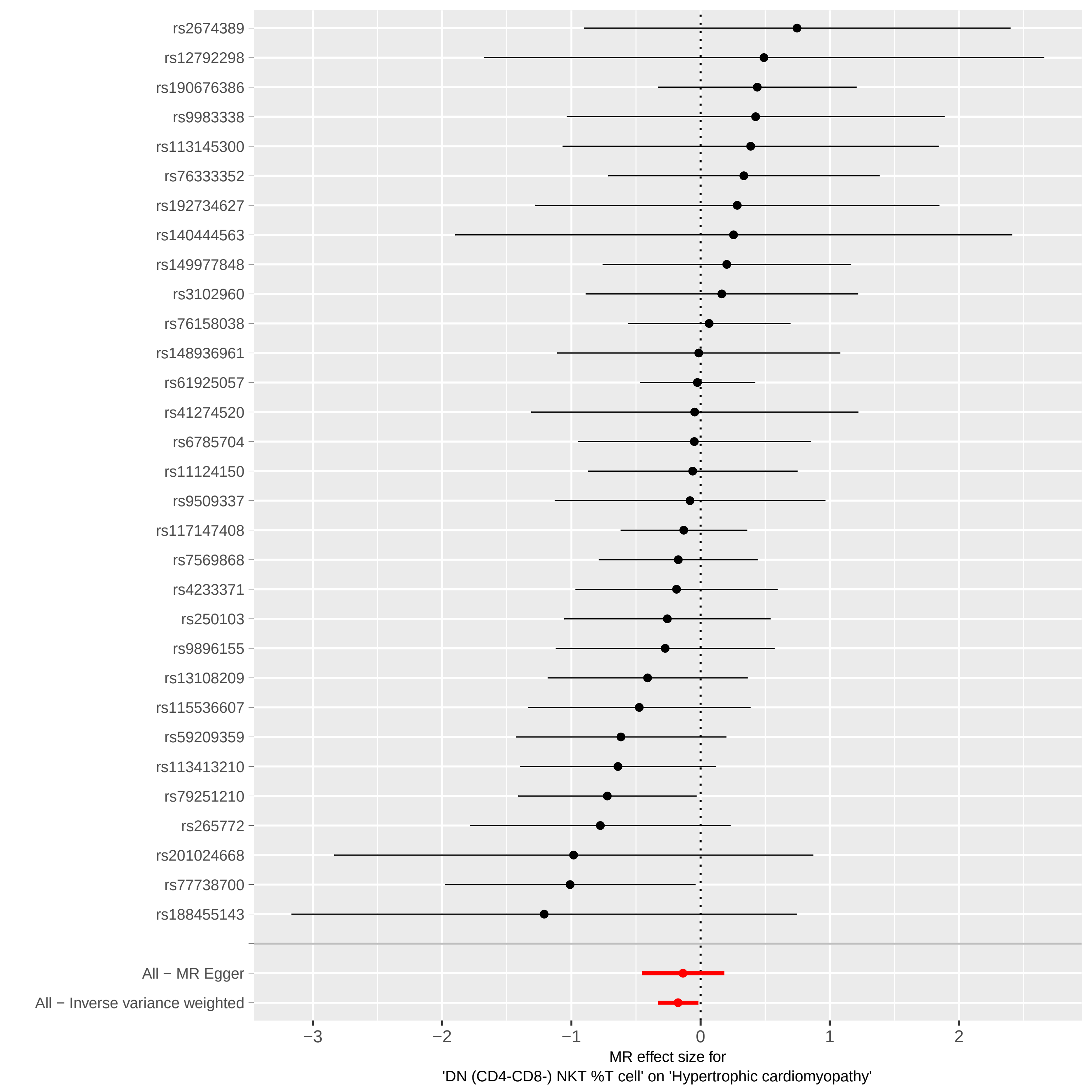

rs2674389
rs12792298
rs190676386
rs9983338
rs113145300
rs76333352
rs192734627
rs140444563
rs149977848
rs3102960
rs76158038
rs148936961
rs61925057
rs41274520
rs6785704
rs11124150
rs9509337
rs117147408
rs7569868
rs4233371
rs250103
rs9896155
rs13108209
rs115536607
rs59209359
rs113413210
rs79251210
rs265772
rs201024668
rs77738700
rs188455143
All − MR Egger
All − Inverse variance weighted
−3
−2
−1
0
1
2
MR effect size for
'DN (CD4-CD8-) NKT %T cell' on 'Hypertrophic cardiomyopathy'

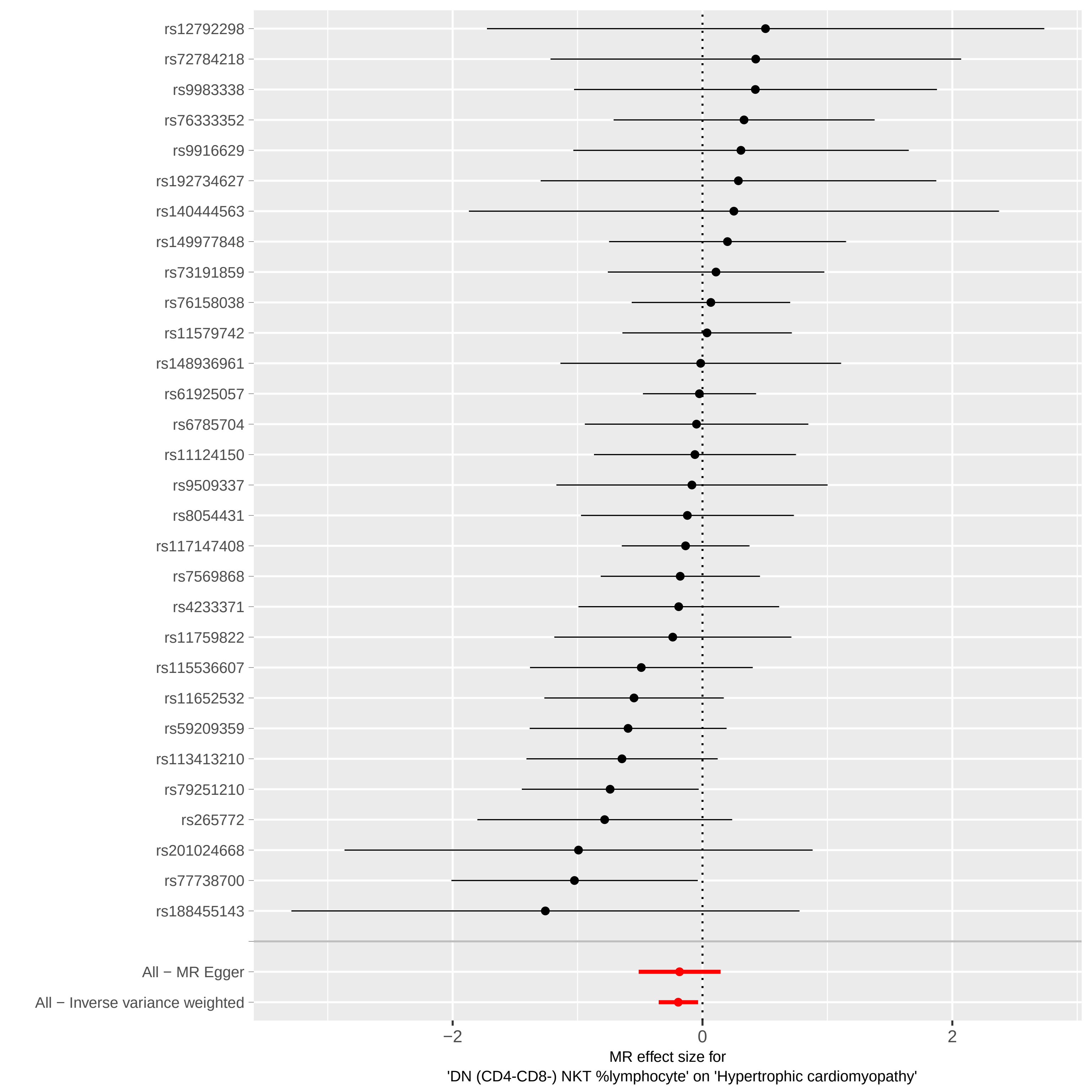
rs12792298
rs72784218
rs9983338
rs76333352
rs9916629
rs192734627
rs140444563
rs149977848
rs73191859
rs76158038
rs11579742
rs148936961
rs61925057
rs6785704
rs11124150
rs9509337
rs8054431
rs117147408
rs7569868
rs4233371
rs11759822
rs115536607
rs11652532
rs59209359
rs113413210
rs79251210
rs265772
rs201024668
rs77738700
rs188455143
All − MR Egger
All − Inverse variance weighted
−2
0
2
MR effect size for
'DN (CD4-CD8-) NKT %lymphocyte' on 'Hypertrophic cardiomyopathy'

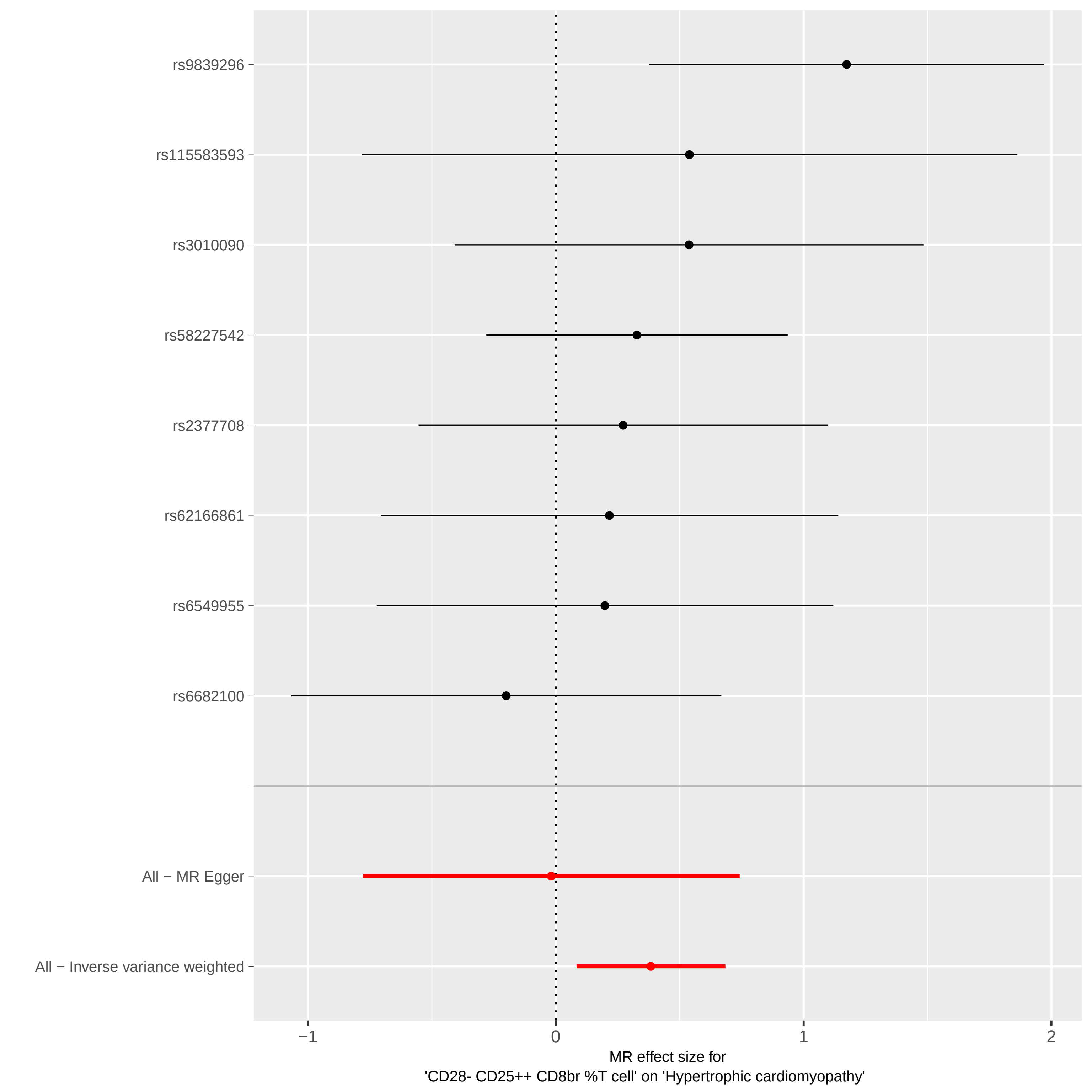
rs9839296
rs115583593
rs3010090
rs58227542
rs2377708
rs62166861
rs6549955
rs6682100
All − MR Egger
All − Inverse variance weighted
−1
0
1
2
MR effect size for
'CD28- CD25++ CD8br %T cell' on 'Hypertrophic cardiomyopathy'

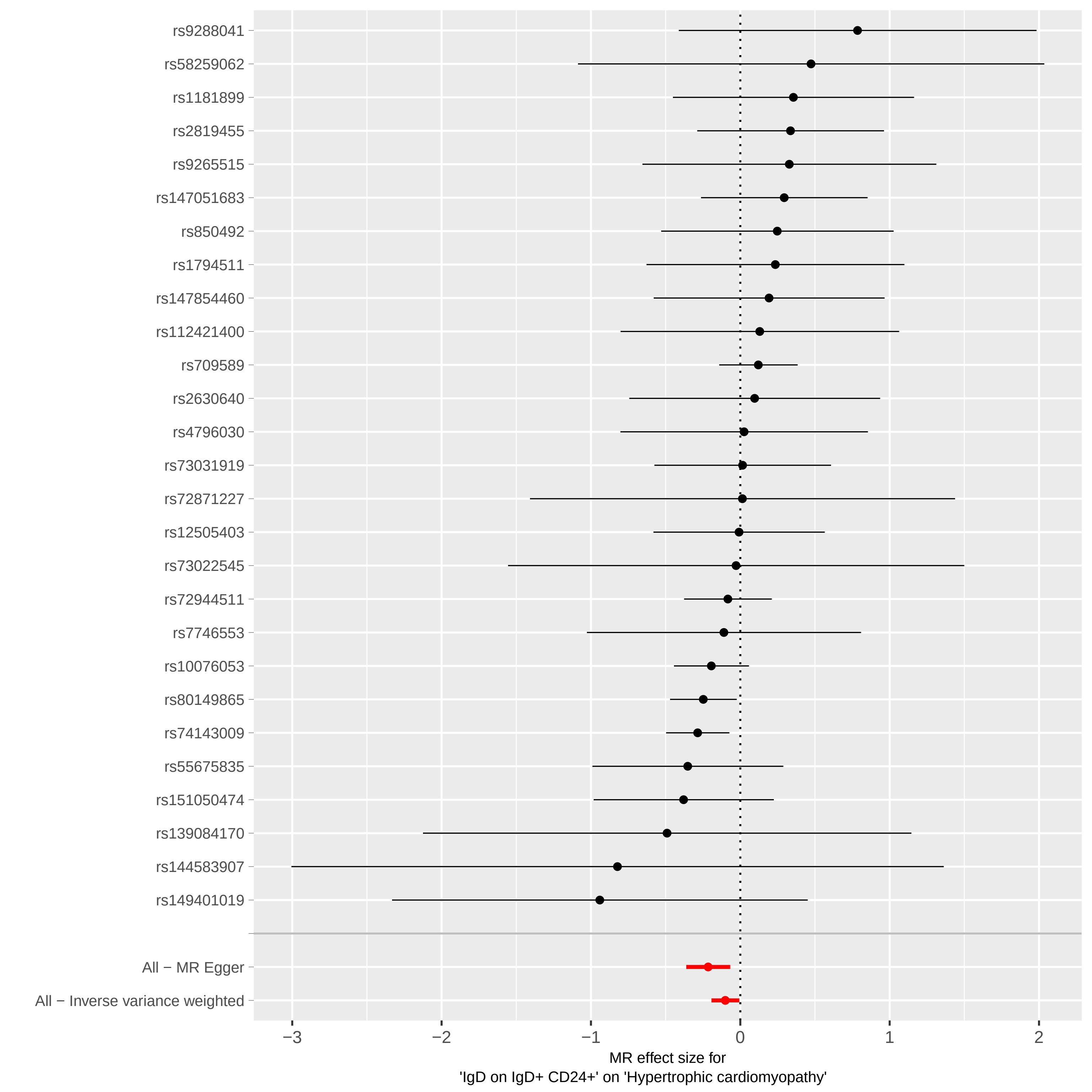

rs9288041
rs58259062
rs1181899
rs2819455
rs9265515
rs147051683
rs850492
rs1794511
rs147854460
rs112421400
rs709589
rs2630640
rs4796030
rs73031919
rs72871227
rs12505403
rs73022545
rs72944511
rs7746553
rs10076053
rs80149865
rs74143009
rs55675835
rs151050474
rs139084170
rs144583907
rs149401019
All − MR Egger
All − Inverse variance weighted
−3
−2
−1
0
1
2
MR effect size for
'IgD on IgD+ CD24+' on 'Hypertrophic cardiomyopathy'

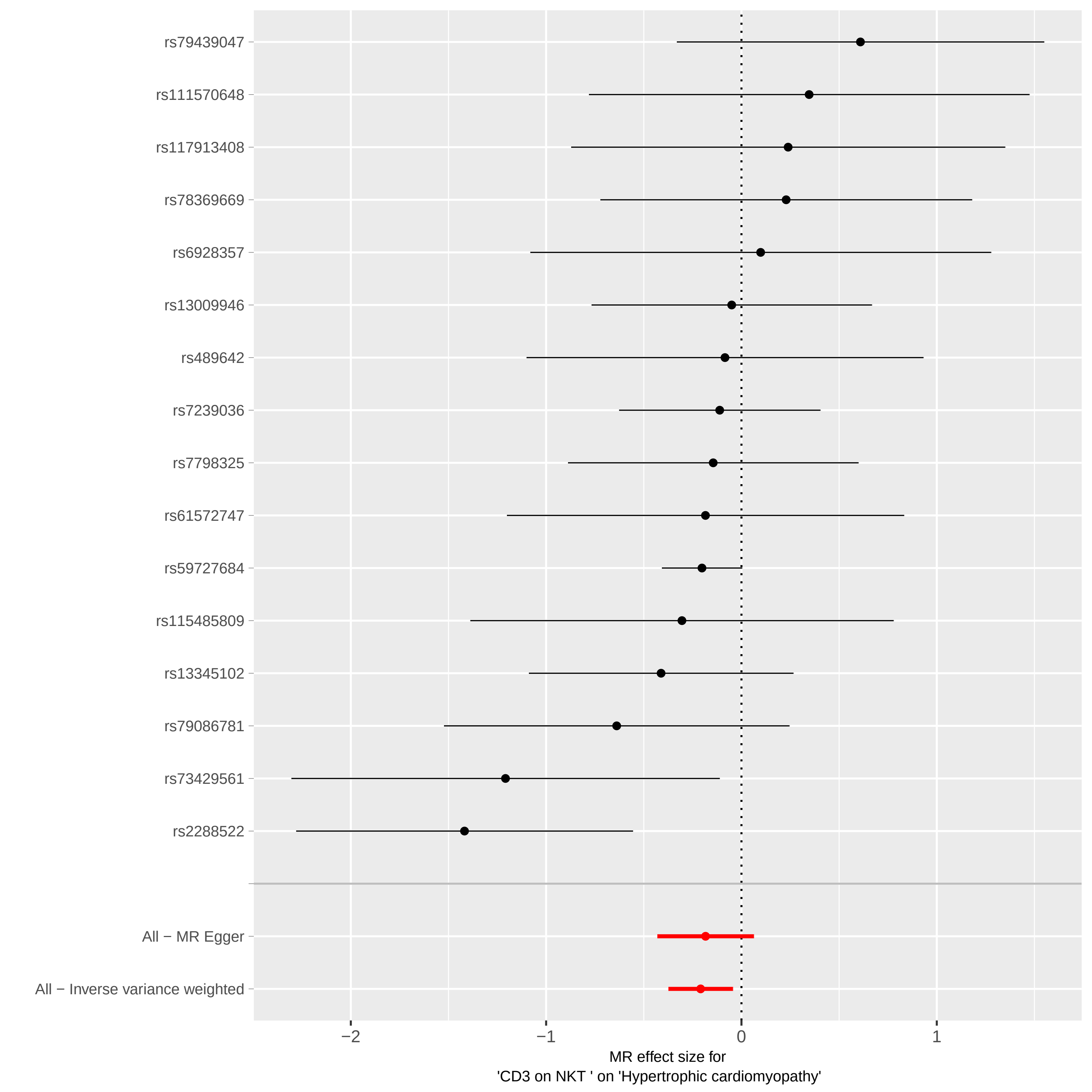

rs79439047
rs111570648
rs117913408
rs78369669
rs6928357
rs13009946
rs489642
rs7239036
rs7798325
rs61572747
rs59727684
rs115485809
rs13345102
rs79086781
rs73429561
rs2288522
All − MR Egger
All − Inverse variance weighted
−2
−1
0
1
MR effect size for
'CD3 on NKT ' on 'Hypertrophic cardiomyopathy'

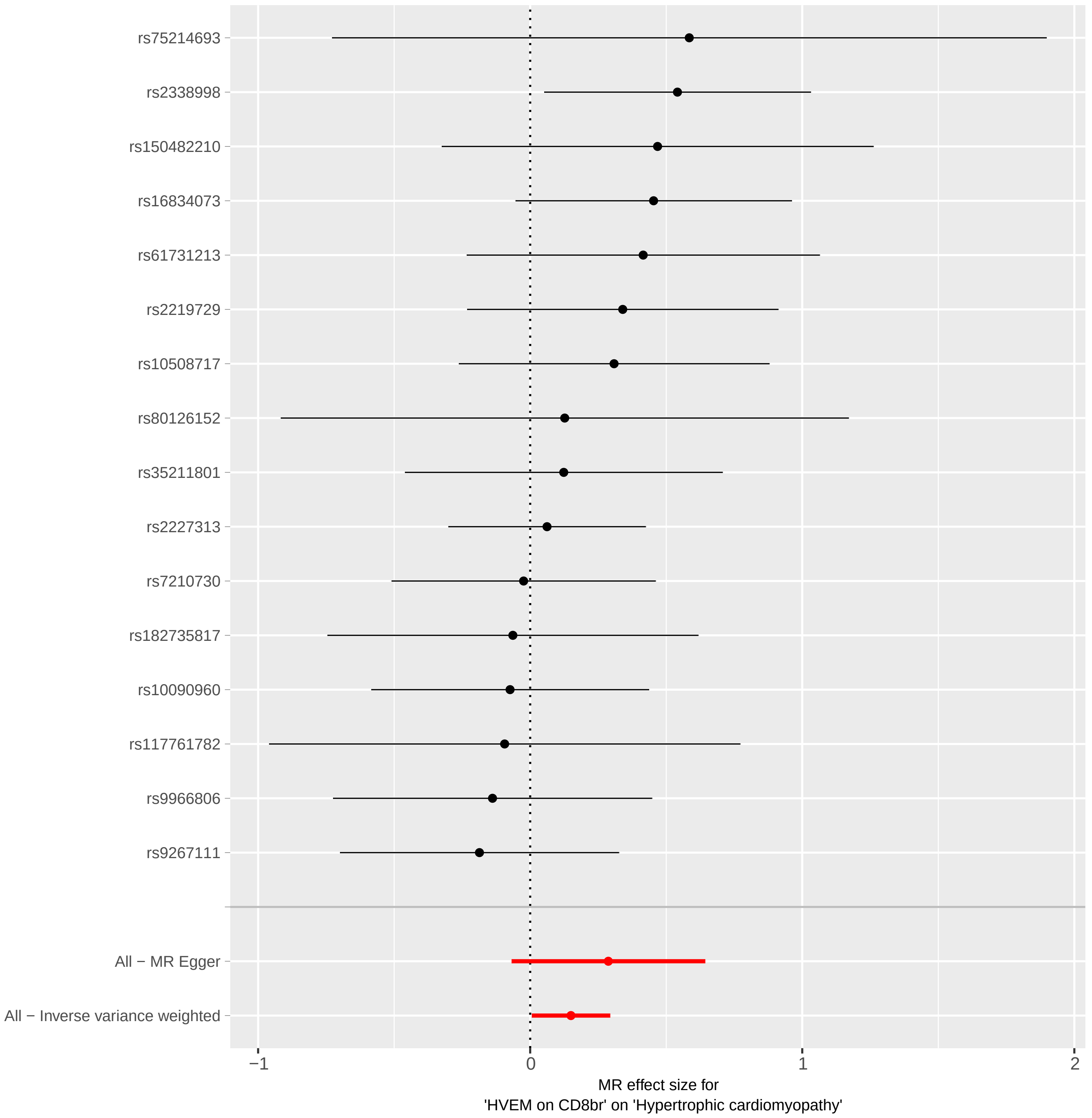
rs75214693
rs2338998
rs150482210
rs16834073
rs61731213
rs2219729
rs10508717
rs80126152
rs35211801
rs2227313
rs7210730
rs182735817
rs10090960
rs117761782
rs9966806
rs9267111
All − MR Egger
All − Inverse variance weighted
−1
0
1
2
MR effect size for
'HVEM on CD8br' on 'Hypertrophic cardiomyopathy'

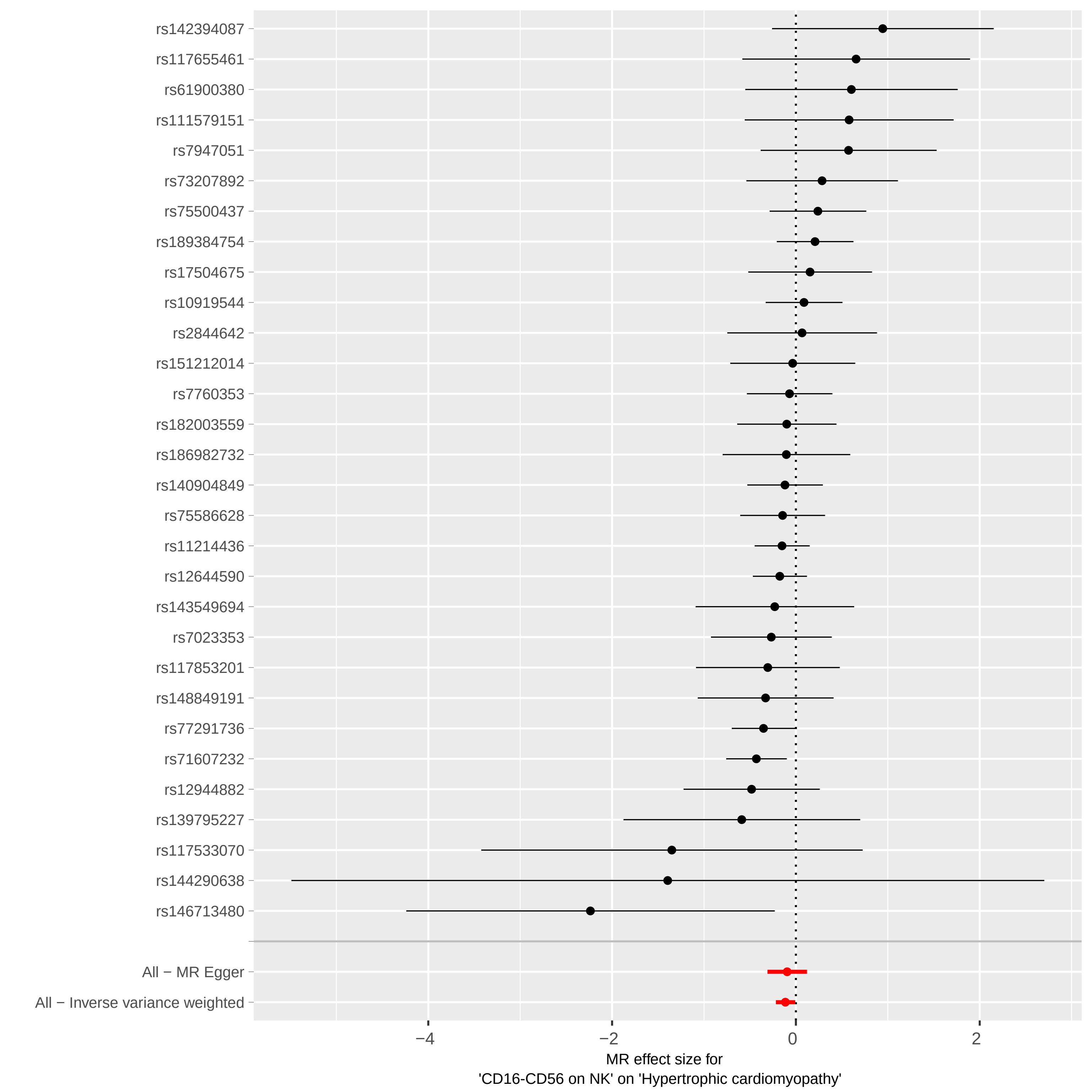
rs142394087
rs117655461
rs61900380
rs111579151
rs7947051
rs73207892
rs75500437
rs189384754
rs17504675
rs10919544
rs2844642
rs151212014
rs7760353
rs182003559
rs186982732
rs140904849
rs75586628
rs11214436
rs12644590
rs143549694
rs7023353
rs117853201
rs148849191
rs77291736
rs71607232
rs12944882
rs139795227
rs117533070
rs144290638
rs146713480
All − MR Egger
All − Inverse variance weighted
−4
−2
0
2
MR effect size for
'CD16-CD56 on NK' on 'Hypertrophic cardiomyopathy'

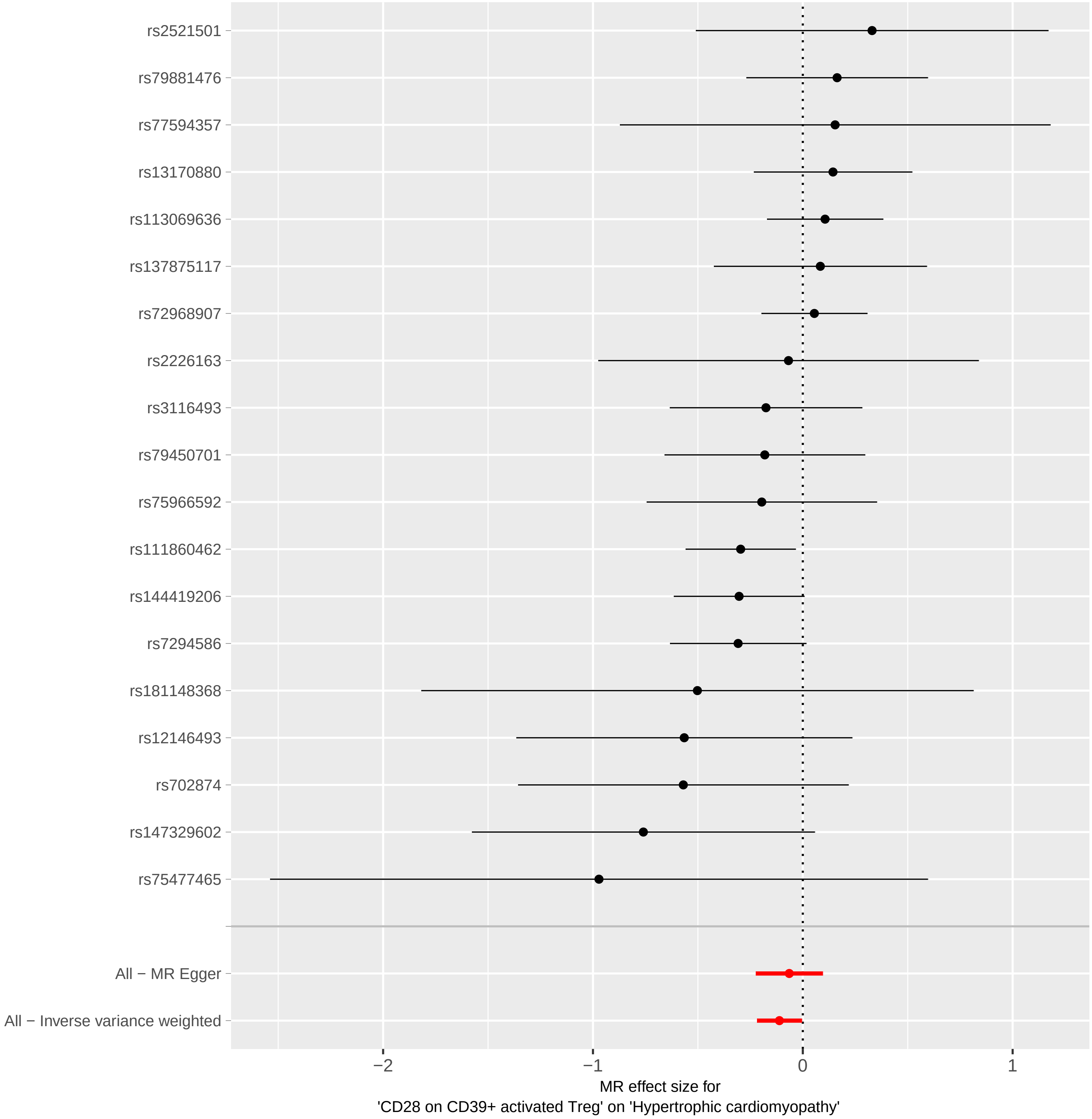

rs2521501
rs79881476
rs77594357
rs13170880
rs113069636
rs137875117
rs72968907
rs2226163
rs3116493
rs79450701
rs75966592
rs111860462
rs144419206
rs7294586
rs181148368
rs12146493
rs702874
rs147329602
rs75477465
All − MR Egger
All − Inverse variance weighted
−2
−1
0
1
MR effect size for
'CD28 on CD39+ activated Treg' on 'Hypertrophic cardiomyopathy'

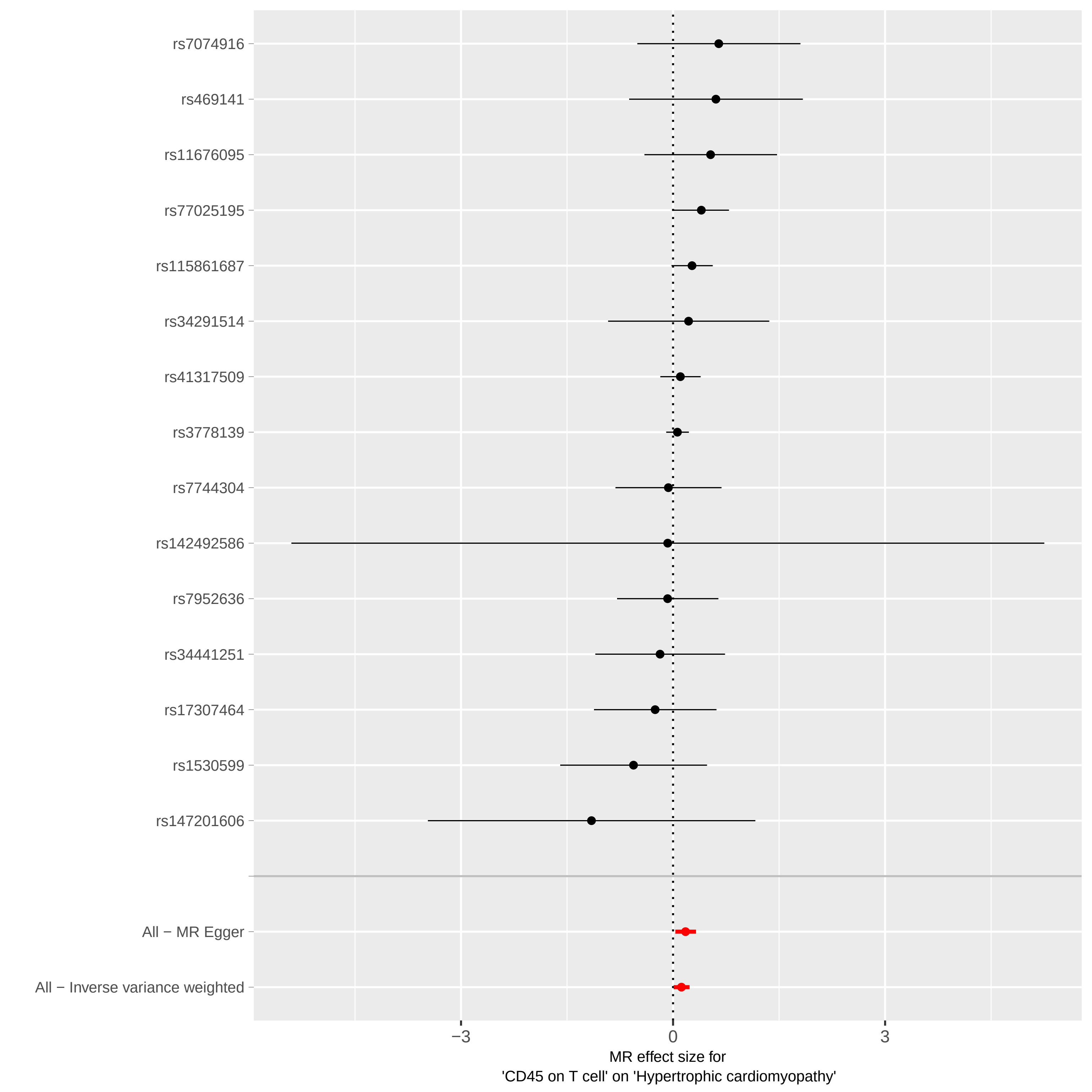
rs7074916
rs469141
rs11676095
rs77025195
rs115861687
rs34291514
rs41317509
rs3778139
rs7744304
rs142492586
rs7952636
rs34441251
rs17307464
rs1530599
rs147201606
All − MR Egger
All − Inverse variance weighted
−3
0
3
MR effect size for
'CD45 on T cell' on 'Hypertrophic cardiomyopathy'

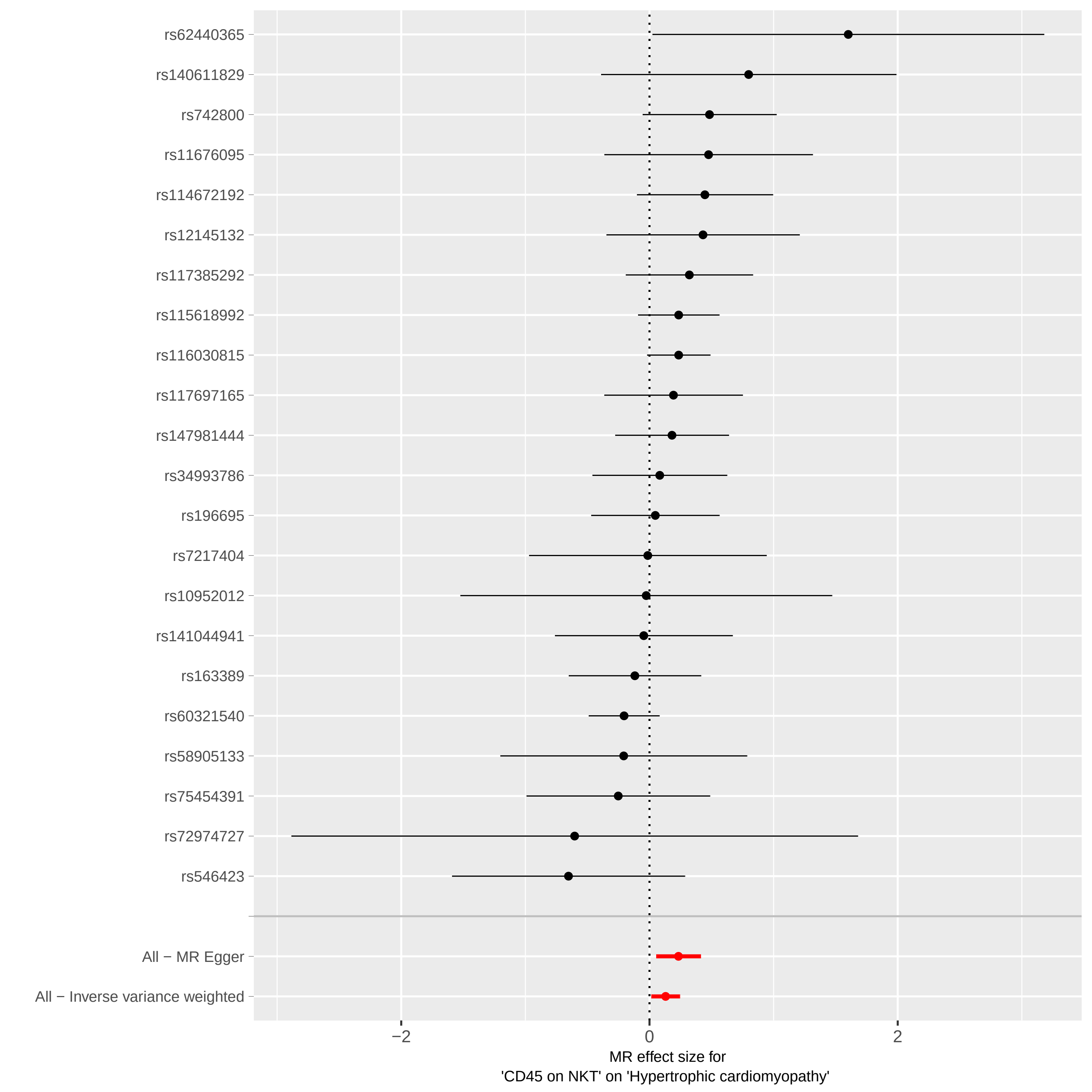

rs62440365
rs140611829
rs742800
rs11676095
rs114672192
rs12145132
rs117385292
rs115618992
rs116030815
rs117697165
rs147981444
rs34993786
rs196695
rs7217404
rs10952012
rs141044941
rs163389
rs60321540
rs58905133
rs75454391
rs72974727
rs546423
All − MR Egger
All − Inverse variance weighted
−2
0
2
MR effect size for
'CD45 on NKT' on 'Hypertrophic cardiomyopathy'

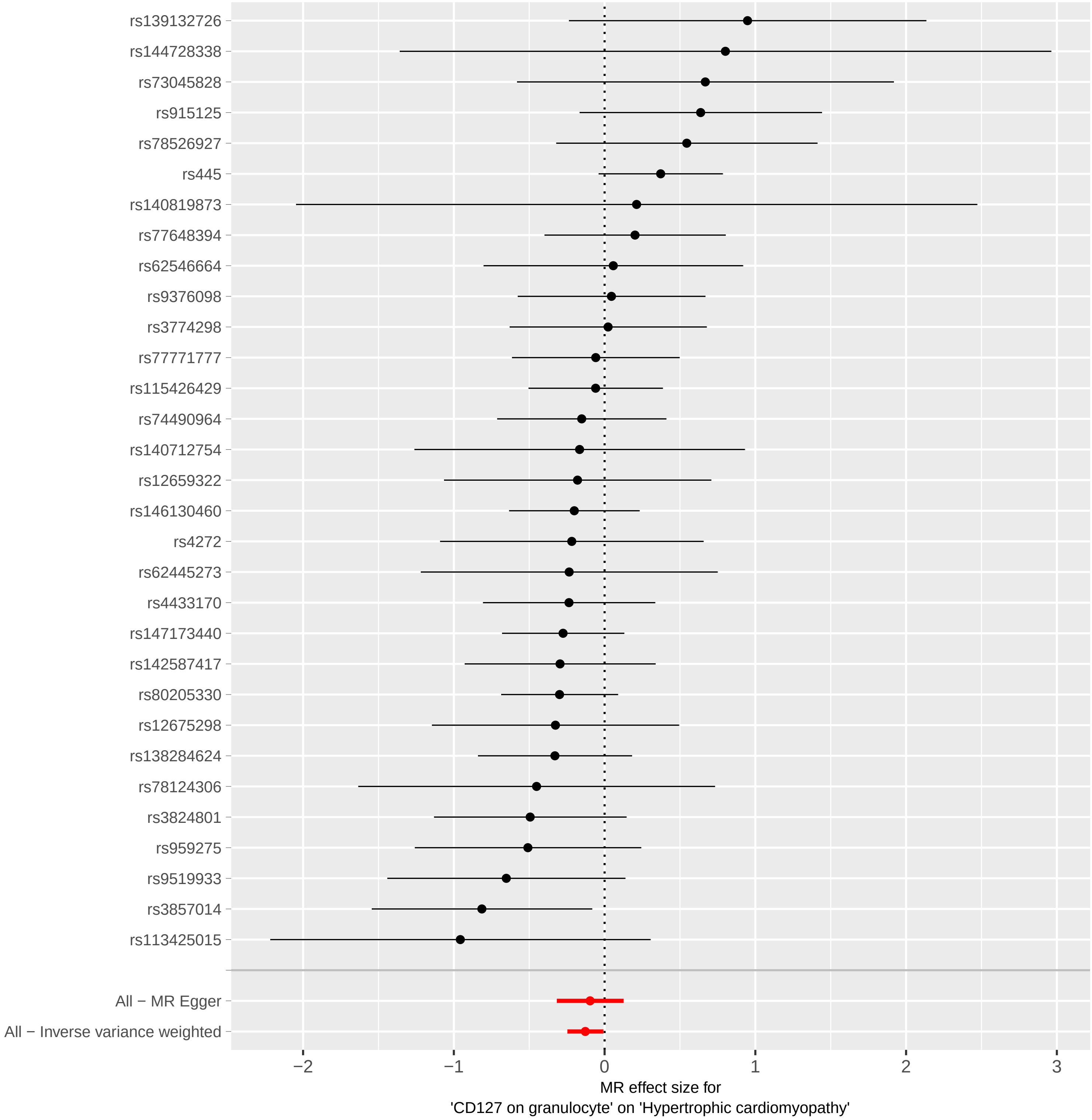
rs139132726
rs144728338
rs73045828
rs915125
rs78526927
rs445
rs140819873
rs77648394
rs62546664
rs9376098
rs3774298
rs77771777
rs115426429
rs74490964
rs140712754
rs12659322
rs146130460
rs4272
rs62445273
rs4433170
rs147173440
rs142587417
rs80205330
rs12675298
rs138284624
rs78124306
rs3824801
rs959275
rs9519933
rs3857014
rs113425015
All − MR Egger
All − Inverse variance weighted
−2
−1
0
1
2
3
MR effect size for
'CD127 on granulocyte' on 'Hypertrophic cardiomyopathy'

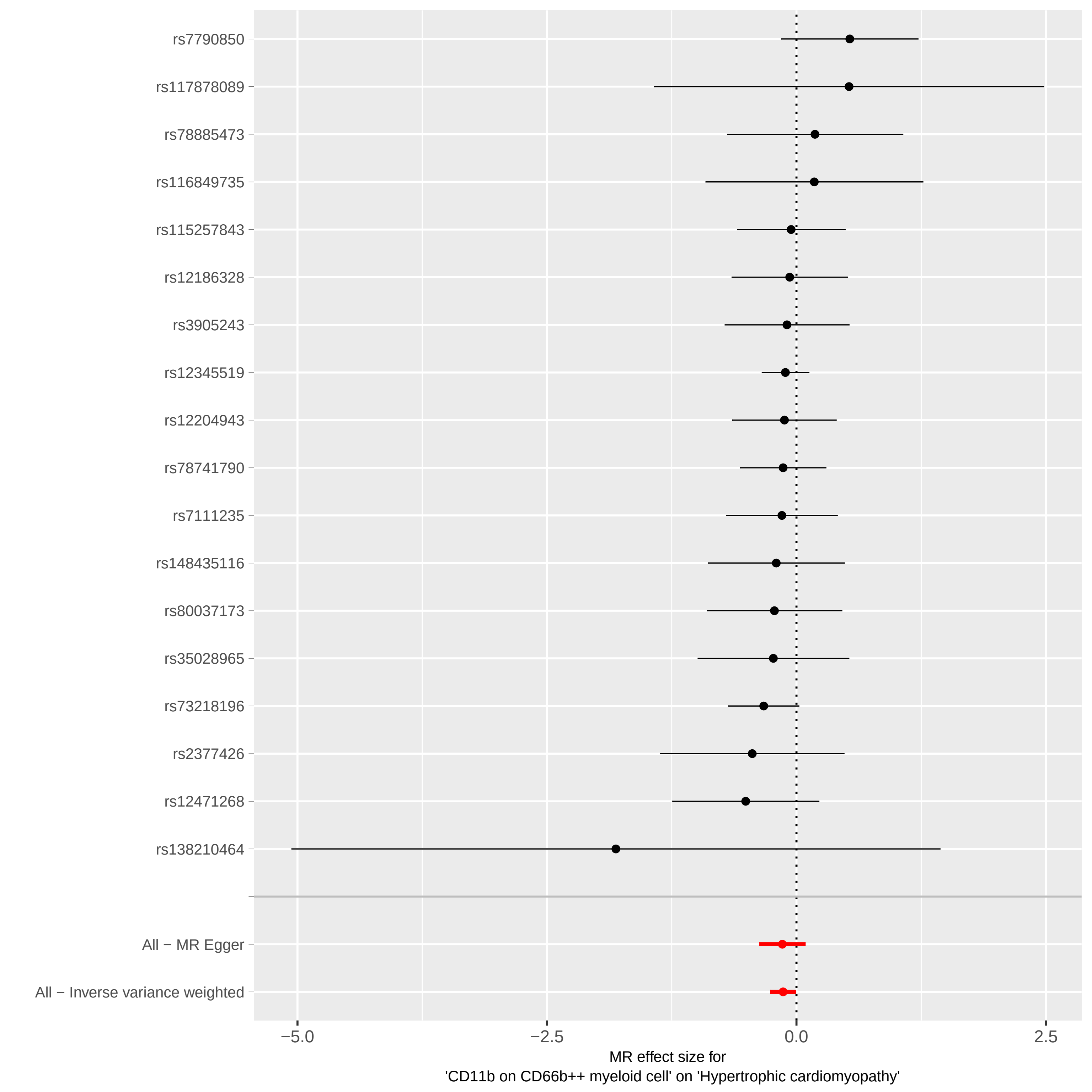
rs7790850
rs117878089
rs78885473
rs116849735
rs115257843
rs12186328
rs3905243
rs12345519
rs12204943
rs78741790
rs7111235
rs148435116
rs80037173
rs35028965
rs73218196
rs2377426
rs12471268
rs138210464
All − MR Egger
All − Inverse variance weighted
−5.0
−2.5
0.0
2.5
MR effect size for
'CD11b on CD66b++ myeloid cell' on 'Hypertrophic cardiomyopathy'

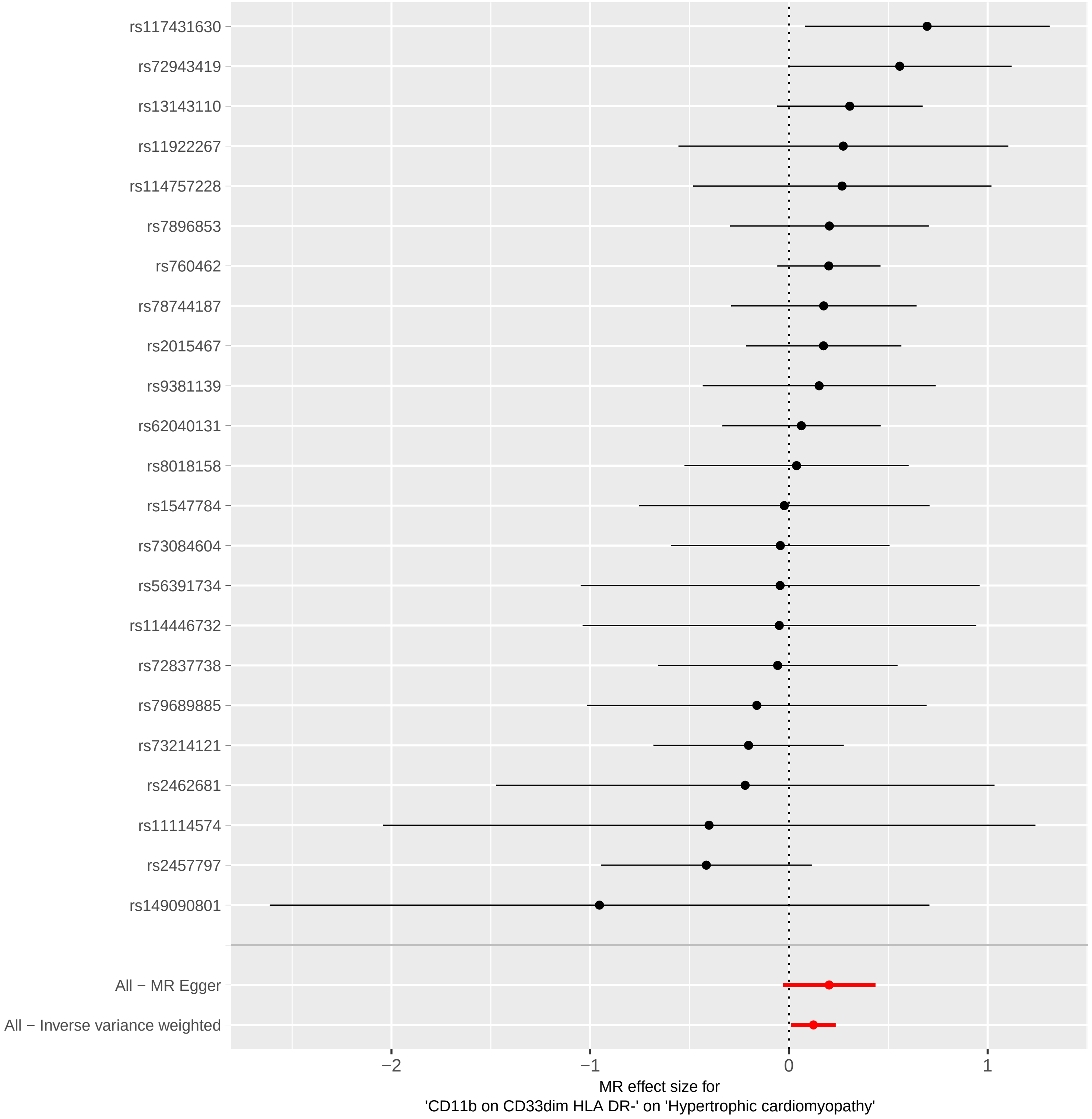

rs117431630
rs72943419
rs13143110
rs11922267
rs114757228
rs7896853
rs760462
rs78744187
rs2015467
rs9381139
rs62040131
rs8018158
rs1547784
rs73084604
rs56391734
rs114446732
rs72837738
rs79689885
rs73214121
rs2462681
rs11114574
rs2457797
rs149090801
All − MR Egger
All − Inverse variance weighted
−2
−1
0
1
MR effect size for
'CD11b on CD33dim HLA DR-' on 'Hypertrophic cardiomyopathy'

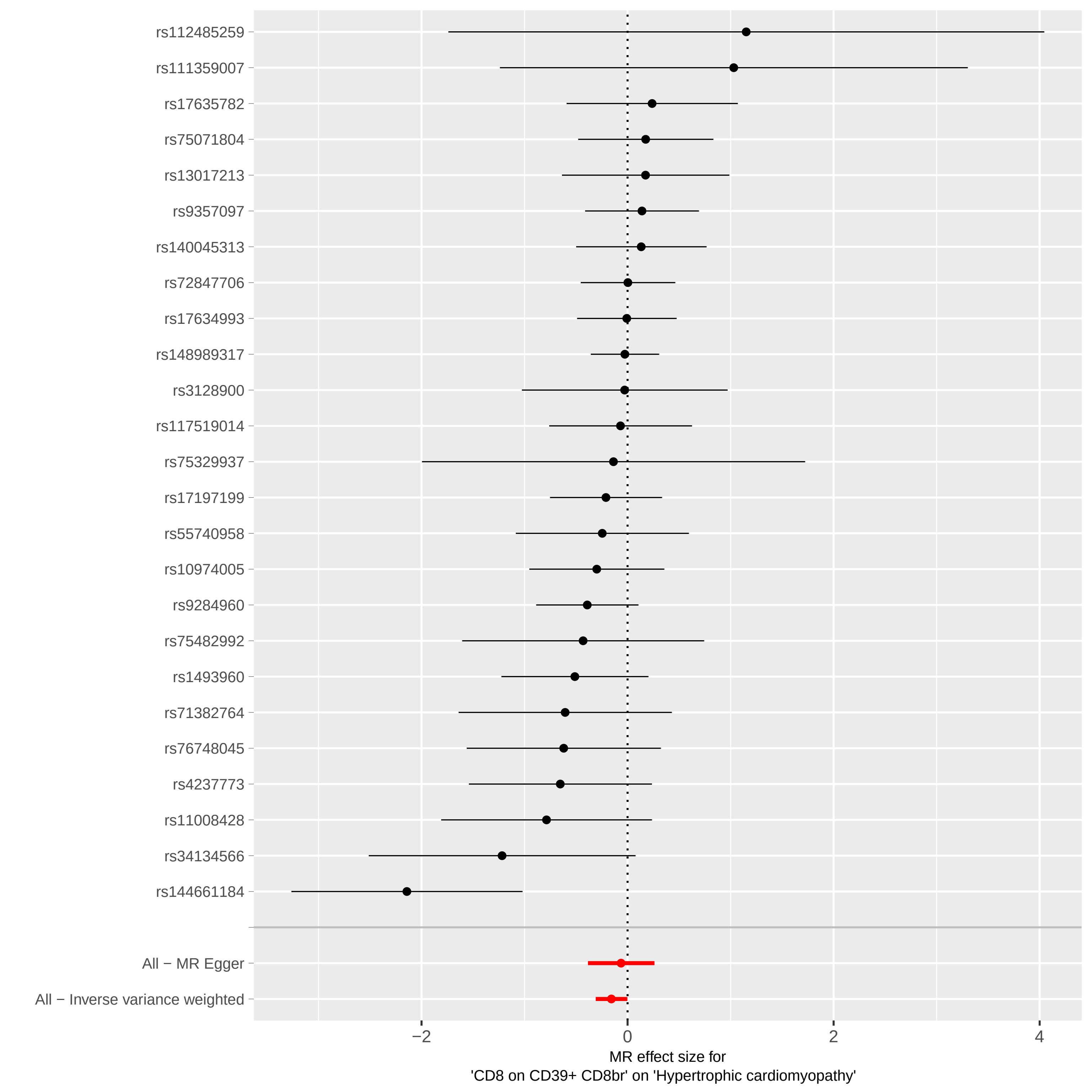
rs112485259
rs111359007
rs17635782
rs75071804
rs13017213
rs9357097
rs140045313
rs72847706
rs17634993
rs148989317
rs3128900
rs117519014
rs75329937
rs17197199
rs55740958
rs10974005
rs9284960
rs75482992
rs1493960
rs71382764
rs76748045
rs4237773
rs11008428
rs34134566
rs144661184
All − MR Egger
All − Inverse variance weighted
−2
0
2
4
MR effect size for
'CD8 on CD39+ CD8br' on 'Hypertrophic cardiomyopathy'

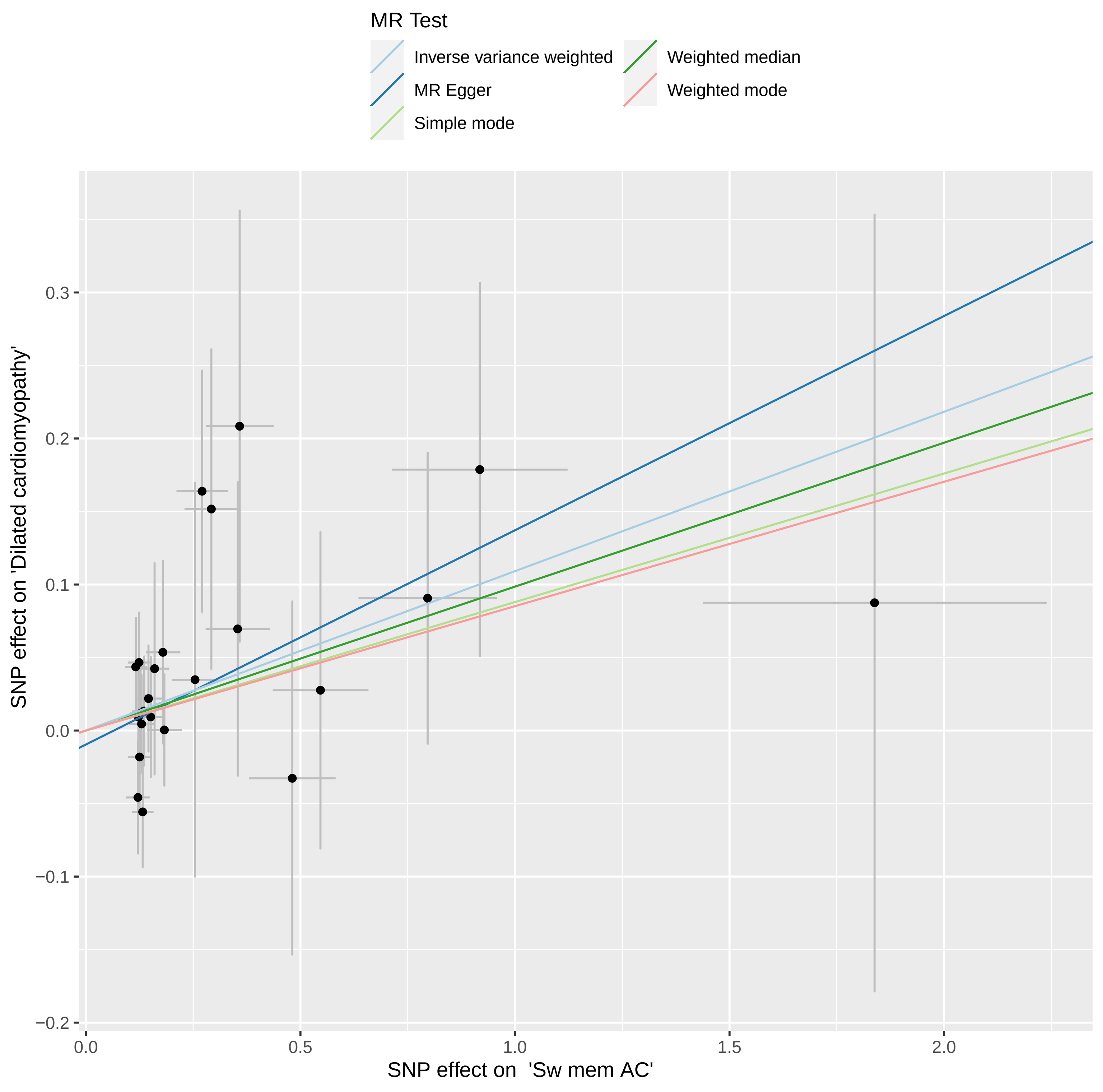

MR Test
Inverse variance weighted
MR Egger
Simple mode
Weighted median
Weighted mode
SNP effect on 'Dilated cardiomyopathy'
0.3
0.2
0.1
0.0
−0.1
−0.2
0.0
0.5
1.0
1.5
2.0
SNP effect on 'Sw mem AC'

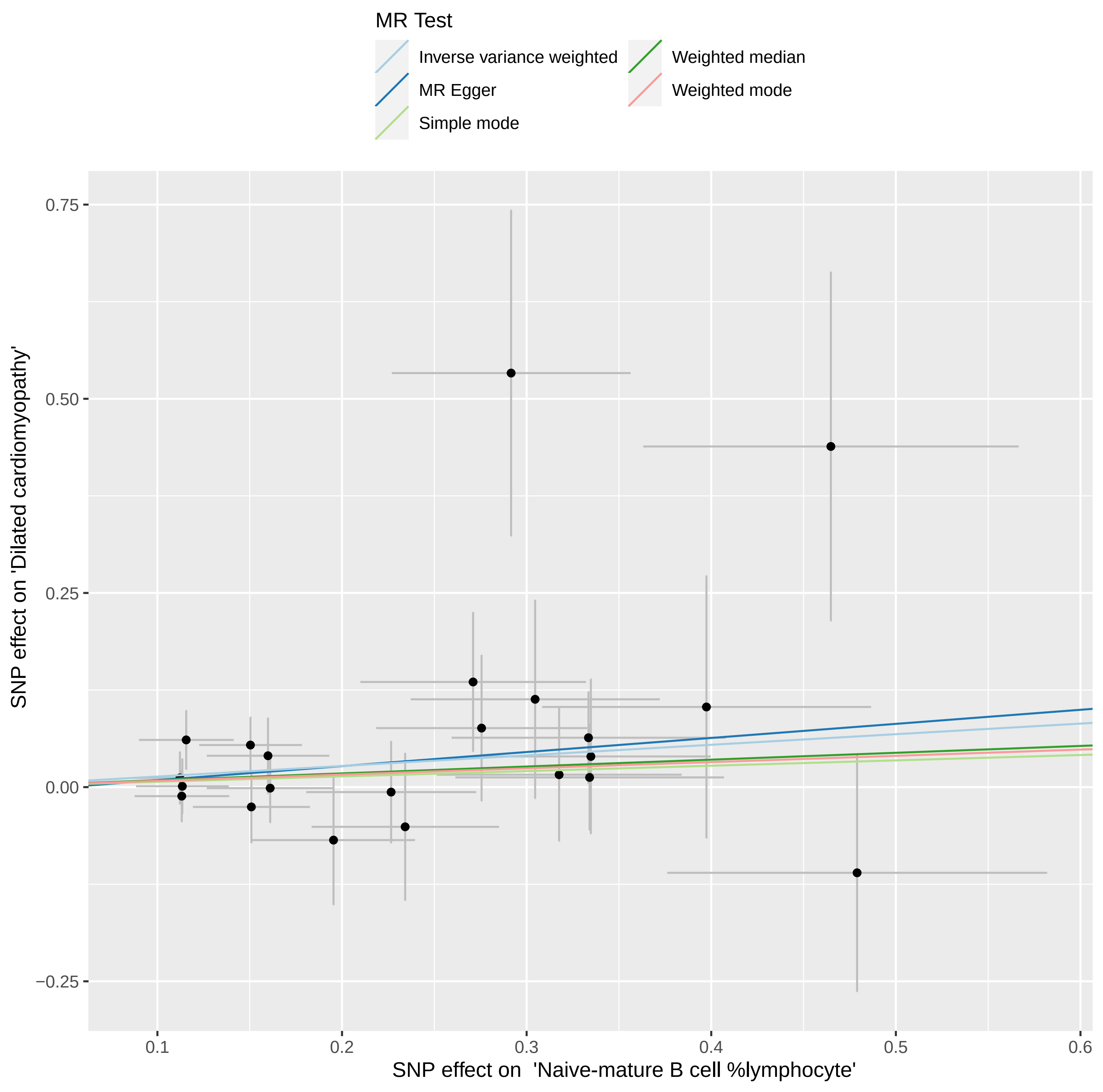
MR Test
Inverse variance weighted
MR Egger
Simple mode
Weighted median
Weighted mode
SNP effect on 'Dilated cardiomyopathy'
0.75
0.50
0.25
0.00
−0.25
0.1
0.2
0.3
0.4
0.5
0.6
SNP effect on 'Naive-mature B cell %lymphocyte'

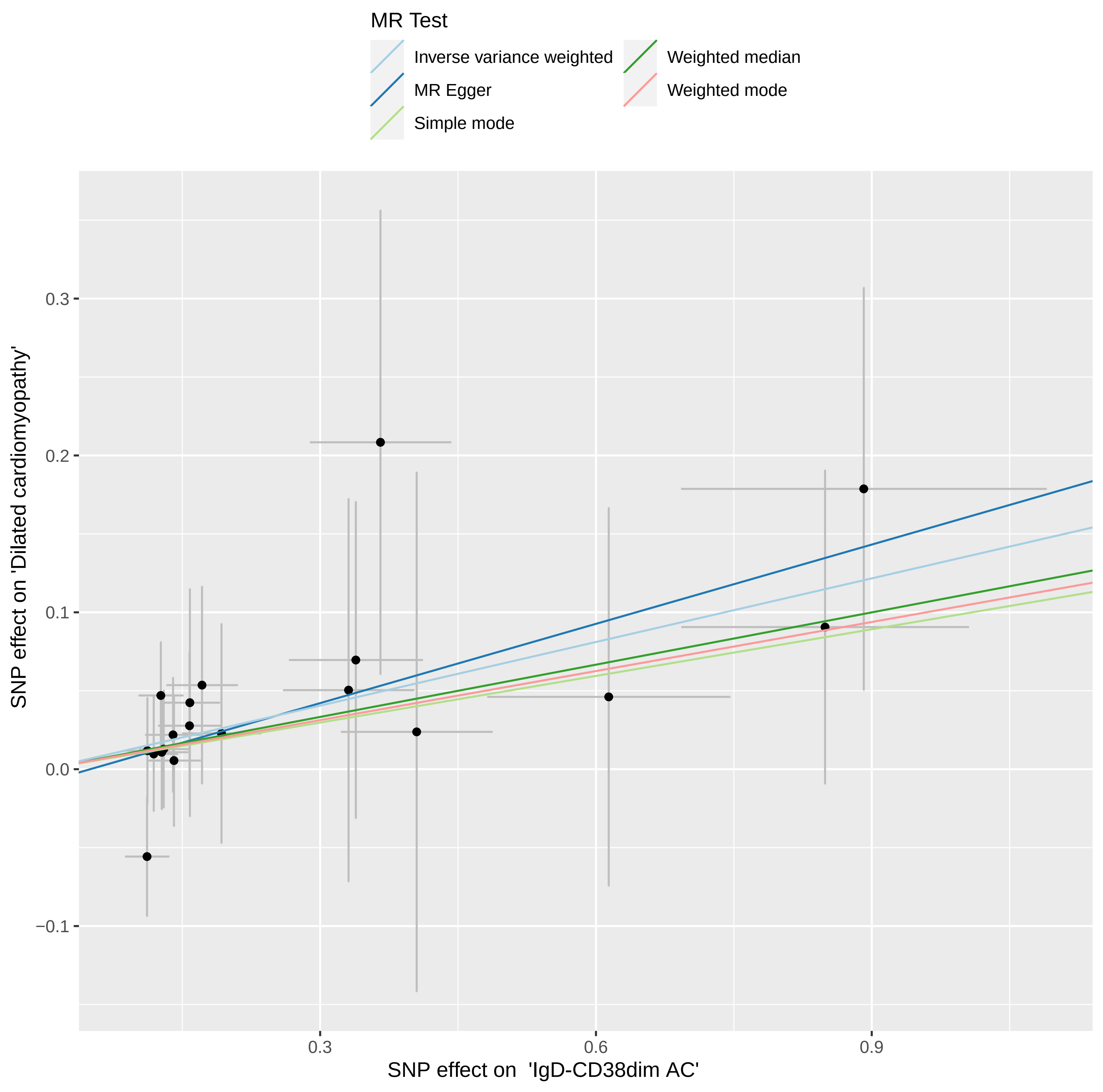

MR Test
Inverse variance weighted
MR Egger
Simple mode
Weighted median
Weighted mode
SNP effect on 'Dilated cardiomyopathy'
0.3
0.2
0.1
0.0
−0.1
0.3
0.6
0.9
SNP effect on 'IgD-CD38dim AC'

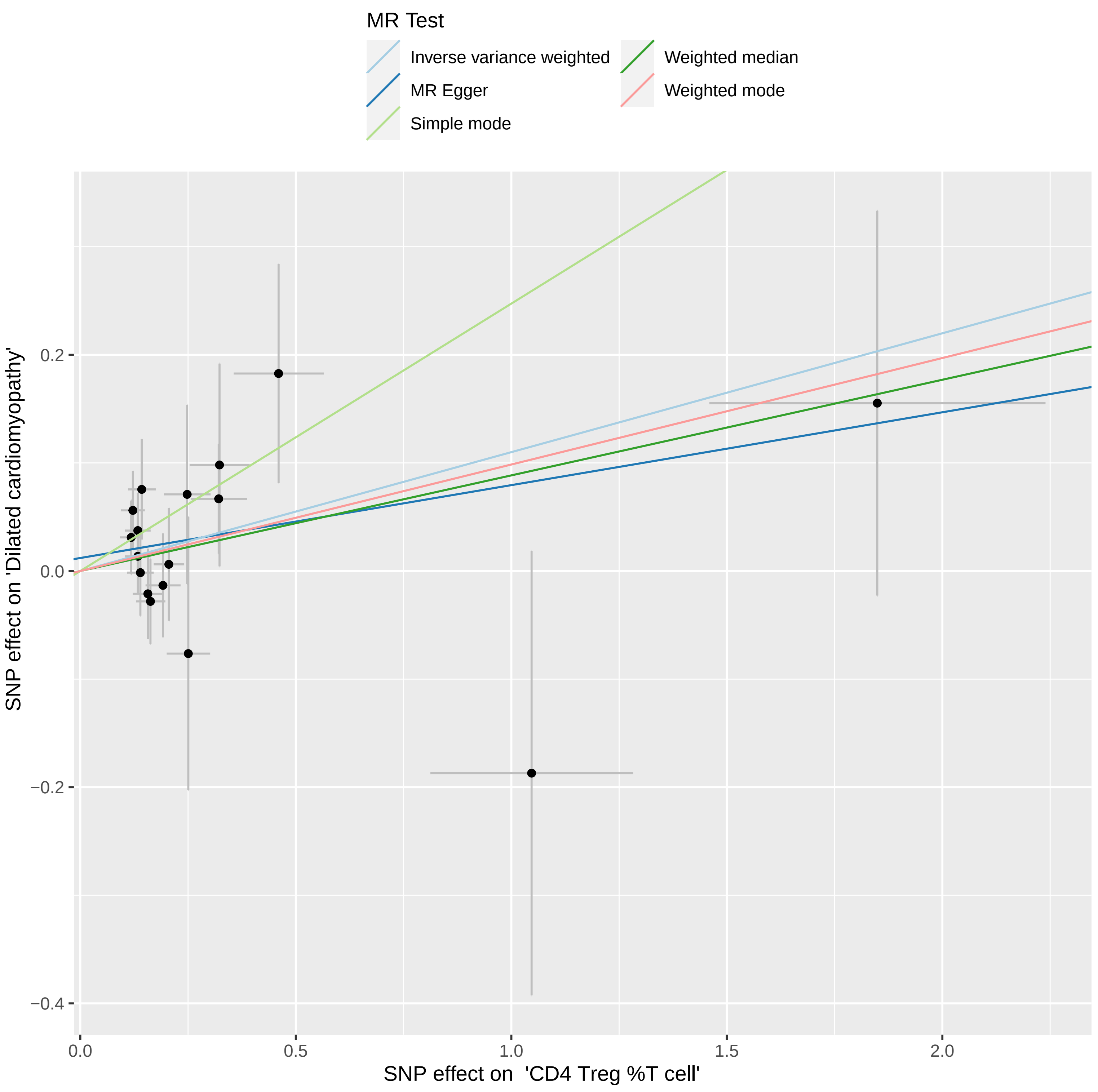
MR Test
Inverse variance weighted
MR Egger
Simple mode
Weighted median
Weighted mode
SNP effect on 'Dilated cardiomyopathy'
0.2
0.0
−0.2
−0.4
0.0
0.5
1.0
1.5
2.0
SNP effect on 'CD4 Treg %T cell'

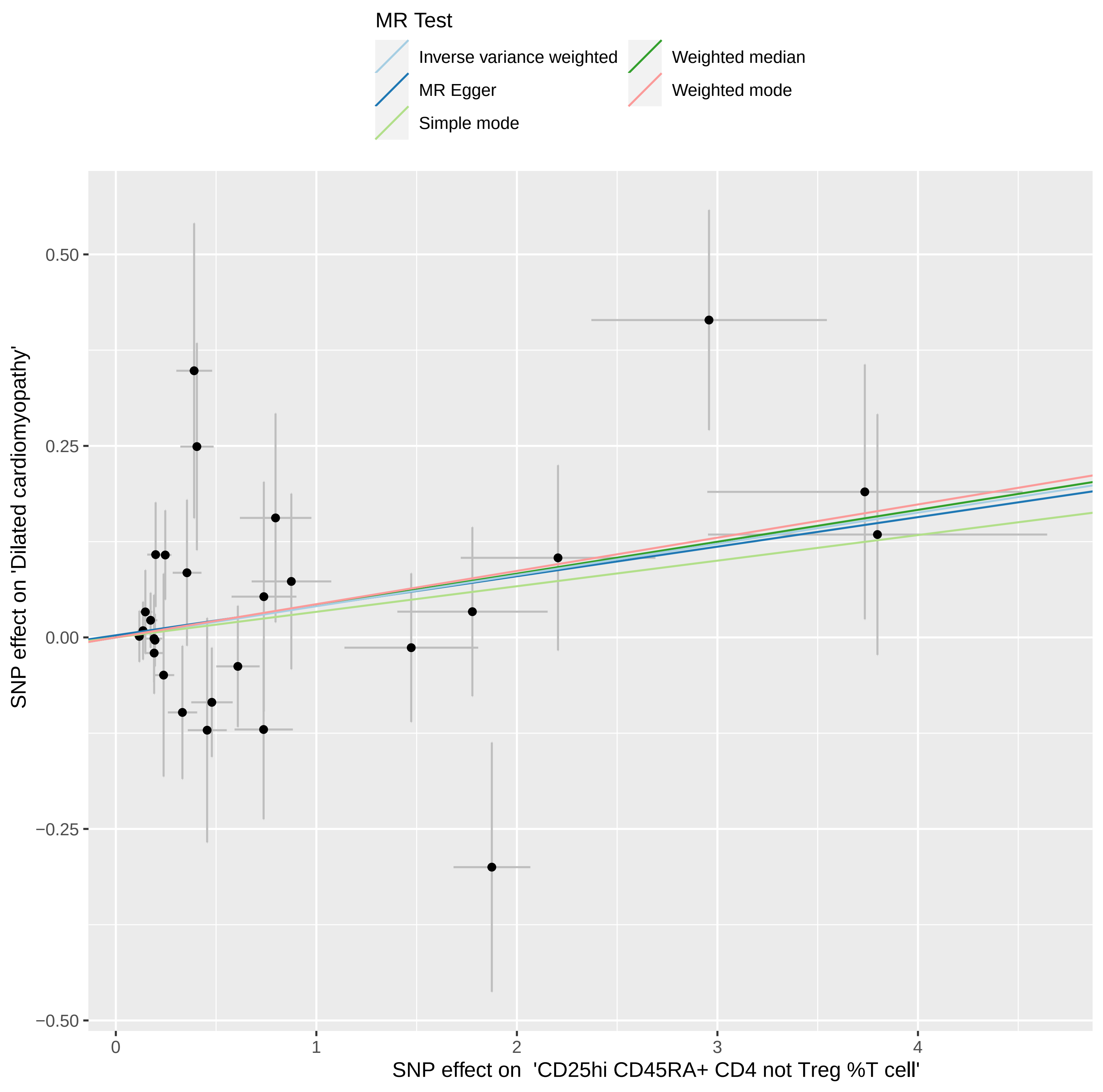
MR Test
Inverse variance weighted
MR Egger
Simple mode
Weighted median
Weighted mode
0.50
0.25
0.00
−0.25
−0.50
0
1
2
3
4
SNP effect on 'Dilated cardiomyopathy'
SNP effect on 'CD25hi CD45RA+ CD4 not Treg %T cell'

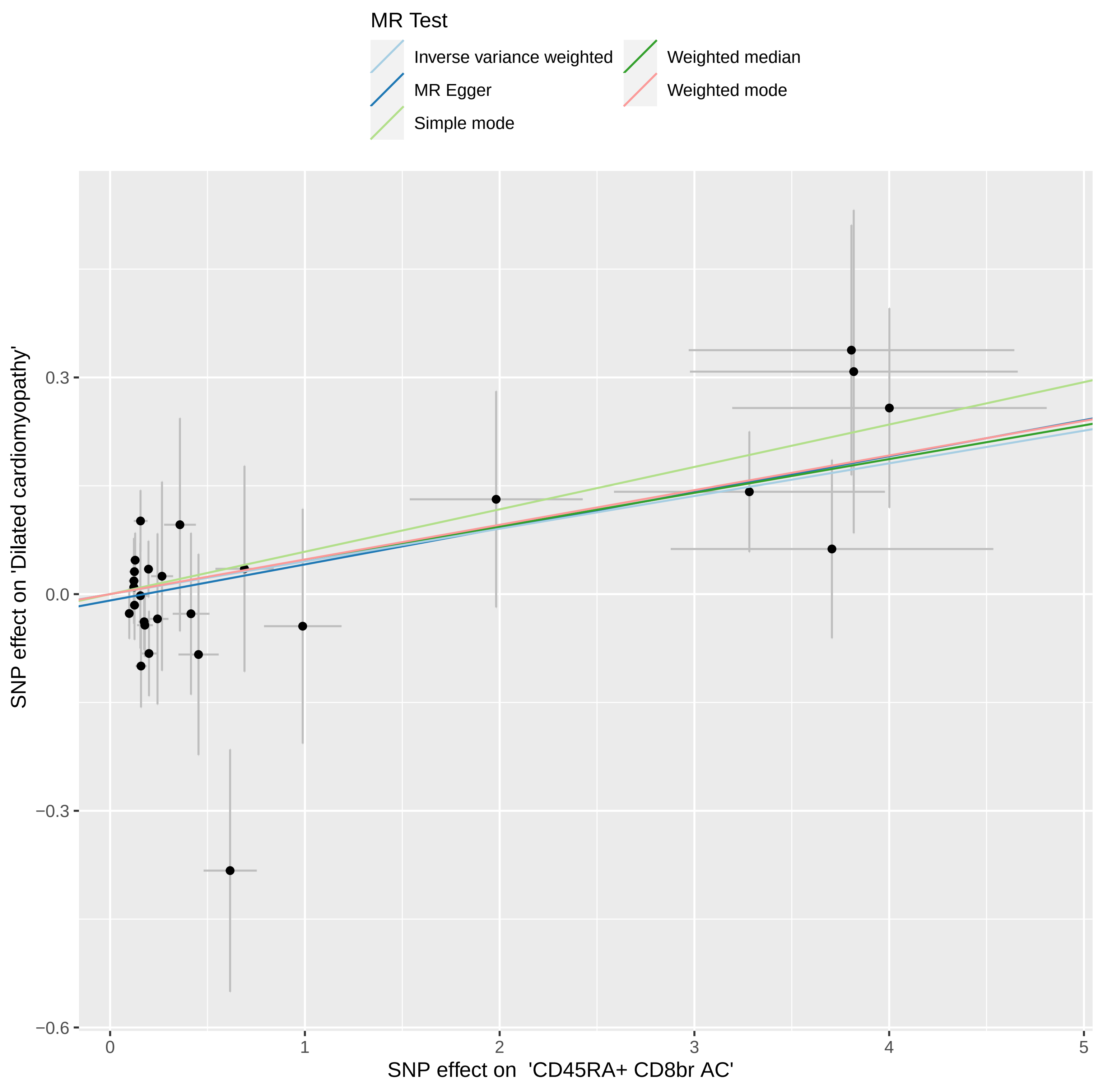
MR Test
Inverse variance weighted
MR Egger
Simple mode
Weighted median
Weighted mode
SNP effect on 'Dilated cardiomyopathy'
0.3
0.0
−0.3
−0.6
0
1
2
3
4
5
SNP effect on 'CD45RA+ CD8br AC'

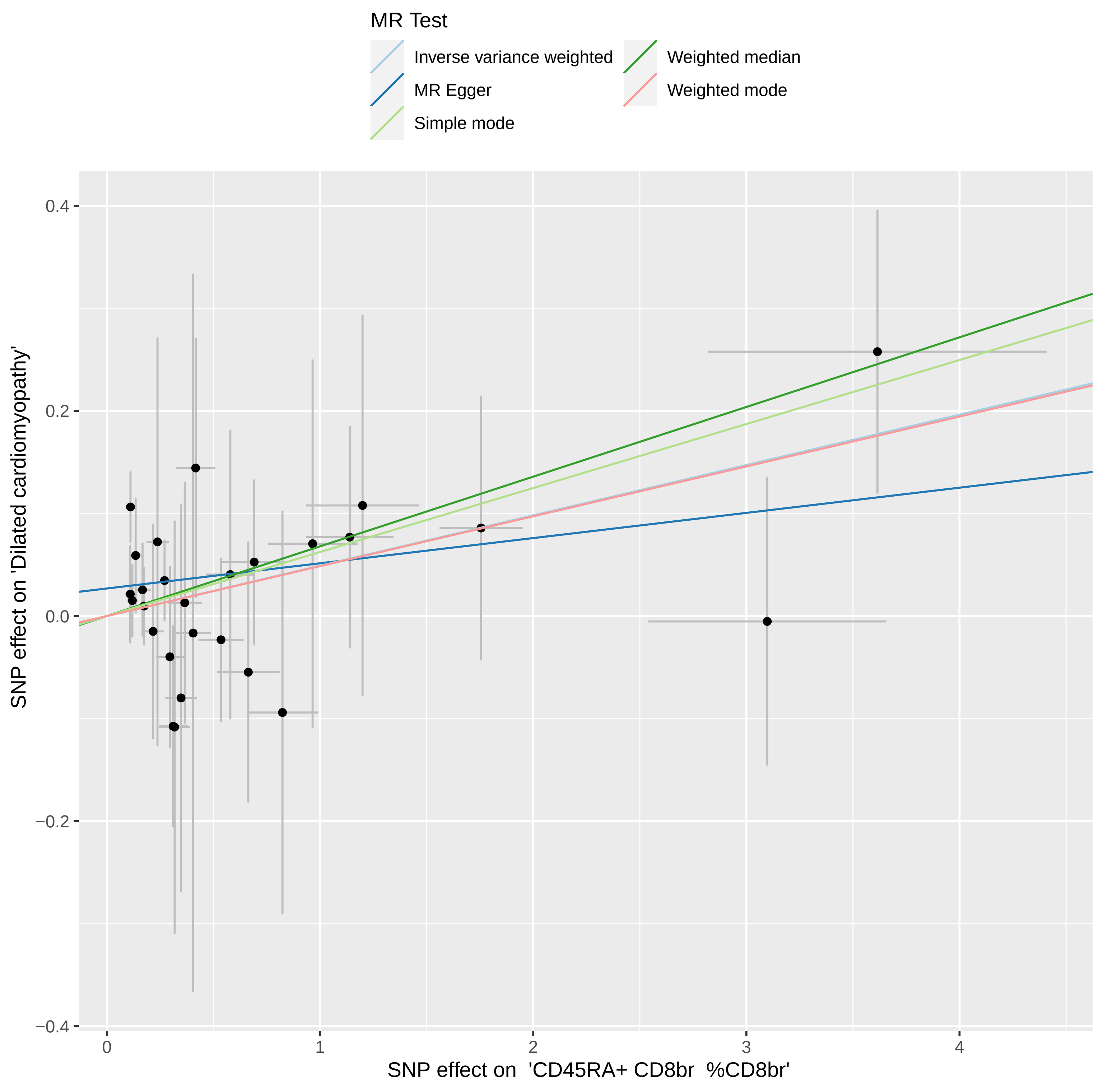

MR Test
Inverse variance weighted
MR Egger
Simple mode
Weighted median
Weighted mode
0.4
0.2
0.0
−0.2
−0.4
0
1
2
3
4
SNP effect on 'Dilated cardiomyopathy'
SNP effect on 'CD45RA+ CD8br %CD8br'

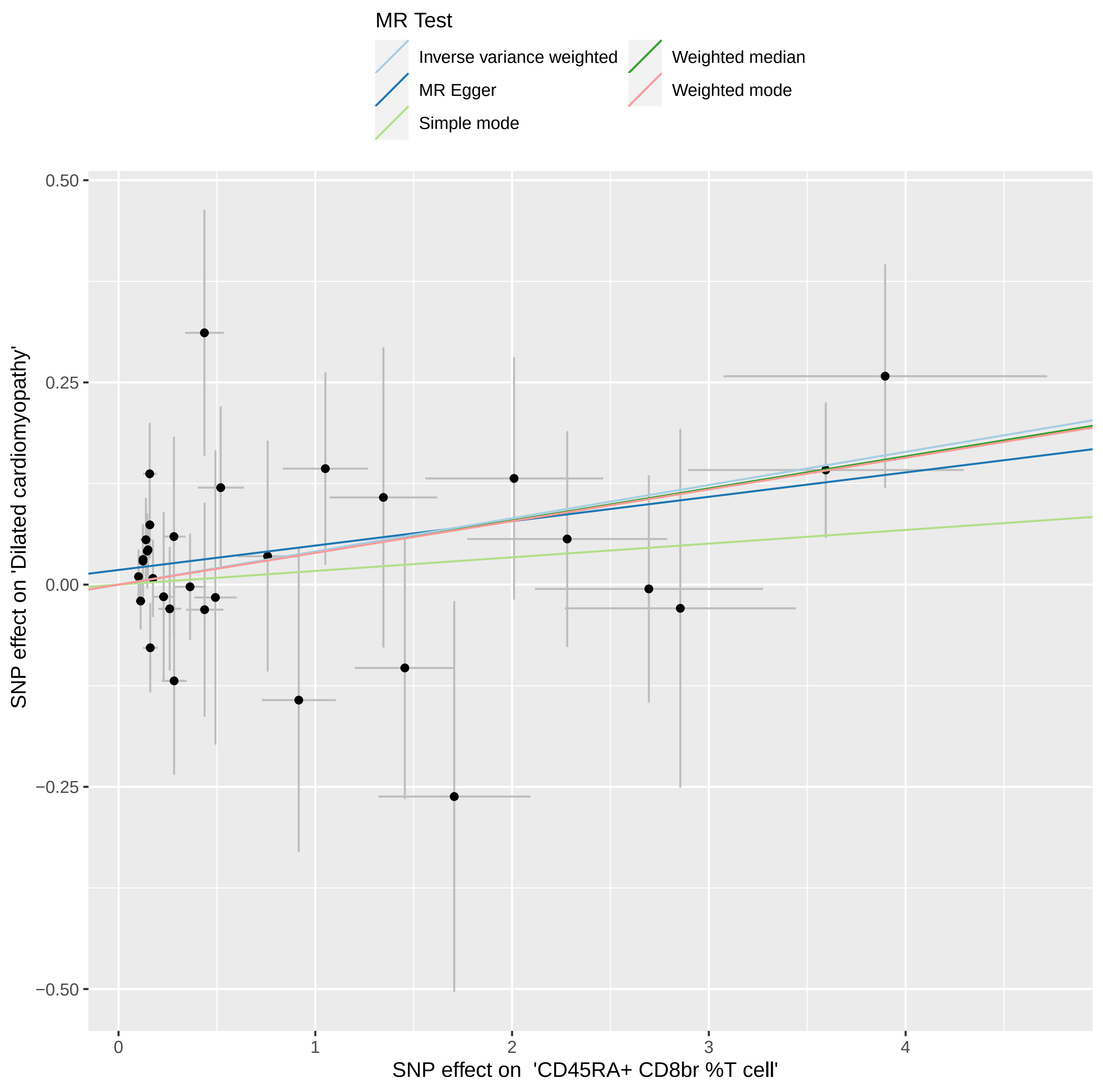
MR Test
Inverse variance weighted
MR Egger
Simple mode
Weighted median
Weighted mode
0.50
0.25
0.00
−0.25
−0.50
SNP effect on 'Dilated cardiomyopathy'
0
1
2
3
4
SNP effect on 'CD45RA+ CD8br %T cell'

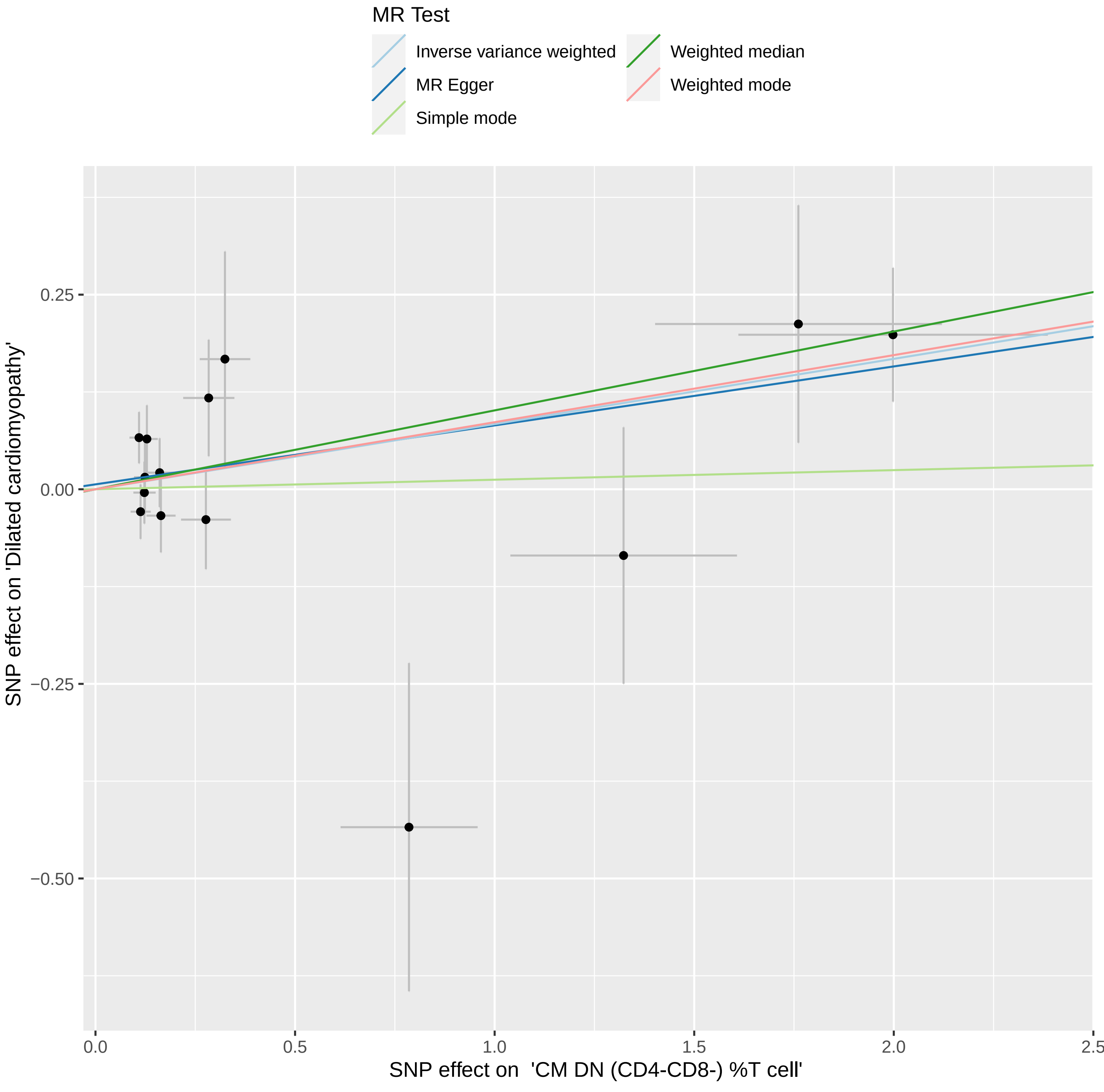

MR Test
Inverse variance weighted
MR Egger
Simple mode
Weighted median
Weighted mode
0.25
0.00
−0.25
−0.50
0.0
0.5
1.0
1.5
2.0
2.5
SNP effect on 'Dilated cardiomyopathy'
SNP effect on 'CM DN (CD4-CD8-) %T cell'

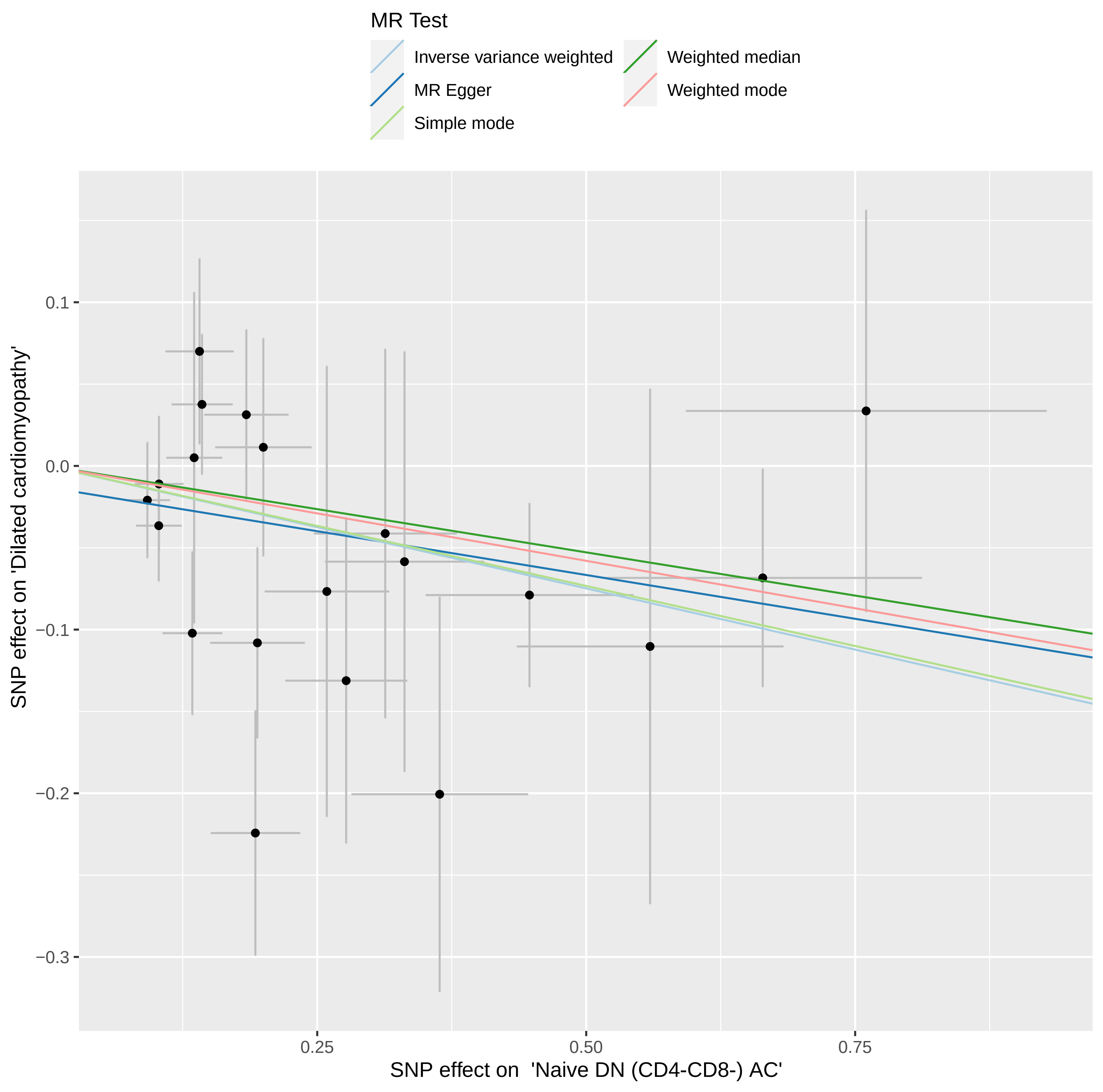
MR Test
Inverse variance weighted
MR Egger
Simple mode
Weighted median
Weighted mode
0.1
0.0
−0.1
−0.2
−0.3
0.25
0.50
0.75
SNP effect on 'Dilated cardiomyopathy'
SNP effect on 'Naive DN (CD4-CD8-) AC'

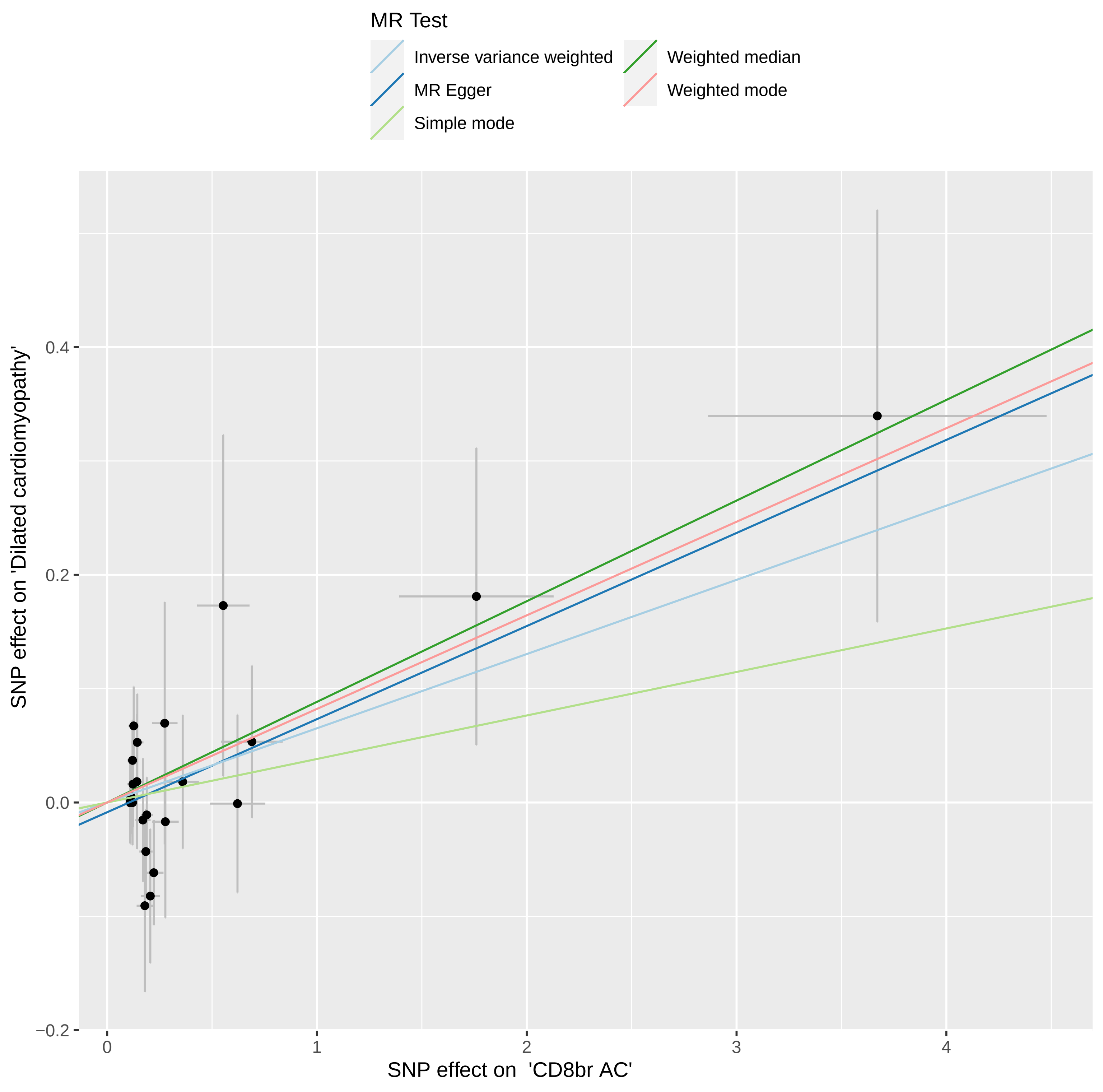

MR Test
Inverse variance weighted
MR Egger
Simple mode
Weighted median
Weighted mode
SNP effect on 'Dilated cardiomyopathy'
0.4
0.2
0.0
−0.2
0
1
2
3
4
SNP effect on 'CD8br AC'

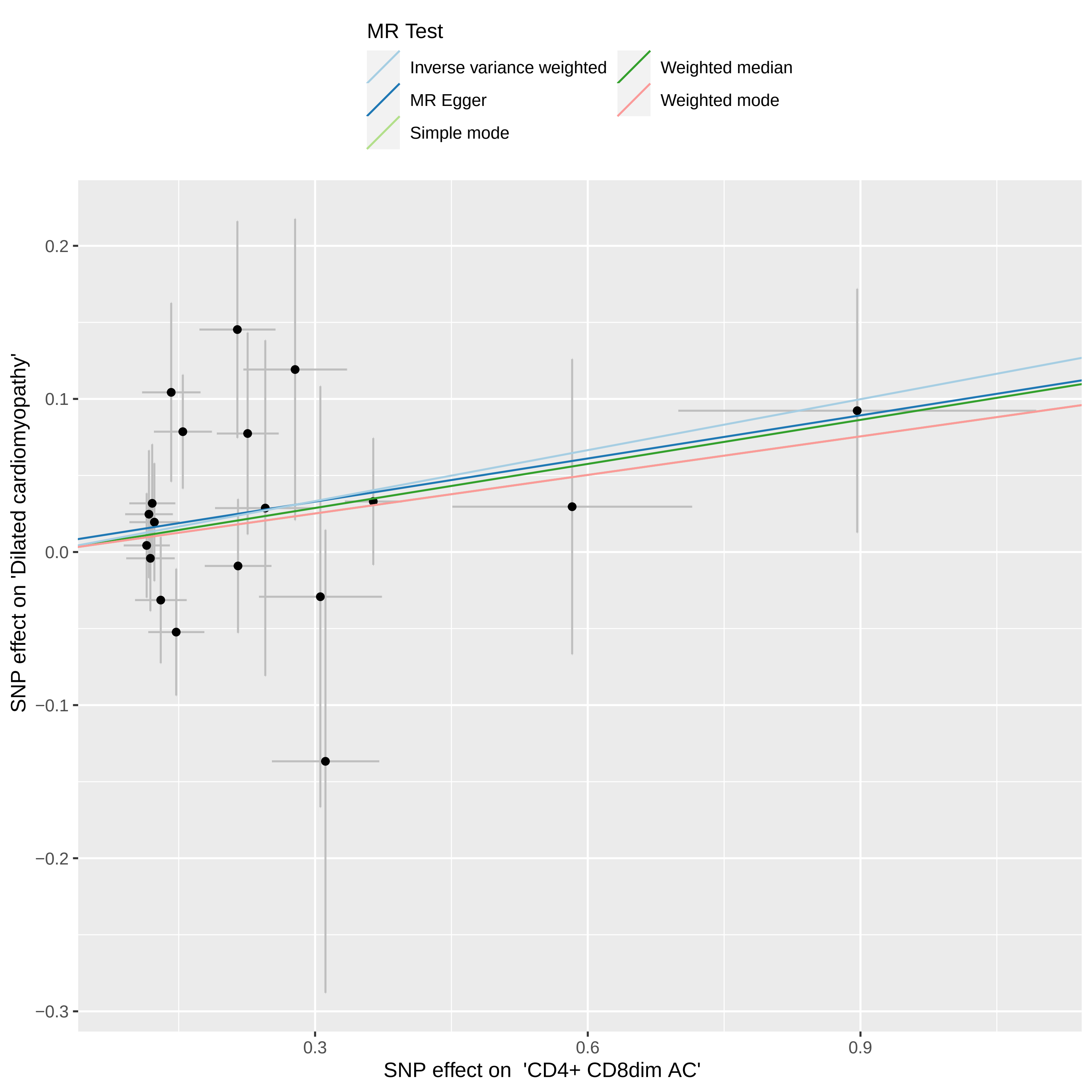
MR Test
Inverse variance weighted
MR Egger
Simple mode
Weighted median
Weighted mode
0.2
0.1
0.0
−0.1
−0.2
−0.3
0.3
0.6
0.9
SNP effect on 'Dilated cardiomyopathy'
SNP effect on 'CD4+ CD8dim AC'

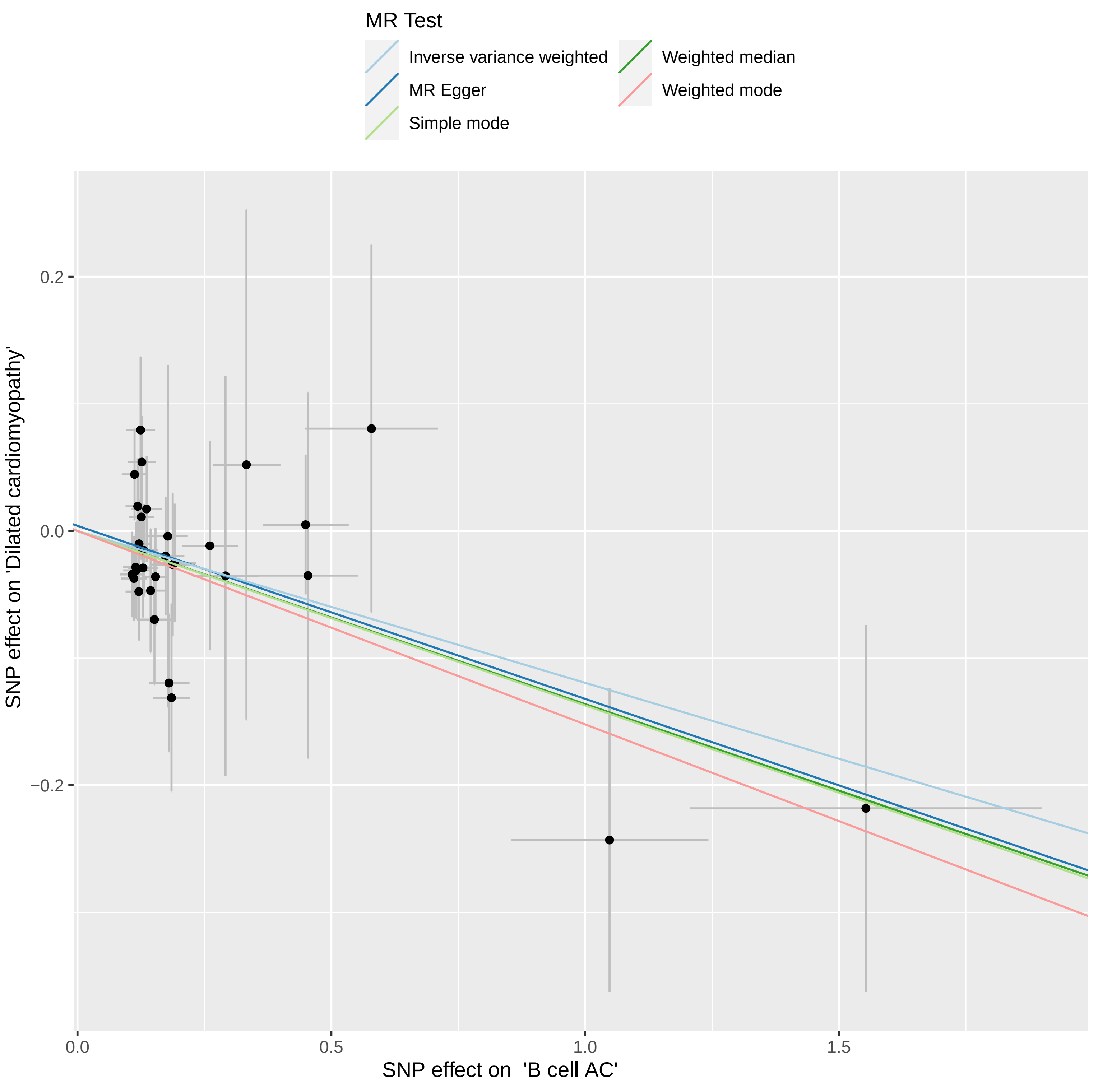
MR Test
Inverse variance weighted
MR Egger
Simple mode
Weighted median
Weighted mode
0.2
0.0
−0.2
0.0
0.5
1.0
1.5
SNP effect on 'Dilated cardiomyopathy'
SNP effect on 'B cell AC'

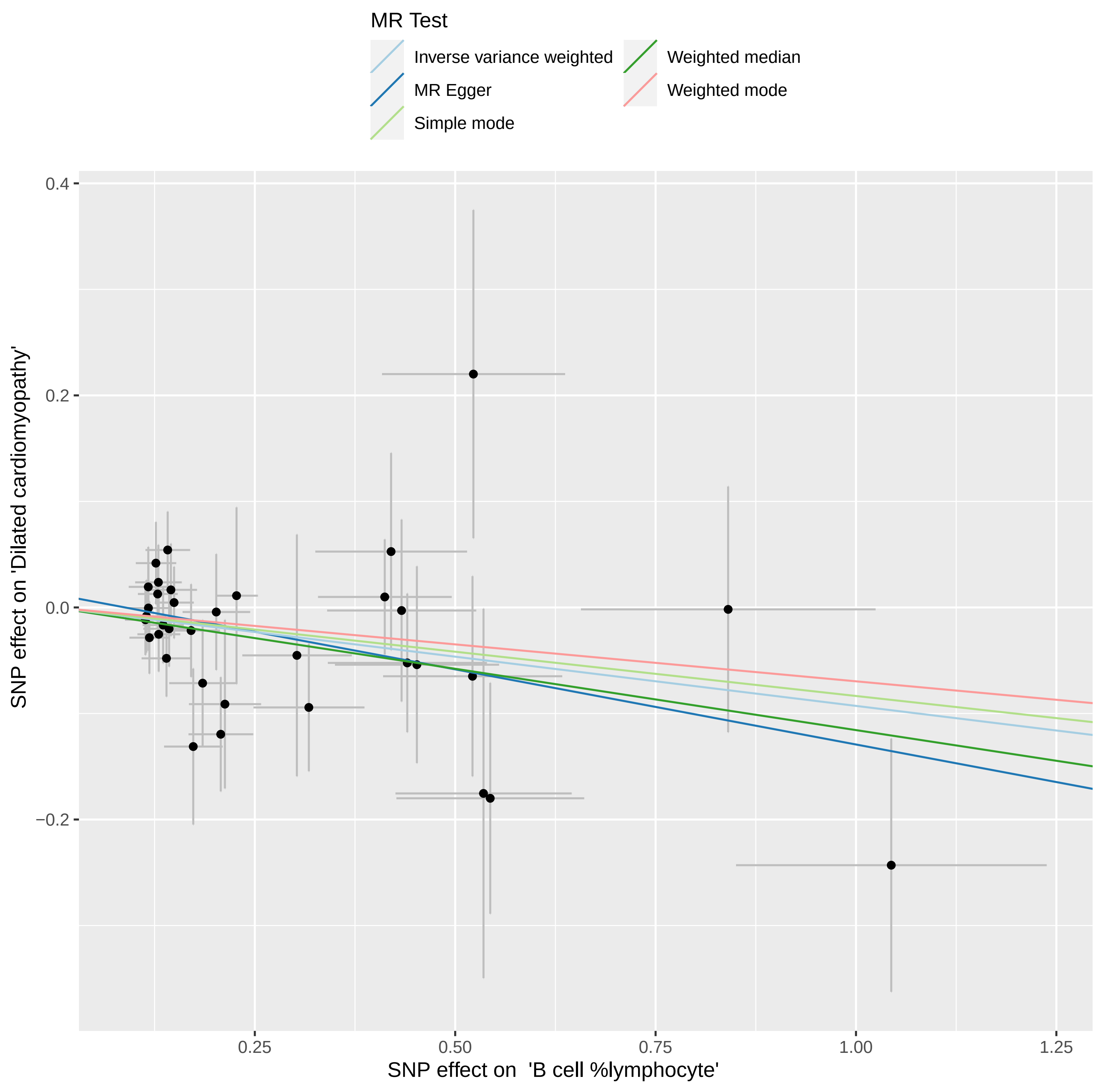

MR Test
Inverse variance weighted
MR Egger
Simple mode
Weighted median
Weighted mode
0.4
0.2
0.0
−0.2
SNP effect on 'Dilated cardiomyopathy'
0.25
0.50
0.75
1.00
1.25
SNP effect on 'B cell %lymphocyte'

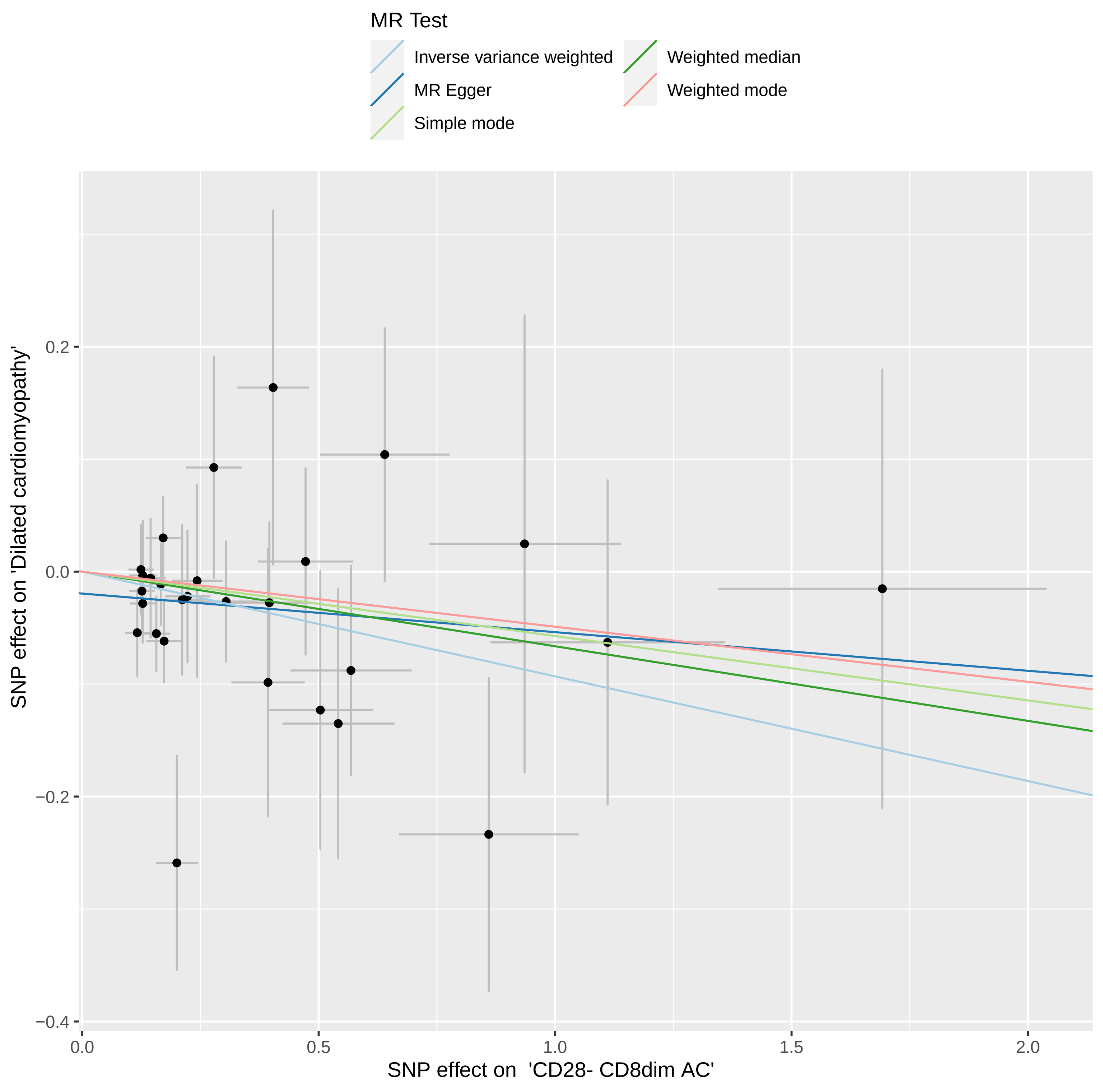

MR Test
Inverse variance weighted
MR Egger
Simple mode
Weighted median
Weighted mode
0.2
0.0
−0.2
−0.4
0.0
0.5
1.0
1.5
2.0
SNP effect on 'Dilated cardiomyopathy'
SNP effect on 'CD28- CD8dim AC'

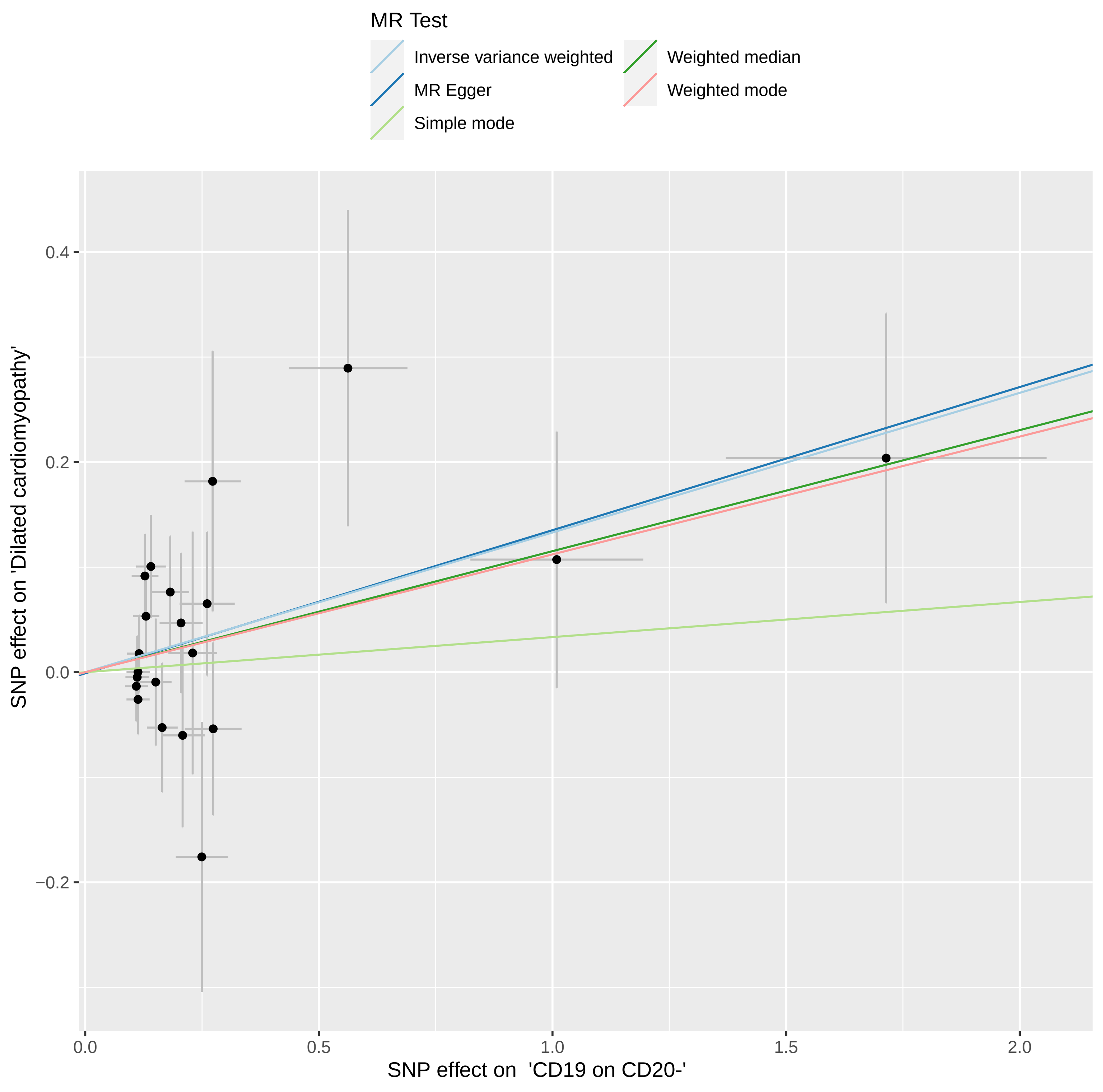
MR Test
Inverse variance weighted
MR Egger
Simple mode
Weighted median
Weighted mode
0.4
0.2
0.0
−0.2
0.0
0.5
1.0
1.5
2.0
SNP effect on 'Dilated cardiomyopathy'
SNP effect on 'CD19 on CD20-'

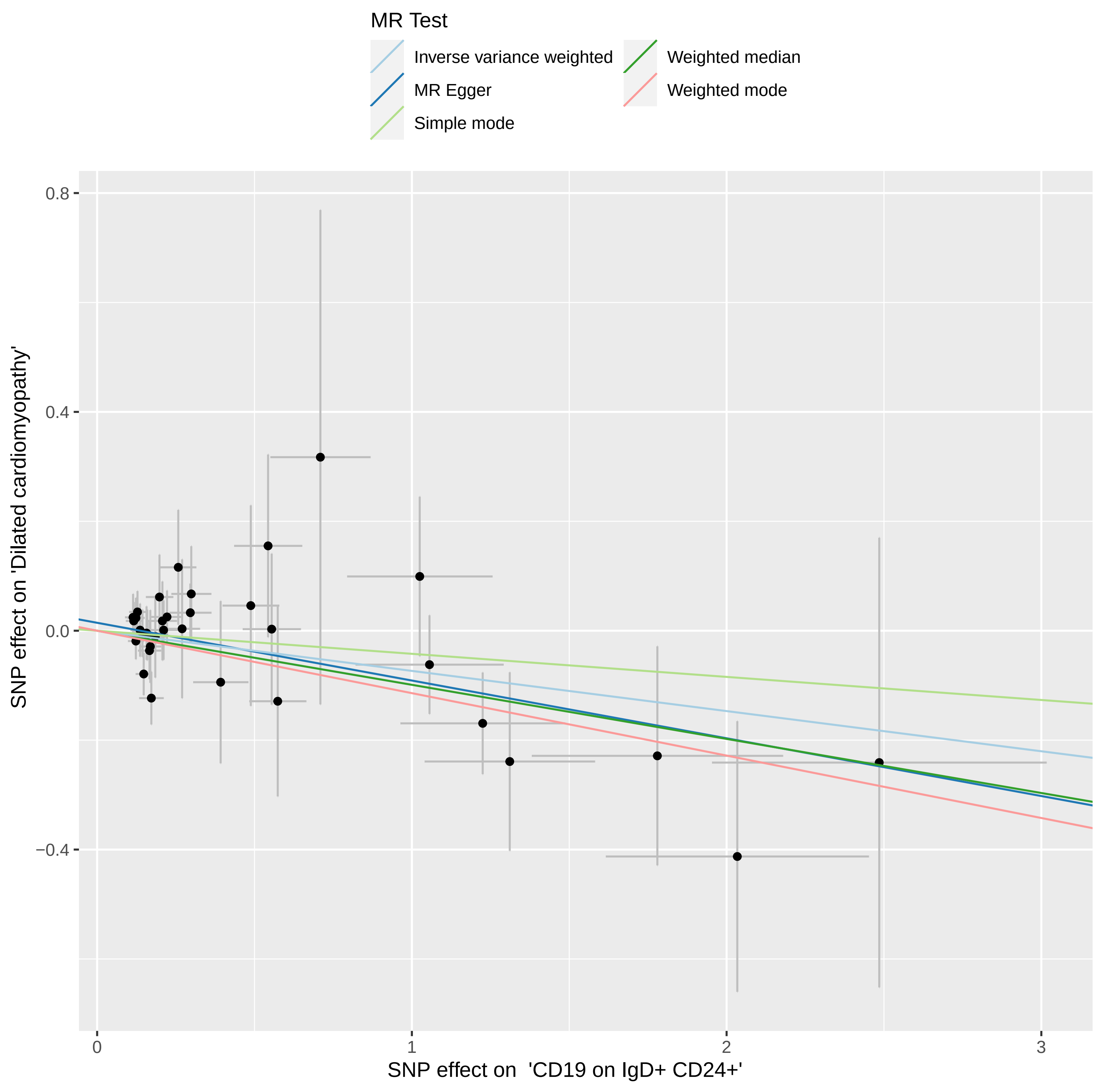
MR Test
Inverse variance weighted
MR Egger
Simple mode
Weighted median
Weighted mode
0.8
0.4
0.0
−0.4
0
1
2
3
SNP effect on 'Dilated cardiomyopathy'
SNP effect on 'CD19 on IgD+ CD24+'

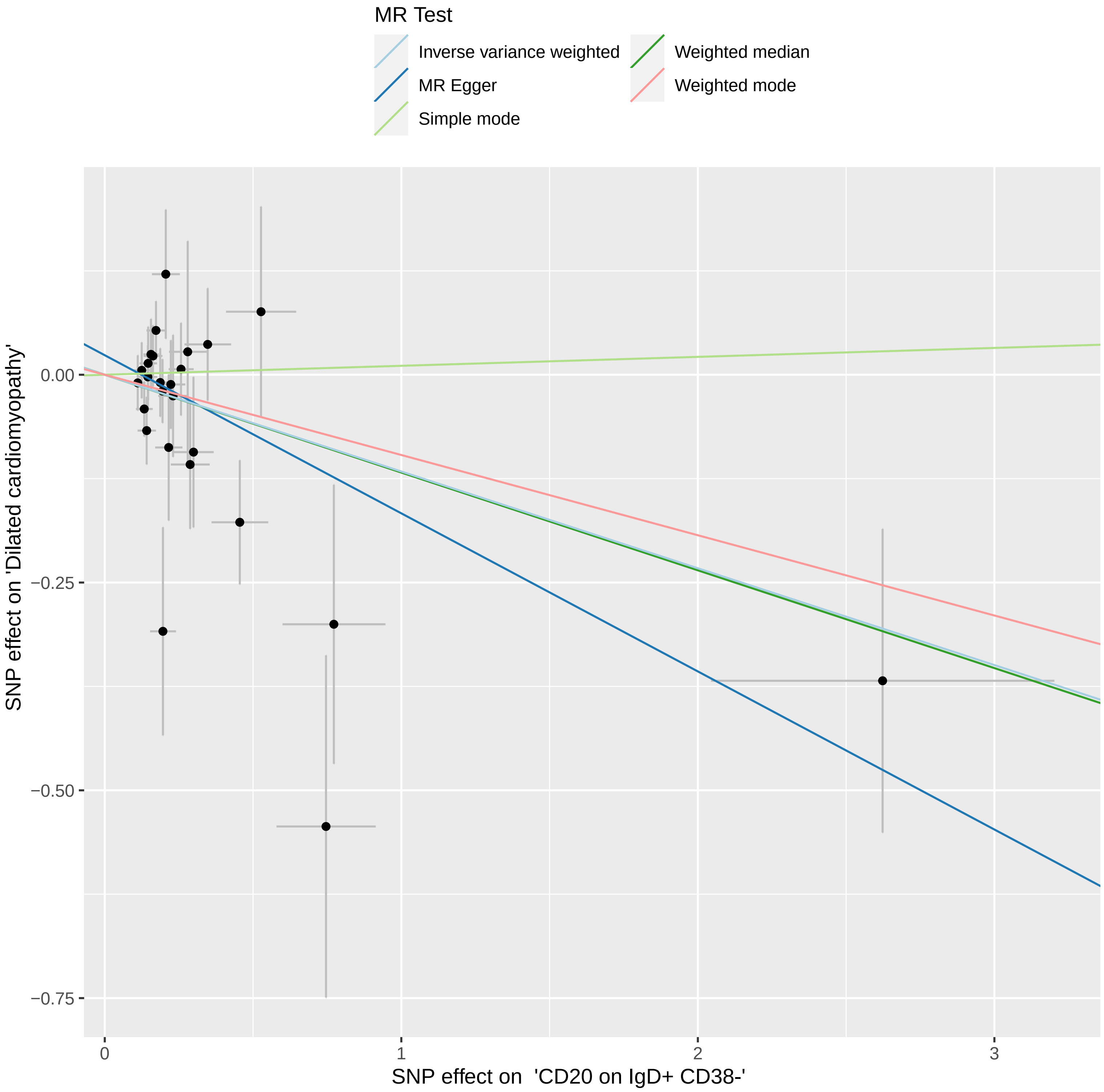

MR Test
Inverse variance weighted
MR Egger
Simple mode
Weighted median
Weighted mode
SNP effect on 'Dilated cardiomyopathy'
0.00
−0.25
−0.50
−0.75
0
1
2
3
SNP effect on 'CD20 on IgD+ CD38-'

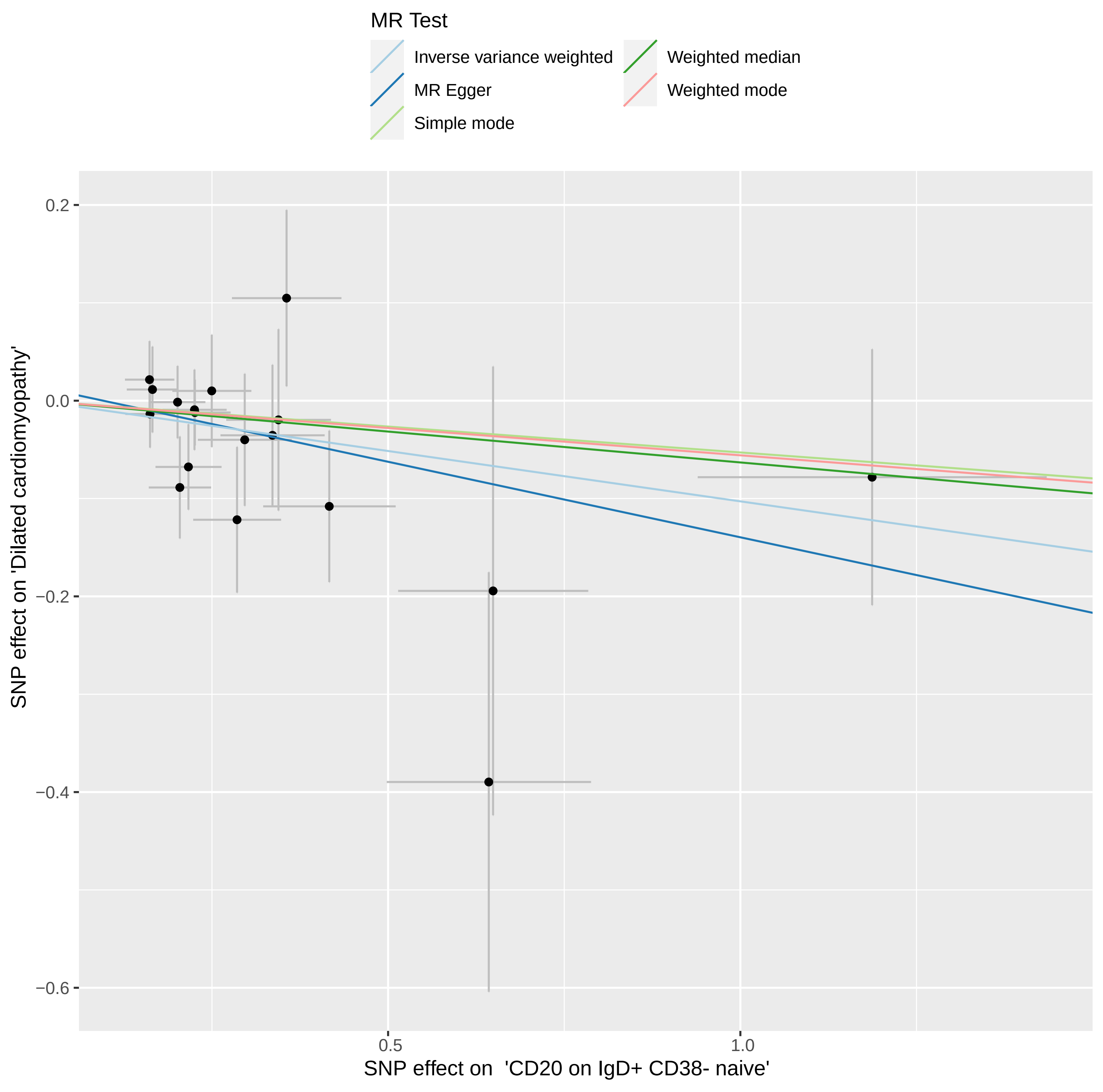
MR Test
Inverse variance weighted
MR Egger
Simple mode
Weighted median
Weighted mode
0.2
0.0
−0.2
−0.4
−0.6
0.5
1.0
SNP effect on 'Dilated cardiomyopathy'
SNP effect on 'CD20 on IgD+ CD38- naive'

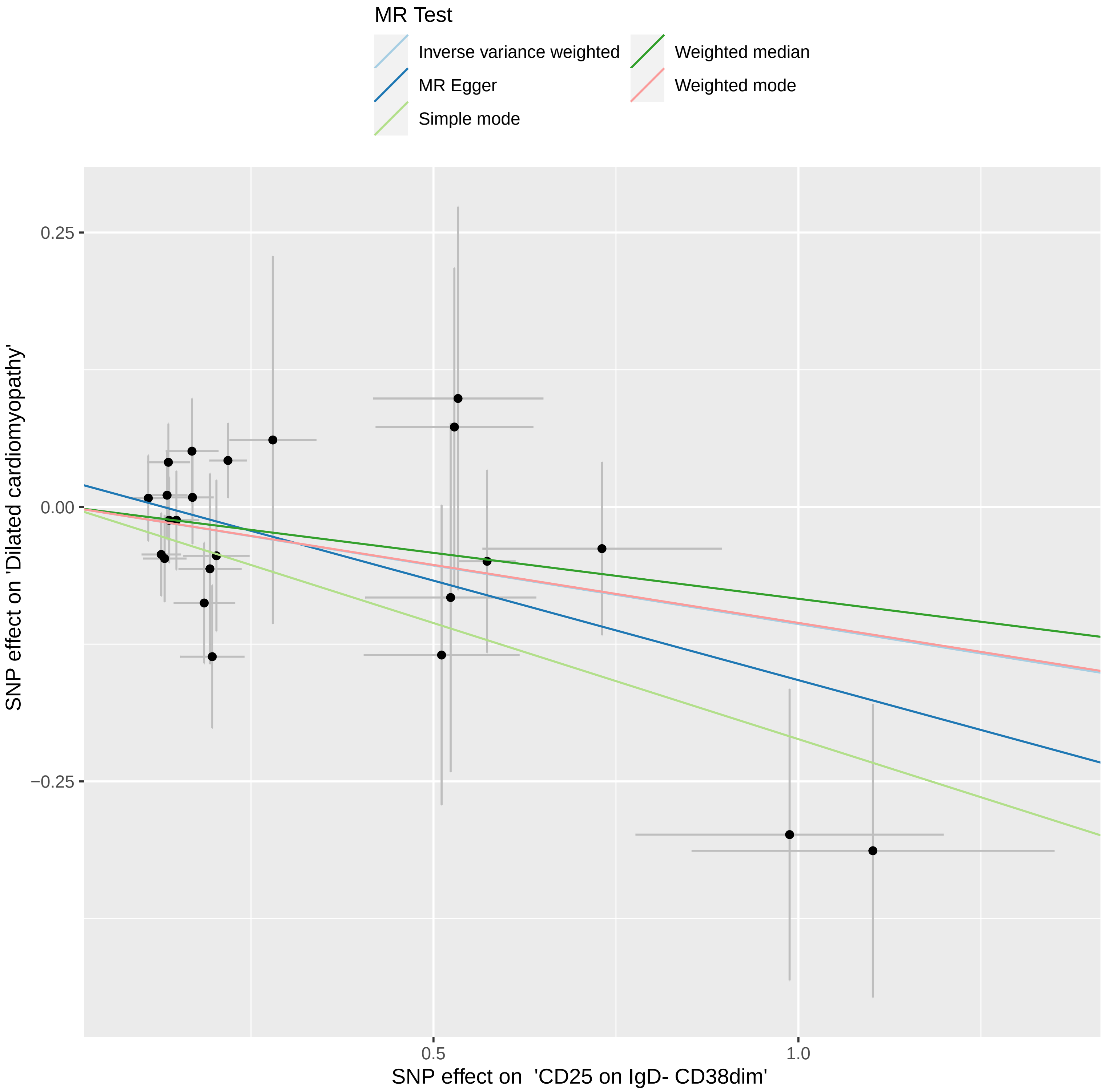

MR Test
Inverse variance weighted
MR Egger
Simple mode
Weighted median
Weighted mode
0.25
0.00
−0.25
0.5
1.0
SNP effect on 'Dilated cardiomyopathy'
SNP effect on 'CD25 on IgD- CD38dim'

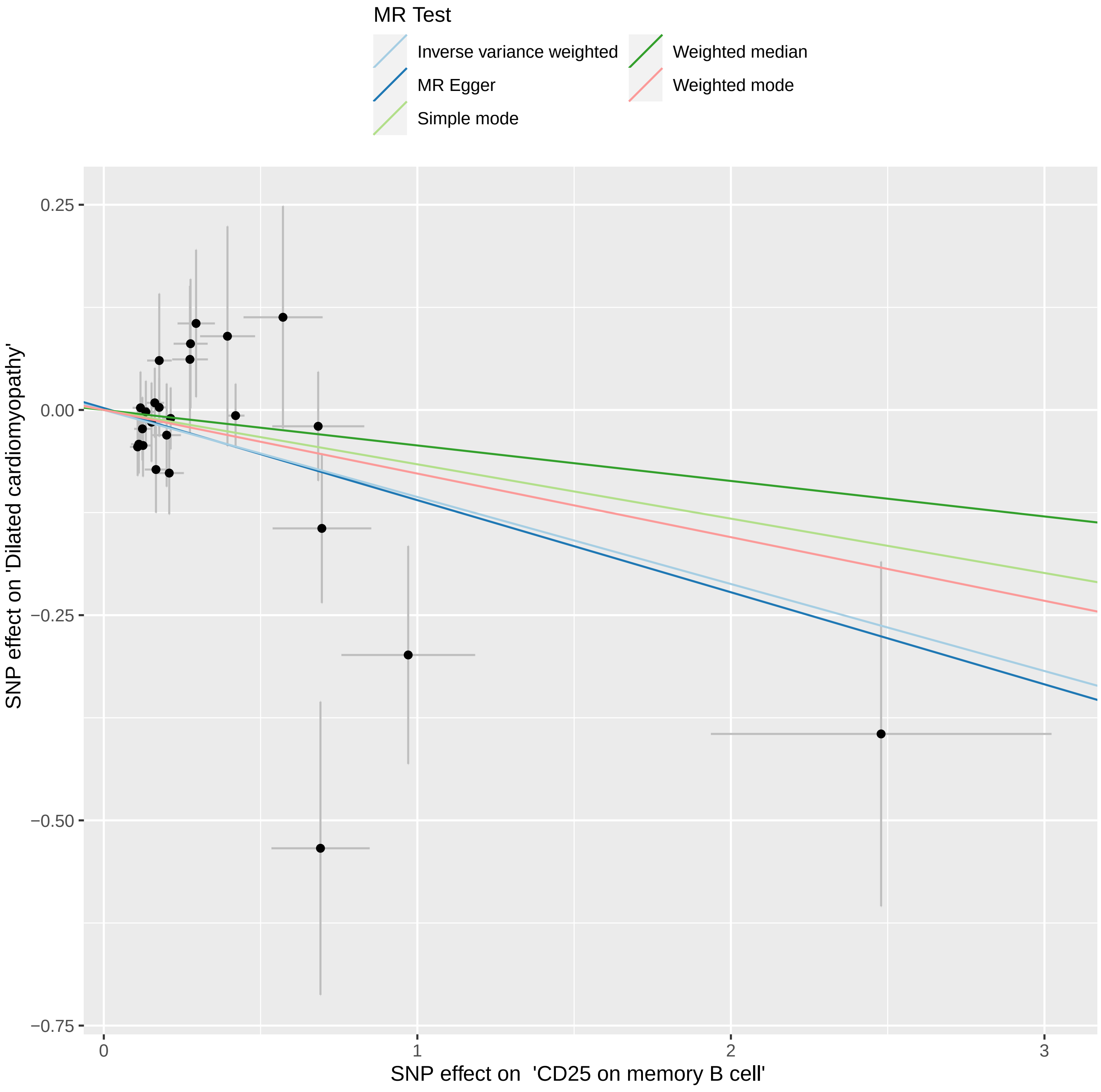
MR Test
Inverse variance weighted
MR Egger
Simple mode
Weighted median
Weighted mode
0.25
0.00
−0.25
−0.50
−0.75
0
1
2
3
SNP effect on 'Dilated cardiomyopathy'
SNP effect on 'CD25 on memory B cell'

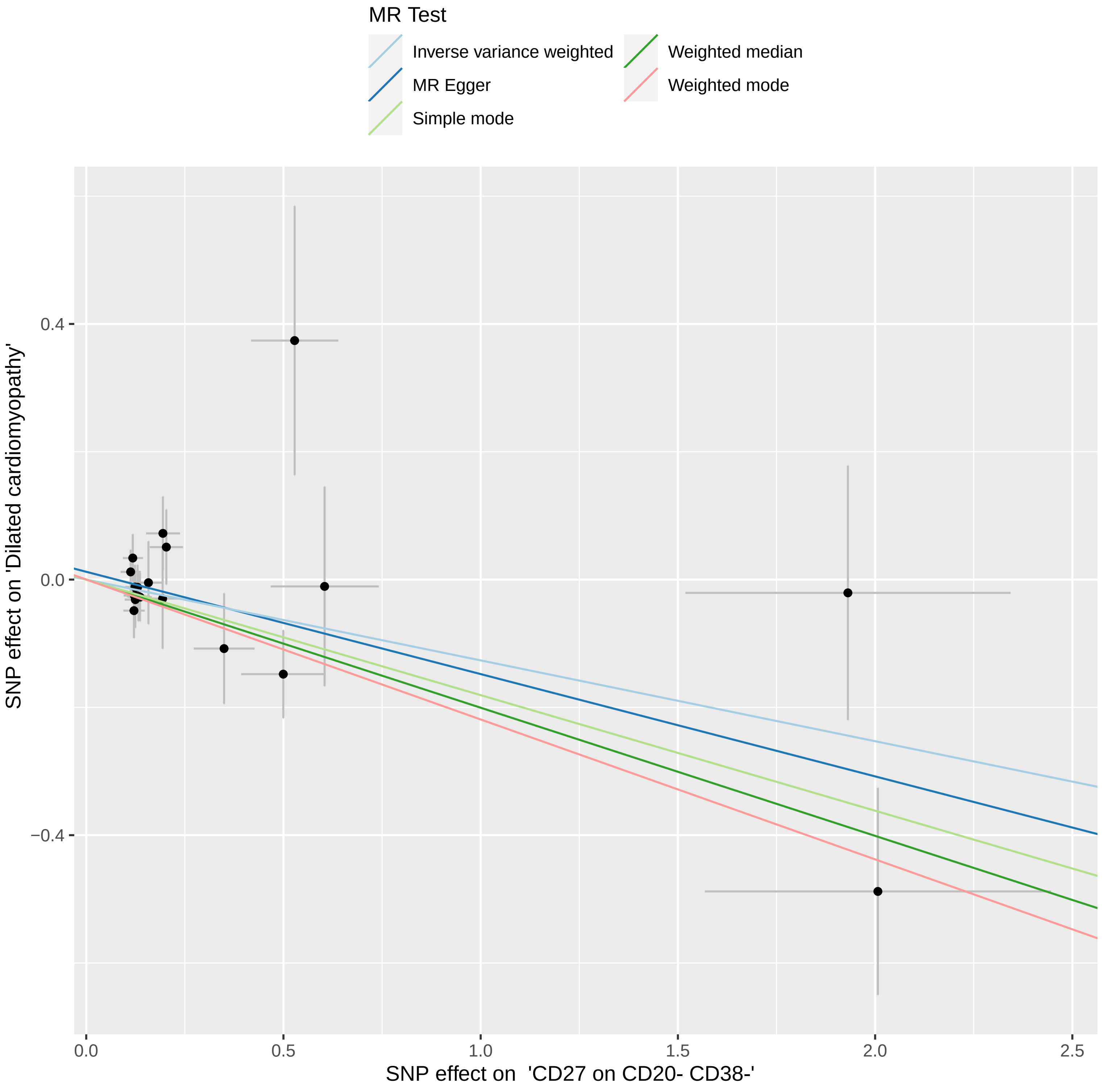
MR Test
Inverse variance weighted
MR Egger
Simple mode
Weighted median
Weighted mode
SNP effect on 'Dilated cardiomyopathy'
0.4
0.0
−0.4
0.0
0.5
1.0
1.5
2.0
2.5
SNP effect on 'CD27 on CD20- CD38-'

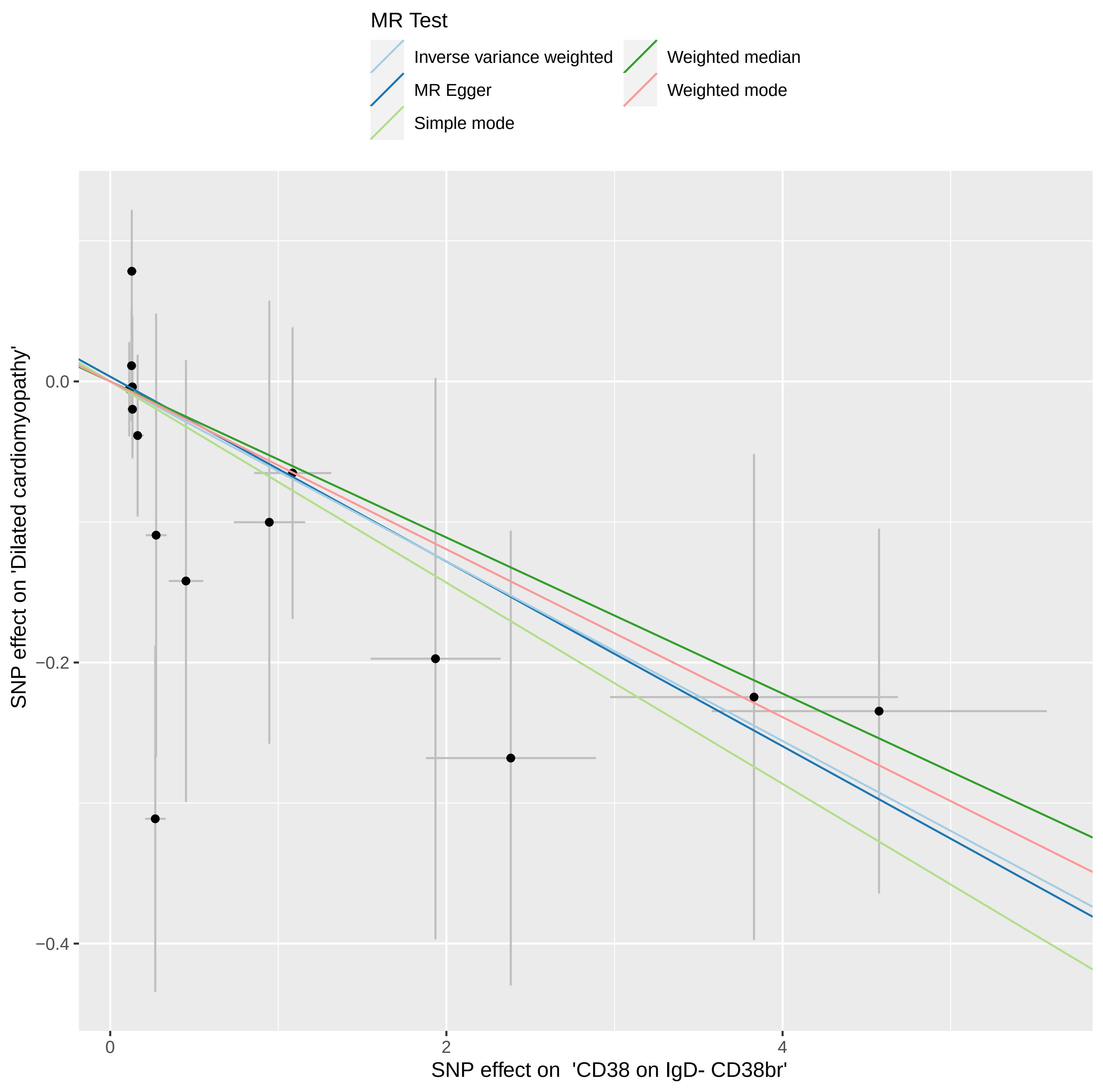

MR Test
Inverse variance weighted
MR Egger
Simple mode
Weighted median
Weighted mode
0.0
−0.2
−0.4
0
2
4
SNP effect on 'Dilated cardiomyopathy'
SNP effect on 'CD38 on IgD- CD38br'

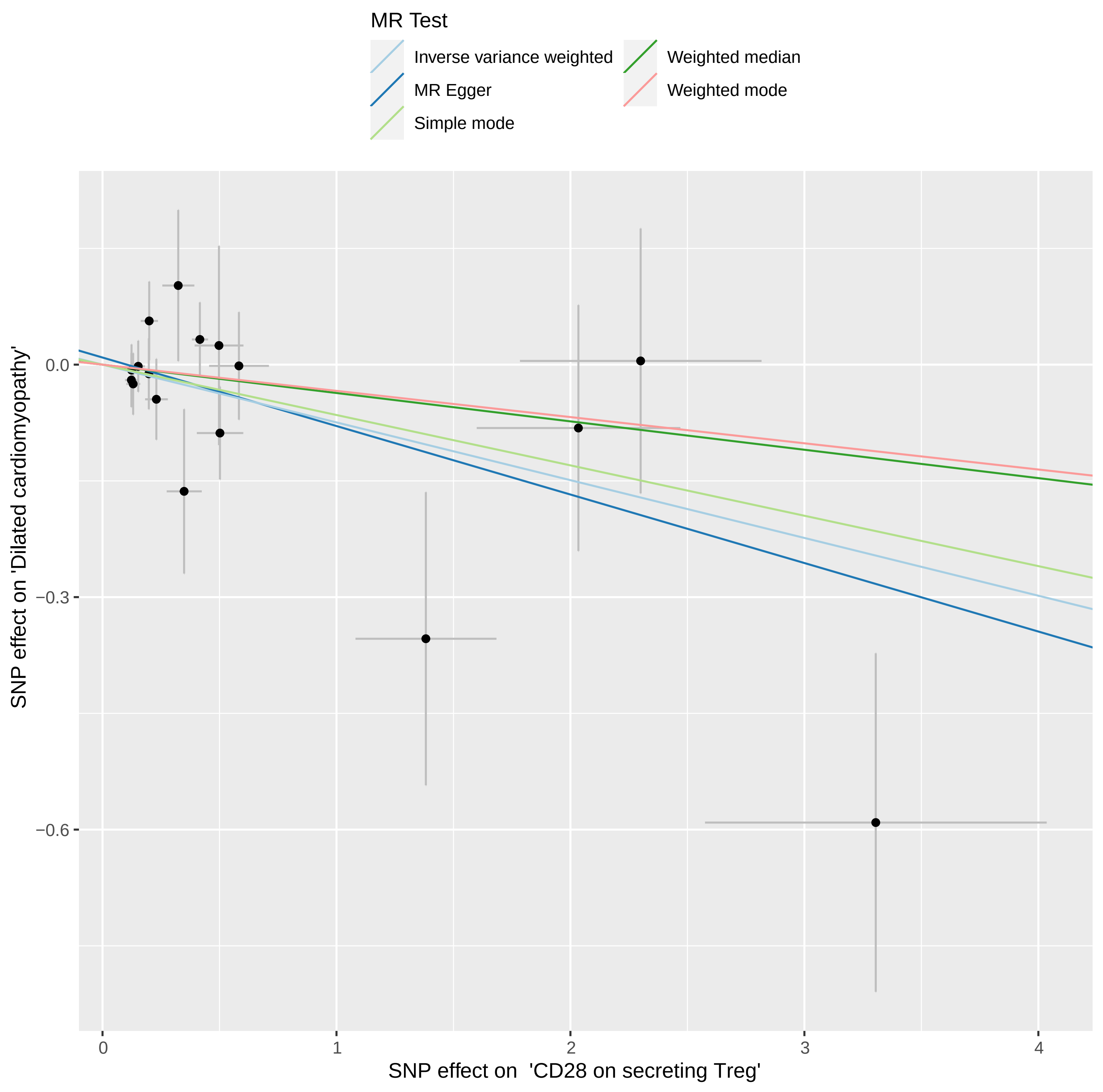
MR Test
Inverse variance weighted
MR Egger
Simple mode
Weighted median
Weighted mode
0.0
−0.3
−0.6
0
1
2
3
4
SNP effect on 'Dilated cardiomyopathy'
SNP effect on 'CD28 on secreting Treg'

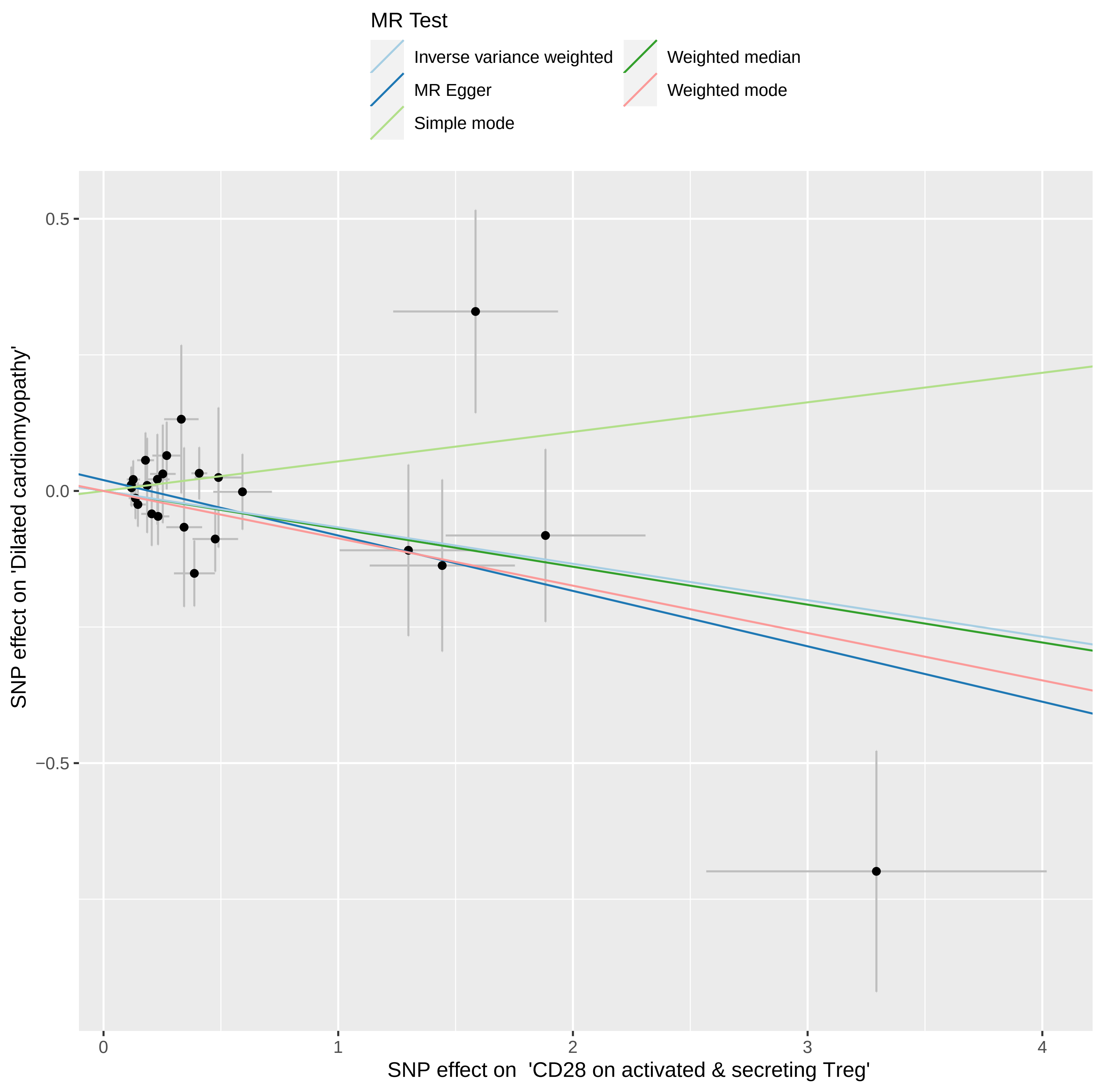

MR Test
Inverse variance weighted
MR Egger
Simple mode
Weighted median
Weighted mode
0.5
0.0
−0.5
0
1
2
3
4
SNP effect on 'Dilated cardiomyopathy'
SNP effect on 'CD28 on activated & secreting Treg'

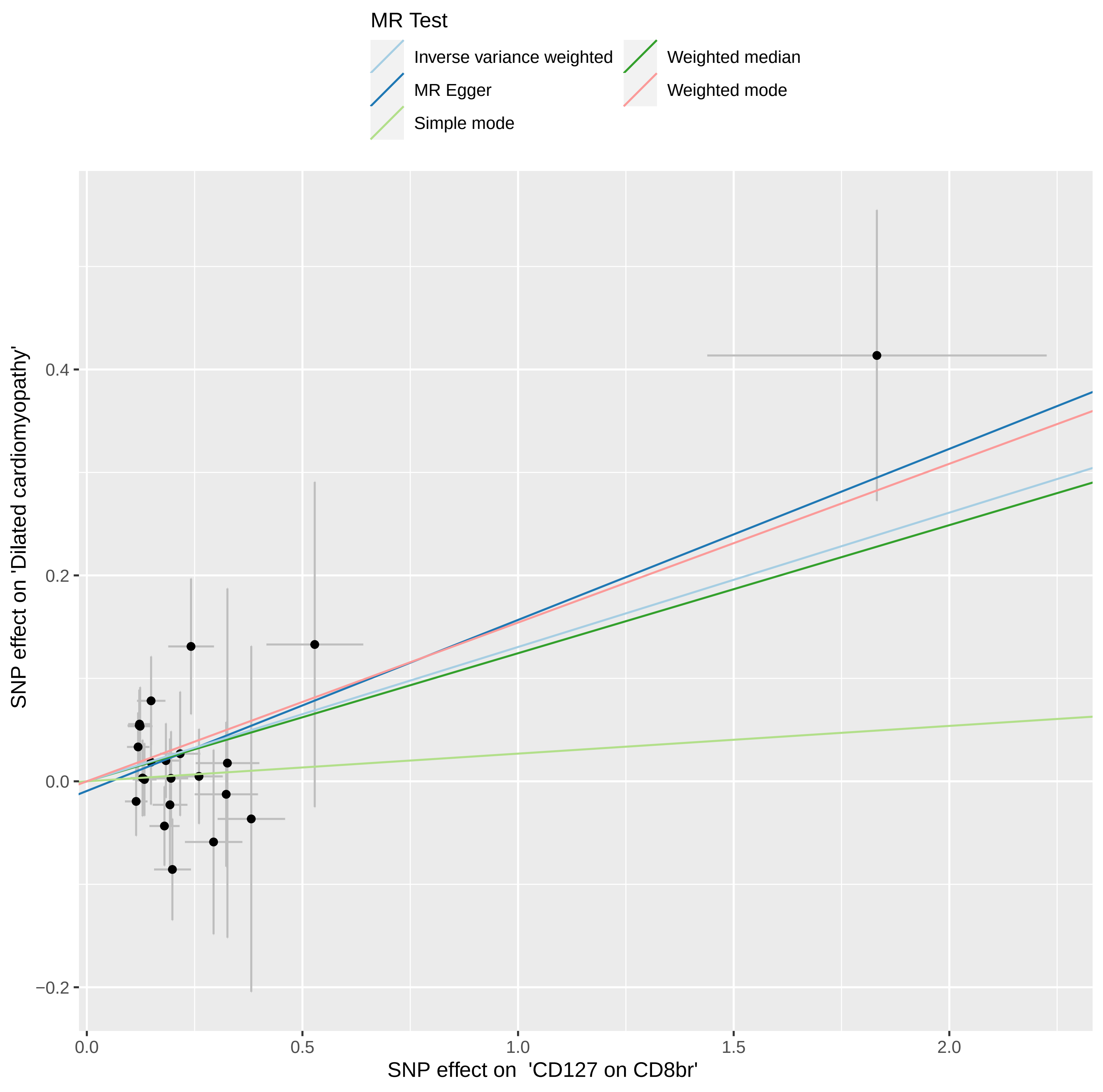

MR Test
Inverse variance weighted
MR Egger
Simple mode
Weighted median
Weighted mode
SNP effect on 'Dilated cardiomyopathy'
0.4
0.2
0.0
−0.2
0.0
0.5
1.0
1.5
2.0
SNP effect on 'CD127 on CD8br'

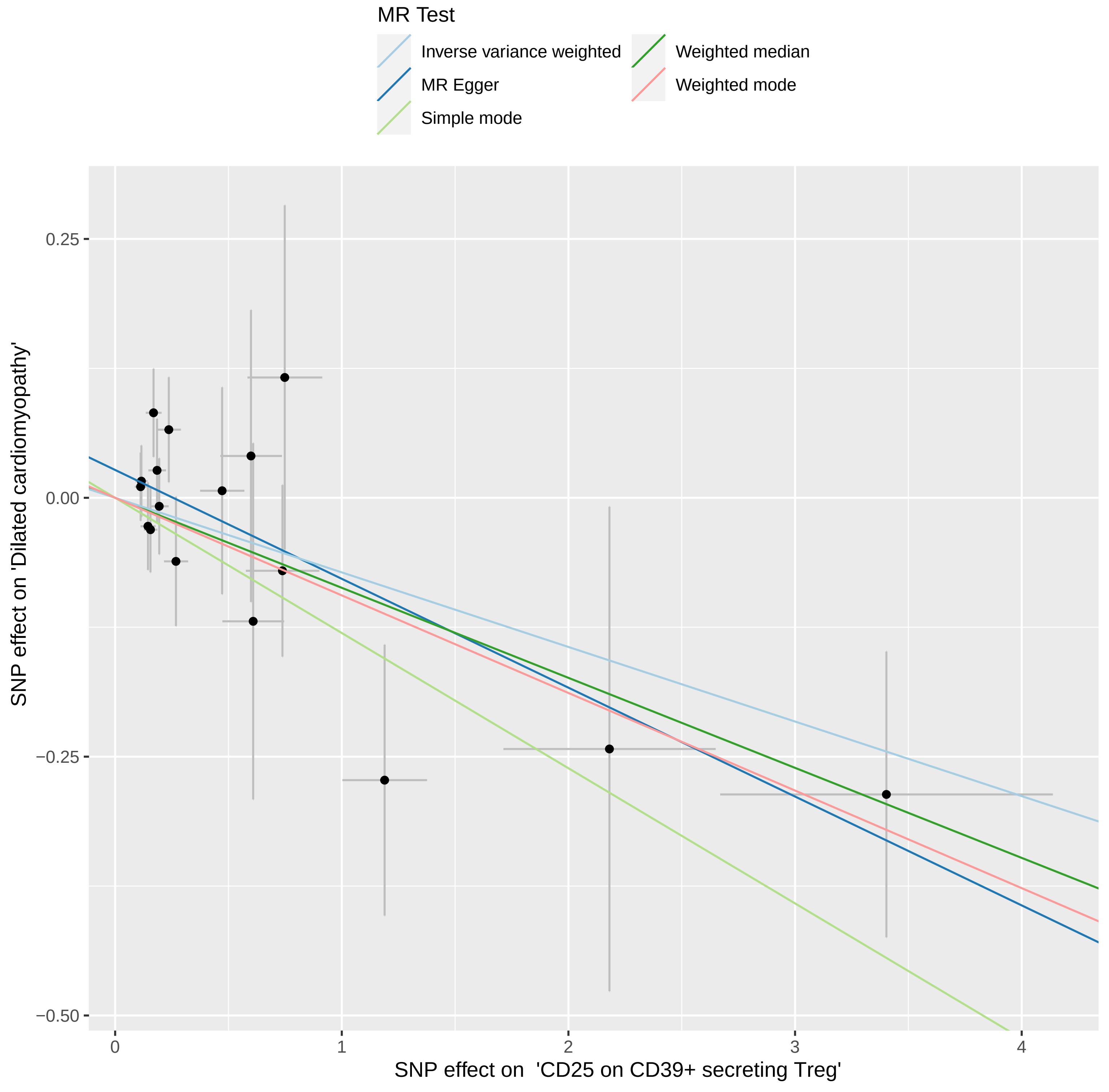
MR Test
Inverse variance weighted
MR Egger
Simple mode
Weighted median
Weighted mode
0.25
0.00
−0.25
−0.50
0
1
2
3
4
SNP effect on 'Dilated cardiomyopathy'
SNP effect on 'CD25 on CD39+ secreting Treg'

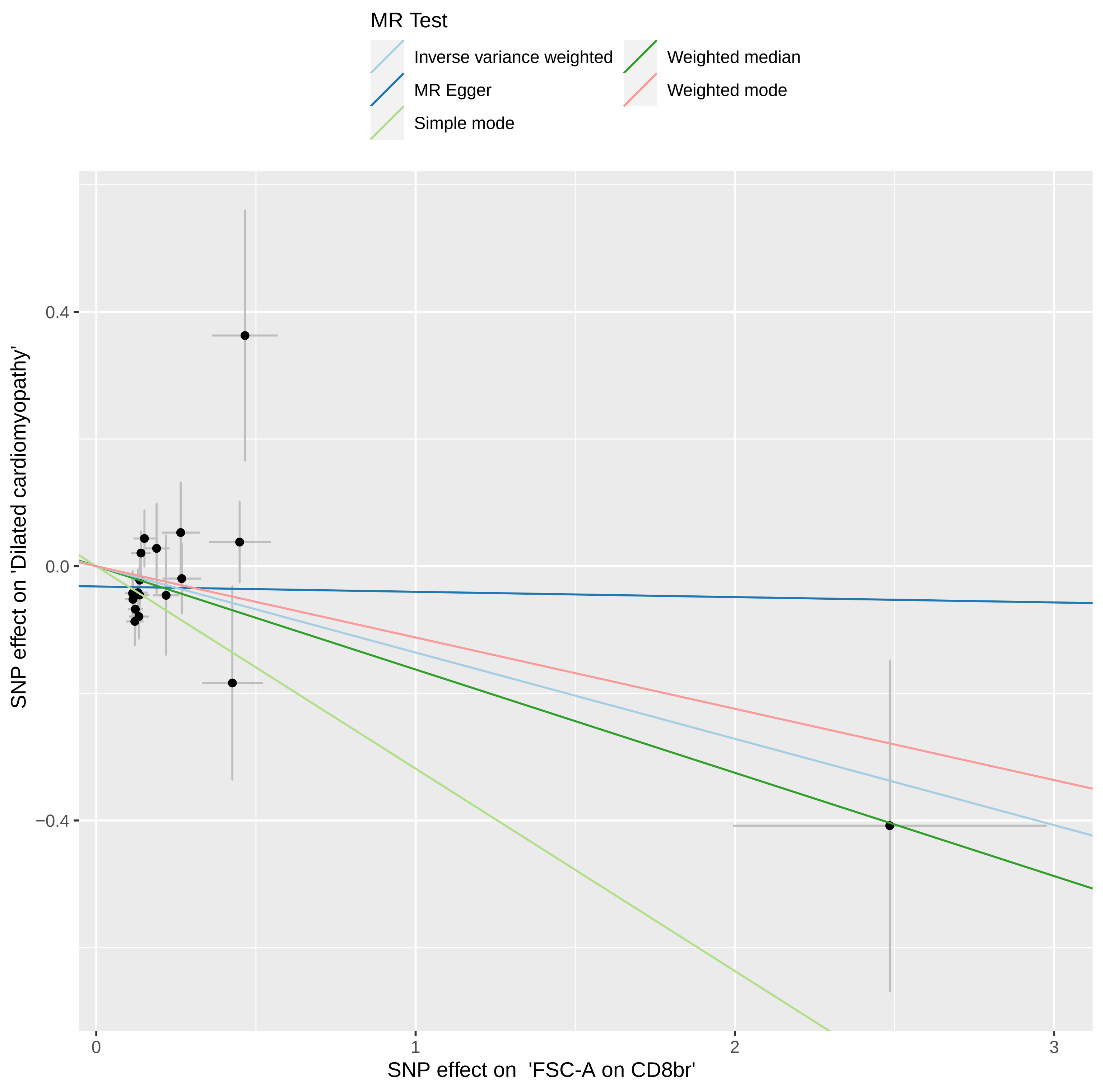
MR Test
Inverse variance weighted
MR Egger
Simple mode
Weighted median
Weighted mode
0.4
0.0
−0.4
0
1
2
3
SNP effect on 'Dilated cardiomyopathy'
SNP effect on 'FSC-A on CD8br'

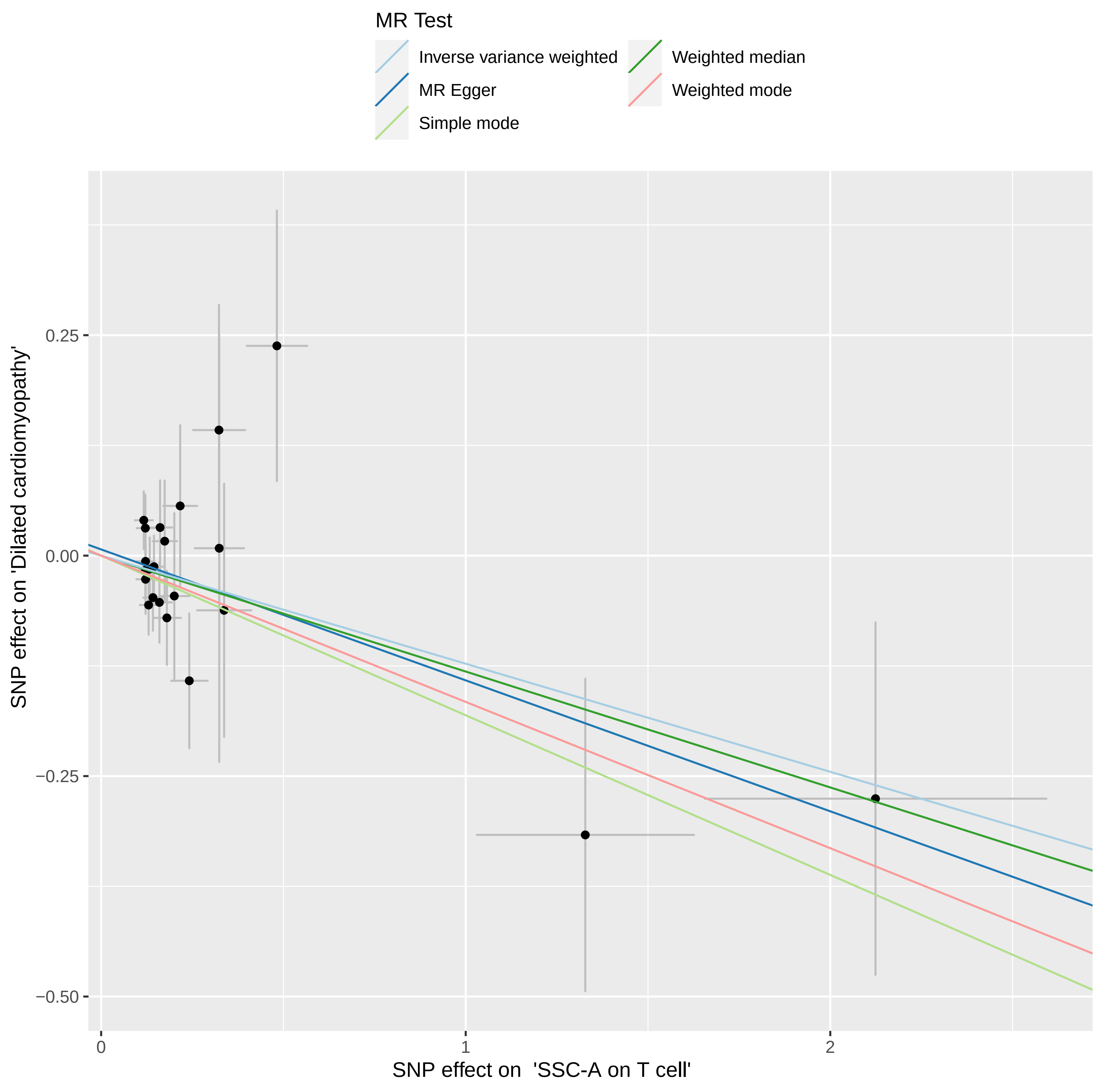
MR Test
Inverse variance weighted
MR Egger
Simple mode
Weighted median
Weighted mode
SNP effect on 'Dilated cardiomyopathy'
0.25
0.00
−0.25
−0.50
0
1
2
SNP effect on 'SSC-A on T cell'

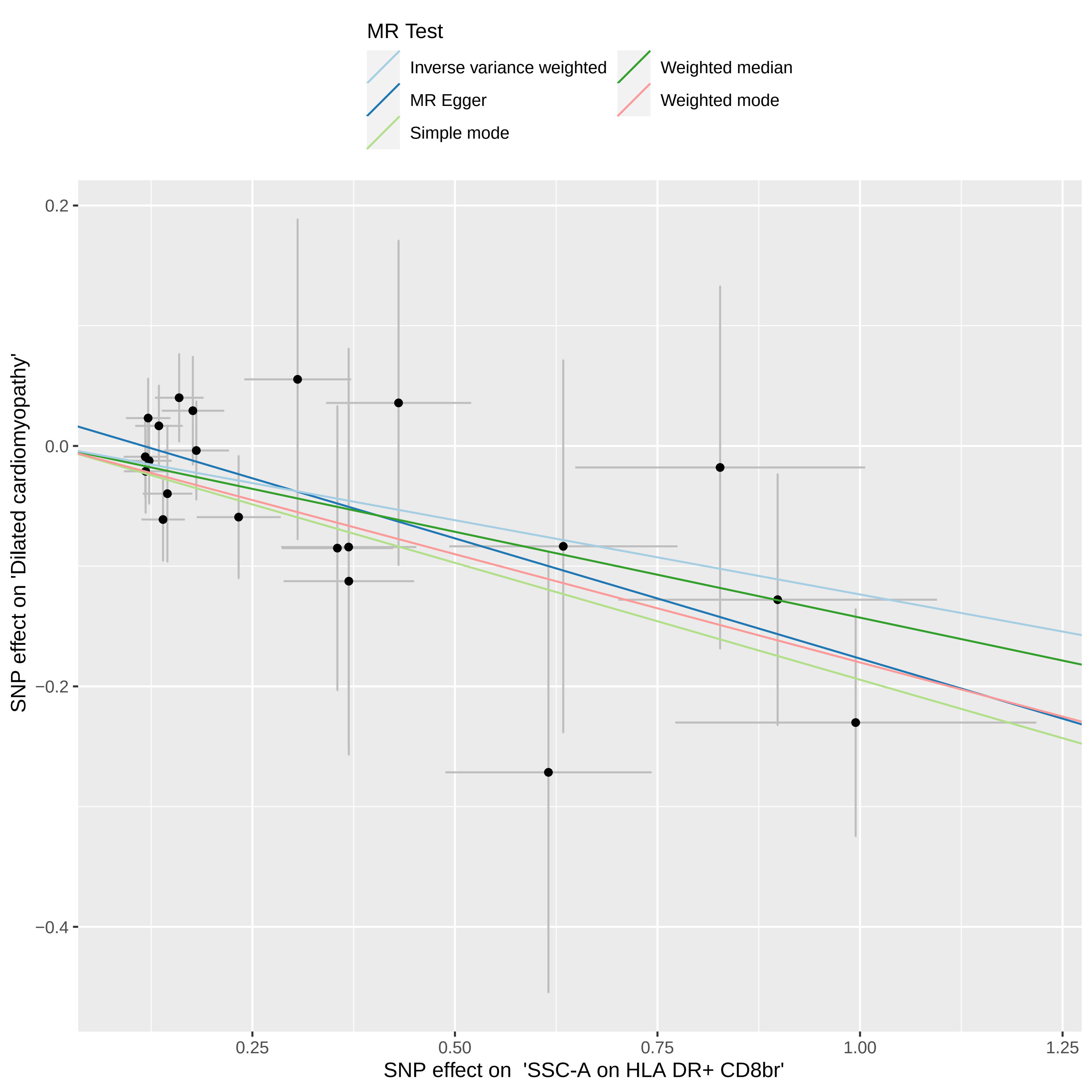
MR Test
Inverse variance weighted
MR Egger
Simple mode
Weighted median
Weighted mode
0.2
0.0
−0.2
−0.4
SNP effect on 'Dilated cardiomyopathy'
0.25
0.50
0.75
1.00
1.25
SNP effect on 'SSC-A on HLA DR+ CD8br'

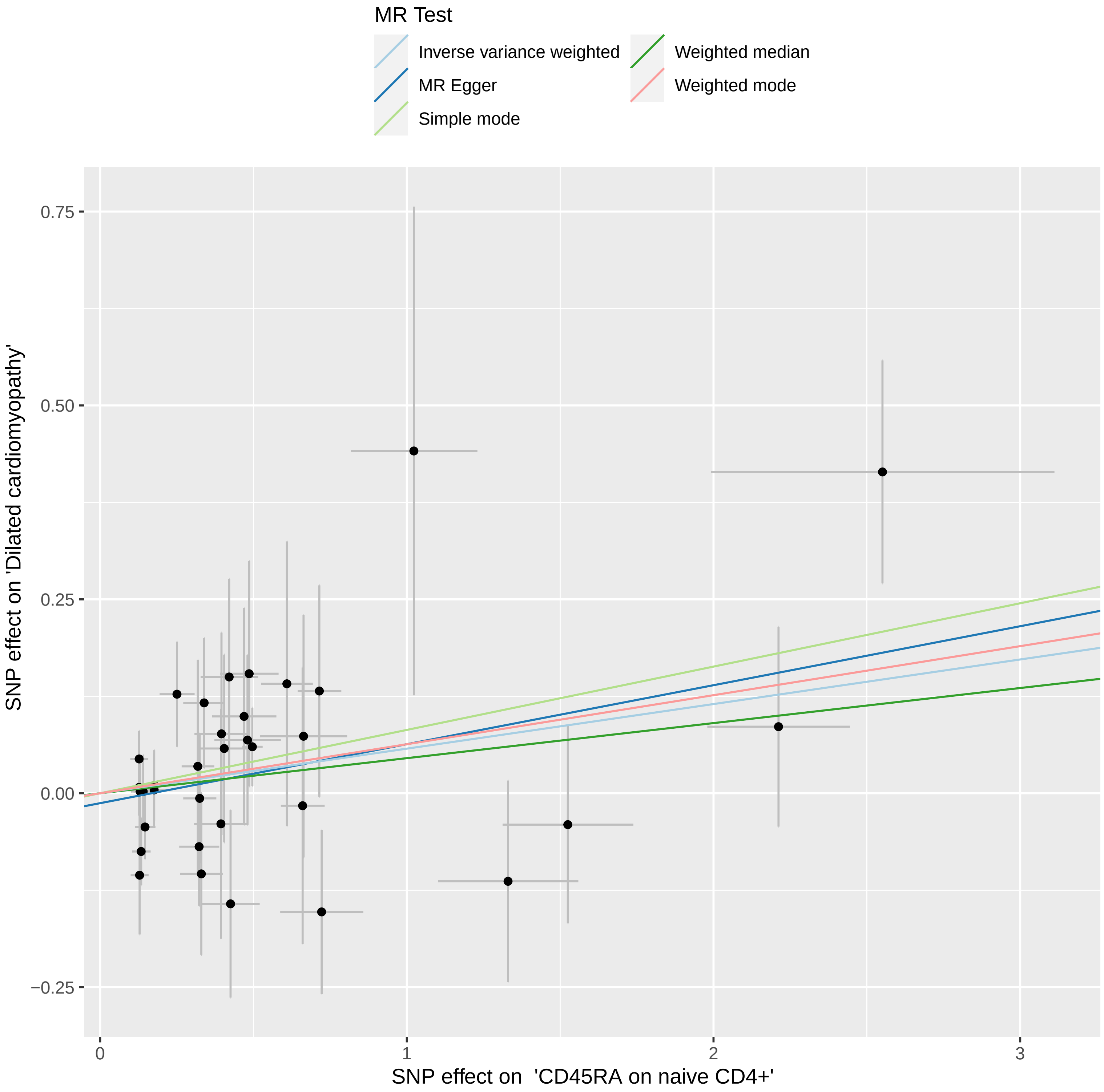

MR Test
Inverse variance weighted
MR Egger
Simple mode
Weighted median
Weighted mode
SNP effect on 'Dilated cardiomyopathy'
0.75
0.50
0.25
0.00
−0.25
0
1
2
3
SNP effect on 'CD45RA on naive CD4+'

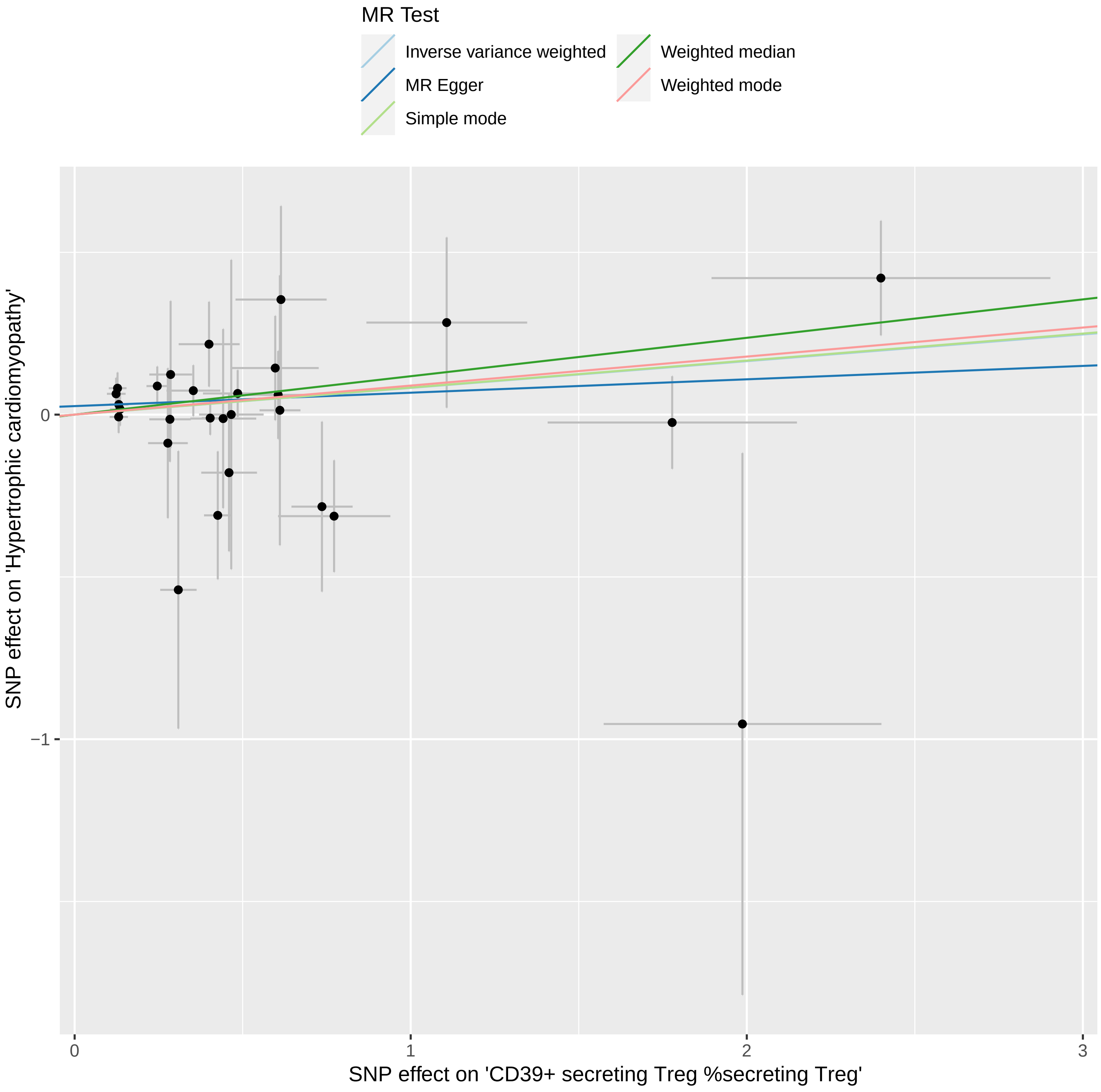
MR Test
Inverse variance weighted
MR Egger
Simple mode
Weighted median
Weighted mode
SNP effect on 'Hypertrophic cardiomyopathy'
0
−1
0
1
2
3
SNP effect on 'CD39+ secreting Treg %secreting Treg'

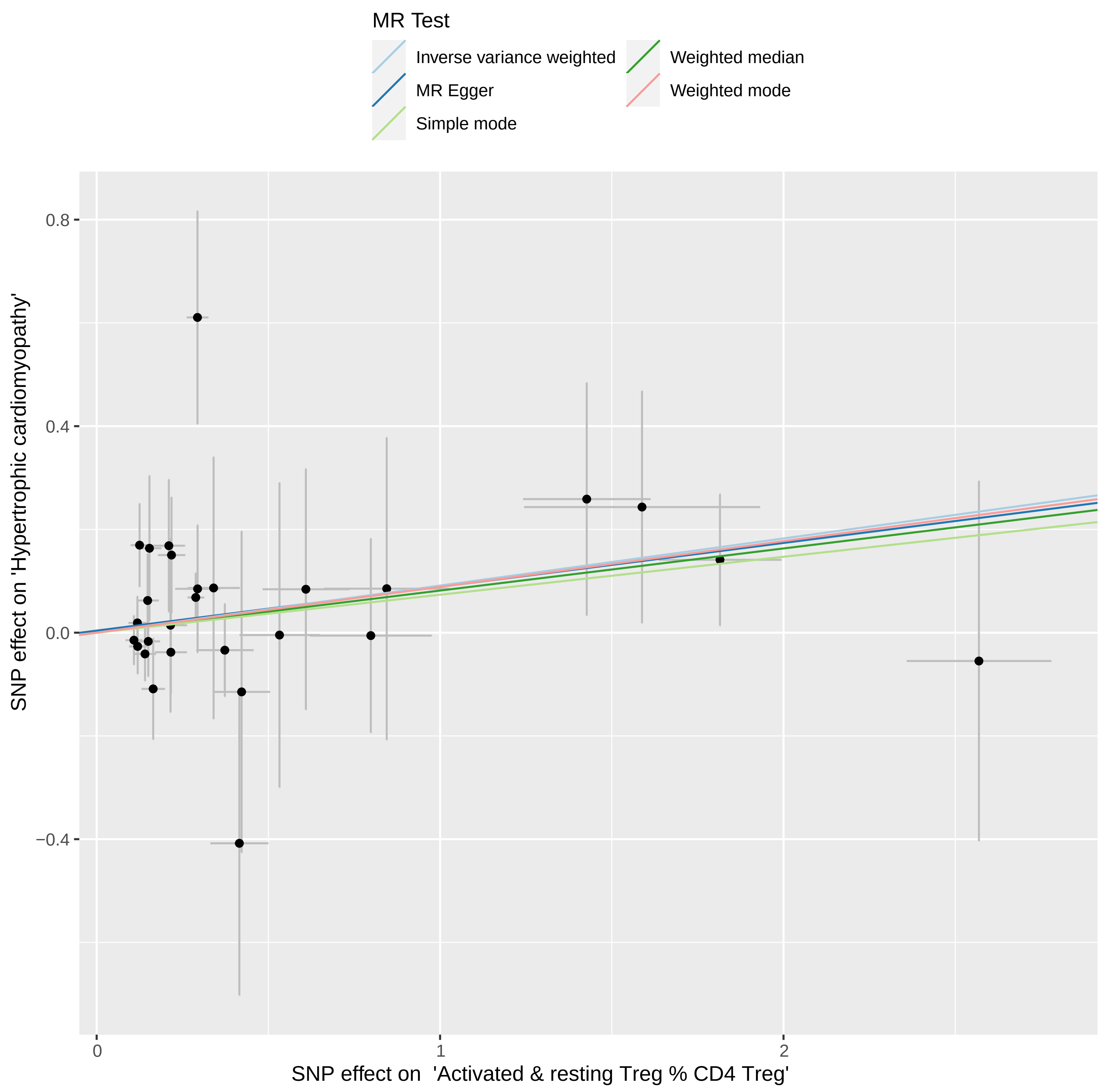
MR Test
Inverse variance weighted
MR Egger
Simple mode
Weighted median
Weighted mode
0.8
0.4
0.0
−0.4
0
1
2
SNP effect on 'Hypertrophic cardiomyopathy'
SNP effect on 'Activated & resting Treg % CD4 Treg'

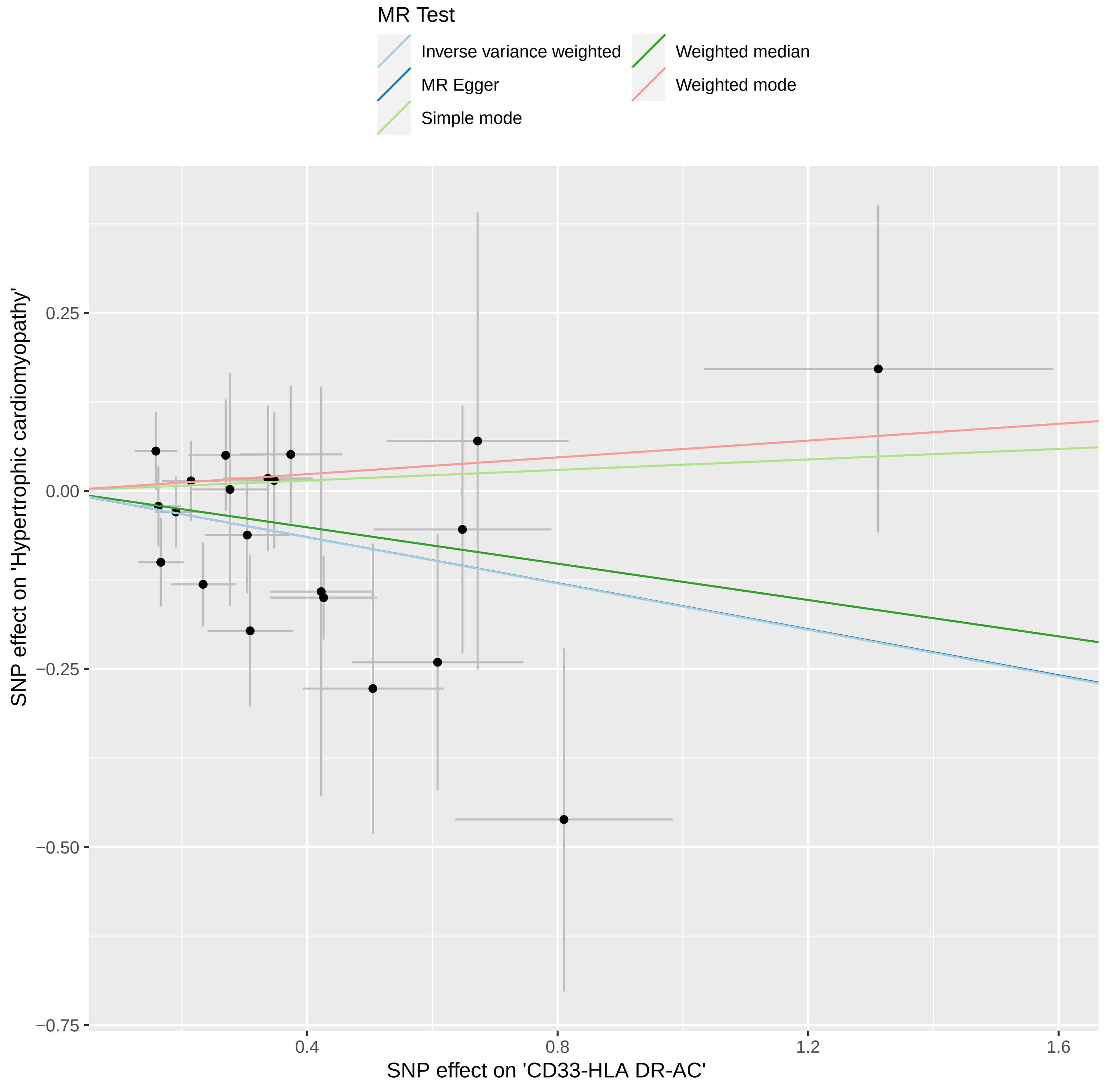
MR Test
Inverse variance weighted
MR Egger
Simple mode
Weighted median
Weighted mode
SNP effect on 'Hypertrophic cardiomyopathy'
0.25
0.00
−0.25
−0.50
−0.75
0.4
0.8
1.2
1.6
SNP effect on 'CD33-HLA DR-AC'

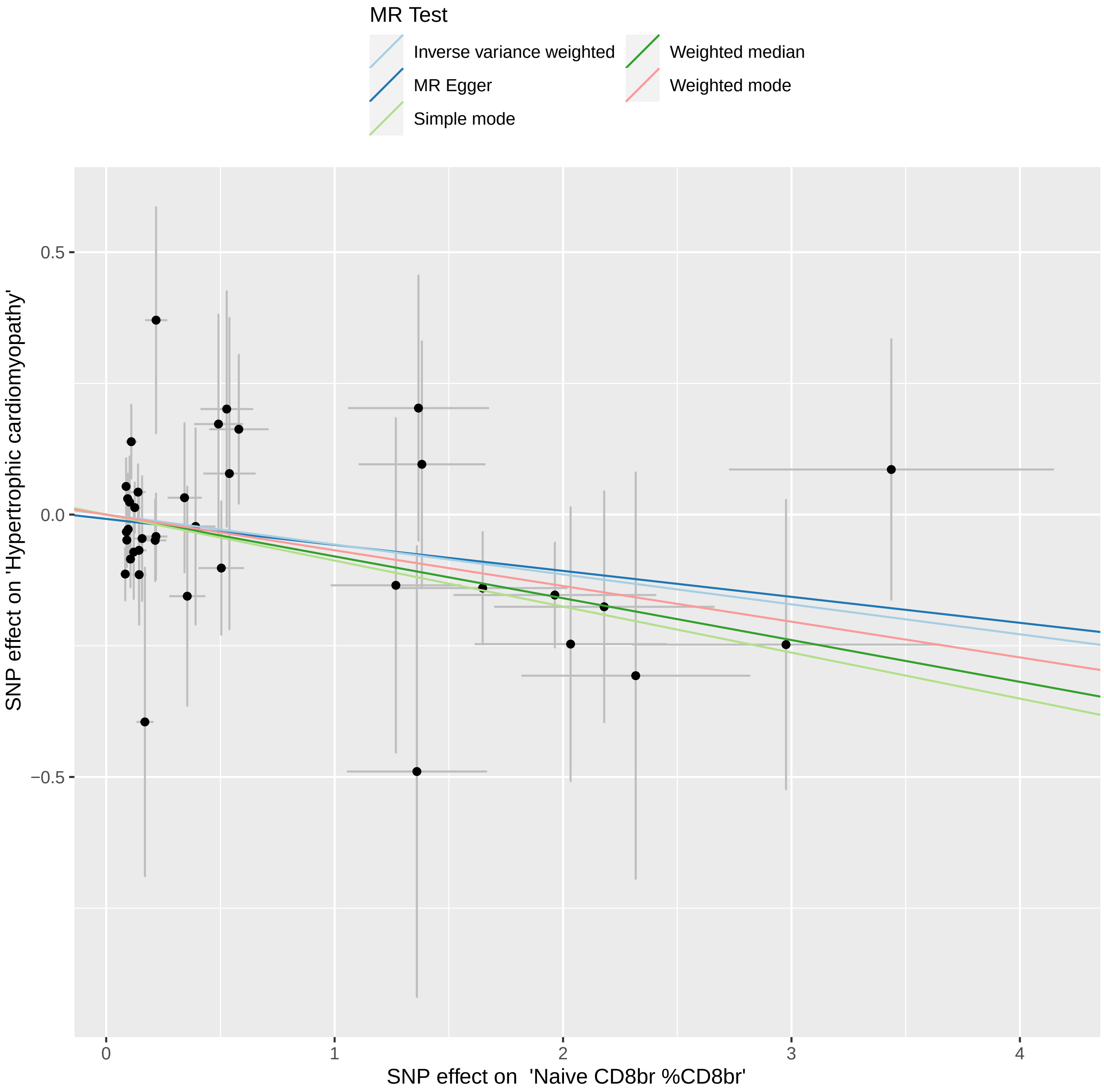
MR Test
Inverse variance weighted
MR Egger
Simple mode
Weighted median
Weighted mode
0.5
0.0
−0.5
0
1
2
3
4
SNP effect on 'Hypertrophic cardiomyopathy'
SNP effect on 'Naive CD8br %CD8br'

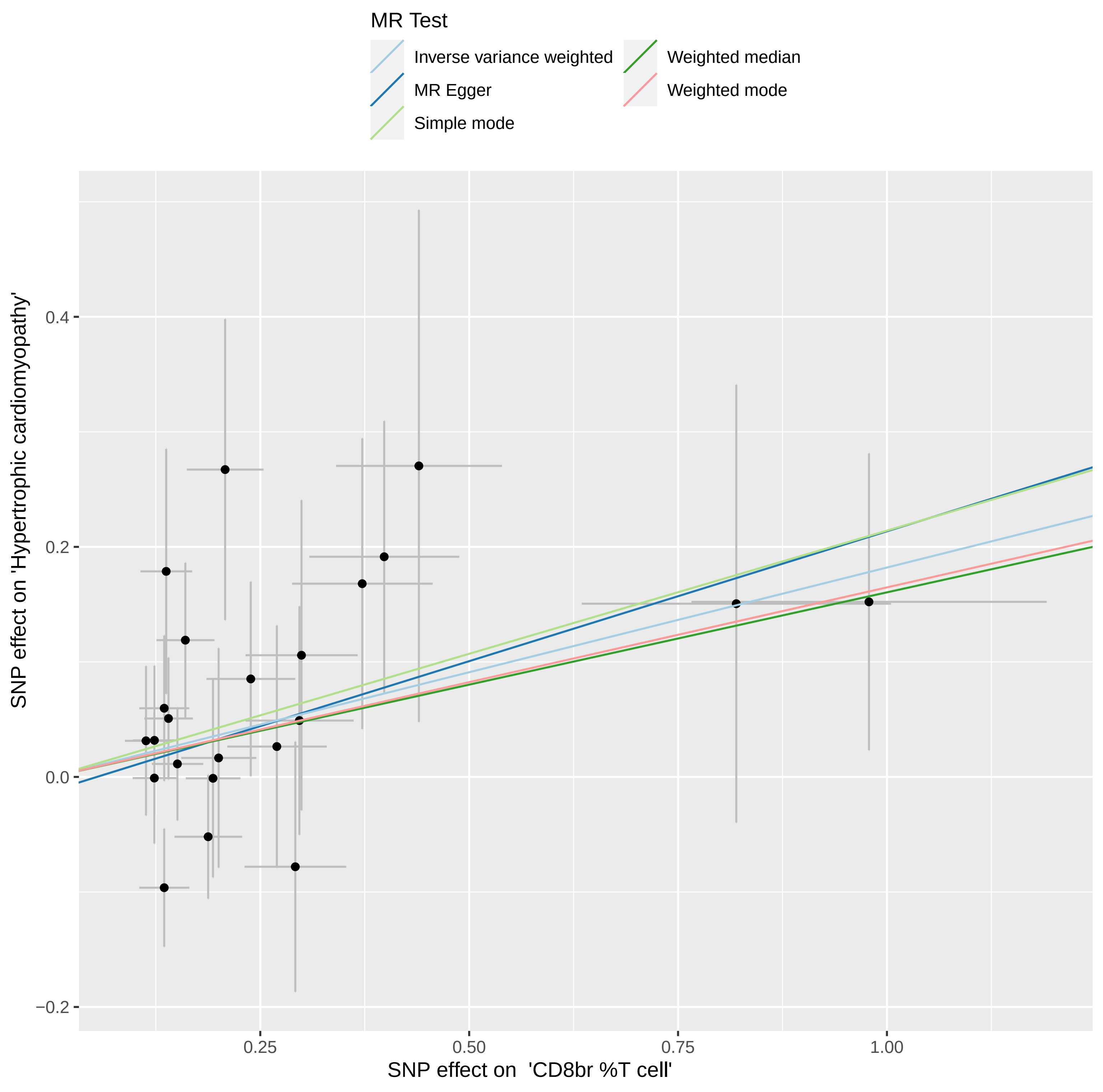

MR Test
Inverse variance weighted
MR Egger
Simple mode
Weighted median
Weighted mode
SNP effect on 'Hypertrophic cardiomyopathy'
0.4
0.2
0.0
−0.2
0.25
0.50
0.75
1.00
SNP effect on 'CD8br %T cell'

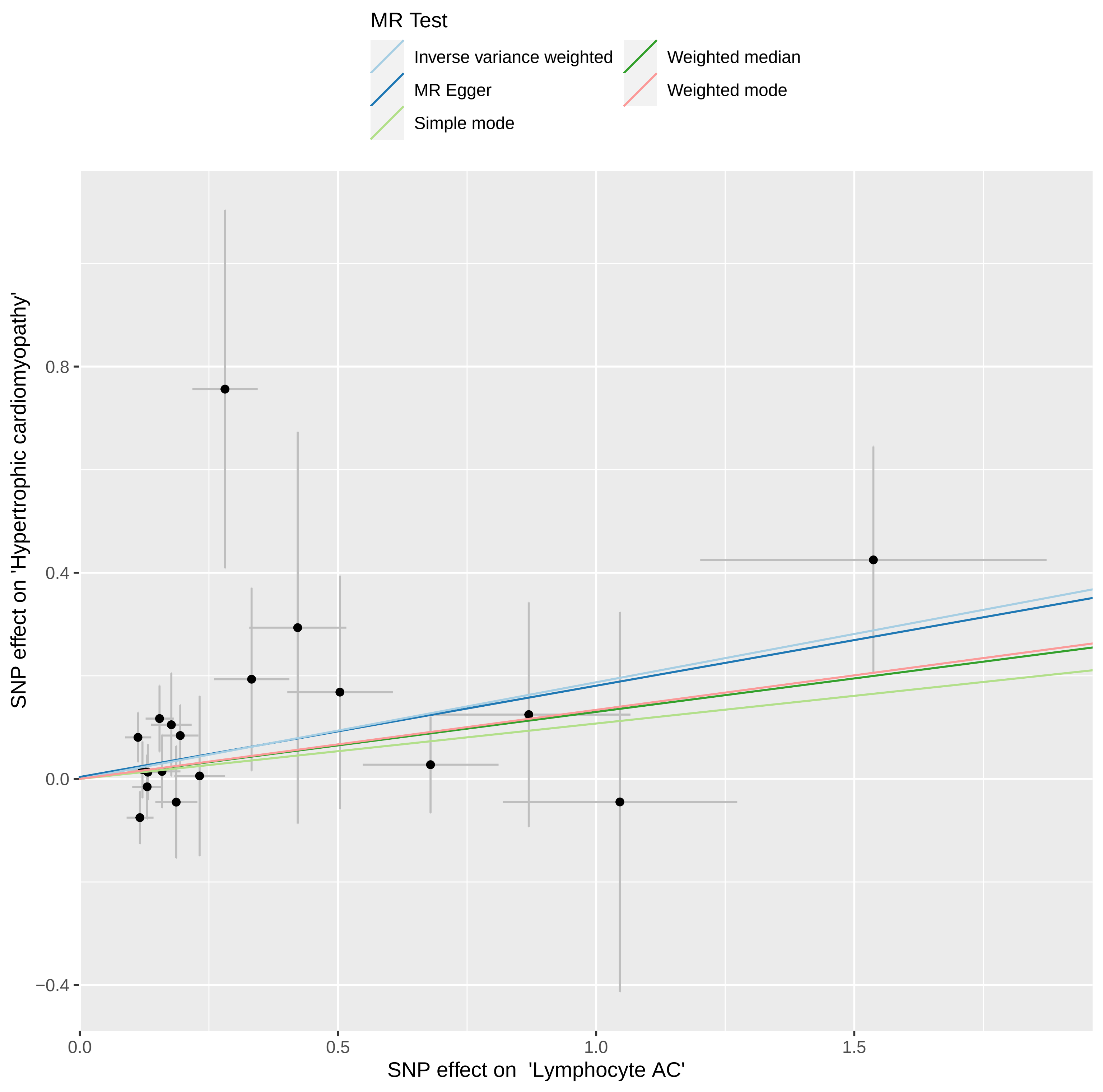
MR Test
Inverse variance weighted
MR Egger
Simple mode
Weighted median
Weighted mode
SNP effect on 'Hypertrophic cardiomyopathy'
0.8
0.4
0.0
−0.4
0.0
0.5
1.0
1.5
SNP effect on 'Lymphocyte AC'

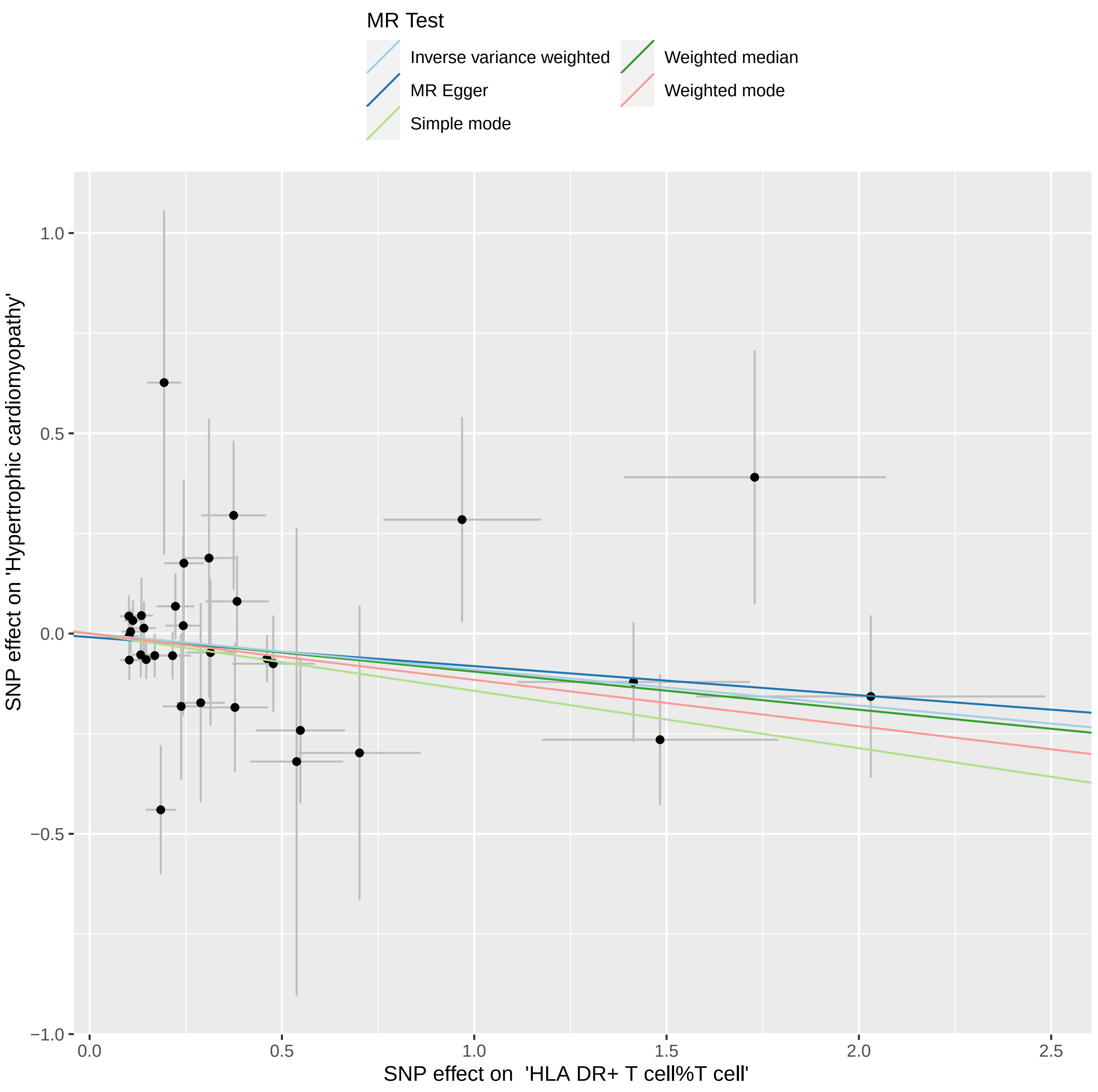
MR Test
Inverse variance weighted
MR Egger
Simple mode
Weighted median
Weighted mode
SNP effect on 'Hypertrophic cardiomyopathy'
1.0
0.5
0.0
−0.5
−1.0
0.0
0.5
1.0
1.5
2.0
2.5
SNP effect on 'HLA DR+ T cell%T cell'

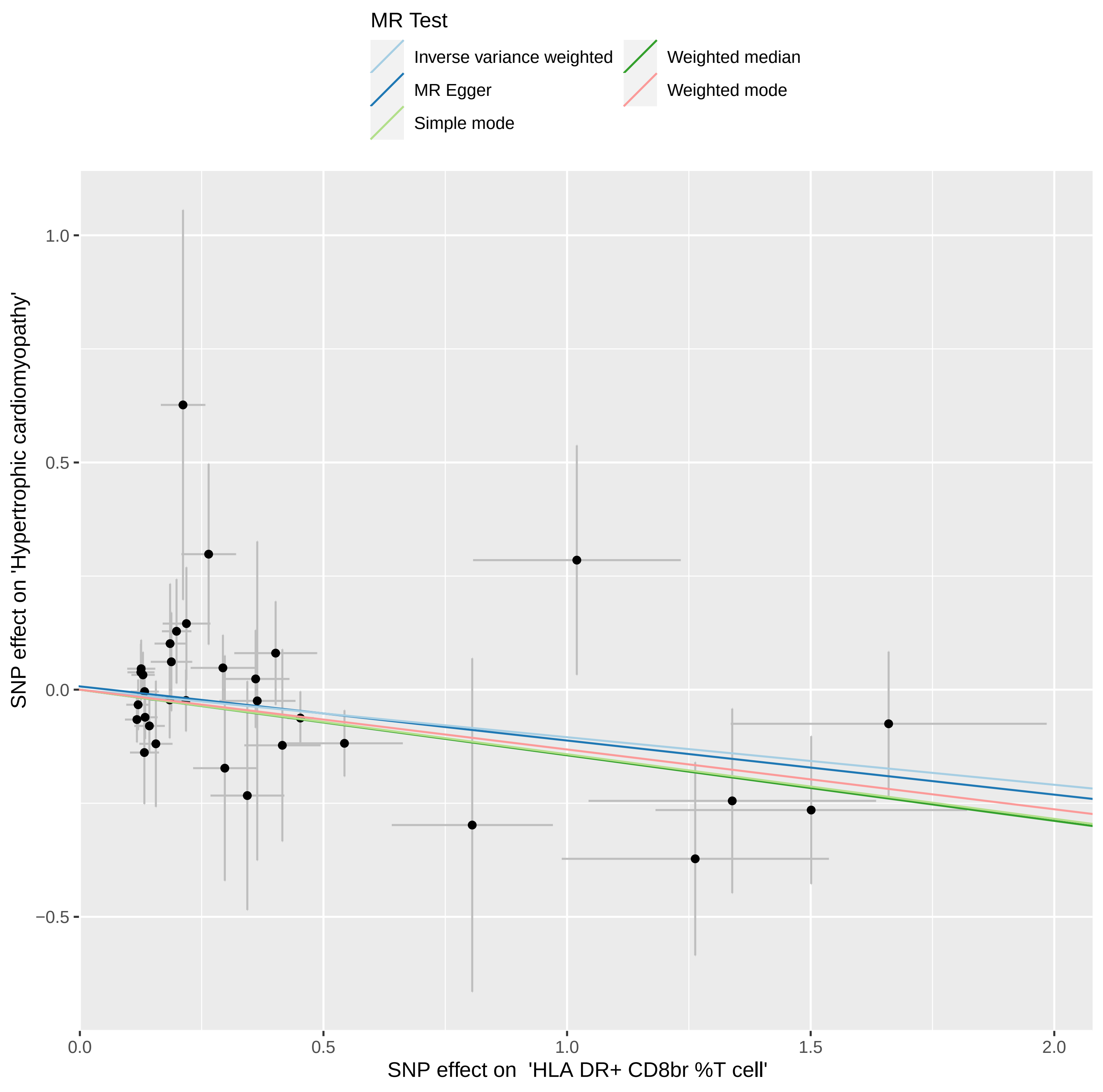

MR Test
Inverse variance weighted
MR Egger
Simple mode
Weighted median
Weighted mode
1.0
0.5
0.0
−0.5
0.0
0.5
1.0
1.5
2.0
SNP effect on 'Hypertrophic cardiomyopathy'
SNP effect on 'HLA DR+ CD8br %T cell'

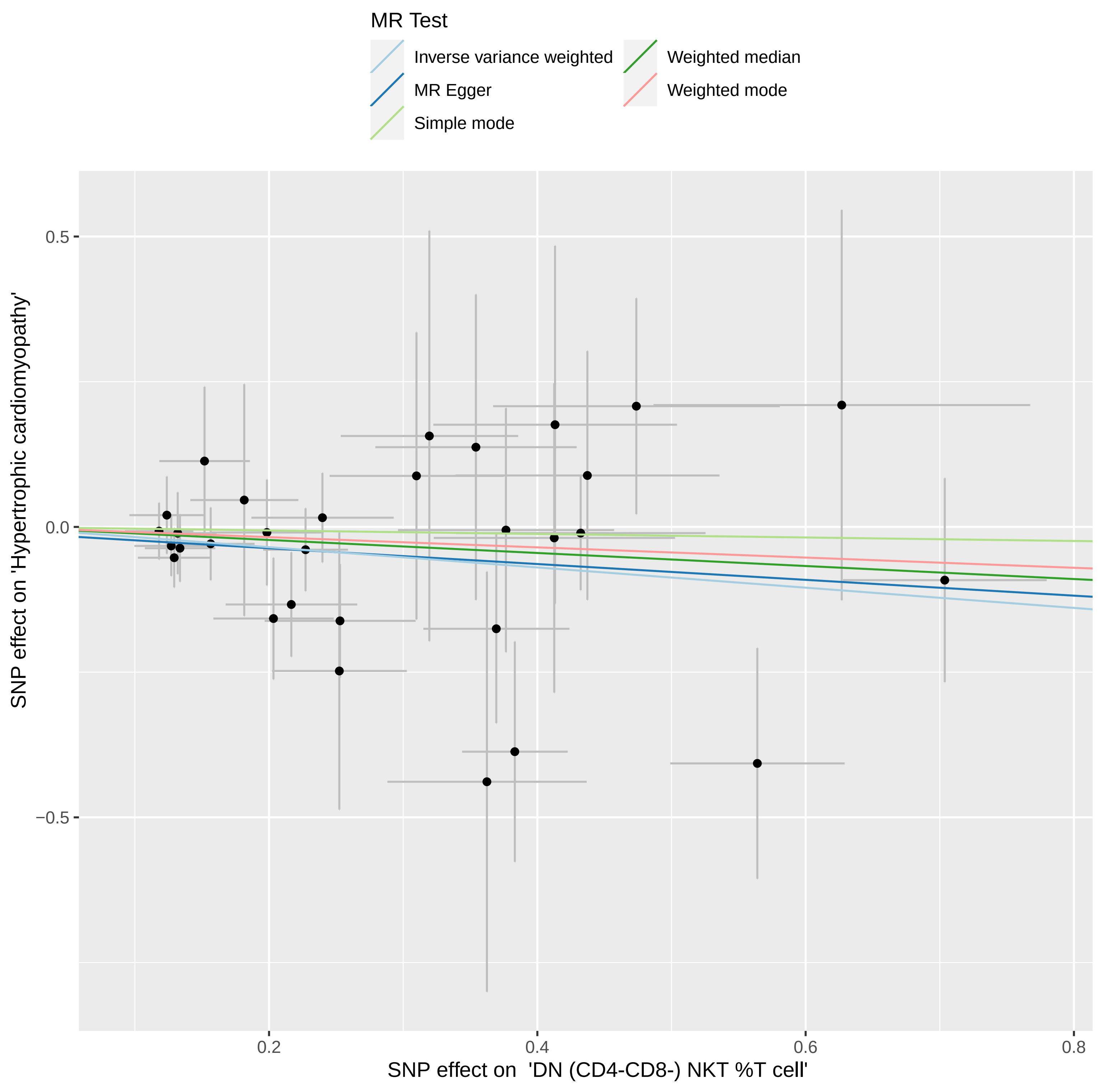
MR Test
Inverse variance weighted
MR Egger
Simple mode
Weighted median
Weighted mode
0.5
0.0
−0.5
SNP effect on 'Hypertrophic cardiomyopathy'
0.2
0.4
0.6
0.8
SNP effect on 'DN (CD4-CD8-) NKT %T cell'

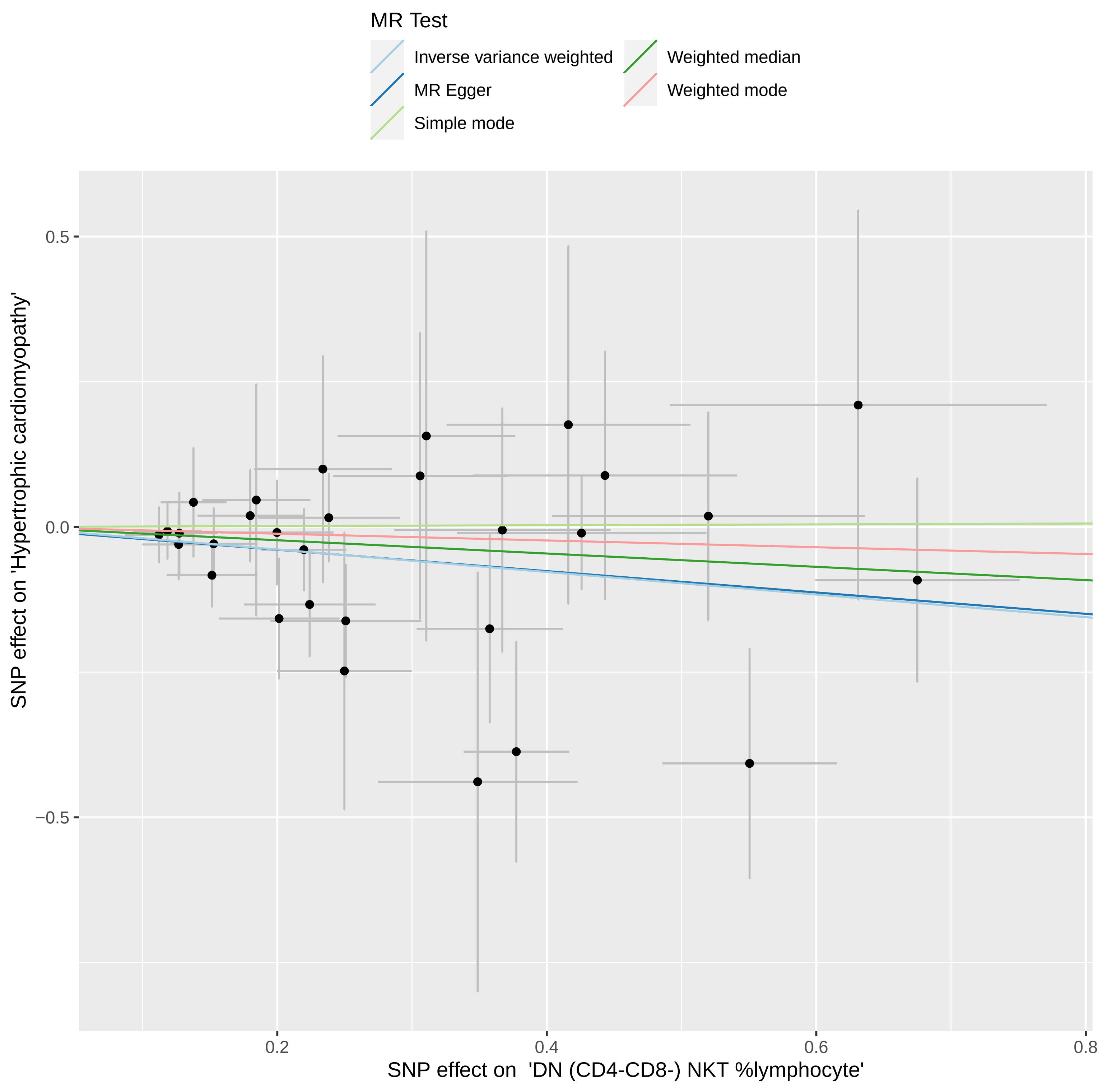

MR Test
Inverse variance weighted
MR Egger
Simple mode
Weighted median
Weighted mode
SNP effect on 'Hypertrophic cardiomyopathy'
0.5
0.0
−0.5
0.2
0.4
0.6
0.8
SNP effect on 'DN (CD4-CD8-) NKT %lymphocyte'

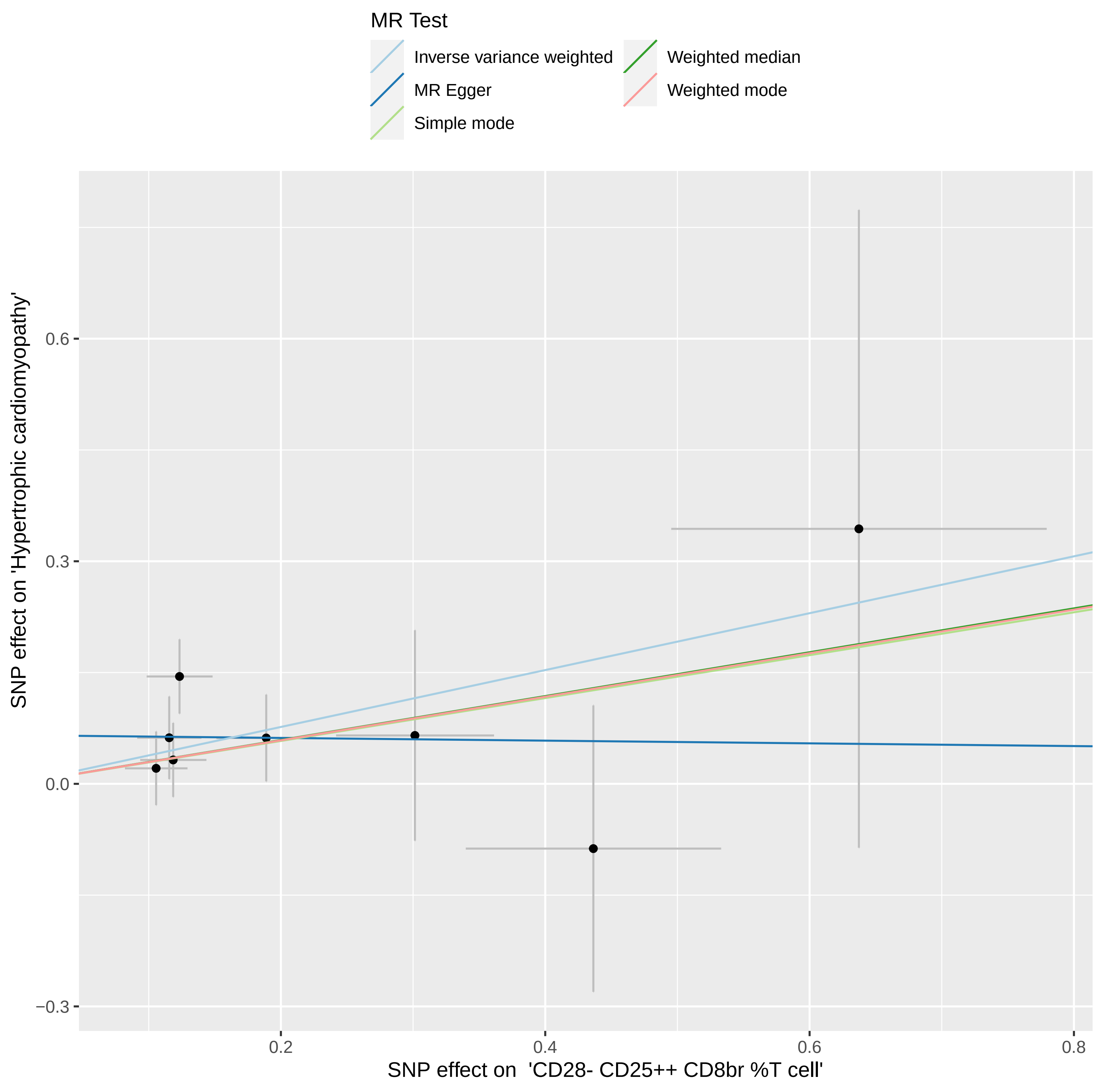
MR Test
Inverse variance weighted
MR Egger
Simple mode
Weighted median
Weighted mode
SNP effect on 'Hypertrophic cardiomyopathy'
0.6
0.3
0.0
−0.3
0.2
0.4
0.6
0.8
SNP effect on 'CD28- CD25++ CD8br %T cell'

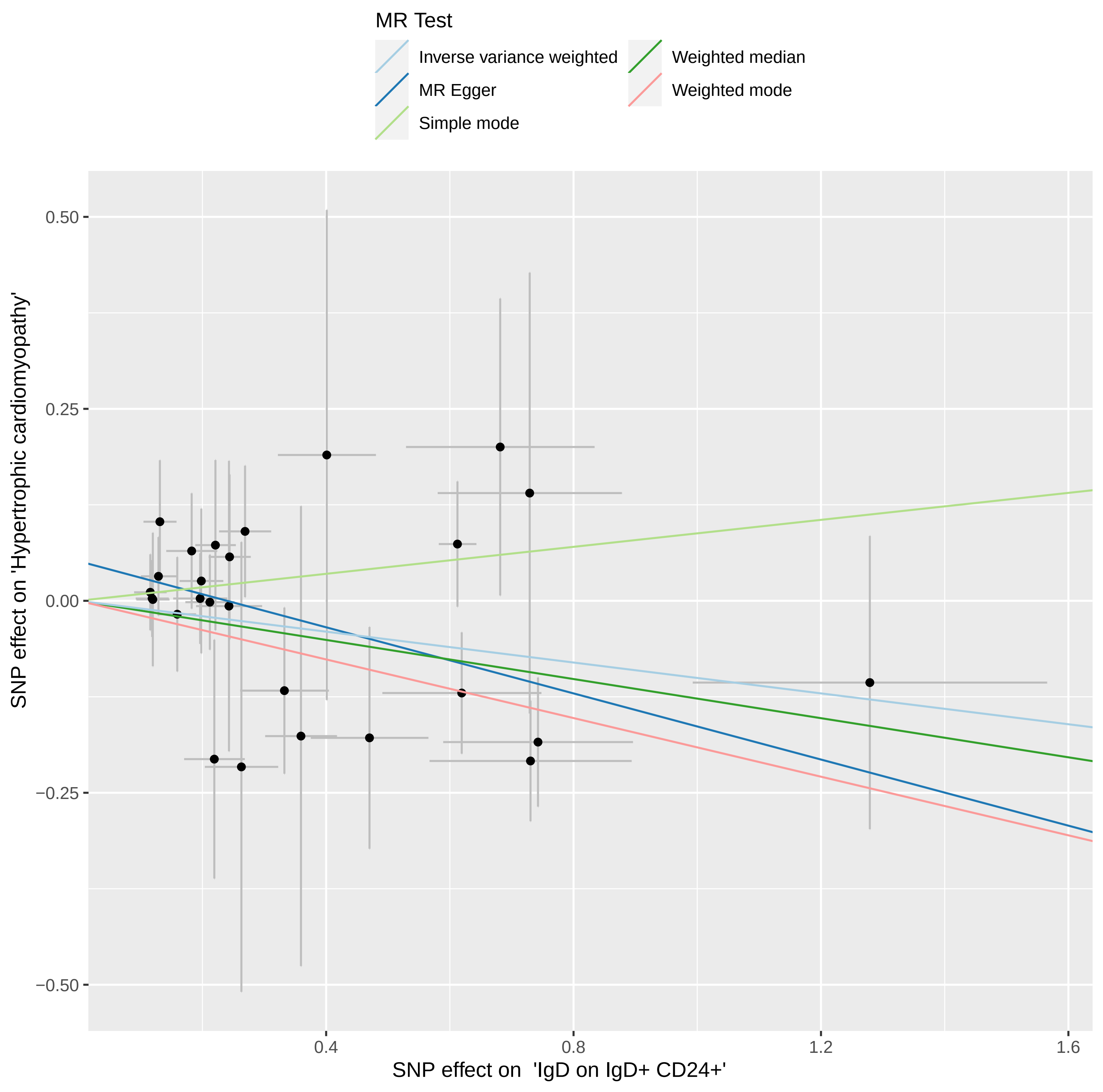

MR Test
Inverse variance weighted
MR Egger
Simple mode
Weighted median
Weighted mode
0.50
0.25
0.00
−0.25
−0.50
SNP effect on 'Hypertrophic cardiomyopathy'
0.4
0.8
1.2
1.6
SNP effect on 'IgD on IgD+ CD24+'

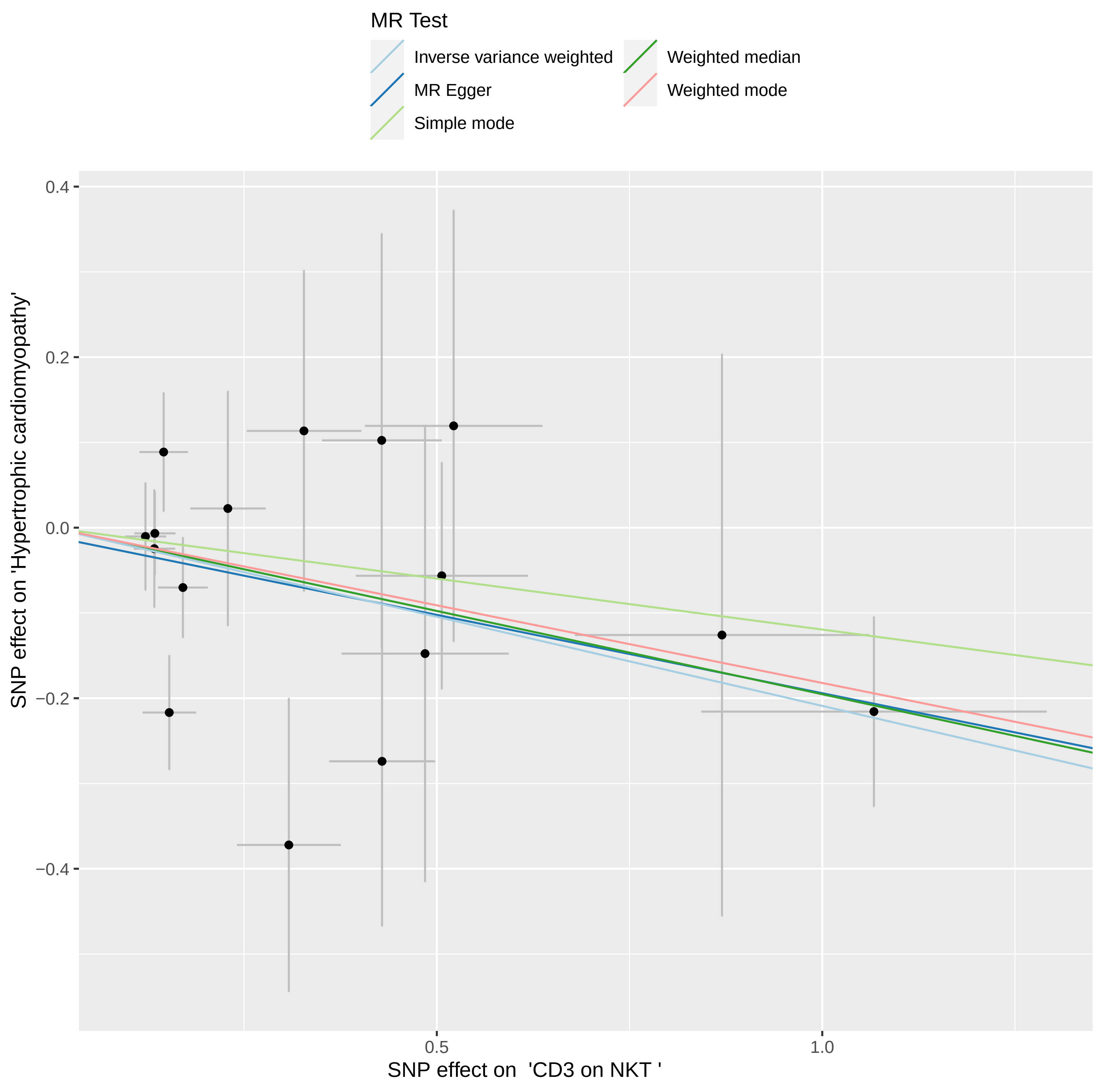
MR Test
Inverse variance weighted
MR Egger
Simple mode
Weighted median
Weighted mode
0.4
0.2
0.0
−0.2
−0.4
SNP effect on 'Hypertrophic cardiomyopathy'
0.5
1.0
SNP effect on 'CD3 on NKT '

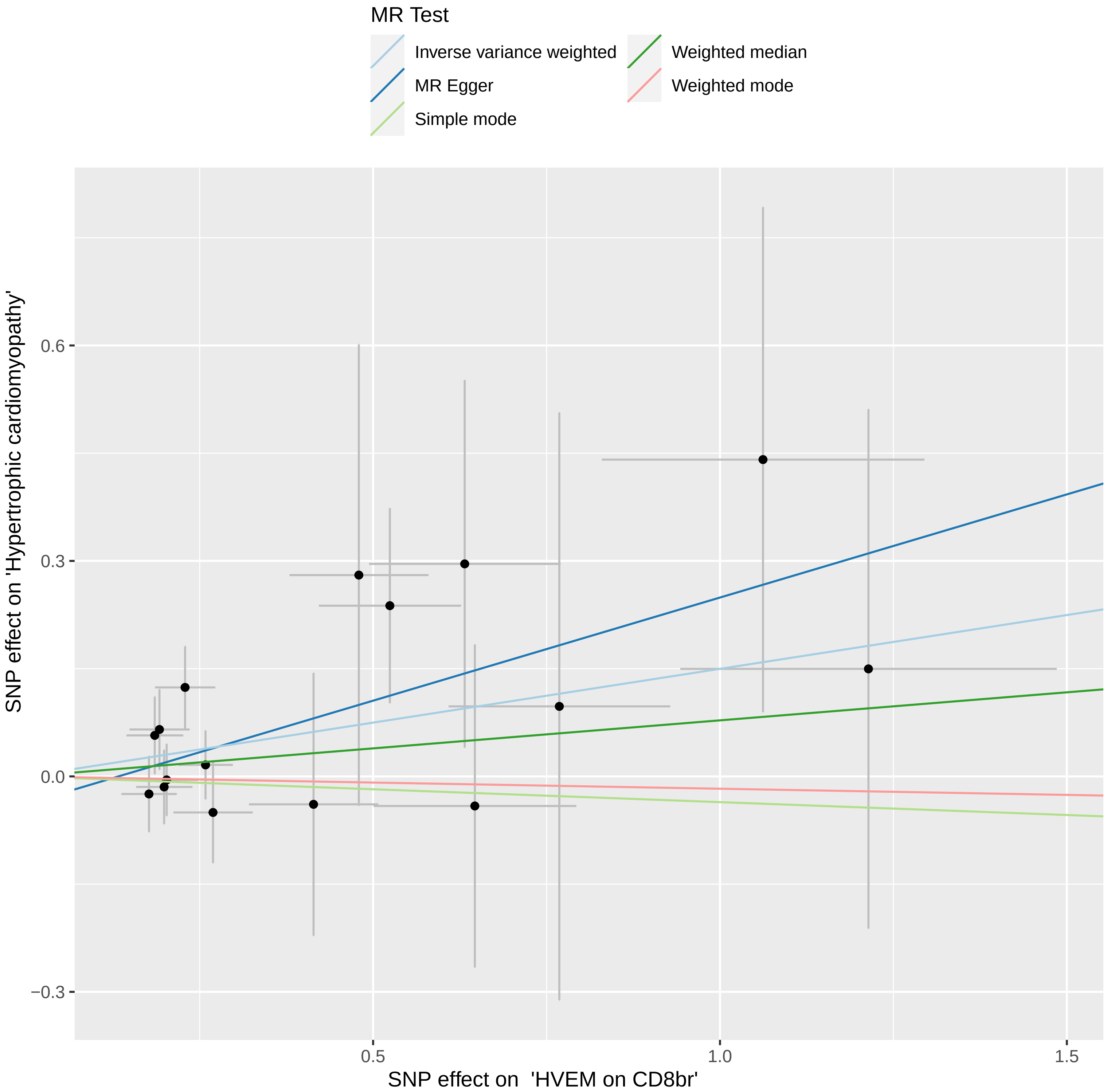

MR Test
Inverse variance weighted
MR Egger
Simple mode
Weighted median
Weighted mode
SNP effect on 'Hypertrophic cardiomyopathy'
0.6
0.3
0.0
−0.3
0.5
1.0
1.5
SNP effect on 'HVEM on CD8br'

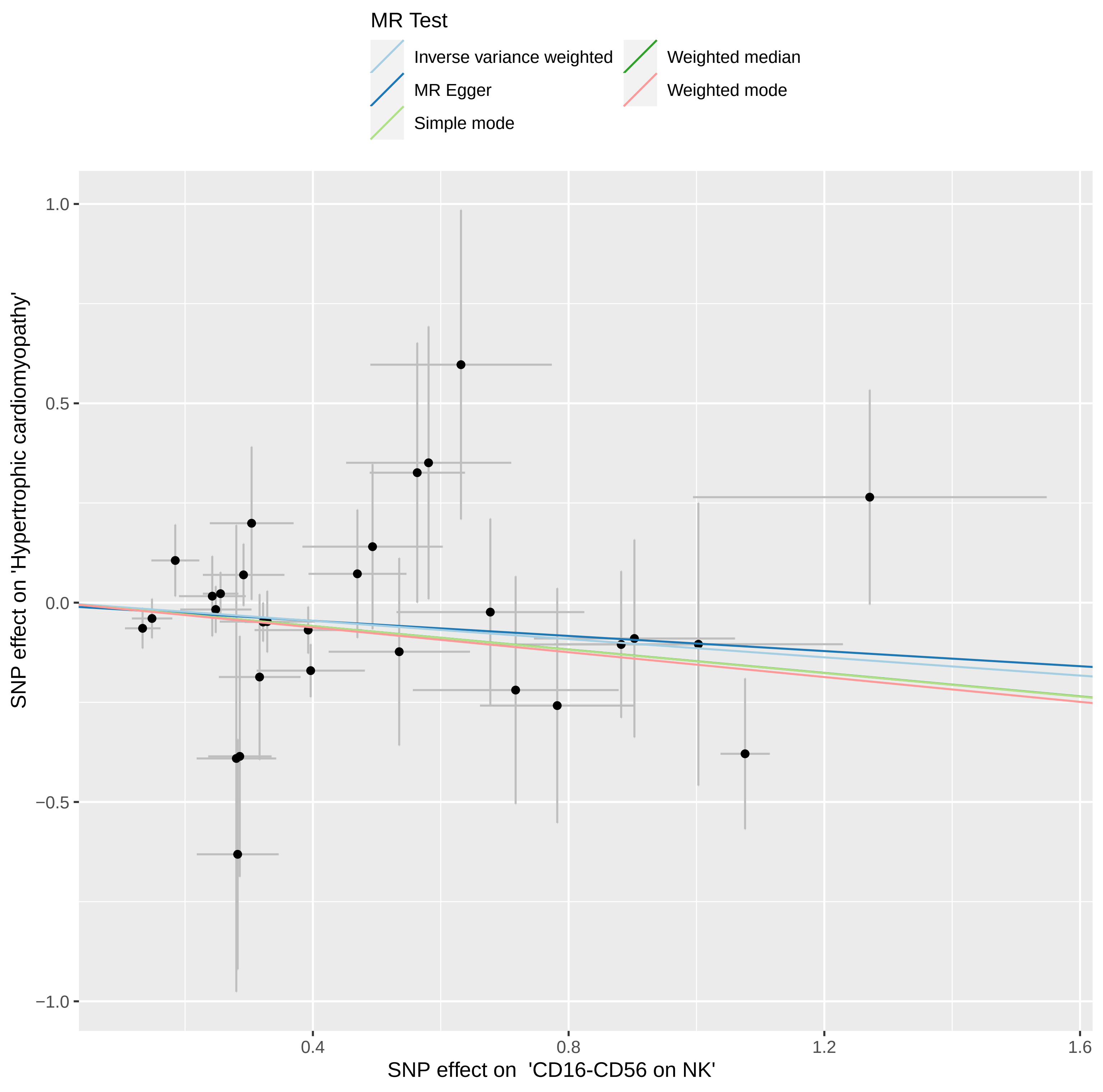

MR Test
Inverse variance weighted
MR Egger
Simple mode
Weighted median
Weighted mode
SNP effect on 'Hypertrophic cardiomyopathy'
1.0
0.5
0.0
−0.5
−1.0
0.4
0.8
1.2
1.6
SNP effect on 'CD16-CD56 on NK'

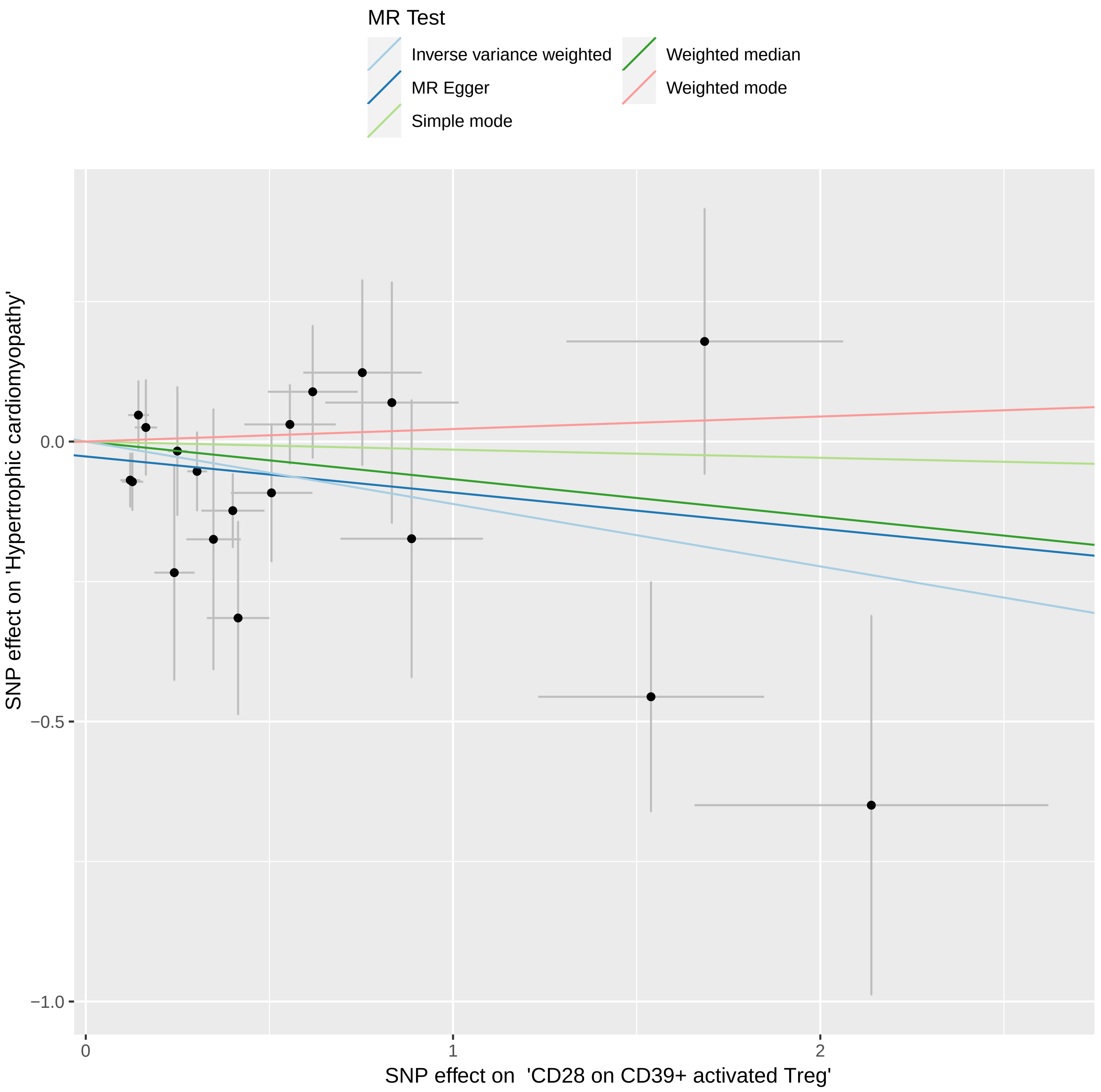
MR Test
Inverse variance weighted
MR Egger
Simple mode
Weighted median
Weighted mode
0.0
−0.5
−1.0
0
1
2
SNP effect on 'Hypertrophic cardiomyopathy'
SNP effect on 'CD28 on CD39+ activated Treg'

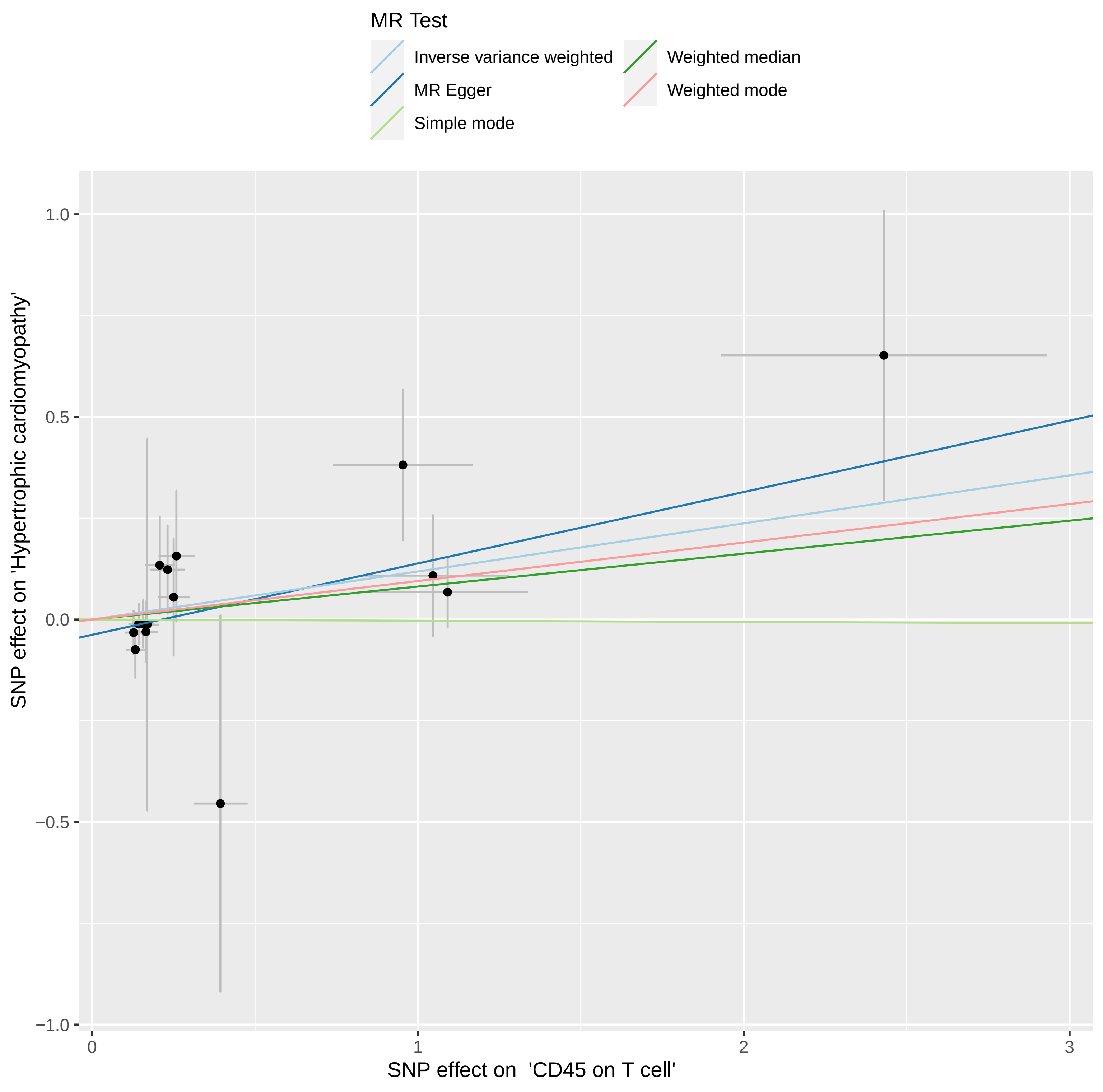
MR Test
Inverse variance weighted
MR Egger
Simple mode
Weighted median
Weighted mode
1.0
0.5
0.0
−0.5
−1.0
0
1
2
3
SNP effect on 'Hypertrophic cardiomyopathy'
SNP effect on 'CD45 on T cell'

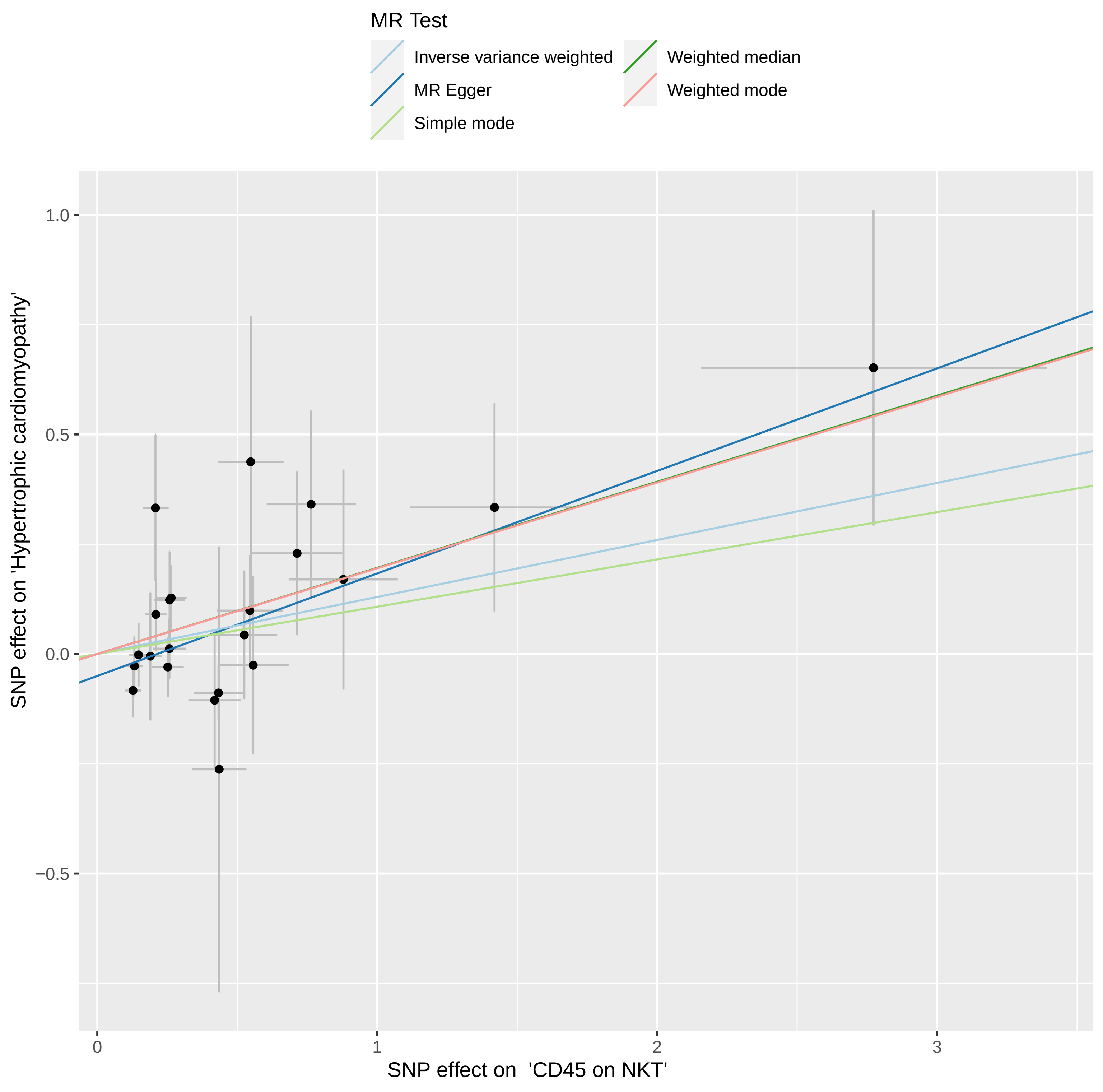

MR Test
Inverse variance weighted
MR Egger
Simple mode
Weighted median
Weighted mode
SNP effect on 'Hypertrophic cardiomyopathy'
1.0
0.5
0.0
−0.5
0
1
2
3
SNP effect on 'CD45 on NKT'

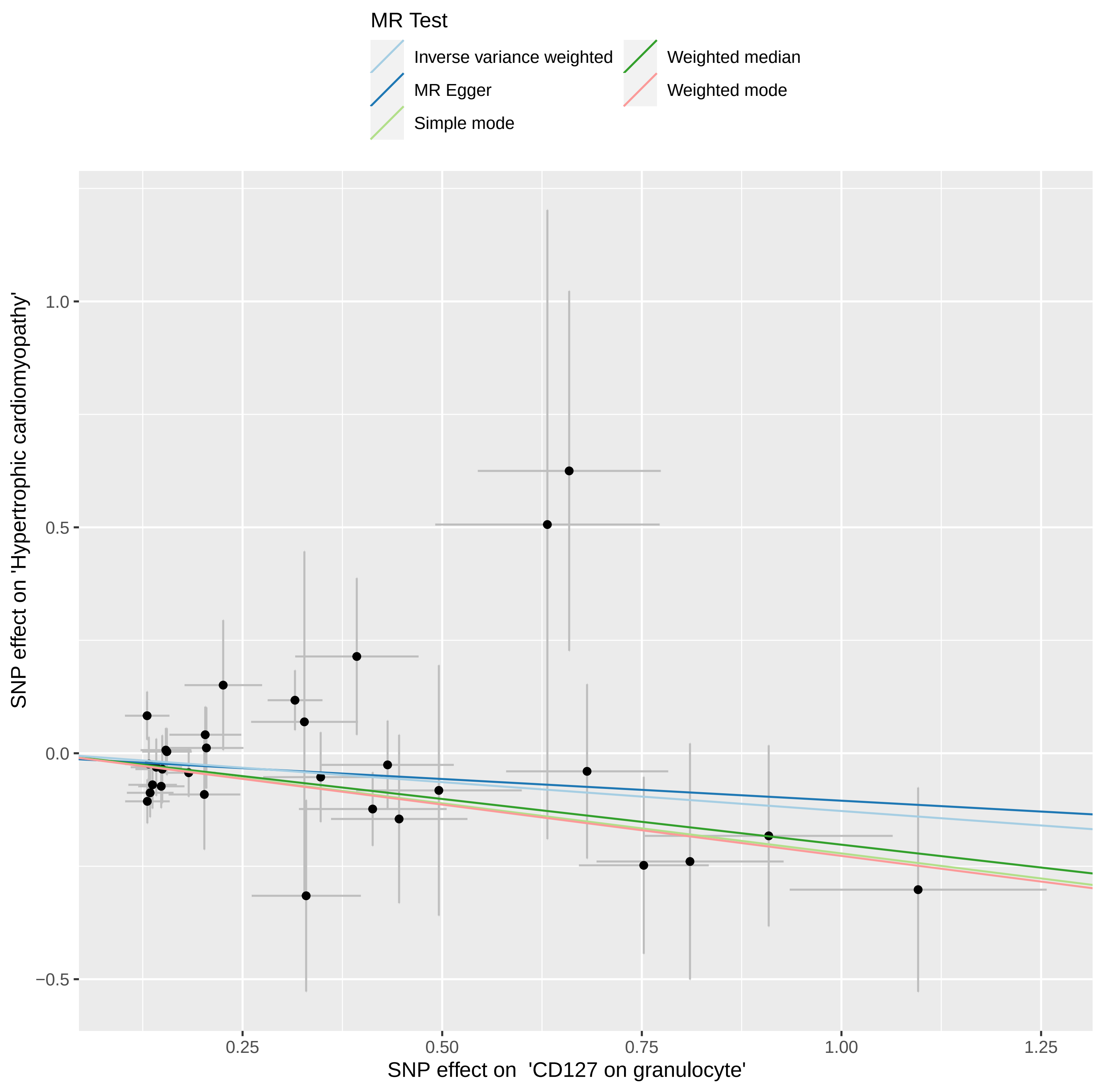

MR Test
Inverse variance weighted
MR Egger
Simple mode
Weighted median
Weighted mode
SNP effect on 'Hypertrophic cardiomyopathy'
1.0
0.5
0.0
−0.5
0.25
0.50
0.75
1.00
1.25
SNP effect on 'CD127 on granulocyte'

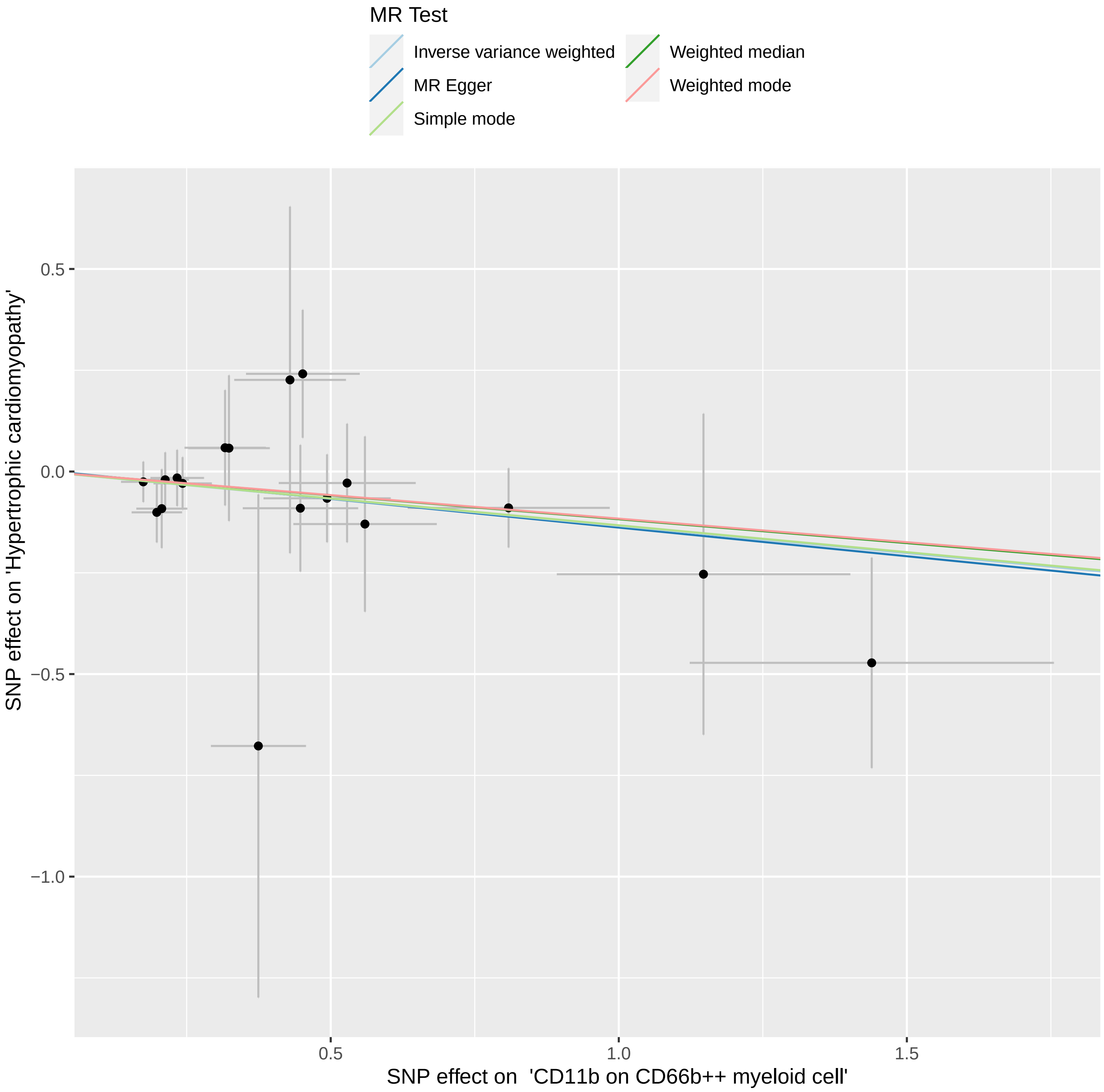
MR Test
Inverse variance weighted
MR Egger
Simple mode
Weighted median
Weighted mode
SNP effect on 'Hypertrophic cardiomyopathy'
0.5
0.0
−0.5
−1.0
0.5
1.0
1.5
SNP effect on 'CD11b on CD66b++ myeloid cell'

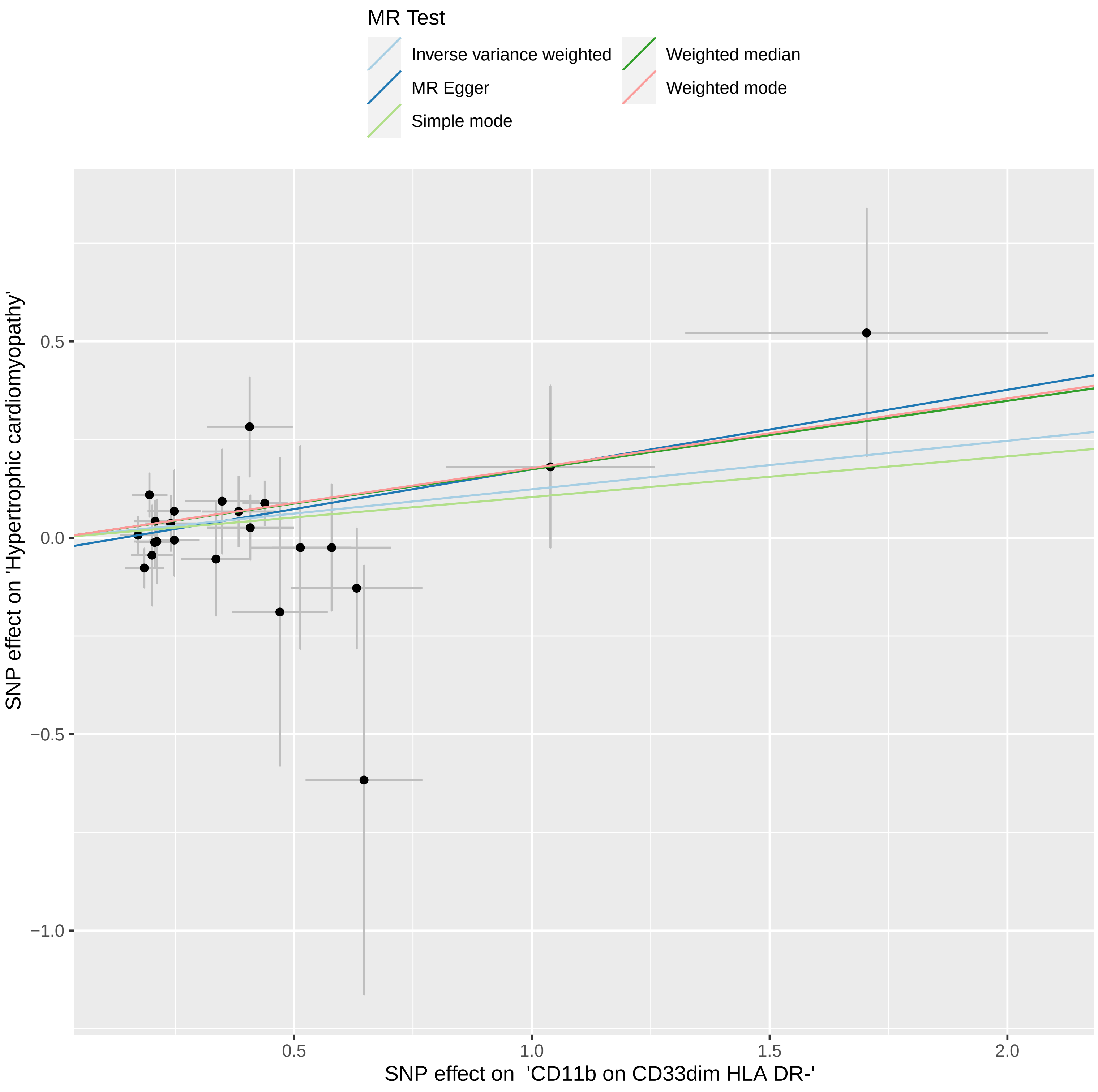

MR Test
Inverse variance weighted
MR Egger
Simple mode
Weighted median
Weighted mode
SNP effect on 'Hypertrophic cardiomyopathy'
0.5
0.0
−0.5
−1.0
0.5
1.0
1.5
2.0
SNP effect on 'CD11b on CD33dim HLA DR-'

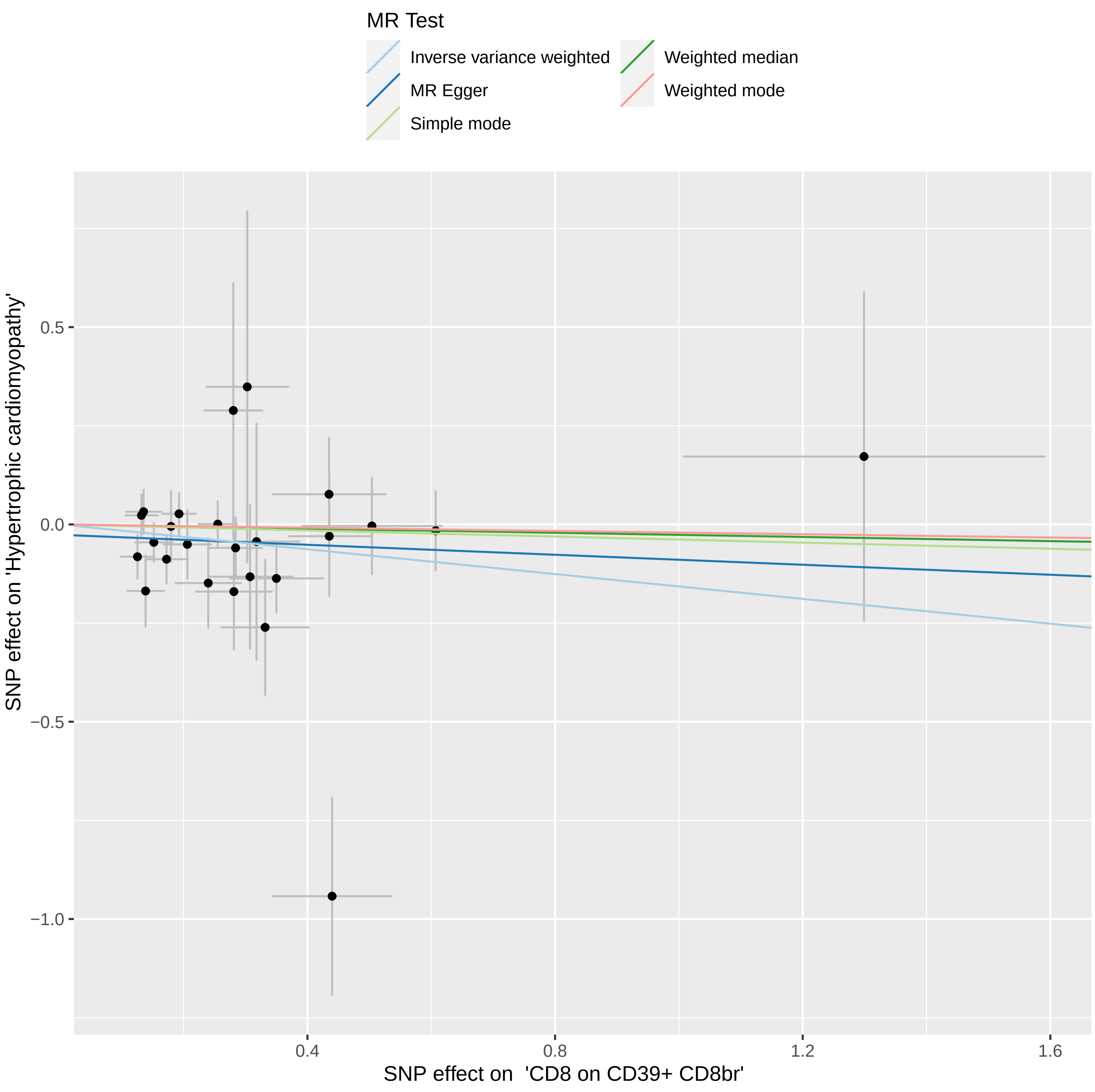
MR Test
Inverse variance weighted
MR Egger
Simple mode
Weighted median
Weighted mode
SNP effect on 'Hypertrophic cardiomyopathy'
0.5
0.0
−0.5
−1.0
0.4
0.8
1.2
1.6
SNP effect on 'CD8 on CD39+ CD8br'

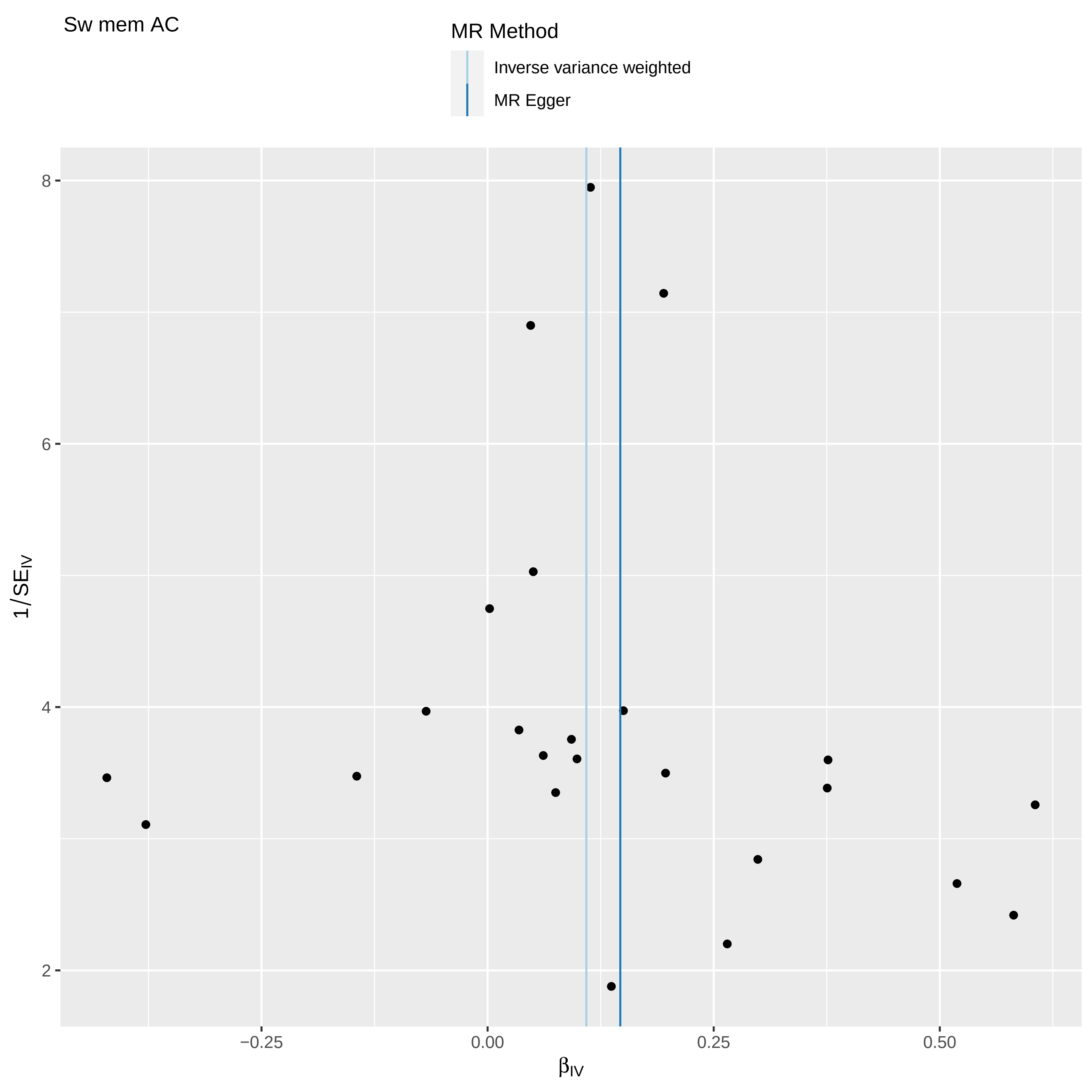
Sw mem AC
MR Method
Inverse variance weighted
MR Egger
1/SE_IV
8
6
4
2
−0.25
0.00
0.25
0.50
β_IV

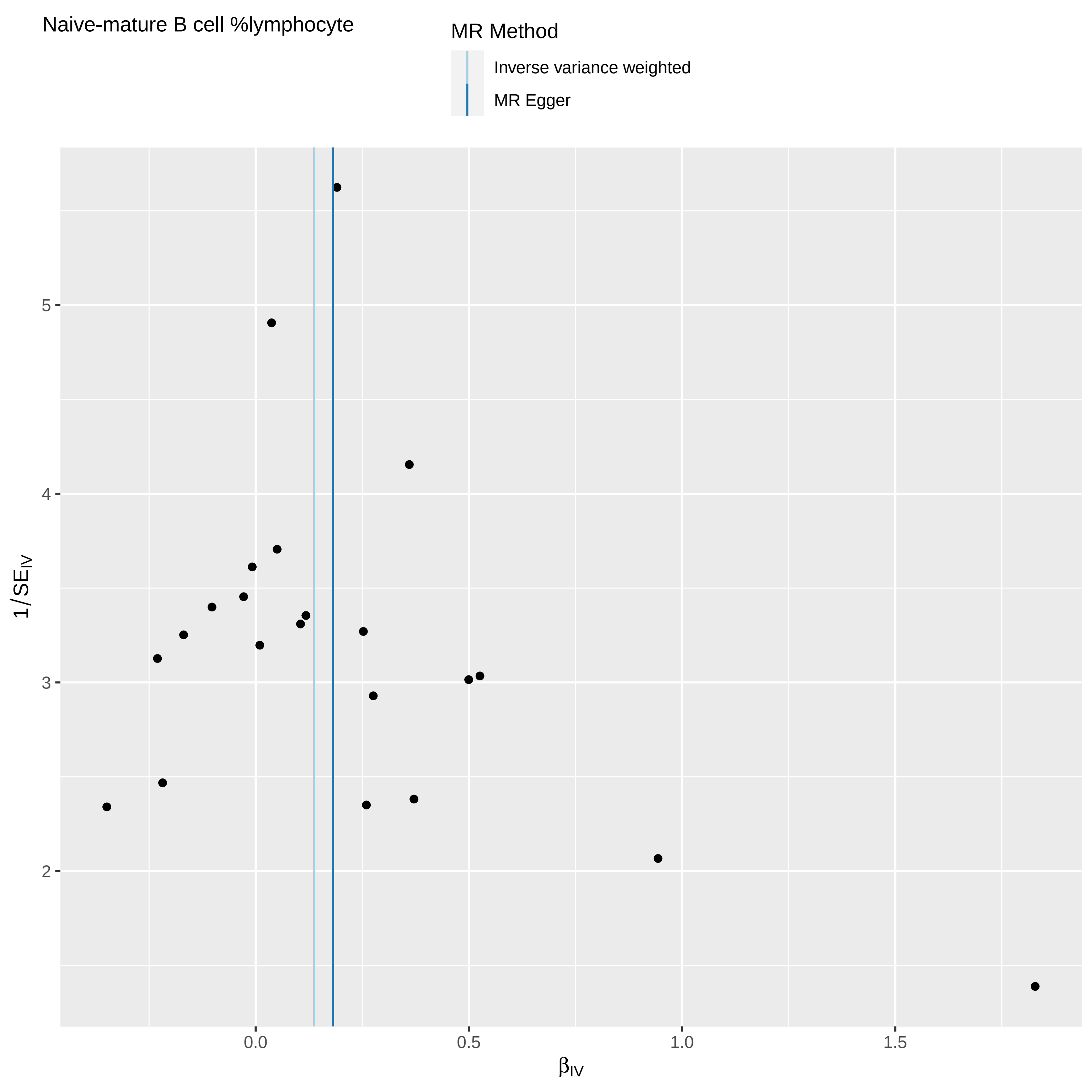
Naive-mature B cell %lymphocyte
MR Method
Inverse variance weighted
MR Egger
1/SE$_{IV}$
5
4
3
2
0.0
0.5
1.0
1.5
β$_{IV}$

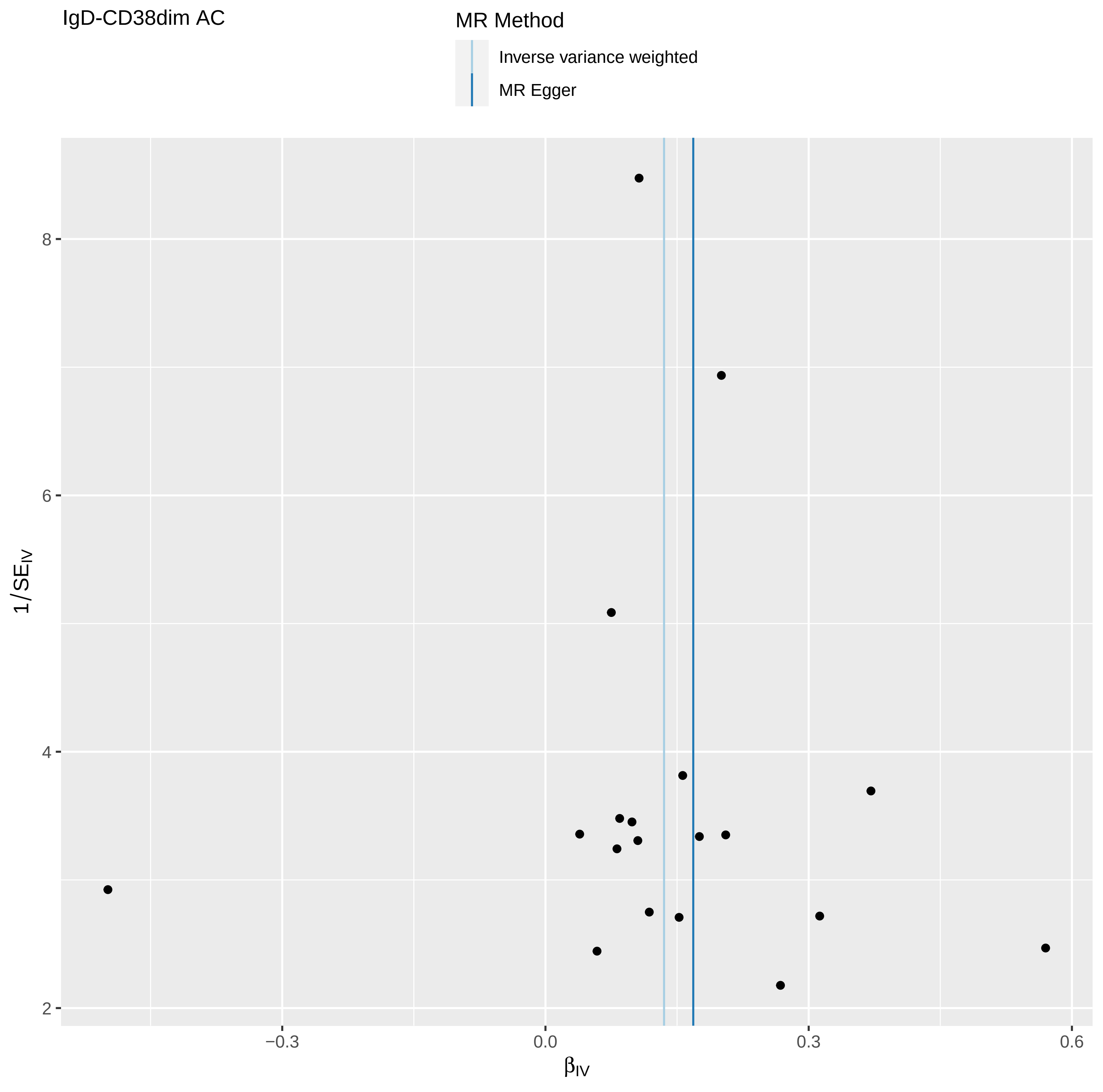
IgD-CD38dim AC
MR Method
Inverse variance weighted
MR Egger
1/SE_IV
β_IV
8
6
4
2
−0.3
0.0
0.3
0.6

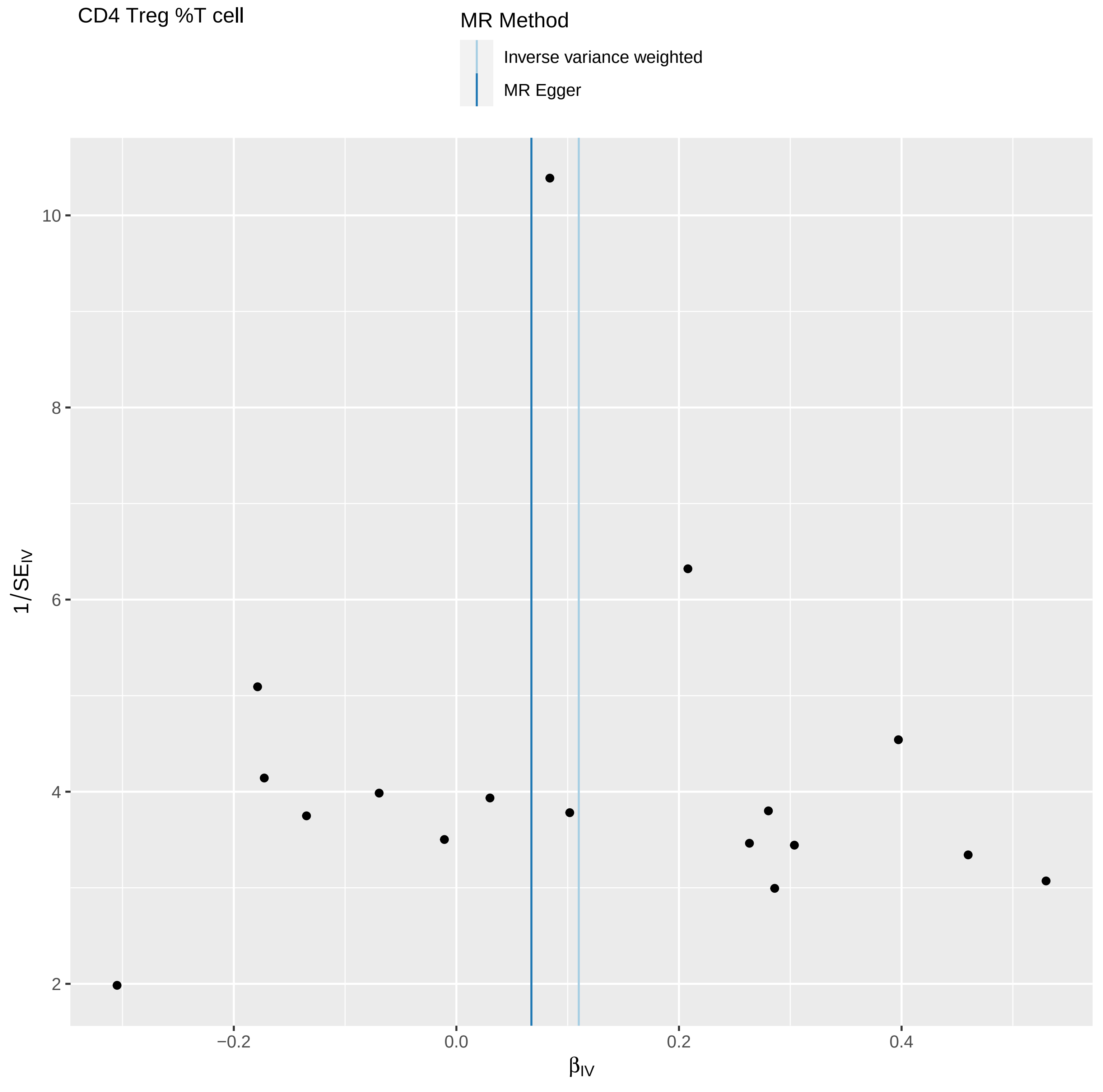

CD4 Treg %T cell
MR Method
Inverse variance weighted
MR Egger
1/SE$_{IV}$
10
8
6
4
2
−0.2
0.0
0.2
0.4
β$_{IV}$

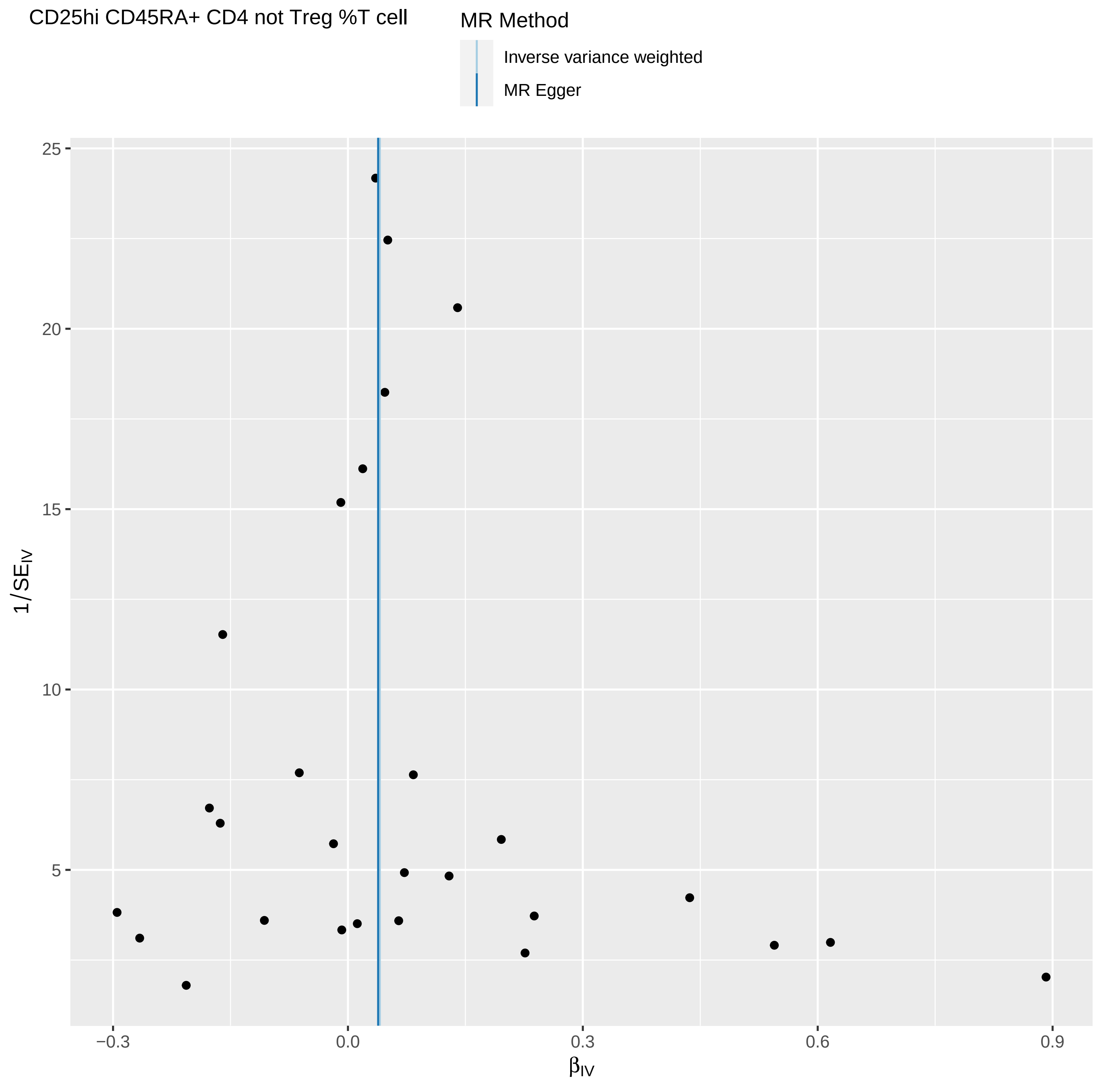

CD25hi CD45RA+ CD4 not Treg %T cell
MR Method
Inverse variance weighted
MR Egger
25
20
15
10
5
1/SE_IV
−0.3
0.0
0.3
0.6
0.9
β_IV

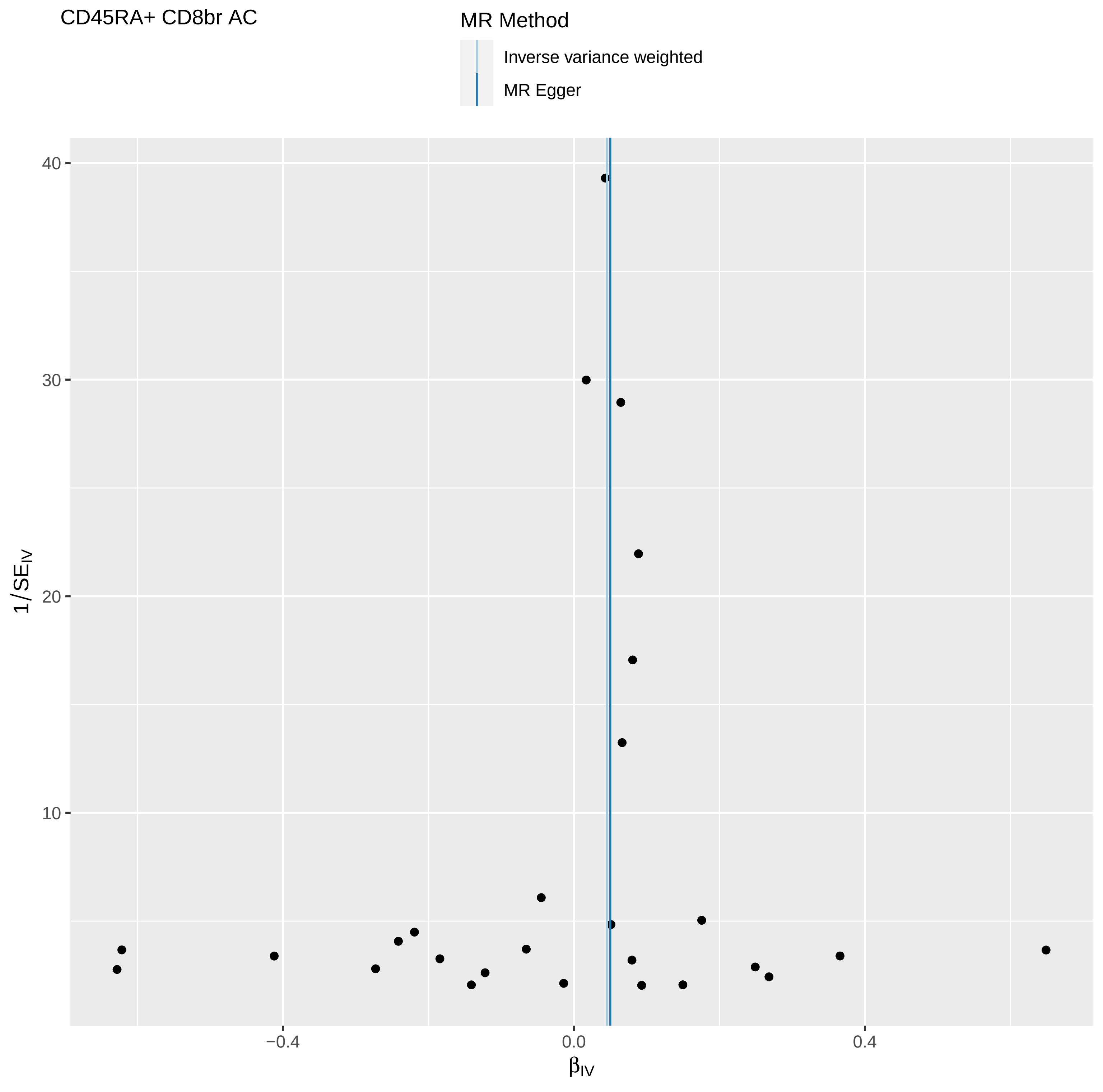
CD45RA+ CD8br AC
MR Method
Inverse variance weighted
MR Egger
40
30
20
10
1/SE_IV
−0.4
0.0
0.4
β_IV

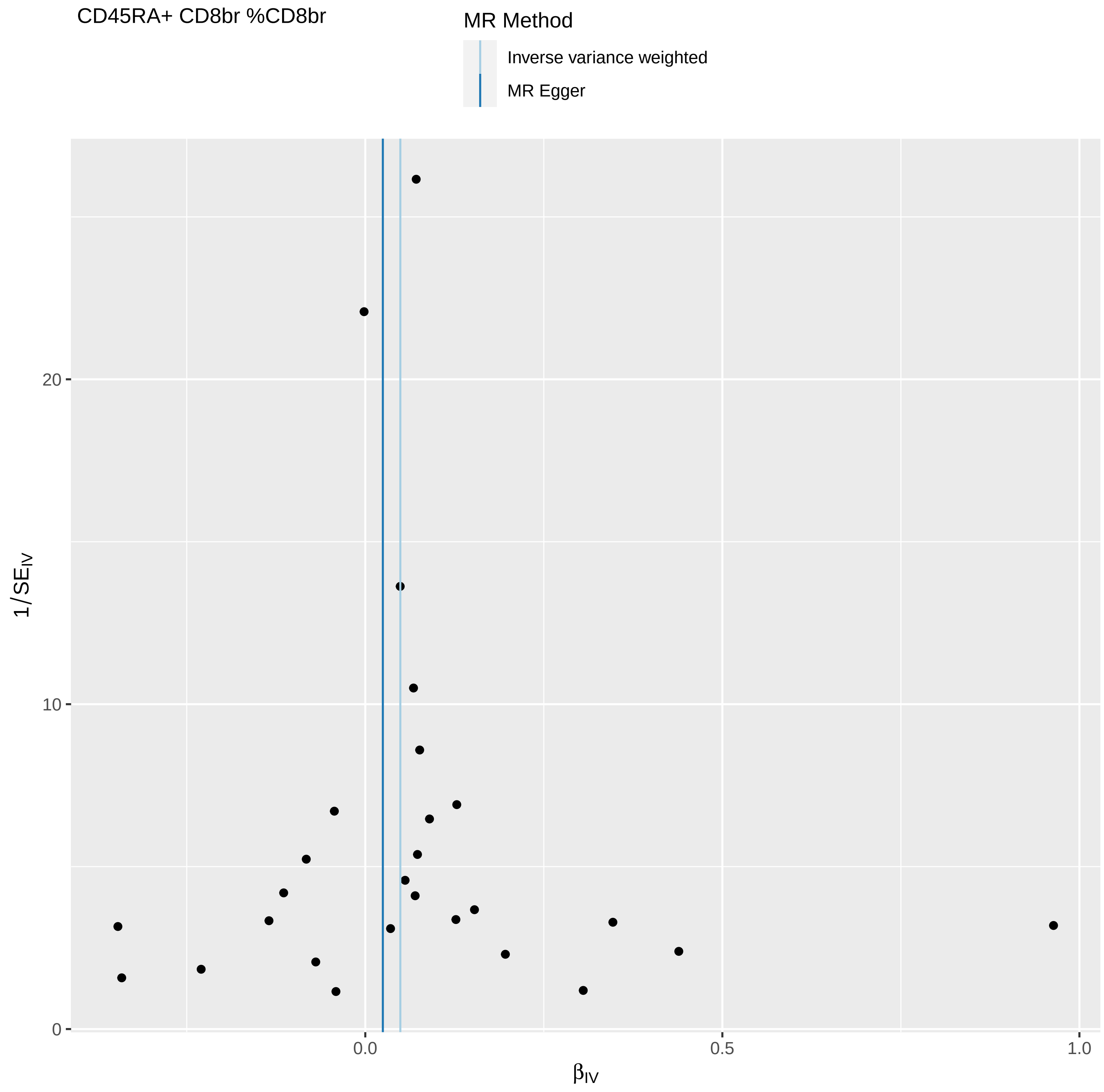
CD45RA+ CD8br %CD8br
MR Method
Inverse variance weighted
MR Egger
20
10
0
1/SE_IV
0.0
0.5
1.0
β_IV

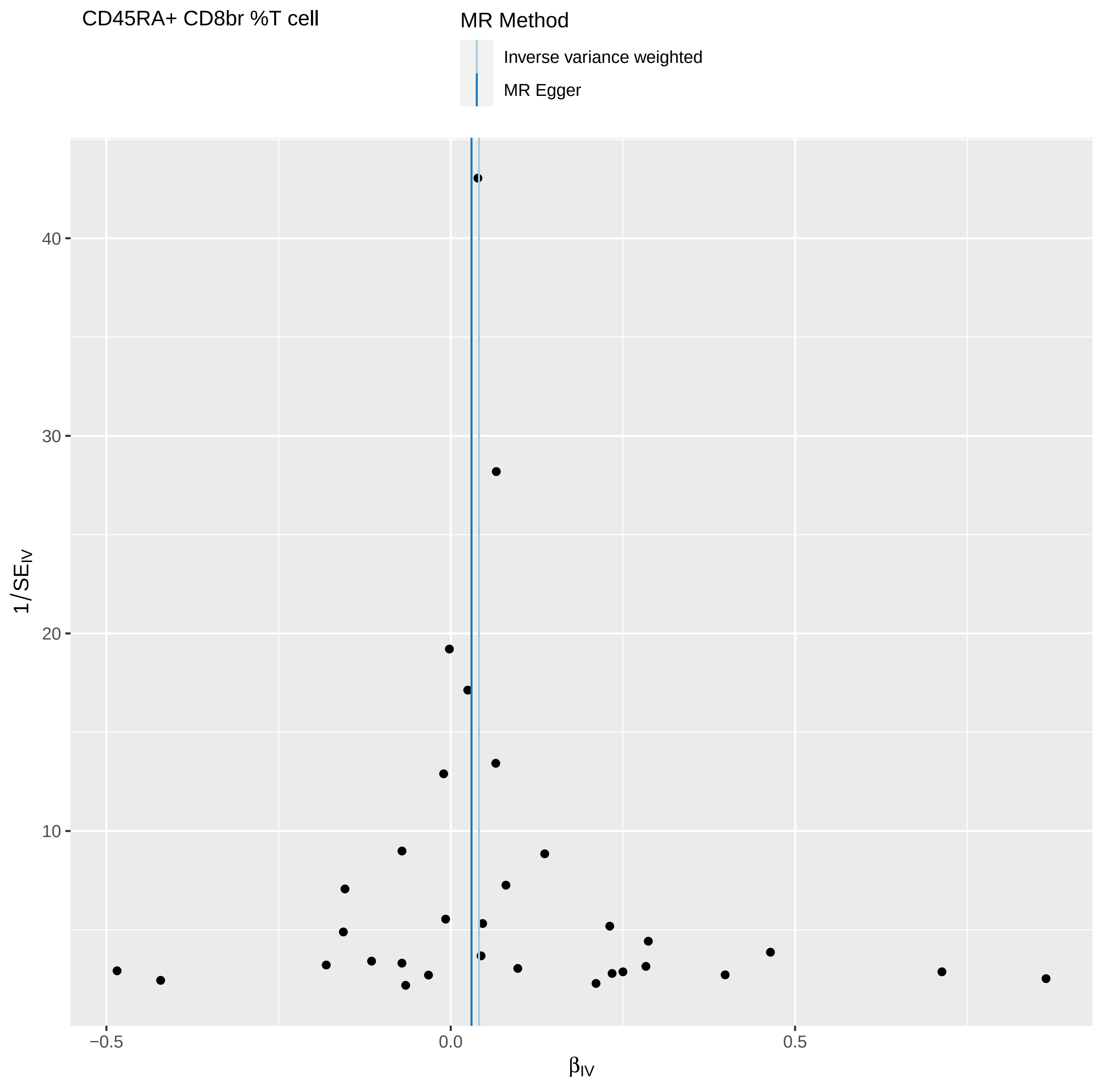
CD45RA+ CD8br %T cell
MR Method
Inverse variance weighted
MR Egger
1/SE_IV
40
30
20
10
−0.5
0.0
0.5
β_IV

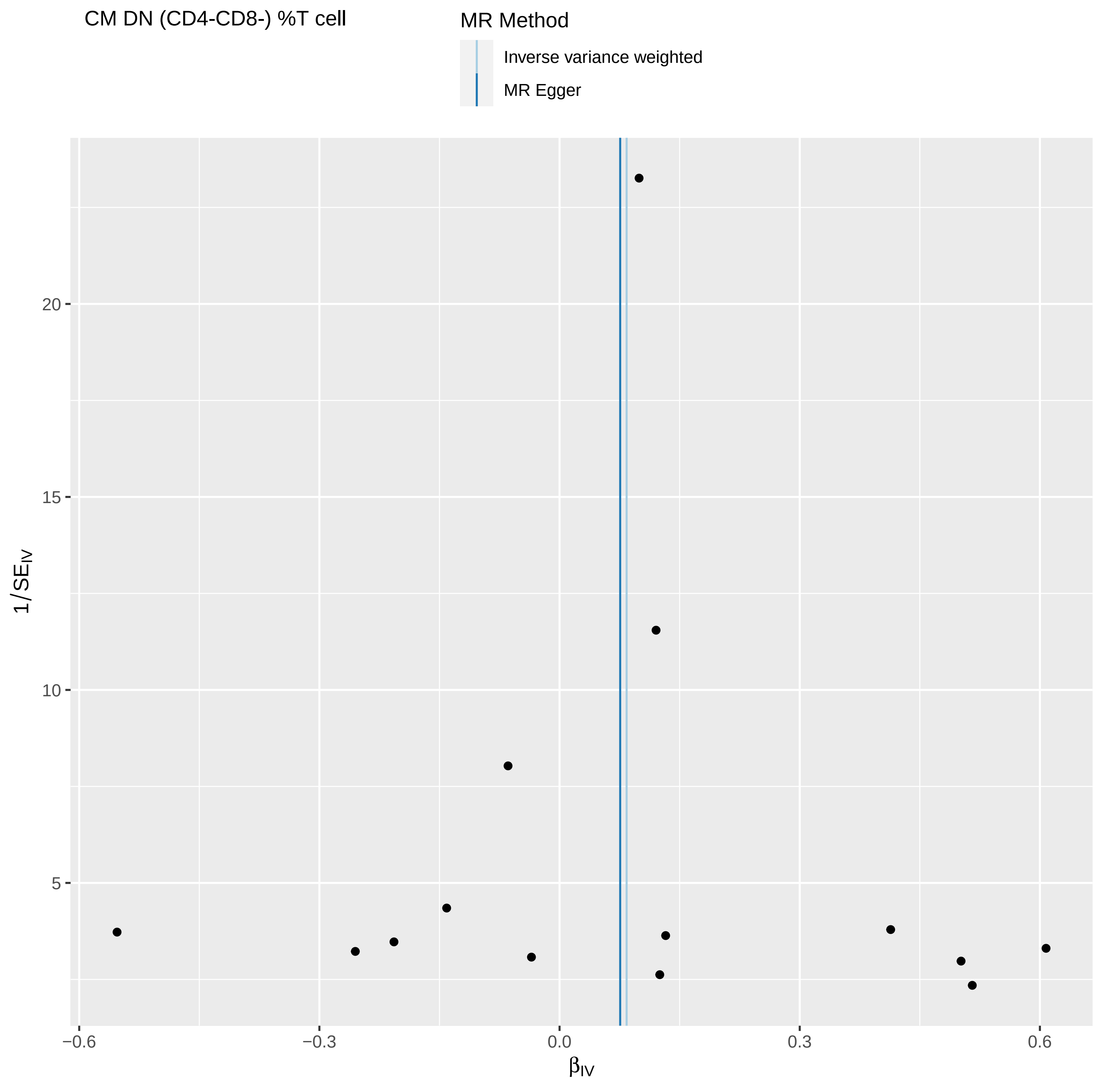
CM DN (CD4-CD8-) %T cell
MR Method
Inverse variance weighted
MR Egger
20
15
10
5
1/SE_IV
−0.6
−0.3
0.0
0.3
0.6
β_IV

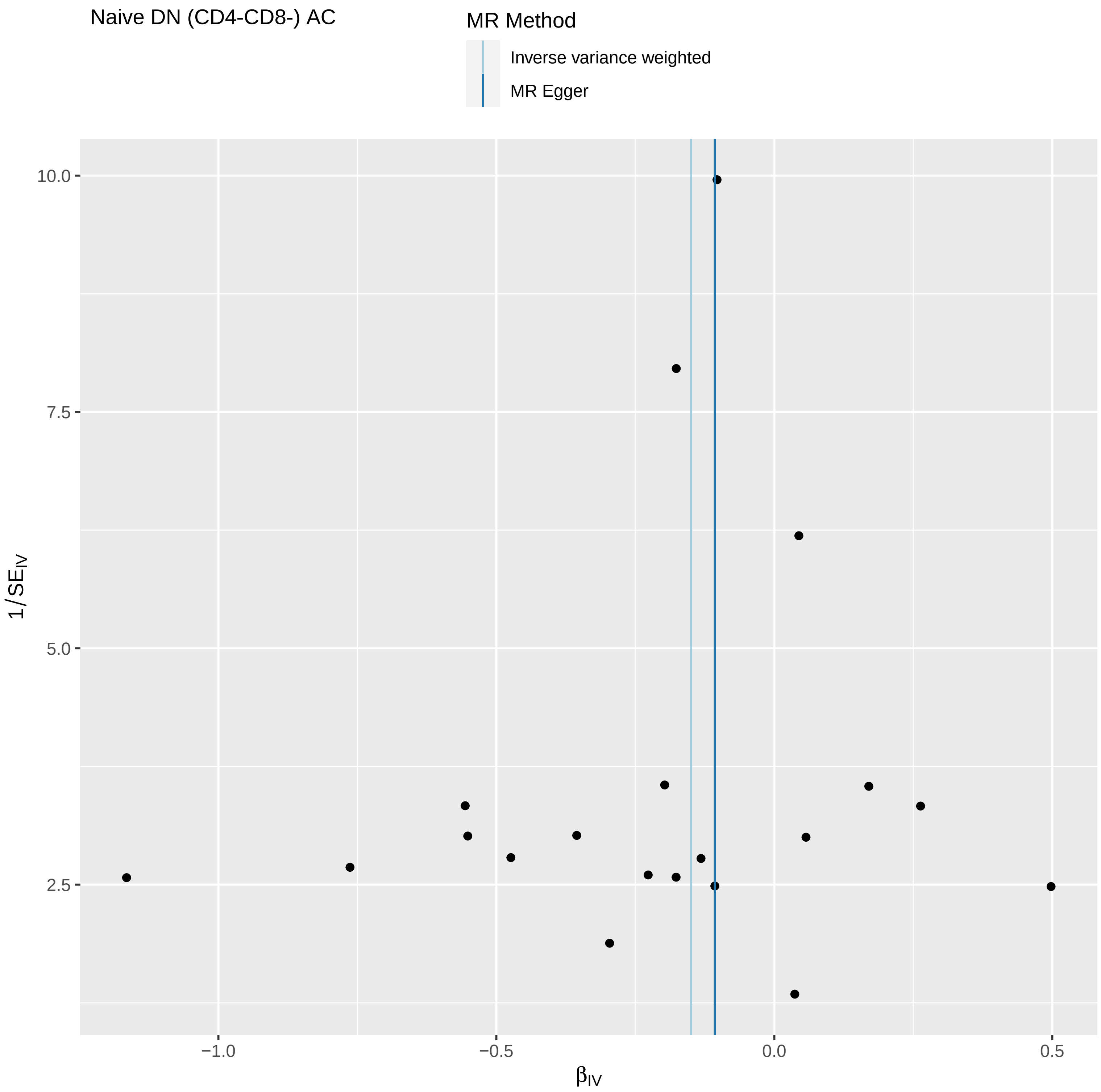
Naive DN (CD4-CD8-) AC
MR Method
Inverse variance weighted
MR Egger
1/SE_IV
β_IV
10.0
7.5
5.0
2.5
−1.0
−0.5
0.0
0.5

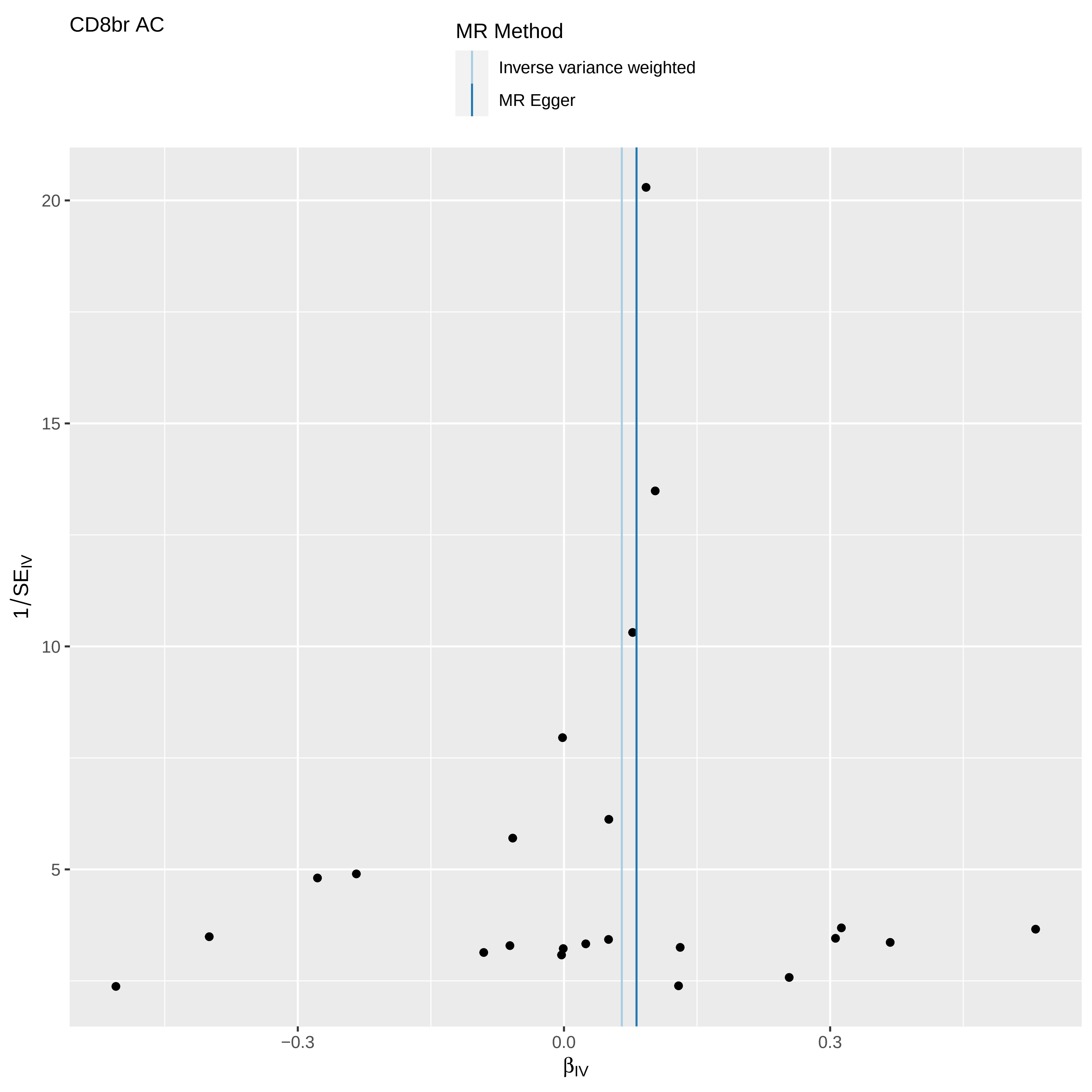
CD8br AC
MR Method
Inverse variance weighted
MR Egger
20
15
10
5
1/SE$_{IV}$
−0.3
0.0
0.3
β$_{IV}$

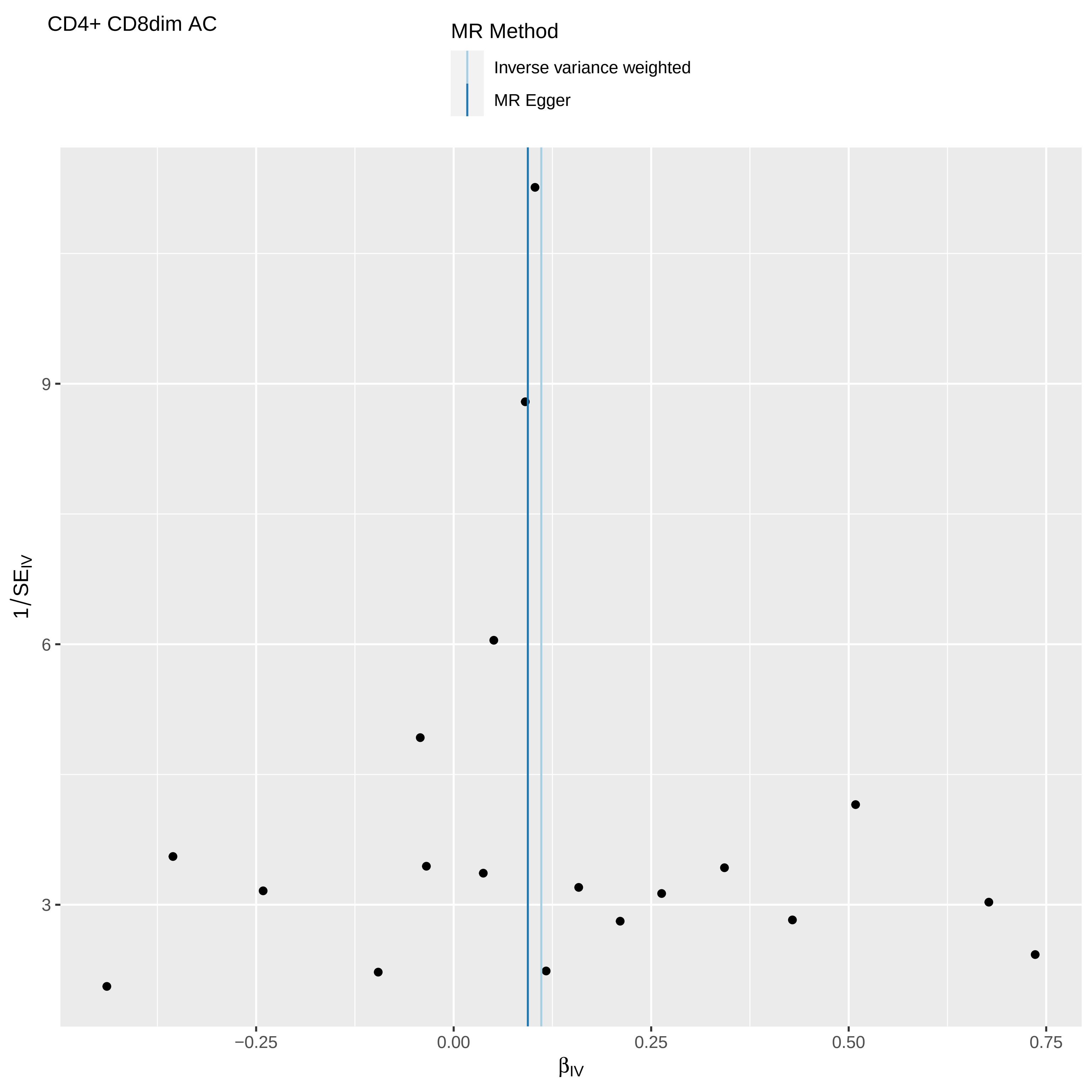
CD4+ CD8dim AC
MR Method
Inverse variance weighted
MR Egger
9
6
3
1/SE_IV
−0.25
0.00
0.25
0.50
0.75
β_IV

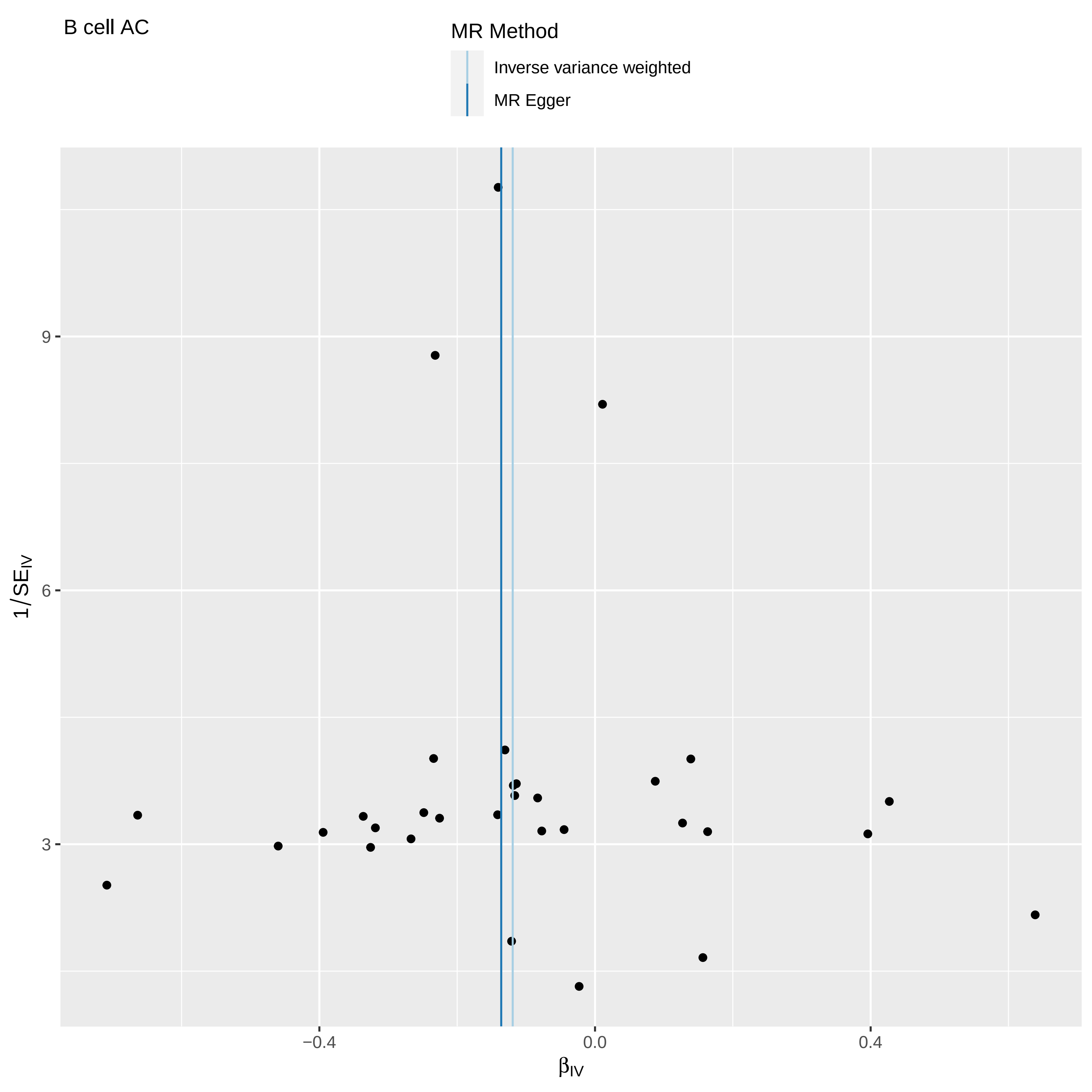

B cell AC
MR Method
Inverse variance weighted
MR Egger
1/SE$_{IV}$
9
6
3
−0.4
0.0
0.4
β$_{IV}$

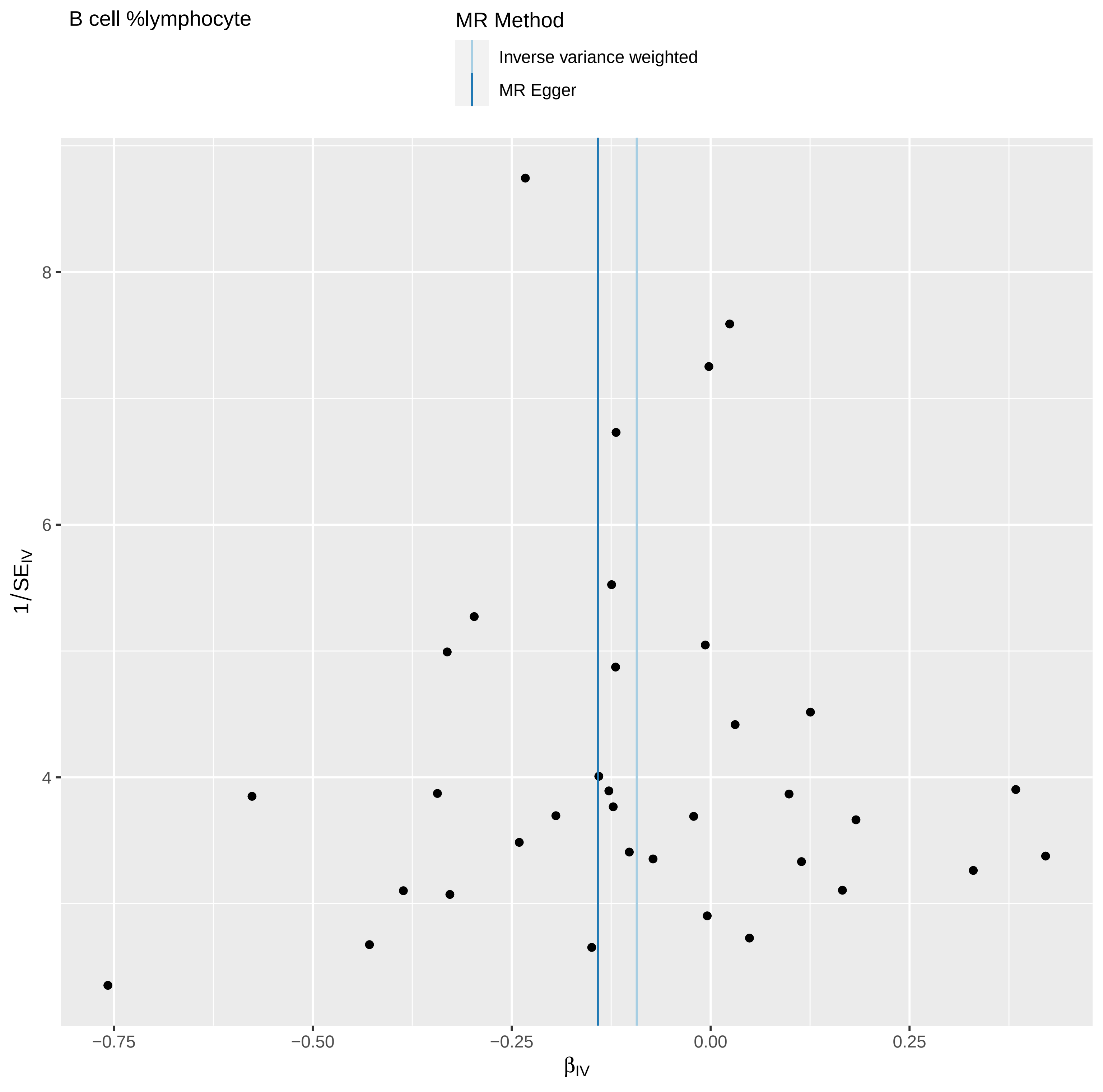
B cell %lymphocyte
MR Method
Inverse variance weighted
MR Egger
8
6
4
1/SE_IV
−0.75
−0.50
−0.25
0.00
0.25
β_IV

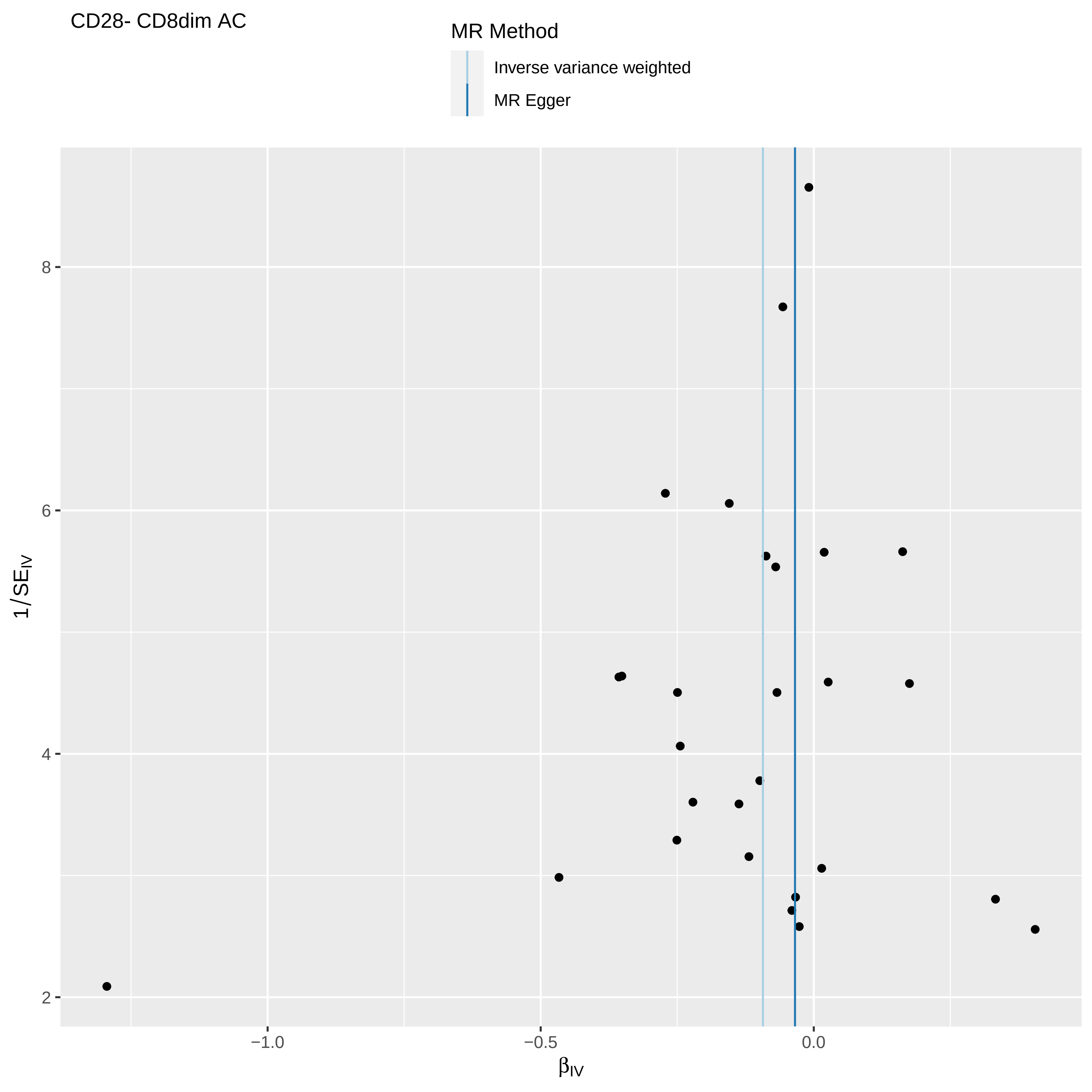
CD28- CD8dim AC
MR Method
Inverse variance weighted
MR Egger
1/SE_IV
β_IV
8
6
4
2
−1.0
−0.5
0.0

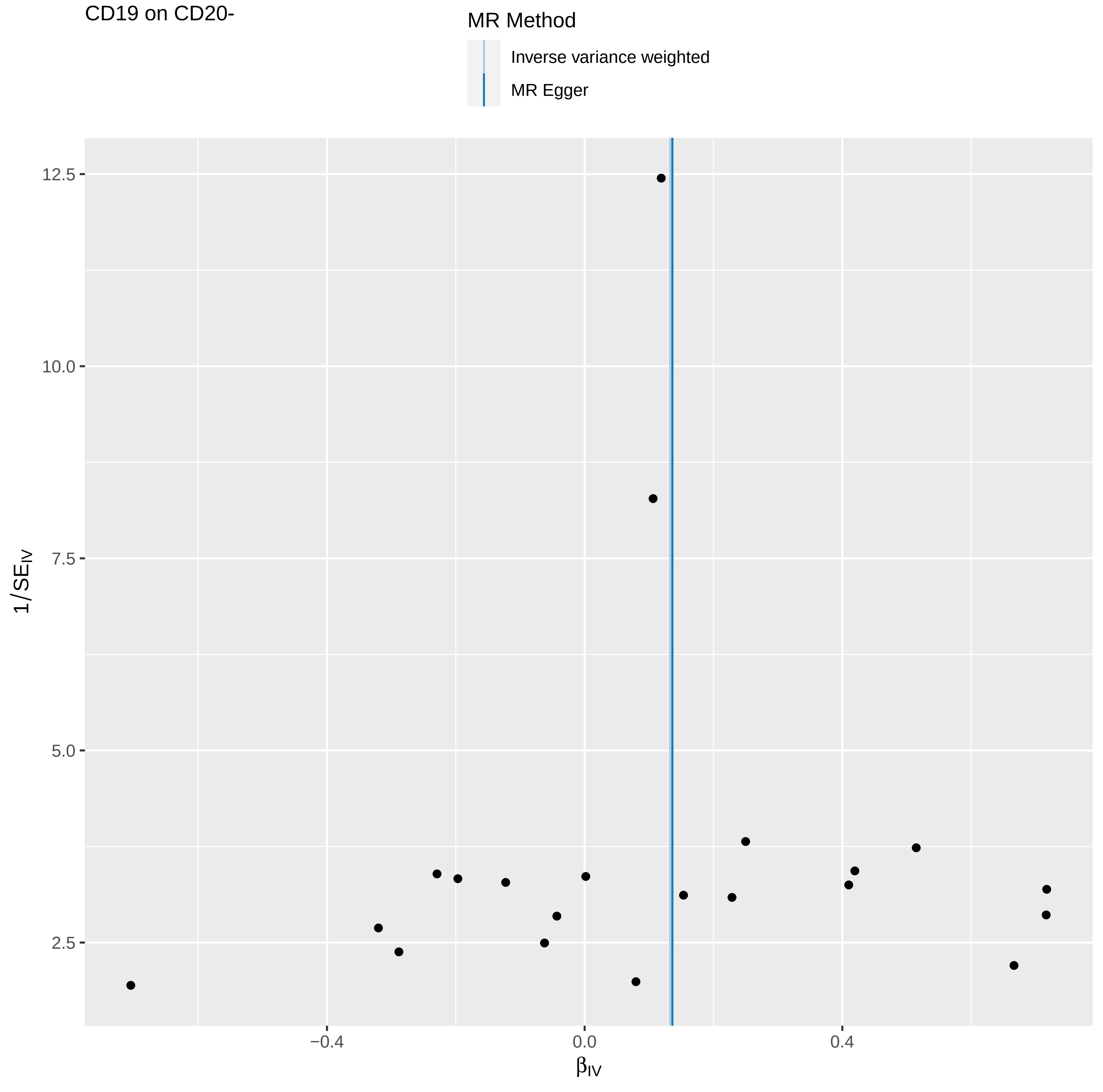

CD19 on CD20-
MR Method
Inverse variance weighted
MR Egger
12.5
10.0
7.5
5.0
2.5
1/SE_IV
−0.4
0.0
0.4
β_IV

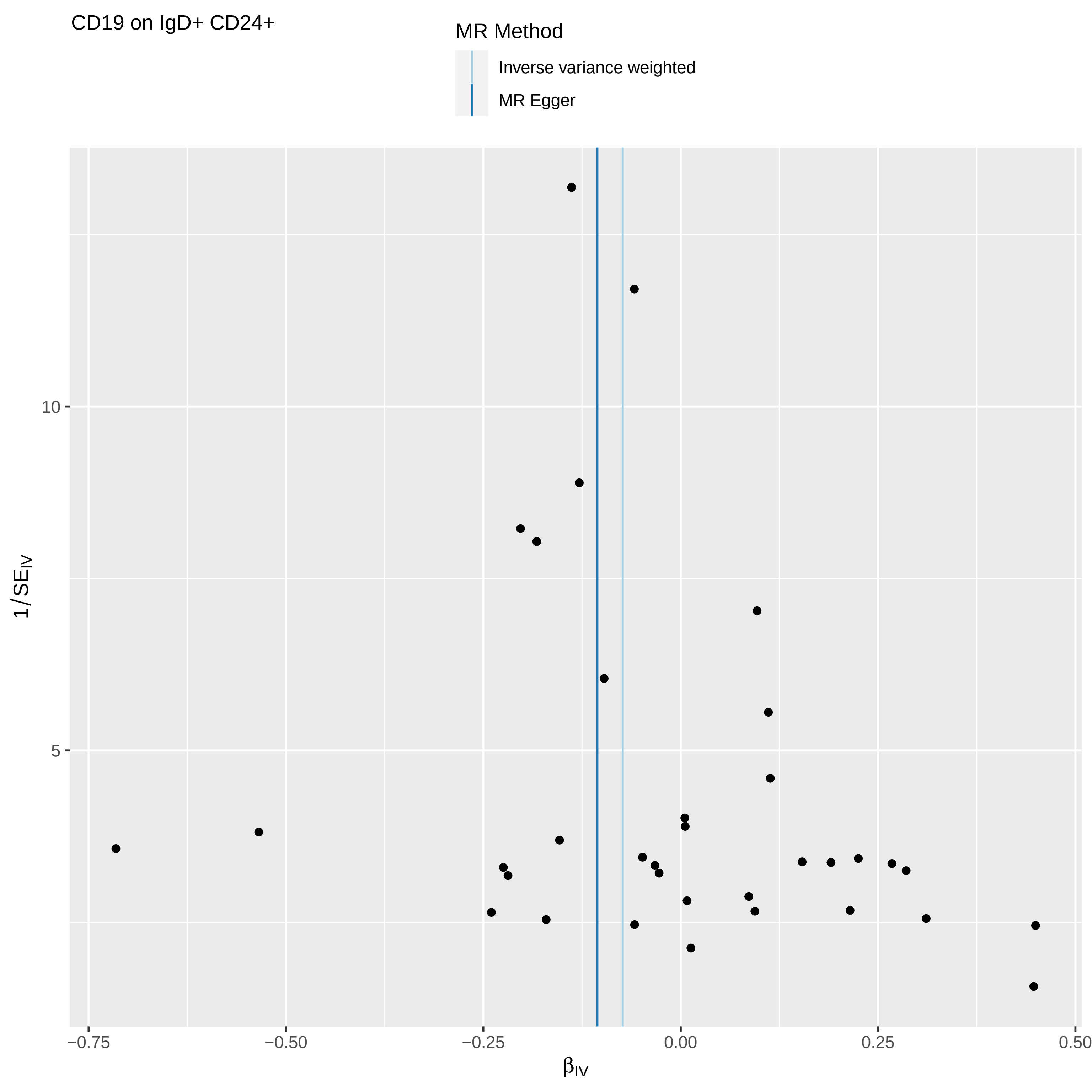
CD19 on IgD+ CD24+
MR Method
Inverse variance weighted
MR Egger
10
5
1/SE_IV
−0.75
−0.50
−0.25
0.00
0.25
0.50
β_IV

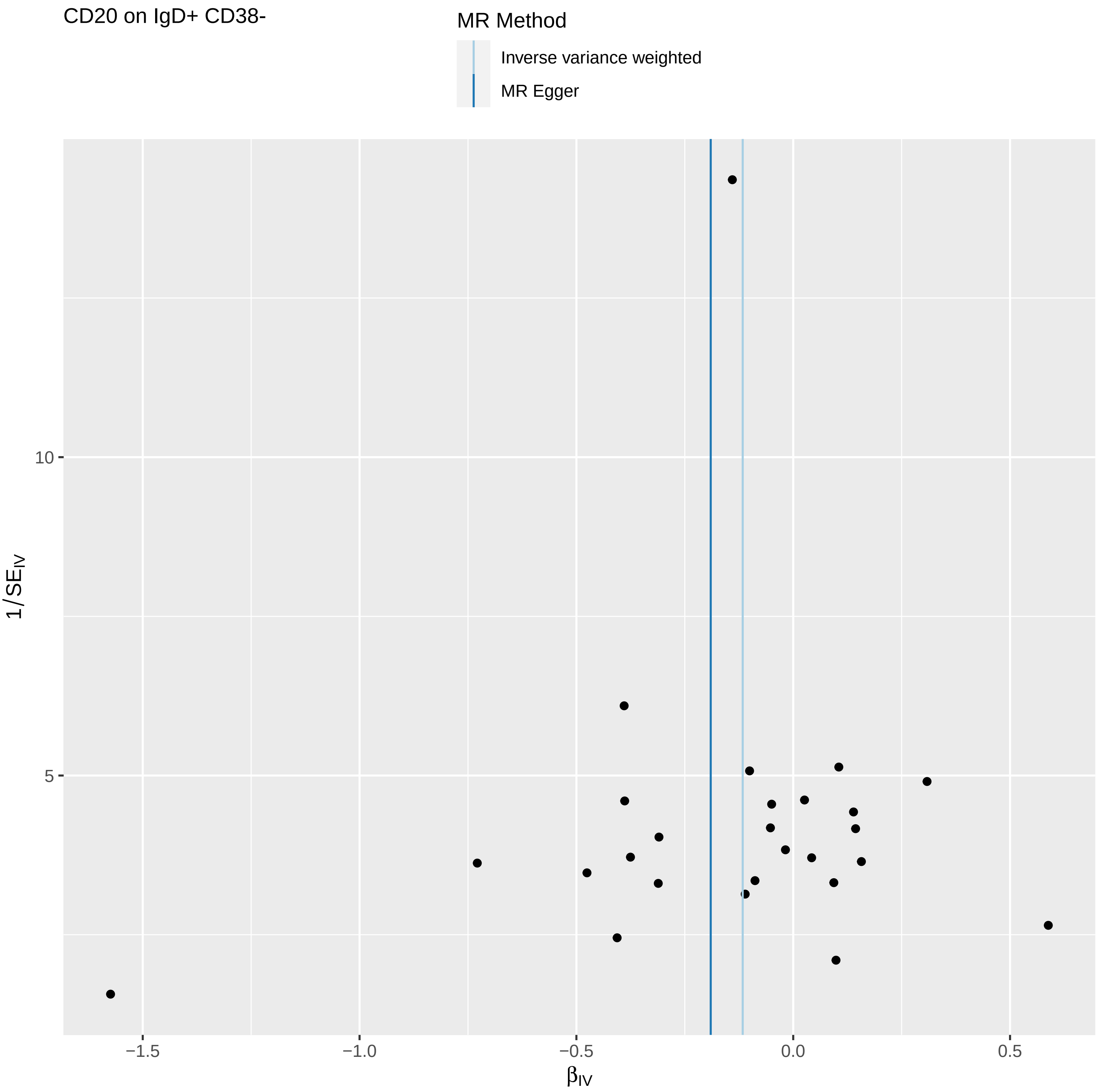
CD20 on IgD+ CD38-
MR Method
Inverse variance weighted
MR Egger
1/SE_IV
10
5
−1.5
−1.0
−0.5
0.0
0.5
β_IV

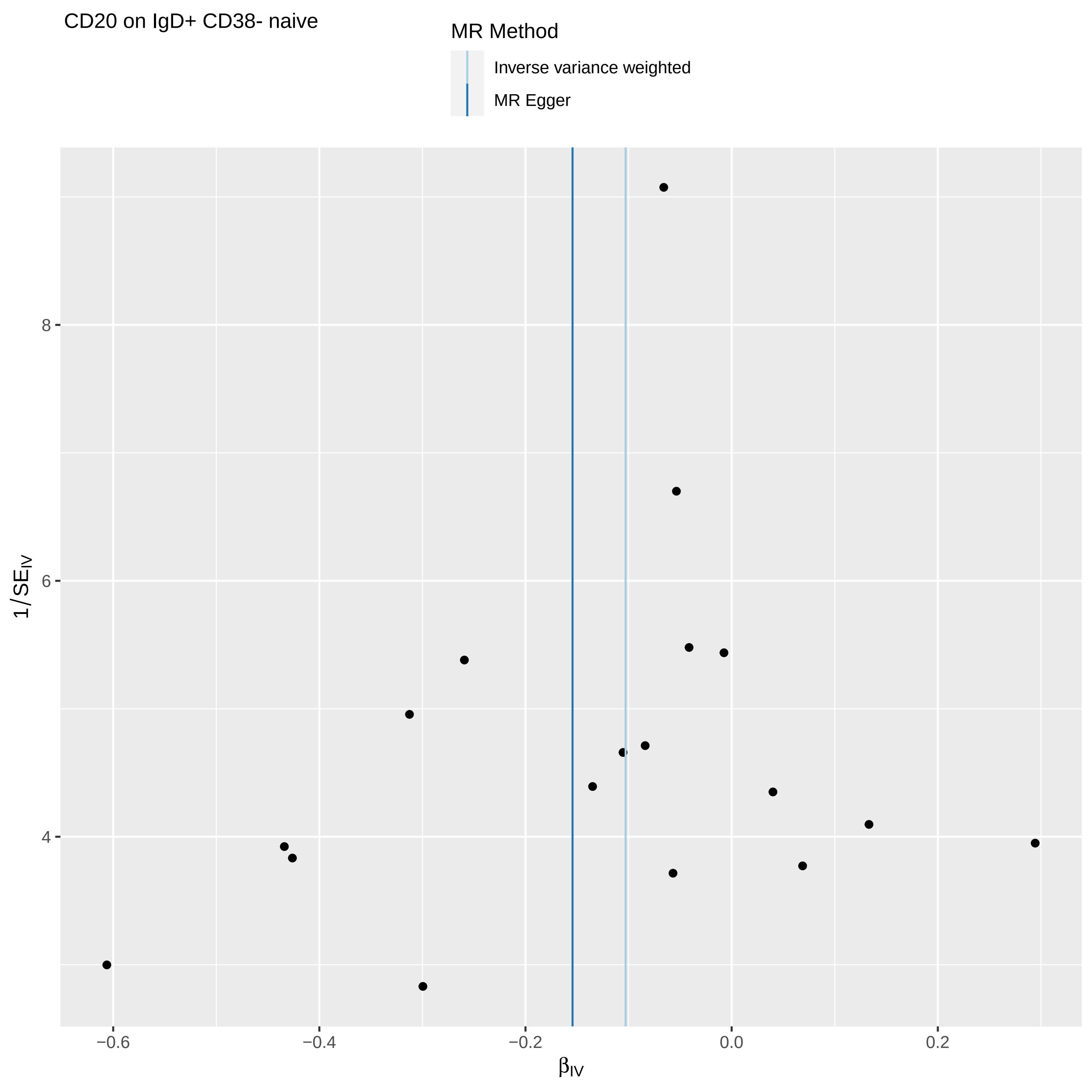

CD20 on IgD+ CD38- naive
MR Method
Inverse variance weighted
MR Egger
8
6
4
1/SE_IV
−0.6
−0.4
−0.2
0.0
0.2
β_IV

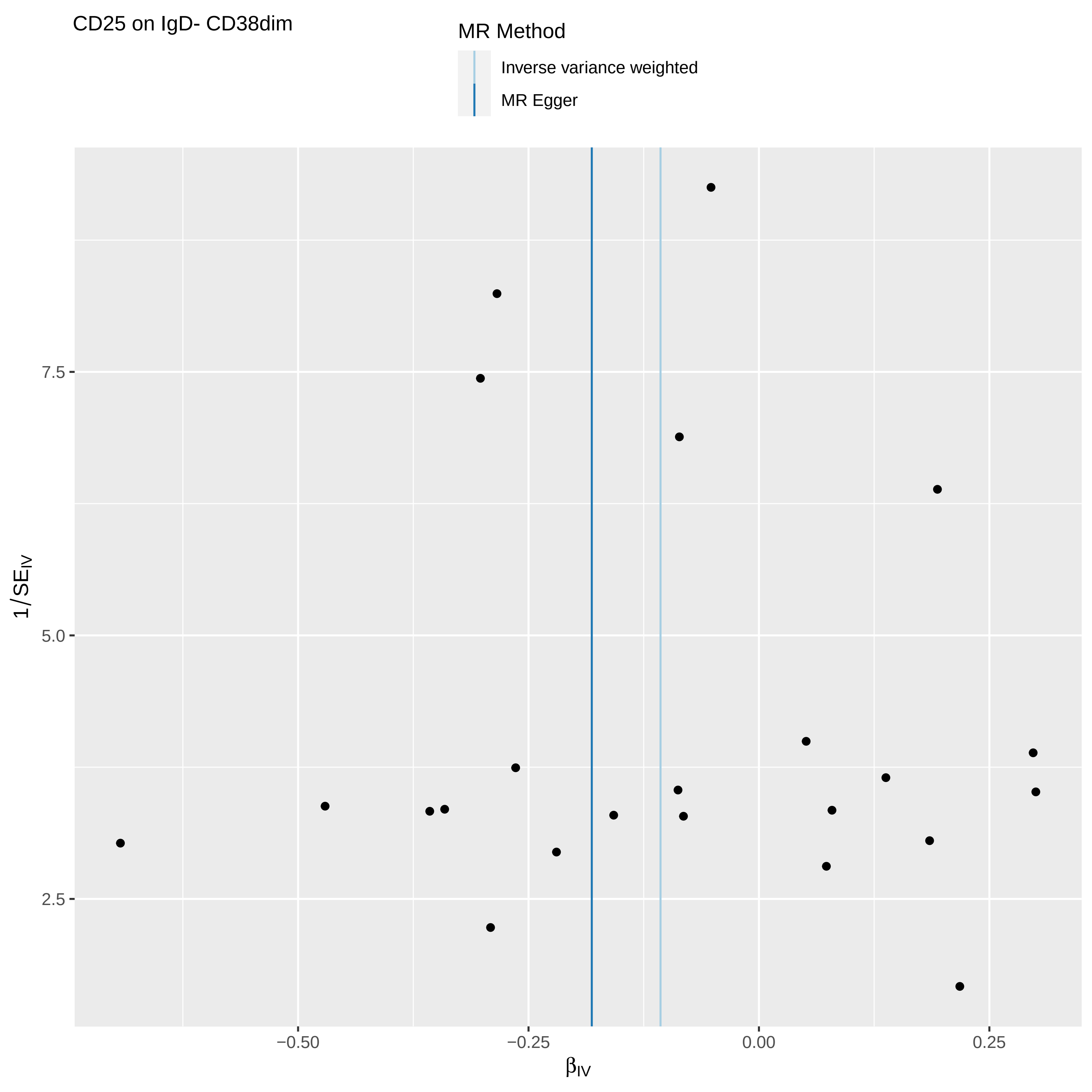
CD25 on IgD- CD38dim
MR Method
Inverse variance weighted
MR Egger
7.5
5.0
2.5
$1/SE_{IV}$
−0.50
−0.25
0.00
0.25
β_{IV}

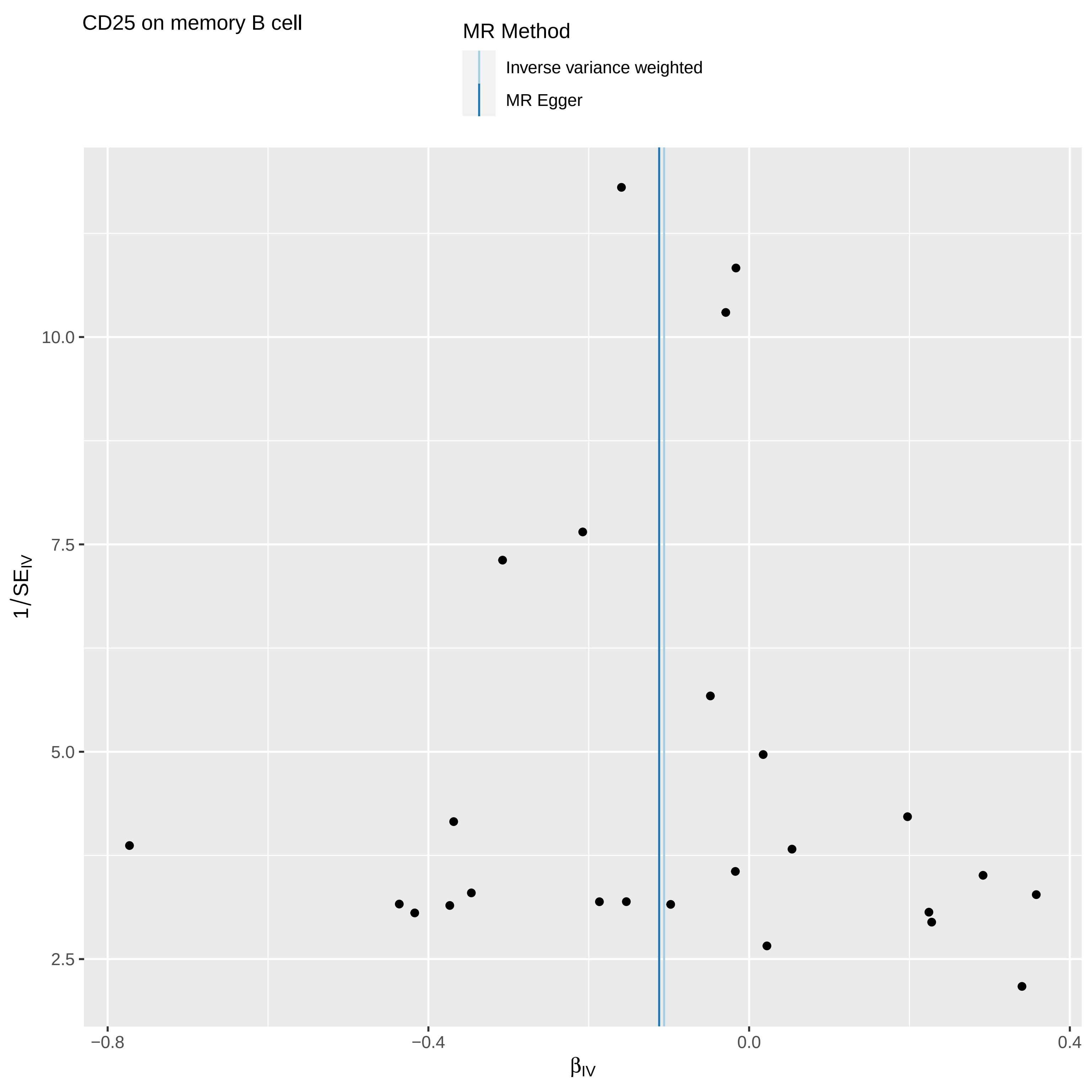
CD25 on memory B cell
MR Method
Inverse variance weighted
MR Egger
10.0
7.5
5.0
2.5
$1/SE_{IV}$
−0.8
−0.4
0.0
0.4
β_{IV}

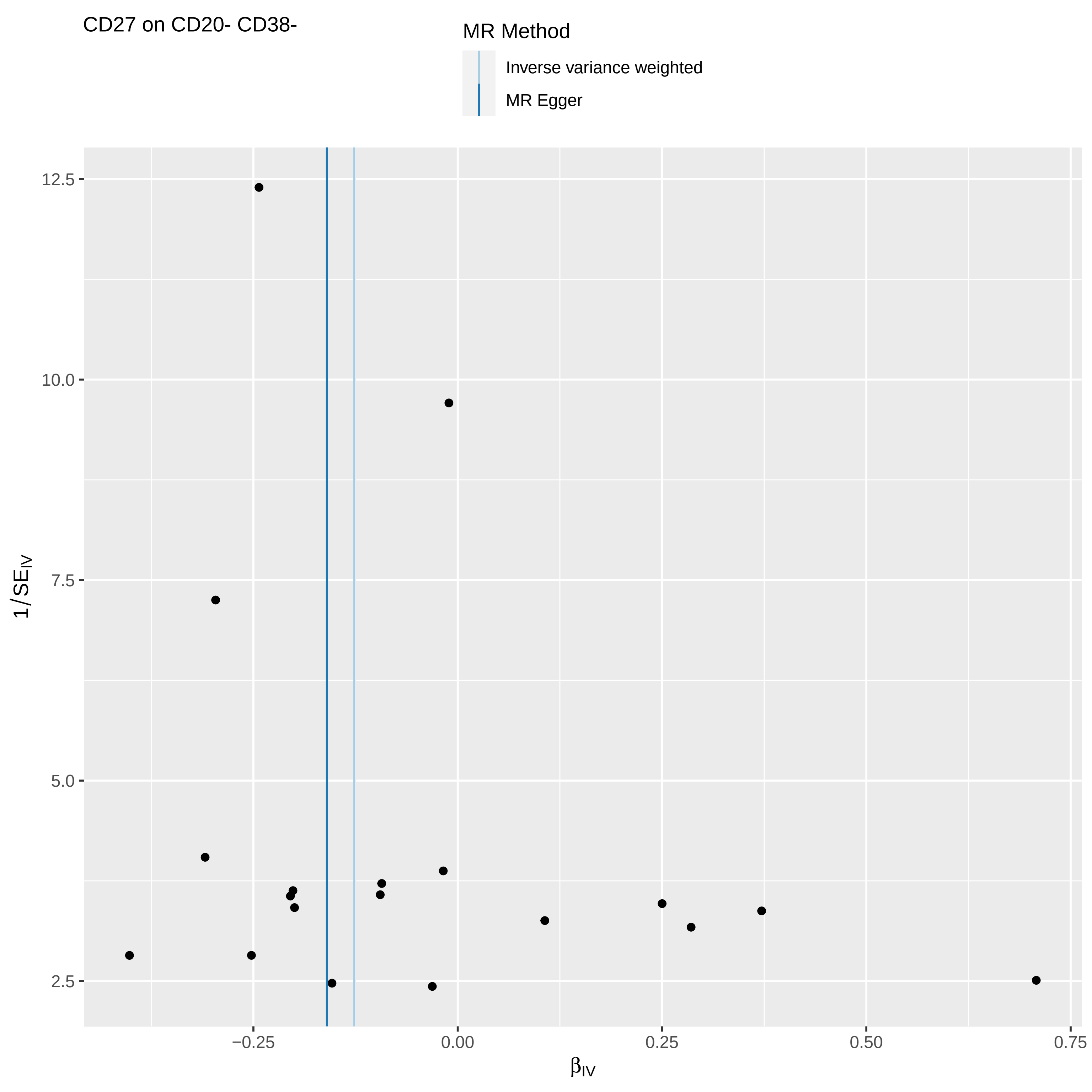
CD27 on CD20- CD38-
MR Method
Inverse variance weighted
MR Egger
12.5
10.0
7.5
5.0
2.5
1/SE_IV
−0.25
0.00
0.25
0.50
0.75
β_IV

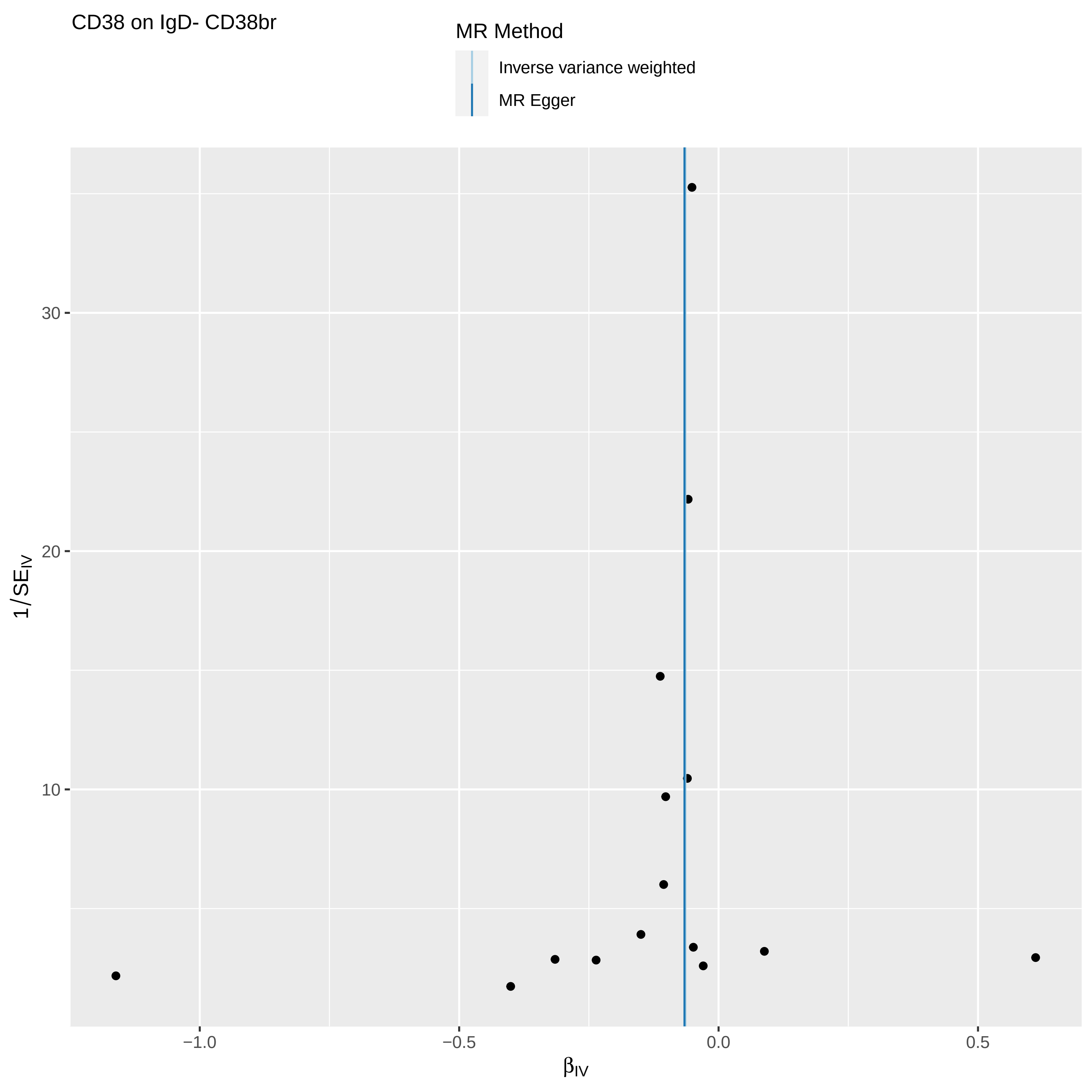
CD38 on IgD- CD38br
MR Method
Inverse variance weighted
MR Egger
30
20
10
1/SE_IV
−1.0
−0.5
0.0
0.5
β_IV

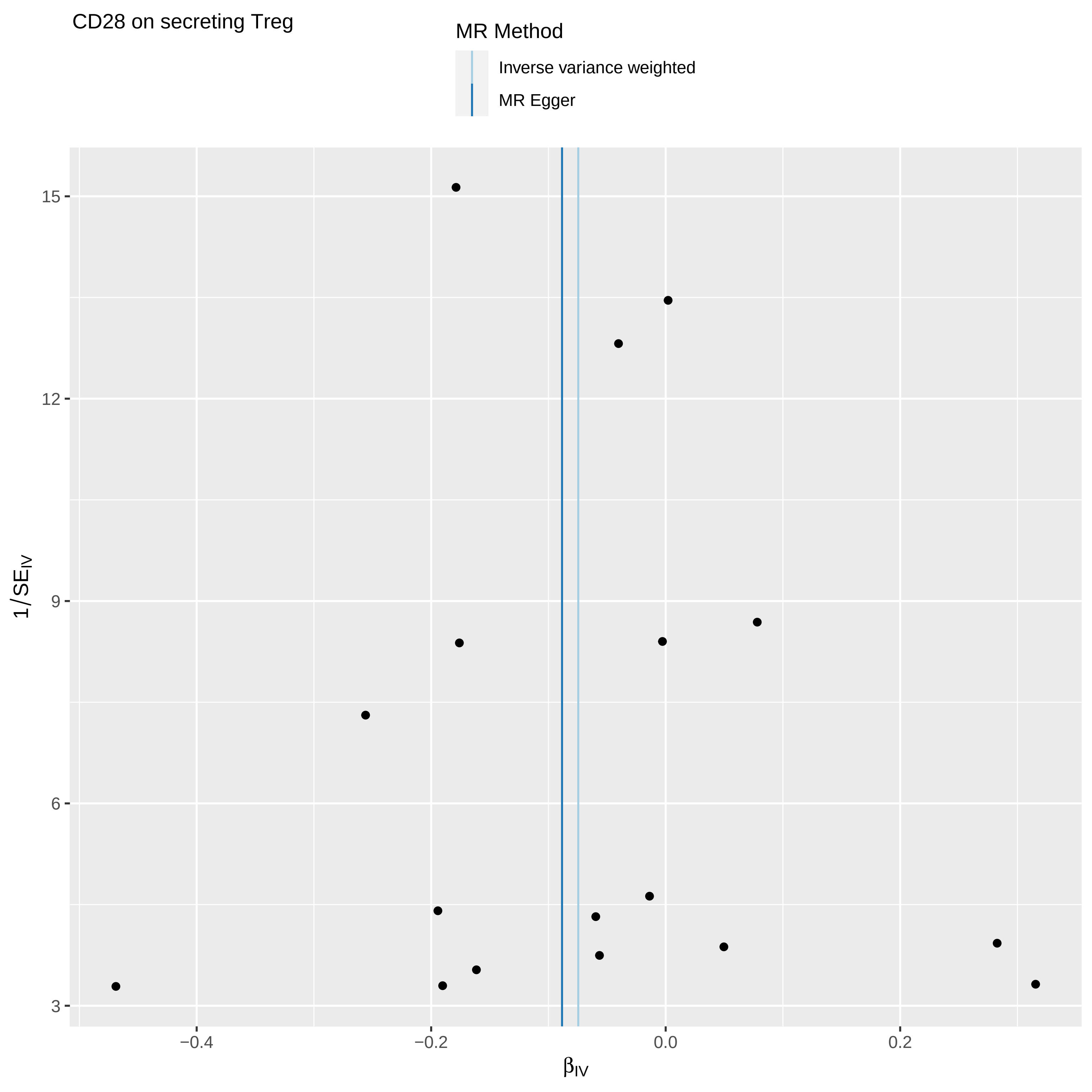

CD28 on secreting Treg
MR Method
Inverse variance weighted
MR Egger
15
12
9
6
3
1/SE_IV
−0.4
−0.2
0.0
0.2
β_IV

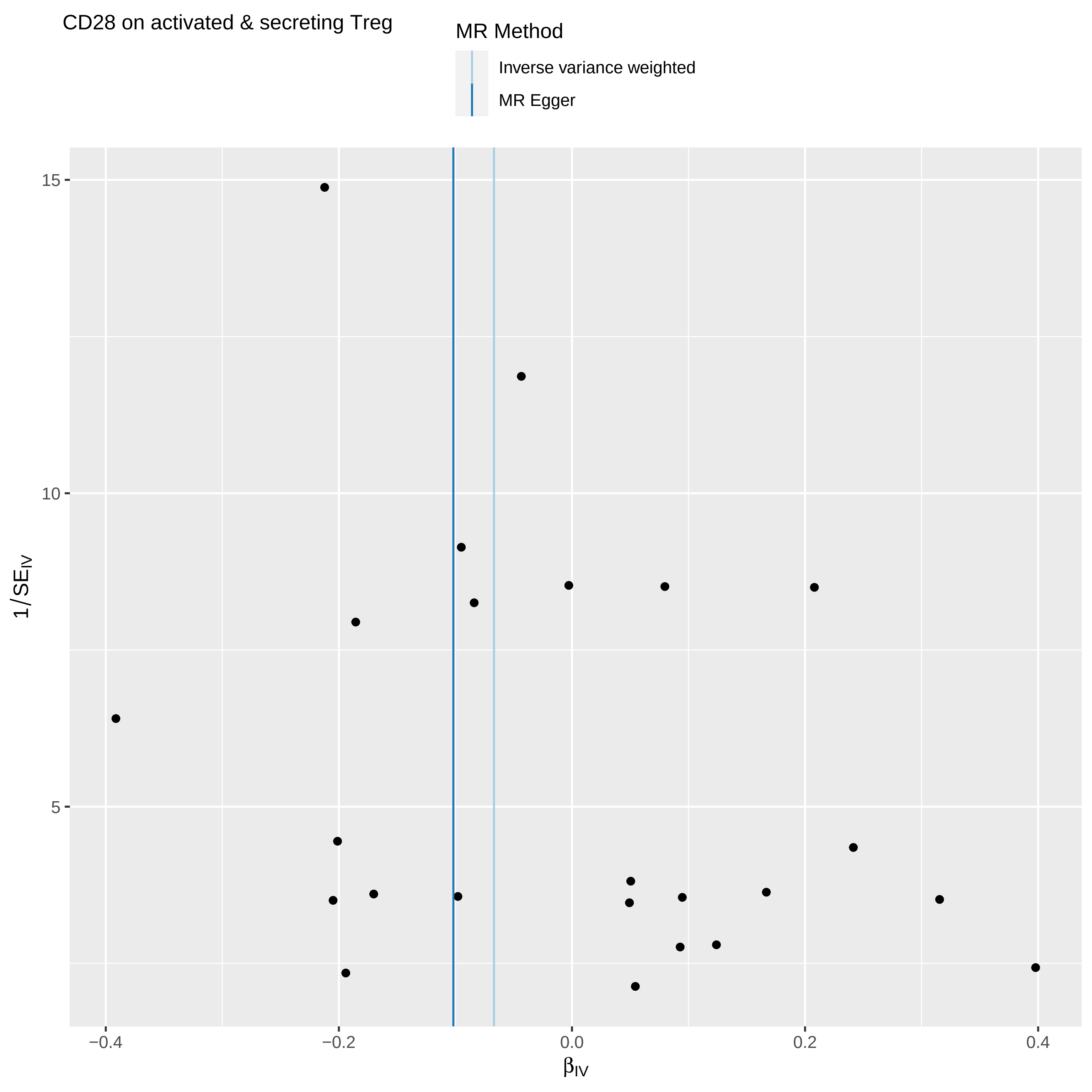

CD28 on activated & secreting Treg
MR Method
Inverse variance weighted
MR Egger
15
10
5
1/SE_IV
−0.4
−0.2
0.0
0.2
0.4
β_IV

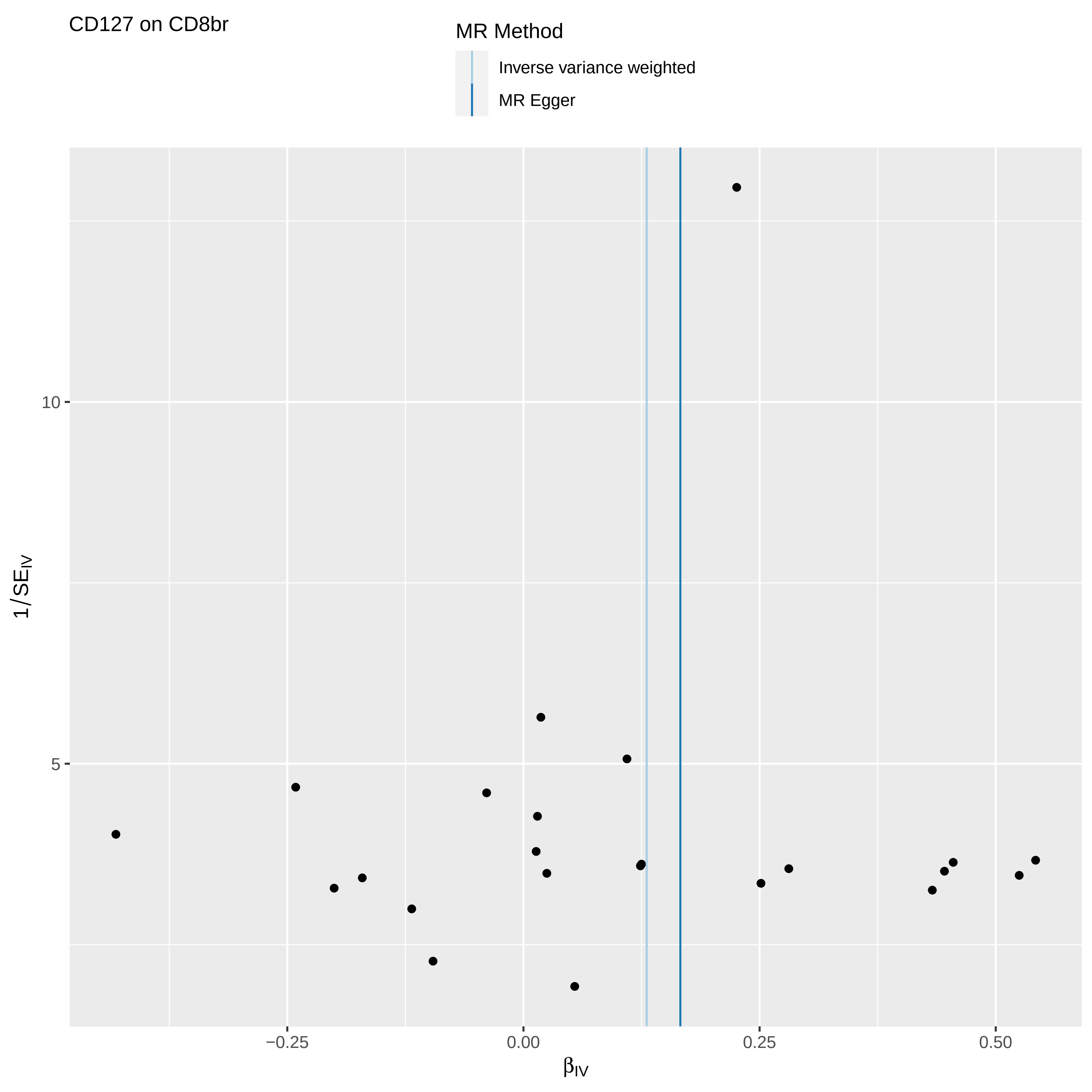
CD127 on CD8br
MR Method
Inverse variance weighted
MR Egger
10
5
1/SE_IV
−0.25
0.00
0.25
0.50
β_IV

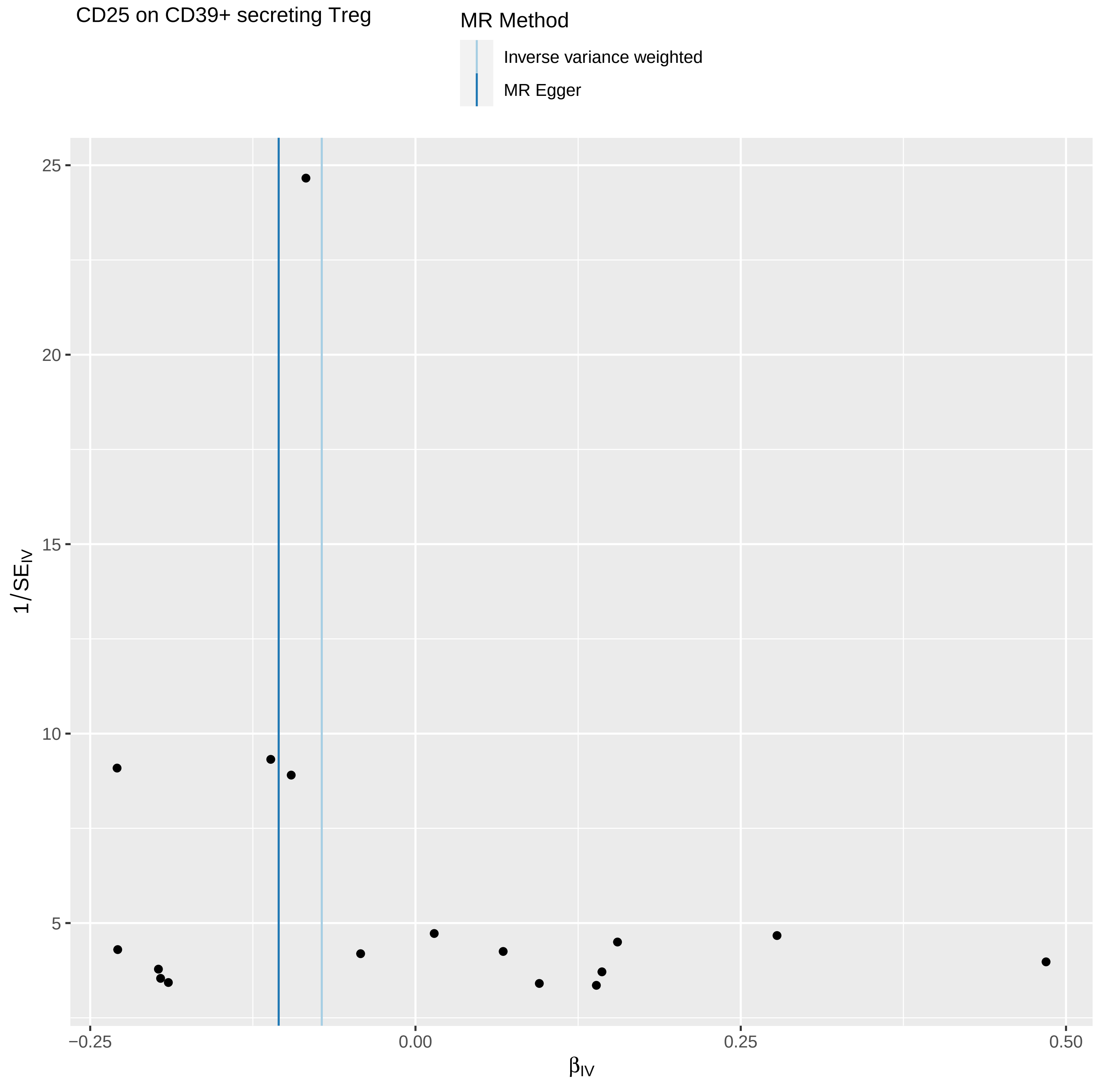
CD25 on CD39+ secreting Treg
MR Method
Inverse variance weighted
MR Egger
25
20
15
10
5
1/SE_IV
−0.25
0.00
0.25
0.50
β_IV

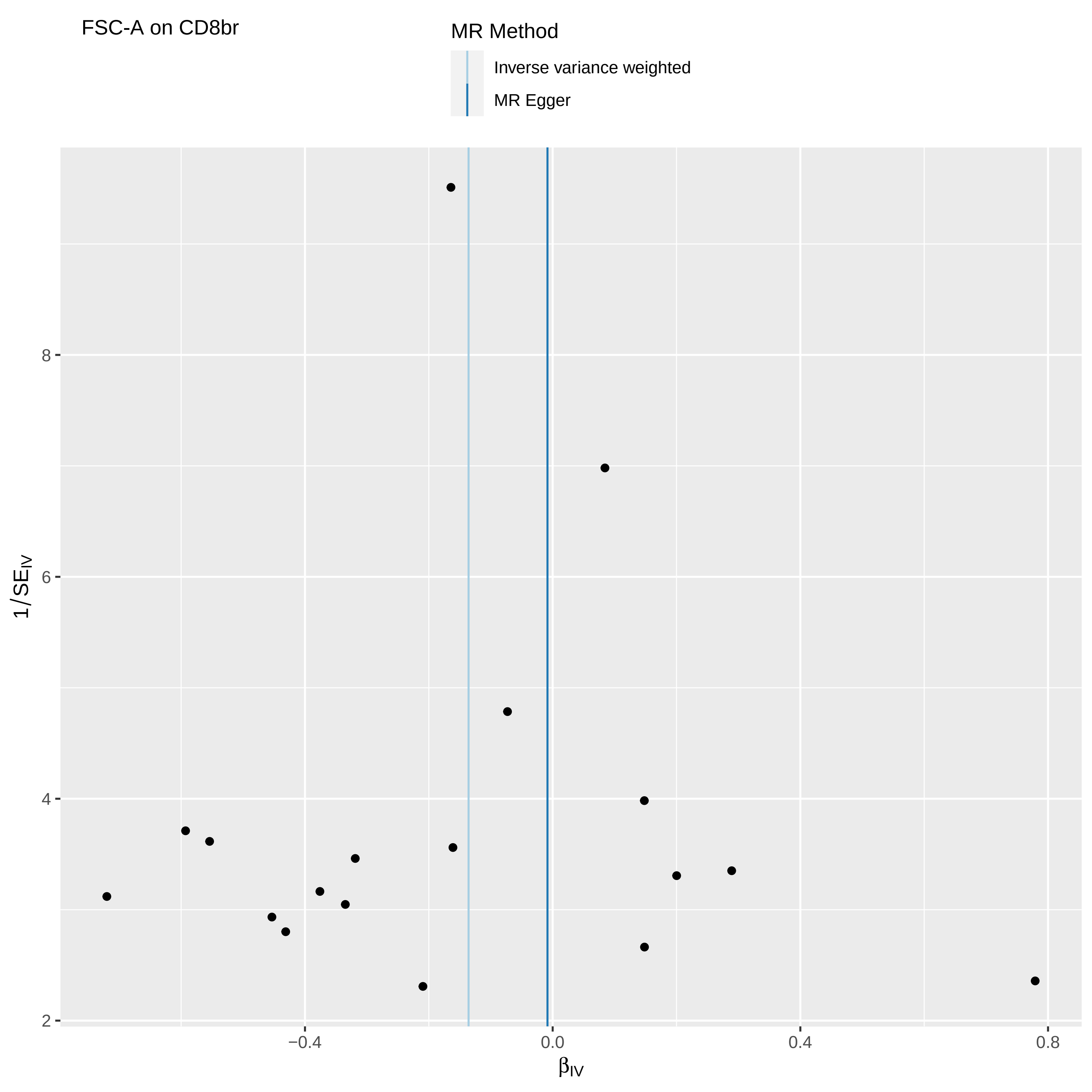

FSC-A on CD8br
MR Method
Inverse variance weighted
MR Egger
1/SE_IV
β_IV
−0.4
0.0
0.4
0.8
2
4
6
8

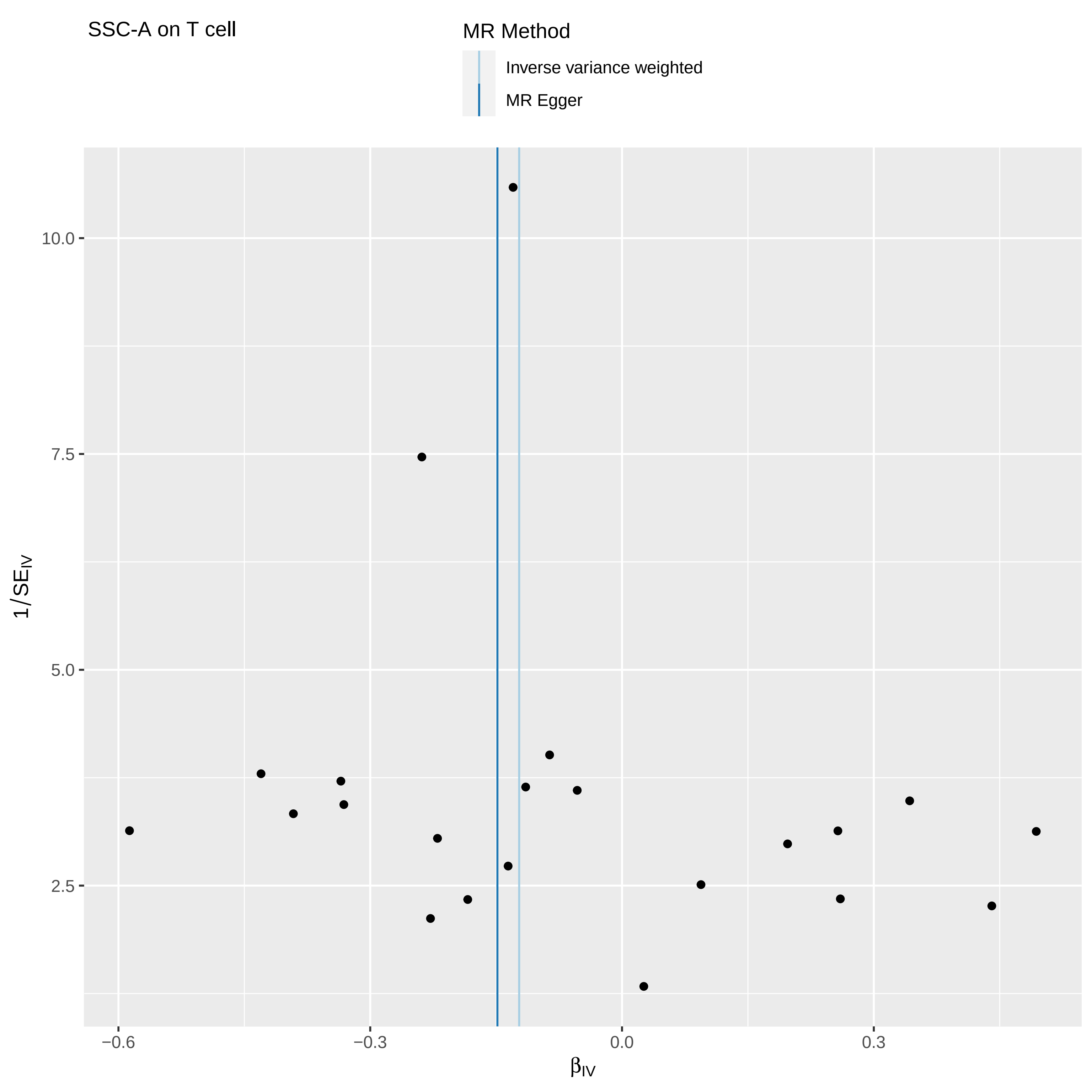

SSC-A on T cell
MR Method
Inverse variance weighted
MR Egger
10.0
7.5
5.0
2.5
1/SE_IV
−0.6
−0.3
0.0
0.3
β_IV

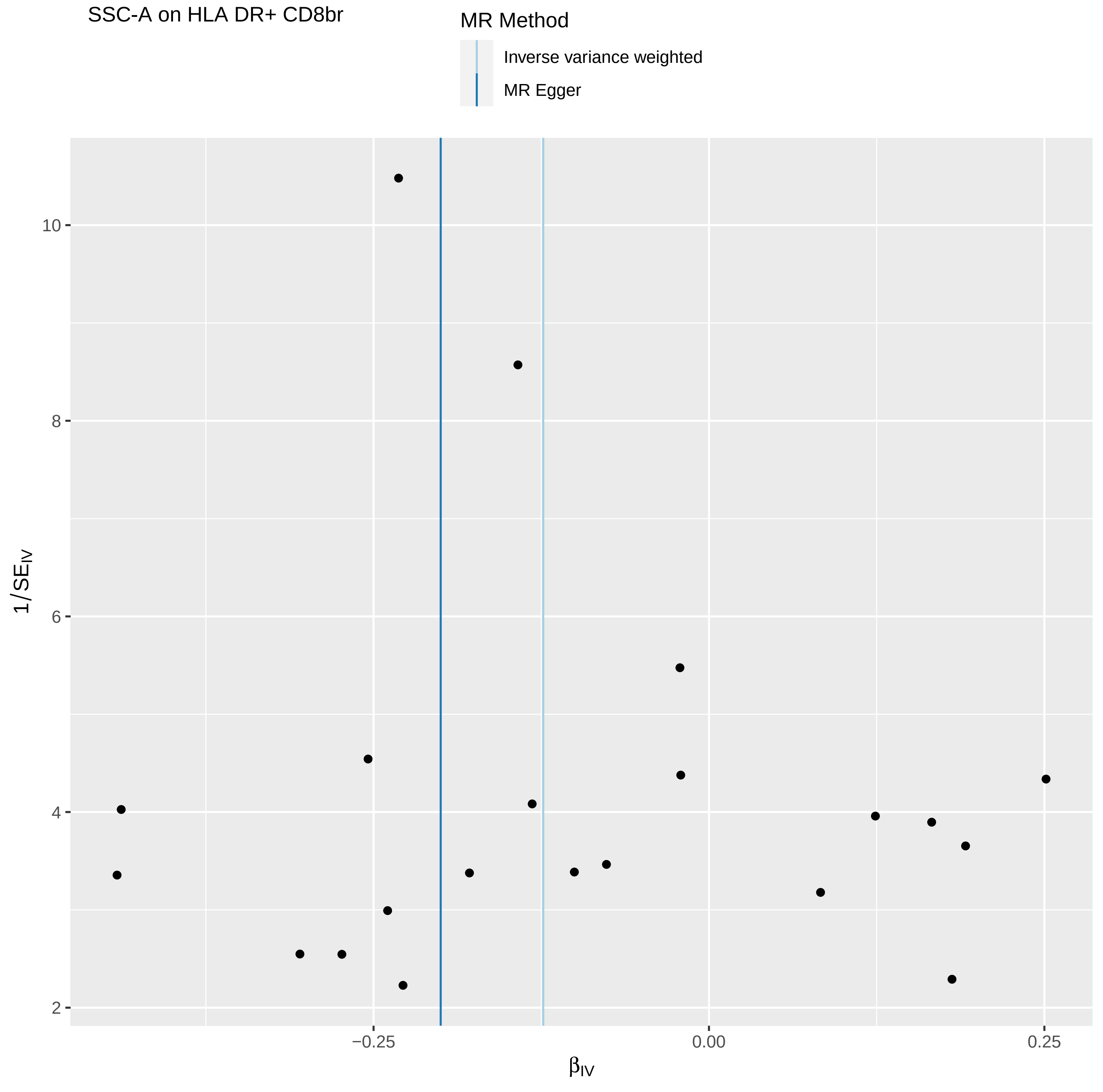
SSC-A on HLA DR+ CD8br
MR Method
Inverse variance weighted
MR Egger
10
8
6
4
2
1/SE_IV
−0.25
0.00
0.25
β_IV

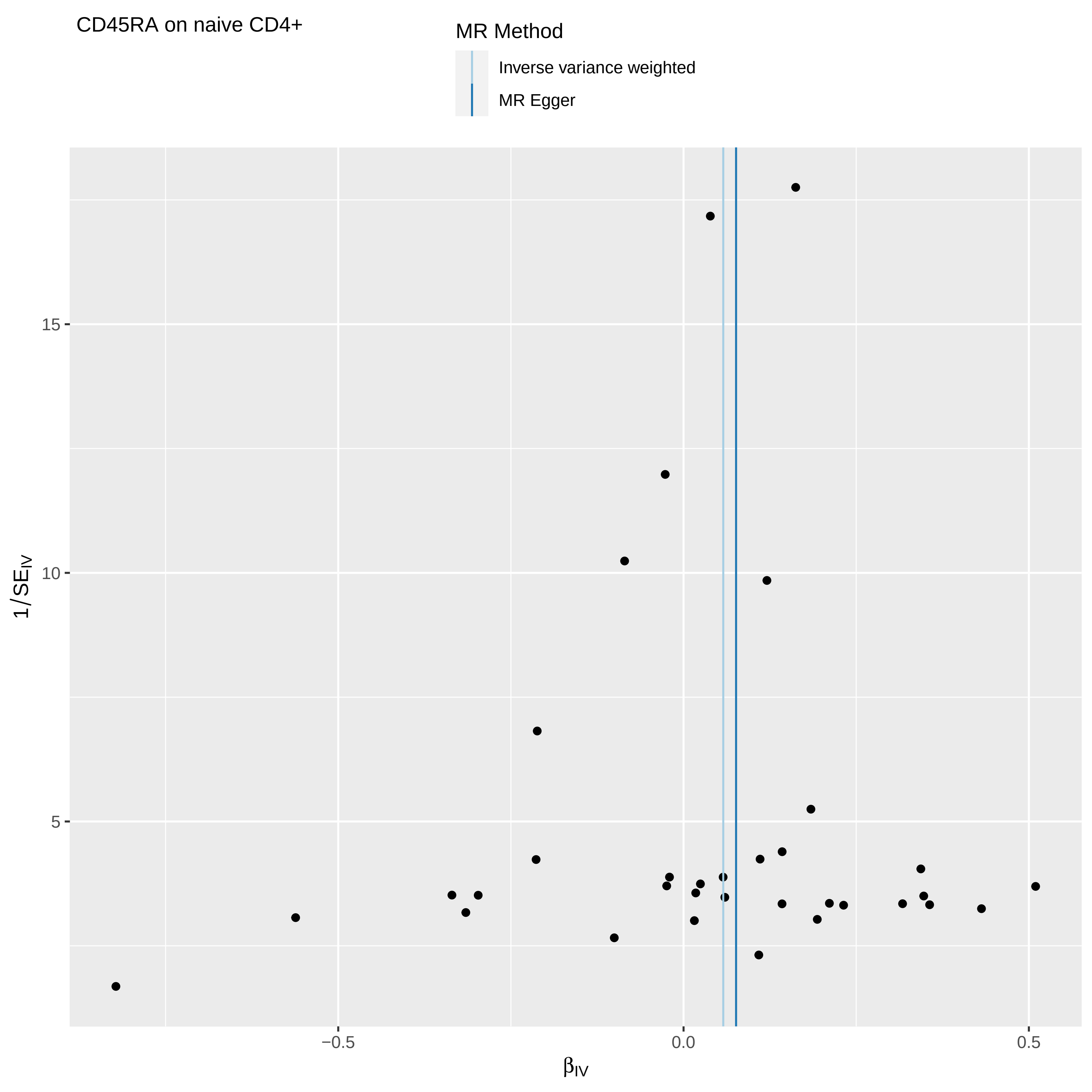
CD45RA on naive CD4+
MR Method
Inverse variance weighted
MR Egger
15
10
5
1/SE$_{IV}$
−0.5
0.0
0.5
β$_{IV}$

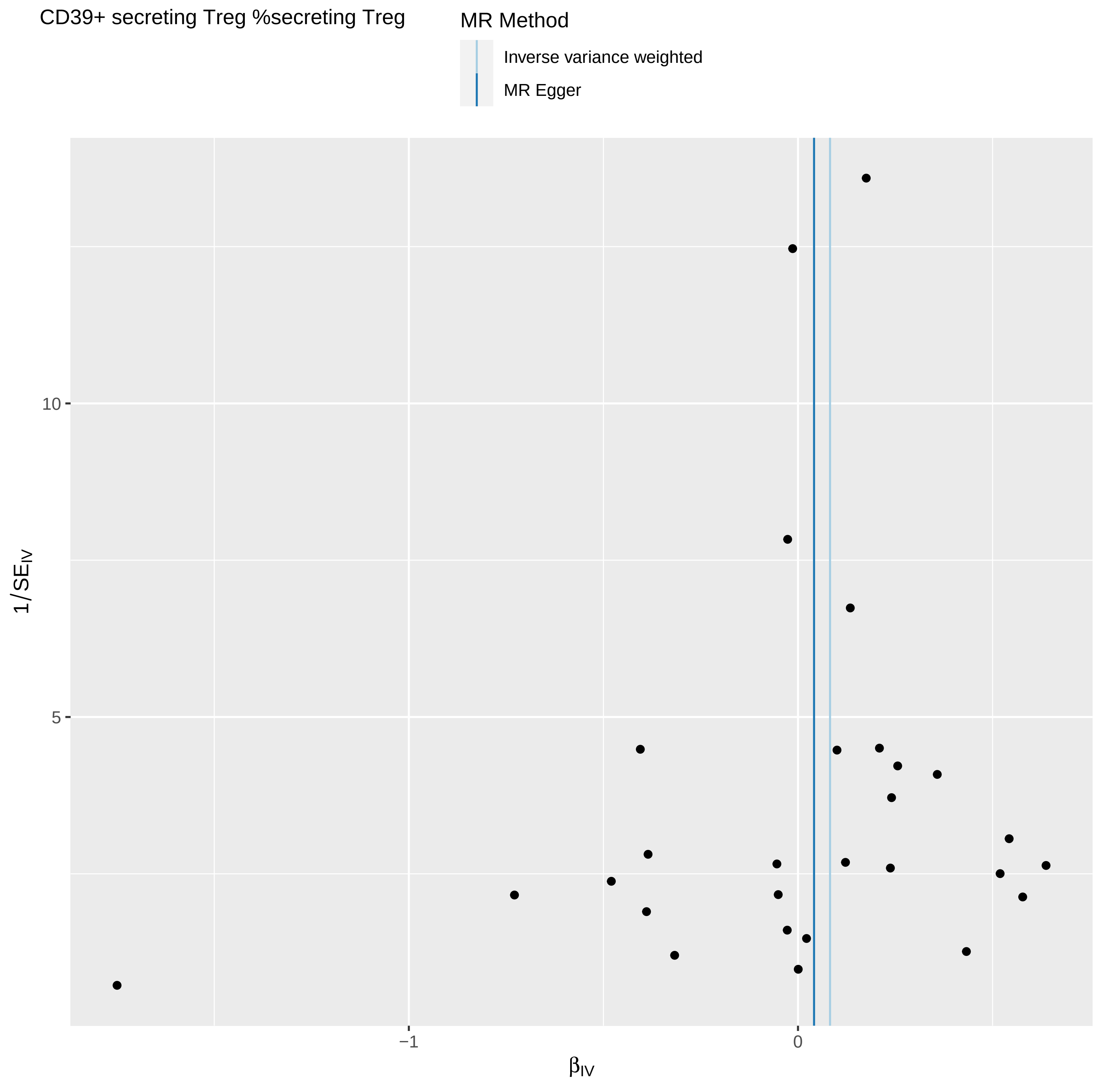

CD39+ secreting Treg %secreting Treg
MR Method
Inverse variance weighted
MR Egger
10
5
1/SE_IV
−1
0
β_IV

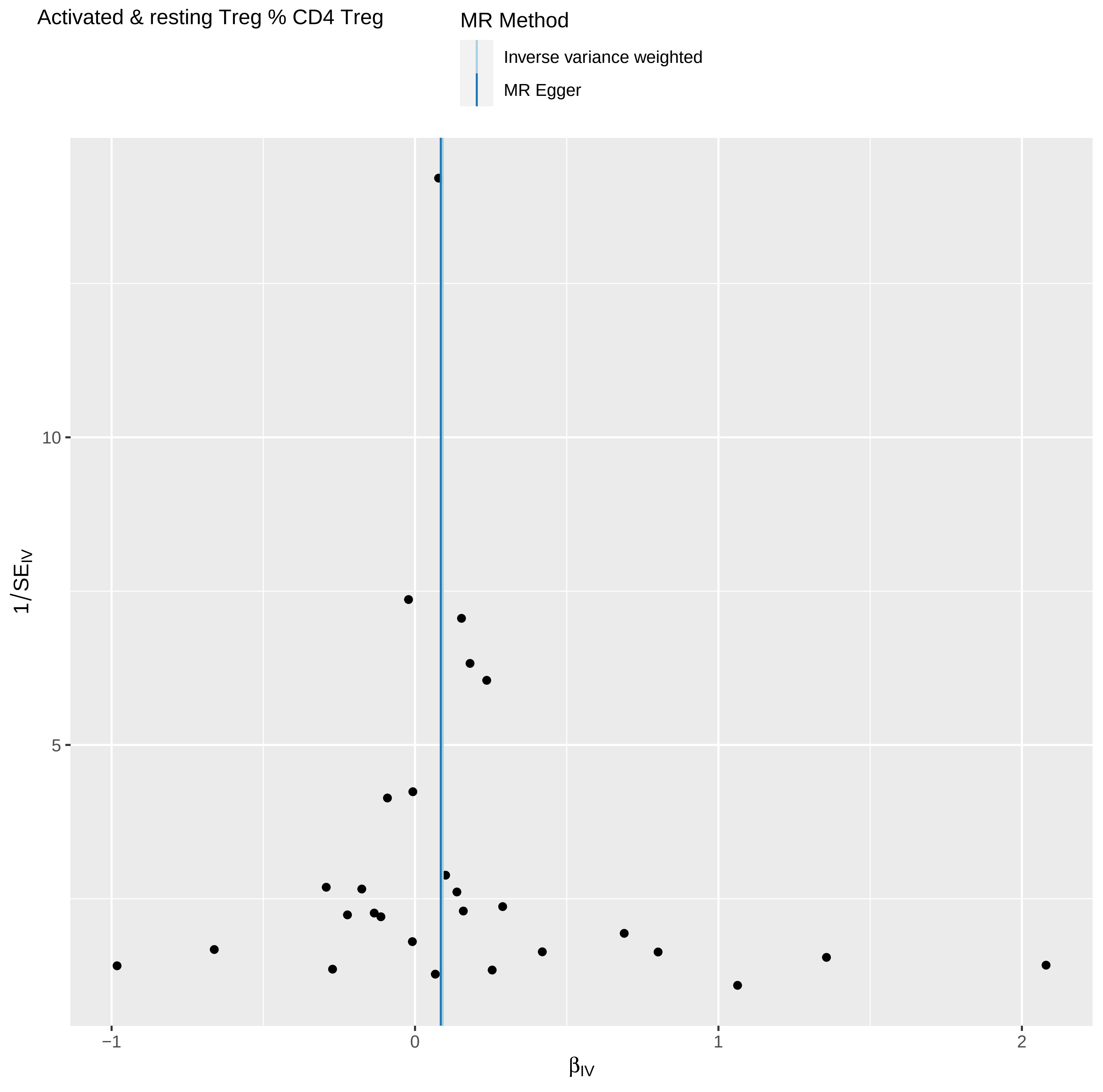
Activated & resting Treg % CD4 Treg
MR Method
Inverse variance weighted
MR Egger
10
5
$1/SE_{IV}$
−1
0
1
2
β_{IV}

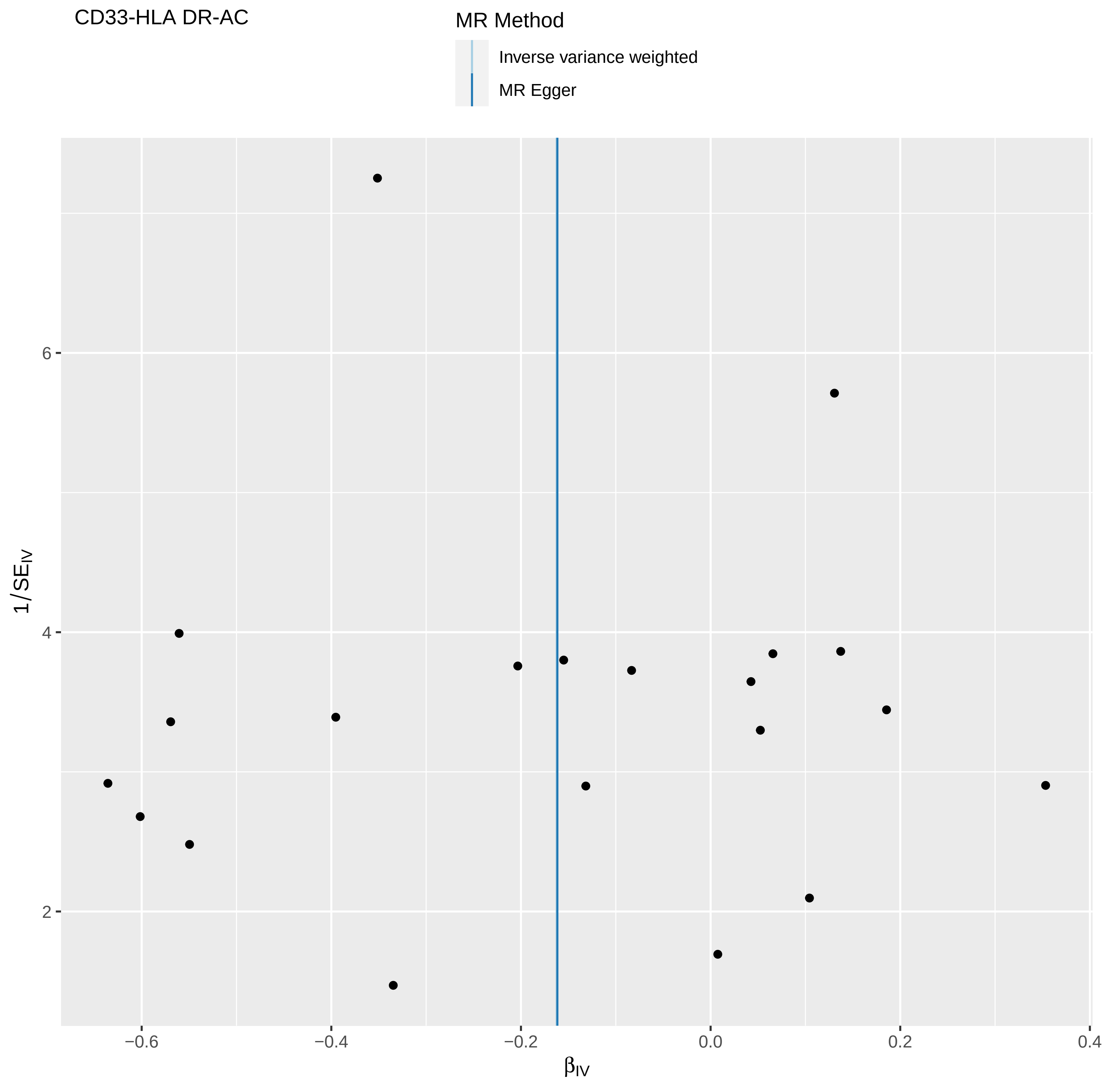
CD33-HLA DR-AC
MR Method
Inverse variance weighted
MR Egger
6
4
2
$1/SE_{IV}$
−0.6
−0.4
−0.2
0.0
0.2
0.4
β_{IV}

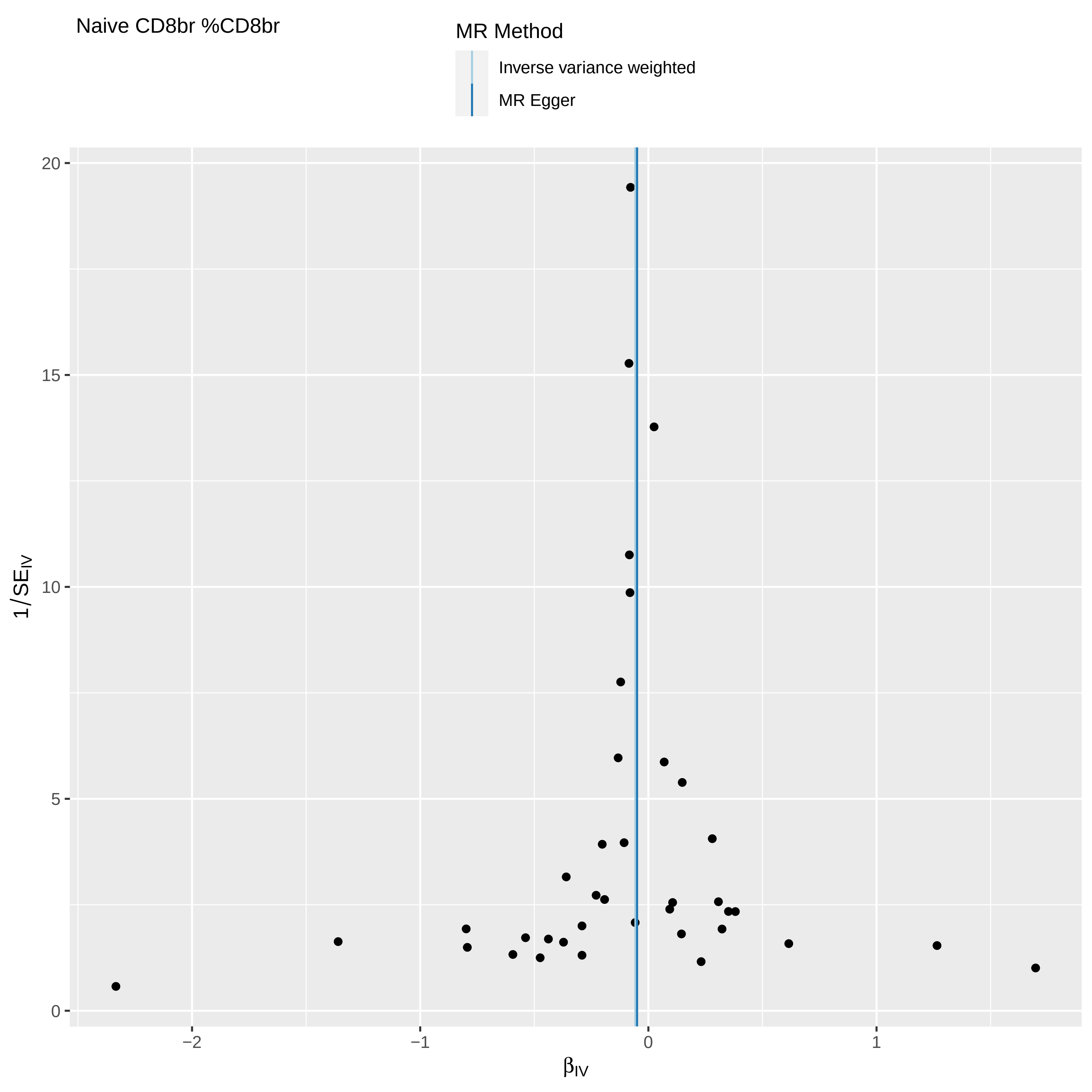
Naive CD8br %CD8br
MR Method
Inverse variance weighted
MR Egger
20
15
10
5
0
1/SE_IV
−2
−1
0
1
β_IV

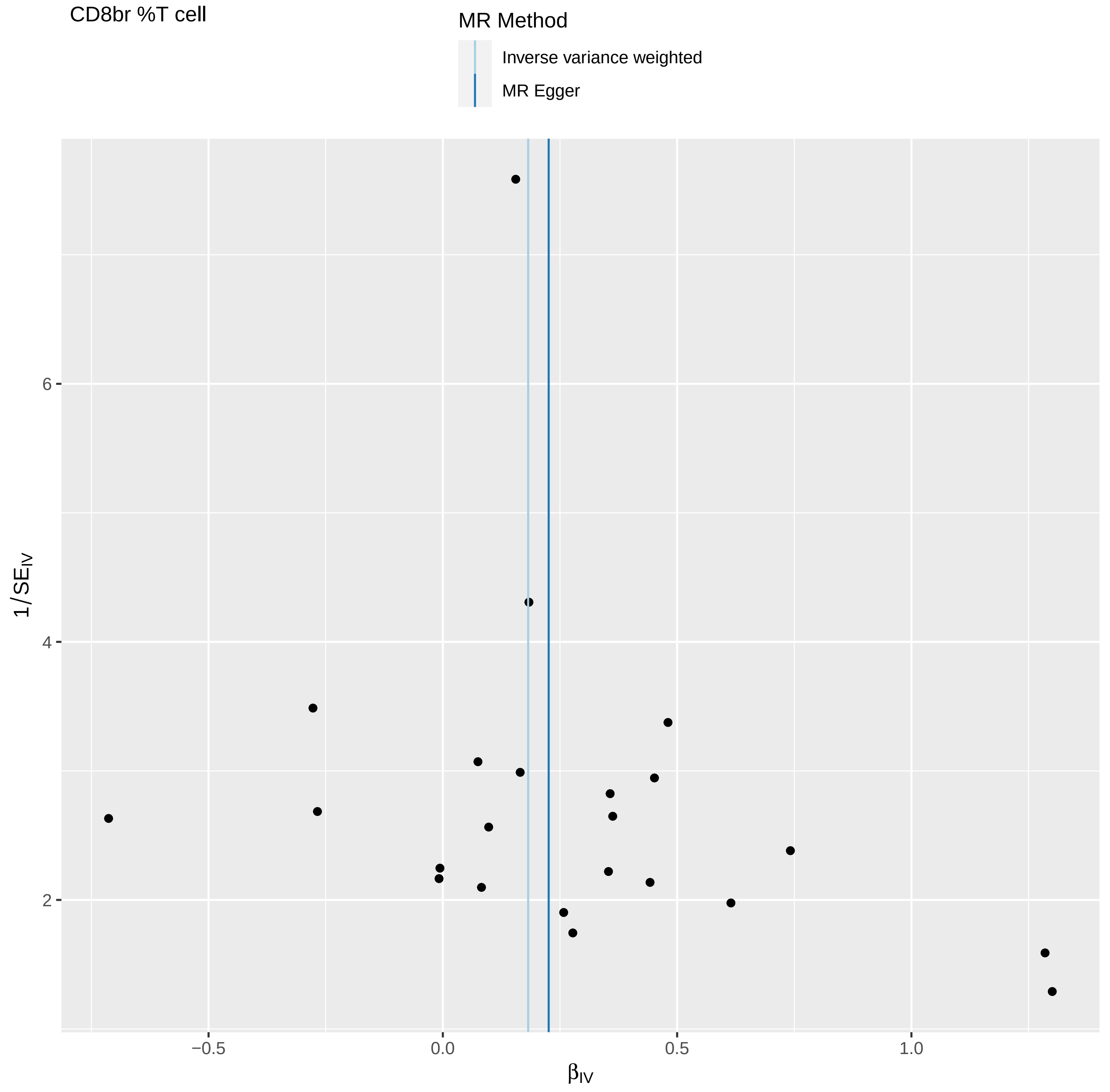
CD8br %T cell
MR Method
Inverse variance weighted
MR Egger
1/SE_IV
6
4
2
−0.5
0.0
0.5
1.0
β_IV

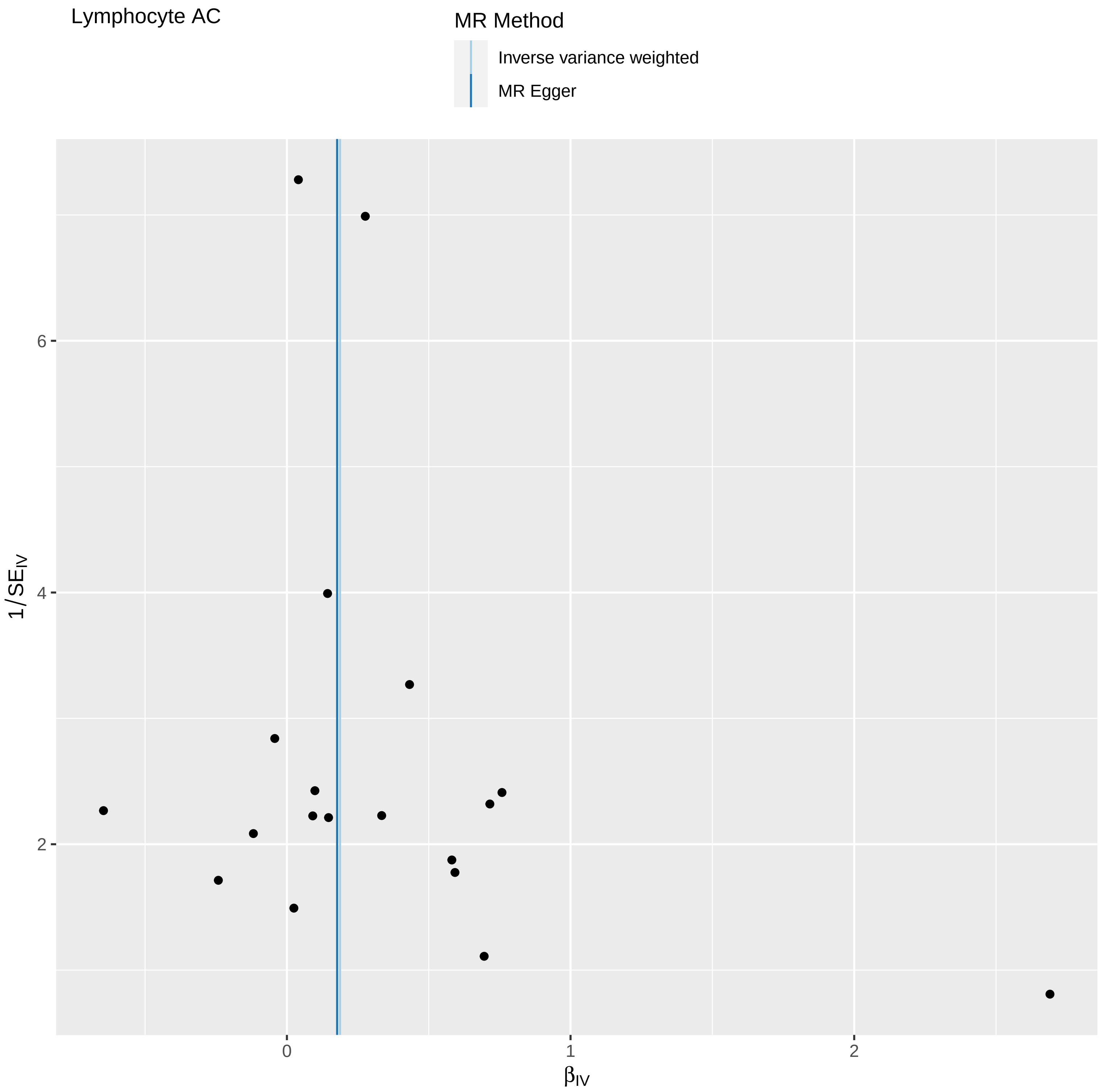

Lymphocyte AC
MR Method
Inverse variance weighted
MR Egger
6
4
2
$1/SE_{IV}$
0
1
2
β_{IV}

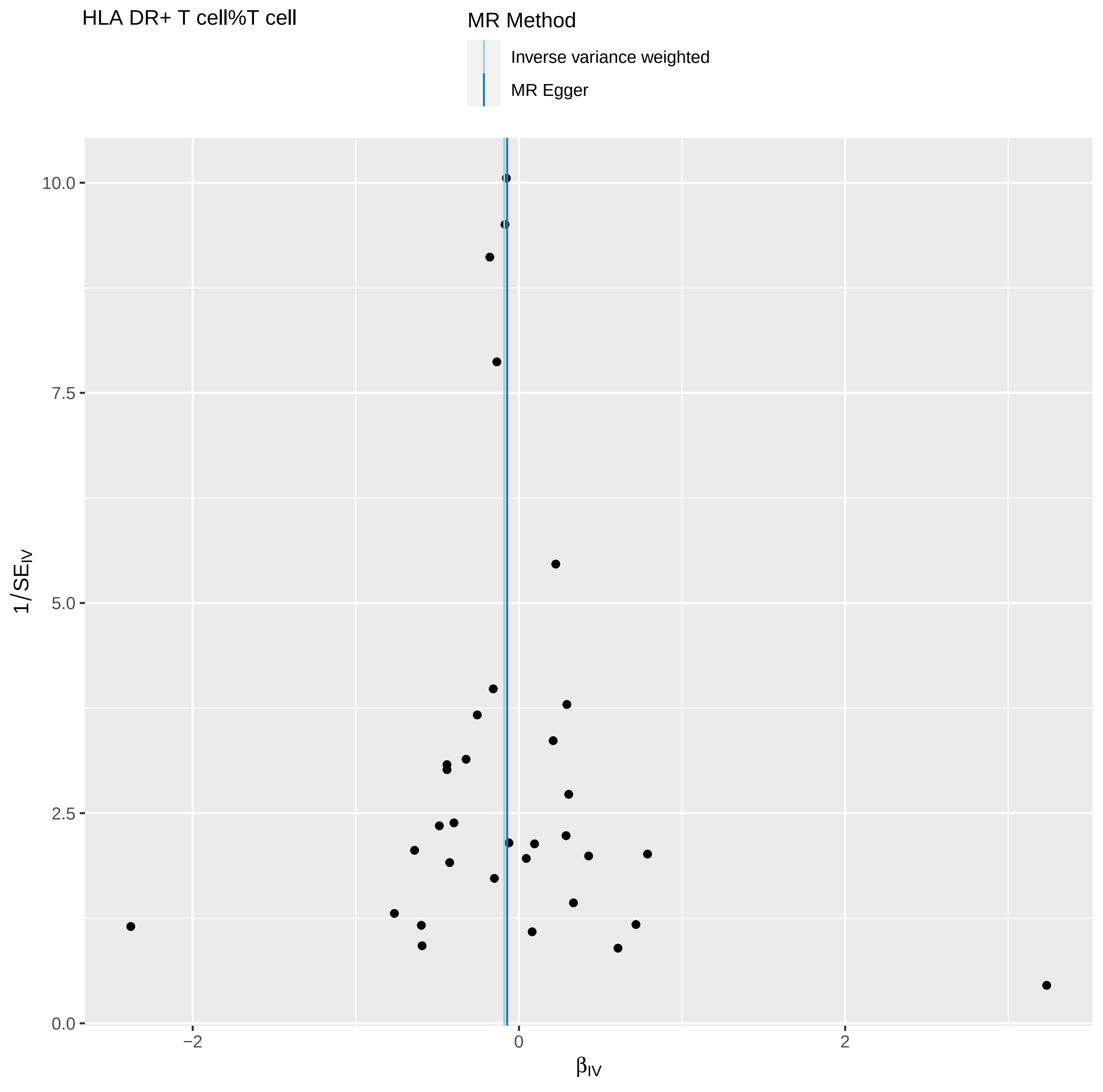

HLA DR+ T cell%T cell
MR Method
Inverse variance weighted
MR Egger
10.0
7.5
5.0
2.5
0.0
−2
0
2
$1/SE_{IV}$
β_{IV}

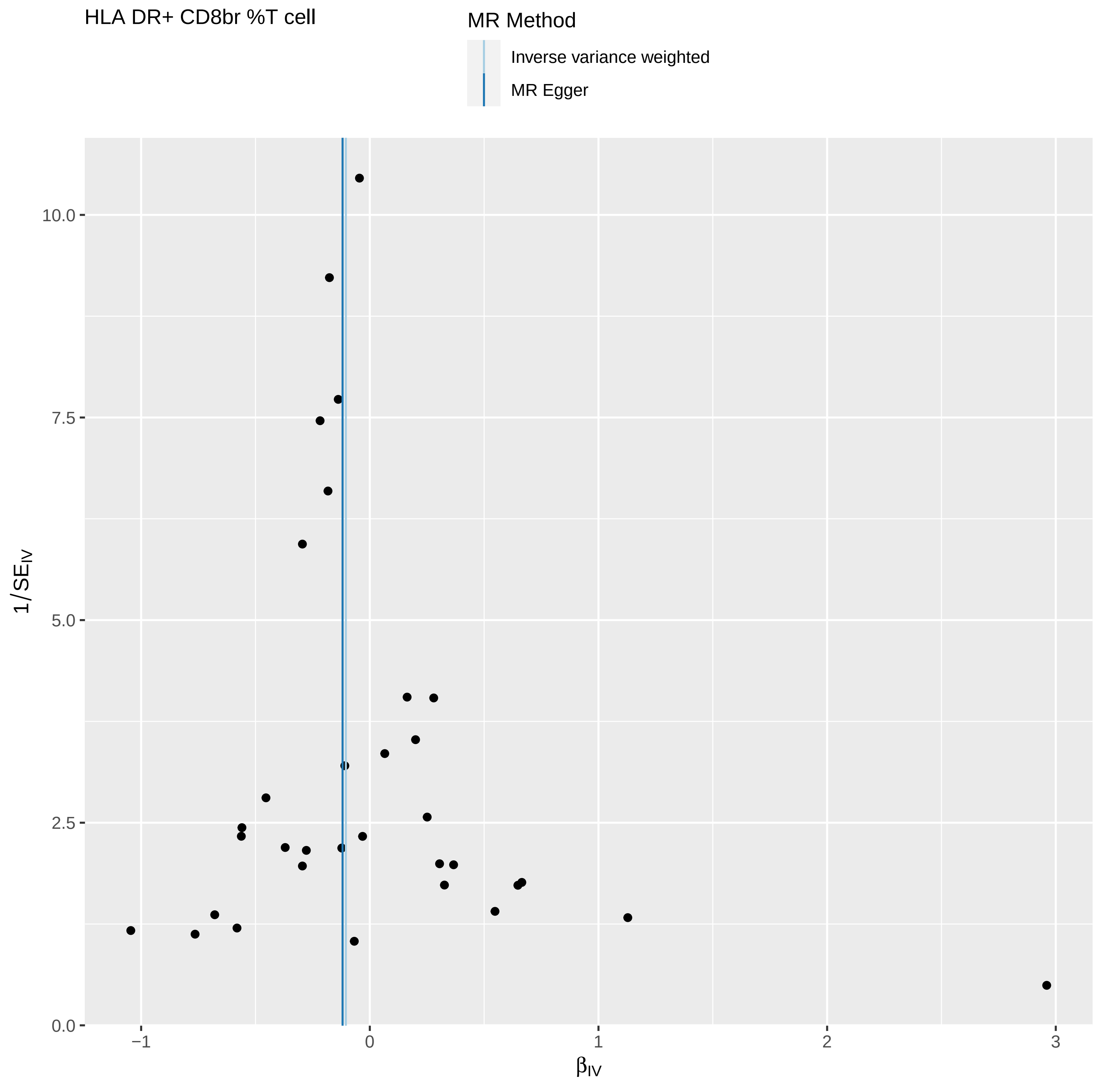
HLA DR+ CD8br %T cell
MR Method
Inverse variance weighted
MR Egger
10.0
7.5
5.0
2.5
0.0
1/SE_IV
−1
0
1
2
3
β_IV

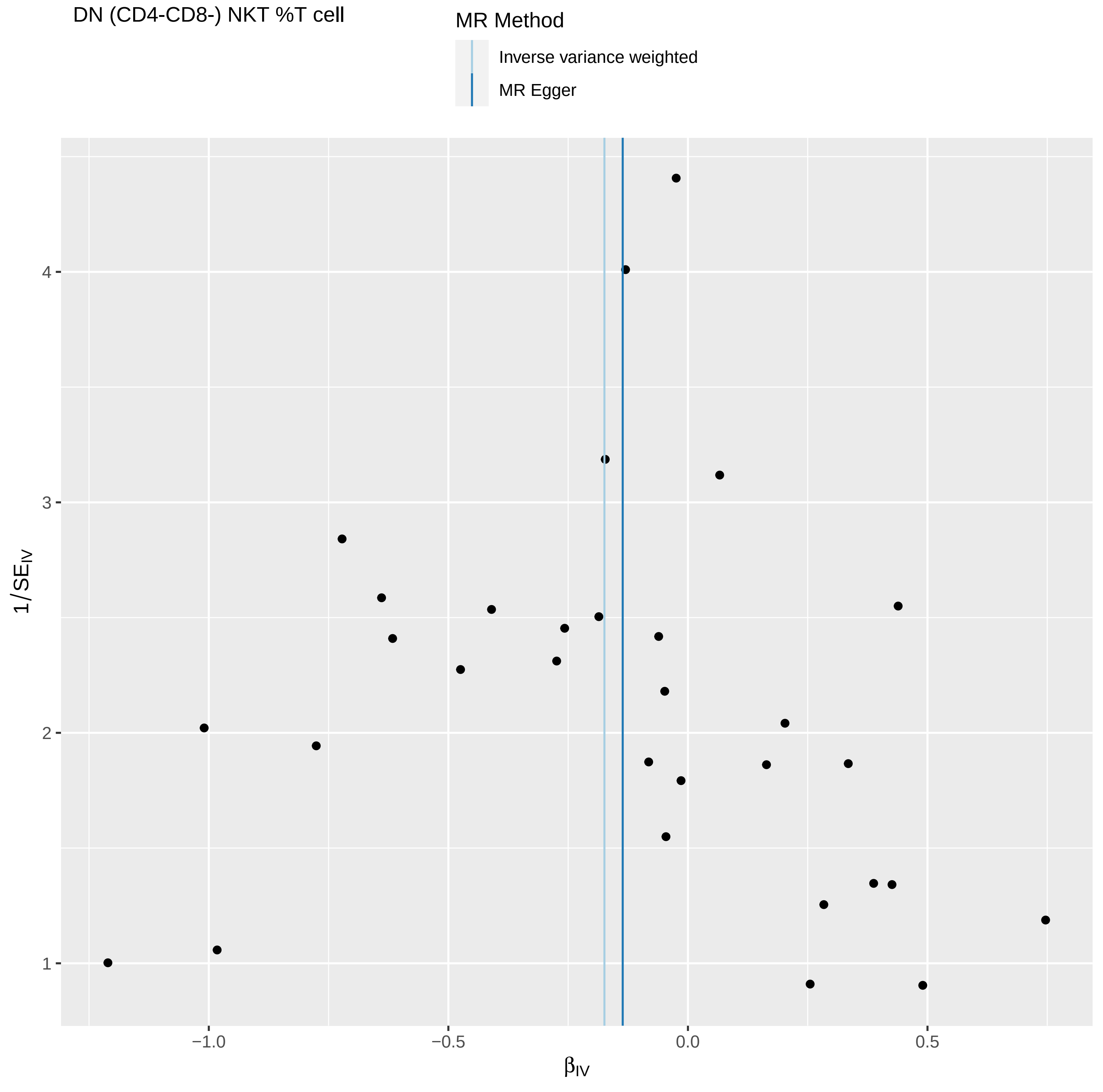
DN (CD4-CD8-) NKT %T cell
MR Method
Inverse variance weighted
MR Egger
1/SE_IV
β_IV
4
3
2
1
−1.0
−0.5
0.0
0.5

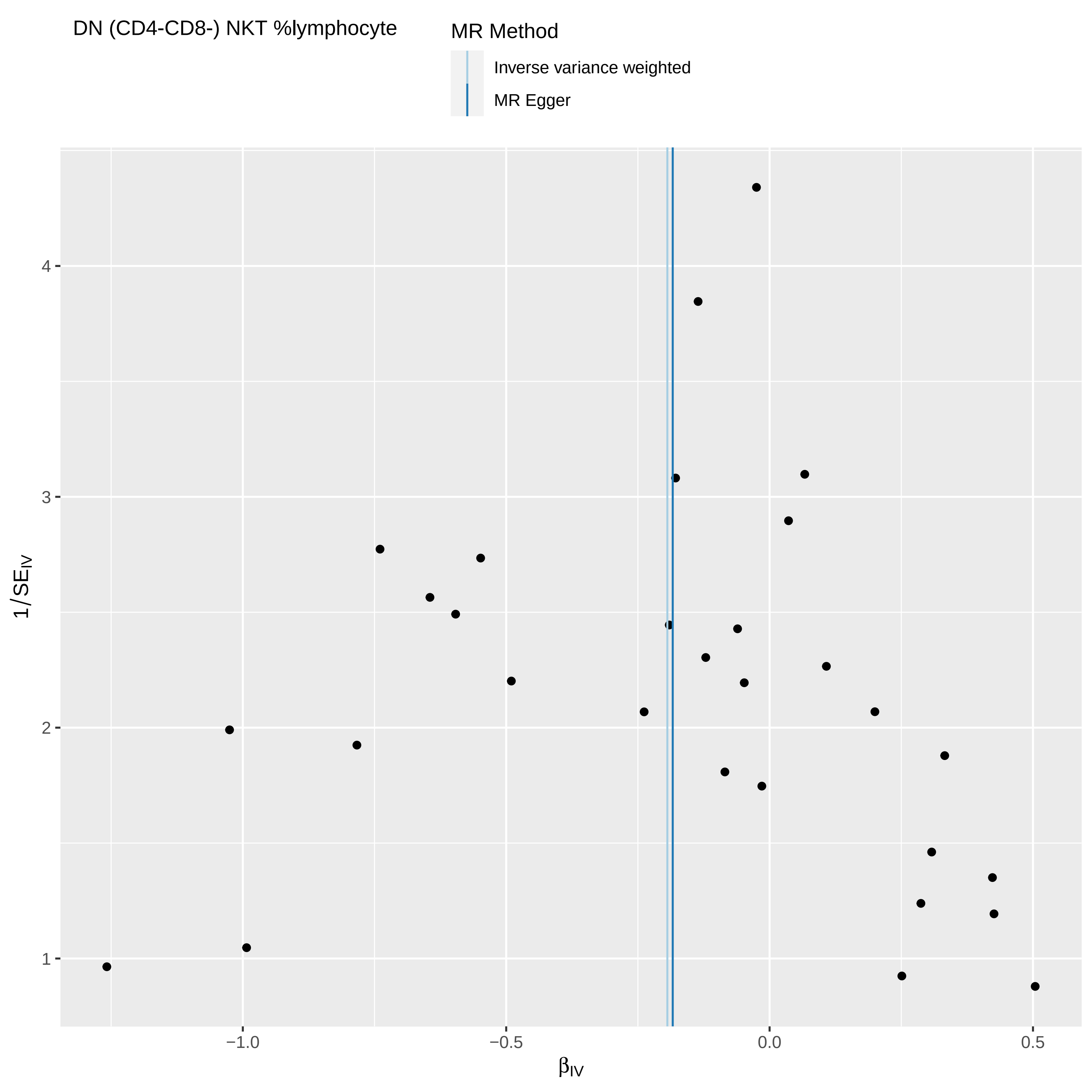

DN (CD4-CD8-) NKT %lymphocyte
MR Method
Inverse variance weighted
MR Egger
1/SE_IV
β_IV
4
3
2
1
−1.0
−0.5
0.0
0.5

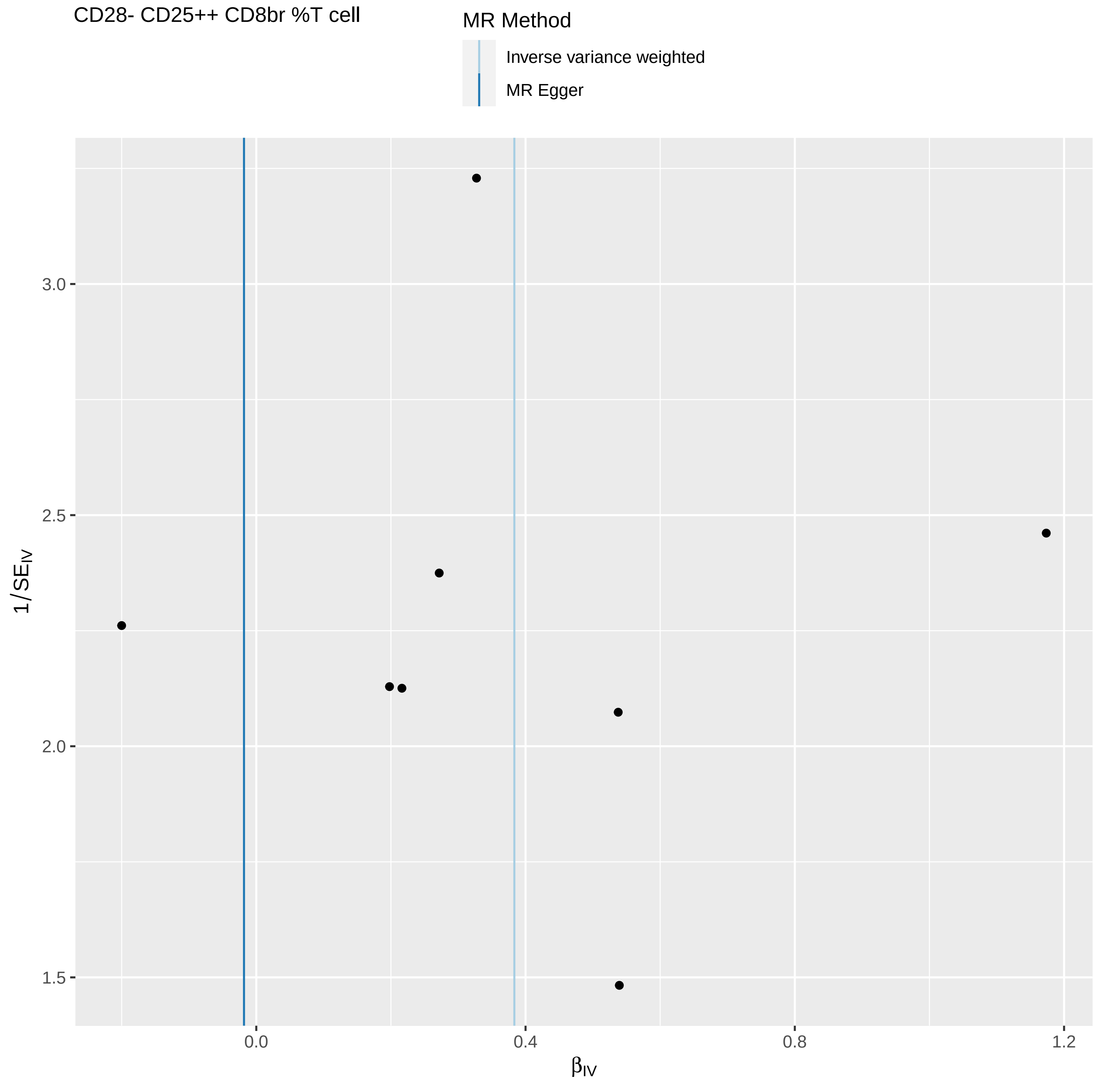

CD28- CD25++ CD8br %T cell
MR Method
Inverse variance weighted
MR Egger
3.0
2.5
2.0
1.5
$1/SE_{IV}$
0.0
0.4
0.8
1.2
β_{IV}

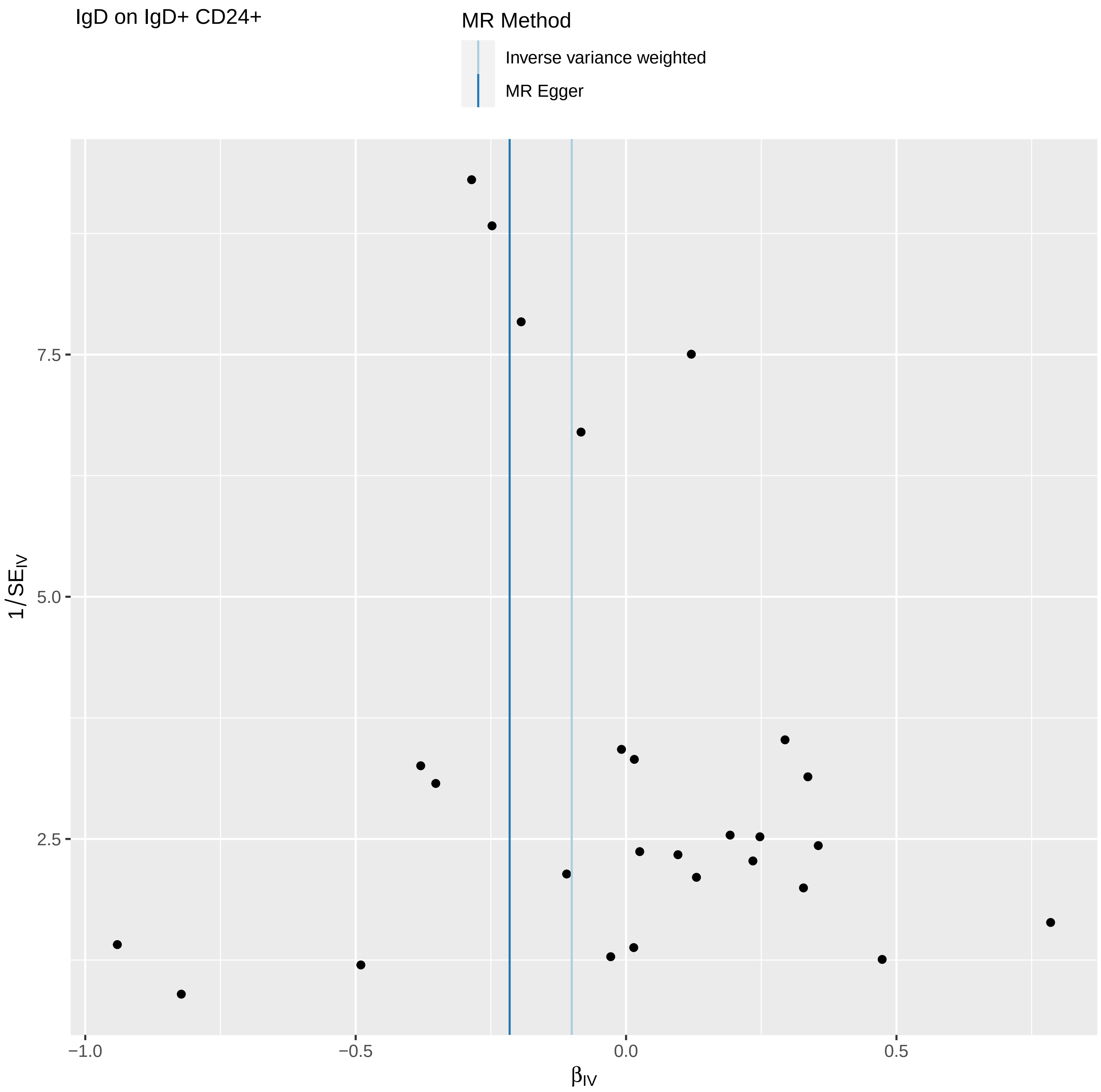
IgD on IgD+ CD24+
MR Method
Inverse variance weighted
MR Egger
7.5
5.0
2.5
1/SE_{IV}
−1.0
−0.5
0.0
0.5
β_{IV}

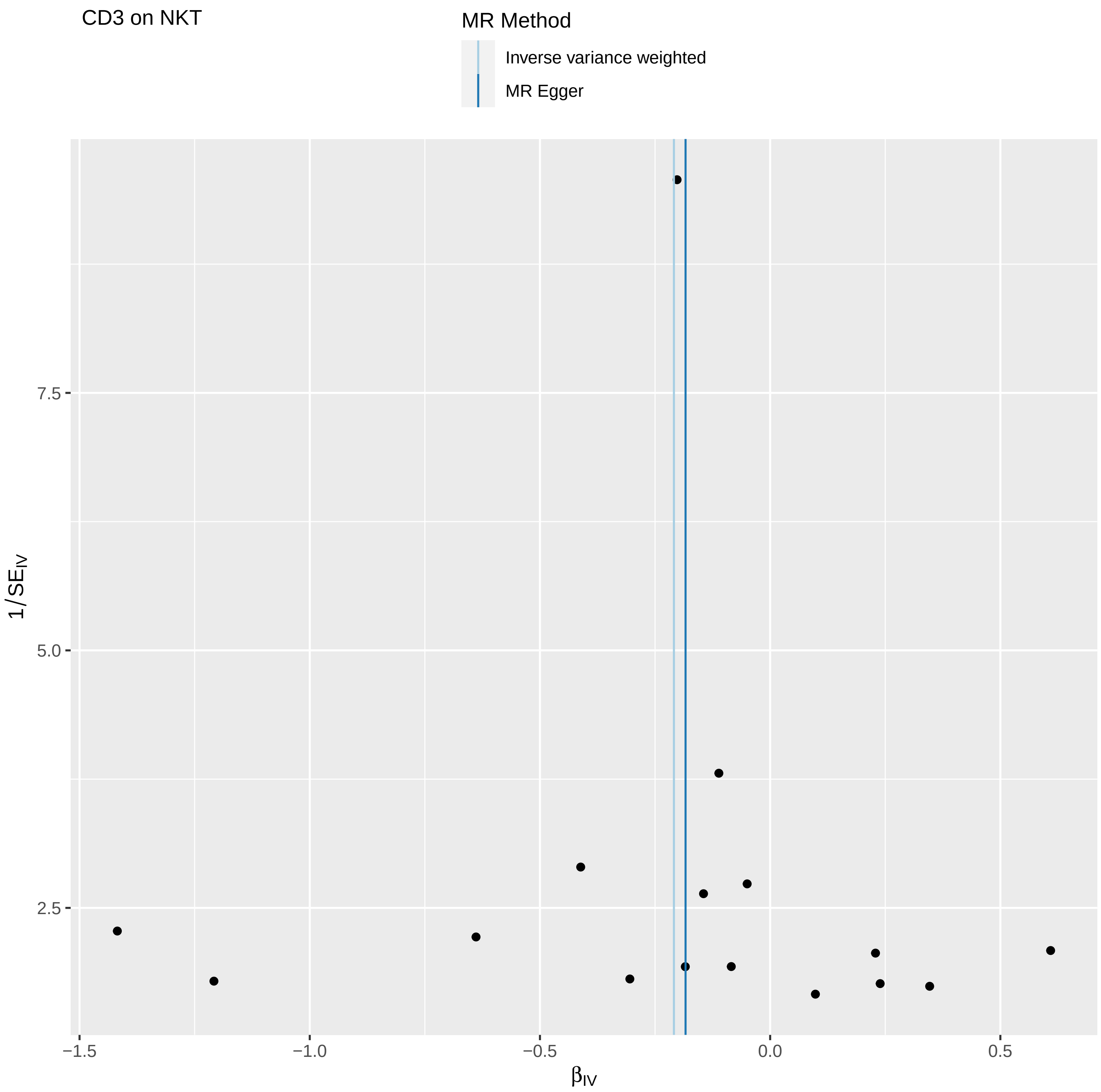
CD3 on NKT
MR Method
Inverse variance weighted
MR Egger
7.5
5.0
2.5
−1.5
−1.0
−0.5
0.0
0.5
$1/SE_{IV}$
β_{IV}

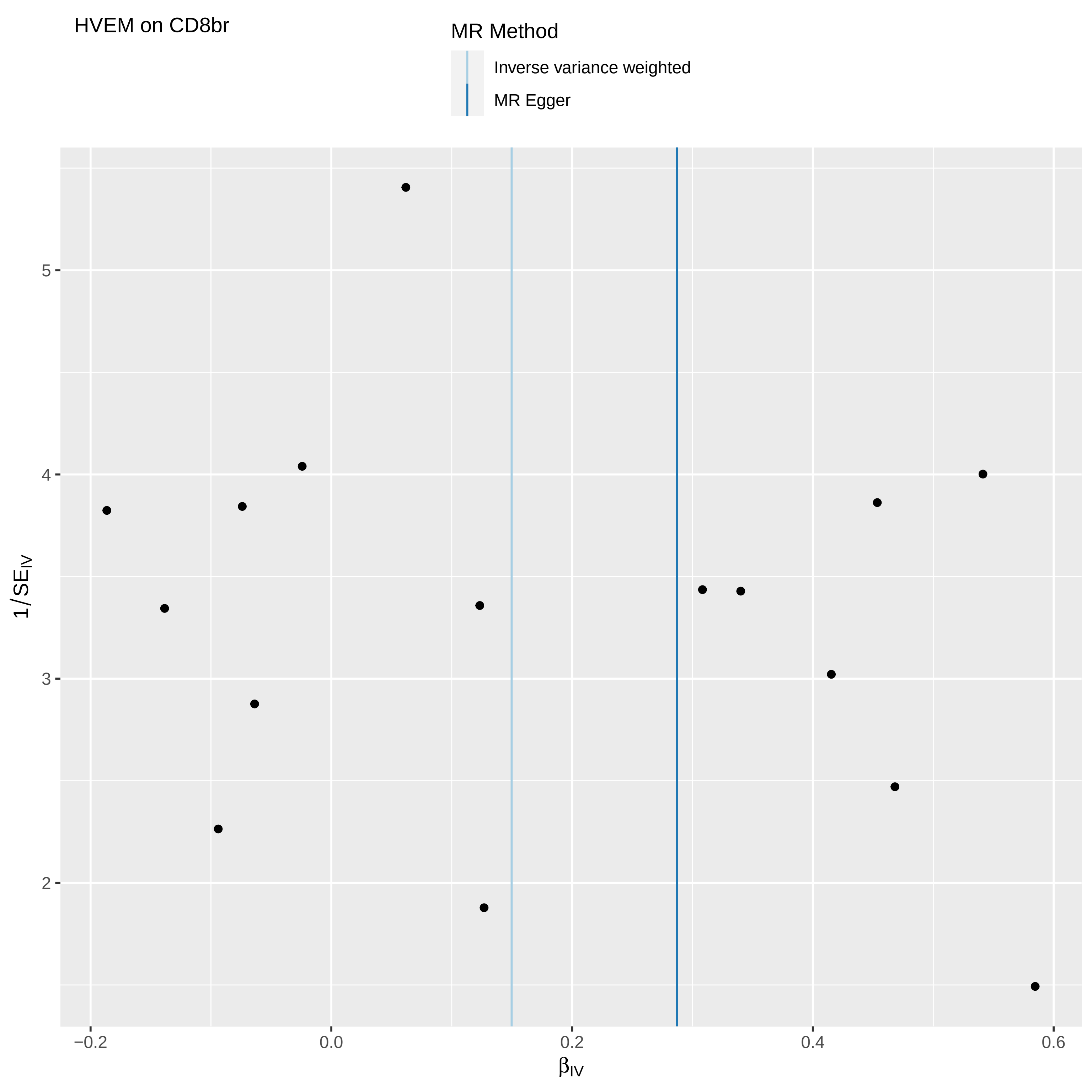
HVEM on CD8br
MR Method
Inverse variance weighted
MR Egger
1/SE_IV
β_IV
5
4
3
2
−0.2
0.0
0.2
0.4
0.6

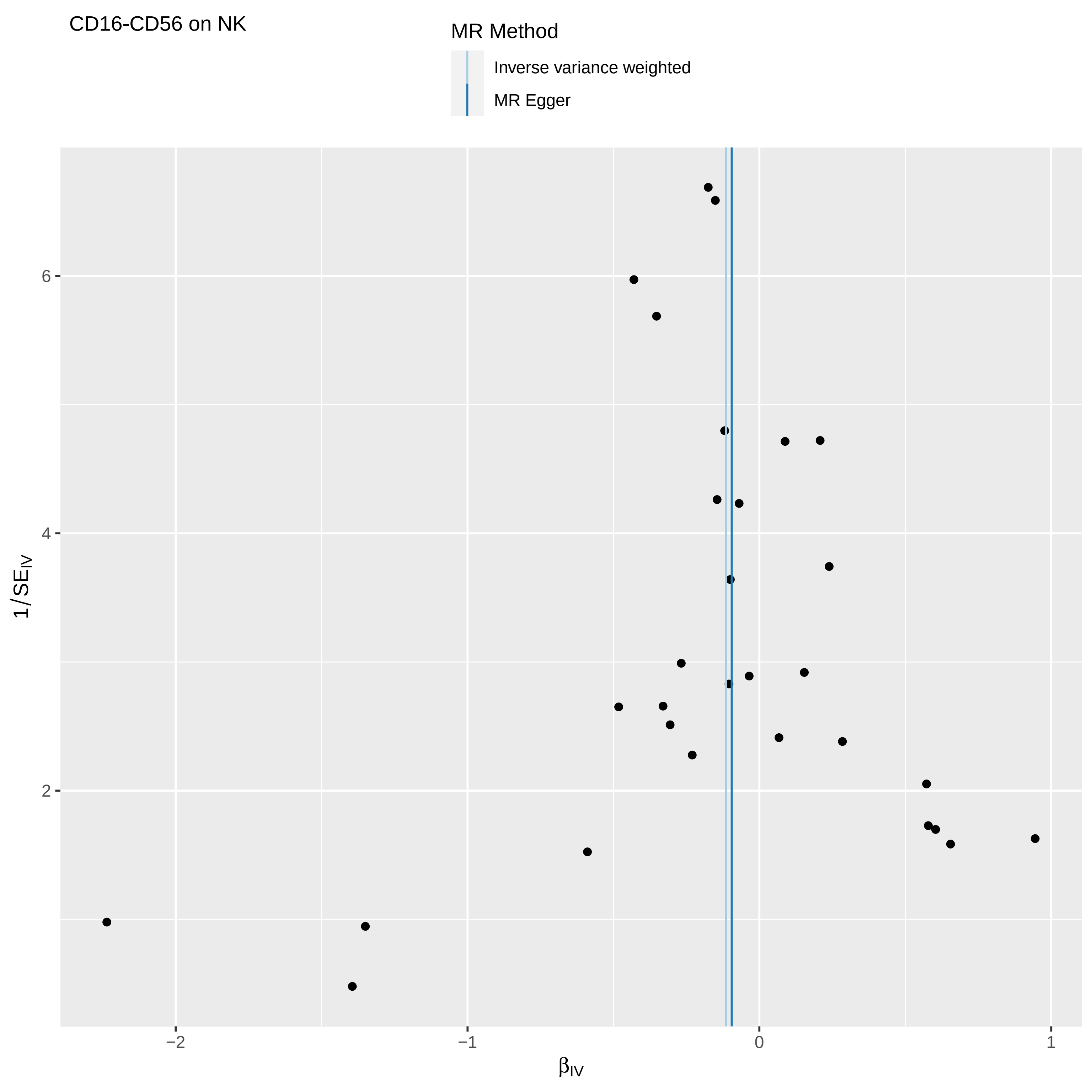
CD16-CD56 on NK
MR Method
Inverse variance weighted
MR Egger
1/SE_IV
β_IV
6
4
2
−2
−1
0
1

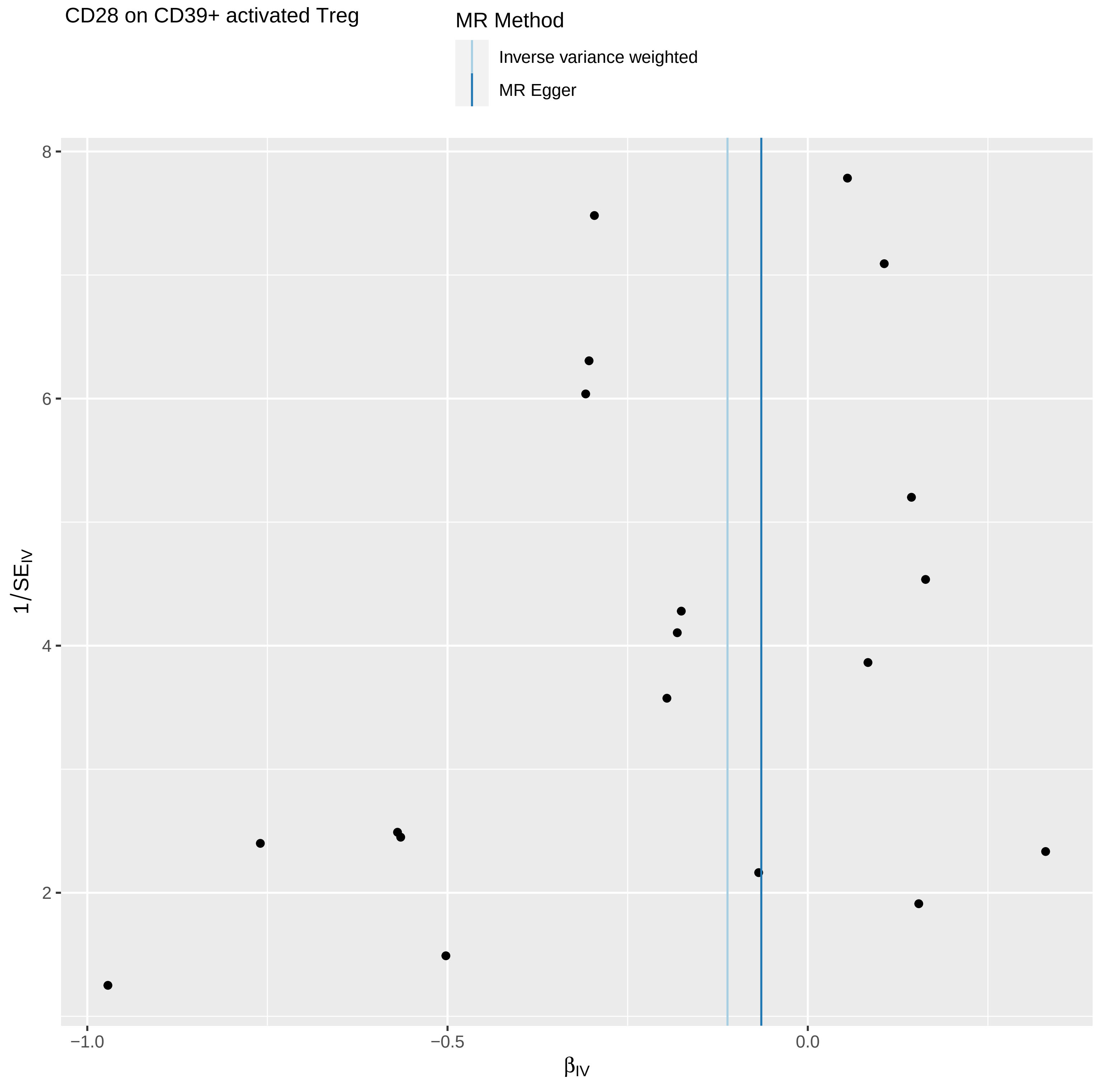

CD28 on CD39+ activated Treg
MR Method
Inverse variance weighted
MR Egger
8
6
4
2
$1/SE_{IV}$
−1.0
−0.5
0.0
β_{IV}

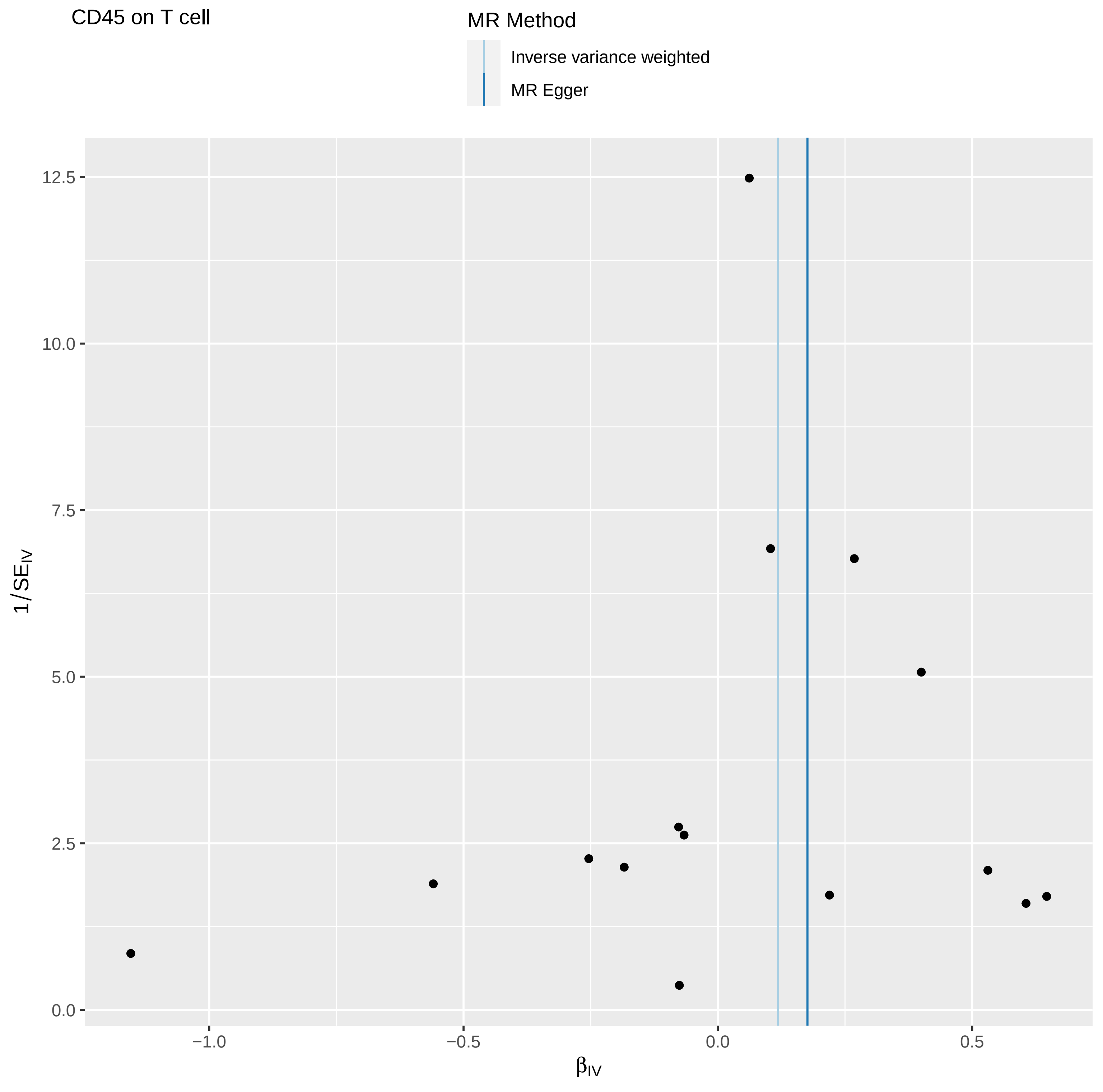

CD45 on T cell
MR Method
Inverse variance weighted
MR Egger
12.5
10.0
7.5
5.0
2.5
0.0
1/SE_IV
−1.0
−0.5
0.0
0.5
β_IV

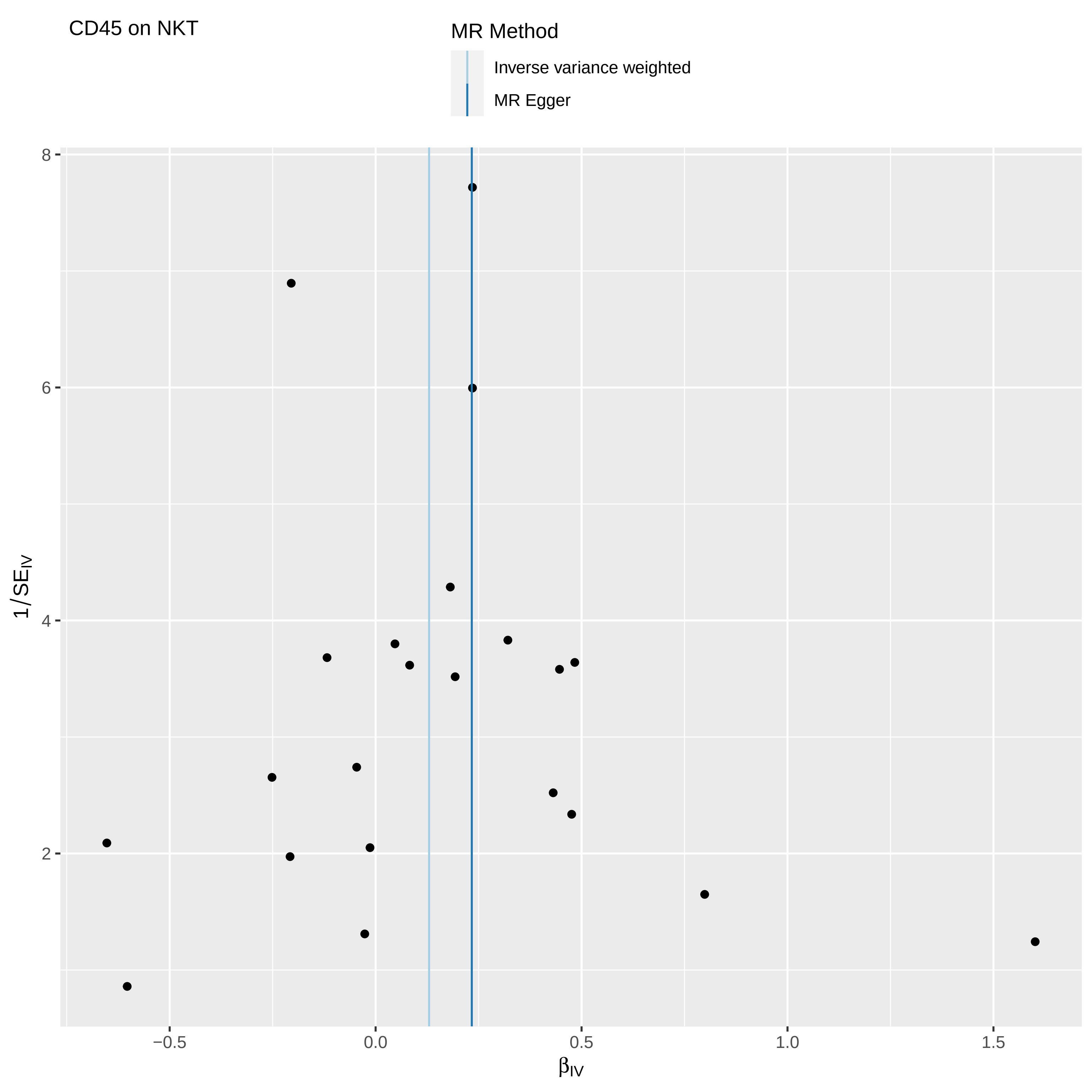

CD45 on NKT
MR Method
Inverse variance weighted
MR Egger
1/SE_IV
β_IV
8
6
4
2
−0.5
0.0
0.5
1.0
1.5

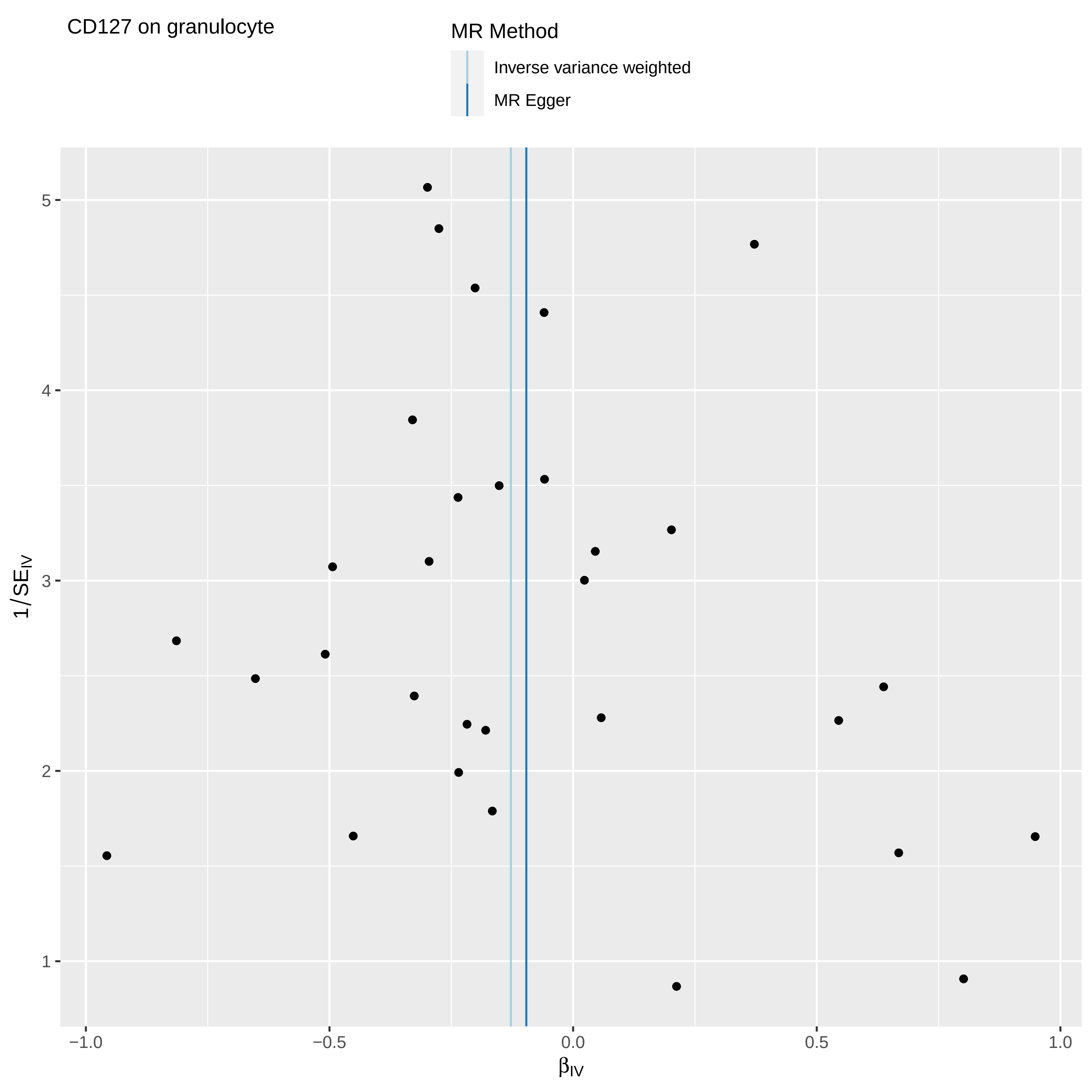
CD127 on granulocyte
MR Method
Inverse variance weighted
MR Egger
1/SE_IV
β_IV
5
4
3
2
1
−1.0
−0.5
0.0
0.5
1.0

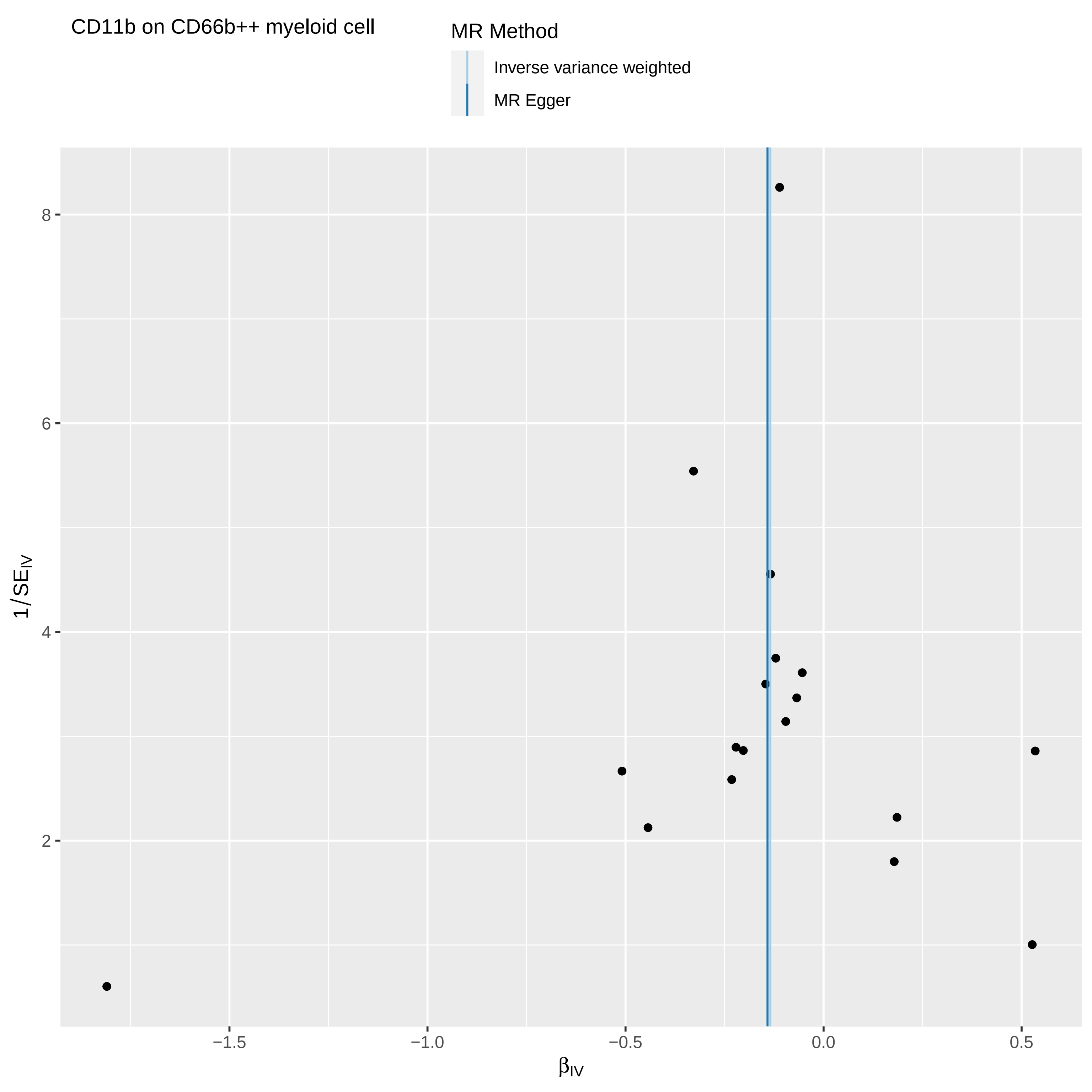
CD11b on CD66b++ myeloid cell
MR Method
Inverse variance weighted
MR Egger
1/SE_IV
β_IV
8
6
4
2
−1.5
−1.0
−0.5
0.0
0.5

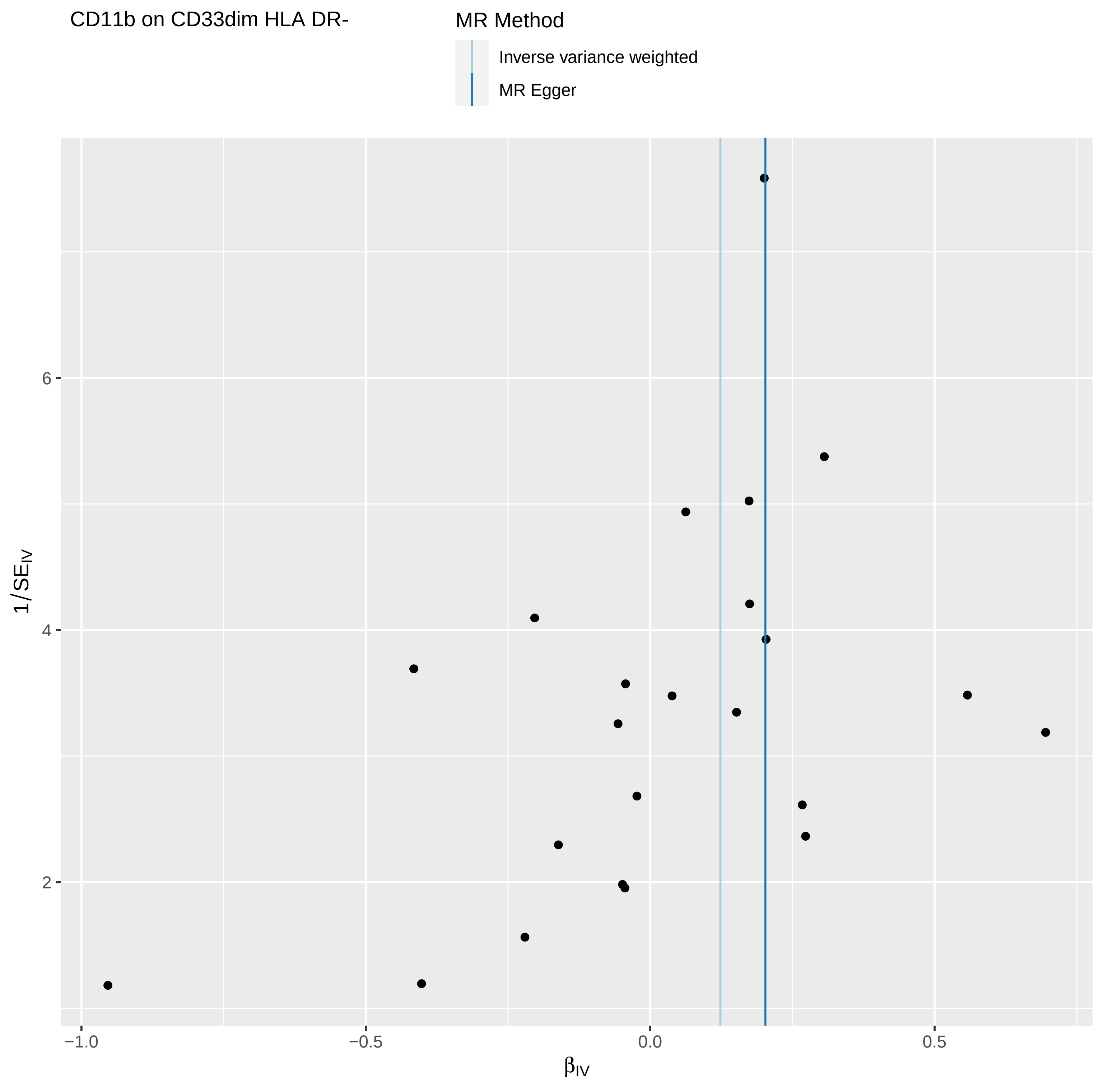
CD11b on CD33dim HLA DR-
MR Method
Inverse variance weighted
MR Egger
1/SE_IV
6
4
2
−1.0
−0.5
0.0
0.5
β_IV

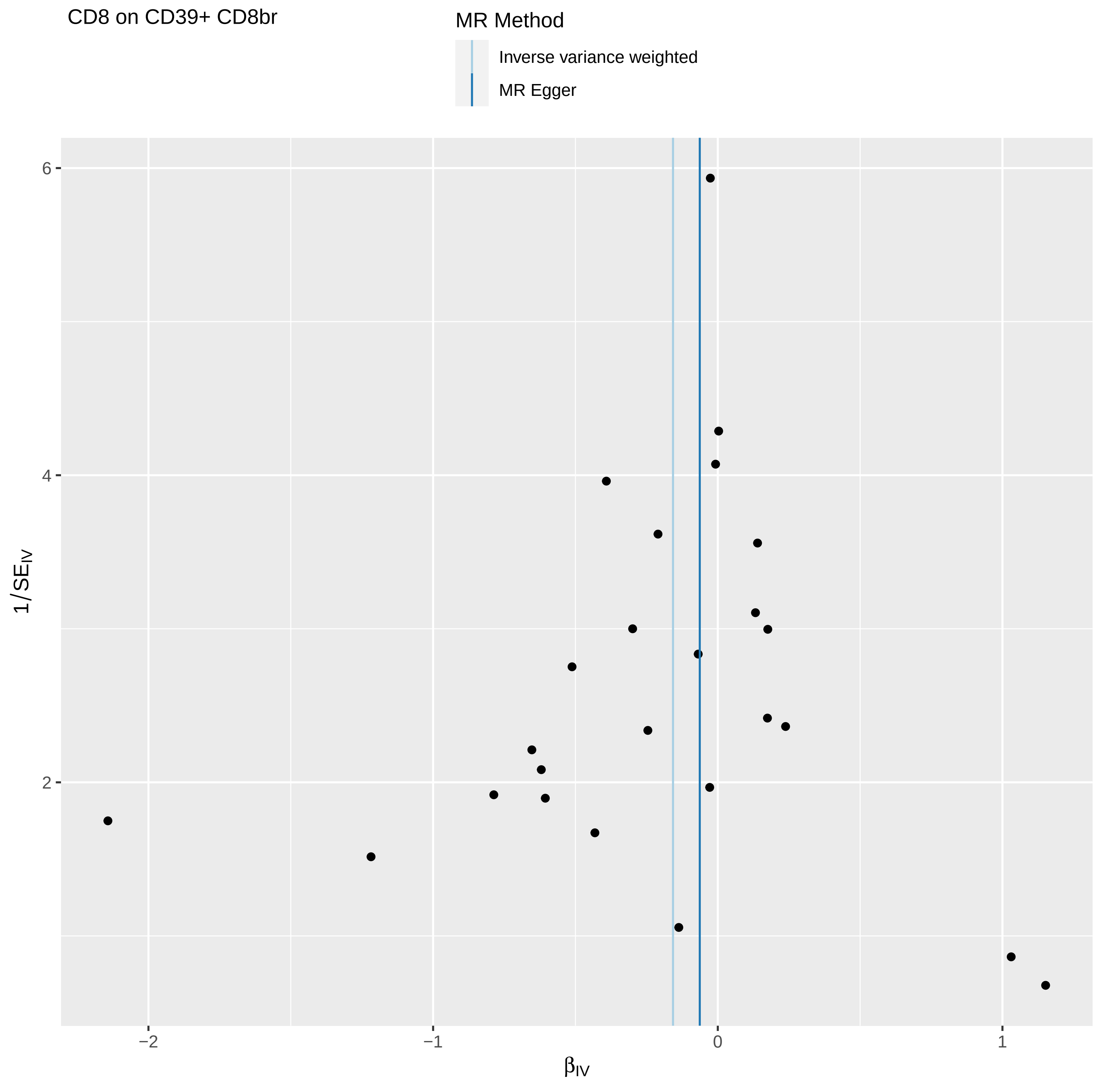

CD8 on CD39+ CD8br
MR Method
Inverse variance weighted
MR Egger
6
4
2
−2
−1
0
1
1/SE_IV
β_IV

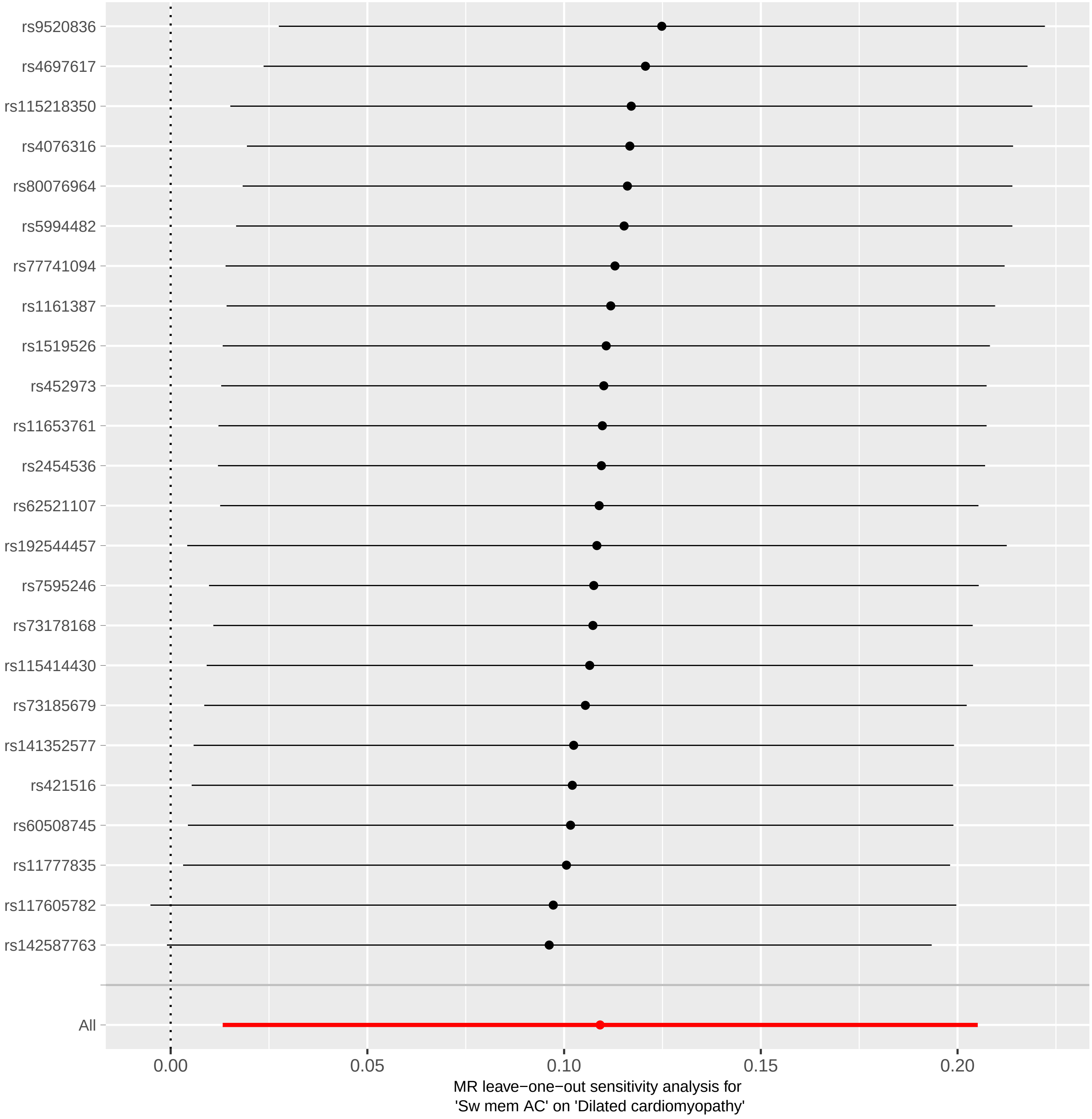

rs9520836
rs4697617
rs115218350
rs4076316
rs80076964
rs5994482
rs77741094
rs1161387
rs1519526
rs452973
rs11653761
rs2454536
rs62521107
rs192544457
rs7595246
rs73178168
rs115414430
rs73185679
rs141352577
rs421516
rs60508745
rs11777835
rs117605782
rs142587763
All
0.00
0.05
0.10
0.15
0.20
MR leave−one−out sensitivity analysis for
'Sw mem AC' on 'Dilated cardiomyopathy'

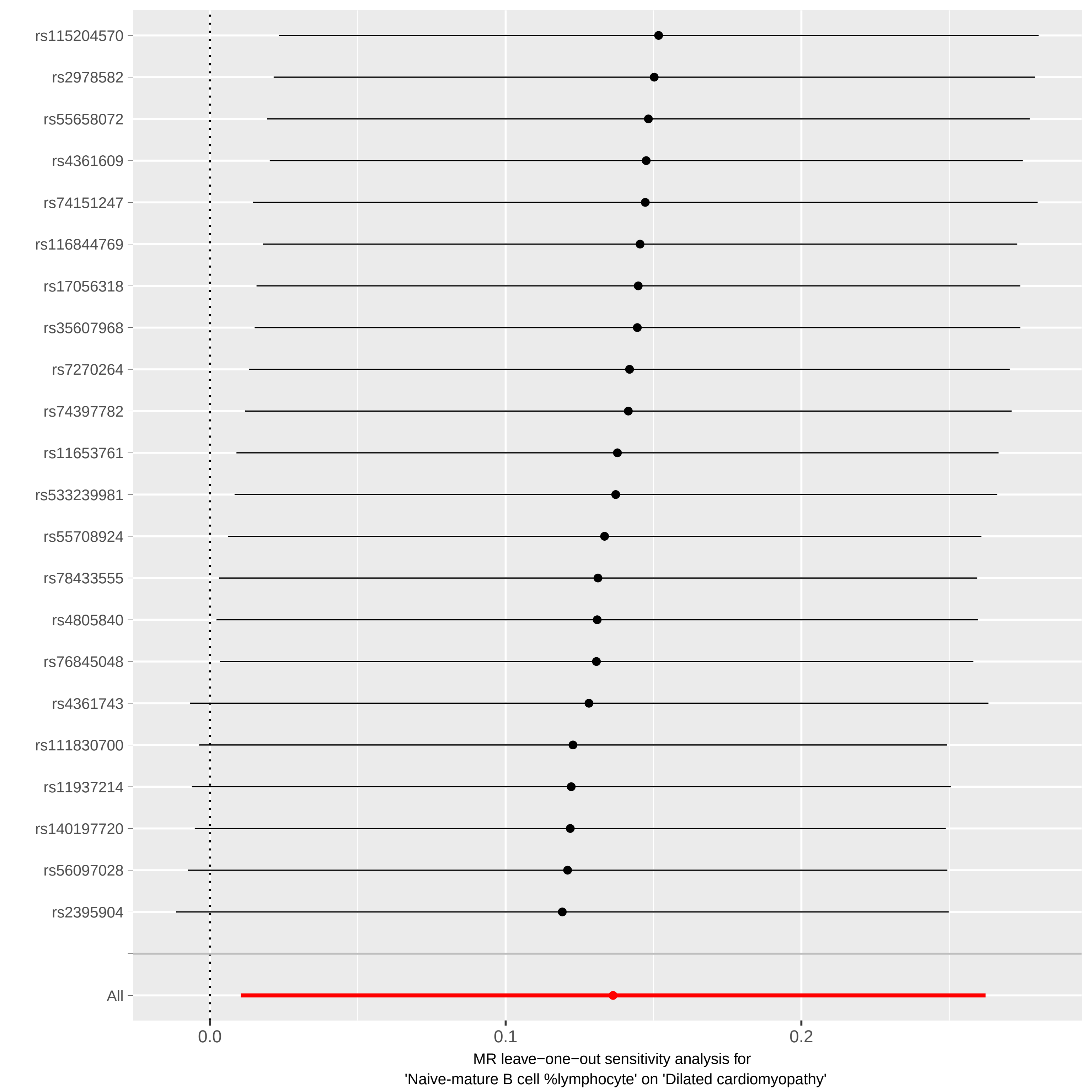
rs115204570
rs2978582
rs55658072
rs4361609
rs74151247
rs116844769
rs17056318
rs35607968
rs7270264
rs74397782
rs11653761
rs533239981
rs55708924
rs78433555
rs4805840
rs76845048
rs4361743
rs111830700
rs11937214
rs140197720
rs56097028
rs2395904
All
0.0
0.1
0.2
MR leave−one−out sensitivity analysis for
'Naive-mature B cell %lymphocyte' on 'Dilated cardiomyopathy'

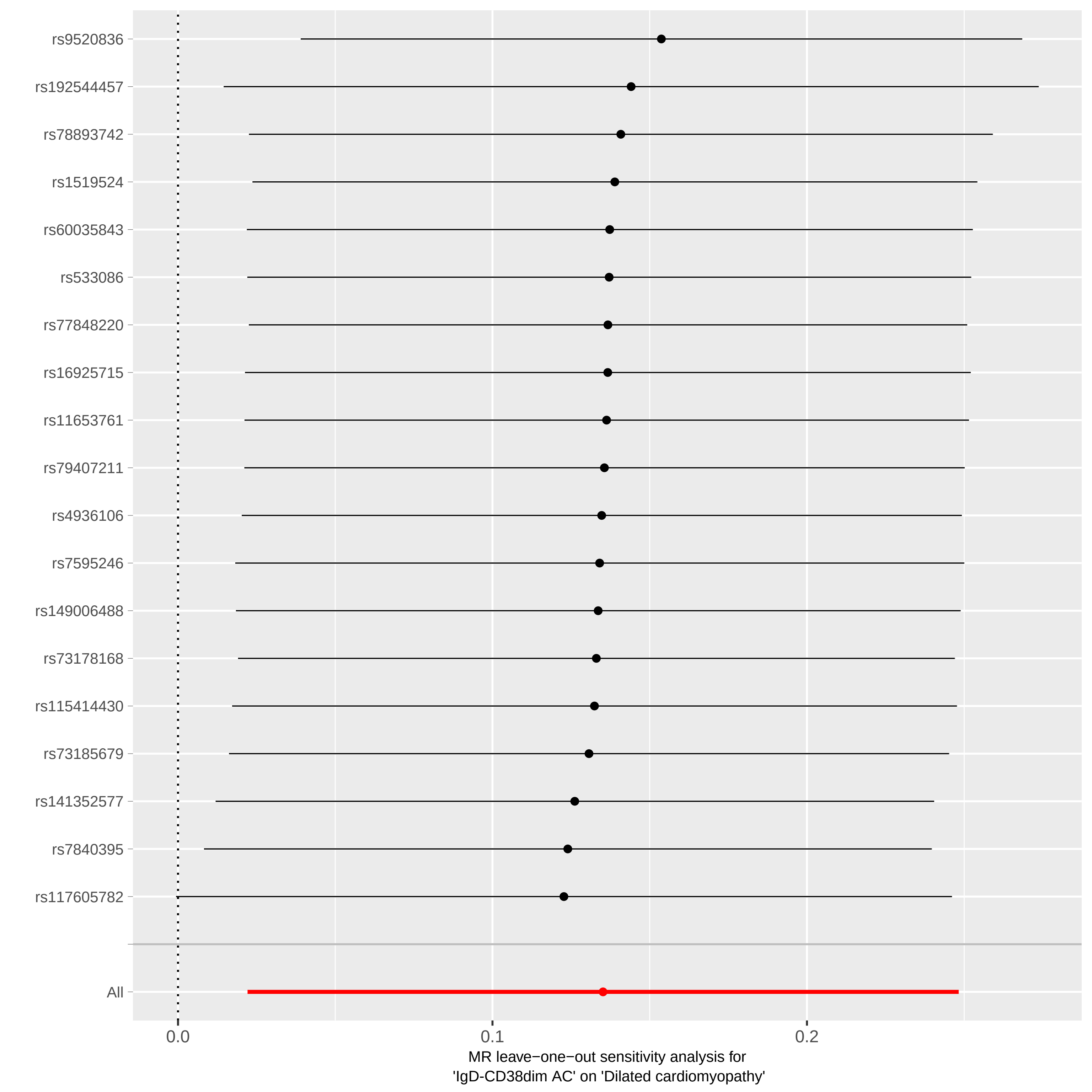
rs9520836
rs192544457
rs78893742
rs1519524
rs60035843
rs533086
rs77848220
rs16925715
rs11653761
rs79407211
rs4936106
rs7595246
rs149006488
rs73178168
rs115414430
rs73185679
rs141352577
rs7840395
rs117605782
All
0.0
0.1
0.2
MR leave−one−out sensitivity analysis for
'IgD-CD38dim AC' on 'Dilated cardiomyopathy'

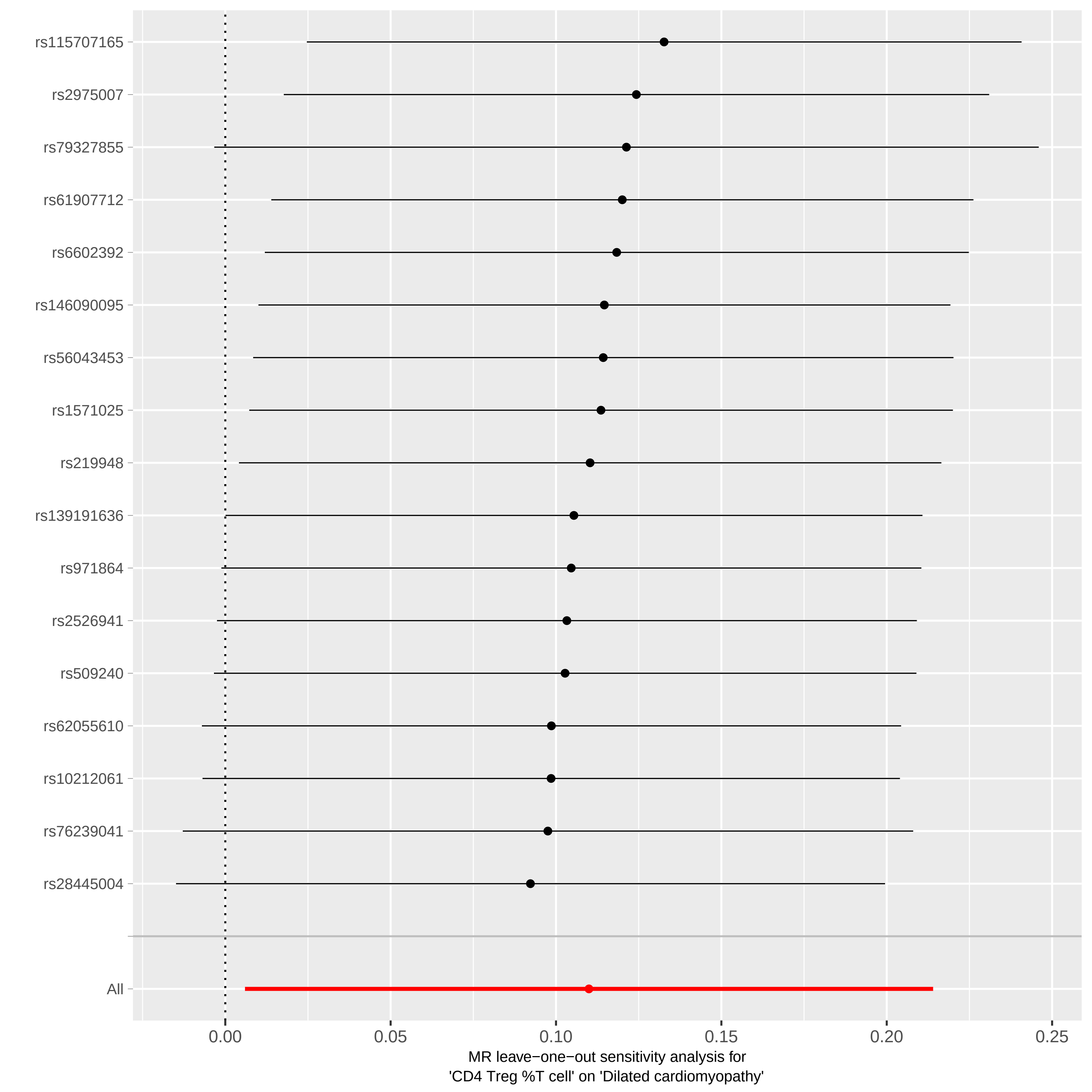
rs115707165
rs2975007
rs79327855
rs61907712
rs6602392
rs146090095
rs56043453
rs1571025
rs219948
rs139191636
rs971864
rs2526941
rs509240
rs62055610
rs10212061
rs76239041
rs28445004
All
0.00
0.05
0.10
0.15
0.20
0.25
MR leave−one−out sensitivity analysis for
'CD4 Treg %T cell' on 'Dilated cardiomyopathy'

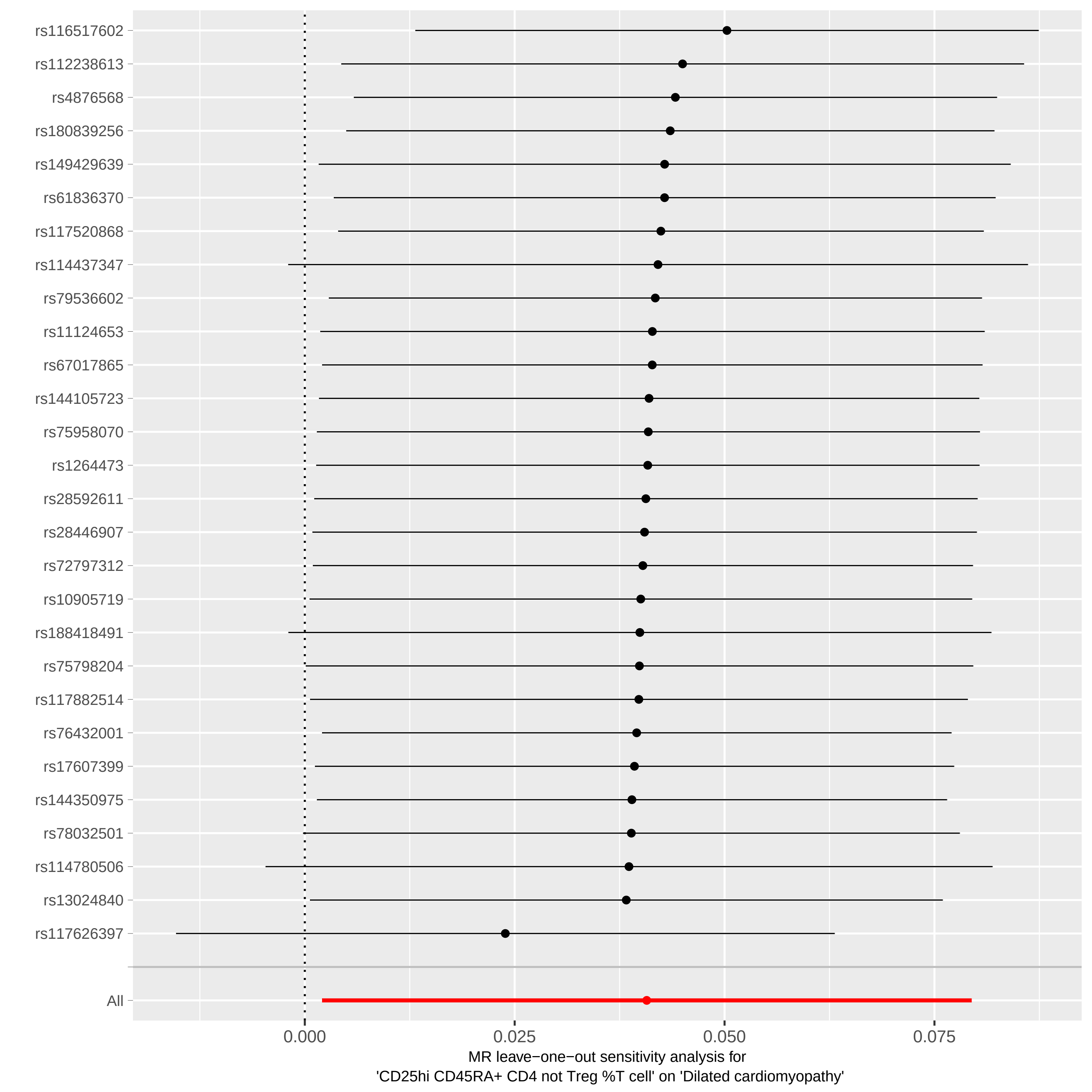

rs116517602
rs112238613
rs4876568
rs180839256
rs149429639
rs61836370
rs117520868
rs114437347
rs79536602
rs11124653
rs67017865
rs144105723
rs75958070
rs1264473
rs28592611
rs28446907
rs72797312
rs10905719
rs188418491
rs75798204
rs117882514
rs76432001
rs17607399
rs144350975
rs78032501
rs114780506
rs13024840
rs117626397
All
0.000
0.025
0.050
0.075
MR leave−one−out sensitivity analysis for
'CD25hi CD45RA+ CD4 not Treg %T cell' on 'Dilated cardiomyopathy'

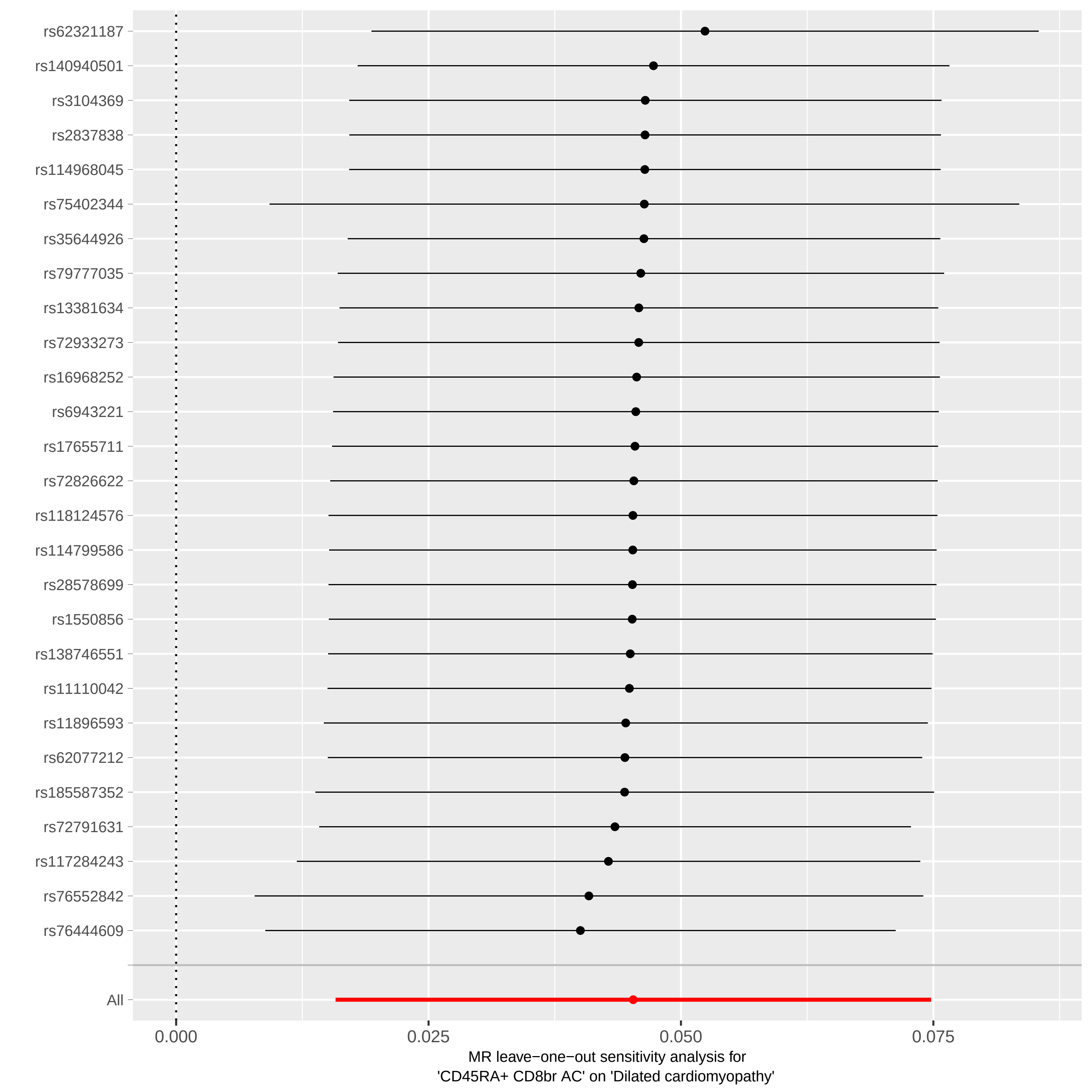
rs62321187
rs140940501
rs3104369
rs2837838
rs114968045
rs75402344
rs35644926
rs79777035
rs13381634
rs72933273
rs16968252
rs6943221
rs17655711
rs72826622
rs118124576
rs114799586
rs28578699
rs1550856
rs138746551
rs11110042
rs11896593
rs62077212
rs185587352
rs72791631
rs117284243
rs76552842
rs76444609
All
0.000
0.025
0.050
0.075
MR leave−one−out sensitivity analysis for
'CD45RA+ CD8br AC' on 'Dilated cardiomyopathy'

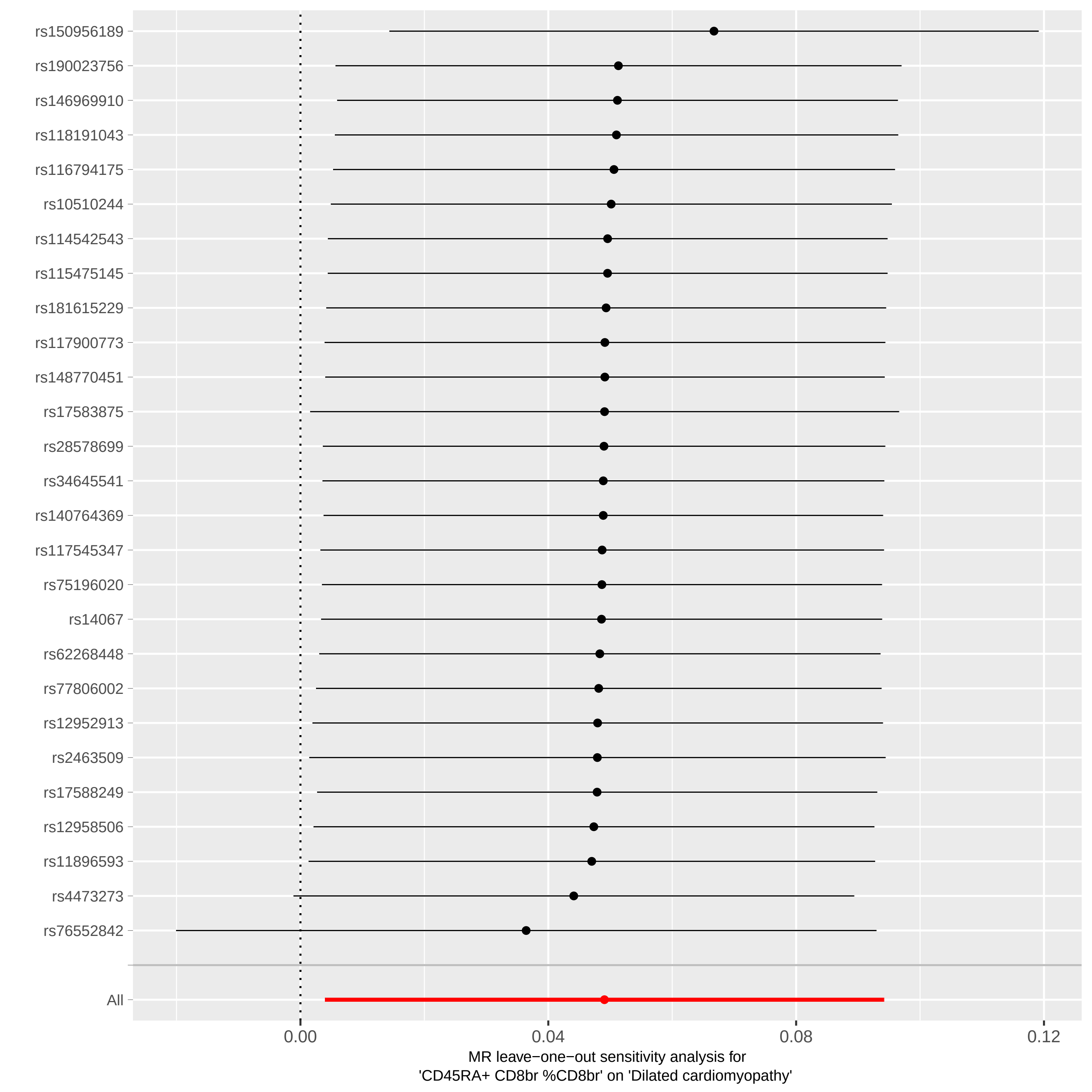

rs150956189
rs190023756
rs146969910
rs118191043
rs116794175
rs10510244
rs114542543
rs115475145
rs181615229
rs117900773
rs148770451
rs17583875
rs28578699
rs34645541
rs140764369
rs117545347
rs75196020
rs14067
rs62268448
rs77806002
rs12952913
rs2463509
rs17588249
rs12958506
rs11896593
rs4473273
rs76552842
All
0.00
0.04
0.08
0.12
MR leave−one−out sensitivity analysis for
'CD45RA+ CD8br %CD8br' on 'Dilated cardiomyopathy'

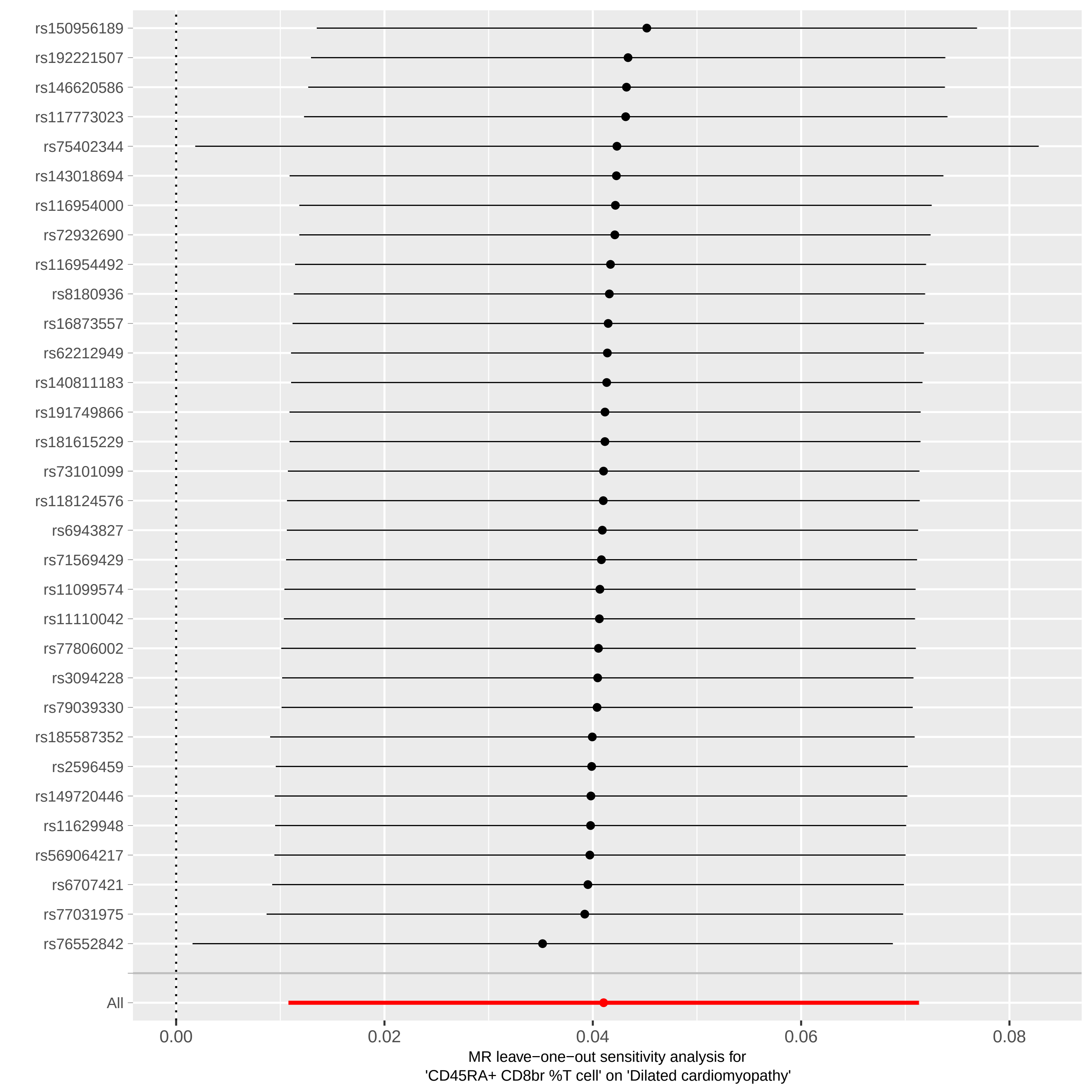

rs150956189
rs192221507
rs146620586
rs117773023
rs75402344
rs143018694
rs116954000
rs72932690
rs116954492
rs8180936
rs16873557
rs62212949
rs140811183
rs191749866
rs181615229
rs73101099
rs118124576
rs6943827
rs71569429
rs11099574
rs11110042
rs77806002
rs3094228
rs79039330
rs185587352
rs2596459
rs149720446
rs11629948
rs569064217
rs6707421
rs77031975
rs76552842
All
0.00
0.02
0.04
0.06
0.08
MR leave−one−out sensitivity analysis for
'CD45RA+ CD8br %T cell' on 'Dilated cardiomyopathy'

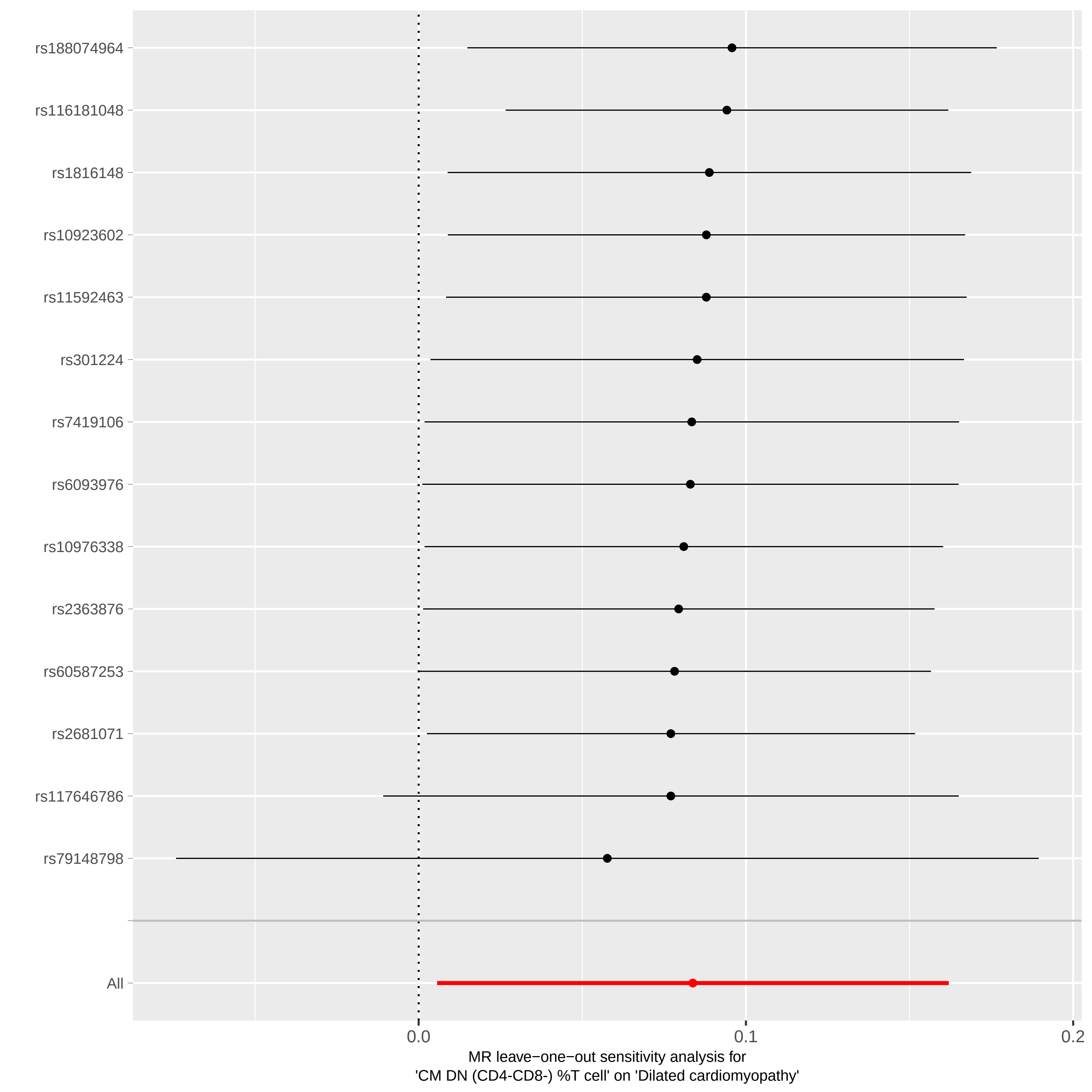
rs188074964
rs116181048
rs1816148
rs10923602
rs11592463
rs301224
rs7419106
rs6093976
rs10976338
rs2363876
rs60587253
rs2681071
rs117646786
rs79148798
All
0.0
0.1
0.2
MR leave−one−out sensitivity analysis for
'CM DN (CD4-CD8-) %T cell' on 'Dilated cardiomyopathy'

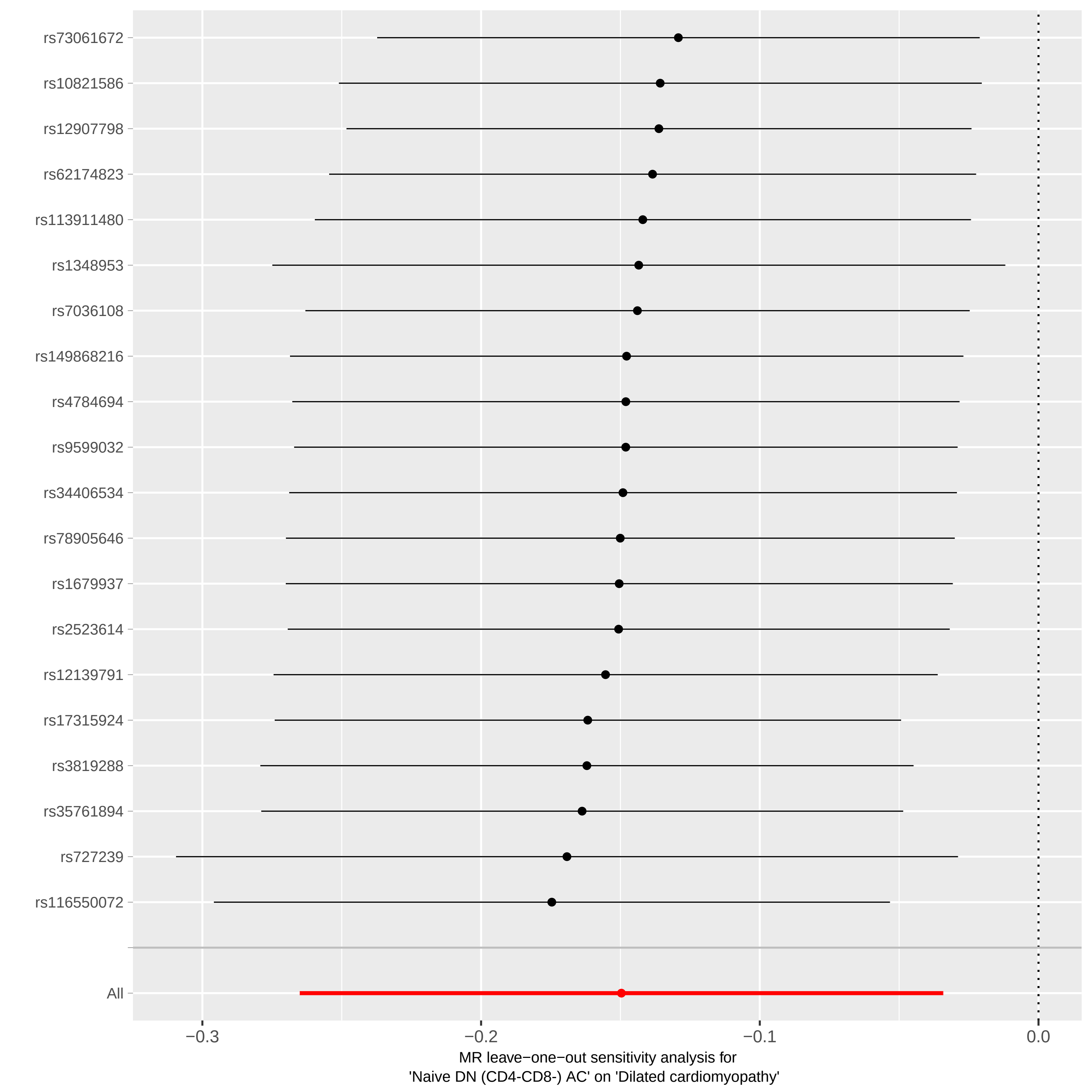
rs73061672
rs10821586
rs12907798
rs62174823
rs113911480
rs1348953
rs7036108
rs149868216
rs4784694
rs9599032
rs34406534
rs78905646
rs1679937
rs2523614
rs12139791
rs17315924
rs3819288
rs35761894
rs727239
rs116550072
All
−0.3
−0.2
−0.1
0.0
MR leave−one−out sensitivity analysis for
'Naive DN (CD4-CD8-) AC' on 'Dilated cardiomyopathy'

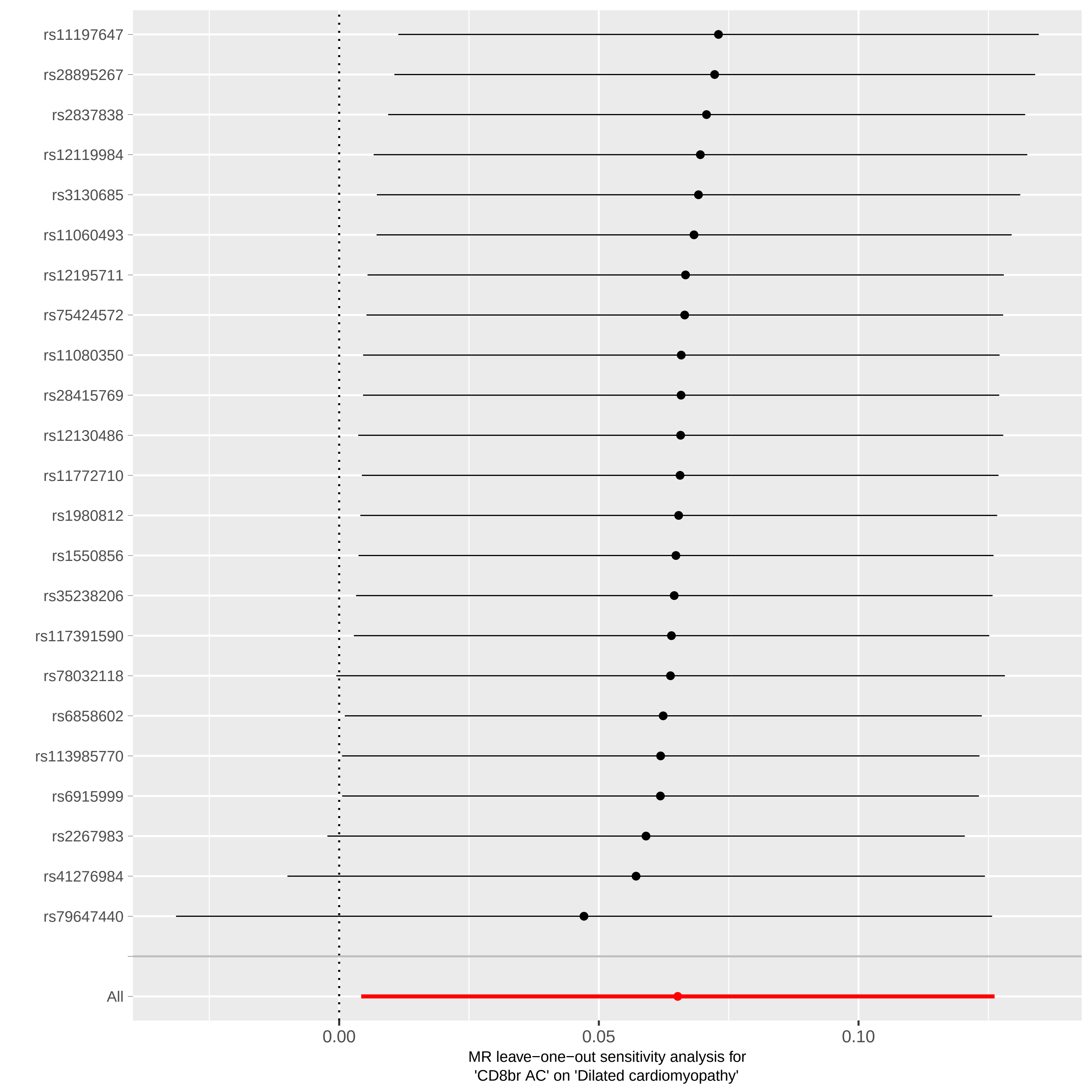
rs11197647
rs28895267
rs2837838
rs12119984
rs3130685
rs11060493
rs12195711
rs75424572
rs11080350
rs28415769
rs12130486
rs11772710
rs1980812
rs1550856
rs35238206
rs117391590
rs78032118
rs6858602
rs113985770
rs6915999
rs2267983
rs41276984
rs79647440
All
0.00
0.05
0.10
MR leave−one−out sensitivity analysis for
'CD8br AC' on 'Dilated cardiomyopathy'

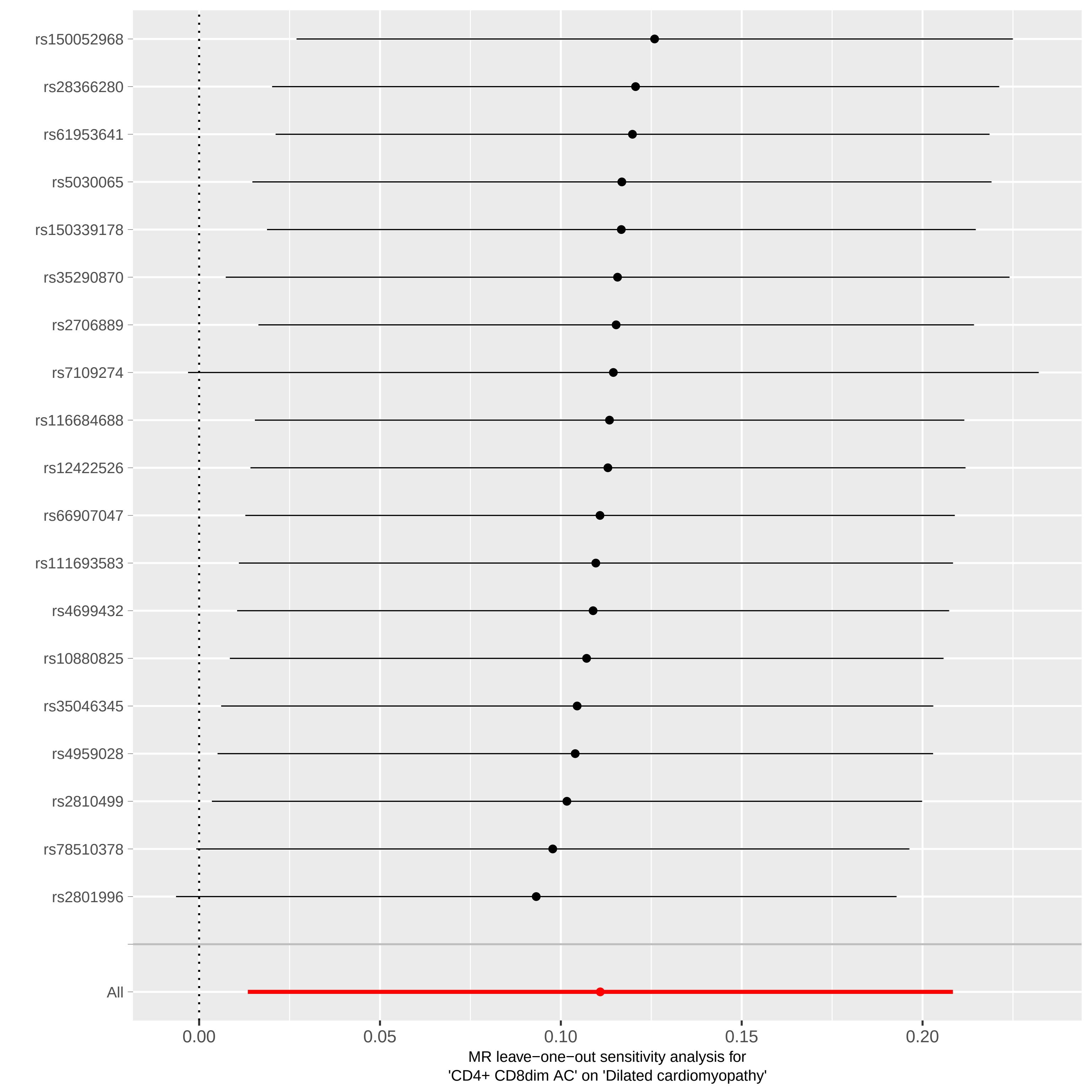
rs150052968
rs28366280
rs61953641
rs5030065
rs150339178
rs35290870
rs2706889
rs7109274
rs116684688
rs12422526
rs66907047
rs111693583
rs4699432
rs10880825
rs35046345
rs4959028
rs2810499
rs78510378
rs2801996
All
0.00
0.05
0.10
0.15
0.20
MR leave−one−out sensitivity analysis for
'CD4+ CD8dim AC' on 'Dilated cardiomyopathy'

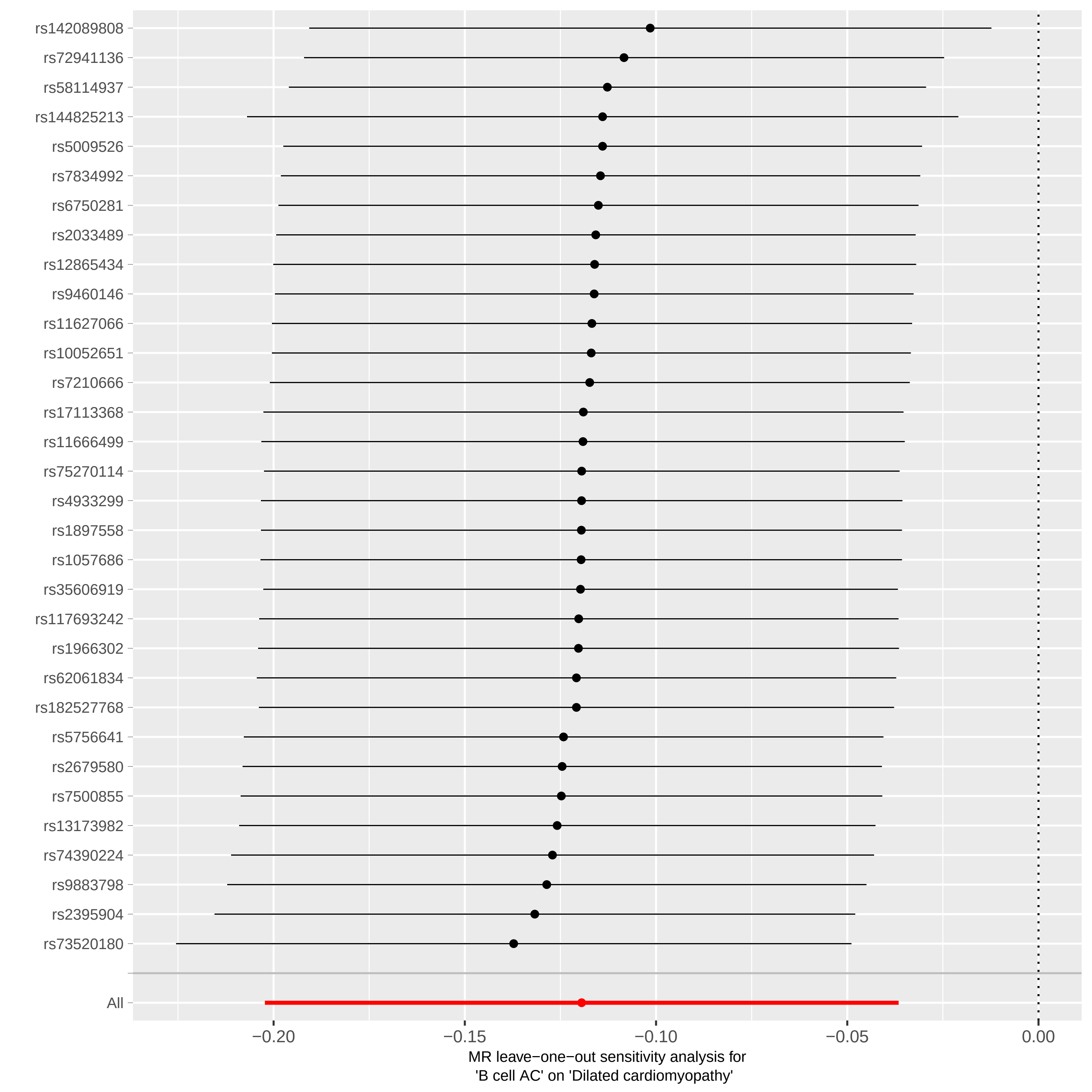

rs142089808
rs72941136
rs58114937
rs144825213
rs5009526
rs7834992
rs6750281
rs2033489
rs12865434
rs9460146
rs11627066
rs10052651
rs7210666
rs17113368
rs11666499
rs75270114
rs4933299
rs1897558
rs1057686
rs35606919
rs117693242
rs1966302
rs62061834
rs182527768
rs5756641
rs2679580
rs7500855
rs13173982
rs74390224
rs9883798
rs2395904
rs73520180
All
−0.20
−0.15
−0.10
−0.05
0.00
MR leave−one−out sensitivity analysis for
'B cell AC' on 'Dilated cardiomyopathy'

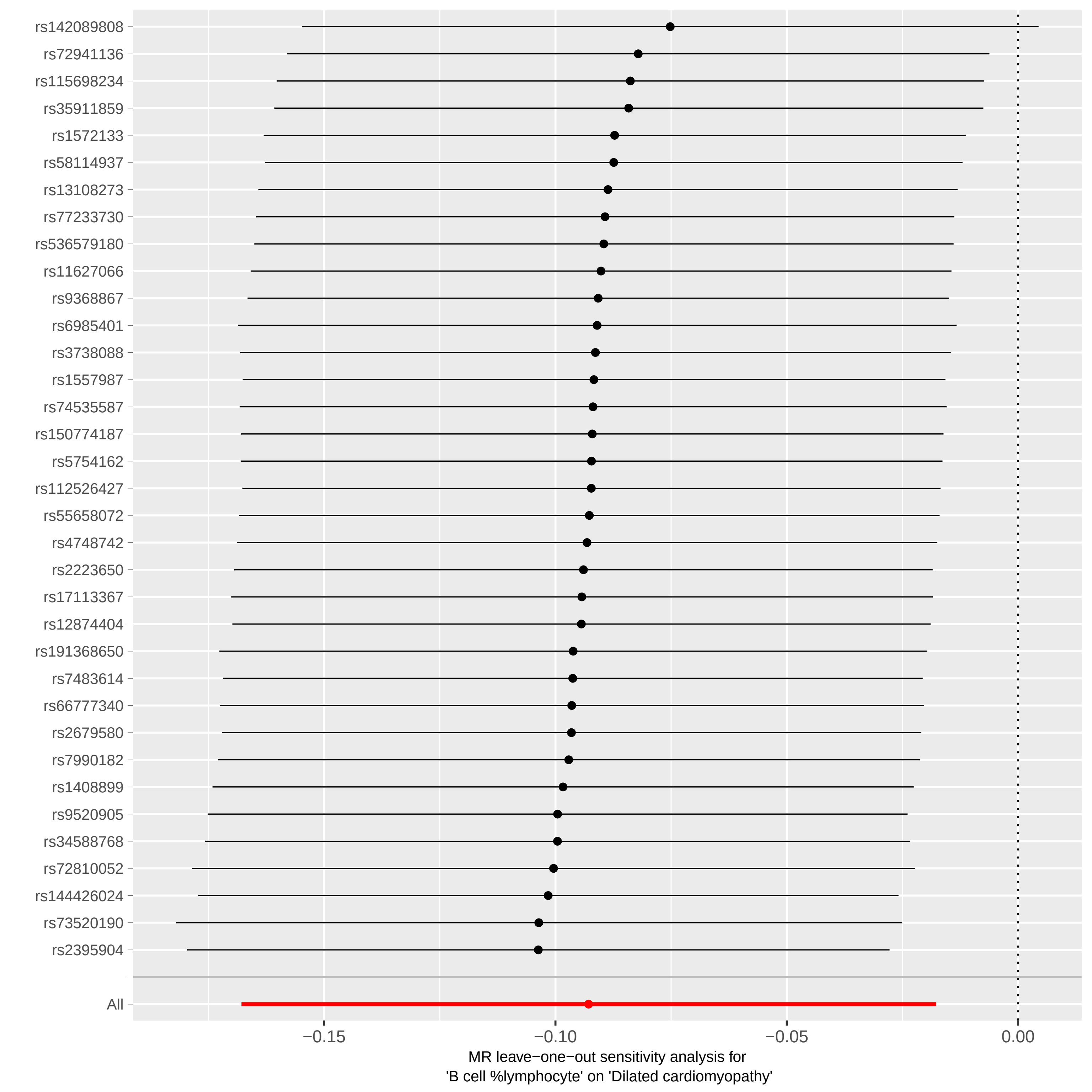
rs142089808
rs72941136
rs115698234
rs35911859
rs1572133
rs58114937
rs13108273
rs77233730
rs536579180
rs11627066
rs9368867
rs6985401
rs3738088
rs1557987
rs74535587
rs150774187
rs5754162
rs112526427
rs55658072
rs4748742
rs2223650
rs17113367
rs12874404
rs191368650
rs7483614
rs66777340
rs2679580
rs7990182
rs1408899
rs9520905
rs34588768
rs72810052
rs144426024
rs73520190
rs2395904
All
−0.15
−0.10
−0.05
0.00
MR leave−one−out sensitivity analysis for
'B cell %lymphocyte' on 'Dilated cardiomyopathy'

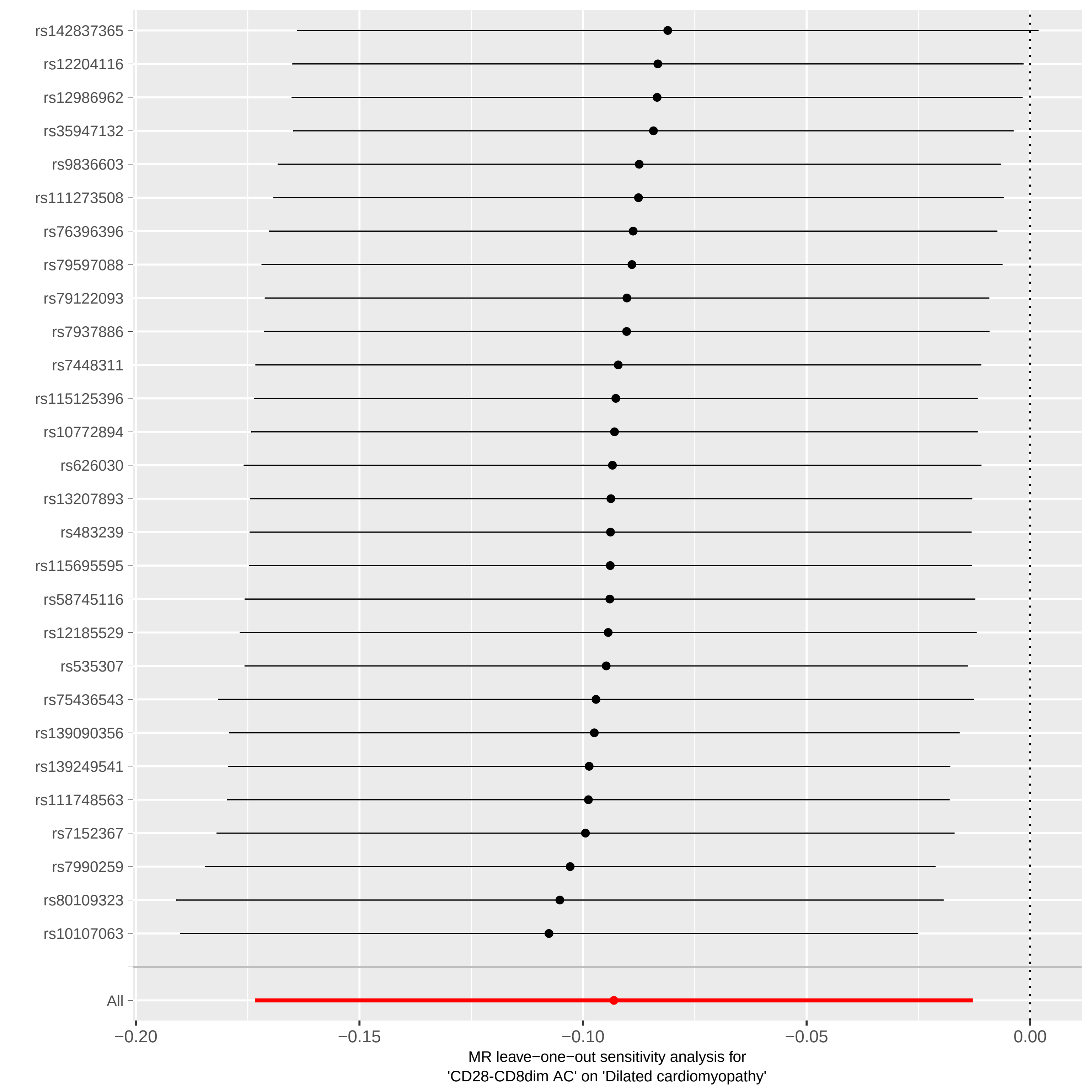
rs142837365
rs12204116
rs12986962
rs35947132
rs9836603
rs111273508
rs76396396
rs79597088
rs79122093
rs7937886
rs7448311
rs115125396
rs10772894
rs626030
rs13207893
rs483239
rs115695595
rs58745116
rs12185529
rs535307
rs75436543
rs139090356
rs139249541
rs111748563
rs7152367
rs7990259
rs80109323
rs10107063
All
−0.20
−0.15
−0.10
−0.05
0.00
MR leave−one−out sensitivity analysis for
'CD28-CD8dim AC' on 'Dilated cardiomyopathy'

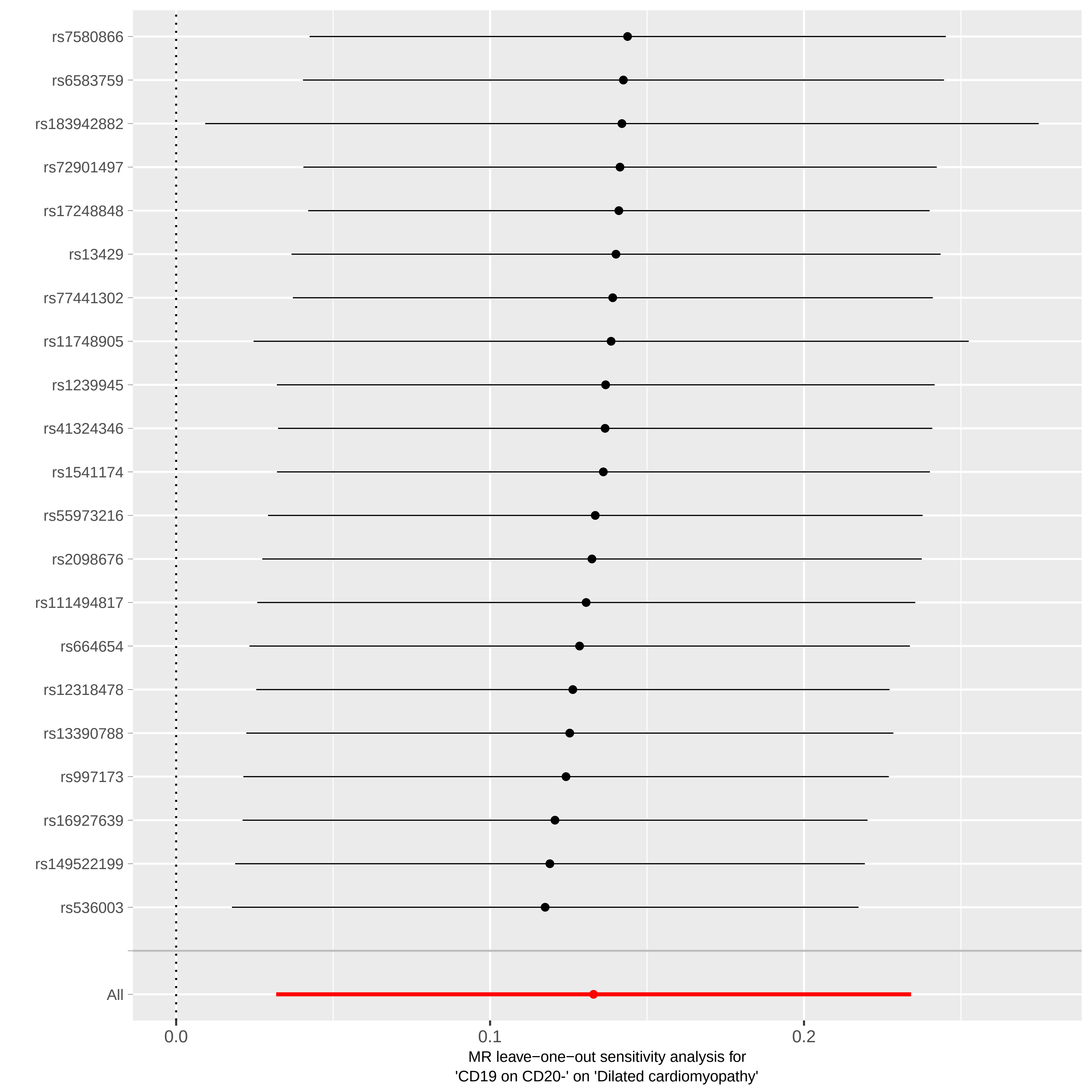
rs7580866
rs6583759
rs183942882
rs72901497
rs17248848
rs13429
rs77441302
rs11748905
rs1239945
rs41324346
rs1541174
rs55973216
rs2098676
rs111494817
rs664654
rs12318478
rs13390788
rs997173
rs16927639
rs149522199
rs536003
All
0.0
0.1
0.2
MR leave−one−out sensitivity analysis for
'CD19 on CD20-' on 'Dilated cardiomyopathy'

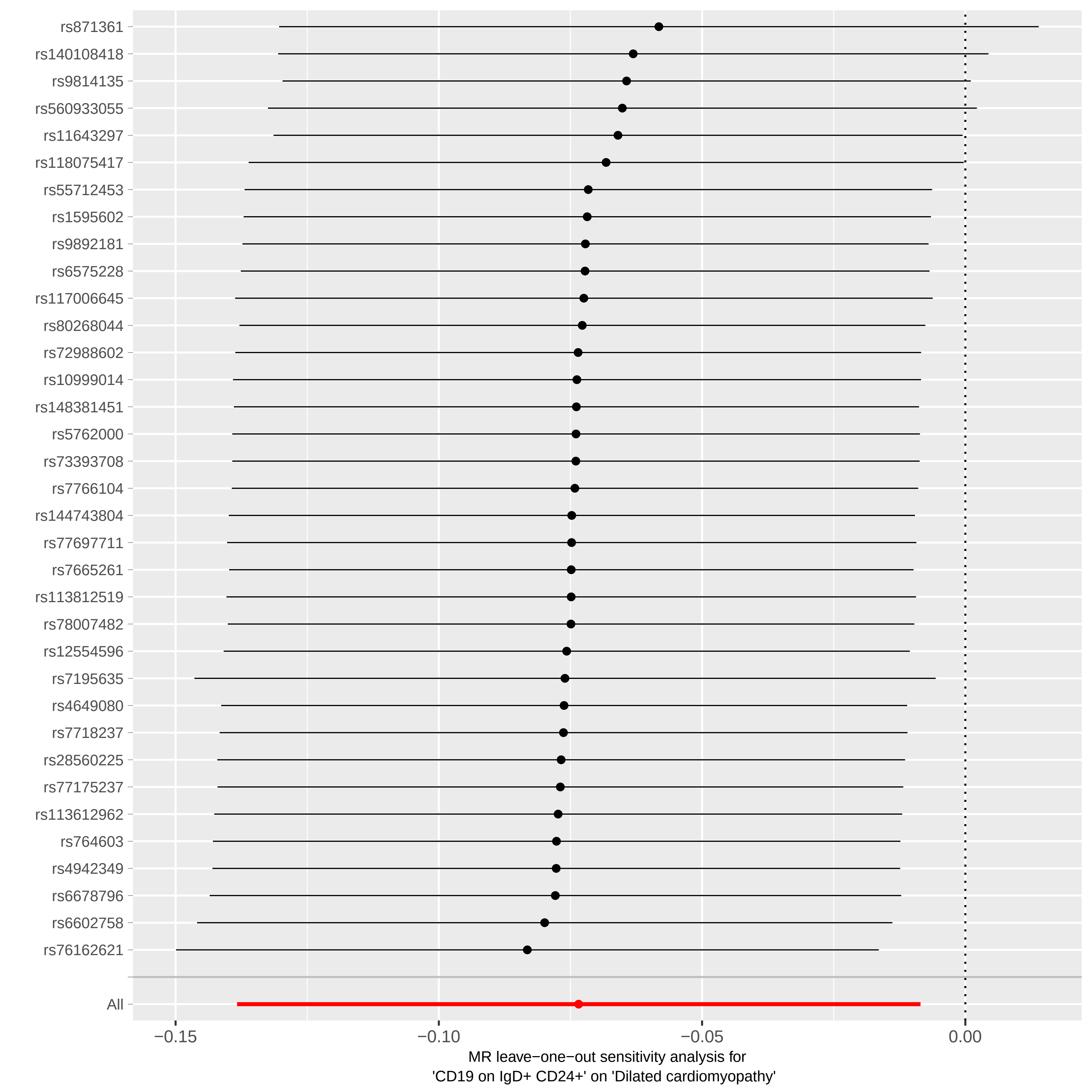

rs871361
rs140108418
rs9814135
rs560933055
rs11643297
rs118075417
rs55712453
rs1595602
rs9892181
rs6575228
rs117006645
rs80268044
rs72988602
rs10999014
rs148381451
rs5762000
rs73393708
rs7766104
rs144743804
rs77697711
rs7665261
rs113812519
rs78007482
rs12554596
rs7195635
rs4649080
rs7718237
rs28560225
rs77175237
rs113612962
rs764603
rs4942349
rs6678796
rs6602758
rs76162621
All
−0.15
−0.10
−0.05
0.00
MR leave−one−out sensitivity analysis for
'CD19 on IgD+ CD24+' on 'Dilated cardiomyopathy'

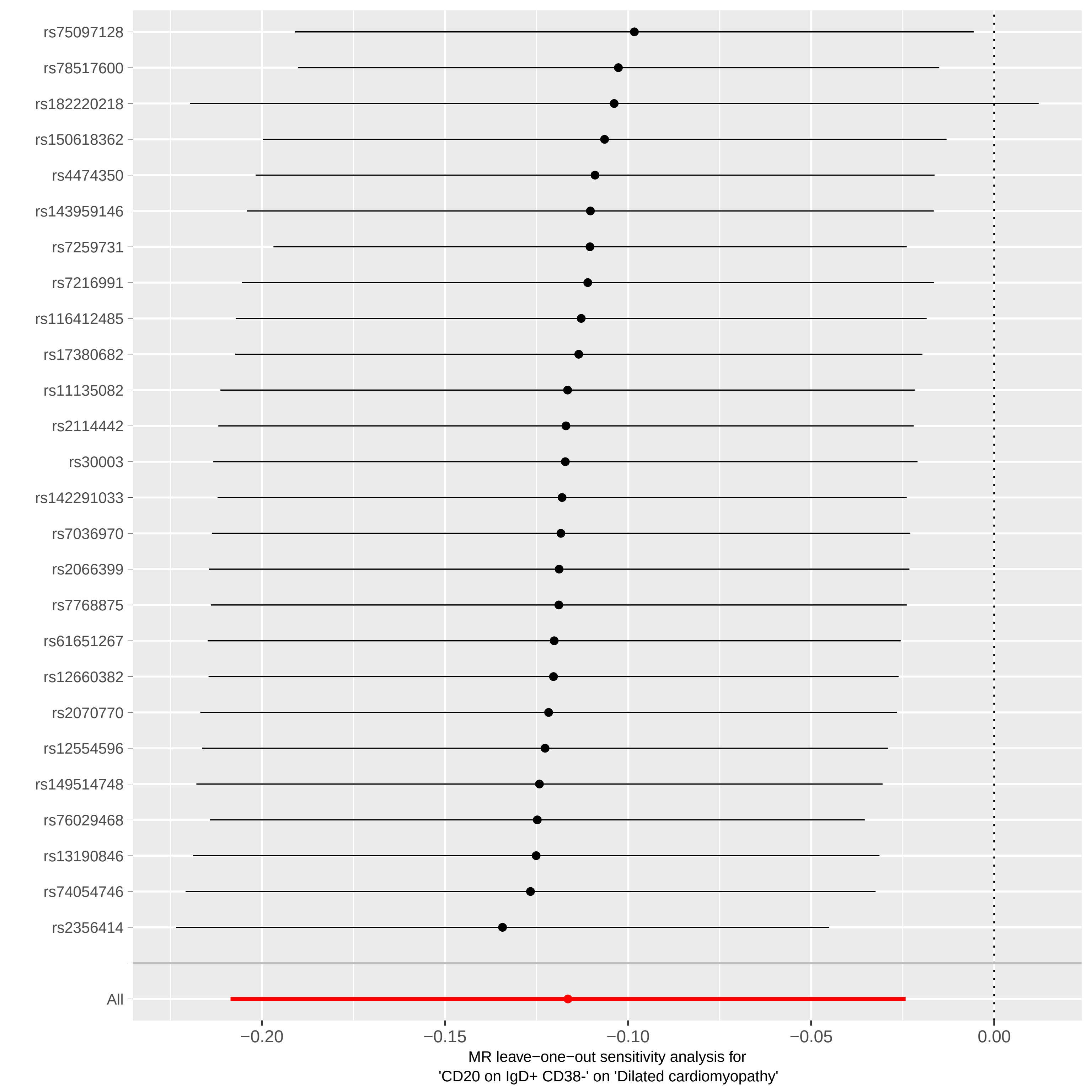
rs75097128
rs78517600
rs182220218
rs150618362
rs4474350
rs143959146
rs7259731
rs7216991
rs116412485
rs17380682
rs11135082
rs2114442
rs30003
rs142291033
rs7036970
rs2066399
rs7768875
rs61651267
rs12660382
rs2070770
rs12554596
rs149514748
rs76029468
rs13190846
rs74054746
rs2356414
All
−0.20
−0.15
−0.10
−0.05
0.00
MR leave−one−out sensitivity analysis for
'CD20 on IgD+ CD38-' on 'Dilated cardiomyopathy'

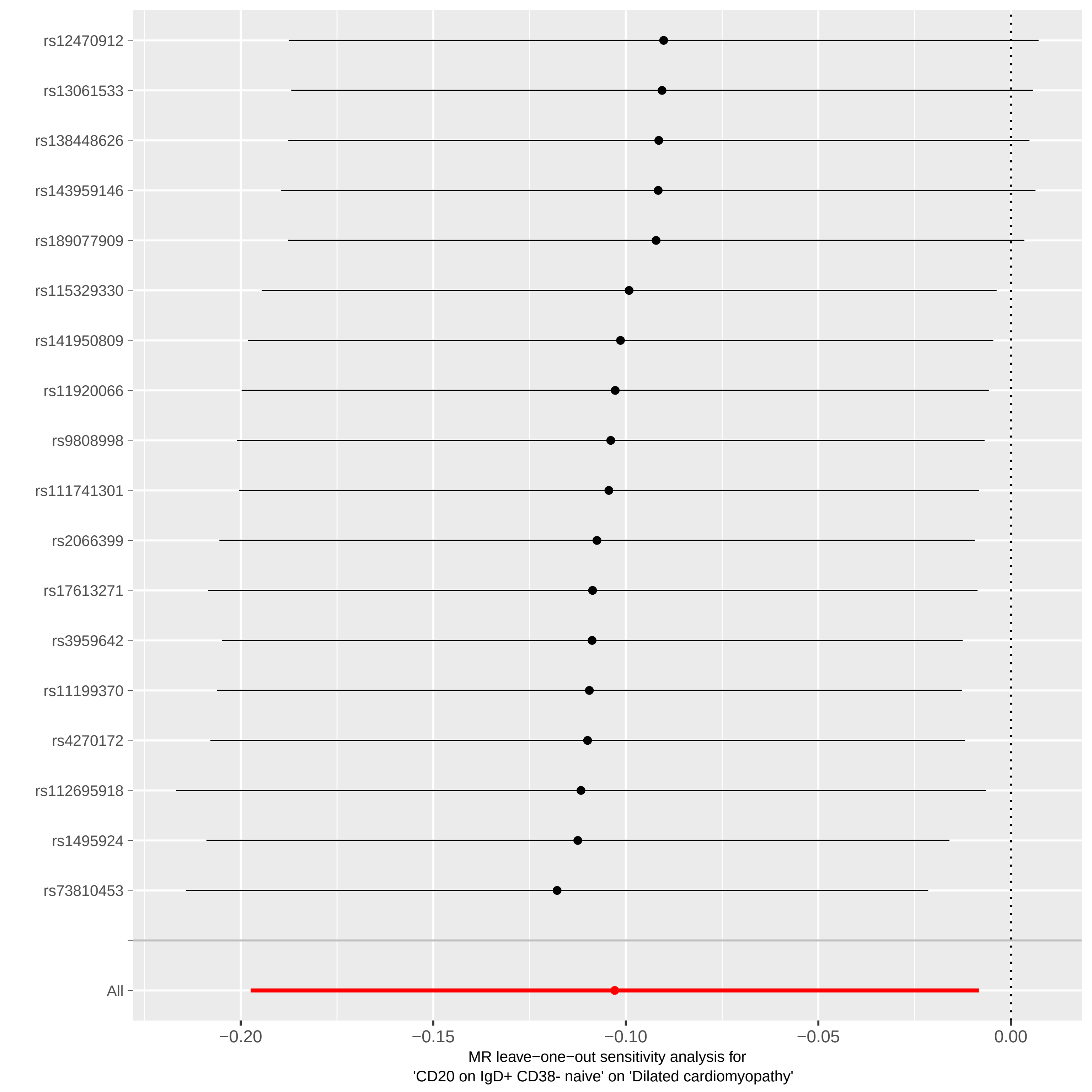

rs12470912
rs13061533
rs138448626
rs143959146
rs189077909
rs115329330
rs141950809
rs11920066
rs9808998
rs111741301
rs2066399
rs17613271
rs3959642
rs11199370
rs4270172
rs112695918
rs1495924
rs73810453
All
−0.20
−0.15
−0.10
−0.05
0.00
MR leave−one−out sensitivity analysis for
'CD20 on IgD+ CD38- naive' on 'Dilated cardiomyopathy'

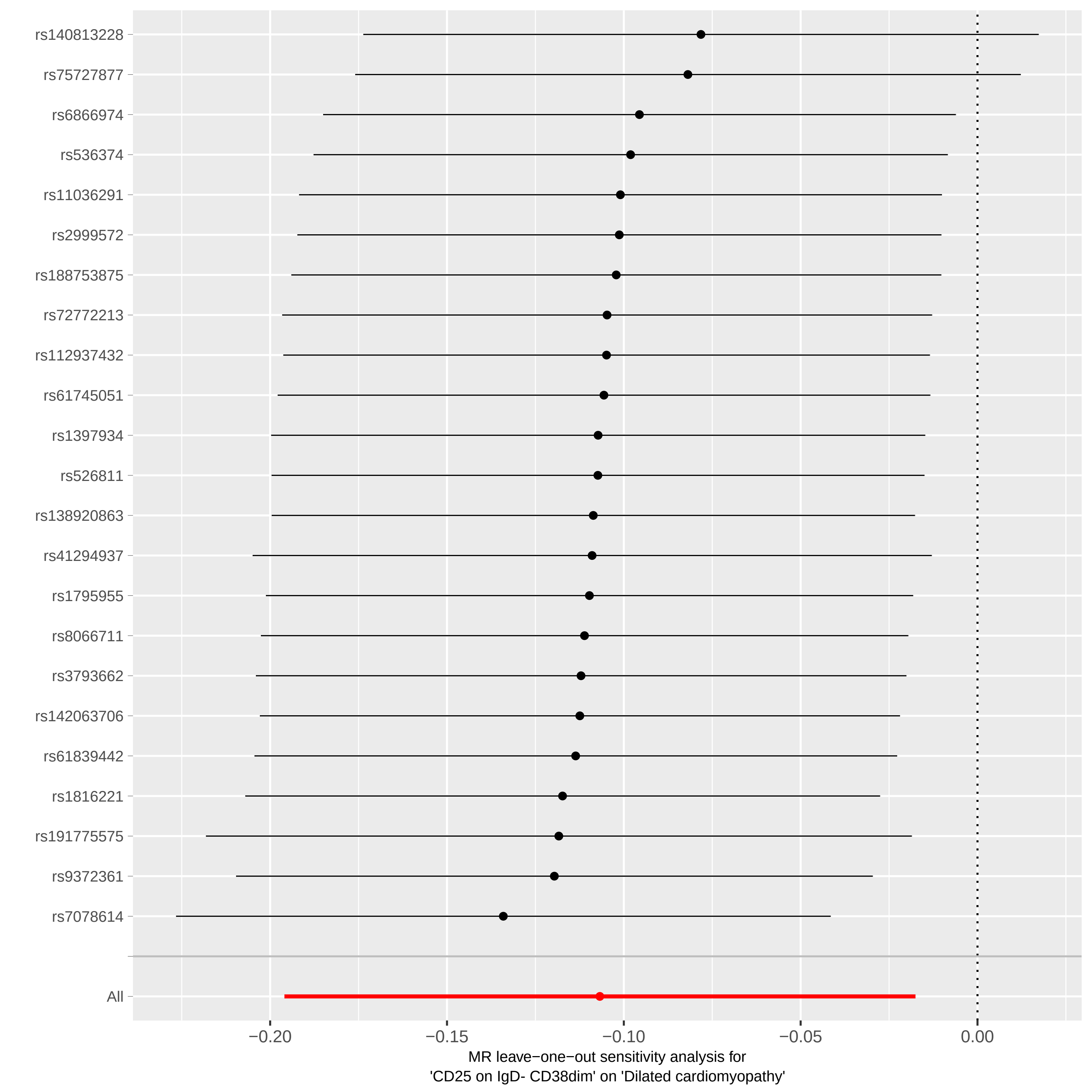
rs140813228
rs75727877
rs6866974
rs536374
rs11036291
rs2999572
rs188753875
rs72772213
rs112937432
rs61745051
rs1397934
rs526811
rs138920863
rs41294937
rs1795955
rs8066711
rs3793662
rs142063706
rs61839442
rs1816221
rs191775575
rs9372361
rs7078614
All
−0.20
−0.15
−0.10
−0.05
0.00
MR leave−one−out sensitivity analysis for
'CD25 on IgD- CD38dim' on 'Dilated cardiomyopathy'

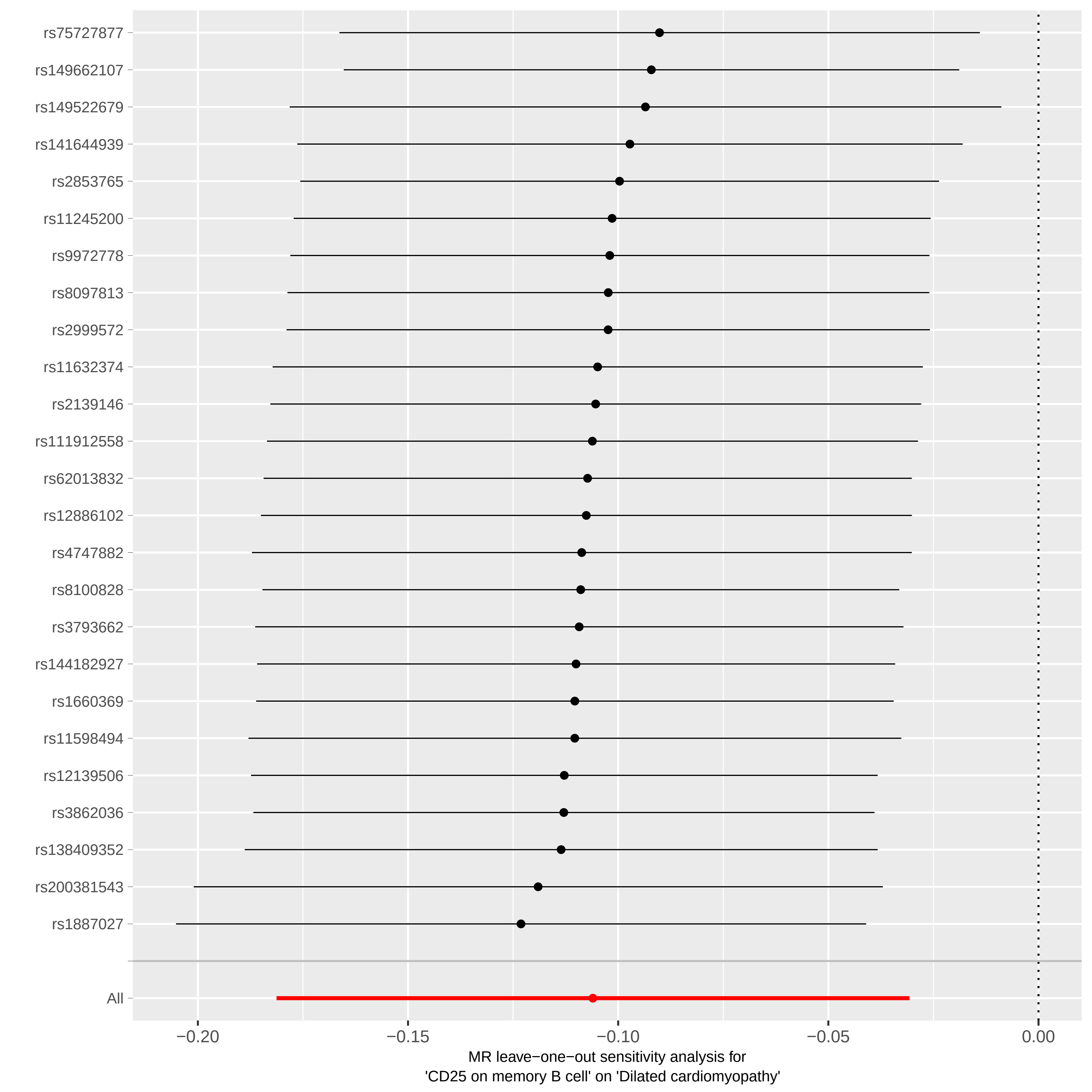
rs75727877
rs149662107
rs149522679
rs141644939
rs2853765
rs11245200
rs9972778
rs8097813
rs2999572
rs11632374
rs2139146
rs111912558
rs62013832
rs12886102
rs4747882
rs8100828
rs3793662
rs144182927
rs1660369
rs11598494
rs12139506
rs3862036
rs138409352
rs200381543
rs1887027
All
−0.20
−0.15
−0.10
−0.05
0.00
MR leave−one−out sensitivity analysis for
'CD25 on memory B cell' on 'Dilated cardiomyopathy'

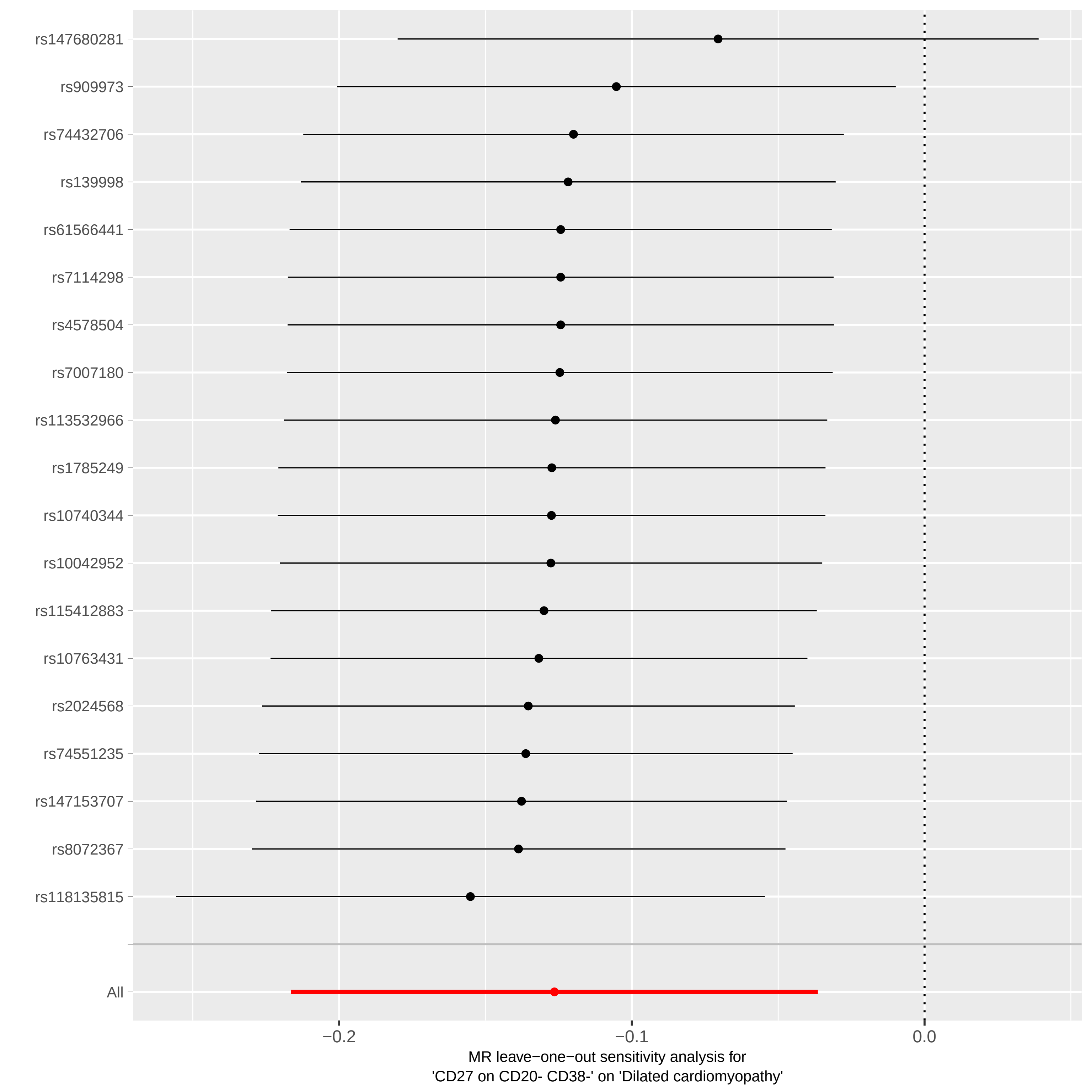

rs147680281
rs909973
rs74432706
rs139998
rs61566441
rs7114298
rs4578504
rs7007180
rs113532966
rs1785249
rs10740344
rs10042952
rs115412883
rs10763431
rs2024568
rs74551235
rs147153707
rs8072367
rs118135815
All
−0.2
−0.1
0.0
MR leave−one−out sensitivity analysis for
'CD27 on CD20- CD38-' on 'Dilated cardiomyopathy'

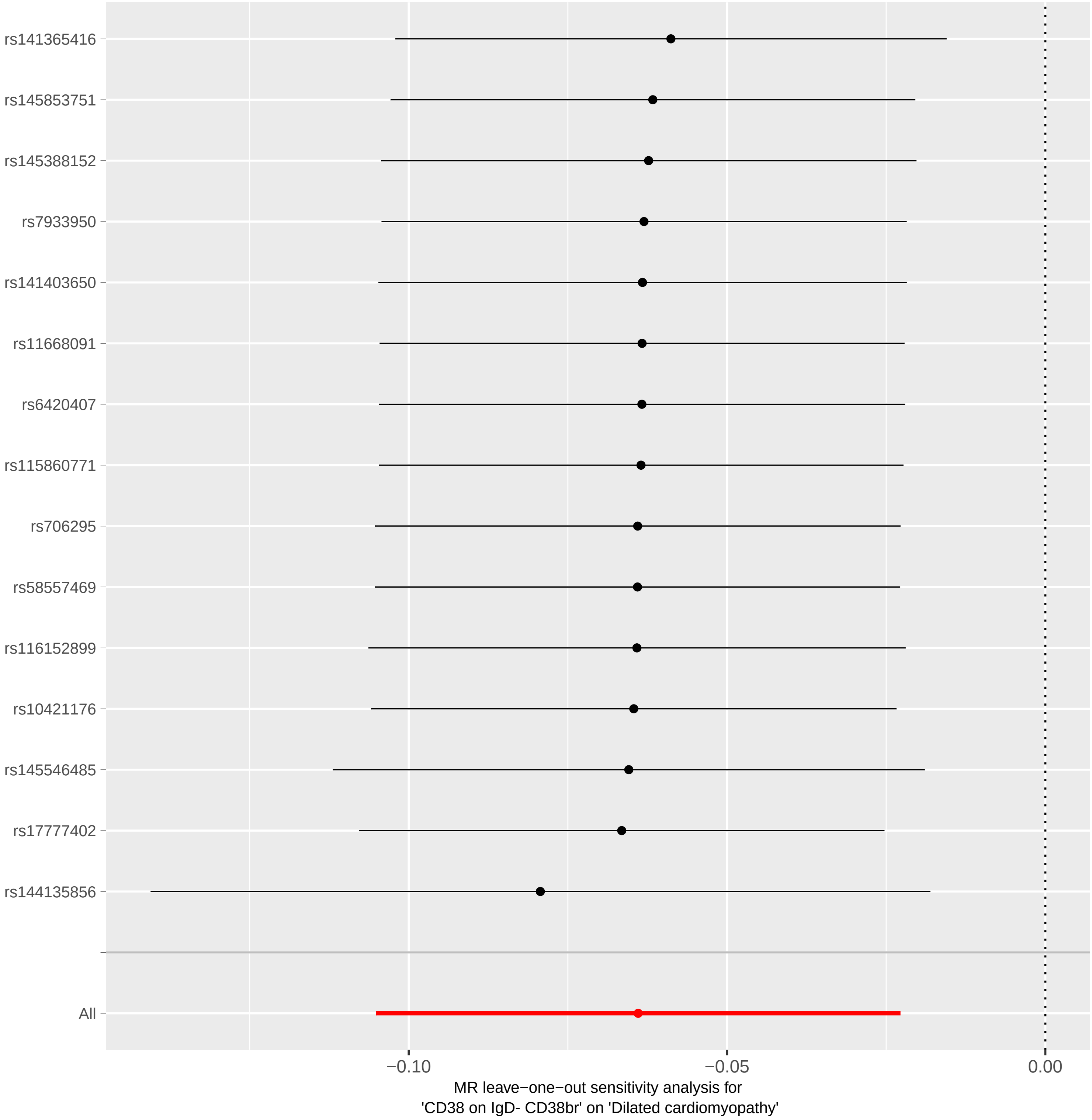
rs141365416
rs145853751
rs145388152
rs7933950
rs141403650
rs11668091
rs6420407
rs115860771
rs706295
rs58557469
rs116152899
rs10421176
rs145546485
rs17777402
rs144135856
All
−0.10
−0.05
0.00
MR leave−one−out sensitivity analysis for
'CD38 on IgD- CD38br' on 'Dilated cardiomyopathy'

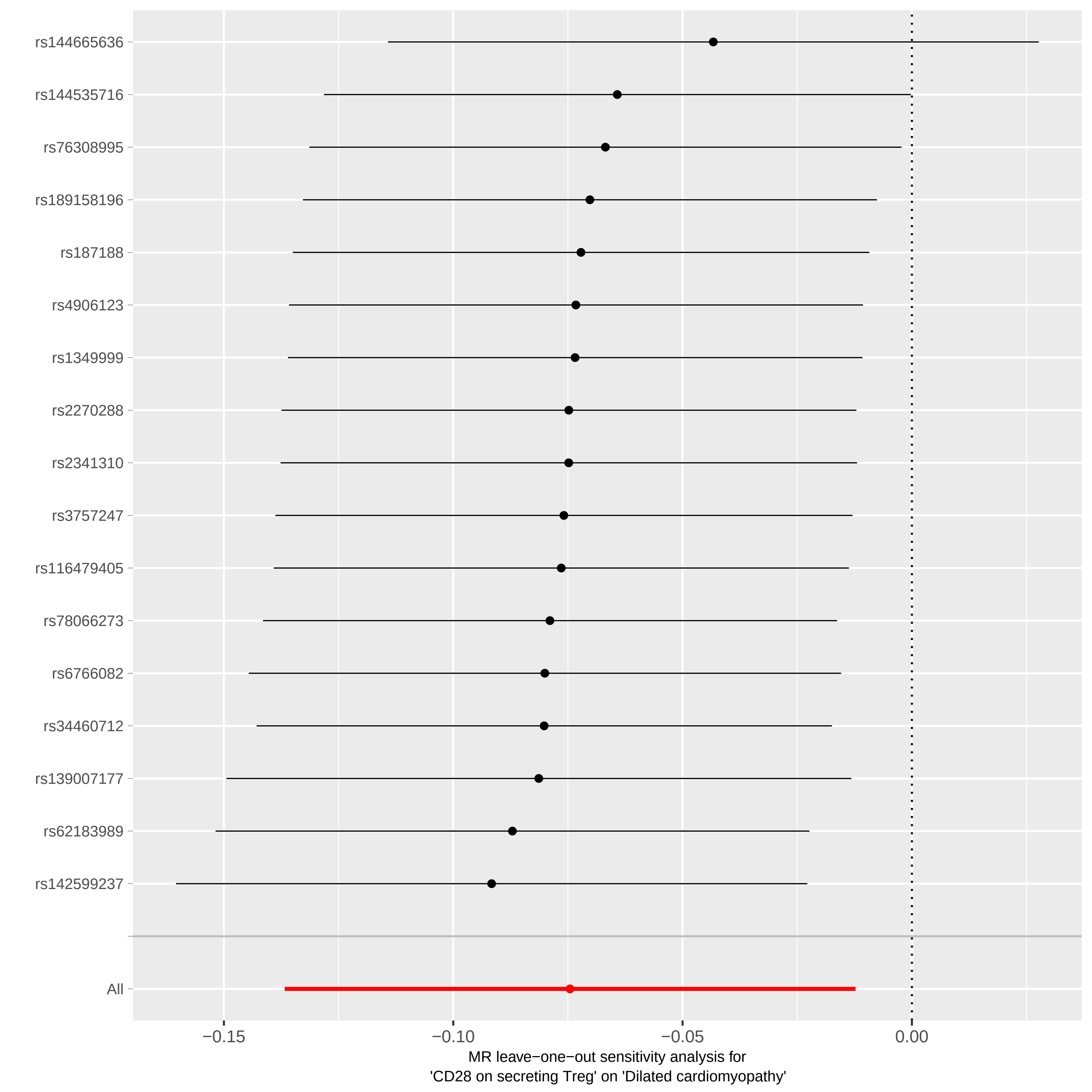
rs144665636
rs144535716
rs76308995
rs189158196
rs187188
rs4906123
rs1349999
rs2270288
rs2341310
rs3757247
rs116479405
rs78066273
rs6766082
rs34460712
rs139007177
rs62183989
rs142599237
All
−0.15
−0.10
−0.05
0.00
MR leave−one−out sensitivity analysis for
'CD28 on secreting Treg' on 'Dilated cardiomyopathy'

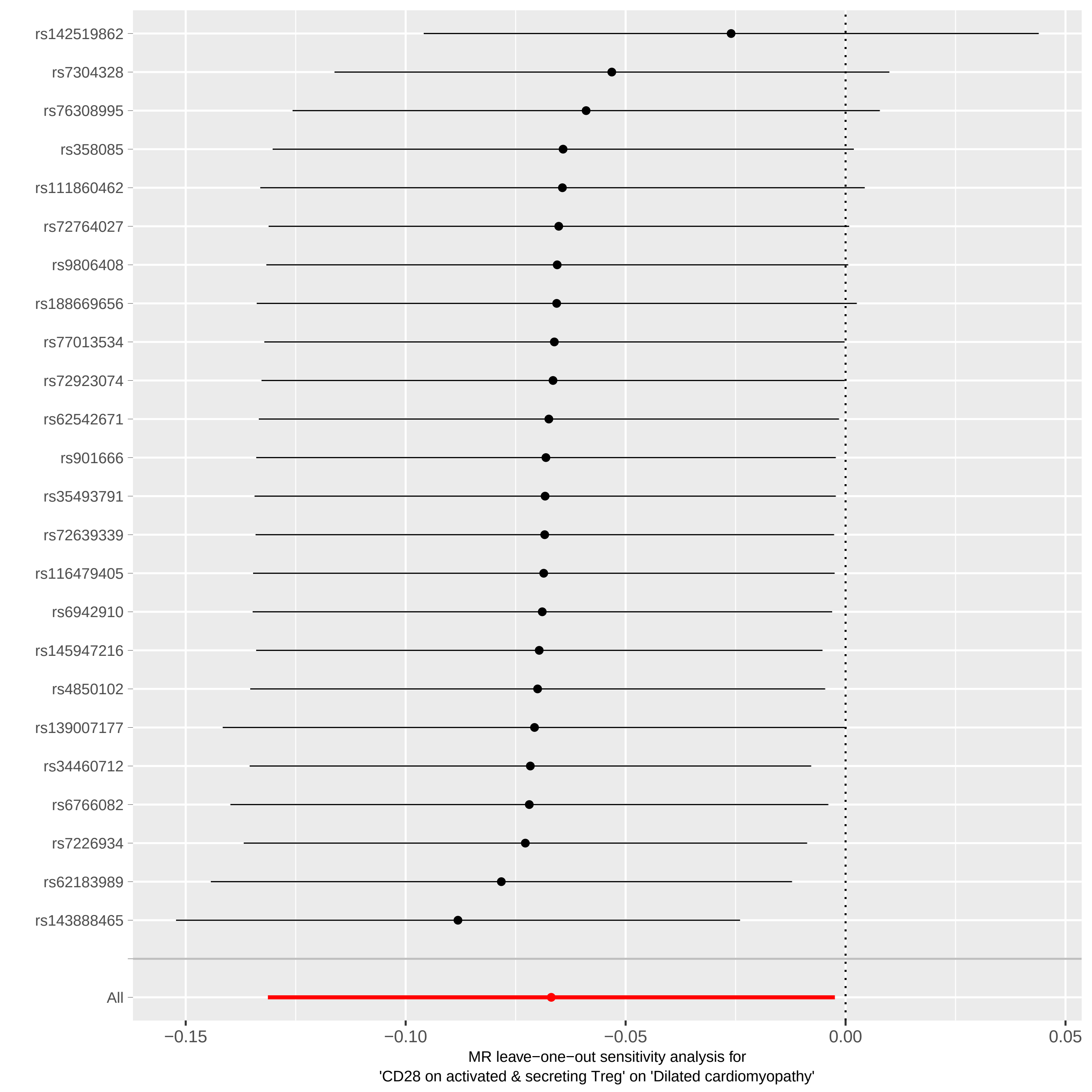
rs142519862
rs7304328
rs76308995
rs358085
rs111860462
rs72764027
rs9806408
rs188669656
rs77013534
rs72923074
rs62542671
rs901666
rs35493791
rs72639339
rs116479405
rs6942910
rs145947216
rs4850102
rs139007177
rs34460712
rs6766082
rs7226934
rs62183989
rs143888465
All
−0.15
−0.10
−0.05
0.00
0.05
MR leave−one−out sensitivity analysis for
'CD28 on activated & secreting Treg' on 'Dilated cardiomyopathy'

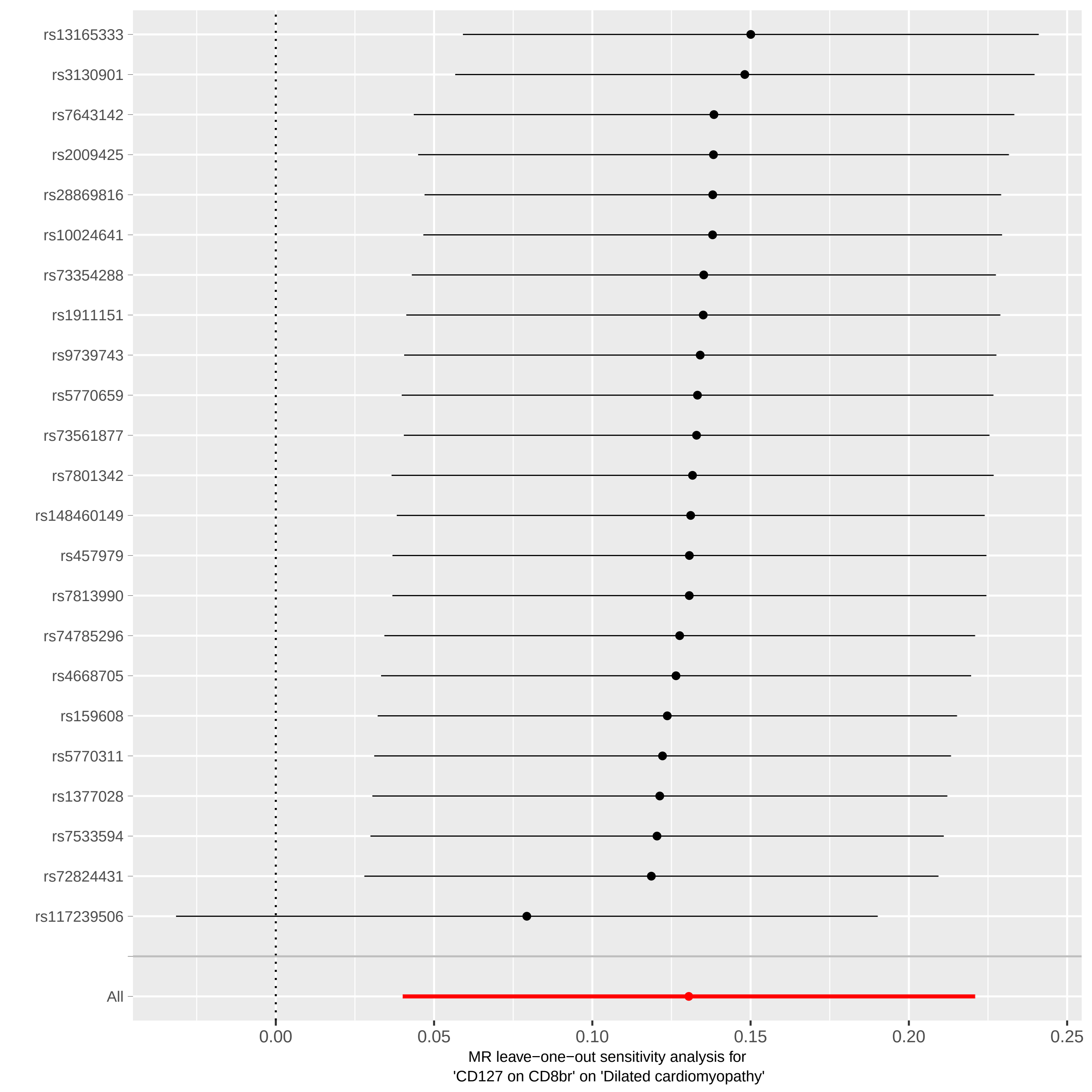
rs13165333
rs3130901
rs7643142
rs2009425
rs28869816
rs10024641
rs73354288
rs1911151
rs9739743
rs5770659
rs73561877
rs7801342
rs148460149
rs457979
rs7813990
rs74785296
rs4668705
rs159608
rs5770311
rs1377028
rs7533594
rs72824431
rs117239506
All
0.00
0.05
0.10
0.15
0.20
0.25
MR leave−one−out sensitivity analysis for
'CD127 on CD8br' on 'Dilated cardiomyopathy'

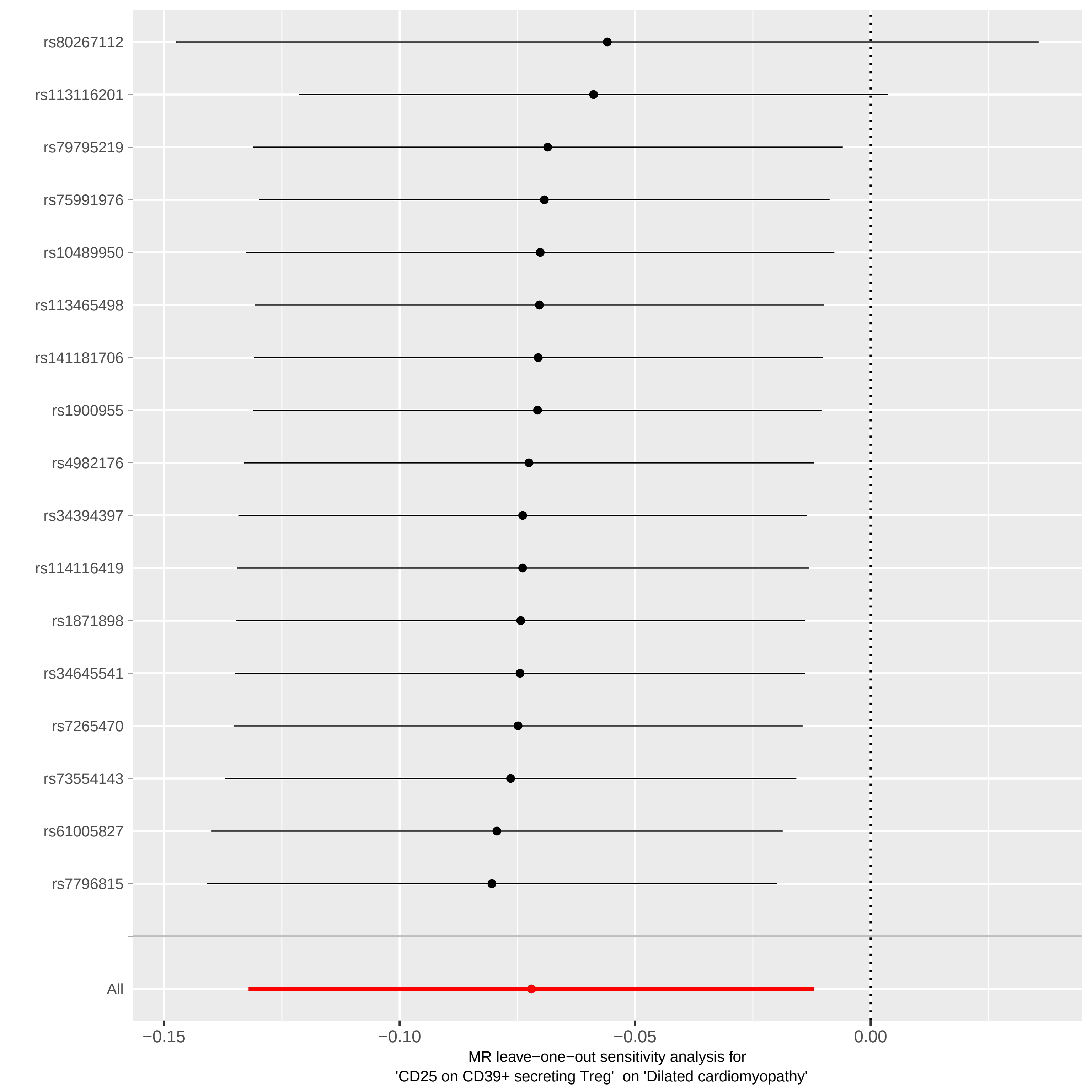

rs80267112
rs113116201
rs79795219
rs75991976
rs10489950
rs113465498
rs141181706
rs1900955
rs4982176
rs34394397
rs114116419
rs1871898
rs34645541
rs7265470
rs73554143
rs61005827
rs7796815
All
−0.15
−0.10
−0.05
0.00
MR leave−one−out sensitivity analysis for
'CD25 on CD39+ secreting Treg' on 'Dilated cardiomyopathy'

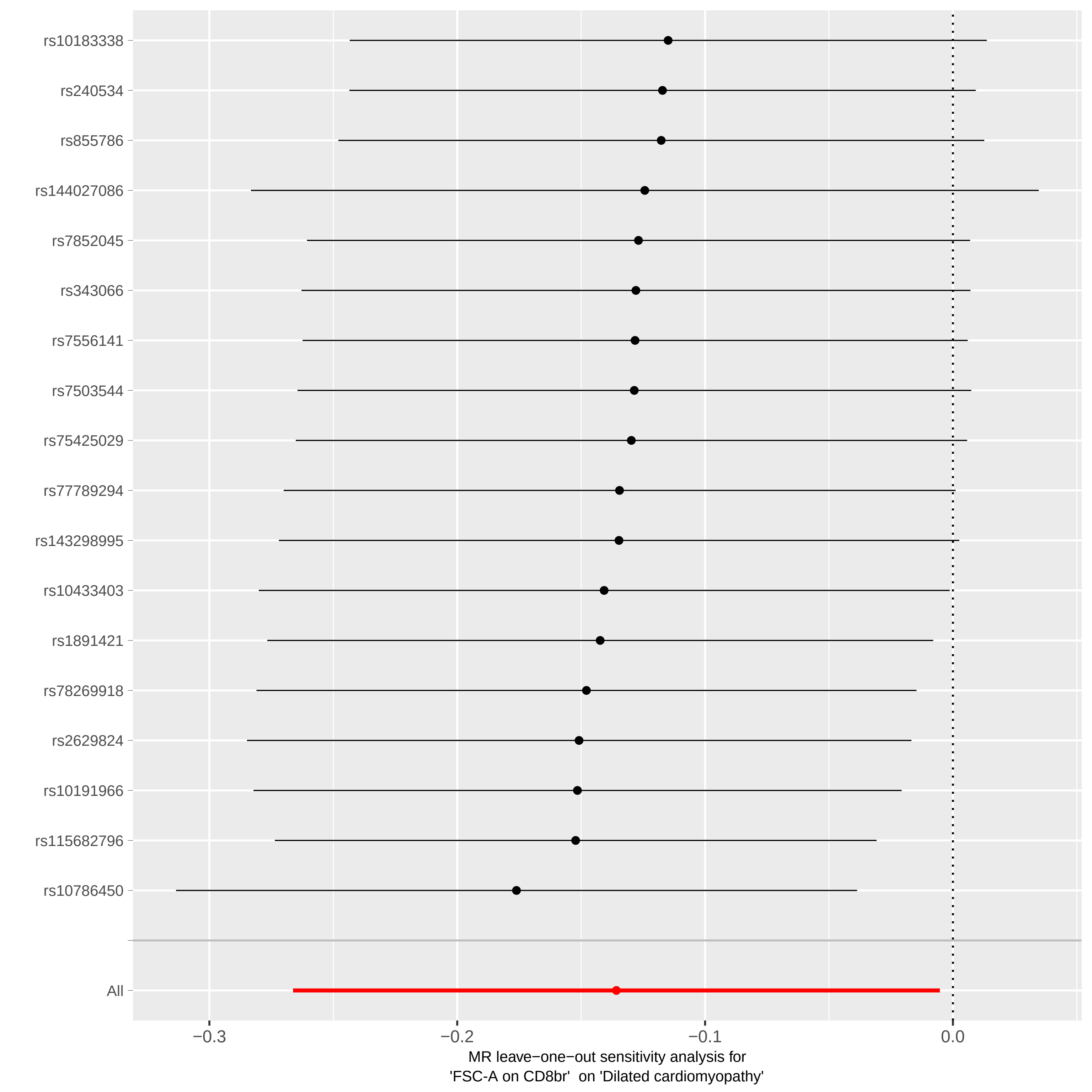
rs10183338
rs240534
rs855786
rs144027086
rs7852045
rs343066
rs7556141
rs7503544
rs75425029
rs77789294
rs143298995
rs10433403
rs1891421
rs78269918
rs2629824
rs10191966
rs115682796
rs10786450
All
−0.3
−0.2
−0.1
0.0
MR leave−one−out sensitivity analysis for
'FSC-A on CD8br' on 'Dilated cardiomyopathy'

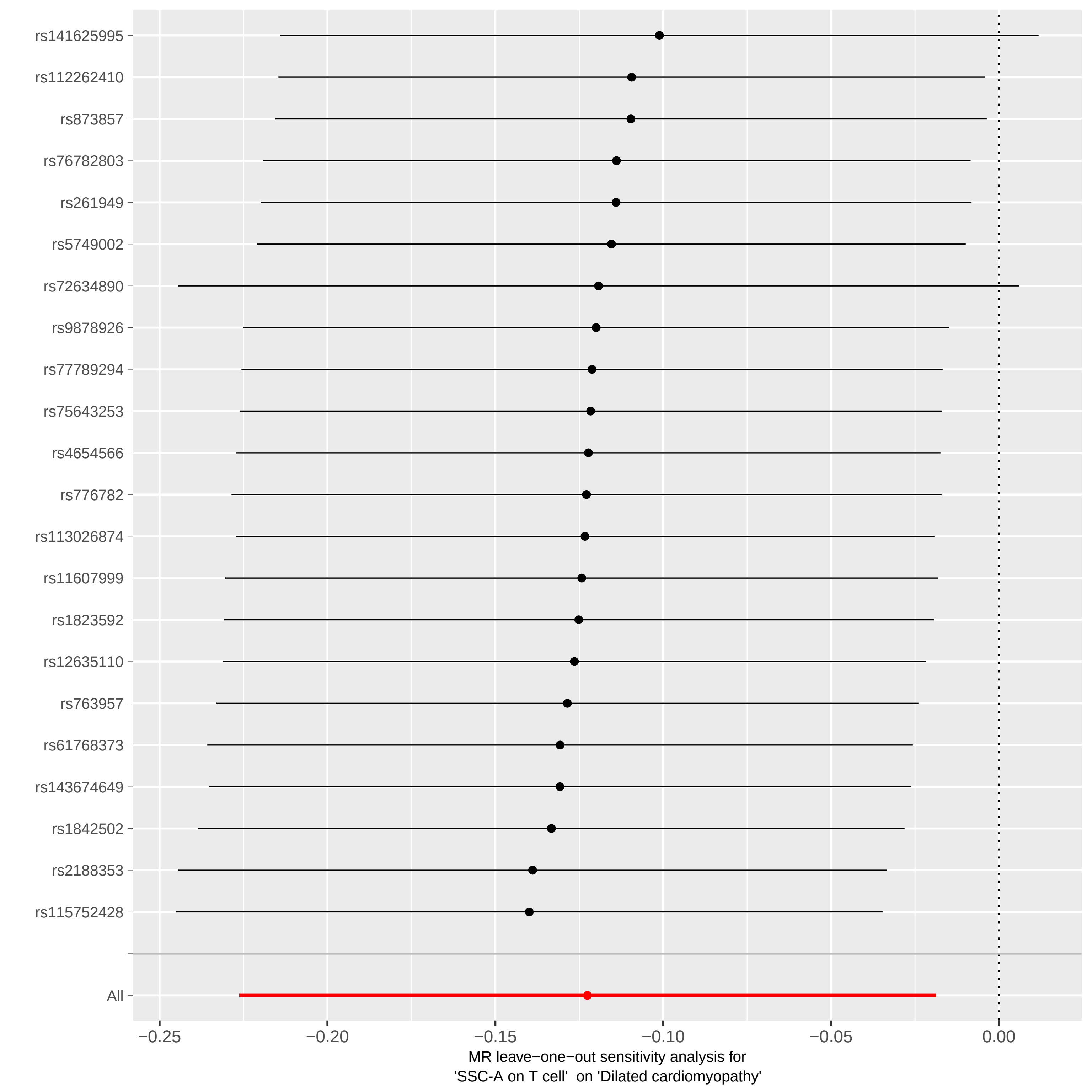

rs141625995
rs112262410
rs873857
rs76782803
rs261949
rs5749002
rs72634890
rs9878926
rs77789294
rs75643253
rs4654566
rs776782
rs113026874
rs11607999
rs1823592
rs12635110
rs763957
rs61768373
rs143674649
rs1842502
rs2188353
rs115752428
All
−0.25
−0.20
−0.15
−0.10
−0.05
0.00
MR leave−one−out sensitivity analysis for
'SSC-A on T cell' on 'Dilated cardiomyopathy'

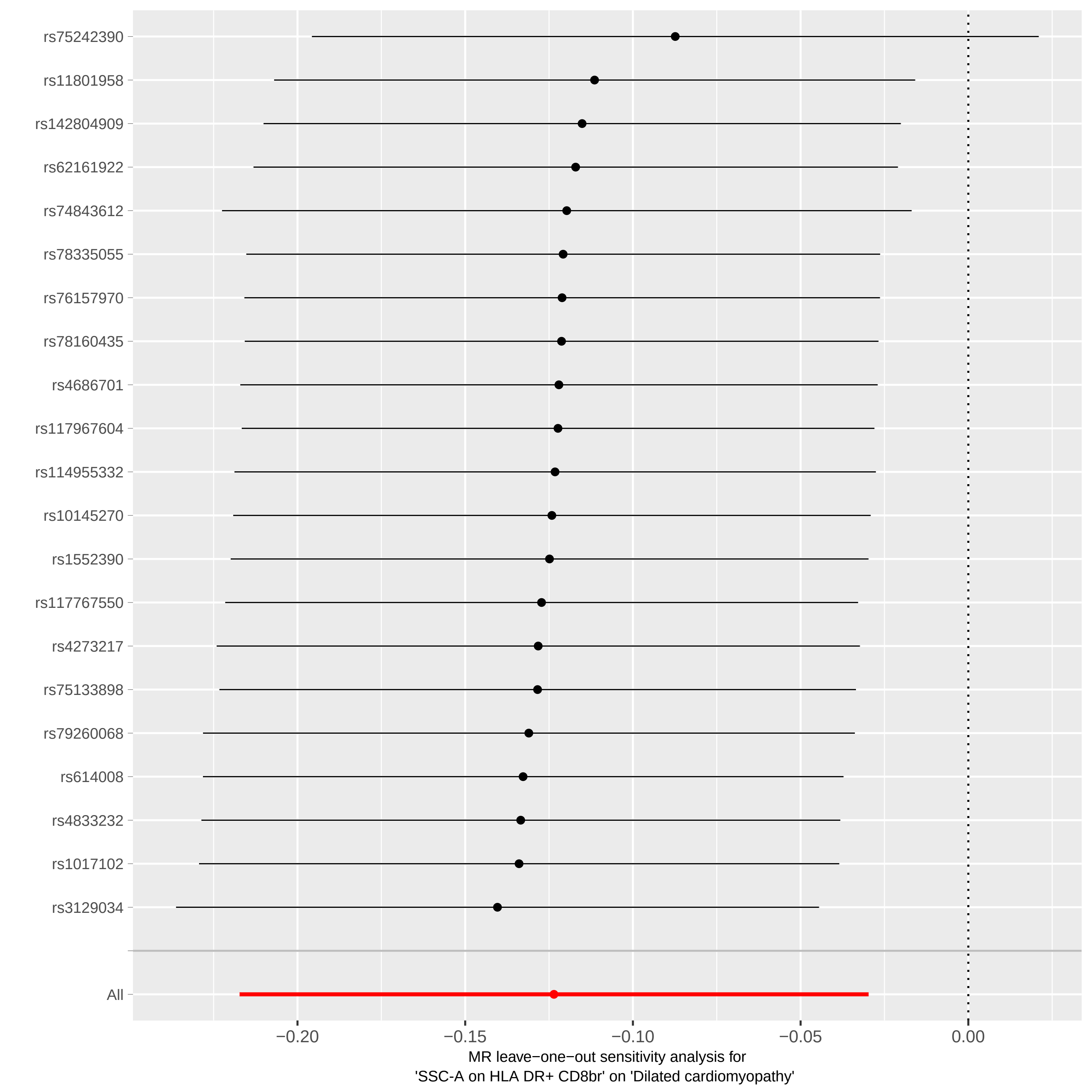
rs75242390
rs11801958
rs142804909
rs62161922
rs74843612
rs78335055
rs76157970
rs78160435
rs4686701
rs117967604
rs114955332
rs10145270
rs1552390
rs117767550
rs4273217
rs75133898
rs79260068
rs614008
rs4833232
rs1017102
rs3129034
All
−0.20
−0.15
−0.10
−0.05
0.00
MR leave−one−out sensitivity analysis for
'SSC-A on HLA DR+ CD8br' on 'Dilated cardiomyopathy'

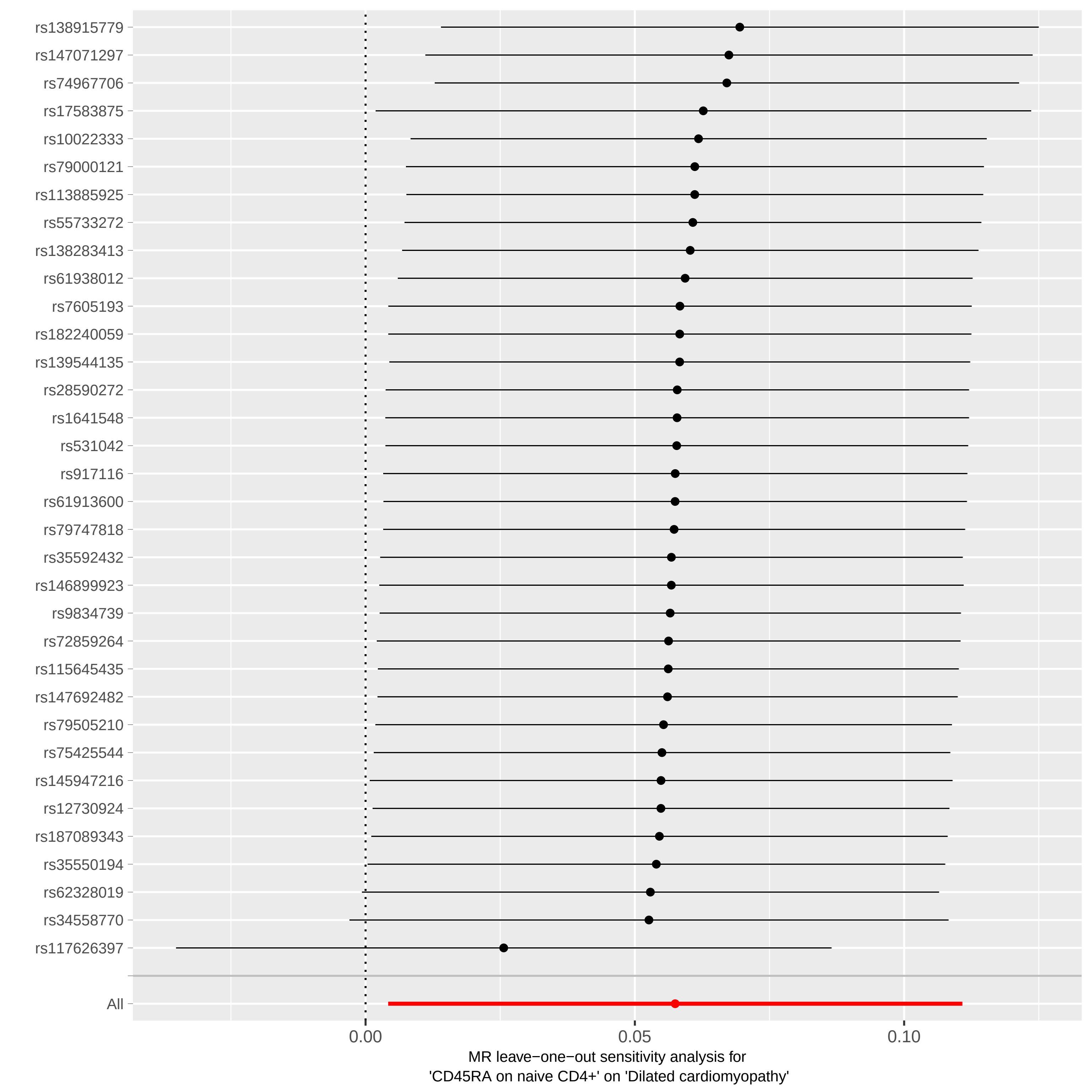
rs138915779
rs147071297
rs74967706
rs17583875
rs10022333
rs79000121
rs113885925
rs55733272
rs138283413
rs61938012
rs7605193
rs182240059
rs139544135
rs28590272
rs1641548
rs531042
rs917116
rs61913600
rs79747818
rs35592432
rs146899923
rs9834739
rs72859264
rs115645435
rs147692482
rs79505210
rs75425544
rs145947216
rs12730924
rs187089343
rs35550194
rs62328019
rs34558770
rs117626397
All
0.00
0.05
0.10
MR leave−one−out sensitivity analysis for
'CD45RA on naive CD4+' on 'Dilated cardiomyopathy'

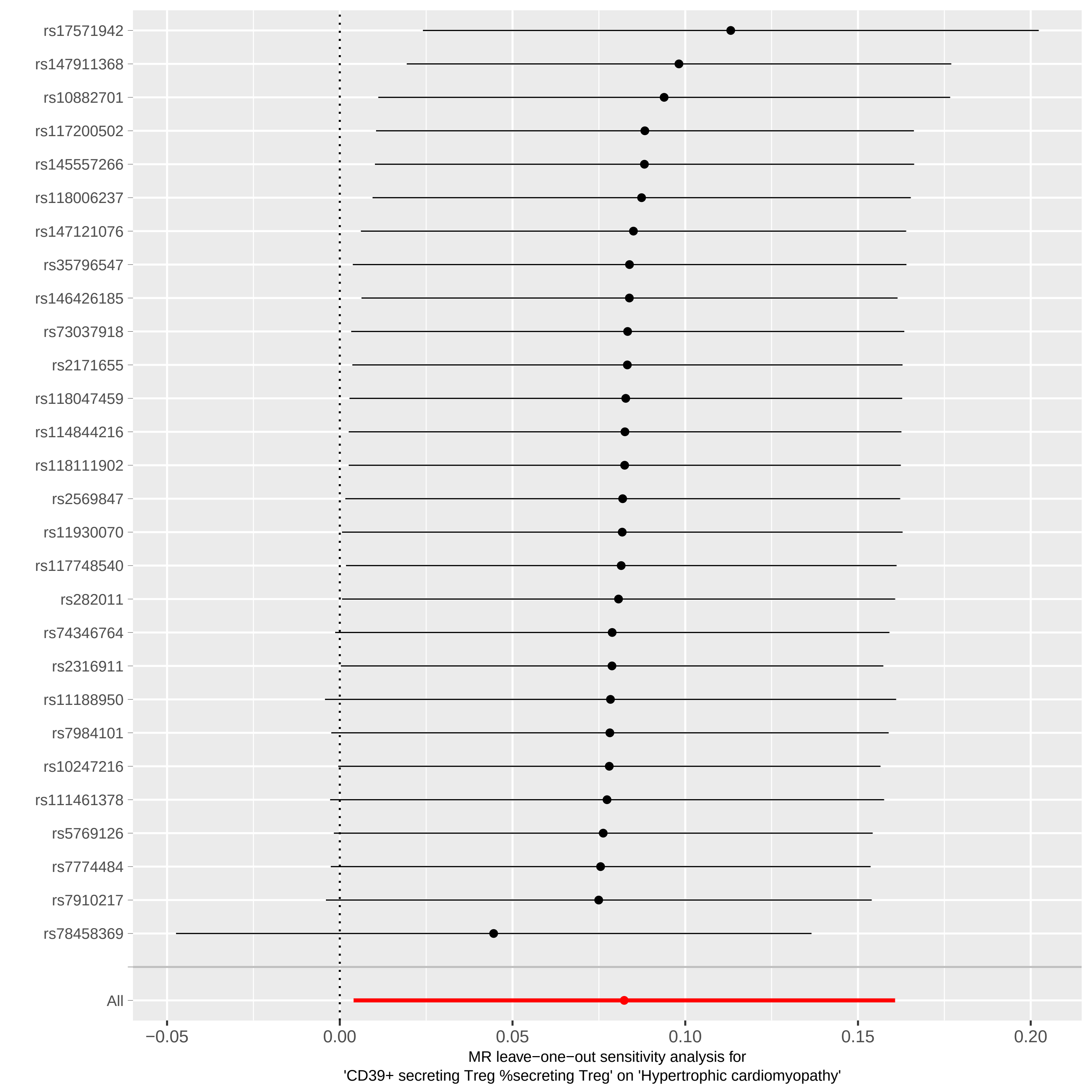

rs17571942
rs147911368
rs10882701
rs117200502
rs145557266
rs118006237
rs147121076
rs35796547
rs146426185
rs73037918
rs2171655
rs118047459
rs114844216
rs118111902
rs2569847
rs11930070
rs117748540
rs282011
rs74346764
rs2316911
rs11188950
rs7984101
rs10247216
rs111461378
rs5769126
rs7774484
rs7910217
rs78458369
All
−0.05
0.00
0.05
0.10
0.15
0.20
MR leave−one−out sensitivity analysis for
'CD39+ secreting Treg %secreting Treg' on 'Hypertrophic cardiomyopathy'

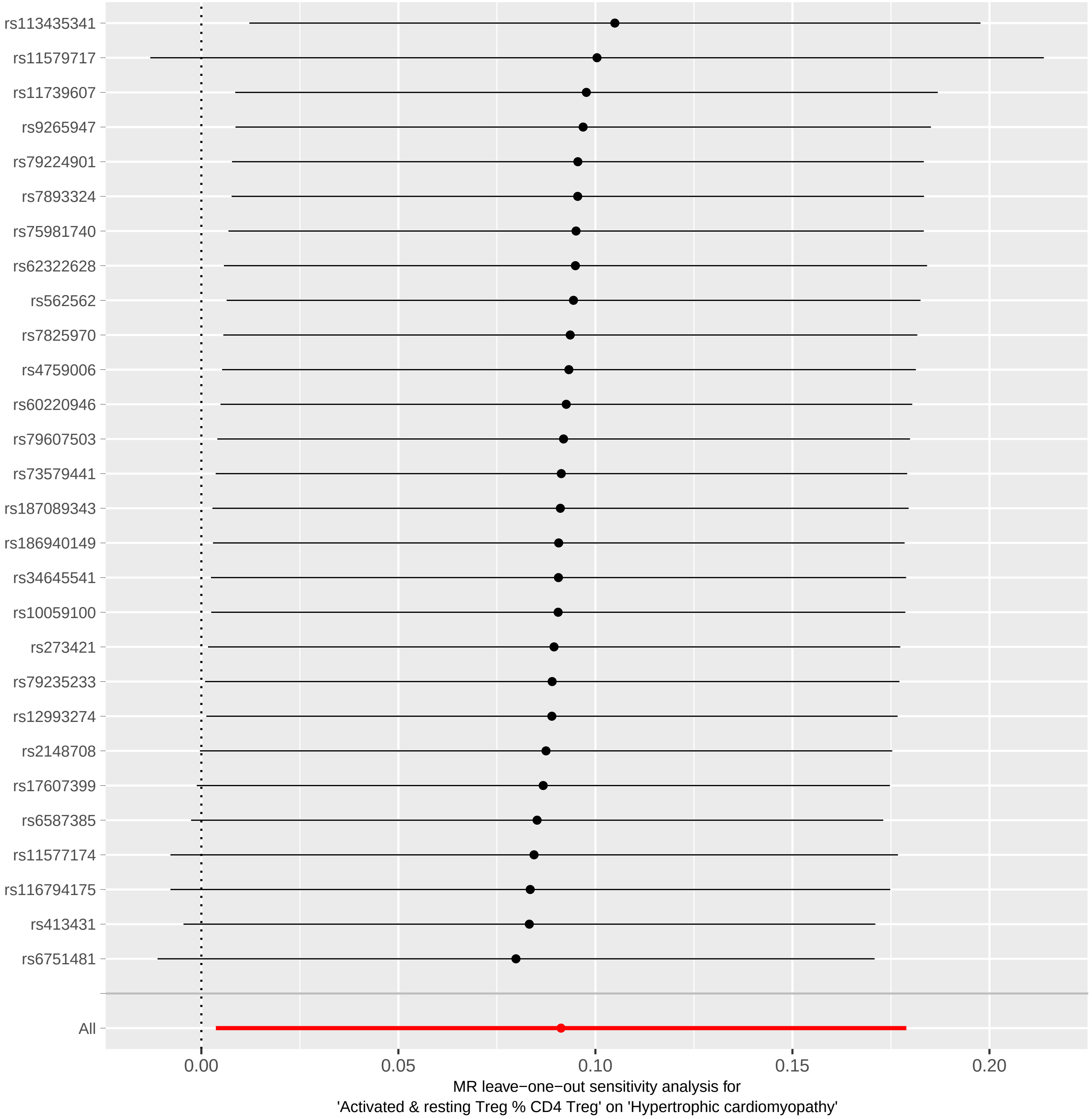
rs113435341
rs11579717
rs11739607
rs9265947
rs79224901
rs7893324
rs75981740
rs62322628
rs562562
rs7825970
rs4759006
rs60220946
rs79607503
rs73579441
rs187089343
rs186940149
rs34645541
rs10059100
rs273421
rs79235233
rs12993274
rs2148708
rs17607399
rs6587385
rs11577174
rs116794175
rs413431
rs6751481
All
0.00
0.05
0.10
0.15
0.20
MR leave−one−out sensitivity analysis for
'Activated & resting Treg % CD4 Treg' on 'Hypertrophic cardiomyopathy'

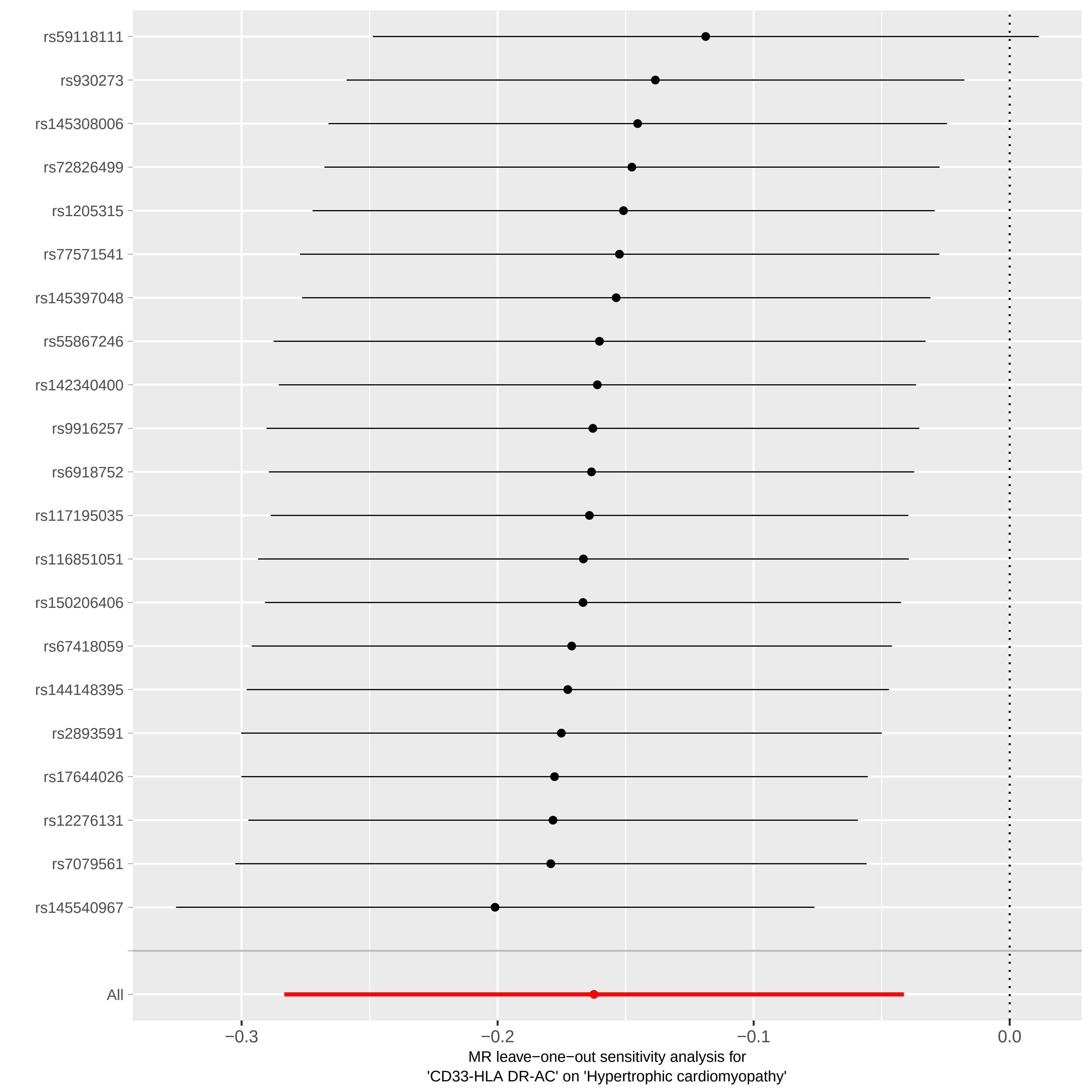
rs59118111
rs930273
rs145308006
rs72826499
rs1205315
rs77571541
rs145397048
rs55867246
rs142340400
rs9916257
rs6918752
rs117195035
rs116851051
rs150206406
rs67418059
rs144148395
rs2893591
rs17644026
rs12276131
rs7079561
rs145540967
All
−0.3
−0.2
−0.1
0.0
MR leave−one−out sensitivity analysis for
'CD33-HLA DR-AC' on 'Hypertrophic cardiomyopathy'

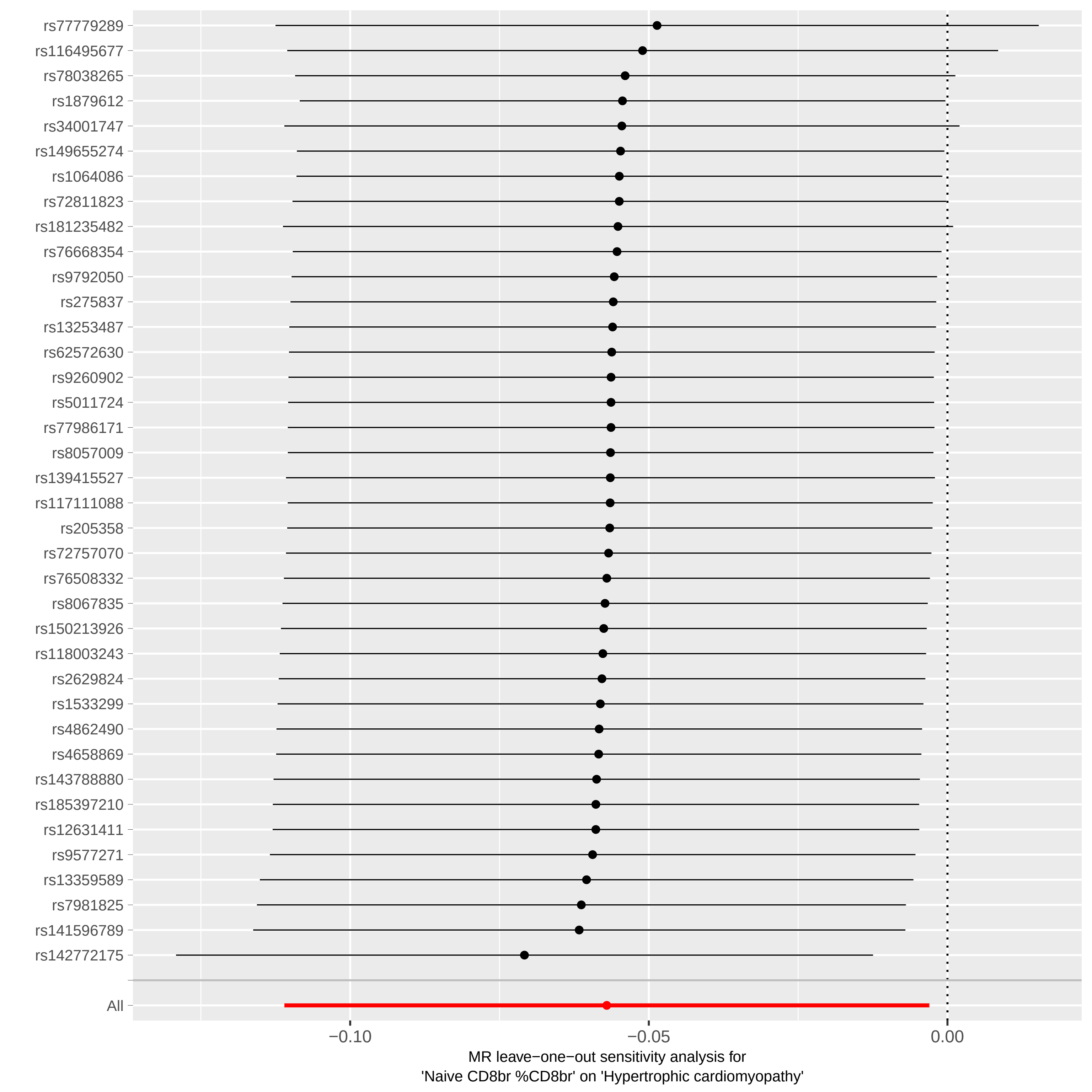

rs77779289
rs116495677
rs78038265
rs1879612
rs34001747
rs149655274
rs1064086
rs72811823
rs181235482
rs76668354
rs9792050
rs275837
rs13253487
rs62572630
rs9260902
rs5011724
rs77986171
rs8057009
rs139415527
rs117111088
rs205358
rs72757070
rs76508332
rs8067835
rs150213926
rs118003243
rs2629824
rs1533299
rs4862490
rs4658869
rs143788880
rs185397210
rs12631411
rs9577271
rs13359589
rs7981825
rs141596789
rs142772175
All
−0.10
−0.05
0.00
MR leave−one−out sensitivity analysis for
'Naive CD8br %CD8br' on 'Hypertrophic cardiomyopathy'

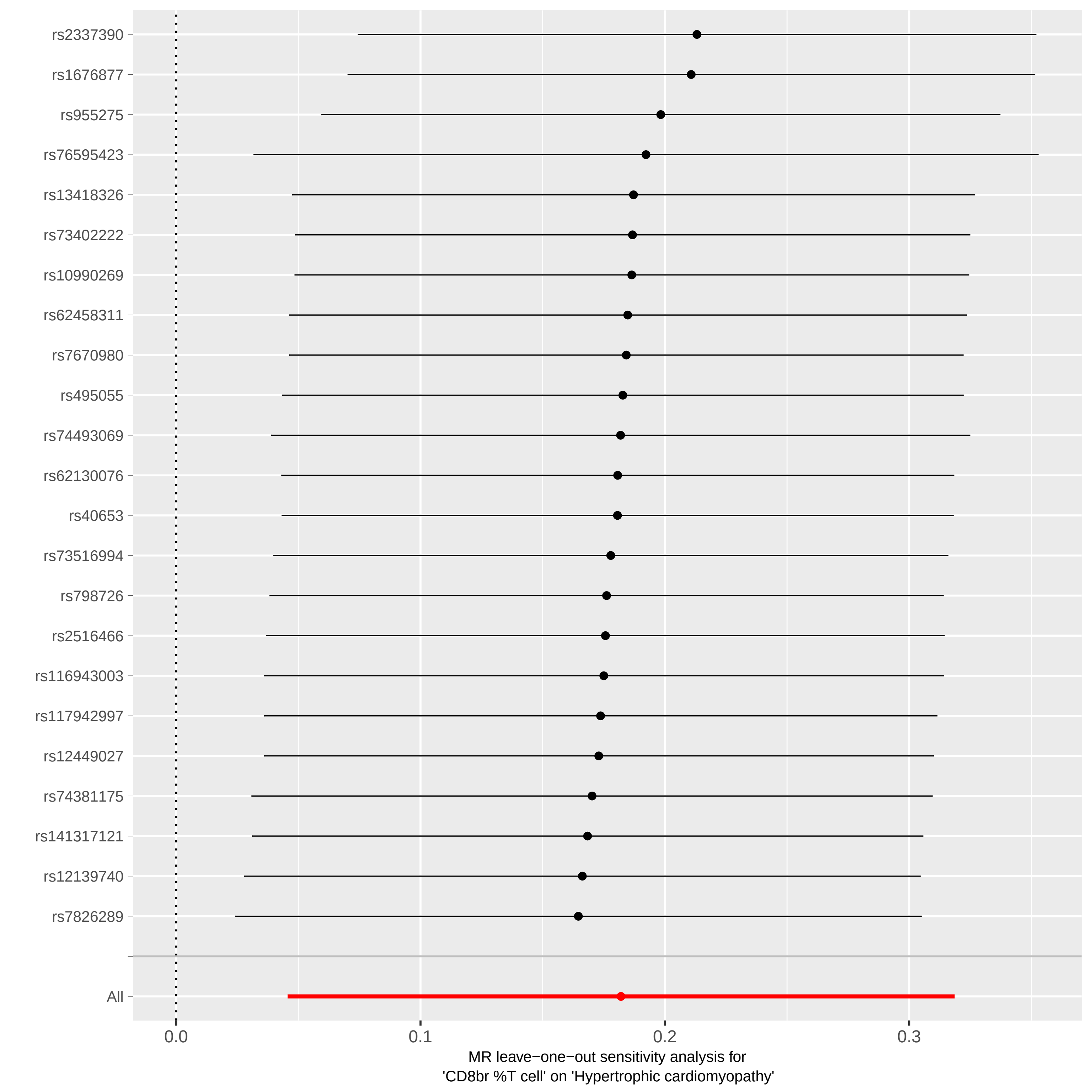
rs2337390
rs1676877
rs955275
rs76595423
rs13418326
rs73402222
rs10990269
rs62458311
rs7670980
rs495055
rs74493069
rs62130076
rs40653
rs73516994
rs798726
rs2516466
rs116943003
rs117942997
rs12449027
rs74381175
rs141317121
rs12139740
rs7826289
All
0.0
0.1
0.2
0.3
MR leave−one−out sensitivity analysis for
'CD8br %T cell' on 'Hypertrophic cardiomyopathy'

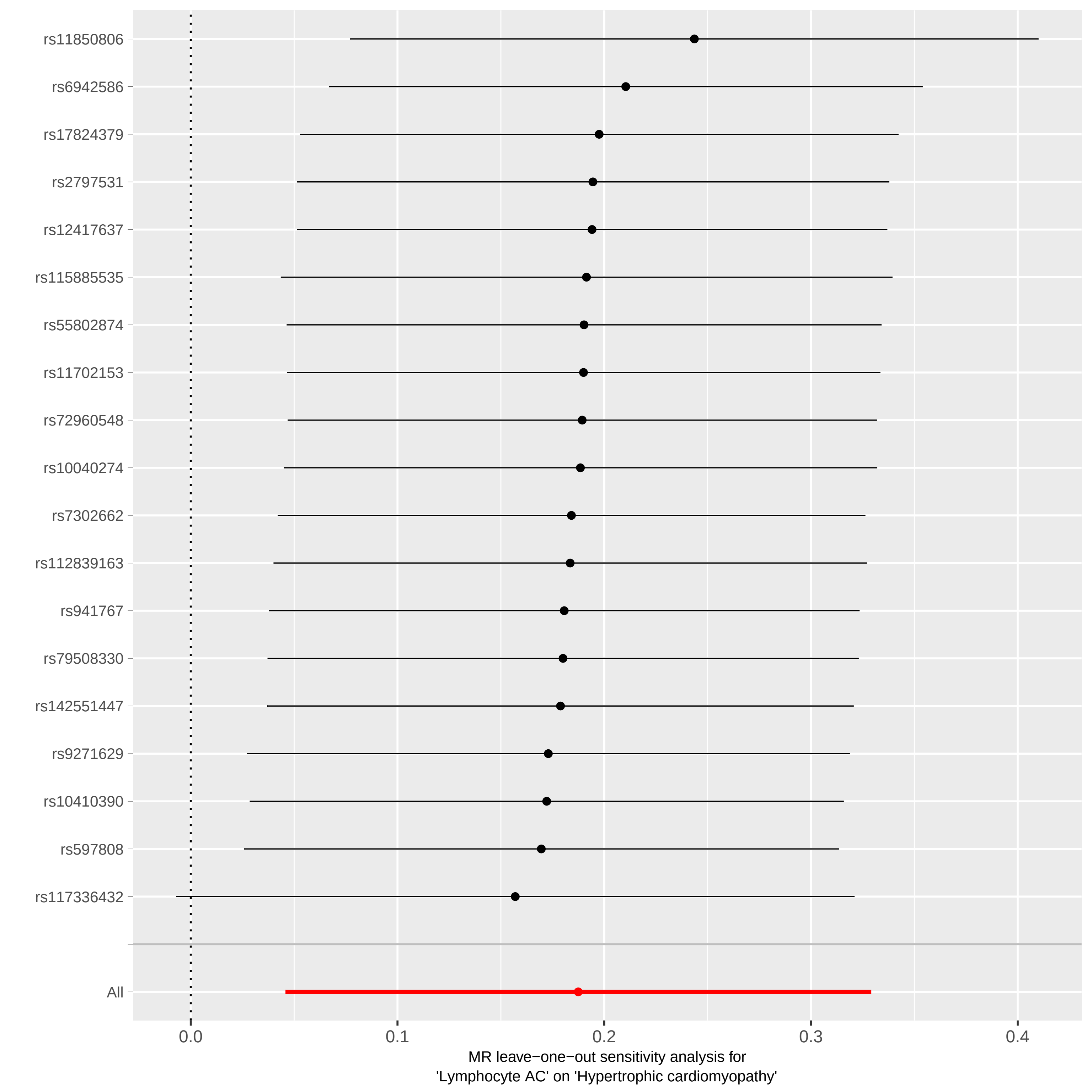
rs11850806
rs6942586
rs17824379
rs2797531
rs12417637
rs115885535
rs55802874
rs11702153
rs72960548
rs10040274
rs7302662
rs112839163
rs941767
rs79508330
rs142551447
rs9271629
rs10410390
rs597808
rs117336432
All
0.0
0.1
0.2
0.3
0.4
MR leave−one−out sensitivity analysis for
'Lymphocyte AC' on 'Hypertrophic cardiomyopathy'

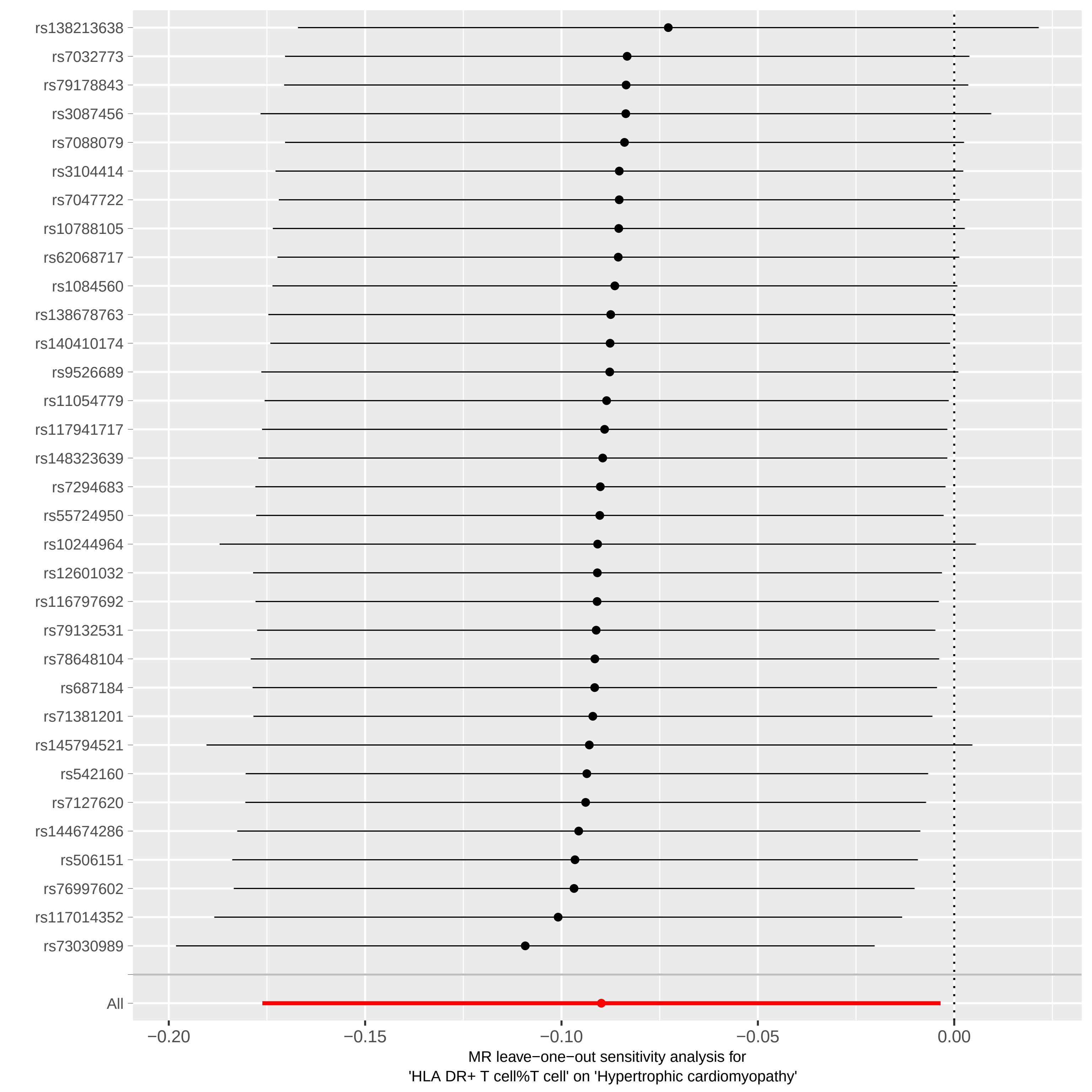
rs138213638
rs7032773
rs79178843
rs3087456
rs7088079
rs3104414
rs7047722
rs10788105
rs62068717
rs1084560
rs138678763
rs140410174
rs9526689
rs11054779
rs117941717
rs148323639
rs7294683
rs55724950
rs10244964
rs12601032
rs116797692
rs79132531
rs78648104
rs687184
rs71381201
rs145794521
rs542160
rs7127620
rs144674286
rs506151
rs76997602
rs117014352
rs73030989
All
−0.20
−0.15
−0.10
−0.05
0.00
MR leave−one−out sensitivity analysis for
'HLA DR+ T cell%T cell' on 'Hypertrophic cardiomyopathy'

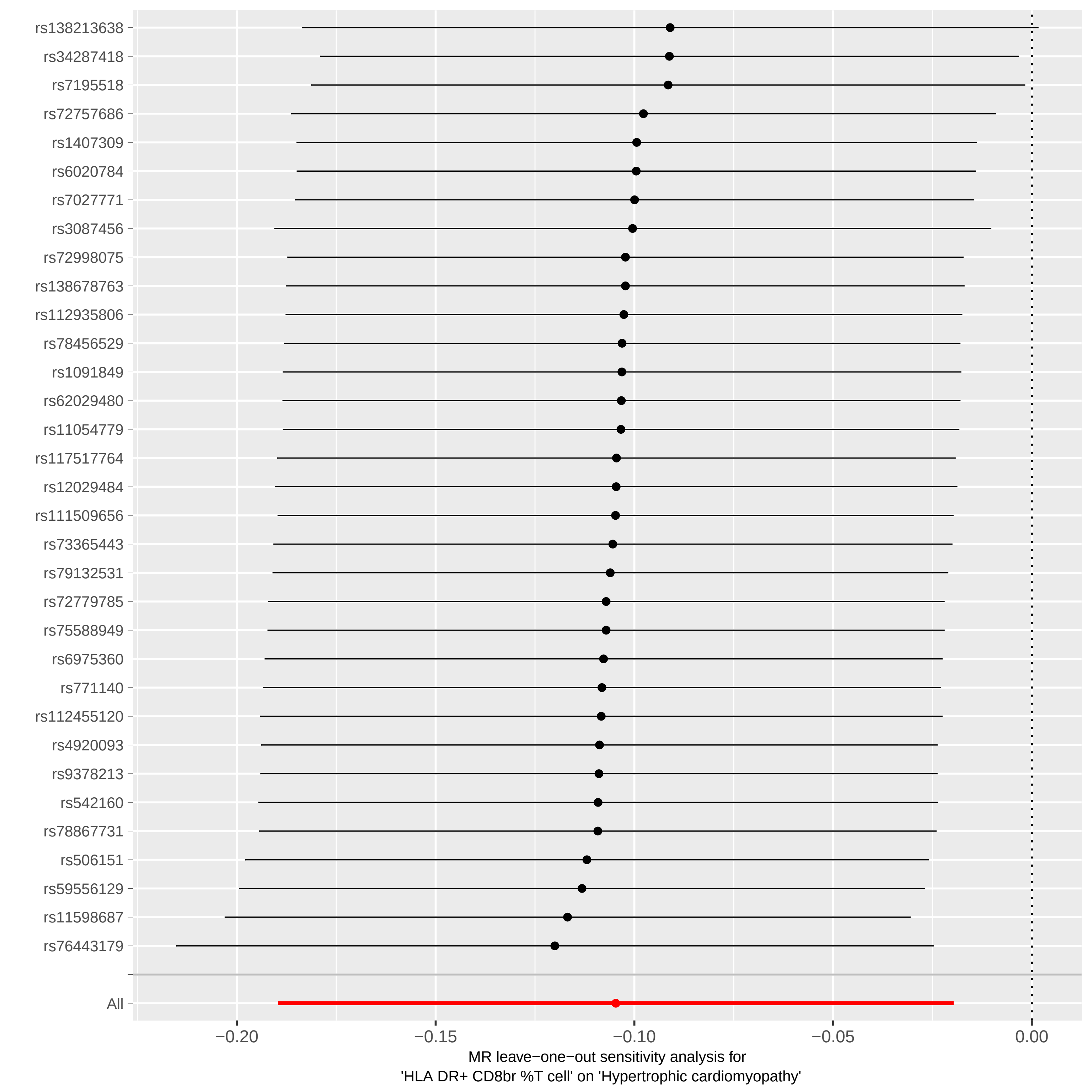
rs138213638
rs34287418
rs7195518
rs72757686
rs1407309
rs6020784
rs7027771
rs3087456
rs72998075
rs138678763
rs112935806
rs78456529
rs1091849
rs62029480
rs11054779
rs117517764
rs12029484
rs111509656
rs73365443
rs79132531
rs72779785
rs75588949
rs6975360
rs771140
rs112455120
rs4920093
rs9378213
rs542160
rs78867731
rs506151
rs59556129
rs11598687
rs76443179
All
−0.20
−0.15
−0.10
−0.05
0.00
MR leave−one−out sensitivity analysis for
'HLA DR+ CD8br %T cell' on 'Hypertrophic cardiomyopathy'

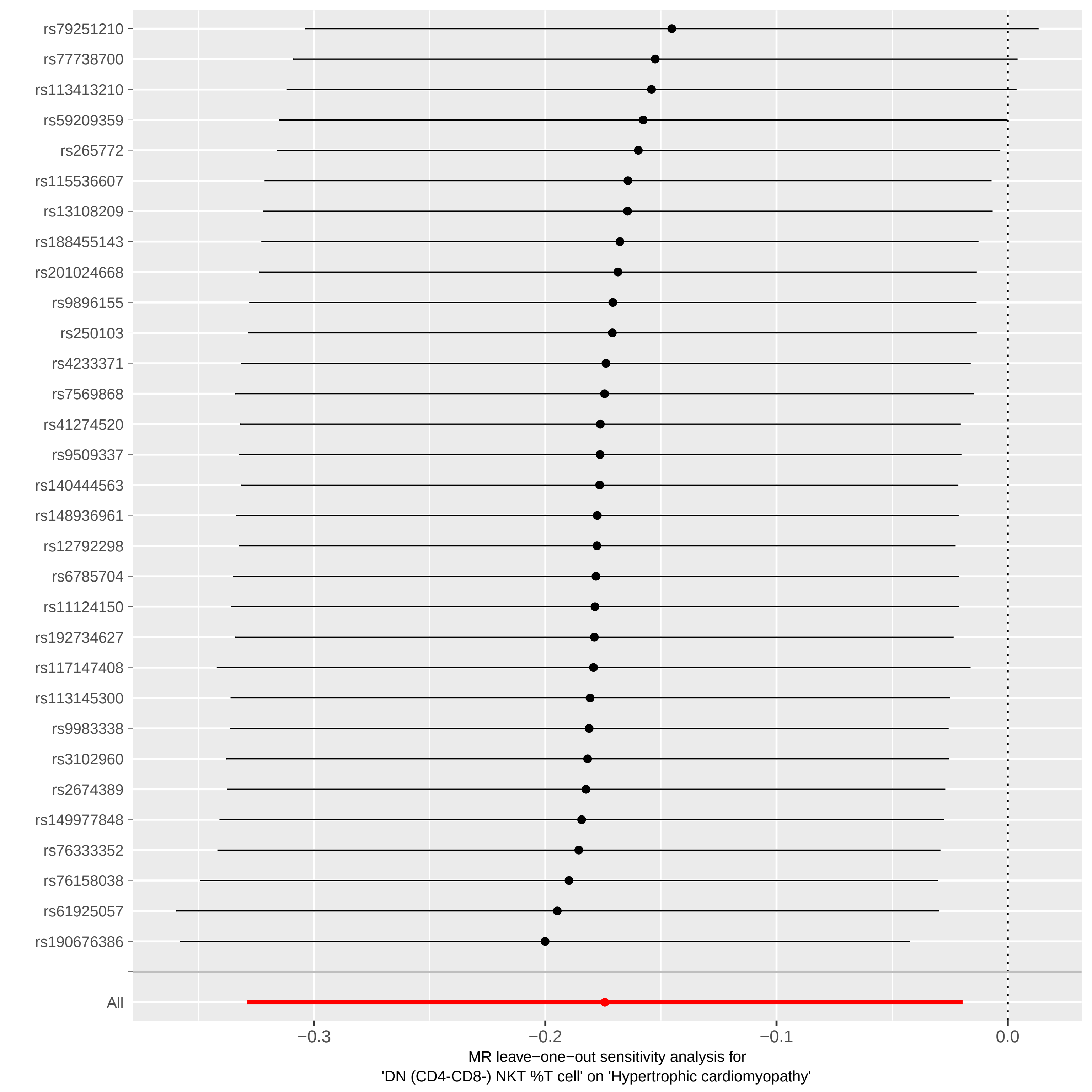
rs79251210
rs77738700
rs113413210
rs59209359
rs265772
rs115536607
rs13108209
rs188455143
rs201024668
rs9896155
rs250103
rs4233371
rs7569868
rs41274520
rs9509337
rs140444563
rs148936961
rs12792298
rs6785704
rs11124150
rs192734627
rs117147408
rs113145300
rs9983338
rs3102960
rs2674389
rs149977848
rs76333352
rs76158038
rs61925057
rs190676386
All
−0.3
−0.2
−0.1
0.0
MR leave−one−out sensitivity analysis for
'DN (CD4-CD8-) NKT %T cell' on 'Hypertrophic cardiomyopathy'

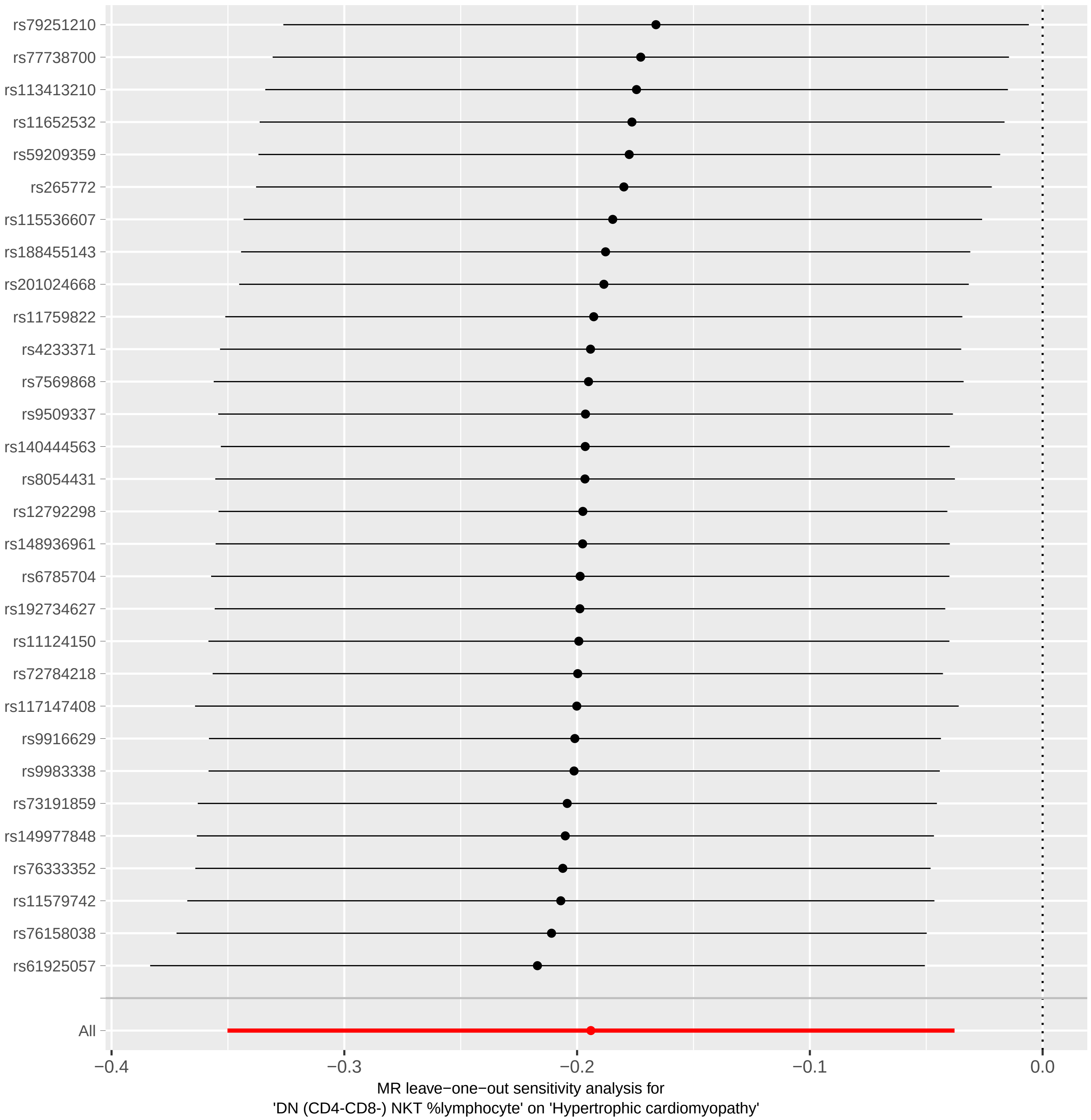
rs79251210
rs77738700
rs113413210
rs11652532
rs59209359
rs265772
rs115536607
rs188455143
rs201024668
rs11759822
rs4233371
rs7569868
rs9509337
rs140444563
rs8054431
rs12792298
rs148936961
rs6785704
rs192734627
rs11124150
rs72784218
rs117147408
rs9916629
rs9983338
rs73191859
rs149977848
rs76333352
rs11579742
rs76158038
rs61925057
All
−0.4
−0.3
−0.2
−0.1
0.0
MR leave−one−out sensitivity analysis for
'DN (CD4-CD8-) NKT %lymphocyte' on 'Hypertrophic cardiomyopathy'

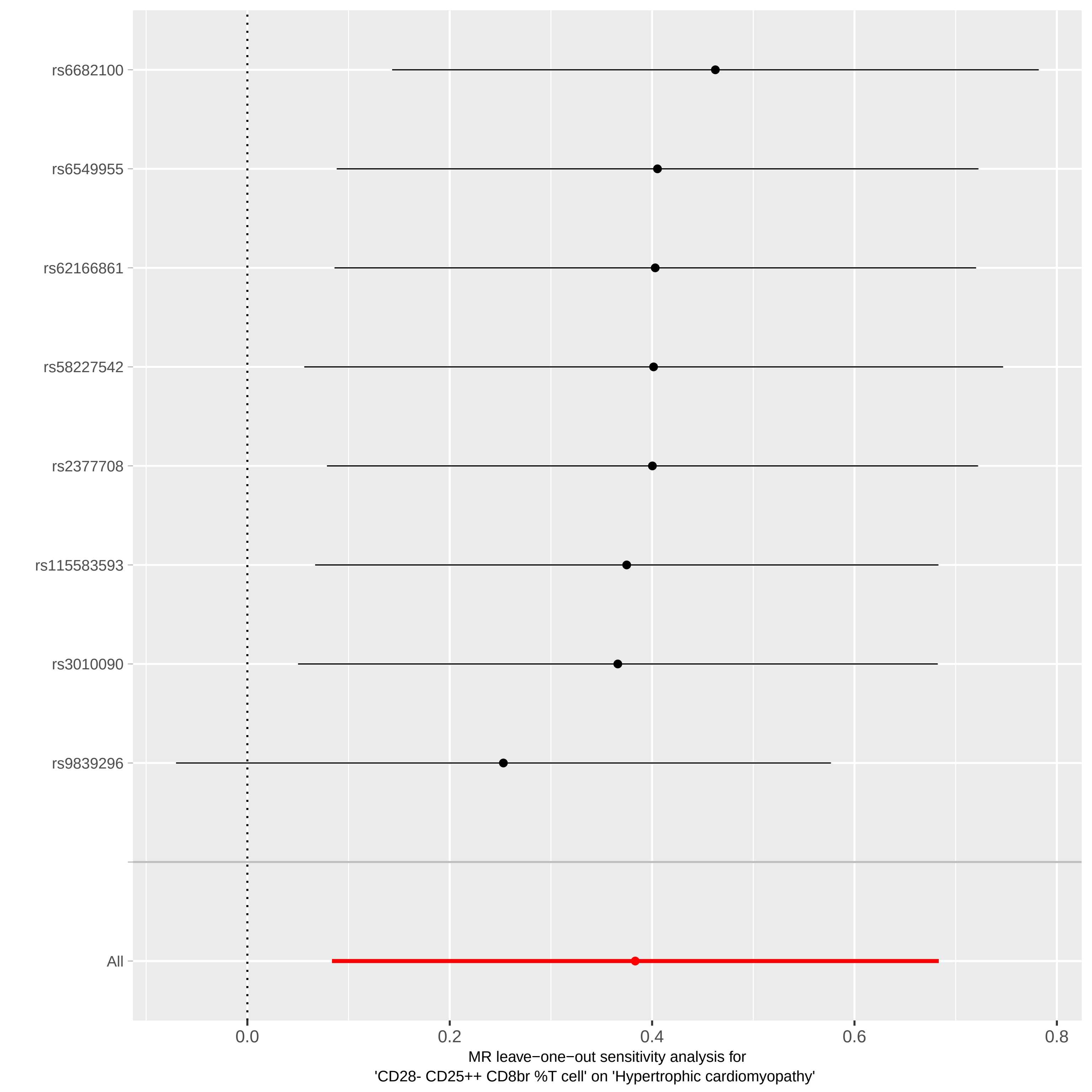

rs6682100
rs6549955
rs62166861
rs58227542
rs2377708
rs115583593
rs3010090
rs9839296
All
0.0
0.2
0.4
0.6
0.8
MR leave−one−out sensitivity analysis for
'CD28- CD25++ CD8br %T cell' on 'Hypertrophic cardiomyopathy'

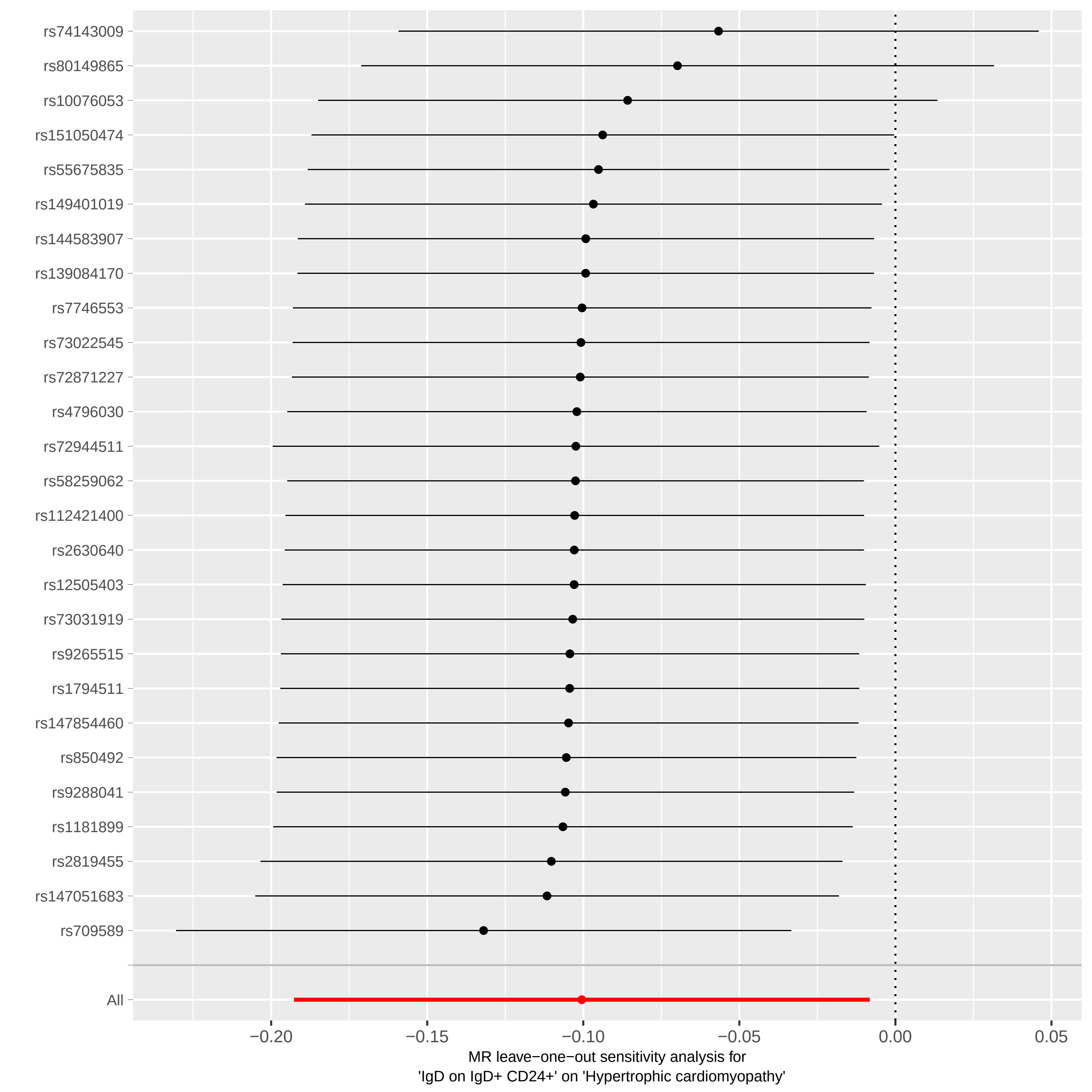

rs74143009
rs80149865
rs10076053
rs151050474
rs55675835
rs149401019
rs144583907
rs139084170
rs7746553
rs73022545
rs72871227
rs4796030
rs72944511
rs58259062
rs112421400
rs2630640
rs12505403
rs73031919
rs9265515
rs1794511
rs147854460
rs850492
rs9288041
rs1181899
rs2819455
rs147051683
rs709589
All
−0.20
−0.15
−0.10
−0.05
0.00
0.05
MR leave−one−out sensitivity analysis for
'IgD on IgD+ CD24+' on 'Hypertrophic cardiomyopathy'

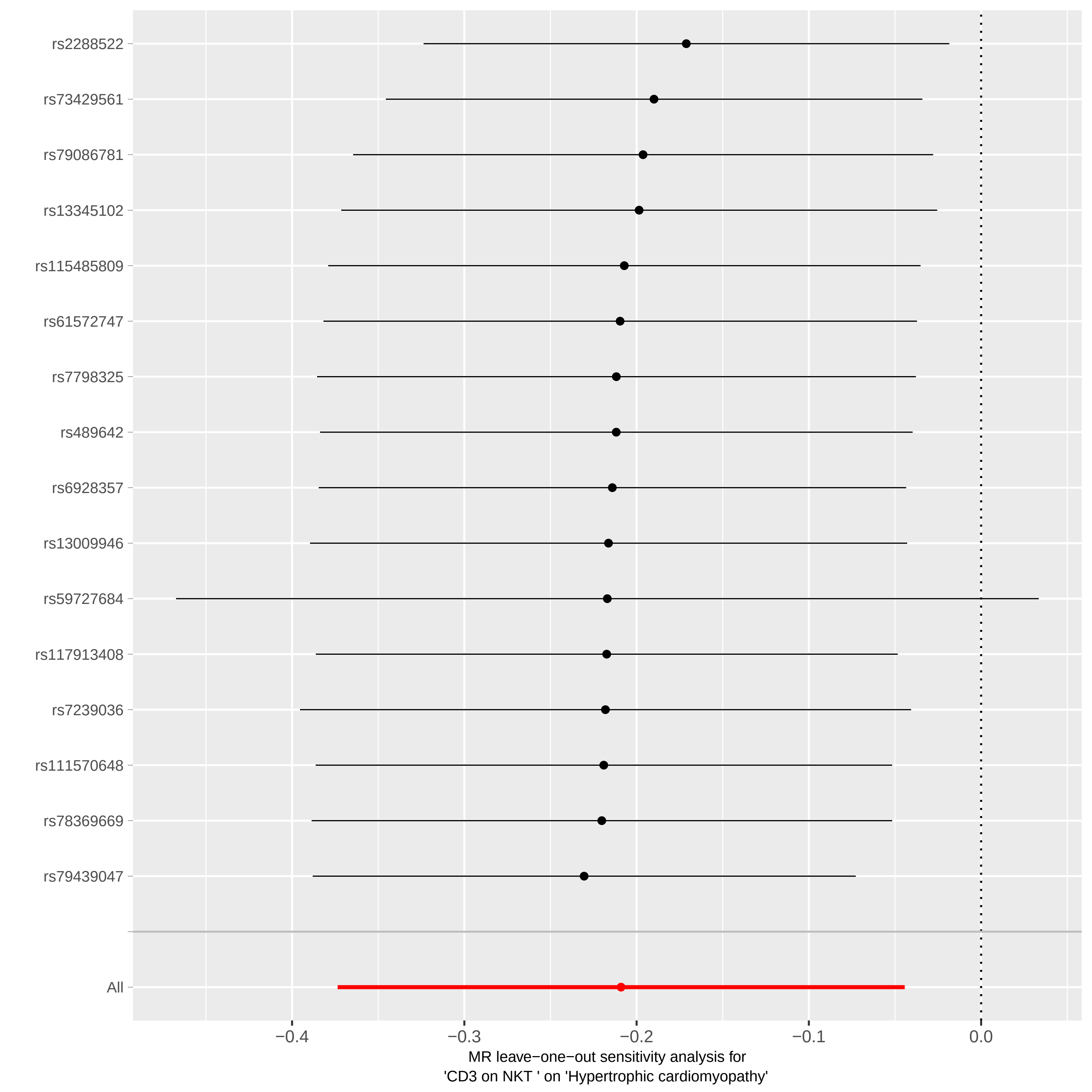

rs2288522
rs73429561
rs79086781
rs13345102
rs115485809
rs61572747
rs7798325
rs489642
rs6928357
rs13009946
rs59727684
rs117913408
rs7239036
rs111570648
rs78369669
rs79439047
All
−0.4
−0.3
−0.2
−0.1
0.0
MR leave−one−out sensitivity analysis for
'CD3 on NKT ' on 'Hypertrophic cardiomyopathy'

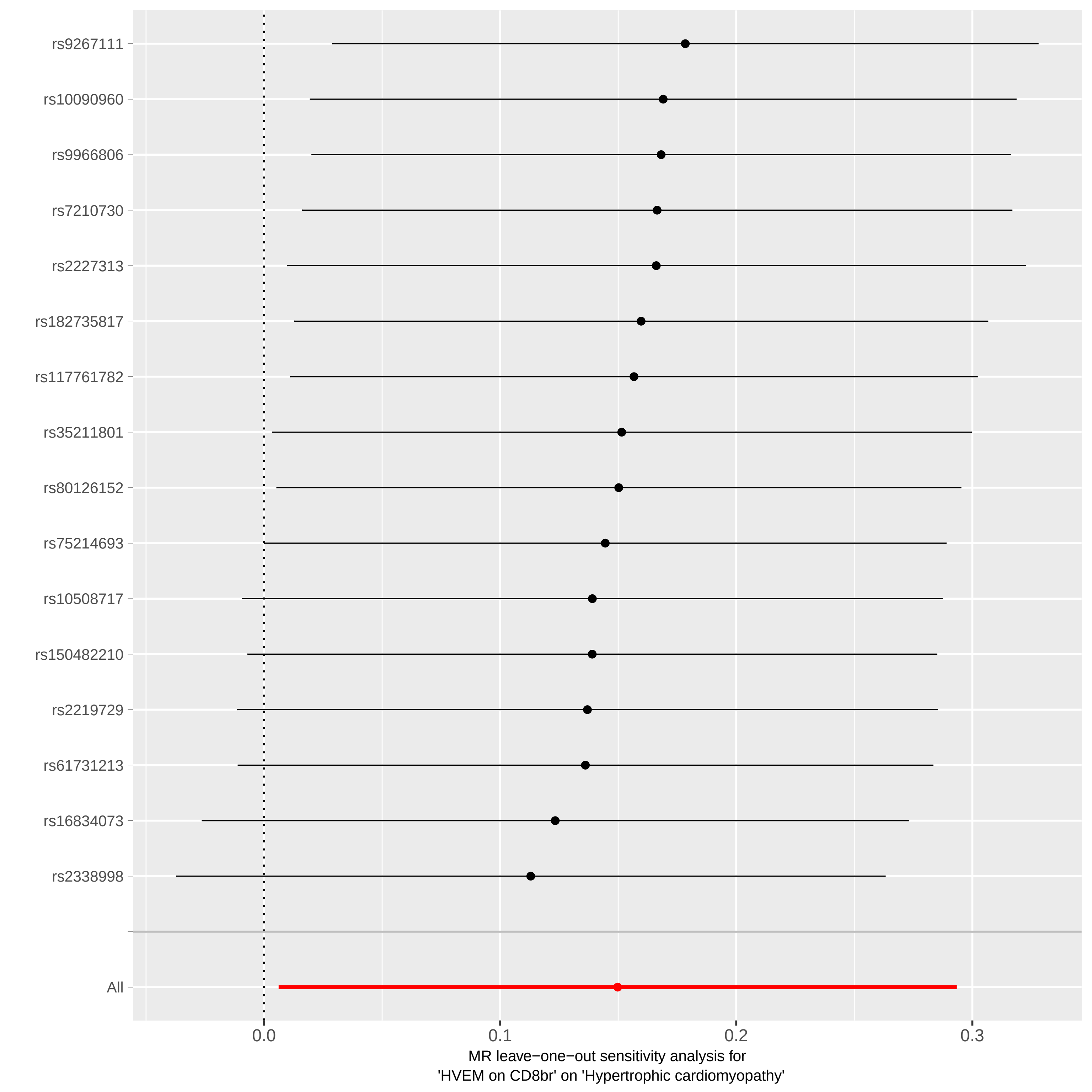
rs9267111
rs10090960
rs9966806
rs7210730
rs2227313
rs182735817
rs117761782
rs35211801
rs80126152
rs75214693
rs10508717
rs150482210
rs2219729
rs61731213
rs16834073
rs2338998
All
0.0
0.1
0.2
0.3
MR leave−one−out sensitivity analysis for
'HVEM on CD8br' on 'Hypertrophic cardiomyopathy'

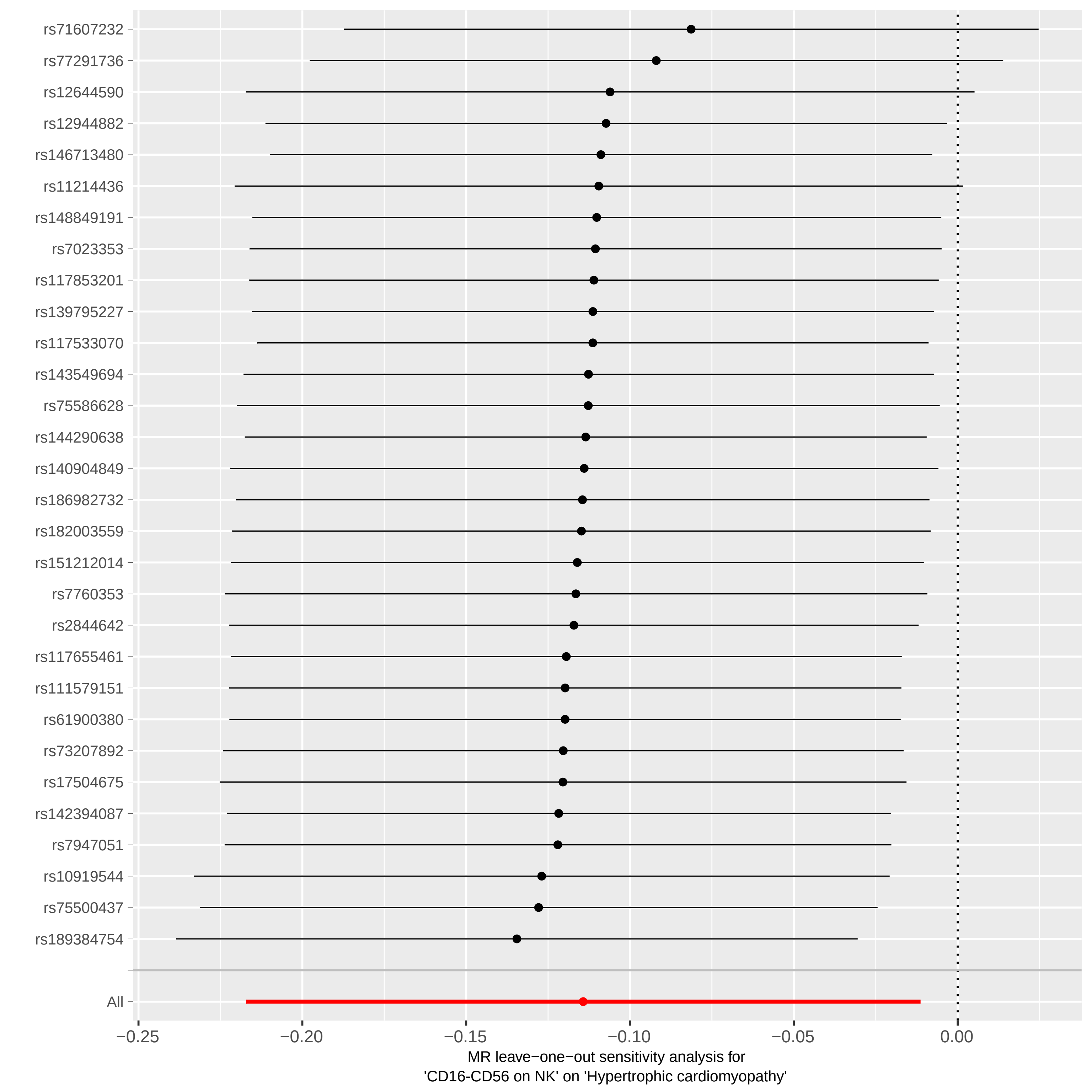
rs71607232
rs77291736
rs12644590
rs12944882
rs146713480
rs11214436
rs148849191
rs7023353
rs117853201
rs139795227
rs117533070
rs143549694
rs75586628
rs144290638
rs140904849
rs186982732
rs182003559
rs151212014
rs7760353
rs2844642
rs117655461
rs111579151
rs61900380
rs73207892
rs17504675
rs142394087
rs7947051
rs10919544
rs75500437
rs189384754
All
−0.25
−0.20
−0.15
−0.10
−0.05
0.00
MR leave−one−out sensitivity analysis for
'CD16-CD56 on NK' on 'Hypertrophic cardiomyopathy'

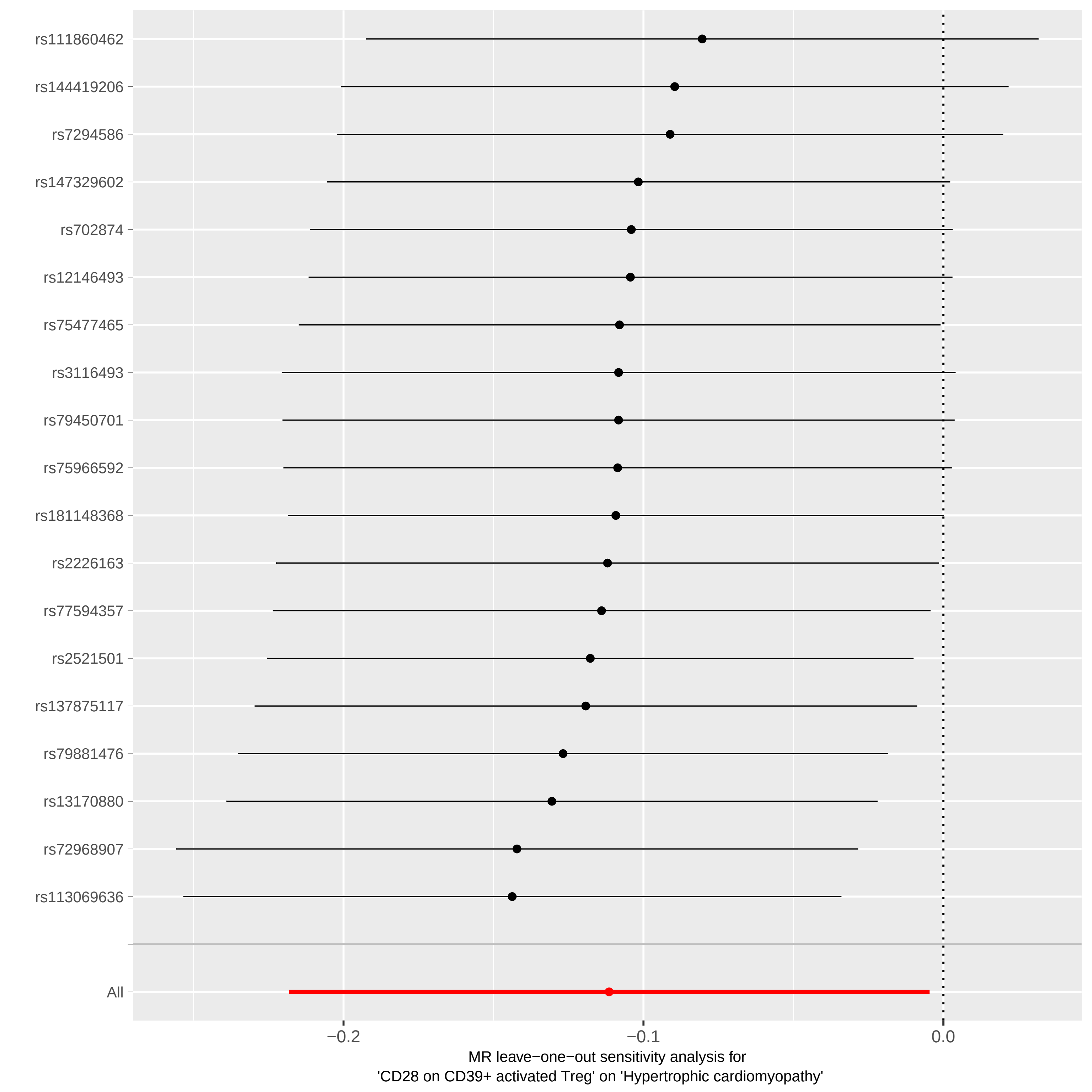

rs111860462
rs144419206
rs7294586
rs147329602
rs702874
rs12146493
rs75477465
rs3116493
rs79450701
rs75966592
rs181148368
rs2226163
rs77594357
rs2521501
rs137875117
rs79881476
rs13170880
rs72968907
rs113069636
All
−0.2
−0.1
0.0
MR leave−one−out sensitivity analysis for
'CD28 on CD39+ activated Treg' on 'Hypertrophic cardiomyopathy'

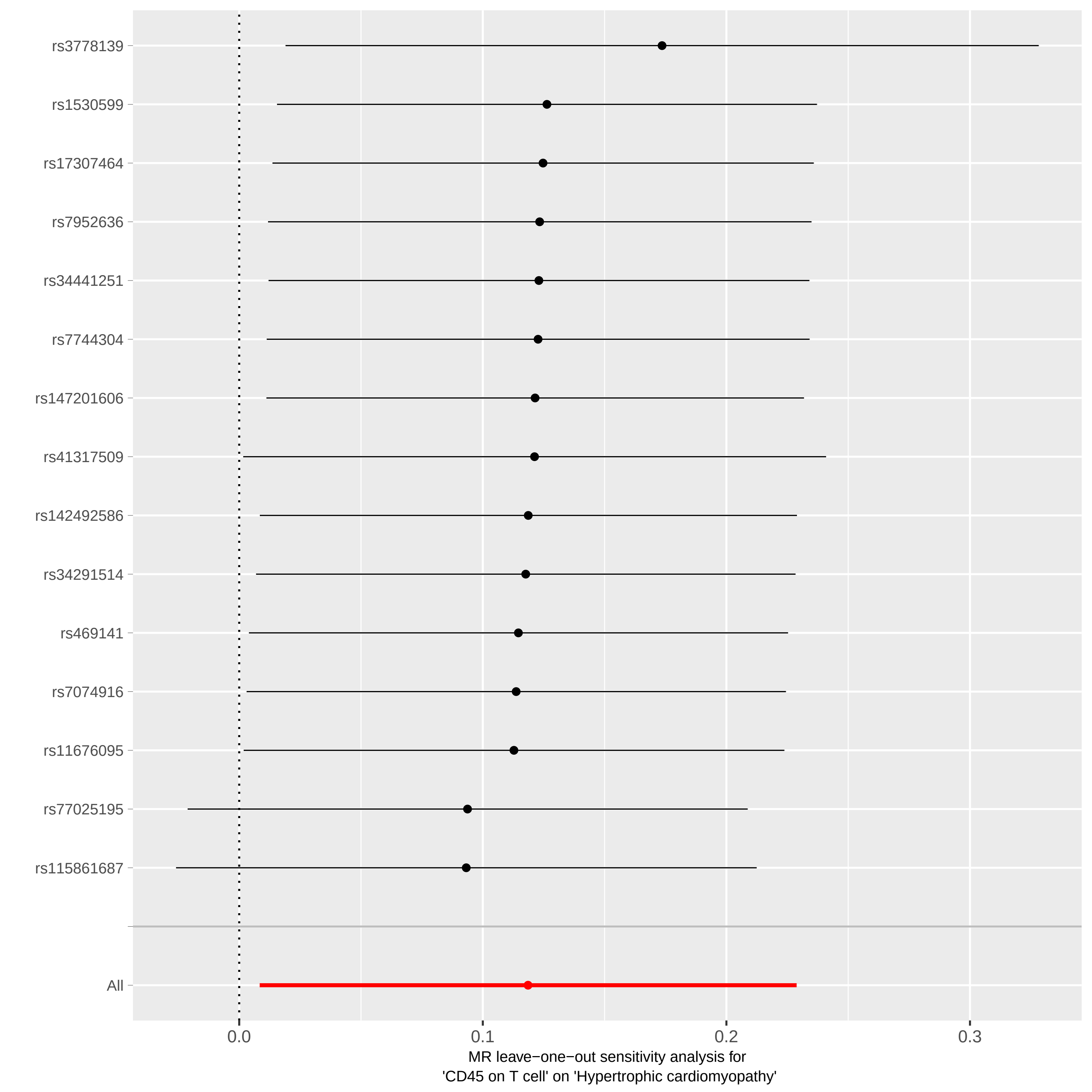
rs3778139
rs1530599
rs17307464
rs7952636
rs34441251
rs7744304
rs147201606
rs41317509
rs142492586
rs34291514
rs469141
rs7074916
rs11676095
rs77025195
rs115861687
All
0.0
0.1
0.2
0.3
MR leave−one−out sensitivity analysis for
'CD45 on T cell' on 'Hypertrophic cardiomyopathy'

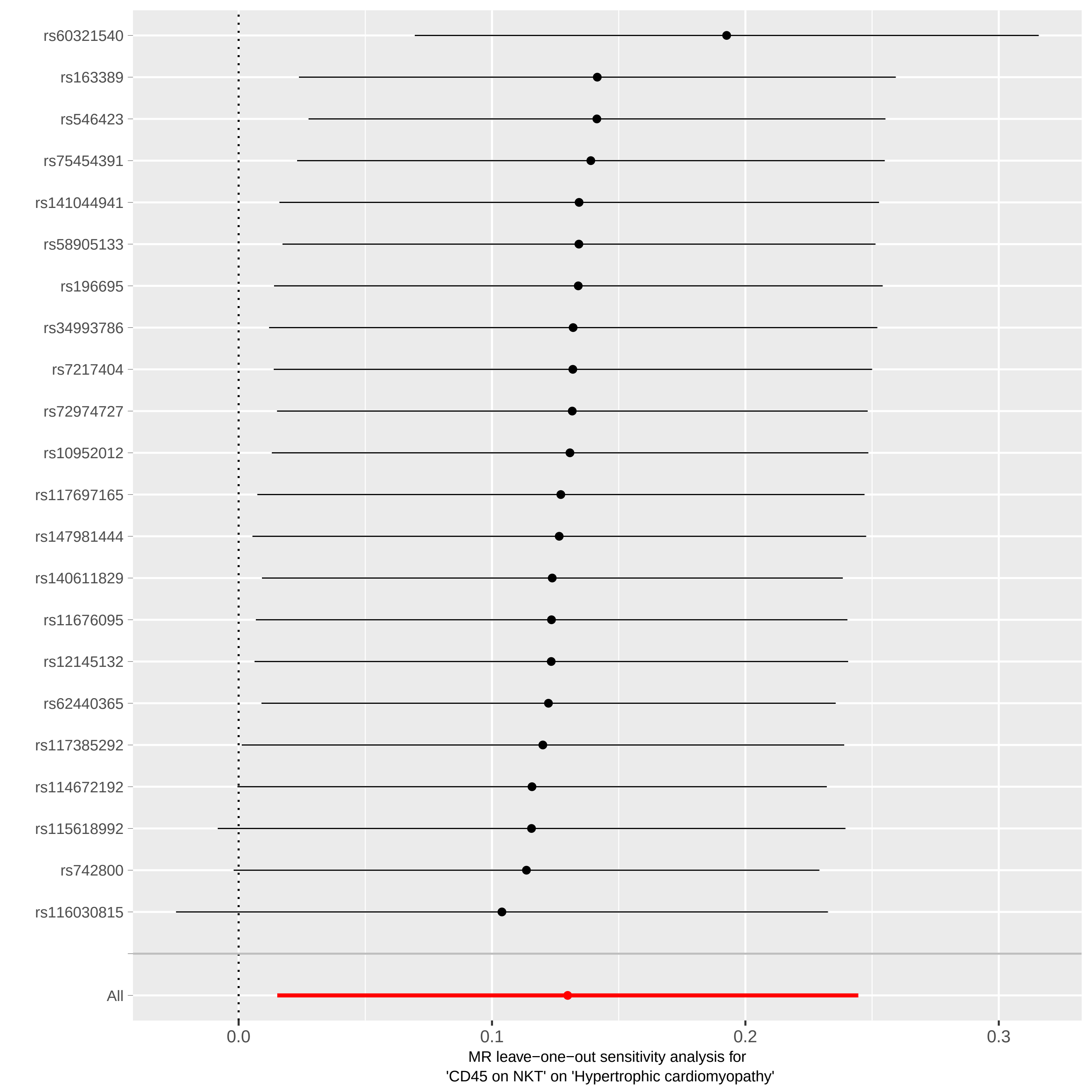
rs60321540
rs163389
rs546423
rs75454391
rs141044941
rs58905133
rs196695
rs34993786
rs7217404
rs72974727
rs10952012
rs117697165
rs147981444
rs140611829
rs11676095
rs12145132
rs62440365
rs117385292
rs114672192
rs115618992
rs742800
rs116030815
All
0.0
0.1
0.2
0.3
MR leave−one−out sensitivity analysis for
'CD45 on NKT' on 'Hypertrophic cardiomyopathy'

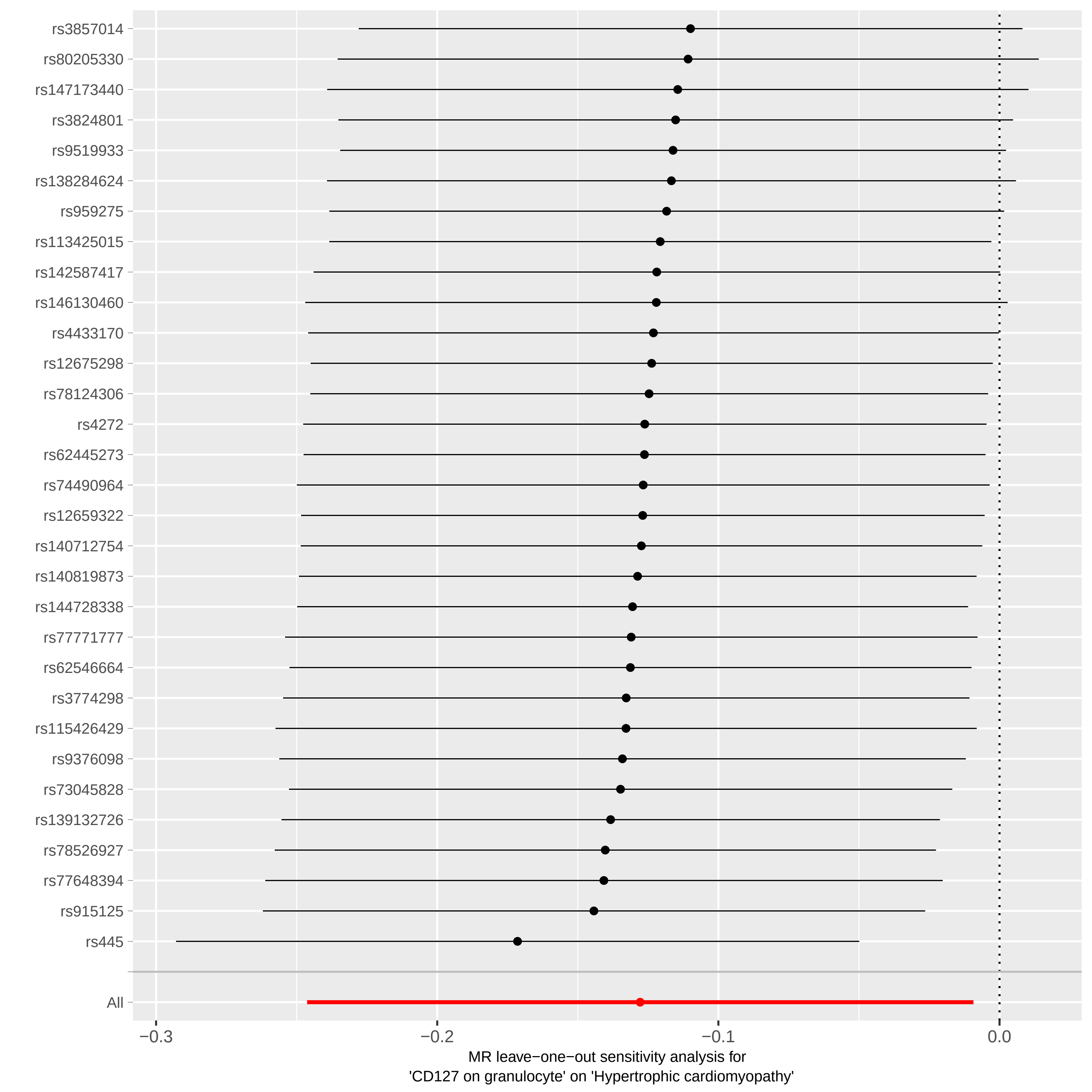

rs3857014
rs80205330
rs147173440
rs3824801
rs9519933
rs138284624
rs959275
rs113425015
rs142587417
rs146130460
rs4433170
rs12675298
rs78124306
rs4272
rs62445273
rs74490964
rs12659322
rs140712754
rs140819873
rs144728338
rs77771777
rs62546664
rs3774298
rs115426429
rs9376098
rs73045828
rs139132726
rs78526927
rs77648394
rs915125
rs445
All
−0.3
−0.2
−0.1
0.0
MR leave−one−out sensitivity analysis for
'CD127 on granulocyte' on 'Hypertrophic cardiomyopathy'

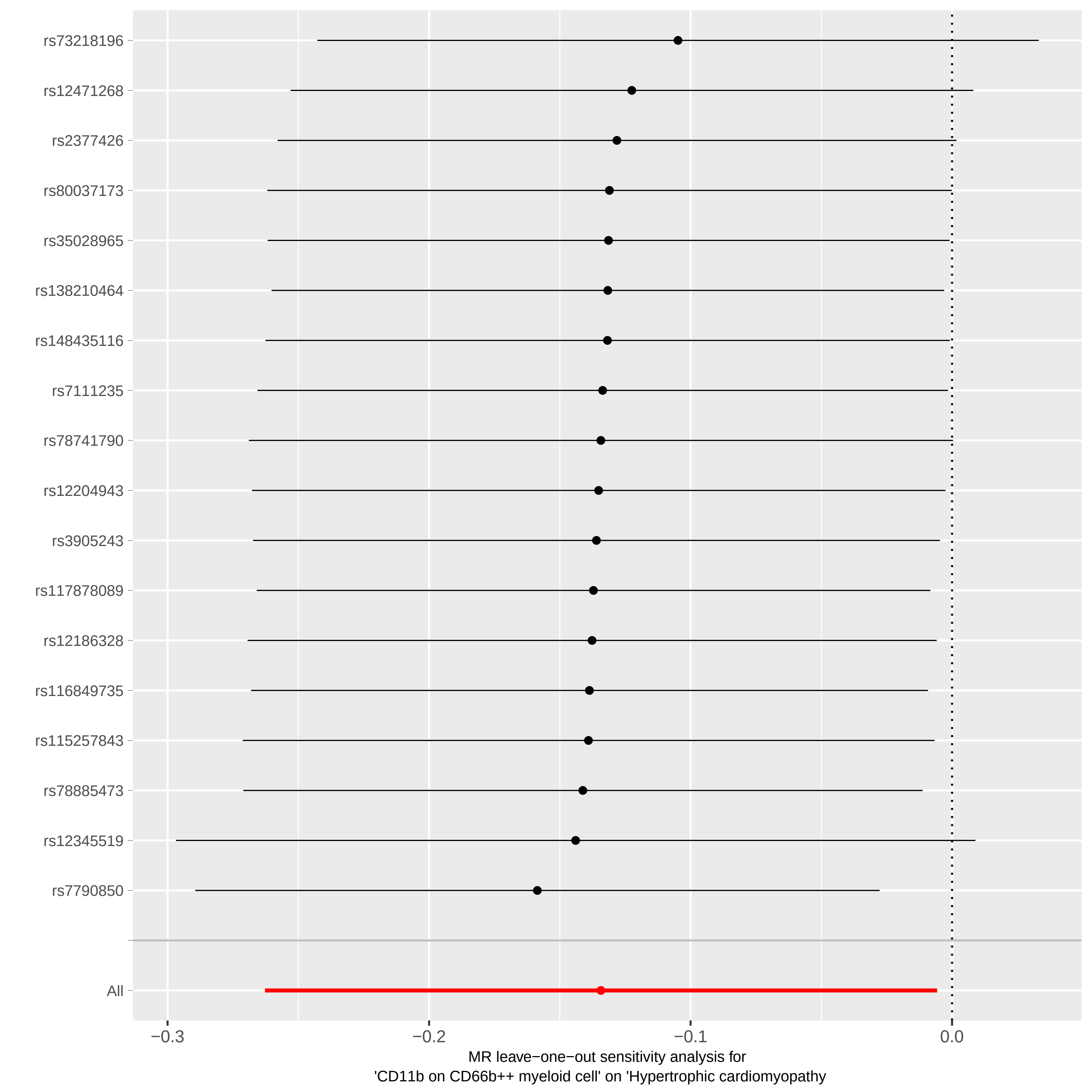

rs73218196
rs12471268
rs2377426
rs80037173
rs35028965
rs138210464
rs148435116
rs7111235
rs78741790
rs12204943
rs3905243
rs117878089
rs12186328
rs116849735
rs115257843
rs78885473
rs12345519
rs7790850
All
−0.3
−0.2
−0.1
0.0
MR leave−one−out sensitivity analysis for
'CD11b on CD66b++ myeloid cell' on 'Hypertrophic cardiomyopathy

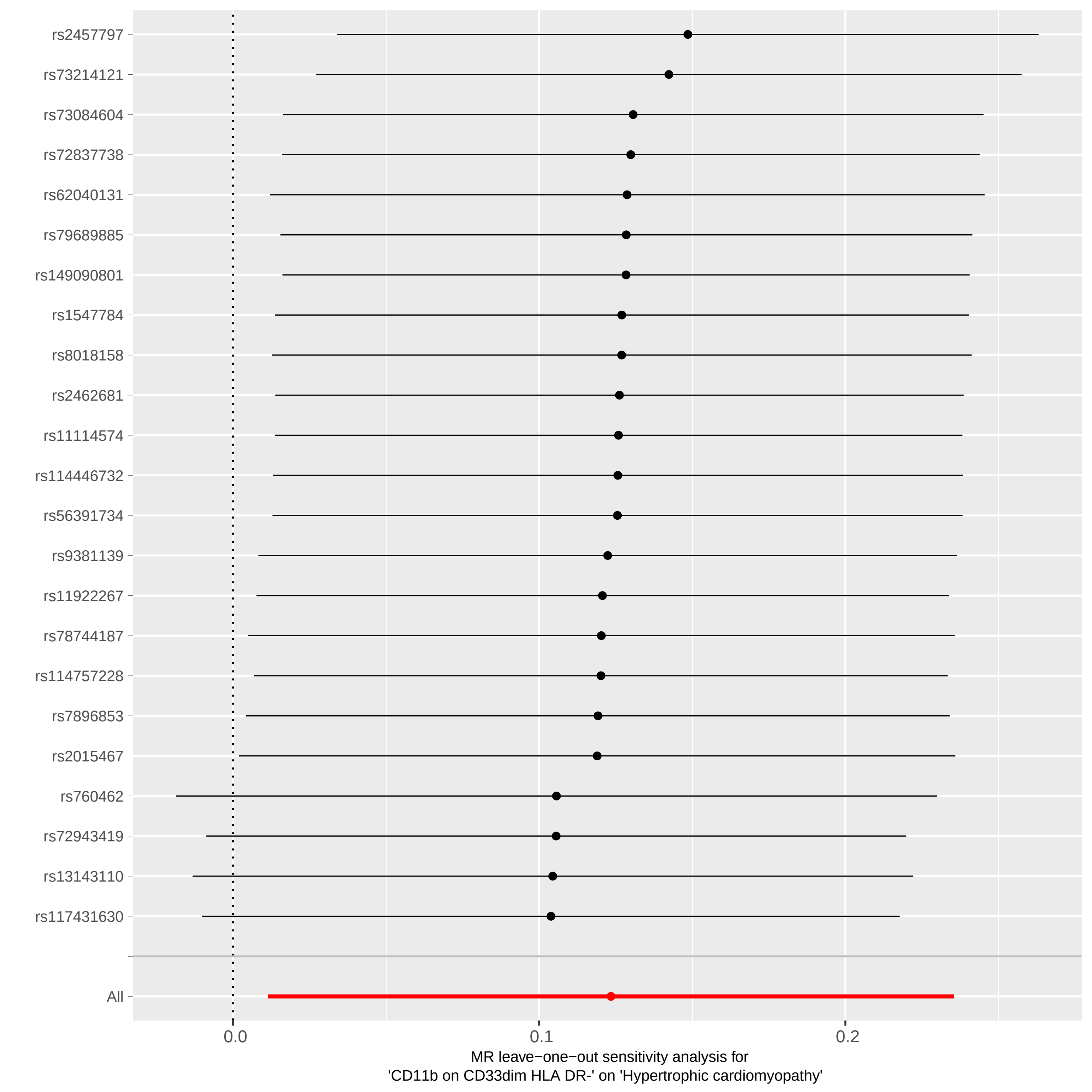

rs2457797
rs73214121
rs73084604
rs72837738
rs62040131
rs79689885
rs149090801
rs1547784
rs8018158
rs2462681
rs11114574
rs114446732
rs56391734
rs9381139
rs11922267
rs78744187
rs114757228
rs7896853
rs2015467
rs760462
rs72943419
rs13143110
rs117431630
All
0.0
0.1
0.2
MR leave−one−out sensitivity analysis for
'CD11b on CD33dim HLA DR-' on 'Hypertrophic cardiomyopathy'

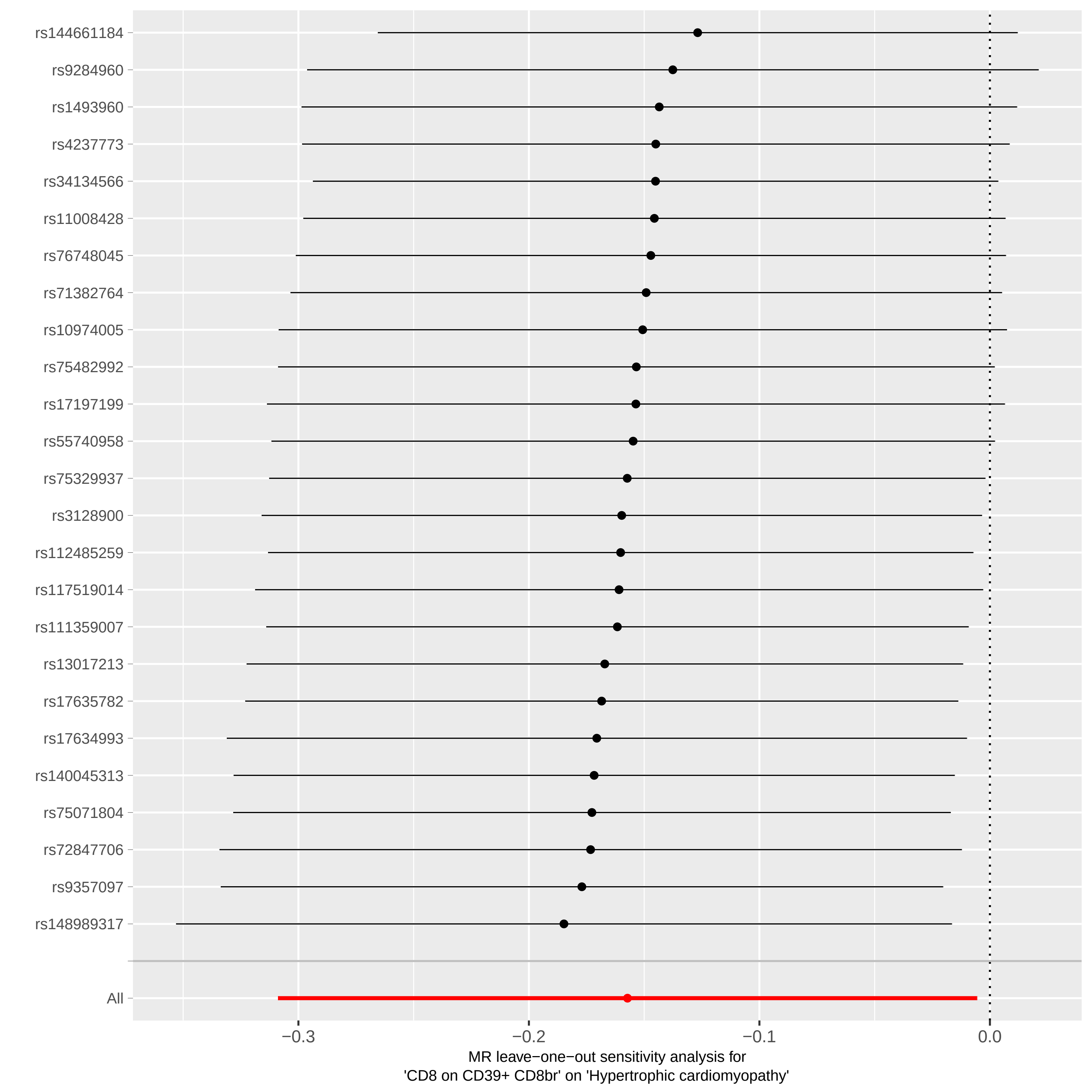
rs144661184
rs9284960
rs1493960
rs4237773
rs34134566
rs11008428
rs76748045
rs71382764
rs10974005
rs75482992
rs17197199
rs55740958
rs75329937
rs3128900
rs112485259
rs117519014
rs111359007
rs13017213
rs17635782
rs17634993
rs140045313
rs75071804
rs72847706
rs9357097
rs148989317
All
−0.3
−0.2
−0.1
0.0
MR leave−one−out sensitivity analysis for
'CD8 on CD39+ CD8br' on 'Hypertrophic cardiomyopathy'

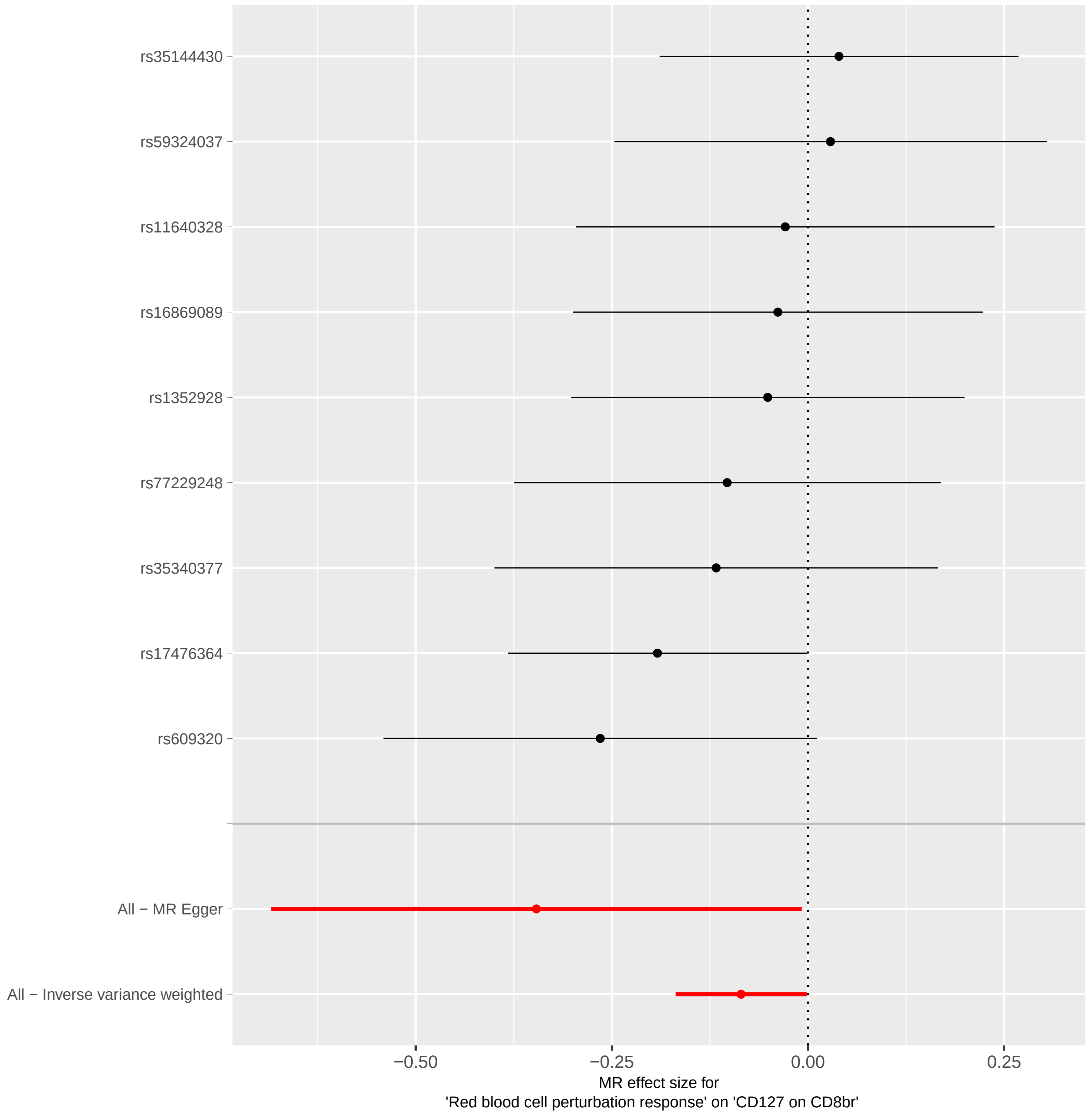
rs35144430
rs59324037
rs11640328
rs16869089
rs1352928
rs77229248
rs35340377
rs17476364
rs609320
All − MR Egger
All − Inverse variance weighted
−0.50
−0.25
0.00
0.25
MR effect size for
'Red blood cell perturbation response' on 'CD127 on CD8br'

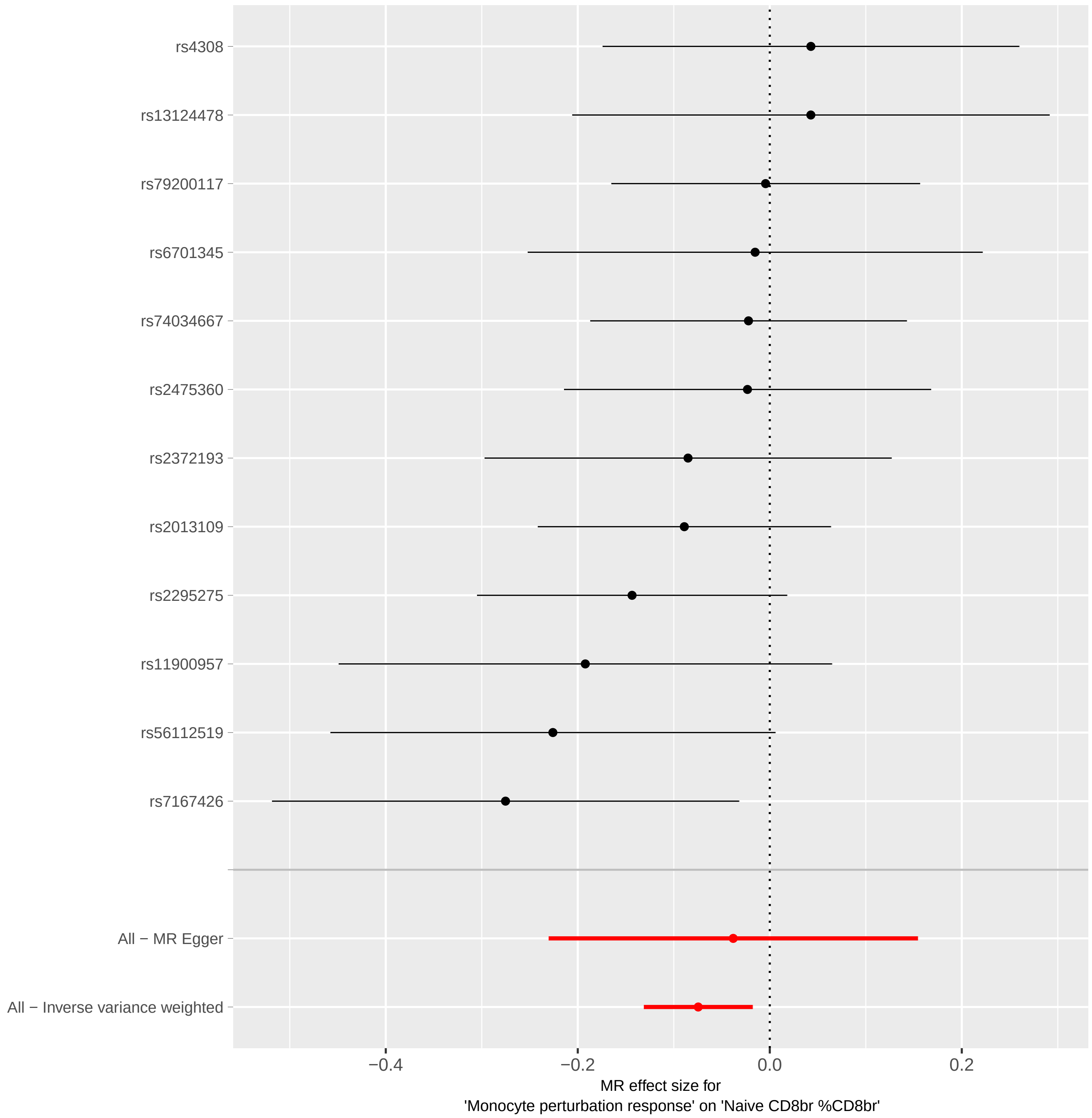

rs4308
rs13124478
rs79200117
rs6701345
rs74034667
rs2475360
rs2372193
rs2013109
rs2295275
rs11900957
rs56112519
rs7167426
All − MR Egger
All − Inverse variance weighted
−0.4
−0.2
0.0
0.2
MR effect size for
'Monocyte perturbation response' on 'Naive CD8br %CD8br'

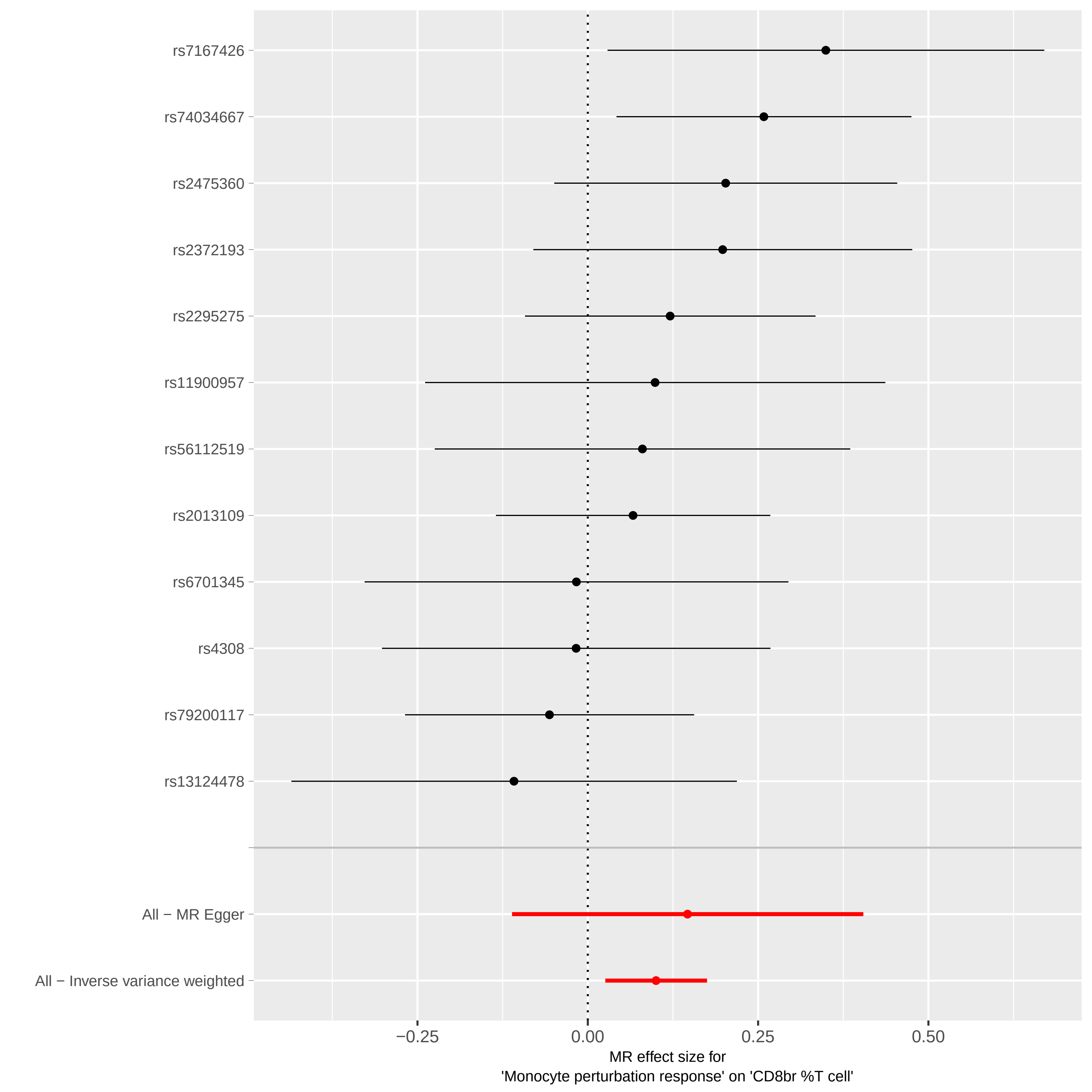
rs7167426
rs74034667
rs2475360
rs2372193
rs2295275
rs11900957
rs56112519
rs2013109
rs6701345
rs4308
rs79200117
rs13124478
All − MR Egger
All − Inverse variance weighted
−0.25
0.00
0.25
0.50
MR effect size for
'Monocyte perturbation response' on 'CD8br %T cell'

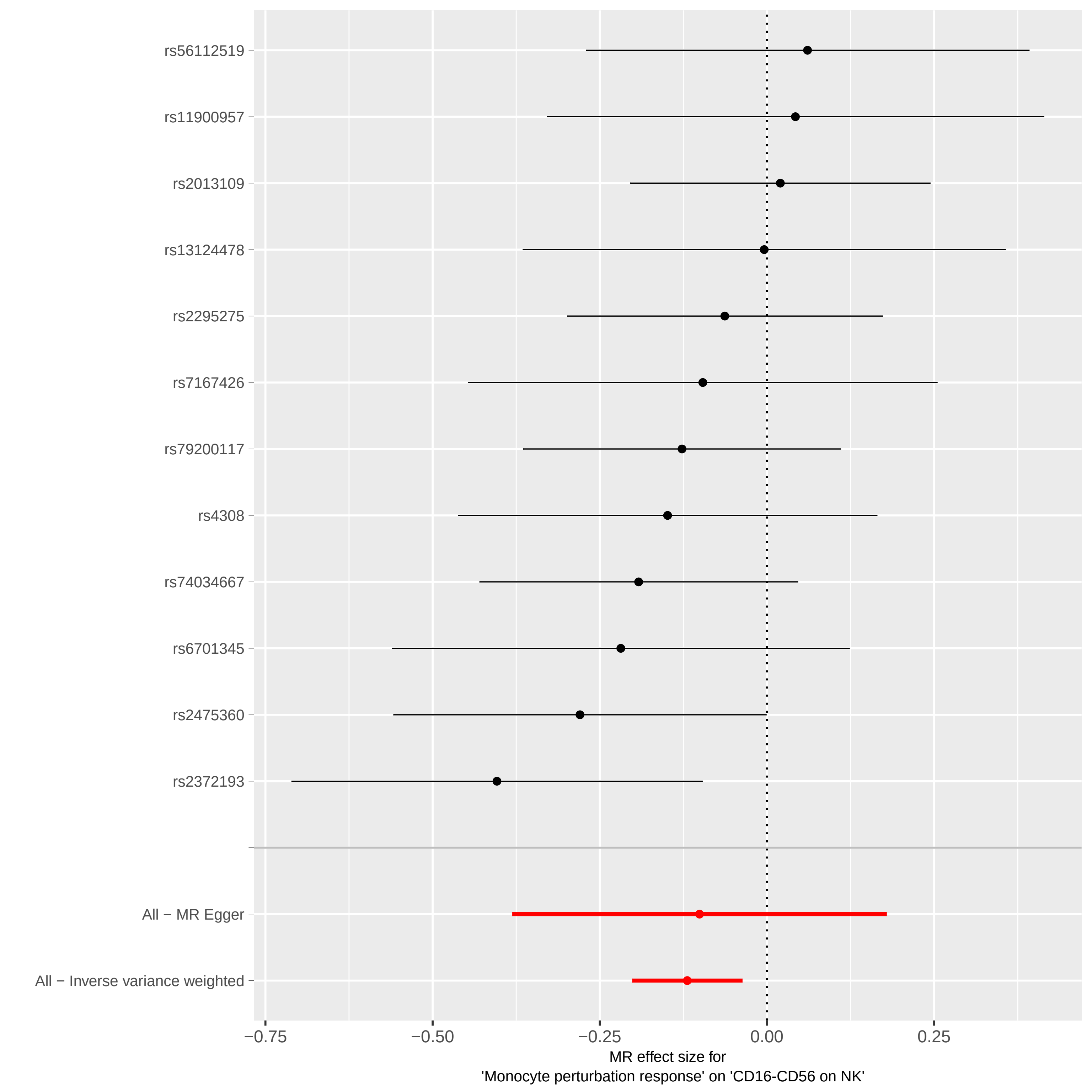
rs56112519
rs11900957
rs2013109
rs13124478
rs2295275
rs7167426
rs79200117
rs4308
rs74034667
rs6701345
rs2475360
rs2372193
All − MR Egger
All − Inverse variance weighted
−0.75
−0.50
−0.25
0.00
0.25
MR effect size for
'Monocyte perturbation response' on 'CD16-CD56 on NK'

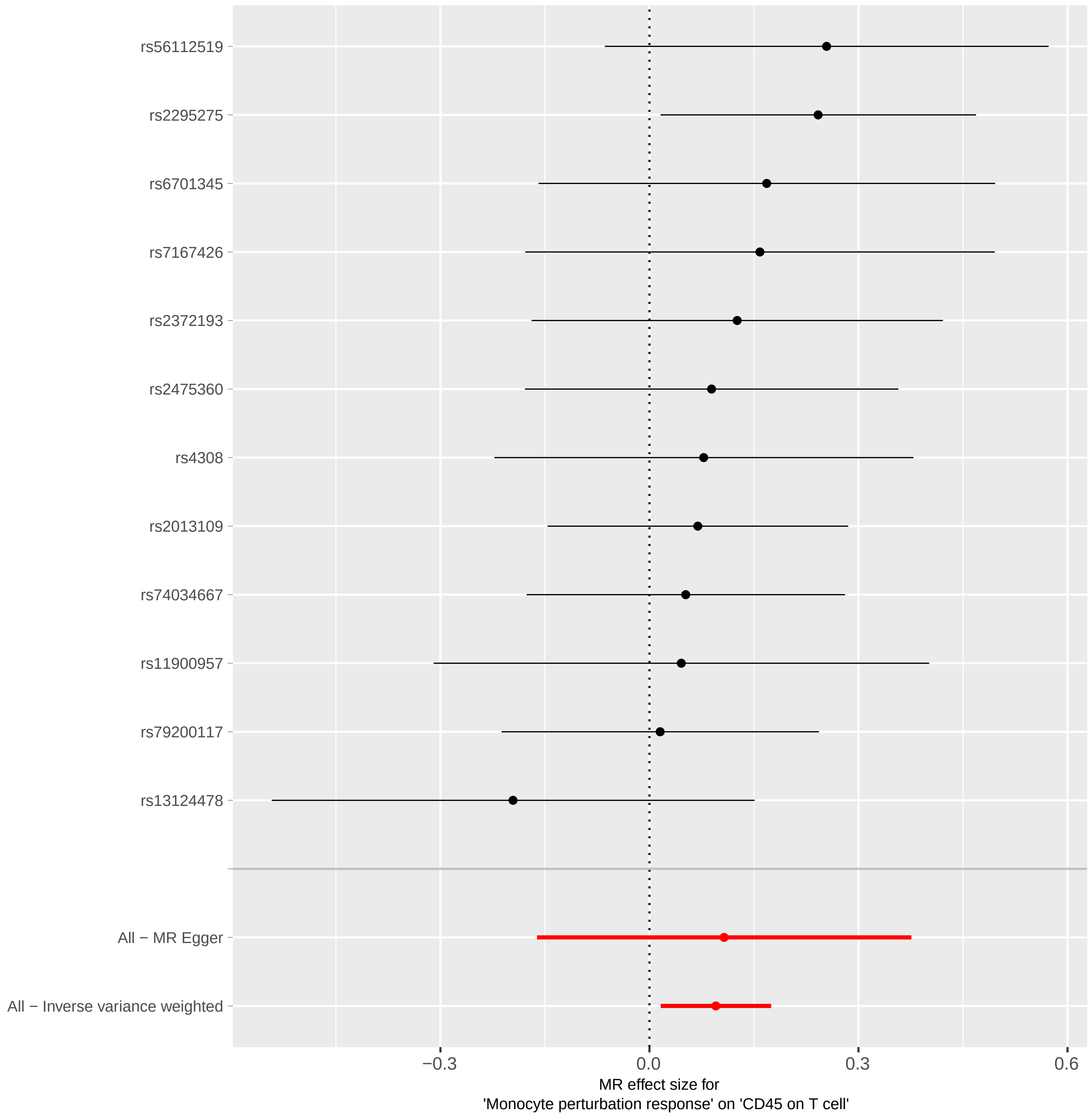

rs56112519
rs2295275
rs6701345
rs7167426
rs2372193
rs2475360
rs4308
rs2013109
rs74034667
rs11900957
rs79200117
rs13124478
All − MR Egger
All − Inverse variance weighted
−0.3
0.0
0.3
0.6
MR effect size for
'Monocyte perturbation response' on 'CD45 on T cell'

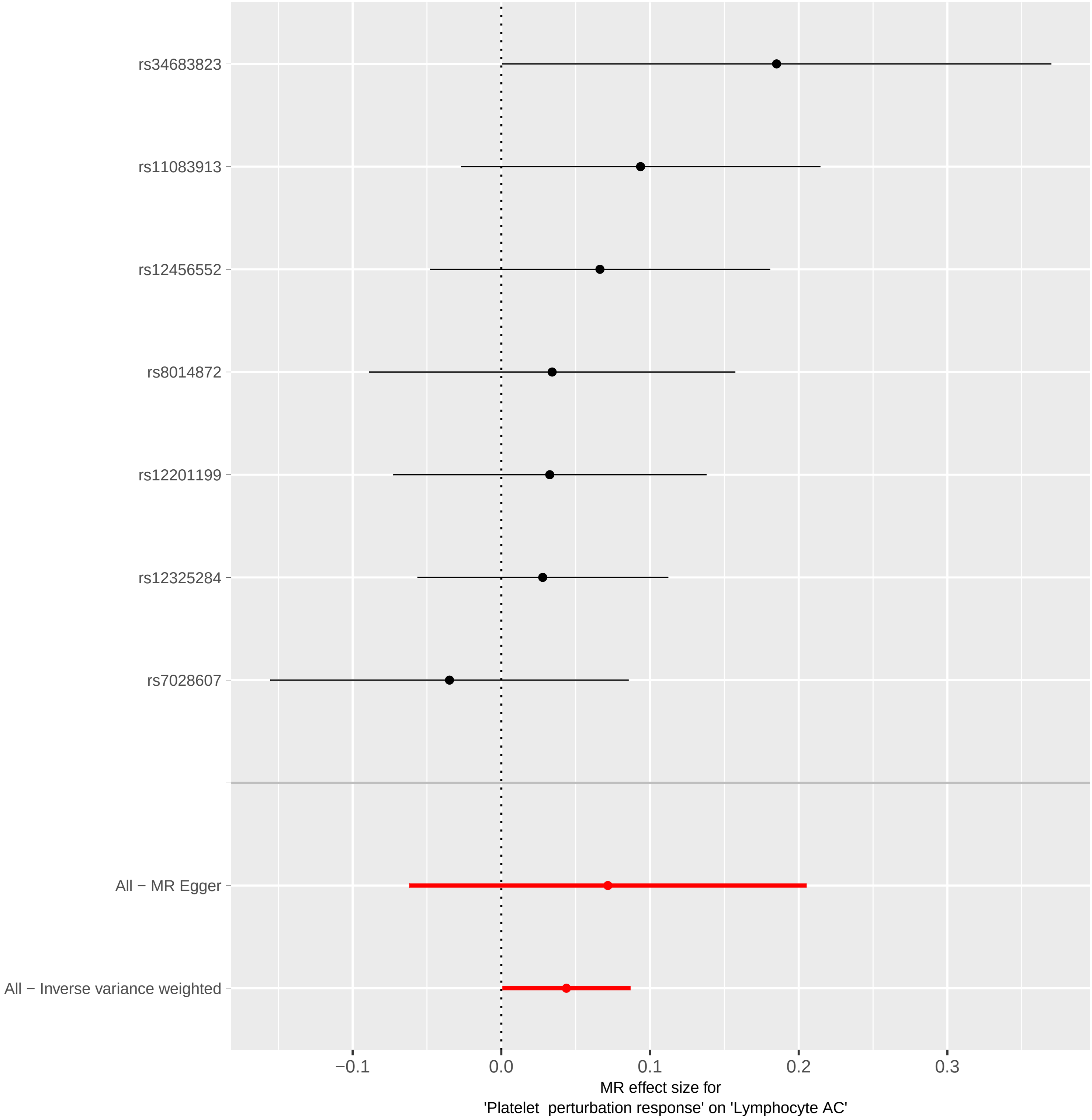

rs34683823
rs11083913
rs12456552
rs8014872
rs12201199
rs12325284
rs7028607
All − MR Egger
All − Inverse variance weighted
−0.1
0.0
0.1
0.2
0.3
MR effect size for
'Platelet perturbation response' on 'Lymphocyte AC'

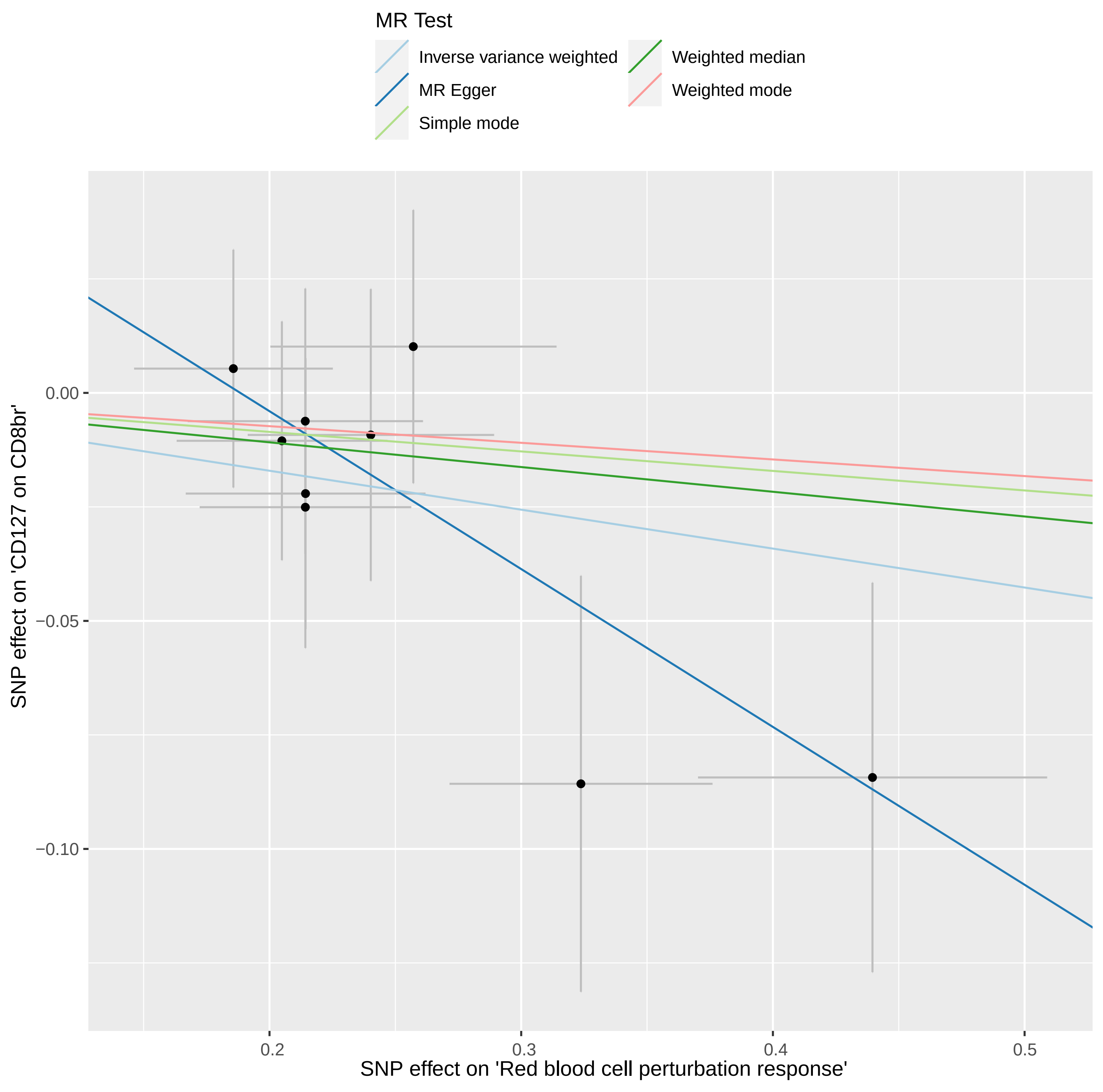

MR Test
Inverse variance weighted
MR Egger
Simple mode
Weighted median
Weighted mode
0.00
−0.05
−0.10
SNP effect on 'CD127 on CD8br'
0.2
0.3
0.4
0.5
SNP effect on 'Red blood cell perturbation response'

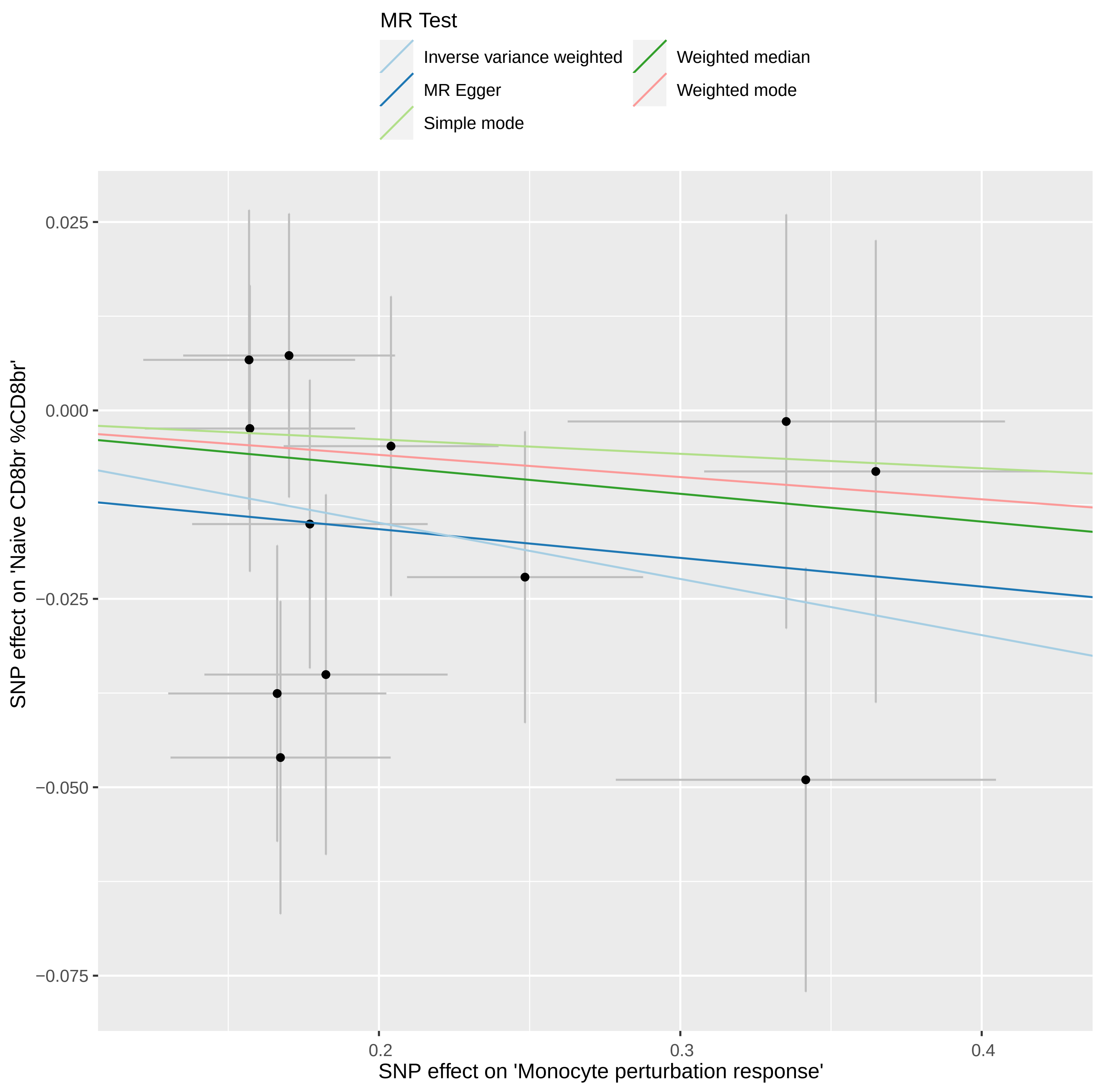

MR Test
Inverse variance weighted
MR Egger
Simple mode
Weighted median
Weighted mode
0.025
0.000
−0.025
−0.050
−0.075
0.2
0.3
0.4
SNP effect on 'Naive CD8br %CD8br'
SNP effect on 'Monocyte perturbation response'

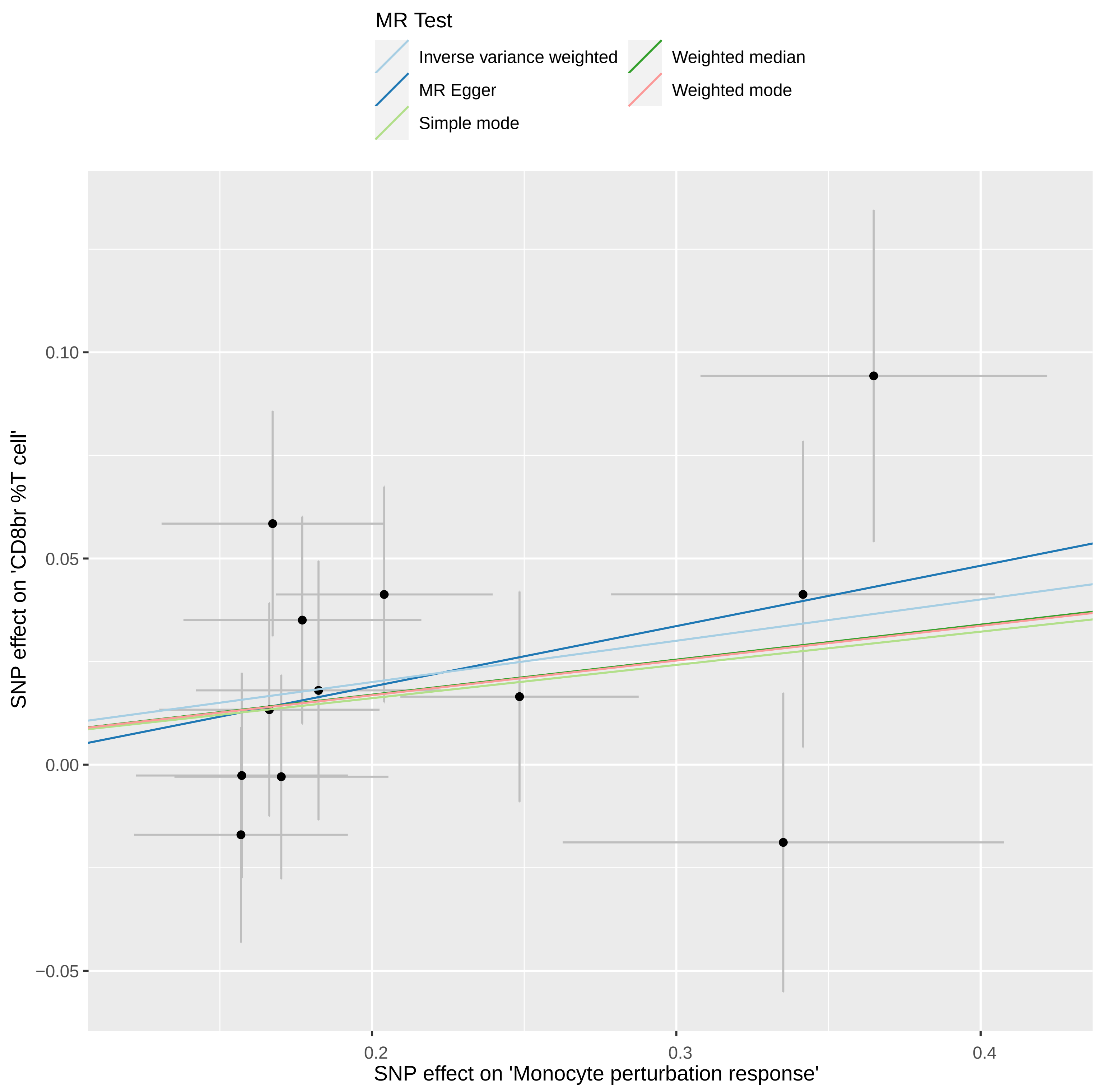

MR Test
Inverse variance weighted
MR Egger
Simple mode
Weighted median
Weighted mode
0.10
0.05
0.00
−0.05
0.2
0.3
0.4
SNP effect on 'CD8br %T cell'
SNP effect on 'Monocyte perturbation response'

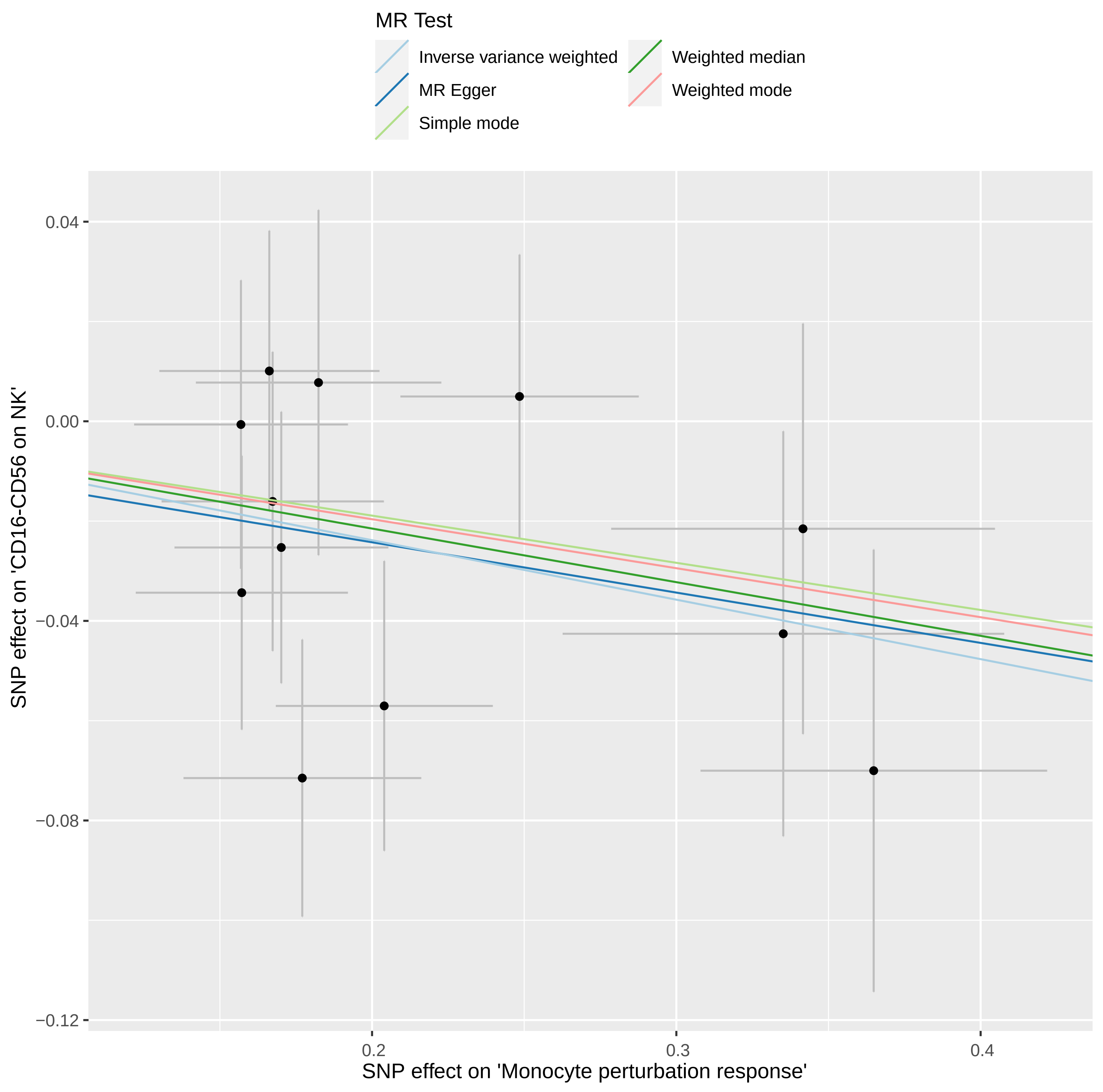

MR Test
Inverse variance weighted
MR Egger
Simple mode
Weighted median
Weighted mode
0.04
0.00
−0.04
−0.08
−0.12
0.2
0.3
0.4
SNP effect on 'CD16-CD56 on NK'
SNP effect on 'Monocyte perturbation response'

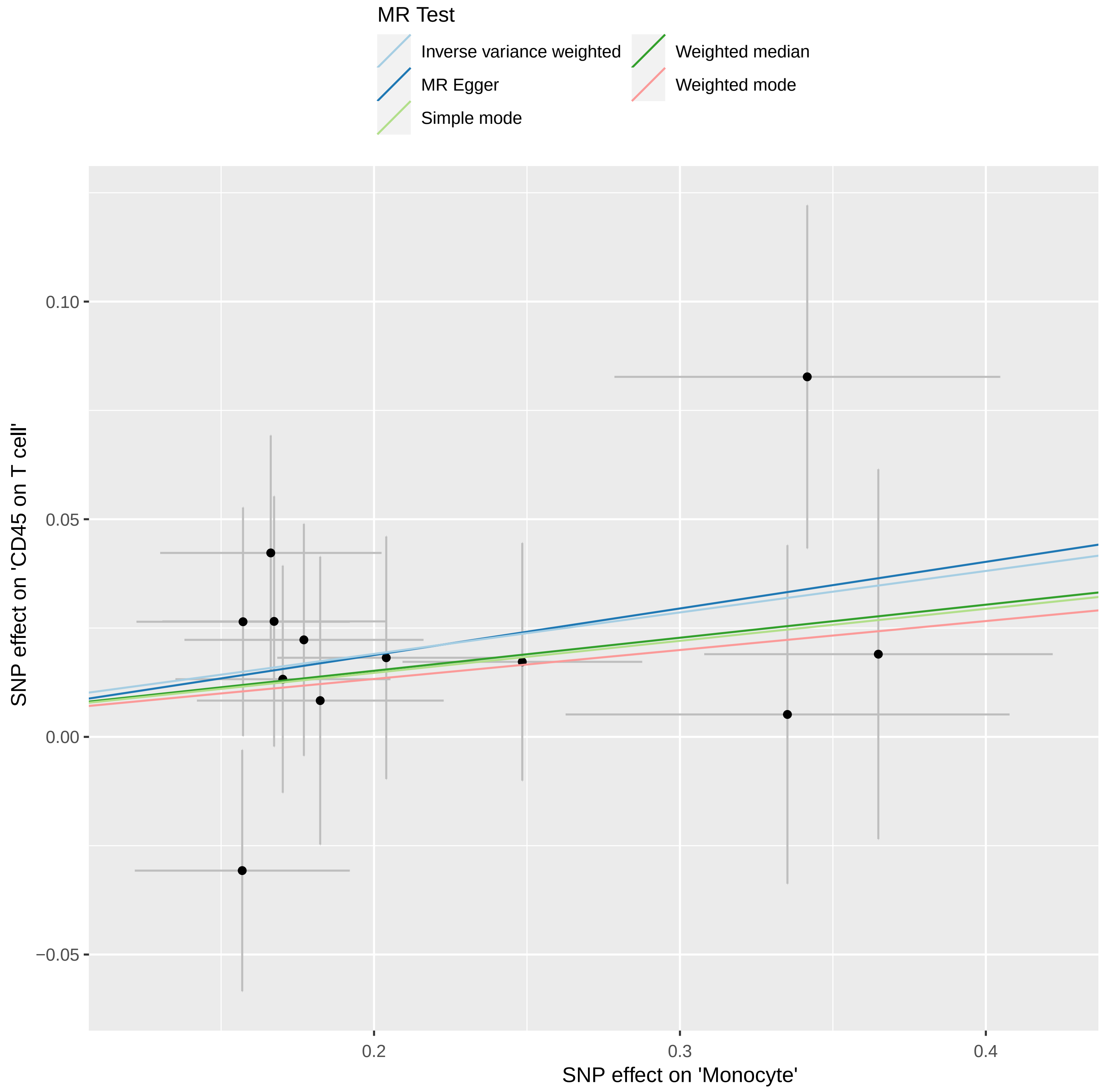

MR Test
Inverse variance weighted
MR Egger
Simple mode
Weighted median
Weighted mode
0.10
0.05
0.00
−0.05
0.2
0.3
0.4
SNP effect on 'CD45 on T cell'
SNP effect on 'Monocyte'

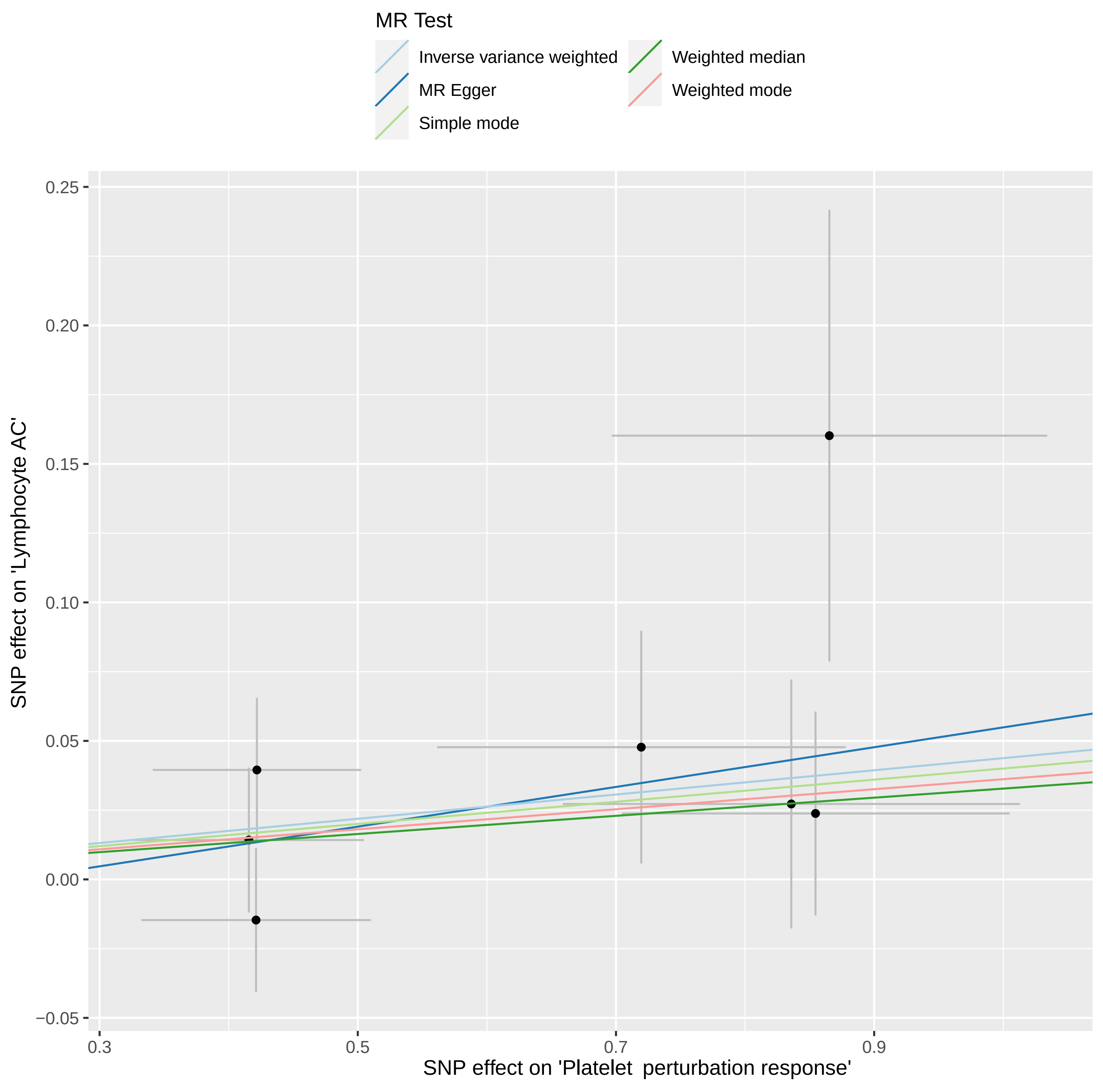

MR Test
Inverse variance weighted
MR Egger
Simple mode
Weighted median
Weighted mode
0.25
0.20
0.15
0.10
0.05
0.00
−0.05
0.3
0.5
0.7
0.9
SNP effect on 'Lymphocyte AC'
SNP effect on 'Platelet perturbation response'

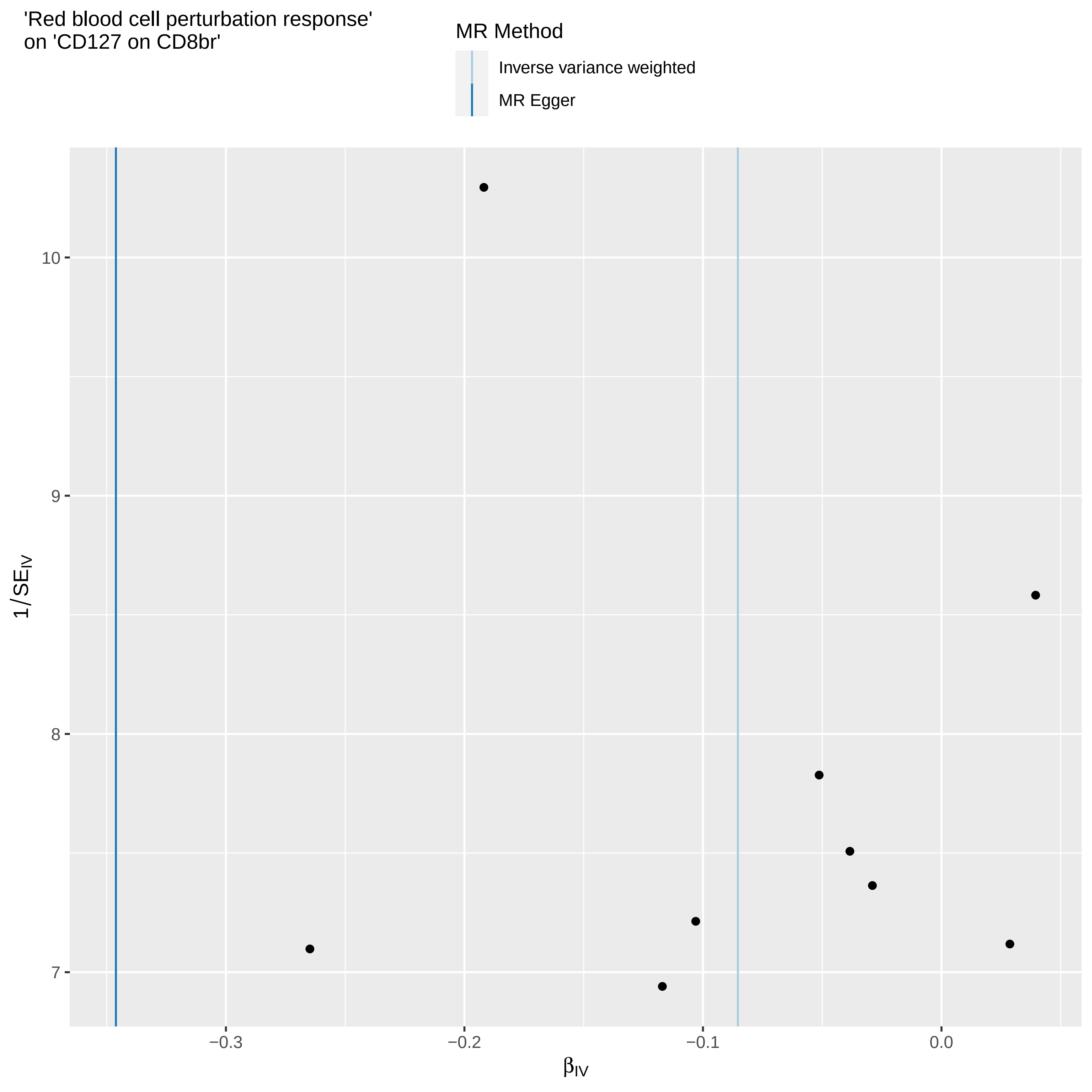
'Red blood cell perturbation response'
on 'CD127 on CD8br'
MR Method
Inverse variance weighted
MR Egger
10
9
8
7
1/SE$_{IV}$
−0.3
−0.2
−0.1
0.0
β$_{IV}$

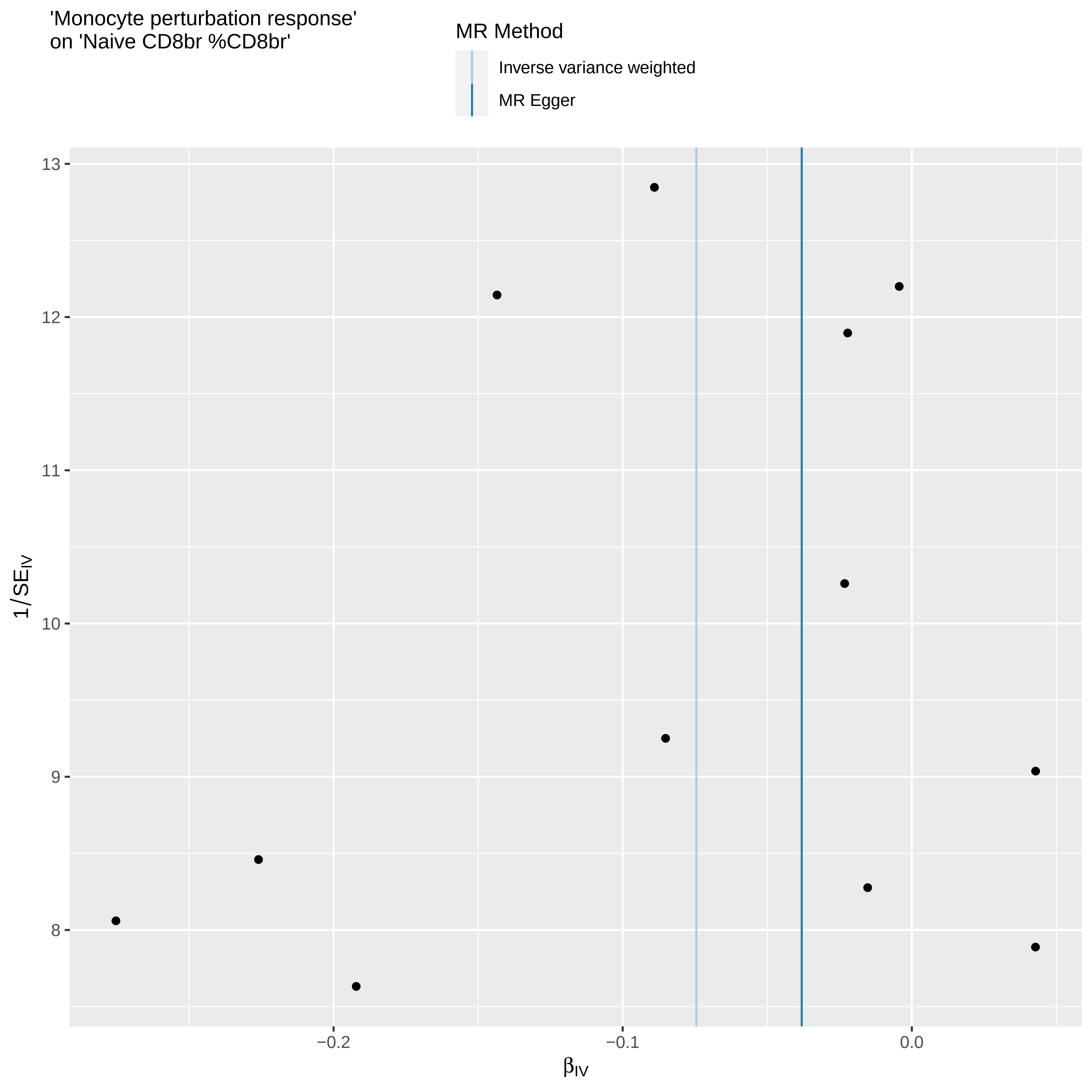
'Monocyte perturbation response'
on 'Naive CD8br %CD8br'
MR Method
Inverse variance weighted
MR Egger
13
12
11
10
9
8
1/SE_IV
−0.2
−0.1
0.0
β_IV

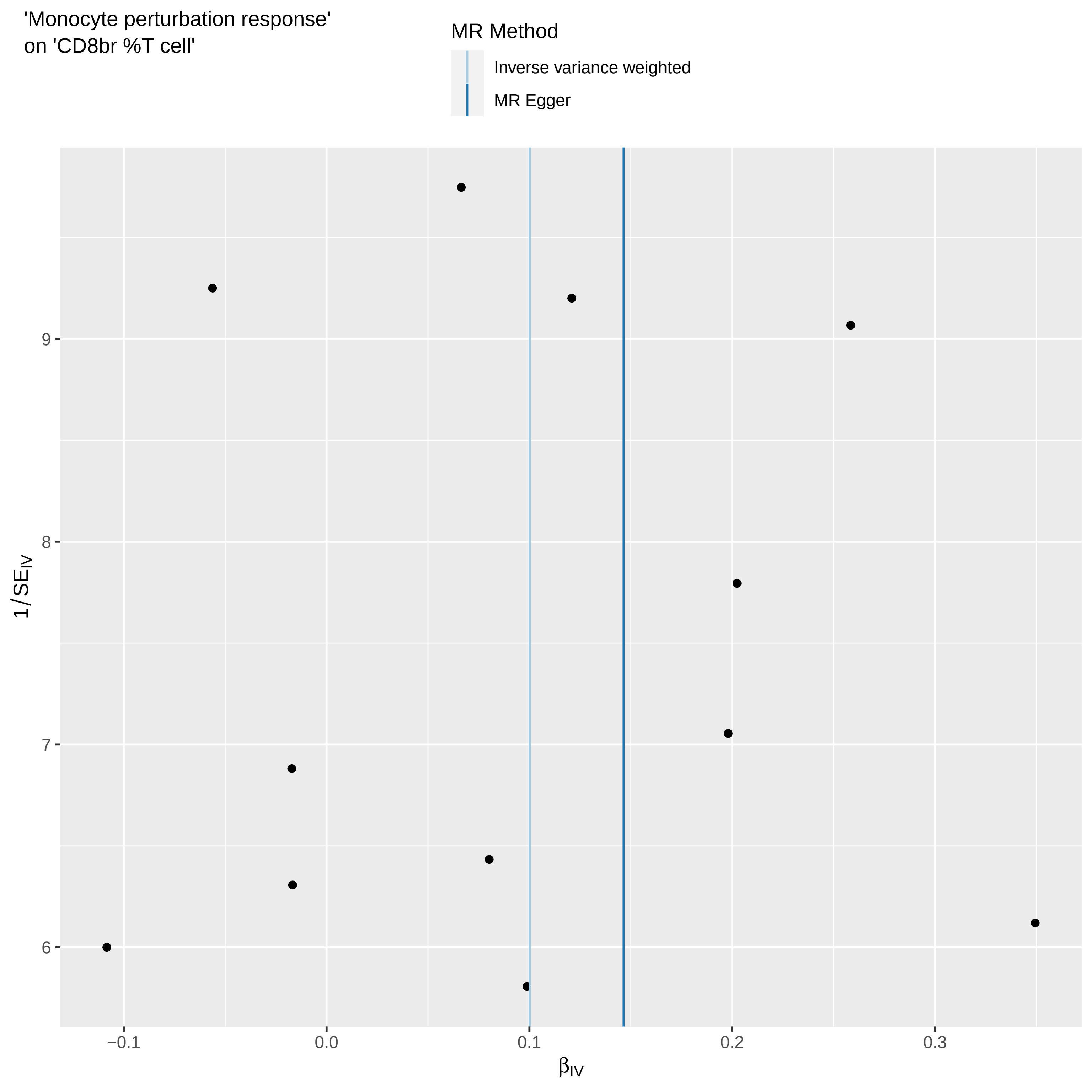
'Monocyte perturbation response'
on 'CD8br %T cell'
MR Method
Inverse variance weighted
MR Egger
9
8
7
6
1/SE_IV
−0.1
0.0
0.1
0.2
0.3
β_IV

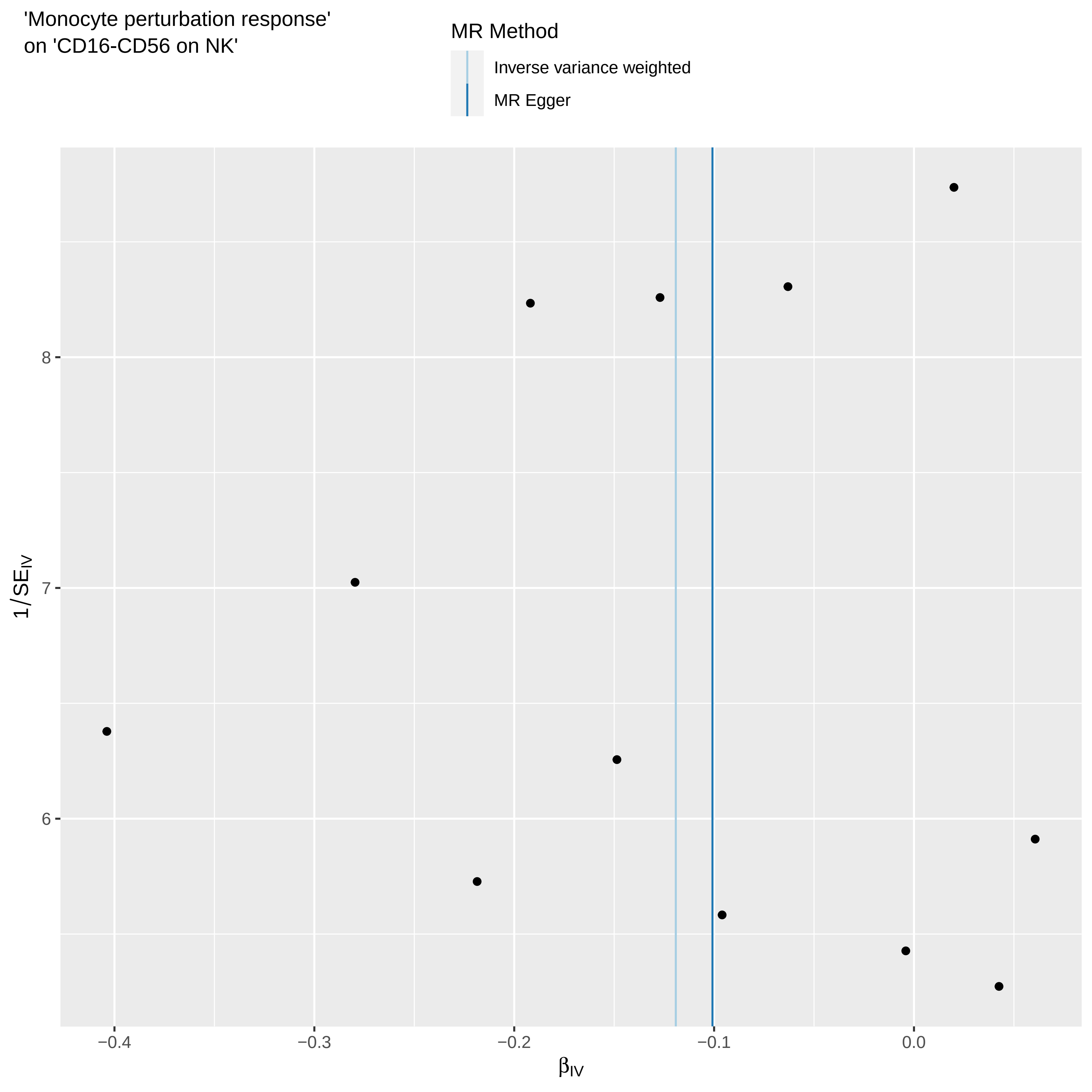

'Monocyte perturbation response'
on 'CD16-CD56 on NK'
MR Method
Inverse variance weighted
MR Egger
8
7
6
1/SE_IV
−0.4
−0.3
−0.2
−0.1
0.0
β_IV

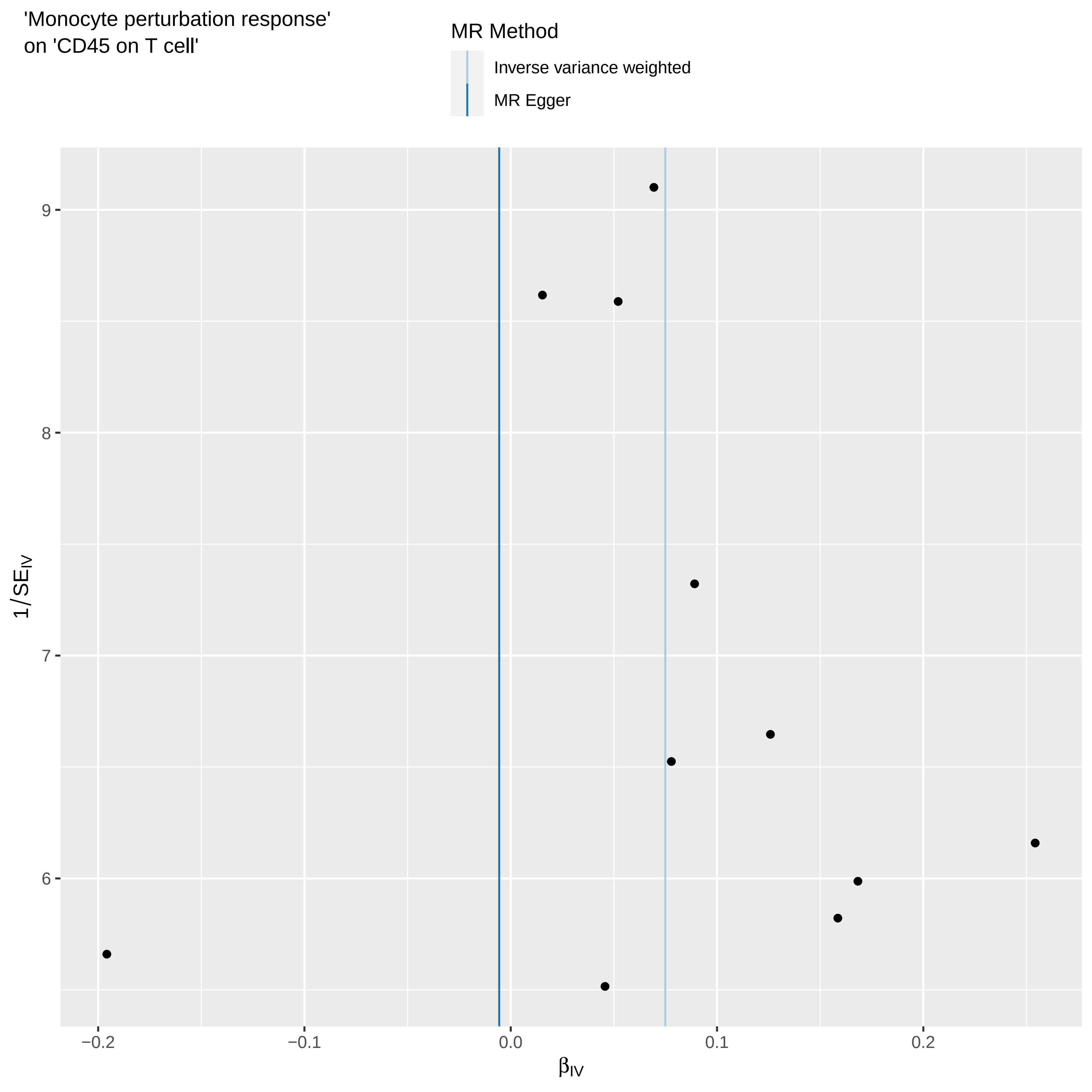

'Monocyte perturbation response'
on 'CD45 on T cell'
MR Method
Inverse variance weighted
MR Egger
9
8
7
6
1/SE$_{IV}$
−0.2
−0.1
0.0
0.1
0.2
β$_{IV}$

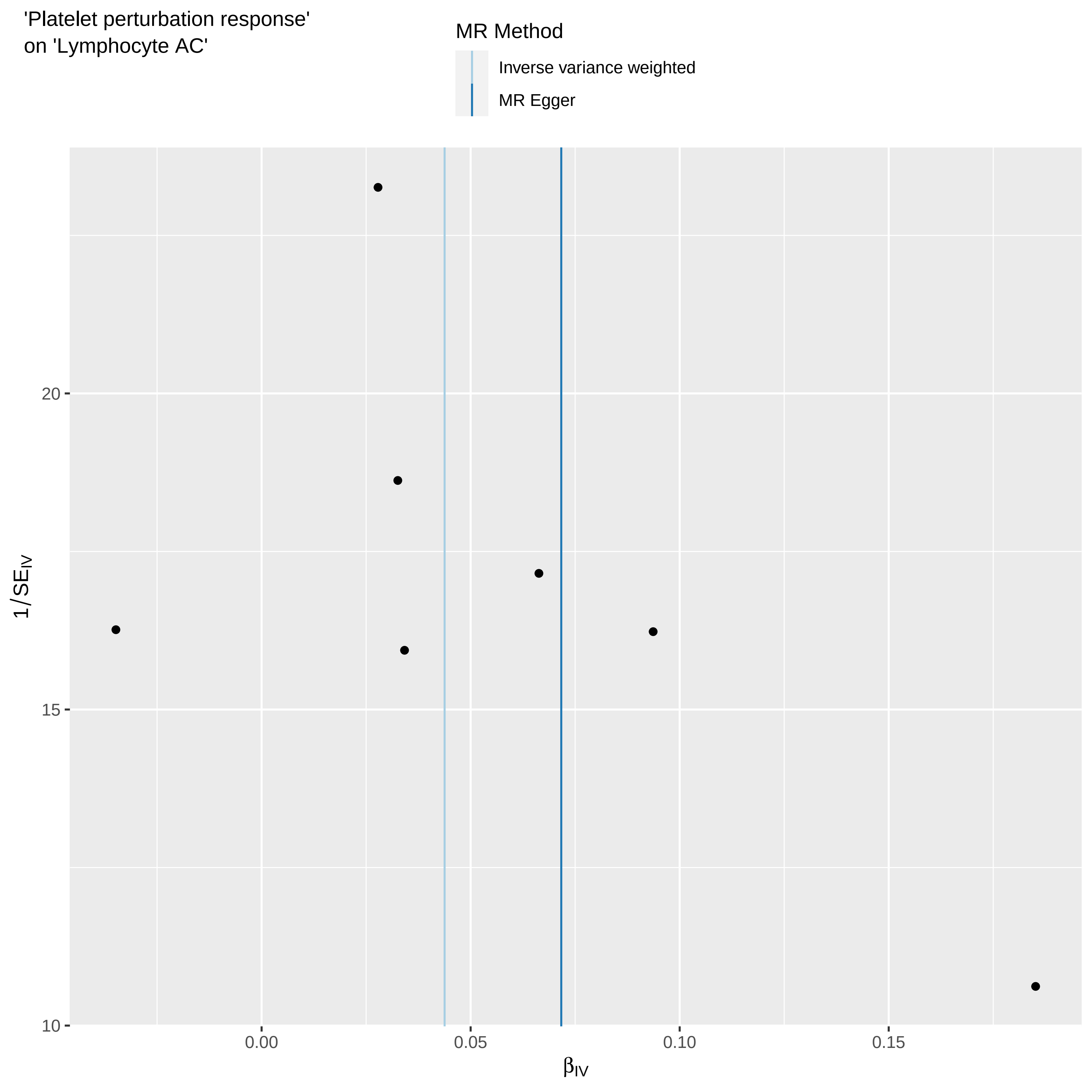

'Platelet perturbation response'
on 'Lymphocyte AC'
MR Method
Inverse variance weighted
MR Egger
20
15
10
1/SE$_{IV}$
0.00
0.05
0.10
0.15
β$_{IV}$

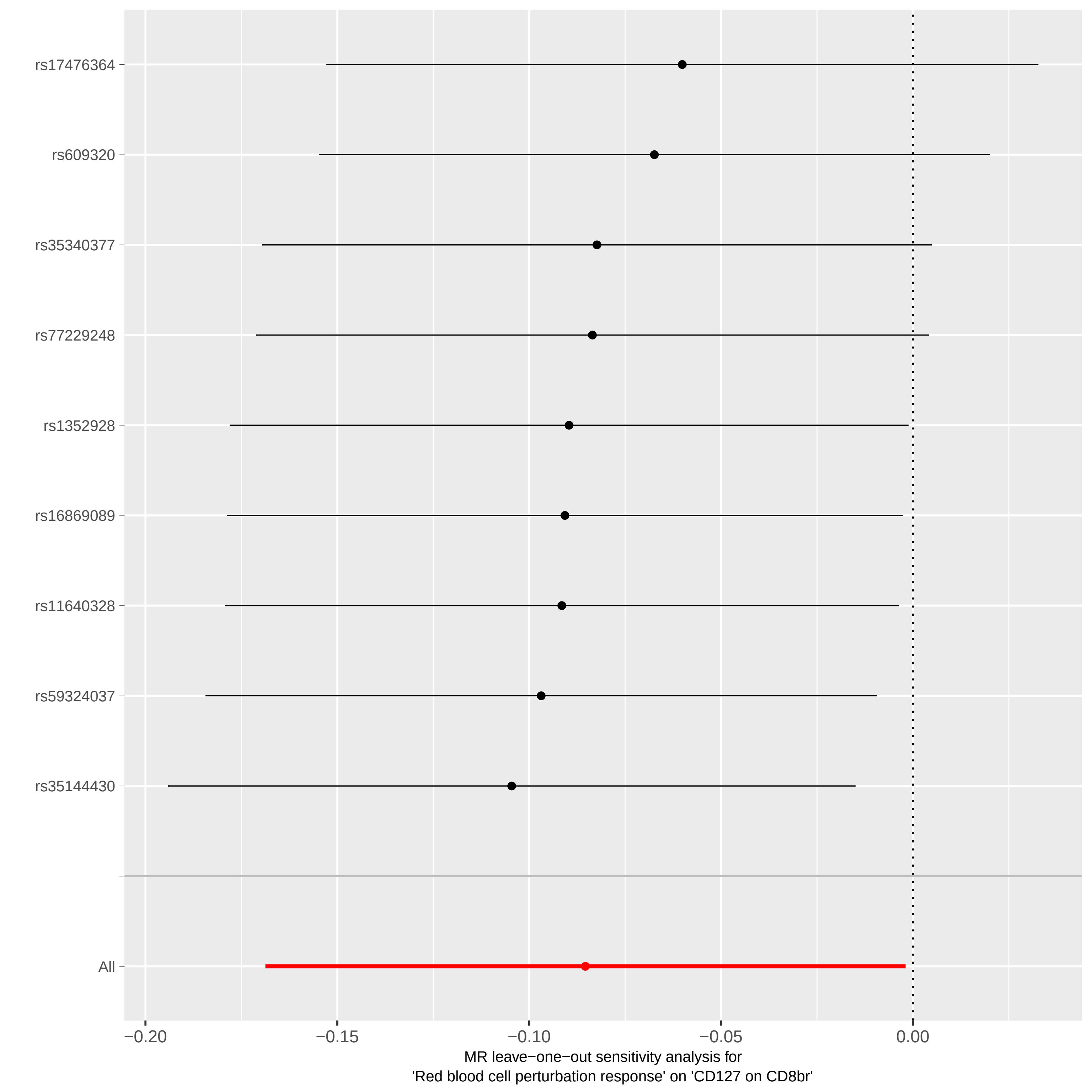

rs17476364
rs609320
rs35340377
rs77229248
rs1352928
rs16869089
rs11640328
rs59324037
rs35144430
All
−0.20
−0.15
−0.10
−0.05
0.00
MR leave−one−out sensitivity analysis for
'Red blood cell perturbation response' on 'CD127 on CD8br'

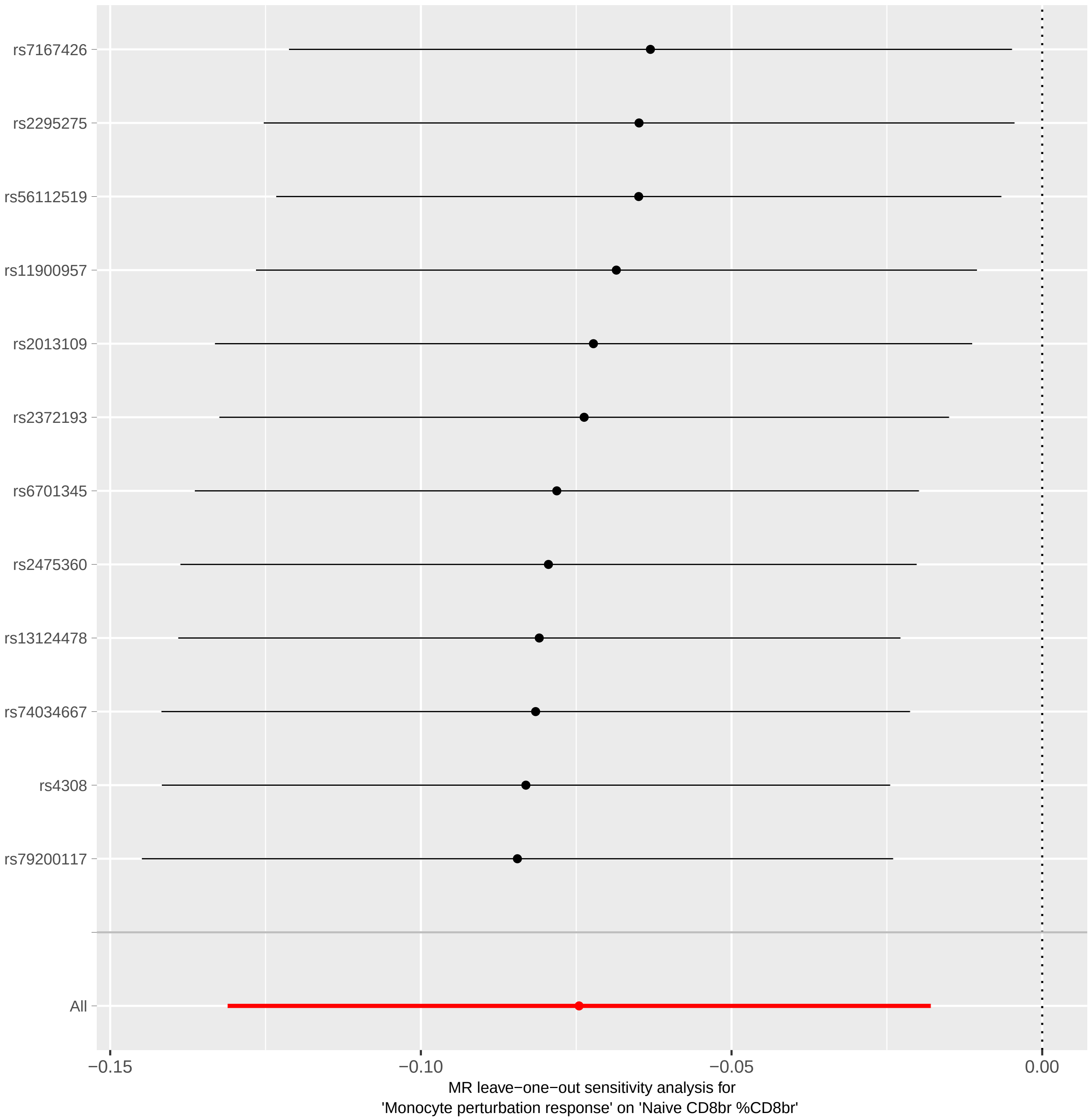
rs7167426
rs2295275
rs56112519
rs11900957
rs2013109
rs2372193
rs6701345
rs2475360
rs13124478
rs74034667
rs4308
rs79200117
All
−0.15
−0.10
−0.05
0.00
MR leave−one−out sensitivity analysis for
'Monocyte perturbation response' on 'Naive CD8br %CD8br'

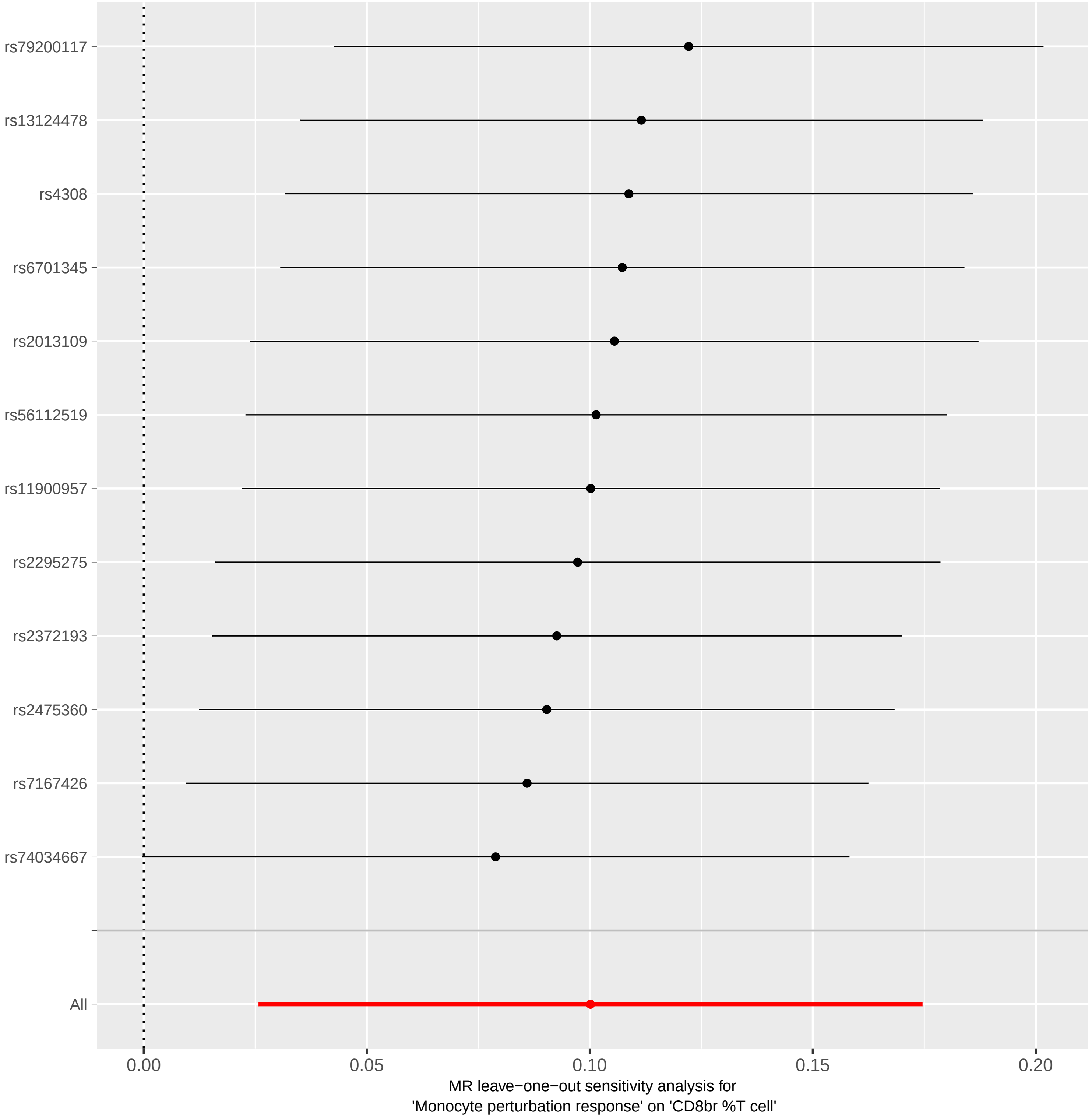
rs79200117
rs13124478
rs4308
rs6701345
rs2013109
rs56112519
rs11900957
rs2295275
rs2372193
rs2475360
rs7167426
rs74034667
All
0.00
0.05
0.10
0.15
0.20
MR leave−one−out sensitivity analysis for
'Monocyte perturbation response' on 'CD8br %T cell'

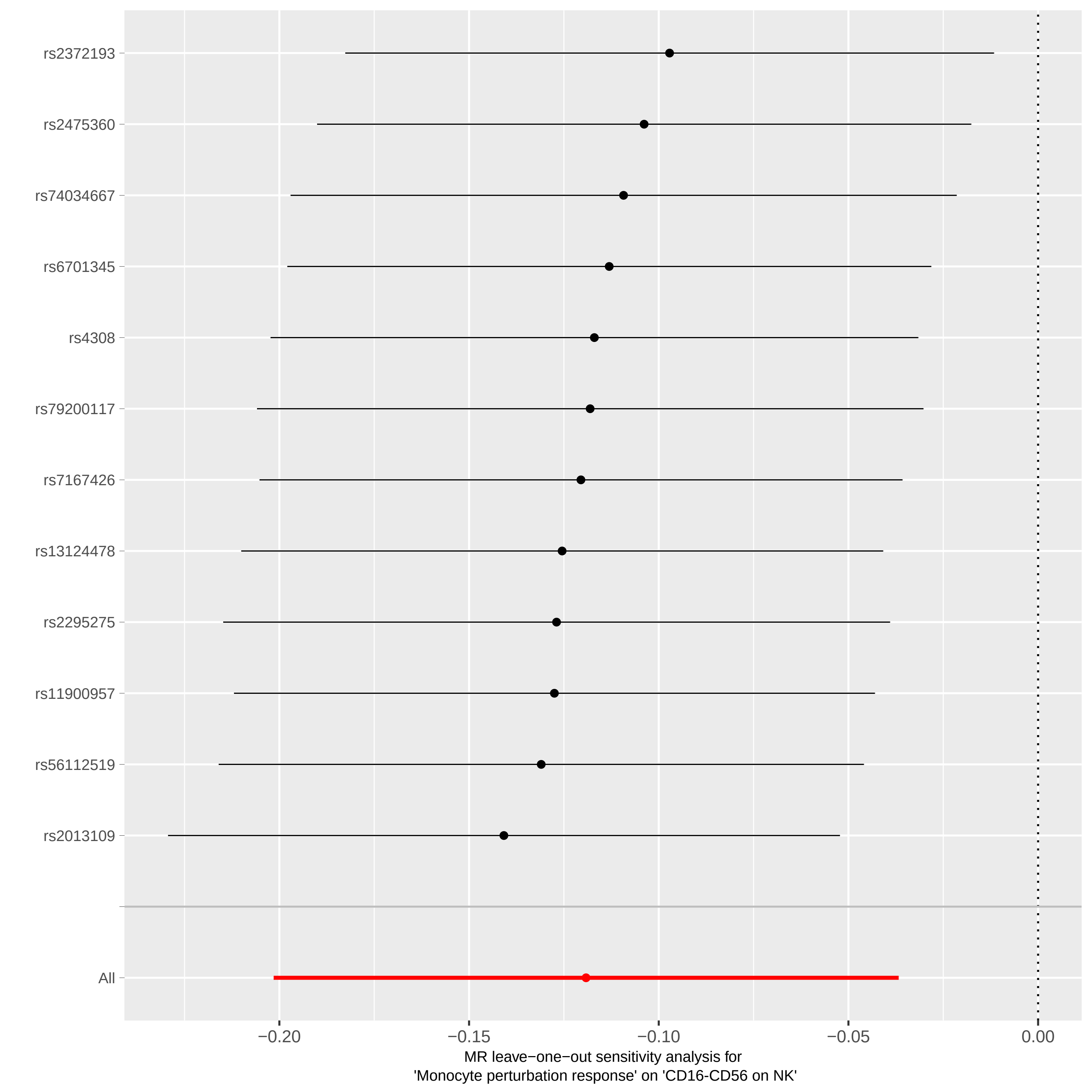

rs2372193
rs2475360
rs74034667
rs6701345
rs4308
rs79200117
rs7167426
rs13124478
rs2295275
rs11900957
rs56112519
rs2013109
All
−0.20
−0.15
−0.10
−0.05
0.00
MR leave−one−out sensitivity analysis for
'Monocyte perturbation response' on 'CD16-CD56 on NK'

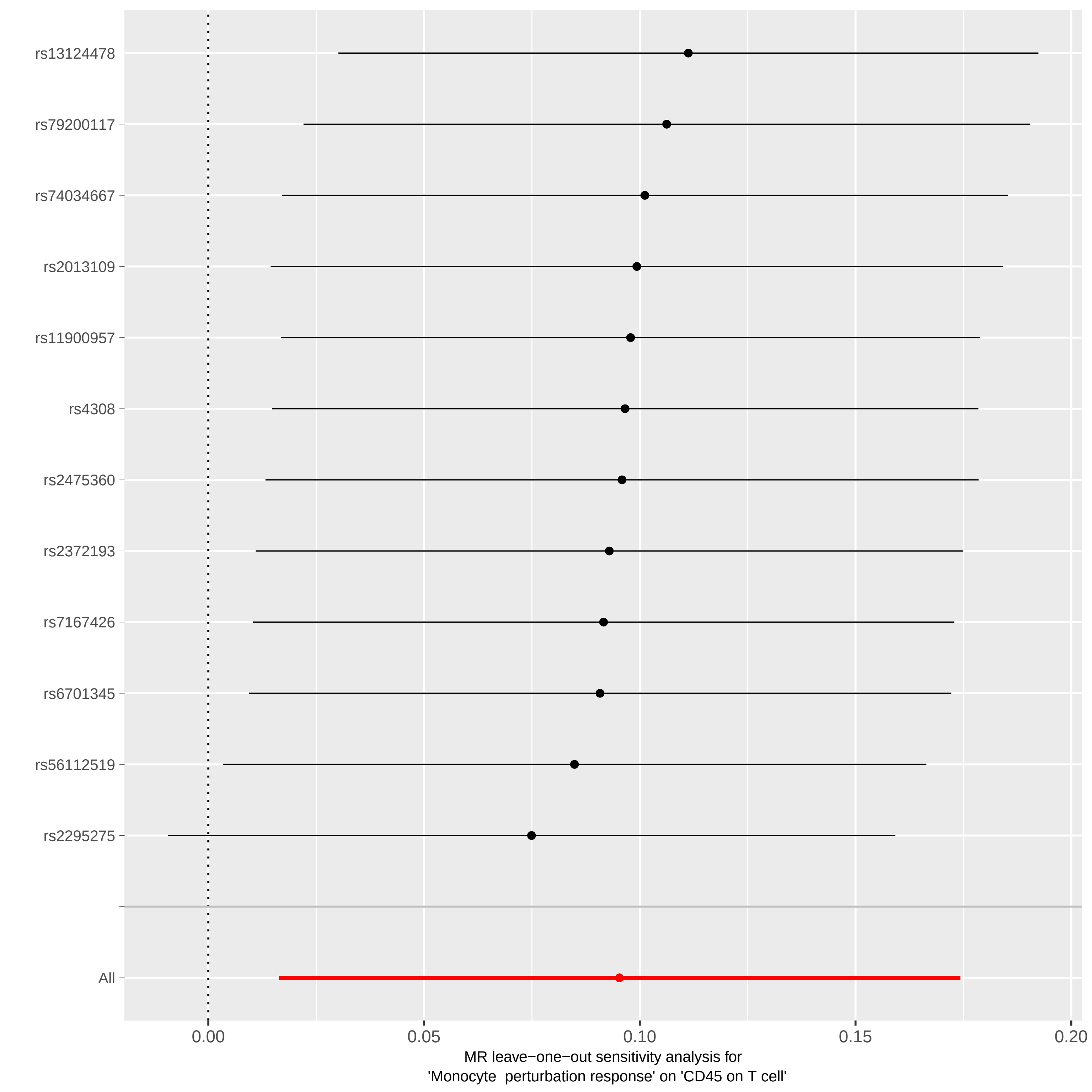
rs13124478
rs79200117
rs74034667
rs2013109
rs11900957
rs4308
rs2475360
rs2372193
rs7167426
rs6701345
rs56112519
rs2295275
All
0.00
0.05
0.10
0.15
0.20
MR leave−one−out sensitivity analysis for
'Monocyte perturbation response' on 'CD45 on T cell'

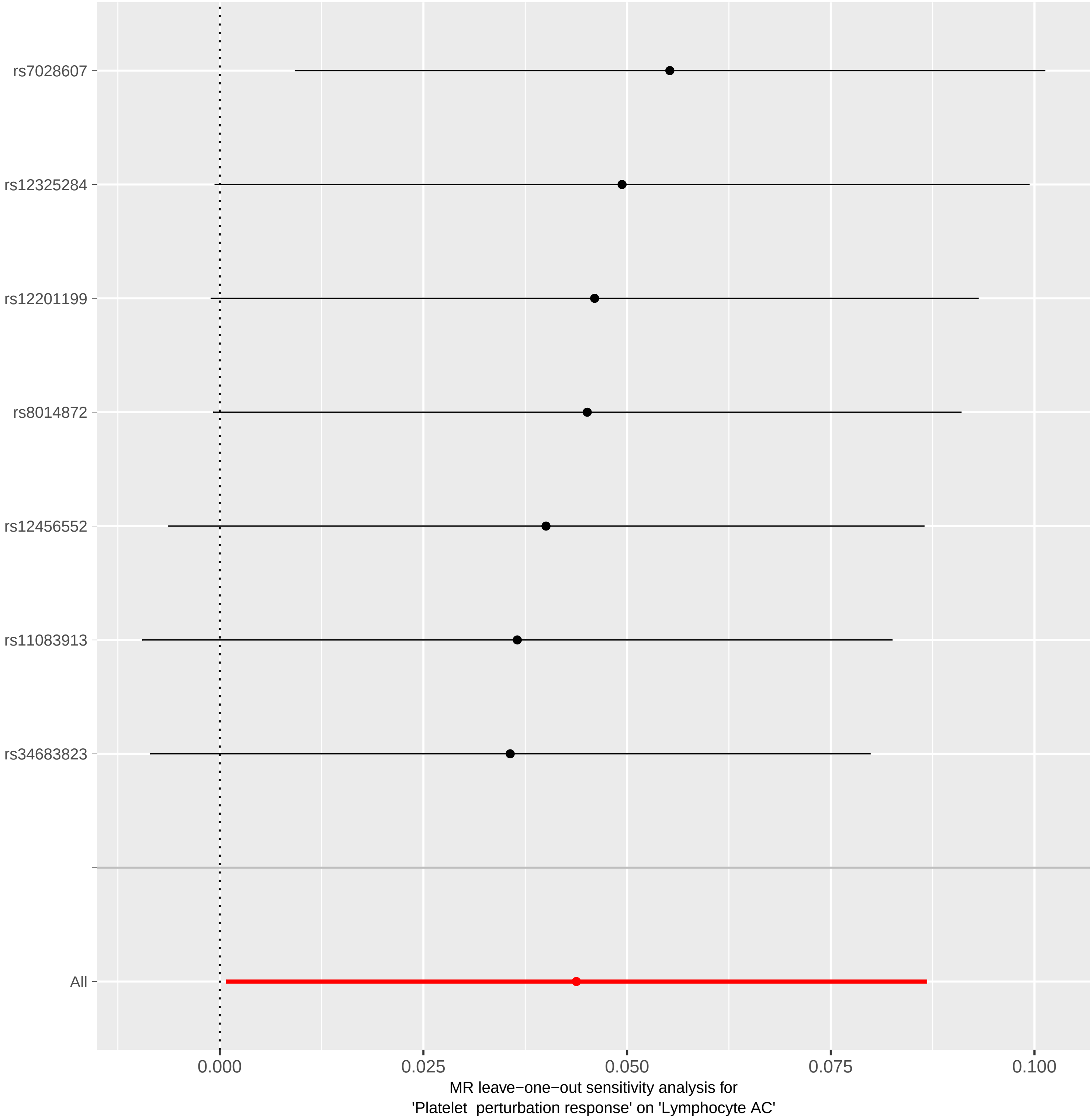
rs7028607
rs12325284
rs12201199
rs8014872
rs12456552
rs11083913
rs34683823
All
0.000
0.025
0.050
0.075
0.100
MR leave−one−out sensitivity analysis for
'Platelet perturbation response' on 'Lymphocyte AC'
